# Evolutionary conservation and enhanced basal immunity of the ZmNBS gene family in maize

**DOI:** 10.3389/fpls.2025.1656786

**Published:** 2025-12-02

**Authors:** Zhe Xu, Liying Feng

**Affiliations:** 1 Institution of Genomics and Bioinformatics, South China Agricultural University, Guangzhou, China; 2 Maize Genome Breeding Team, Yazhouwan National Laboratory, Yazhouwan National Laboratory, Sanya, Hainan, China

**Keywords:** ZmNBS family, evolutionary patterns, basal immunity, structural variation, gene duplication

## Abstract

The nucleotide-binding site (NBS) gene family is central to plant innate immunity. However, a comprehensive understanding of its evolutionary dynamics and functional diversity in maize, particularly within a pan-genomic context, remains limited. We conducted a systematic pan-genomic analysis of the ZmNBS gene family across 26 representative maize inbred lines. Our approach integrated evolutionary genetics, structural variation analysis, and expression profiling to investigate presence-absence variation (PAV), duplication modes, evolutionary rates, and the impact of structural variants (SVs). We observed extensive presence–absence variation (PAV), distinguishing conserved “core” subgroups (ZmNBS31 and ZmNBS17-19) from highly variable ones (ZmNBS1-10 and ZmNBS43-60), thereby supporting a “core-adaptive” model of resistance gene evolution. Duplication mode analysis revealed subtype-specific preferences: canonical CNL/CN genes largely originated from dispersed duplications, while N-type genes were enriched in tandem duplications. Evolutionary rate analysis showed that whole-genome duplication (WGD)-derived genes exhibited strong purifying selection (low Ka/Ks), whereas tandem and proximal duplications (TD/PD) showed signs of relaxed or positive selection. Structural variants (SVs) were associated with altered motif structures and significantly impacted gene expression. Notably, ZmNBS31 emerged as a conserved, highly expressed gene under both stressed and control conditions, underscoring its potential role in basal immunity. Our findings demonstrate how duplication mechanisms, structural variations and differential selection pressures collectively shape the evolution of the ZmNBS gene family. The identification of ZmNBS31 as a candidate for basal immunity, along with our established "core-adaptive" framework, provides valuable insights and a conceptual foundation for identifying and improving broad-spectrum resistance genes in maize breeding programs.

## Introduction

Plants have evolved sophisticated and multilayered immune systems to defend against pathogens during long-term coevolution (Rui et al., 2023; Pieterse et al., 2012). Among these systems, nucleotide-binding site (*NBS*)–leucine-rich repeat (LRR) genes play a pivotal role in effector-triggered immunity (ETI) by recognizing pathogen-derived effectors and initiating downstream immune responses such as hypersensitive cell death (Jones and Dangl, 2006). Typical NBS proteins consist of three domains: a variable N-terminal coiled-coil (CC) or Toll/interleukin-1 receptor-like (TIR) domain, a conserved central NBS domain, and a C-terminal LRR domain. The NBS domain is often used as a defining feature due to its high conservation and functional importance (Meyers et al., 2003).

Maize (*Zea mays*), as one of the world’s most important crops, faces constant biotic stress in the field (Shuyan et al., 2024; Wang et al., 2024). Although previous studies have identified *NBS* genes in reference genomes like B73 (e.g., a genome-wide survey identified 109 *ZmNBS*-encoding genes in B73 with distinct chromosomal distribution and expression profiles), emerging pan-genome resources reveal that many of these immune-related genes exhibit presence/absence variations (PAVs), particularly in non-reference lines (B, 2014; Cheng et al., 2012; Jin et al., 2016; Webb et al., 2002). These variations likely underlie resistance diversity among genotypes and are often overlooked in single-reference analyses (Hufford et al., 2021). The pan-genomic framework thus offers a powerful lens through which to capture the full spectrum of *ZmNBS* gene evolution in maize.

Gene duplication has been recognized as a major force driving the expansion and diversification of *NBS* genes (Ling et al., 2007). Different duplication modes—such as whole-genome duplication (WGD), tandem duplication (TD), proximal duplication (PD), and dispersed duplication (DSD)—not only contribute to gene copy number variation but also influence evolutionary rate and functional fate (Qiao et al., 2019). For instance, TD-derived *NBS* genes often show elevated Ka/Ks ratios, indicative of relaxed or positive selection, while WGD-derived genes tend to be more conserved. Additionally, structural variations (SVs), which are widespread in plant genomes, can alter gene function by disrupting regulatory elements or coding sequences (Man et al., 2024; Sun et al., 2023). However, the combined impact of duplication modes and SVs on the evolution and functional plasticity of *NBS* genes remains poorly understood.

Based on the maize pan-genome, this study systematically identified the members of the *ZmNBS* gene family and integrated orthogroup clustering, PAV analysis, duplication type classification, Ka/Ks evolutionary rate estimation, and gene expression profiling to reveal the structural evolutionary patterns of *ZmNBS* genes under different duplication mechanisms. Furthermore, we explored how SVs regulate their expression and immune response functions. This research provides a theoretical foundation and methodological framework for the discovery and targeted improvement of core disease resistance genes in maize.

## Material and methods

### Identification of maize *ZmNBS* gene family

The data for the 26 maize pan-genomes were obtained from the study by Hufford et al. (2021). Protein domains were identified using HMMER v3.1 with an E-value threshold of 1e−15 to search against the following Pfam profiles: PF00931 (NB-ARC), PF01582 (TIR), PF05659 (RPW8), and PF00560/PF07725/PF12799 (LRR) (Ma et al., 2021; Sun et al., 2014; Wang et al., 2020). In addition, the presence of CC domains was examined using EMBOSS v6.6.0. The integrity of the identified domains was further validated using the Conserved Domain Database (CDD) search tool provided by NCBI (Wang et al., 2023). Custom scripts were used to extract and calculate molecular weight, isoelectric point, and other protein characteristics (**Supplementary Table S1**).

### *ZmNBS* presence–absence variation analysis

The presence–absence variation information of *ZmNBS*s was obtained from (Hufford et al., 2021). The ggplot2 package in R was used to construct the PAV heatmap.

We performed correlation analysis between the PAV frequency (PAV frequency = number of lines containing the gene/total number of lines) and Ka/Ks ratios of *ZmNBS* gene subfamilies. We conducted the analysis using the cor.test function in the R software (version 4.3.2), employing Pearson’s correlation coefficient. Prior to correlation analysis, we filtered extreme Ka/Ks values (Ka/Ks > 5) that may represent computational artifacts or recently duplicated genes undergoing strong positive selection, as such outliers can disproportionately influence correlation estimates.

### Phylogenetic analysis of *ZmNBS*s

The classification of the ZmNBS subfamily is based on the research by Hufford et al. (2021). The conserved domains of *ZmNBS* subfamilies were used for phylogenetic tree construction. To generate a robust and interpretable phylogenetic tree from the large set of 1,457 genes, a representative sequence from each of the 129 identified subfamilies was selected based on the integrity and conservation of the NBS domain. Multiple sequence alignment of the conserved domains was performed using MUSCLE, followed by model selection using IQ-TREE v1.6.9 to determine the best-fit evolutionary model (Nguyen et al., 2015). A maximum likelihood (ML) phylogenetic tree was then constructed with 1,000 bootstrap replicates. The resulting tree was visualized and refined using TVBOT (Xie et al., 2023).

### Ka/Ks calculation and replication type analysis of *ZmNBS*s

To investigate the evolutionary dynamics of *ZmNBS* genes, two approaches were employed to calculate Ka/Ks values.

The protein sequences of sorghum (*Sorghum bicolor*) were used as an outgroup reference (https://sorghum.genetics.ac.cn/SGMD/Genome.html) (**Supplementary Table S2**). Orthologous gene pairs between sorghum and maize were identified through protein sequence alignment, and the corresponding Coding sequence (CDS) pairs were used to calculate Ka and Ks values using the KaKs_Calculator. The replication type of *ZmNBS* was analyzed based on DupGen-finder (**Supplementary Table S2**).

Based on the ZmNBS protein and CDS sequences obtained from the 26 maize genomes reported by Hufford et al. (2021), pairwise comparisons among *ZmNBS* gene copies were performed (**Supplementary Table S3**). After aligning coding sequences, Ka and Ks values were estimated using the KaKs_Calculator to assess selection pressure among intraspecific paralogs (Wang et al., 2010). The pheatmap and ggplot2 packages were used for subsequent plotting.

### Analysis of the expression of *ZmNBS*s overlapped with SVs

Structural variation (SV) data were obtained from the study conducted by Hufford et al. (2021) using the B73 genome as the reference for SV identification. Structural variants were annotated using ANNOVAR (Wang et al., 2010), and those associated with *ZmNBS* gene family members were subsequently extracted. Correlation analysis was then performed between structural variation and gene expression levels of *ZmNBS* genes. For genes showing statistically significant expression differences associated with SV presence, bar plots were generated to visualize their expression levels. Subsequently, the maize accession with the highest number of *ZmNBS* genes overlapping structural variation regions was selected for comparative analysis with the reference genome. MEME Suite v5.5.8 (https://meme-suite.org/meme/tools/meme) was employed to analyze and compare conserved motifs between the ZmNBS proteins of this accession and those from the reference genome (Bailey et al., 2009). Subsequently, the InterProScan 5.76-107.0 program was used to perform functional domain annotation for all identified motifs, with databases including Pfam, SMART, CDD, and PRINTS. The functional annotations obtained through this process were used for the subsequent analysis of how structural variations affect protein function.

### RNA-seq data analysis

RNA-seq data of maize B73 leaves subjected to *Spodoptera litura* feeding for 0, 6, and 12 h (Project ID: PRJCA003103) were used to analyze the expression patterns of *ZmNBS* genes. Low-quality reads were filtered using fastp (Chen et al., 2018), and the clean reads were aligned to the B73 reference genome using HISAT2 (Kim et al., 2019). Gene-level read counts were obtained using HTSeq (Putri et al., 2022), and differentially expressed genes (DEGs) were identified with the criteria of |log_2_FoldChange| > 1 and False discovery rate (FDR) ≤ 0.05. Heatmaps of *ZmNBS* gene expression were visualized using the ComplexHeatmap package (v2.6.2) in R (Gu et al., 2016). Finally, the annotation files of the differentially expressed genes were extracted using custom scripts, and gene structure diagrams were generated using GSDS v2.0 (http://gsds.gao-lab.org/) (Hu et al., 2015).

## Results

### Genome-wide identification and phylogenetic classification of *ZmNBS* genes based on the maize pan-genome

**Figure 1:**
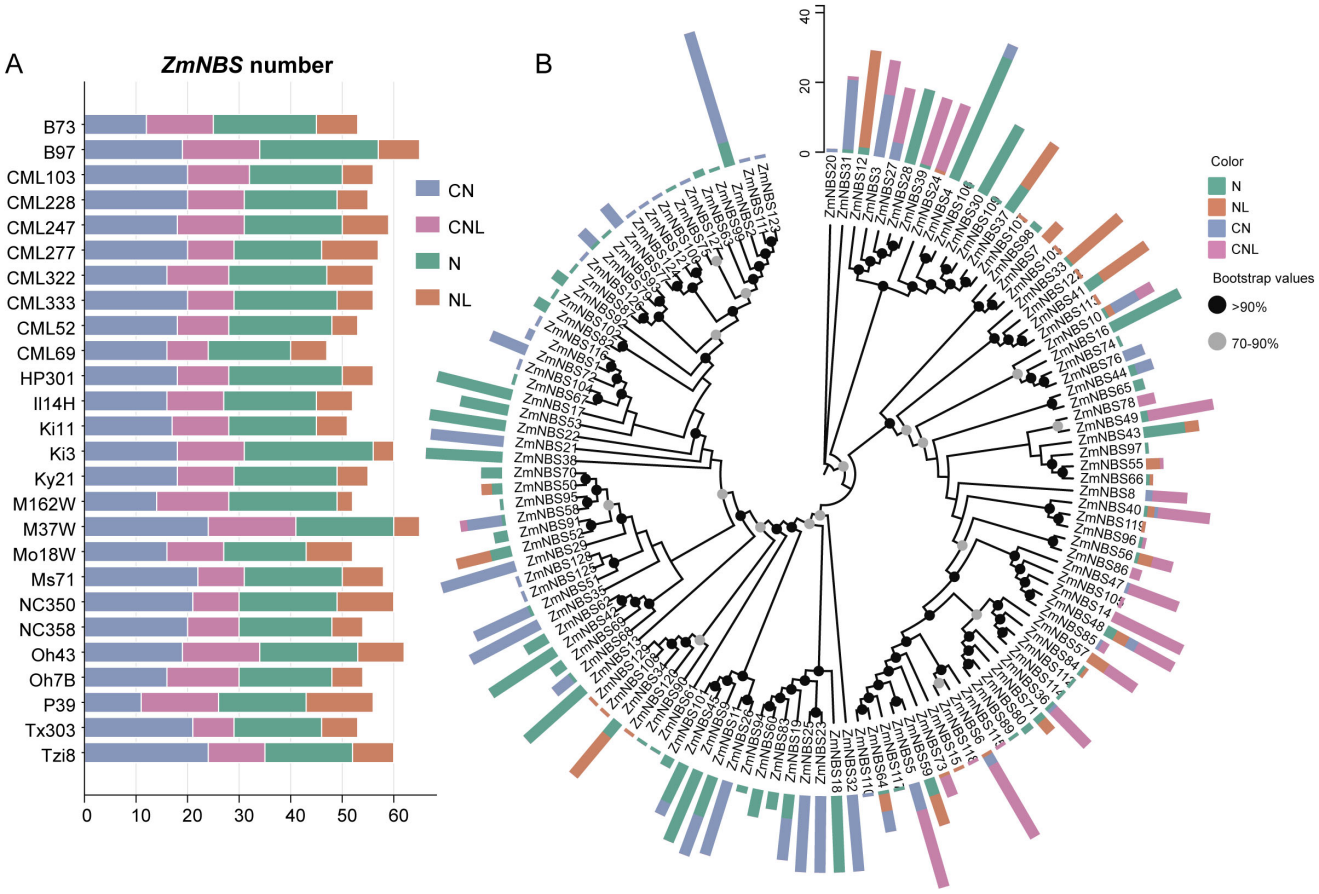
Phylogenetic classification of maize *ZmNBS*. **(A)** Distribution of *ZmNBS* in different varieties of maize. **(B)** Phylogenetic tree of *ZmNBS* subfamily.

In total, we identified 1,457 *ZmNBS* genes across the maize pan-genome, including 53 genes from the reference genome. Among the diverse inbred lines, B97, M37W, and Oh43 harbored notably more *ZmNBS* genes than other genotypes (**Figure 1A**). Using domain annotation tools such as Pfam, we classified all *ZmNBS* genes into four structural types (**Table 1**): CN, CNL, N, and NL. Consistent with previous findings, we identified no TIR or RPW8 domain-containing *ZmNBS* genes in maize (Lan et al., 2019; Li et al., 2015). Notably, we found that 775 genes contained the CC motif, which is significantly more than the 491 genes carrying LRR motifs, highlighting the overrepresentation of CC-type ZmNBS proteins in maize. A phylogenetic analysis of 129 full-length ZmNBS protein sequences revealed that N and CN types tended to cluster together within the same clade. Interestingly, we also found CNL-type members nested within this group, suggesting potential structural convergence or recent domain fusion events (**Figure 1B**).

**Table 1:** Distribution of *ZmNBS* gene types in the maize pan genome.

ZmNBS type	Abbreviation	Number
CC–NBS	CN	474
CC–NBS–LRR	CNL	301
TIR–NBS	TN	0
TIR–NBS–LRR	TNL	0
NBS-only	N	492
NBS–LRR	NL	190
RPW8–NBS–LRR	RNL	0

NBS, nucleotide-binding site; CC, coiled-coil; LRR, leucine-rich repeat; TIR, Toll/interleukin-1 receptor-like.

### PAV indicates genomic reshaping of *ZmNBS* genes

**Figure 2:**
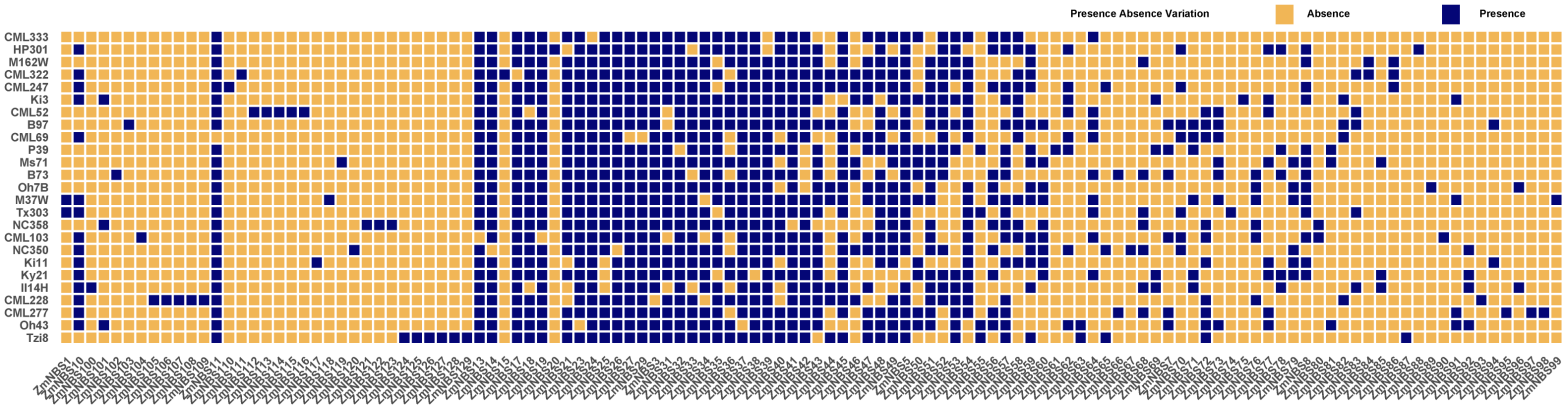
PAV analysis of *ZmNBS* genes across maize pan genome. Blue and orange squares represent subfamily presence and absence, respectively, across different maize lines. Each column represents a *ZmNBS* subfamily, and each row a maize line. The distribution highlights core and variable *ZmNBS* members among lines. PAV, presence–absence variation.

We explored PAV patterns of *ZmNBS* genes across diverse maize inbred lines and observed that a significant portion of *ZmNBS* loci were variably present or entirely missing in certain genotypes (**Figure 2**), reflecting the high plasticity and genomic dynamism of the *ZmNBS* gene family.

Notably, several subfamilies—including *ZmNBS11*, *ZmNBS13-14*, *ZmNBS17-19*, and *ZmNBS21-42*—showed strong conservation and were retained across the majority of genotypes, suggesting that these genes may perform fundamental basal immune functions. In contrast, subfamilies such as *ZmNBS1-10*, *ZmNBS12*, *ZmNBS15-16*, and *ZmNBS43–60* displayed strong genotype-specific absences, with some genes restricted to only one or two inbred lines. This implies that these variable genes may be involved in lineage-specific adaptive responses.

Additionally, considerable differences in overall *ZmNBS* gene retention were observed across genotypes. For instance, CML247, Ki3, B97, and HP301 harbored substantially more *ZmNBS* copies, whereas M162W and Tzi8 retained fewer *ZmNBS* genes. These patterns may reflect distinct selection histories or genetic bottlenecks associated with specific breeding lineages (Catlin et al., 2025).

To quantitatively assess the relationship between gene conservation and evolutionary constraint, we calculated the PAV frequency for each *ZmNBS* subfamily and correlated it with the mean Ka/Ks ratio. After filtering out extreme Ka/Ks values (Ka/Ks > 5) that may represent computational artifacts or recent duplications under strong positive selection, we observed a negative correlation between PAV frequency and Ka/Ks (r = −0.151, p = 0.189) (**Supplementary Figure S1**). Although not statistically significant, this trend suggests that core *ZmNBS* subfamilies (high PAV frequency) tend to evolve under stronger purifying selection (lower Ka/Ks), while variable subfamilies (low PAV frequency) may experience relaxed selection or positive selection (higher Ka/Ks). This pattern is consistent with the core-adaptive model of resistance gene evolution.

### Duplication modes and Ka/Ks analysis of orthologous genes reveal evolutionary constraints

**Figure 3:**
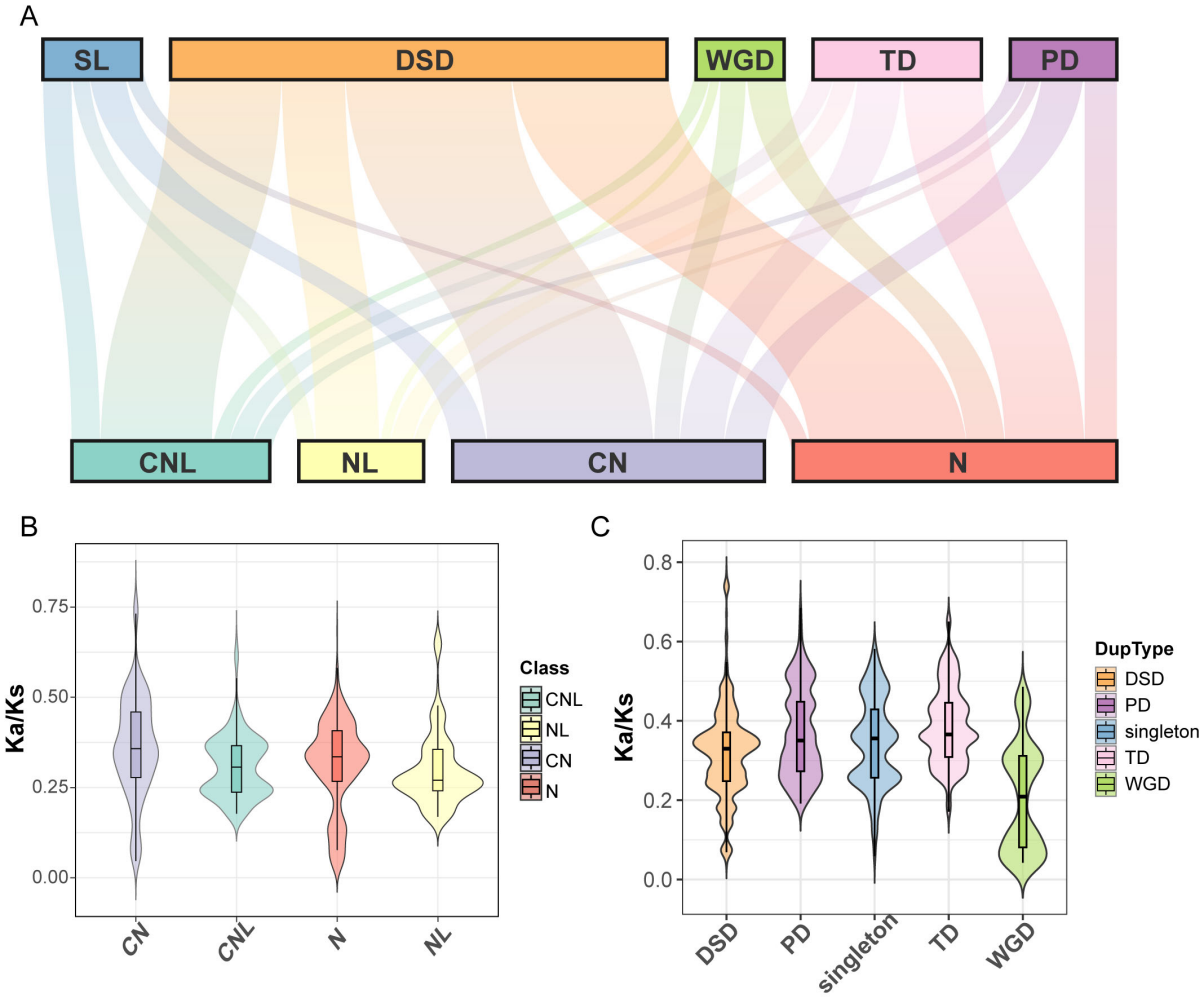
Duplication and structural classification drive *ZmNBS* gene evolution in maize. **(A)** Mapping relationship between *ZmNBS* gene subclasses and their duplication type. **(B)** Distribution of Ka/Ks values among different *ZmNBS* structural types. **(C)** Distribution of Ka/Ks values across different gene duplication modes within *ZmNBS* genes.

To explore the evolutionary constraints of the *ZmNBS* gene family, we first identified the duplication modes of *ZmNBS* genes (1,457) in all the maize lineage using the sorghum genome as an outgroup (**Figure 3A**). The statistical analysis of duplication modes indicates that DSD was the dominant mechanism for the expansion of the ZmNBS gene family, driving the formation of significantly more genes (755) than any other mode. TD, PD, and WGD accounted for 256, 162, and 134 genes, respectively. The analysis revealed a significant association between the structural classification of *ZmNBS* subfamilies and their duplication modes. Specifically, CNL- and NL-type genes are mainly derived from DSD; in contrast, the expansion of N-type genes is primarily driven by TD and DSD, indicating that these mechanisms play a more prominent role in the proliferation of non-canonical *ZmNBS* gene structures.

Subsequently, we evaluated the long-term selective pressures acting on *ZmNBS* genes in the maize lineage by calculating the Ka/Ks ratios between maize *ZmNBS* genes and their sorghum orthologs. The results showed significant differences in selective pressures among genes with different structural types (**Figure 3B**) and different duplication modes (**Figure 3C**).

At the gene structural level, the orthologous Ka/Ks ratios of CN-type genes were significantly higher than those of CNL, N, and NL types, indicating that CN-type genes may have experienced stronger adaptive selection during evolution. At the duplication mode level, compared with genes generated via WGD, genes derived from TD and PD had significantly higher Ka/Ks ratios with their sorghum orthologs. This suggests that genes of TD/PD origin may have an average faster evolutionary rate throughout their evolutionary history since divergence from the common ancestor and have been subjected to positive selection or relatively relaxed functional constraints. In contrast, genes derived from WGD exhibited the lowest Ka/Ks ratios, strongly implying that they have undergone continuous purifying selection throughout evolution, thereby being more likely to retain important core biological functions.

Taken together, these results collectively support the hypothesis that “duplication mode shapes structure, which in turn influences function”. Our analysis reveals an association between two evolutionary processes operating on different timescales: first, the specific duplication mode that a gene undergoes in the early stages of the maize lineage predetermines the domain composition of its encoded protein (e.g., CNL and N types). Subsequently, this inherent structural feature shapes the selective pressure the gene experiences during subsequent long-term evolution (since the divergence of maize and sorghum)—a pattern reflected in the Ka/Ks ratios between the gene and its sorghum orthologs. Therefore, the duplication origin of a gene, by influencing its structure, ultimately shapes the evolutionary trajectory it exhibits in cross-species comparisons.

### Structural variants affect *ZmNBS* genes

We systematically identified the types of SVs in *ZmNBS* genes and their potential impacts. We detected multiple SVs in *ZmNBS* genes, primarily deletions and insertions. Most of these SVs are located in intergenic regions and may indirectly affect gene expression through regulatory sequences. A more critical finding, however, is that we identified SVs that can directly disrupt the coding structure of *ZmNBS* genes. For example, an insertion was found in the exon region of gene *Zm00001eb015450*; this variation is predicted to directly disrupt protein integrity and function, providing evidence that SVs directly contribute to the functional evolution of *ZmNBS* genes (**Supplementary Table S4**).

**Figure 4:**
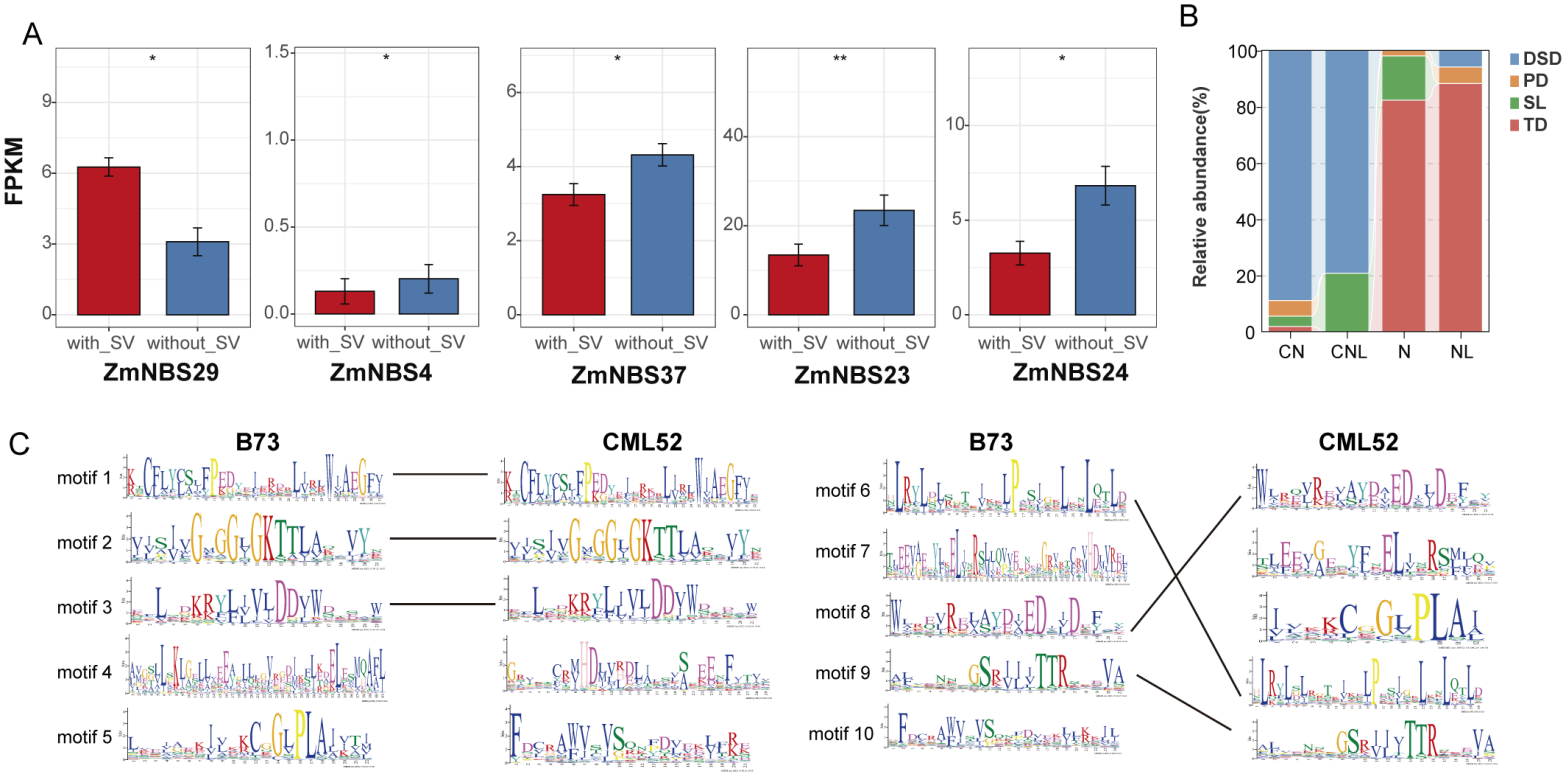
Structural variation and replication history shape the expression regulation and functional structure of *ZmNBS* genes. **(A)** Structural variants influence expression of *ZmNBS* genes. **(B)** Duplication type composition of *ZmNBS* subfamily. **(C)** Motif comparison between the reference genome B73 and the genome with the highest overlap with SV. SV, structural variation. * p< 0.05, ** p<0.01

Furthermore, we analyzed the impact of SVs on gene expression. By comparing the expression profiles of genes with and without nearby SVs, we investigated whether structural variations affect gene expression. Several *ZmNBS* subfamilies, such as *ZmNBS23* and *ZmNBS24*, showed markedly reduced expression in SV-positive lines (**Figure 4A**). Meanwhile, **Figure 4C** displays the differences in motif composition between SV-affected *ZmNBS* genes and reference *ZmNBS* genes, further supporting that SVs may impair functional expression by disrupting core domains. We next investigated the potential molecular mechanisms by which SVs alter gene function beyond expression changes. Functional annotation of SV-associated motifs revealed their direct impact on key protein domains. For instance, Motif5 (IPR001024) and Motif7 (IPR058922), present in CML52 but absent in B73, are predicted to alter subcellular localization for membrane-associated pathogen sensing and facilitate nuclear translocation for defense gene regulation, respectively. Conversely, the B73-specific Motif4 (IPR038005), which is absent in CML52, is a hallmark of resistance proteins like Rx and is critical for oligomerization and immune signalosome assembly. Therefore, these are not neutral variations but represent gains and losses of functional modules that directly drive functional divergence among paralogs, underscoring that SVs are a key mechanism in the adaptive evolution of *ZmNBS* genes.

Among different *ZmNBS* subtypes (CN, CNL, N, and NL), TD and segmental duplication (SL) were more enriched in N and NL types, while CNL and CN types were predominantly expanded through DSD, indicating a preferential association between duplication mechanisms and *ZmNBS* structural subtypes (**Figure 4B**).

### *ZmNBS31* maintains a primed state with high basal expression

**Figure 5:**
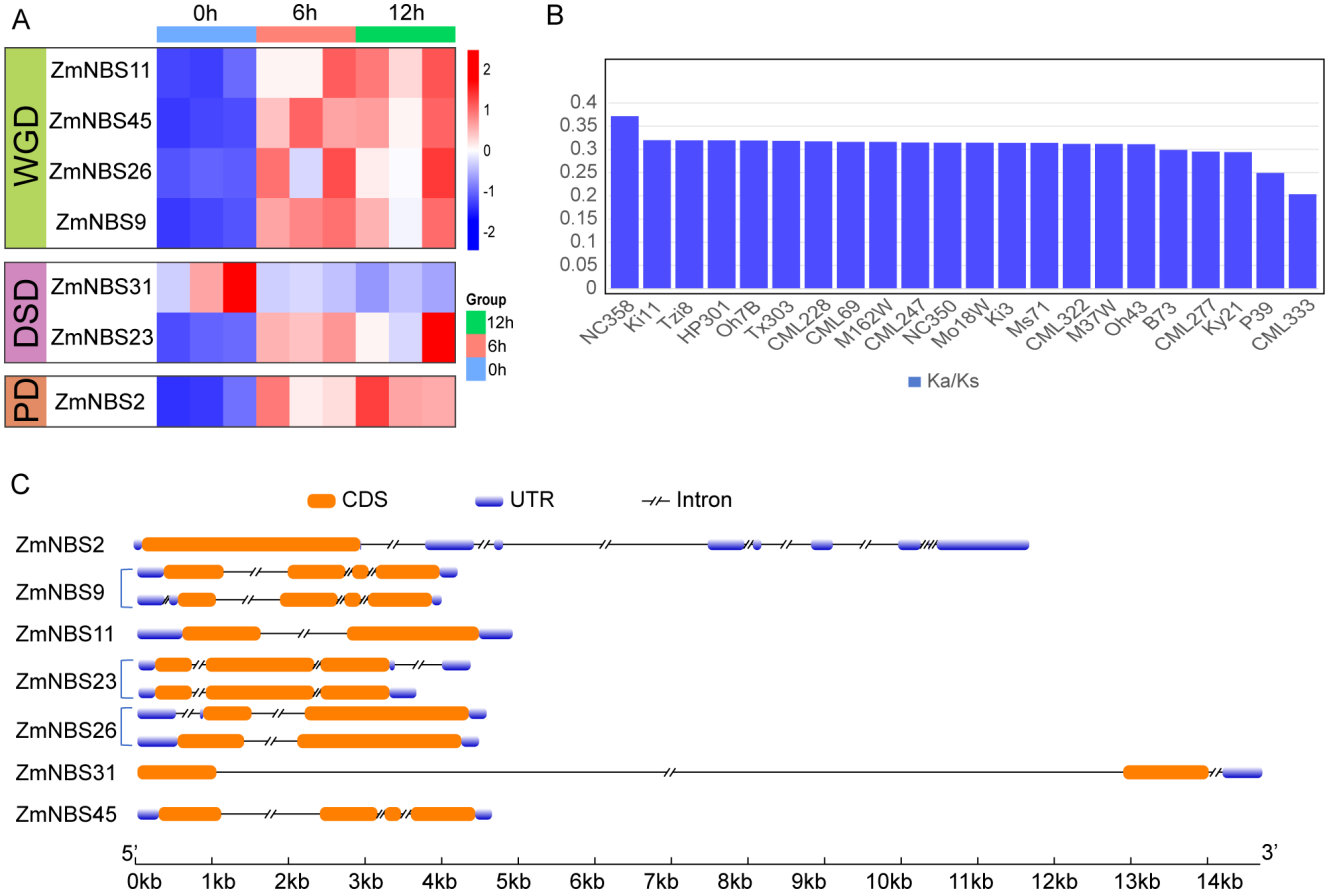
*ZmNBS31* exhibits high basal expression. **(A)** Time-course expression of selected *ZmNBS* genes under insect herbivory simulation. **(B)** Ka/Ks distribution of *ZmNBS31* orthologs across maize line. **(C)** Structural comparison of gene models among *ZmNBS* subfamily members.

To investigate stress-responsive expression, we conducted a time-course analysis following insect herbivory simulation. While many *ZmNBS* genes, *ZmNBS26* and *ZmNBS23*, were transcriptionally upregulated at 6 or 12 h post-treatment, *ZmNBS31* stood out for its high expression even at 0 h (**Figure 5A**). This pattern was consistent across biological replicates, suggesting that *ZmNBS31* maintains a high basal level of expression and may function in early immune surveillance, with its regulation potentially involving a rapid feedback mechanism upon pathogen perception (**Figure 5A**).

Subsequently, we examined the Ka/Ks values of the *ZmNBS31* subfamily across different maize lines. We found that most copies exhibited low Ka/Ks values (average < 0.35), suggesting that this subfamily has been subjected to long-term purifying selection at the population level and retains a high degree of functional conservation (**Supplementary Figure S2**). This finding is consistent with its high expression under pathogen treatment, indicating that *ZmNBS31* members may have been preferentially retained during evolution to maintain basal immune function (**Figure 5B**). To further investigate the selective pressure acting on *ZmNBS31* family members across different maize genomes, we calculated Ka/Ks values for each homolog (**Supplementary Figure S2**). Notably, the lowest Ka/Ks was observed in CML333 (0.2033), indicating that this allele has undergone particularly strong purifying selection. All *ZmNBS31* copies exhibited Ka/Ks < 1, indicating that this gene family has been subject to strong purifying selection and maintains a high degree of functional conservation during evolution (**Supplementary Figure S2**).

Structural comparison across *ZmNBS* subfamilies (**Figure 5C**) revealed that genes derived from WGD and DSD generally possess intact CDS regions and canonical exon–intron structures, whereas PD-derived *ZmNBS2* shows signs of CDS truncation and compressed structure, suggesting possible loss of function due to incomplete architecture. In contrast, *ZmNBS31*, which belongs to the DSD type, exhibits a more complex gene structure with multiple exons and extended introns, which may contribute to its high basal expression level, a configuration that aligns with its role in immediate immune surveillance and subsequent feedback regulation.

## Discussion

In this study, we systematically characterized the evolutionary dynamics, structural diversity, and functional responsiveness of the maize *ZmNBS* gene family in the context of a pan-genomic framework. We observed extensive PAV across diverse inbred lines, particularly in subfamilies such as *ZmNBS1–10* and *ZmNBS43-60*, which were absent in many genotypes, while others like *ZmNBS11*, *ZmNBS17-19*, and *ZmNBS31* were widely retained. This pattern suggests a dichotomy within the family: certain *ZmNBS* subgroups likely function as broadly conserved immune factors, while others may be involved in genotype-specific adaptive responses—supporting a “core-versus-variable” structural paradigm for plant resistance gene families (Kourelis and van der Hoorn, 2018). To validate whether this “core–variable” dichotomy reflects distinct evolutionary constraints, we further analyzed the relationship between gene conservation (measured by PAV frequency) and evolutionary rate (Ka/Ks). Correlation analysis revealed a negative trend consistent with model predictions (r = −0.151).

Further analyses revealed a strong coupling between *ZmNBS* structural subtypes and duplication modes. Canonical CNL and CN genes were predominantly derived from DSDs, whereas N-type genes showed a notable enrichment from TDs. These preferences appear to have not only shaped structural architectures but also influenced evolutionary rates. Ka/Ks analysis showed that WGD-derived genes had the lowest Ka/Ks values, suggesting strong purifying selection and functional conservation (Panchy et al., 2016). Conversely, genes from TD and PD exhibited significantly higher Ka/Ks, reflecting accelerated evolution likely driven by positive selection or relaxed constraints, possibly enabling adaptive novelty (Qiao et al., 2019; Rensing, 2014).

SVs further contributed to functional divergence (Saxena et al., 2014). We found that SVs had a marked impact on gene expression levels and disrupted motif architecture, indicating that SVs can not only affect gene copy number and positioning but also impair expression by altering *cis*-regulatory or coding regions. Plants rely on a two-tiered innate immune system, comprising pattern-triggered immunity (PTI) and ETI, to defend against pathogen invasion. Among these, ETI mediated by Nucleotide-binding Oligomerization Domain-like Receptors (NLR) receptors generally activates a faster and stronger disease resistance response (Jones and Dangl, 2006; Chen et al., 2022). In this context, the expression analysis of *ZmNBS31* revealed that even under untreated conditions (0 h), this gene maintains a high expression level during biotic stress, suggesting that it may be in a constitutively primed state for immune surveillance, thereby enabling a rapid response to pathogen attack. Notably, *ZmNBS31* orthologs displayed consistently low Ka/Ks values across diverse lines, implying evolutionary constraint and functional conservation, consistent with its hypothesized role in basal immunity.

Taken together, the evolution of the *ZmNBS* gene family is shaped by the combined influence of duplication mechanisms, SVs, and selective pressures. TD/PD provides the basis for genetic variability, WGDs help maintain core immune functions, and SVs add a regulatory layer (Puji et al., 2016). The integration of Ka/Ks ratios and expression profiles further reveals functional specialization across different *ZmNBS* subgroups. This composite framework delineates a “core-adaptive” evolutionary model for *ZmNBS* genes, offering both theoretical support and practical guidance for identifying broad-spectrum resistance genes and achieving targeted genetic improvement.

Our integrative analysis of the maize *ZmNBS* gene family reveals both dynamic evolutionary expansion and selective retention of immune components. *ZmNBS31* exemplifies a class of evolutionarily conserved defense genes, characterized by transcriptional readiness, structural robustness, and strong selection constraints. These findings provide new insights into the architecture of plant immunity and offer a genomic target for improving basal resistance in maize.

## Data Availability

The data presented in this study are publicly available. The data can be found here: https://ngdc.cncb.ac.cn, accession PRJCA003103.

## Supplementary material

Supplementary Table 1 Summary of DupType of *ZmNBSs*.

Supplementary Table 2 Ka/Ks value of ZmNBSs (compare with sorghum).

Supplementary Table 3 Ka/Ks value of *ZmNBSs* (Pan-genomic comparisons).

Supplementary Table 4 Structural Variants Identified of *ZmNBS*.

Supplementary Figure 1 Evolutionary Constraint in *ZmNBS* Genes.

Supplementary Figure 2 Ka/Ks profiles of *ZmNBS* genes across 26 maize genomes. **(A)**. Distribution of Ka/Ks ratios of *ZmNBS* gene in 26 maize genomes. **(B)**. Heat map of the frequency of Ka/Ks > 1 for each *ZmNBS* gene in different maize lines.

Supplementary Data Sheet 1 The CDS sequences of the *ZmNBS* genes.

## References

[B1] Bailey T. L. Boden M. Buske F. A. Frith M. Grant C. E. Clementi L. . (2009). MEME SUITE: tools for motif discovery and searching. Nucleic Acids Res. 37, W202–W208. doi: 10.1093/nar/gkp335, PMID: 19458158 PMC2703892

[B2] Catlin N. S. Agha H. I. Platts A. E. Munasinghe M. Hirsch C. N. Josephs E. B. (2025). Structural variants contribute to phenotypic variation in maize. Mol. Ecol., e17662. doi: 10.1111/mec.17662, PMID: 39945381 PMC12717986

[B3] Cheng Y. Li X. Jiang H. Ma W. Miao W. Yamada T. . (2012). Systematic analysis and comparison of nucleotide-binding site disease resistance genes in maize. The FEBS journal, 279(13), 2431–2443. doi: 10.1111/j.1742-4658.2012.08621.x, PMID: 22564701

[B4] Chen G. Zhang B. Ding J. Wang H. Deng C. Wang J. . (2022). Cloning southern corn rust resistant gene RppK and its cognate gene AvrRppK from Puccinia polysora. Nat. Commun. 13, 4392. doi: 10.1038/s41467-022-32026-4, PMID: 35906218 PMC9338322

[B5] Chen S. Zhou Y. Chen Y. Gu J. (2018). fastp: an ultra-fast all-in-one FASTQ preprocessor. Bioinformatics 34, i884–i890. doi: 10.1093/bioinformatics/bty560, PMID: 30423086 PMC6129281

[B6] Gu Z. Eils R. Schlesner M. (2016). Complex heatmaps reveal patterns and correlations in multidimensional genomic data. Bioinformatics 32, 2847–2849. doi: 10.1093/bioinformatics/btw313, PMID: 27207943

[B7] Hu B. Jin J. Guo A. Y. Zhang H. Luo J. Gao G. (2015). GSDS 2.0: an upgraded gene feature visualization server. Bioinformatics 31, 1296–1297. doi: 10.1093/bioinformatics/btu817, PMID: 25504850 PMC4393523

[B8] Hufford M. B. Seetharam A. S. Woodhouse M. R. Chougule K. M. Ou S. Liu J. . (2021). *De novo* assembly, annotation, and comparative analysis of 26 diverse maize genomes. Sci. (New York N.Y.) 373, 655–662. doi: 10.1101/2021.01.14.426684, PMID: 34353948 PMC8733867

[B9] Jin M. Liu H. He C. Fu J. Xiao Y. Wang Y. . (2016). Maize pan-transcriptome provides novel insights into genome complexity and quantitative trait variation. Scientific Reports, 6, 18936. doi: 10.1038/srep18936, PMID: 26729541 PMC4733048

[B10] Jones J. D. Dangl J. L. (2006). The plant immune system. Nature 444, 323–329. doi: 10.1038/nature05286, PMID: 17108957

[B11] Kim D. Paggi J. M. Park C. Bennett C. Salzberg S. L. (2019). Graph-based genome alignment and genotyping with HISAT2 and HISAT-genotype. Nat. Biotechnol. 37, 907–915. doi: 10.1038/s41587-019-0201-4, PMID: 31375807 PMC7605509

[B12] Kourelis J. van der Hoorn R. A. L. (2018). Defended to the nines: 25 years of resistance gene cloning identifies nine mechanisms for R protein function. Plant Cell 30, 285–299. doi: 10.1105/tpc.17.00579, PMID: 29382771 PMC5868693

[B13] Lan D. Tang L. Li J. Xie P. Lv Y. Xiaoli Q. (2019). NBS-LRR resistance gene in grass species: structure, function and evolution. Chin. Agric. Sci. Bull. 35, 124–127. doi: 10.11924/j.issn.1000-6850.casb18090052

[B14] Li X. Kapos P. Zhang Y. (2015). NLRs in plants. Curr. Opin. Immunol. 32, 114–121. doi: 10.1016/j.coi.2015.01.014, PMID: 25667191

[B15] Ling L. K. Xionglun L. Liangying D. Guoliang W. (2007). Recent progress in elucidating the structure, function and evolution of disease resistance genes in plants. J. Gene. Genomics 9, 765–776. doi: 10.1016/S1673-8527(07)60087-3, PMID: 17884686

[B16] Ma Y. Chhapekar S. S. Lu L. Oh S. Singh S. Kim C. S. . (2021). Genome-wide identification and characterization of NBS-encoding genes in Raphanus sativus L. and their roles related to Fusarium oxysporum resistance. BMC Plant Biol. 21, 47. doi: 10.1186/s12870-020-02803-8, PMID: 33461498 PMC7814608

[B17] Man Q. C. Wang Y. Q. Gao S. J. Gao Z. C. Peng Z. P. Cui J. H. (2024). Pan-genome analysis and expression verification of the maize ARF gene family. Front. Plant Sci. 15. doi: 10.3389/fpls.2024.1506853, PMID: 40007769 PMC11850412

[B18] Meyers B. C. Kozik A. Griego A. Kuang H. Michelmore R. W. (2003). Genome-wide analysis of NBS-LRR-encoding genes in Arabidopsis. Plant Cell 15, 809–834. doi: 10.1105/tpc.009308, PMID: 12671079 PMC152331

[B19] Nguyen L. T. Schmidt H. A. Von Haeseler A. Minh B. Q. (2015). IQ-TREE: a fast and effective stochastic algorithm for estimating maximum-likelihood phylogenies. Mol. Biol. Evol. 32, 268–274. doi: 10.1093/molbev/msu300, PMID: 25371430 PMC4271533

[B20] Panchy N. Lehti-Shiu M. Shiu S. H. (2016). Evolution of gene duplication in plants. Plant Physiol. 171, 2294–2316. doi: 10.1104/pp.16.00523, PMID: 27288366 PMC4972278

[B21] Pieterse C. M. van der Does D. Zamioudis C. Leon-Reyes A. Van Wees S. C. (2012). Hormonal modulation of plant immunity. Annu. Rev. Cell Dev. Biol. 28, 489–521. doi: 10.1146/annurev-cellbio-092910-154055, PMID: 22559264

[B22] Puji L. Suk-Ha L. Tasma I.M. Asadi (2016). Gene duplication to reveal adaptation clue of plant to environmental stress: A case study of NBS-LRR genes in soybean. Jurnal AgroBiogen 12, 119–130. doi: 10.21082/jbio.v12n2.2016.p119-130"10.21082/jbio.v12n2.2016.p119-130

[B23] Putri G. H. Anders S. Pyl P. T. Pimanda J. E. Zanini F. (2022). Analysing high-throughput sequencing data in Python with HTSeq 2.0. Bioinformatics 38, 2943–2945. doi: 10.1093/bioinformatics/btac166, PMID: 35561197 PMC9113351

[B24] Qiao X. Li Q. Yin H. Qi K. Li L. Wang R. . (2019). Gene duplication and evolution in recurring polyploidization-diploidization cycles in plants. Genome Biol. 20, 38. doi: 10.1186/s13059-019-1650-2, PMID: 30791939 PMC6383267

[B25] Rensing S. A. (2014). Gene duplication as a driver of plant morphogenetic evolution. Curr. Opin. Plant Biol. 17, 43–48. doi: 10.1016/j.pbi.2013.11.002, PMID: 24507493

[B26] Rui L. Su J. Y. Li T. Sun J. M. Wu G. H. (2023). Molecular regulation of immunity in tea plants. Mol. Biol. Rep. 50, 2883–2892. doi: 10.1007/s11033-022-08177-4, PMID: 36538170

[B27] B W. L. A. B M. F. Réné Huel B. C. D C. L. N. B. B X. W. A. B D. A. G. . (2014). The Stripe Rust Resistance Gene Yr10 Encodes an Evolutionary-Conserved and Unique CC–NBS–LRR Sequence in Wheat. Molecular Plant, 7(12), 1740–1755. doi: 10.1093/mp/ssu112, PMID: 25336565

[B28] Saxena R. K. Edwards D. Varshney R. K. (2014). Structural variations in plant genomes. Brief Funct. Genomics 13, 296–307. doi: 10.1093/bfgp/elu016, PMID: 24907366 PMC4110416

[B29] Shuyan G. Qingping J. Liyuan H. Xiaotong W. Peng J. Siyan L. . (2024). Advances in biological breeding for adverse stress in maize. J. Jilin Agric. Univ. 46, 1–9. doi: 10.21082/jbio.v12n2.2016.p119-130"10.21082/jbio.v12n2.2016.p119-130

[B30] Sun X. Pang H. Li M. Chen J. Hang Y. (2014). Tracing the origin and evolution of plant TIR-encoding genes. Gene 546, 408–416. doi: 10.1016/j.gene.2014.04.060, PMID: 24786214

[B31] Sun Y. Xiao W. Wang Q. N. Wang J. Kong X. D. Ma W. H. . (2023). Multiple variation patterns of terpene synthases in 26 maize genomes. BMC Genomics 24, 46. doi: 10.1186/s12864-023-09137-3, PMID: 36707768 PMC9881264

[B32] Wang J. Chitsaz F. Derbyshire M. K. Gonzales N. R. Gwadz M. Lu S. . (2023). The conserved domain database in 2023. Nucleic Acids Res. 51, D384–d388. doi: 10.1093/nar/gkac1096, PMID: 36477806 PMC9825596

[B33] Wang T. Feng J. Zhang C. (2024). Research progress on molecular mechanisms of heat stress affecting the growth and development of maize. Bull. Bot. 59, 963–977. doi: 10.11983/CBB24049

[B34] Webb C. A. Richter T. E. Collins N. C. Nicolas M. Hulbert S. H . (2002). Genetic and molecular characterization of the maize rp3 rust resistance locus. Genetics, 162(1), 381–394. doi: 10.1093/genetics/162.1.381, PMID: 12242248 PMC1462242

[B35] Wang K. Li M. Hakonarson H. (2010). ANNOVAR: functional annotation of genetic variants from high-throughput sequencing data. Nucleic Acids Res. 38, e164. doi: 10.1093/nar/gkq603, PMID: 20601685 PMC2938201

[B36] Wang Y. Lu Q. Xiong F. Hao X. Wang L. Zheng M. . (2020). Genome-wide identification, characterization, and expression analysis of nucleotide-binding leucine-rich repeats gene family under environmental stresses in tea (Camellia sinensis). Genomics 112, 1351–1362. doi: 10.1016/j.ygeno.2019.08.004, PMID: 31408701

[B37] Wang D. Zhang Y. Zhang Z. Zhu J. Yu J. (2010). KaKs_Calculator 2.0: a toolkit incorporating gamma-series methods and sliding window strategies. Genom Proteomi Bioinf 8, 77–80. doi: 10.1016/s1672-0229(10)60008-3, PMID: 20451164 PMC5054116

[B38] Xie J. Chen Y. Cai G. Cai R. Hu Z. Wang H. (2023). Tree Visualization By One Table (tvBOT): a web application for visualizing, modifying and annotating phylogenetic trees. Nucleic Acids Res. 51, W587–w592. doi: 10.1093/nar/gkad359, PMID: 37144476 PMC10320113

