# Supplementary figures and images for: Evolutionary conservation and enhanced basal immunity of the ZmNBS gene family in maize
Source: Front Plant Sci. 2025 Dec 2;16:1656786. doi: 10.3389/fpls.2025.1656786 (PMC12705624; doi:10.3389/fpls.2025.1656786)

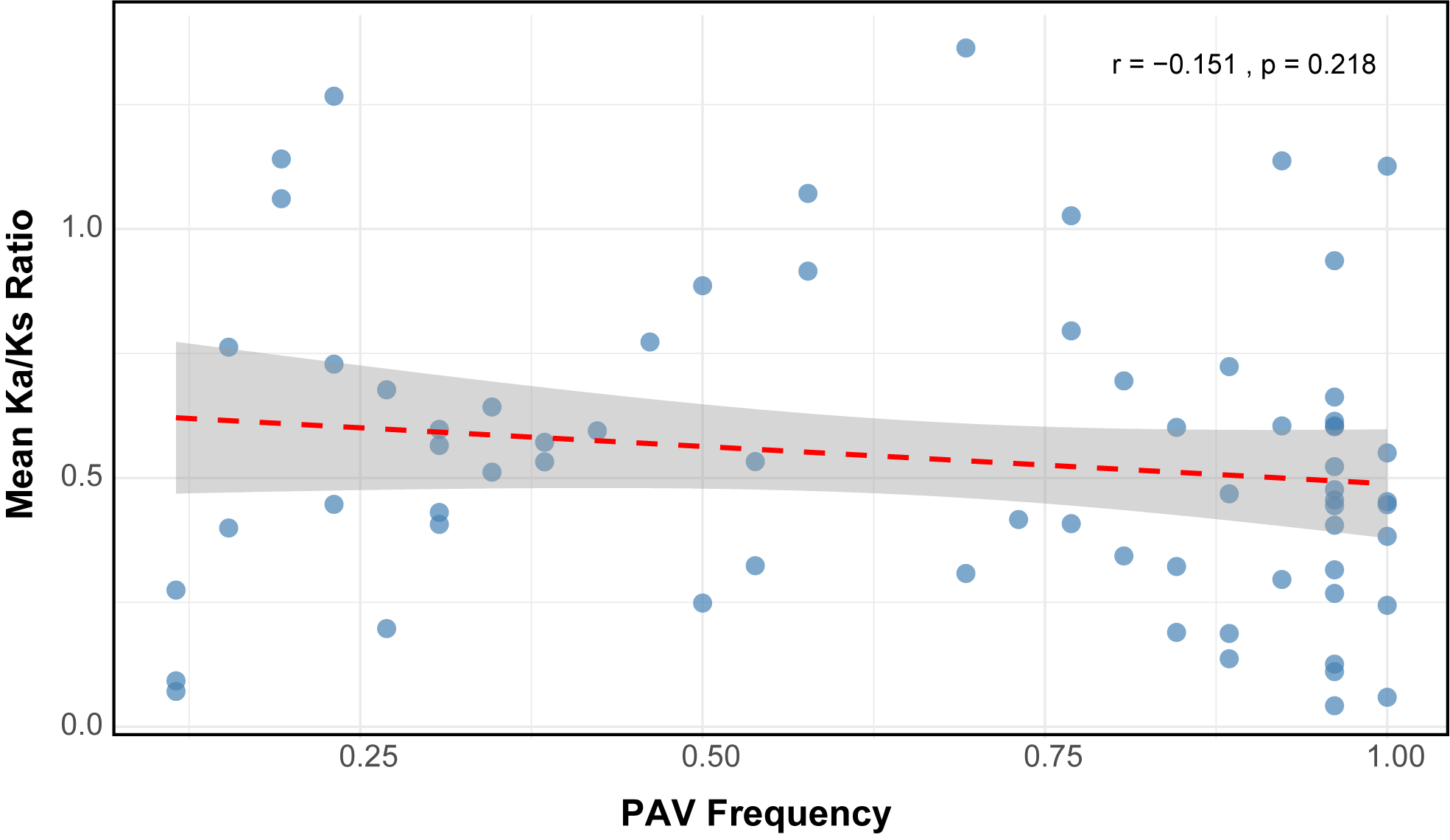

Supplement: Supplementary Figure 1 — Evolutionary Constraint in ZmNBS Genes. [file Image1.tif]

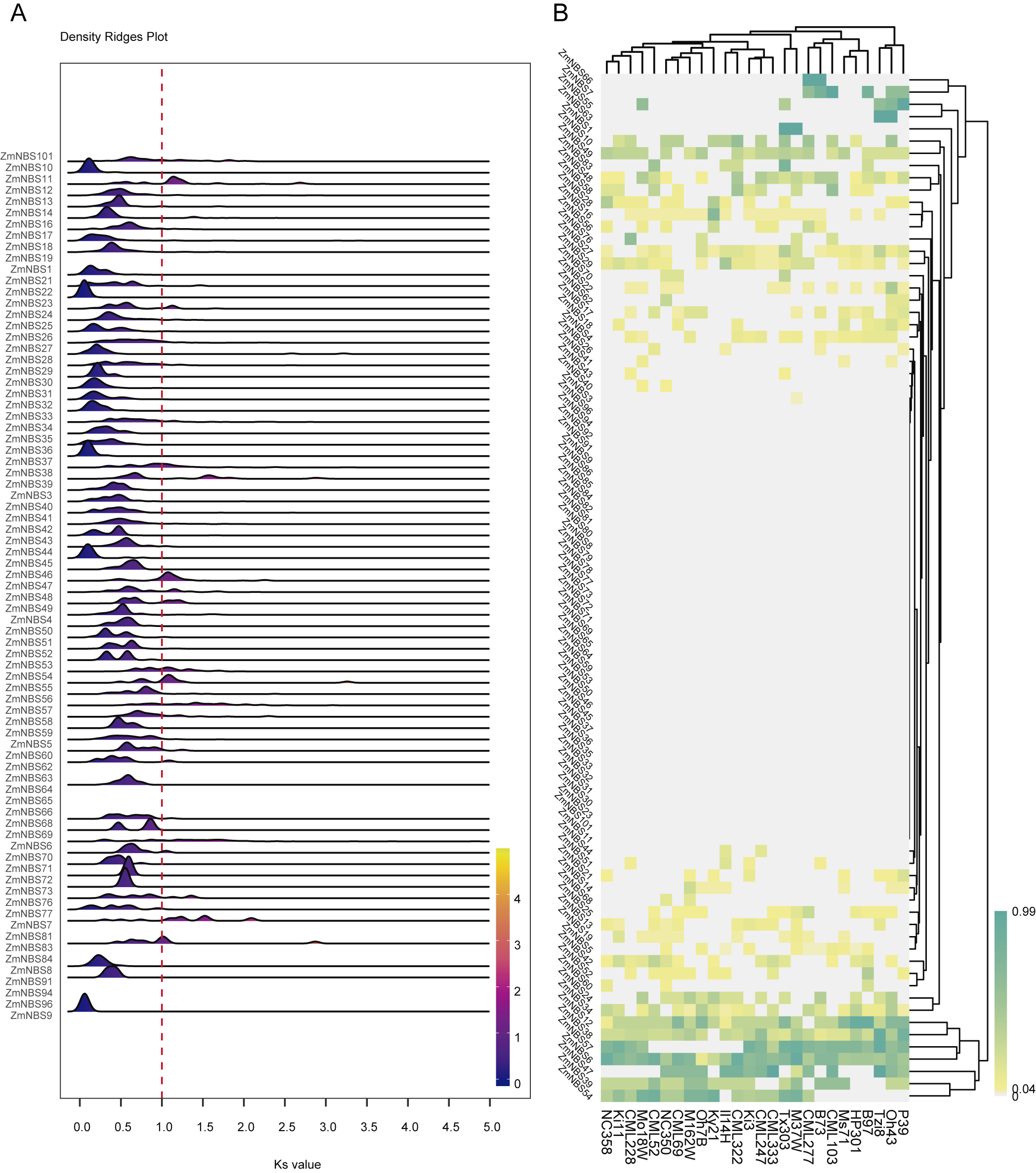

Supplement: Supplementary Figure 2 — Ka/Ks profiles of ZmNBS genes across 26 maize genomes. (A). Distribution of Ka/Ks ratios of ZmNBS gene in 26 maize genomes. (B). Heat map of the frequency of Ka/Ks > 1 for each ZmNBS gene in different maize lines. [file Image2.tif]
