# Supplementary material for: Evolutionary conservation and enhanced basal immunity of the ZmNBS gene family in maize
Source: Front Plant Sci. 2025 Dec 2;16:1656786. doi: 10.3389/fpls.2025.1656786 (PMC12705624; doi:10.3389/fpls.2025.1656786)
Supplement: Supplementary Data Sheet 1 — The CDS sequences of the ZmNBS genes. [file DataSheet1.docx]

Supplementary data 1：The CDS sequences of the *ZmNBS* genes

>B73_Zm00001eb010990

ATGGAGCTACAGCTGTCGGCCGTGCTCGGCTCGCTCGCCCTCGGCGGCGCGGTGCTGGTGCTGTTCTTCGTTAAGTGGTGGCAGCCGCTGGCCGGCACCGACCGGCGCGTCAAGGAGCTTGCAGACGCGGTGGAGGCCCTGCTGCGGCAGCGGTACGAGGTGCTGGGCCACGACCCGGCGCCGTCGTCGGATACCGTGCGCGCGTGGCTGCGGCGCGTGCAGGAGGCGCAGGACGAGATGGCGTCCATCAAGGCGCGGCACGACGGCGGGCAGCTATACGTGGTCCGCCTGGTGCAGTACCTCTTCCTCCCCACGGGCCCGGTCGCGGGGCTGGCCGAGCAGCAGCTCAAGGCAGTGCGCGCGCTCCGAGAGCAGGGCGCCGCGATCCTCGATGCCGCACTGGCCACGCCGCAGGCGCCGCCGCCTCTTCTCTGCGACCCCGAAGAGCTGGAGGGTCTCCCAGCGGAGGCGGGGCCCGCGAGGGCCTACCTCAACGAGGCGCTTCGCTTCCTCGGCGACTGCGACGCCGCGCTTGGCGTCTGGGGTGCCGGCGGCGTGGGCAAGACCACGGTGCTGAAGCTGGTGCGCGAGGTGTGCGGCCGCGTCGCGCGATTCGACCACGTCCTACTCGTCGCGGCCTCCAGGGACTGCACGGTGGCCAAGCTCCAGAGGGAGGTCGTGTCCGTGCTCGGGCTGCGCGACGCGCCCACGGAGCAGGCGCAGGCCGCCGGGATCCTGAGCTTCCTGAGGGACAAGAGCTTCCTGCTGCTGCTGGACAGCGTGTGGGAACGTCTGGACCTGGAGAGGGTCGGCATCCCGCAGCCCCTCGGCATGGCTAACGGCAAGGTGAGGAAGATCATAGTGGCGTCGAGGAGCGAGGCCTTGTGCGCCGACATGGGCTGCCGCAACAAGATCAAGATGGAGTGCTTGAACGAGGAGGATGCGTGGAGCCTGTTTCAAGCTAATGTTGGCGGCGACATCATCCATGGCCACGCTCAAATTCCTGCACTTGCTAAACAGGTCGCTGCCGAATGCAAGTGCTTGCCTTTGGCCCTCGTCACCGTCGGCCGCGCAATGTCAAATAAGCGCACACCAGAGGAGTGGTCCAACGCACTCGACACCCTCAAGGCATCGCTCCGCTCCGGCACGCCCGGCTTGGACAAGAGCACGCAGGCGCTAGTGAAGTTCTGCTACGACAACCTGGAGAGCGACATGGTGAGGGAATGCTTCCTGACCTGTGCGCTATGGCCGGAGGACCACAACATCTCCAAGGAGGAGCTCGTGCAGAGCTGGATAGGACTCGGCCTGCTCCCCGATCTCAGCGACATCGAAGAGGCCCACAGGTTCGGGCTCTCGGTGATTGCCATCATGAAGGCCGCGTGCCTGCTGGAGCCCGGGGACAACCACCGCTACAACATGTTCCCGTCAGACACTCACGTCAGGATGCACGACGTCGTGCGCGACGCGGCGCTCCGGTTCGCGCCCGCCAAGTGGCTGGTCCGCGCAGGCGCTGGGCTCAGGGAGCCCCCGCGCGAGGAGGCGCTGTGGCGGGGCGCGCAGCGCGTGTCCCTGATGCACAACACCATCGAGGACGTGCCGGCGAAGGTGGGTGGCGCCCTCGCGGACGCGCAGCCGGCGTCGCTGATGCTCCAGTGCAACAAGGCCCTGCCGAAGAGGATGCTCCAGGCGATCCAGCATTTCACCAAGCTCACGTACCTGGACCTCGAGGACACCGGCATTCAGGACGCCTTCCCCATGGAGATCTGCTGTTTGGTCAGCTTGAAGCACCTCAACCTATCCAAGAACAAGATCCTGTCGCTGCCGATGGAGCTGGGCAACCTGAGCCAGCTCGAGTACTTCTACCTGCGCGACAACTACTACATCCAGATCACCATACCACCGGGGCTGATCTCGCGGCTTGGGAAGCTGCAGGTGCTGGAGGTTTTCACCGCGAGCATCGTCTCCGTCGCGGACAACTACGTCGCGCCAGTCATTGACGACCTCGAGAGCAGCGGCGCGCGCATGGCGTCGCTCGGCATCTGGCTCGACACCACCCGCGACGTGGAGCGCCTCGCGCGGCTAGCGCCGGGCGTGCGCGCCCGGTCGCTGCACCTGCGCAAGTTAGAAGGGACGCGCGCCCTGCCGCTGCTGTCCGCGGAGCACGCGCCGGAGCTTGCCGGCGTGCAGGAGAGCCTGCGGGAGCTGGTGGTCTACTCCTCCGATGTCGACGAGATCACGGCCGACGCGCACGTGCCCATGCTGGAGGTCATCAAGTTTGGGTTCCTTACGAAGCTGCGCGTCATGGCGTGGTCCCACGCCGCCGGGTCCAACCTCCGCGAGGTCGCCATGGGCGCGTGCCACAGTCTAACTCACTTGACGTGGGTGCAGAACCTCCCCTGCCTAGAATCGCTGAACCTCAGCGGGTGCAACGGGCTGACGAGACTGCTGGGTGGCGCGGAGGACAGCGGCAGCGCCACGGAGGAGGTGATCGTGTTCCCGCGTCTGAAGCTGCTGGCCCTGCTGGGGCTGCCGAAGCTGGAGGCCGTGCGAGTCGAGGGAGAGTGCGCGTTCCCGGAGCTGCGGCGCCTGCAGACGAGGGGGTGCCCGCGGCTGAAGAGGATTCCTATGCGCCCGGCGCGCGGGCAGCAGGGTACCGTGCGGATCGAGTGCGACAAGCACTGGTGGAACGCTCTACAGTGGGCGGGCGAGGACGTCAAGGCCTGCTTTGTCCCTGTGCTGTGA

>B73_Zm00001eb015450

ATGGATTGGTTGAGCAGCATGCTGGGGGACCGCCCGCTGAAGAGCGTCTTCACGGCGCTCGGTCTCCCGGATAAGATTGGCGGCGCGGTAATCGACGCTCTCTGCTACCGGGGCGTCCGCCTGTGGAACGTCGAGGAGGAGGCCGACAAGCTGCGGCGCACCAAGGAACGCATCCGCGCCGTGCTCGAGGACGCCGAGCAACGCCGCTTCATCGACCACGACTCTGTCAGGCTCTGGCTCCGGGAGCTTAGGGCCGTCGCCTTCGACGTCGACGCCCTGCTCGACCGCCTGGGAACCATCACGGCCGTGTCCAGGCTAGCGGCCGCCGAGCAATCACGGAAGCGGAAGCGGCTATGGCCCAGCGTCGAGCTCGGCCCGCGGCAGCGGTGGGAGCTGGATGAGAAGATCGCGAAGATCAACGAACGCCTCGACGAGATCAACACGGGCAGGAAATGGTATAGGTTGCAGGCCGGGGACGGGACGAGGGCAGCGTCTCAGCCGACGCAGCGCCCACGGTTCCTTGAATCTGCCGCGCATCGCGACGAGAGGCCCATTGGCCGCAACGAAGAGAAGGAGCAGATTGTCCGTGCTCTGGTTTCGGATAGCGCAGATATGGCGGTGATTTCCATATGGGGAACGACAGGCATCGGGAAGACAGCACTGGCACAATCGGTTTACAAAGATCCTGAGGTACAAAACTTCTTCACCGACAAGATCTGGGTTTGGTTATCAGATAGGTGTGATATCAGAAAGGCCACCAAAATGATCATCGAAGCGGCGACCAATCAAAAATGTGAGCTTCTAAGCTTGGACATATTGCAGCAACGGCTGCACGACCACCTACATAAAAAGCAGTTCTTGCTGGTGATTGATAACCTTTGGGCAGAGAGCTTTCAGTTCTGGGAGTTTCTGAGGCCCTCATTGACTGGTGGAGCGGAAGGAAGCAAGGTTCTGATCACTACTCAGCATGAAAAGGTGTCTAGGATGATTTCCACCAATCTAAACATCCATTTAAAGGGCTTGGAAGATGAAGAATGCTGGCAAATCCTCAAACTCTATGCGTTCTCGGGGTGGGGCAGCAGAGATCAGCATGATCTGGAACCCATTGGGCGGAGCATTGCCTCAAACTGCCAGGGCTCCCCGTTAGCTGCTAAATCTCTTGGGTTACTACTGTCCGACACTCATGGAGACAAAGAACAGTGGGAAAACATACTAGGTGAAATGCAGATTCTCGGAGATGGCGAAAACACAAACAGCATATTACCAAGTTTGCAGATAAGTTACCAGCACTTGTCATATCATCTCAAACAATGTTTTGCCTTCTGTTCAATACTTCCTCCTGGTGTTGAGTTTGAGAAGGATGAGCTCGTCAGACTCTGGATAGCTGATGGTCTTGTTAAGAGTAACGGAAGGGAAAGGGTTGAGATGGAAGCAGGACGATGCTTTGATGAGCTCCTATGGAGATCATTCTTTGAAACATCCCGCAGCTTCCCTGATCAAAAGTTTAGAGTGCCAAGTTTGATGCTTGAGCTAGCACAACTTGTTTCTAAACACGAATCTCTGACTCTCAGACCTGAGGATTCACCGGTAGTCGACCATCCCGAGTGGATACGTTATACAACTATACTGTGCCCGAAAGATGAGCCTCTTGCATTCGACAAGATCTATCGCTATGAAAATTCGAGGCTCTTGAAATTATGCCCCGCAATGAAACTACCTTTGAACCAGGTTCGTTGTGGTCTCCAGGGATGGGGGCAGGTGCAACATCAACGAGCTGAGGAACTTGAAGATCCGCGGAGAGCTTTGCATTCTTAA

>B73_Zm00001eb024240

ATGGCAGAGGGTGTTGTTGGCATCCTTATTCTGAAGCTTGGTTCAGCCTTGTTTTTAGAGGCTTGCAGGCTTGGCACAAAACAGCTCTACCATGAAGCTTCAGCCCTTGGCAGGCTCTTTGGTGAGATCCGTGATATCAAGGAGGAATTGGAGAGCATGCAATCTTTTCTACAAGGAGCTGAGAGATTCAAAGATACTGACAACAACACTGCCAACTTTATCAAGAAGATTCGTGGCCTTGCTTTCGACATTGAAGATGTTATCGATGAGTTTATCTACAAGATGGAGGACAAGCATGGCAGTTTTGCTACAAAGATGAATCGTCGGATTAATCGTATTTGGACATGGCGACGTCTCACGTCCAAATTGCAAAAGATCAAACTGAAACTAGAGAATGTTGATAAGAGAAATGTTCGATATGACATGAGAGGAATTGCTAGAGAAGATGGAAGCAGTGATGCTCATCATAGATCTACCGACCAGATTTCTTACTTCCCCAAAGAGGAAAATCTTGTGGGCATTGATGAAAACAAGGAGTTATTGATGAATTGGCTAAGGGGTGATCTACATCAGCAAAGTGTAATTACAACAGTATGGGGGATGGGGGGAGTTGGCAAGACCACTTTGGTTGCACATGTTTACAACACTGTGAAGGTAGACTTTGACAGTGCTGCATGGATAACTGTTTCAAAAGCTTACCAAGTGGAGGACTTGCTGAAGCAGATCATCAGGGGATTTCAGAAAAGTGATTTGAAGGGTGAACTTCGTGTTGACATAATTGACATGGAAAAGAGAAGCCTAGTTGAGATCATCCGTGATTATTTGCATGGCAAAAGCTATGTTTTAGTGCTAGATGACGTCTGGGGTGTTGACATCTGGTTCAAGATAAGAGATGCTTTTCCTACCAATAGCACTAGCAGGTTTATTATTACATCGAGGATACATGAAGTAGCATTGCTGGCTAATGGAAATTGCATAATTGAGTTGAAGCCACTAGAGGCACACCATTCATGGGAGTTATTTTGTAAAGAGGCCTTCTGGAAAAATGAAAACAAAATGTGCCTGCTGGAACTTAATAATTTGGCACAAAGATTTGTTGACAAGTGTAACGGGCTGCCCATTGCCATTGCATGTGTAGGCCGTCTTTTGTCTTGCAGAAGCCCAACCTACTCTGATTGGGAAAGCTTTTTCAAGGAACTAGAGTTGCAGATGACAAATAATGTGATTCTTAATGTTAATGTTCTTCTAAAGGTTAGTTTAGAGGATCTTCCATATATTTTAAAGAACTGCTTTTTGCACTGTACAATATTTCCCGAGGATCATTTGATCAAAAGGAAAAGGTTAATTAGGCACTGGGTAGCAGAGGGATTCATCAGAGAAACAGAGCACAAAACAATGGAGGAAGTGGCAGAGGGCTATTTGTATGAACTTGTCAATCGTAGCCTATTACAAGTAGTGGAGAGAAATGAAAGTGGACGAGTGCAGAGTTGCCGAATGCATGATATTATTCGACTTCTTGCTCTGACAAAAGCAAATGAGGAAGGCTTCTGTAAAGTTTATGATGGCATGGGGAGTTATTCAGCAGAAAAGACACGTCGTTTATCGATTCACAGTGCAAATATTAAGTTGTCGACTCAACCAACAAAGCTTACAGTCCGCTCAATATATGTTTTTAGTAATGGTTTGACTATTGAATCACTTAGGTCTTTCTTGAAACATTTCTACTTGCTGTCAACTCTAGATCTCCAGGGTGCCCAGATTGTGGAGCTGCCAGATGAGGTTTTCAACTTGTTTAATCTACGGTTTCTCAGCCTTCGAAATACTGAGGTTACGAATATCCCCAGCACAGTTGGAAGATTACAAAAACTTGAAGTCTTGGATGTTTATAATGCTAAACTGTTGGCTTTGCCAGAGAGTGTTTCGAAGCTTAGAAAATTGAGATATCTACATGTAGCTACTGTTCCAAAGATAAATACTAAAGGGGTTGTGACCTGGATTGGAATCCAGGTGCCTAAAAGCATCAAATACCTGACAGGCTTGCAAACCTTGAGGCTTGTTGAGGCGAGCTCAGAGACTTTATTTCACCTTGGTGCTTTGACACAGTTGAGAACTTTTGCCATCACAAATGTGCAGAGGGATCAGTGTGCCGATTTGTGCACTGTTATAATGAGCATGAAGCATCTTGTTAGCTTAGCAATTATGGCTATAAGTGAGGAGGAAATACTTCAACTTGAAGAACTTTGTTTACCCCCAACTCTTTCAAAGCTTGAATTAGGAGGGCAGCTGGACAAGAAAGCAATGCCCCAGATTGTATCATCCTTTTCAGATCTTGGTAACCTCACCTTATTGGCCTTGGCATTCTCCAAACTTGATGAGGACTCATTTTCATGCCTCTTGACGTTGCATGGTCTACGTGGGCTTTGGGTTGATAAGGCTTATGAAGGGAAGAGGCTTCACTTTAATGCTATGTCTTTTCCAAATCTTCGACAGCTTGCAATATCAGATGCACCGCAGCTCAACAGCGTTGTAATCGAACGAAGCGCACTGCAAAGCCTTGTTCAGCTGACACTTGTAGATTGTCCAGAACTGAAGGCCCTGCCTGATGGCATTGAGCATCTTAGAACACTTGAGAAATTATATCTGCGAGGAGCATCCAAAGAGCTCACAAAGCTATTTCAGTGCAATGAAGAAACACATGAGTCCAATGGGAATCTTGAGAAGATCGGTCATATCCGAAGGGTTACTGTTTATCCA

>B73_Zm00001eb030390

ATGGACCGGATGCTGCTCGACCAGCTGGCCGGCGAGGCCCTGCGGGAGGTGCTGCACGCGGTGCAGGGCACCCTGTTCTGCCGCTCCACCGCCGAGCGCCTGCGCCGGAGCGTCGAGCCGCTGCTGCCGCTCGTCCAGGGCCTCGGCCCGCACAGCACCCAGCGCTCCGCGGGGGAGCTCGGCGAGCTCGCGGCGCGGGTCAGGGAGGCGCTCGACCTGGCGCGCCGCGCCGCCGCGTCCCCGCGCTGGAACGTCTACCGCGCCGCGCAGCTGTCGCGCCGGATGGAGGCGGCCGACCGCGGCATCGCGCGCTGGCTGGAGCGCCACGCCCCCGCGCACGTCATCGGCGGCGTGCGCAGGCTCCGCGACGAGGCCGACGCGCGCATCGGTCGCCTCGAGCGCCGCGTCGAGGAGATCGCCGCCGCCACCGCGCAGCCGCCGCCCCCCGCCCTCTCCGTCCCCGTCGCGCCGCCGCCGCACAAGGGCGTGCCCATGCCGATGGAGGCGCCGCTCGCTAAGCCCGCCTTCGTCGCTATGACGAAGGAGGTGCCGCAGCACAAGGGCATGGCTATGTCGGAGCCGGTGCCGGCGAAGGCGGCGCCCGCCAAAGCCGGGGTGATGGCCATGGACATCGCCGACGGACACGAAGACGCGGAGGGGATGGTTGGCGGCGGCGTCAAGGTGGCCAAGGAAAAGGTGAAGGAGATGGTTATGAGCGGCGGCGGCAGCTGGGAGGTGGTCGGGATCTCCGGCATGGGCGGCAGCGGCAAGACCACGCTCGCCATGGAGGTCTTCAGGGATCACAAGGTCCGAGCCTACTTCAACGACAGGATCTTCTTCGAGACGATCTCGCAGTCCGCGAATCTGGAGGCCATCAAGATGAAGCTGTGGGAGCAGATCAGCGGCAACATGGTGCTGGGTACATACAACCAGATCCCAGAATGGCAGCTCAAGCTAGGACCAAGGGACCGAGGACCCGTCCTTGTGATCCTCGACGATGTTTGGTCTCTCCCGCAGCTTGAGGAGCTCATCTTCAAGTTCCCTGGGTGCAAGACCCTAGTCGTATCAAGGTTCAAGTTCCCCACGCTGGTGAAACAGACGTATGAGATGCAGCTGCTAGACGAGGCGGCGGCTCTGTCCGTCTTCTGCCGCGCTGCGTTCGACCAGGAGTGTGTTCCGCAGACCGCCGACAAGAGATTGGTCAGGCAGGTCTCTGCAGAGTGCAGAGGTCTCCCTCTGGCTCTGAAGGTCATCGGCGCGTCGCTGCGCGACCAGCCTCCGAAGATTTGGCTCAGCGCCAAAAACCGGTTGTCTCGAGGAGAGGCCATTTCTGACTGCCATGAGACCAAGCTTCTGGAGAGGATGGCGGCCAGTGTCGAGTGCTTGTCCGAGAAGGTTAGGGACTGTTTCCTTGACCTGGGCTGCTTCCCGGAGGACAAGAAGATCCCCCTCGACGTCTTGATCAACATCTGGATGGAGATCCATGACCTTGATGAGCCAGATGCTTTTGCCATCTTGGTTGAGCTTTCGAACAAGAACCTTCTTACCCTCGTTAACGATGCACAGAACAAGGCTGGAGATCTGTACAGTAGCTACCATGACTACTCGGTGACACAGCACGACGTGTTGAGAGATCTTGCTCTTCACATGAGCGGGCGTGACCCGCTCAACAAGCGCAGGCGGTTGGTGATGCCGAGAAGGGAAGAAACACTTCCGAGGGATTGGCAGAGGAACAAGGATGCTCCGTTTGAAGCTCAGATAGTCTCCATTCATACAGGCGAAATGAAAGAATCCGACTGGTTCCAGATGAGCTTCCCCAAGGCAGAAGTGCTGATCCTCAACTTCGCGTCGAGCCTGTACTACCTGCCGCCGTTCATCGCGACGATGCAGAACCTGAAGGCCCTGGTGCTGATCAACTACGGCAGCAGCAGCAGCAGCGCAGCCCTGGACAACCTCTCCGCCTTCACCACGCTGAGCGGGCTGAGGAGCCTGTGGCTGGAGAAGATCAGGCTGCCGCCGCTGCCCAAGACGACGATCCCGCTGAGGAACCTGCACAAGATCTCGCTCGTGCTCTGCGAGCTGAACAGCAGTCTAAGAGGGTCGACGATGGACCTGTCGACGACGTTCCCGCGCCTGTCCAACCTGACGATCGACCACTGCATAGACCTCAAGGAGCTGCCGCCGAGCGTCTGCGAGATCGGGTCCCTGGAGACCATCTCCATCTCCAACTGCCACGACCTCACCGAGCTGCCATACGAGCTGGGGCGGCTGCGCTGCCTCAGCATCCTCCGCGTGTACGCCTGCCCGGCGCTGTGGCGGCTGCCGGCGTCGGTGTGCAGCCTGAAGCGGCTCAAGTACCTGGACATCTCGCAGTGCATCAACCTGACGGACCTCCCCGAGGAGCTCGGCCACCTGACGAGCCTGGAGAAGATCGACATGCGCGAGTGCTCGCGCCTCAGGAGCCTCCCCAGGTCGTCGTCCTCGCTCAAGTCCCTCGGACACGTCGTGTGCGACGAGGAGACGGCGCTGCTGTGGCGTGAGGCCGAGCAGGTCATCCCTGACCTCCGCGTGCAGGTGGCCGAGGAGTGCTACAACCTGGACTGGCTCGCGGACTGA

>B73_Zm00001eb033040

ATGGCGGACGCGGGGGTGACGGGGGTGCTGGCCAAGCTGGGTGAGCTGGCGGCGGAGGAGGCGACGGCGCTGCTGCGCGTGGACGCCGAGATCCGGGCGTTGCGGCGGAAGCTGGCCTACCTGCAGGCGCTCGTACGCGGGGCCGACCGCCAGCGCCGCGGCCGCGCAAGCGAGCTGCTCCTGCTCTGGCTGCGCGAGACCAGAGAGGTTGCTTTCGAGGTTGAGGACGCCGTCGATGAGTTCCACCTCCGCGTCGAGGCCTGCCGCCCCGGGGCCCGGTGGCGCCGGCGCCGCAGATGGTGGTGGGGCTGGCACCGCGACGCCGTCAGCCTCGTCCAGGACCTCGCCACGCAGTTTTTTGTACGTCATGGGCTGTCAAATCAAATATCTAAGATCAATGAAAGGATTGATGAGCTTAACCAGAACAAGGAAACATATCAAATTGAAAGTTCTCCTTCTGAAATTTGGAGTTCTTCATCGGTTGAAATGGATCCTGAGTGGTACGAAGATAAATATGTTATAGGCTCTAGAGAACGTGAATTTGCTATCCTTAAGGACCTAATCATCAACAAAGAGGGAGATATGCCTCACCGGGCTGTCATCTCTATTTTTGGGGAGCGTGGCATTGGAAAGACTACACTTGCAAAACAACTGTACAATGACCCAGATATCATAAAACACTTTGAGGTCCATGCATGGGTATGTCTTCCACCACATGTCAGGTTCAGGGACTATGTTGAGATTATGCACATGCAGGTCAACCCACAGATTCCAGAAGCTTCTGAGAAAAATGATAATACAACATTTGCACTCGGTAACGAAGAAACCACTGATATGGAATTCAACCTTCGGCAGAACCTTGAGAACAGGAGGTACCTAGTTGTTCTTGATGGTTTAGTCAGCATTAGTGACTGGAACTCATTATTTGCTGTGCTGCCACATACCAATGCCAATGGCAGCCGGATCTTACTTACCACACATCTCAATGTGAAGGAAATCAATCACATCGACCCACAGATAGCTCCTGTCAAGCTTCCTTATCTTGACGAAAAACATGGAGAGGAGTTATTTTGTCAAAGAGTTTTTGGGACAATAGAACCTCCACAAATTTATAGGAGCAAGGTTTACTATAAAAAAGTTCACAATATATCAACAGGTCTACCCCTGGCAATTACTGTGCTTGCAGGAATATTACGATCAAAGTTTATCCCCATGGAGTGGGATGTCATATTCGAACAACTCGAGTCCAATGGCCAGCCAAAACCGGTTAGAAGCATATGGTCTTTGGCTTTTGATGACTTGCCACACTACCTCAAGTCATGTTTCCTATACTTGGCGTCCGTTTCAGAAAATGTTATTCTTTACCCAGATCGTTTGGTGCGTCTGTGGATTGCTGAAGGTTTTGTTATGCCCAAGAAAGCAGAAACACTGGAGGACGTTGGGTTTGACTATCTAAAAGAACTGGTCGCGAGAGGGTTAGTCCAGGTCGTGCAGAAGGATGCTGGTGGATCCATCAAGCTGGTAGCCATCCACAATCTGCTCCATGCTTTTGTGGAGTCTGAAGCACAGGACTCTAGTTTCCTTGAAATCCACCATCATGCTAATGTTGTAAACCCAAATGCAGTGCGGCGCCTTGCCATACAAAACTATGTGGATGCATATGTCCACATTCCTAATGTGTTCCCCAAGCTGCGTTCGCTTCTCTGTGATTTTGCAGAAGACCAACAACGCGGTAGCTCAAGCTCTGGAGAGCTGCAACCTCAGTCGTTATGGGGCAATCTTGCAGAGTTGTGTTCAAGAGCCTGTGGCACTTCGGAGAATGTTGGCTCAAGCACATTGCATGGGCTCCACTTCTTACAGGGCTCCAGGTACCTACGAGTTGTTGACCTGTATGGTCTTAAGACGCGAAAGCTGCCAGATGAGATCGGCAGCATAATCCACTTGAGGTACCTTGGCATCAGGAACAGCAACCTGGTGGAACTCCCCTCATCCATCTACAAGCTTGACAGTCTCCAGACGTTGGATGTAAGGAAAACAAACGTGGCGAAAGCCGTCGATGAGTTCTGGGACATCGAAGCACTACGGCATGTGCTCGCCGAGAAAATGATTTTGCCCGATTGCTCAGTAGTTCCCTTGAATAATTTGATGACACTCAACGGTGTGGTGCCTTGTGGTCCGTGGGATGAAAAGAGCTGCCCCTTGAACAGTATGATTTATCTCCGATCCTTGTCCCTGTCTGACATTTCAGCAGCCCATACCGCCGCGCTTTCAGCAGCTCTGAGGAAAATGGAATTCCTTTTGTACCTGAACCTGTCAGGTGAATTTCTCCCGTCCAGCATGTTCACCAGCTCAAGCATGCGCCGTCTCCAGACCCTCATCCTGCATGGTAAGTTACAAGGGATTAATGATCTGCCGAGCGACGACCGCTATGTCCTACCAAACCTTACCATGCTCTATTTGCATGGATCTGAGGTGTCTCAGCAGTTTGTGCACAAGCTTGGTACGCTTCCGTGCCTTGTTGAGATGGAGCTGTCAGCTGTTTCGTACAGTGAGGGTGAGATGAAACTGTTCTTGGACGGATTCCCAAGCCTAGCAAGGCTGAAGCTCAAGAACGTGTCTATGTTACAGGAGGTAGAGATACGTGGAGGGGCCATGCCAATGCTTTCCATCCTAGCCATGTATGATTGCGACAGCTTGAAGACCTTCAAGAACCTGAATAGCTTGGAACACCTTCAAGAAGTGGCGATCTACAATACGCCGCCGGAGATTGTTGATAACATAAAGCTTGAGGACGAGAAGCTTTTCAGCAAGATCAAACTCCTGACTAACCCCATGATGACAGCAGACCGAGACTTTCCAGGTCATGTTGTCAGAATGAGAATTAAAATGTTGGATGAACCGGCTCATGATCTACGTGTCGCAGTGGCCTCTGAACCACATTGCAGTGACATGGAGGGTGATTATCATGGTGAAAGGAAAGCAACAGACCAAGTACACTACGGCCCTGGTCTGTTATAA

>B73_Zm00001eb037410

ATGGCTGACACGCTGCTTGTCCCCGTGGTGGCCAGGGTGGCCGGCAAGGCCGCCGACGAGCTCGTCCAGAGCGTCGCCCGCACGTGGGGCGTCGACGCCGACCGTGCCATGCTGGAGCGGACGCTGCTGGCGGTGCAGCGCGTGCTGCCTGACGCCGAGGCGAAGGGCGAGTCCAGCCCCGTCGTCCGGATGTGGATGAGGGAGCTCAAGGCCGTCGCCTACCGGGCAGACGACGTCCTCGACGACCTACAGCACGAGGCTCTACGCCGTGAAGCTAGCGAGCGTGAGCCGGAGCCGCCCATGGCGTGCAAGCCGACGAGGCGGTACCTGACCCTCCGCAACCCTCTCCTGCTCCGTCGTCTCACCGTGAGCAGGAGTCTGCGCAAGGTCCTCAAGGAACTGAACGGTCTTGTCCTGGAGACGCGCGCTCTAGGCCTGGCGGAGCGCCCGGCGGCGCGACACCGACACGCACACGCTCCGTGTCAGCAAGTGCGGGTAGCACTGAACGGCGGGTCGGCTGAGATCTTCGGCAGAGACGGTGACAGGGACGAGGTAGTGAAGCTGTTGCTTGACCAGCGACACCACCAAGACCAGAAGAACGTGCAGGTGCTGCCCGTCGTAGGGGCCGGTGGTGTGGGCAAGACGACGCTGGCAAGGATGGTGTACACGGACCGCAGGGTTCAGAAGCACTTCGAGCTGAGGATGTGGCACTGCGTGTCCGGAAACTTCGGAGCCGCCTCTGTCGTGCGATCCGTCGTCGAGCTGGCTACGGGCGAGAGGTGTGACCTGCCTGACGCCGGCAGGTTCTGGAGAGCGCGGCTCCAGCAAGTCGTCGGCAGGAAACGGTTCCTGCTCGTCCTTGACGACGTGCGGGACGACGAAGAGCGGGAGAAGTGGGAGGGCGAGCTCAAGCCGCTGCTGTGCACGTGTATCGGTGGGTCAGGGAGCGTGATTCTTGTCACTACTCGAAGCCAGCAGGTGTCTGCTGTGATGGGCAGCCTTCCGAGCAAGGAGCTAGCACGCTTGACTGAAGAAGATTCATGGGAGTTCTTCTCAAAGAAAGCGTTCAGCAGAGGAGTACAAGAGCGACCAGAACTGGTCGCTATCGGCAGGCGCATCGTCCACGTGTGCAAGGGGCTGCCTCTTGCTCTGAGTACAATGGGTGGCCTGATGAGTTCGAAGCAAGAAGCTCAGGACTGGGAGGCCATTGCAGAAAGCTGTAGTAGTGATACTGATACCAGTACAGGCTCAGGCACGGATGATGAAGTGTTGTCCATGCTTAAACTGAGCTACGGACACTTGCCAGATGAGATGAAGCAGTGTTTTGCGTTCTGCGCGGTGTTTCCCAAGGACCATGAGATGGAGAAGGACAGGTTGATCCAGCTATGGATGGCGAACGGTTATGTTGGTGGAGAGGGAACGGTGGATTTGGCGCAGAAAAGCGAATCCGTTTTCAGCGAGCTGGTCTGGAGGTCCTTTCTCCAAGATGTGGAAGGGAAGGTGTTCTGTAACTCGCTTCATGAGACGGTTATATGTAGAATGCATGGCTTAATGCACGACCTGGCAAAAGATGTCTCGGACGAATGCGCGTCTTCAGAAGAGTTGGTTCGAGGAAAAGCAGCGATGGAAGATGTATATCACCTGCGAGTGTCATGTCATGAGCTGAATGGAATCAATGGGTTACTGAAAGGCACGCCATCTCTCCACACATTGTTACTAACTCAGTCAGAGCATGAGCATGATCATCTTAAGGAGCTGAAACTGAAGTCAGTGAGATCGTTATGCTGCGAAGGTCTCTCTGCCATCCATGGCCACCAGCTTATAAACACGGCGCACTTGCGGTATCTTGATCTTTCCAGGTCAAAGATCGTTAGCCTGCCAGATTCACTATGCGCGCTGCACAACCTGCAGTCGCTGTGGCTCAATGGATGCTCCAGGCTACGGTACCTACCAGATTGTATGTCAGCCATGAGGAAGATCAGCTACATCCATCTCTTGGAATGCGACAGCTTGGAACGGATGCCGCCAAAACTCGGTCGACTGCAGAACCTTCACACGCTGACAACGTTTATCGTGGACACTGAAGATGGATTAGGGATCGATGAGCTCAGAGACCTGCGACACCTCGGCAACAGGTTGGAGCTGTTCAATCTAAGCAAGGTGAAGGACGACGGTTCAGAGGCGGCCAATCTCCATGAGAAGCGGAACCTTAGCGAACTGGTGCTGTACTGGGGCCGCGACCGGGATTATGATCCGTTGGATAACGAGGCCTGTGATGAAGACGAAGGGGTACTGGAATCTCTTGTCCCTCACGGTGAGCTCAAGGTTCTGAAGCTGCATGGGTACGGTGGCTTGGCTGTGTCAAAATGGATGAGAGATTCTAGGATGTTCCAGTGCCTTAGAGAGCTCGTTGTCACTGAGTGCCCGAGATGCAAGGATCTGCCGGTGGTATGGCTGTCACCTTCTCTCGAGGTCTTGGAATTATCCGGGATGATCGGCCTGACAACACTGTGCACGAACGTCGATGTGGCAGAGGCGGCAGGACGCAGCGCGTCCCGGCAGATTTTCCCGAAGCTGAGGAGGATGCGGTTGCAGTACTTGCCTGAGTTGGAGAGATGGACAGATCAGGACAGTGCAGGAGAGCCTGCTGGTGCCTCAGTGATGTTTCCCATGCTTGAAGAGCTAAGAGTCTACGAGTGCTACAAGCTTGCGAGTTTCCCCGCGAGTCCAGCTCTCACGCTTCTATCTTGCCGAGGTGACTCTGGGCGCTGCCTTGTTCCTGTGAGCATGCCCATGGGCTCTTGGCCGTCTCTTGTCCACCTGGACATTGGGTTGCTGGCTGAGGTGGTGATGCCCGTAGAAGACACTCAAAGCCAAAACCAAAGGCATCTGAACACCATGCGGAGTGTGAAAGTCCTTGGCGAAGACGGCTTCGTGTCAGTGTTCAACCTGTCGAAATCTCAACTCGGGTTCCGGGGCTGCTTGGCCTTGGTGGAGAAACTGGAGATCGGATCATGCCCGAGCGTCGTTCACTGGCCAGTGGAGGAGCTCCGGTGCTTGCCTCGCCTTCGGTCTCTGGATGTCTGGTACTGTAAGAACCTGGAGGGGAAGGGCGCATCGTCTGAAGAAACGCTTCCGCTGCCCCAGCTGGAATGGCTATCGATACAGCACTGCGAGAGCTTGCTGGAGATCCCCAGGCTGCCCACGTCCCTCGAGCAAATGGCGGTCCGCTGCTGCAGCAGTCTGGTGGCTCTGCCCTCAAACCTTGGAAGTCTGGCCAAGCTCGGGCATCTCTGCGTTGATGACTGTGGTGAGATGAAAGCGCTGCCTGATGGGATGGATGGCCTCGCTTCCCTTGAGAGCTTGAGCGTTGAGGAGTGCCCGGGGGTTGAGATGTTTCCACAGGGTCTCCTCCAGCGGCTCCCAGCCCTCAAGTTCCTGGAGATCAAAGCCTGCCCTGGCCTGCAGAGACGTTGCAGACAAGGTGGGGAGTACTTCGGCTTGGTTTCTTCTATTTCGAATATAGACATTCCAGCAGTAGAGTCCAACGTAAAGAAGTTTGTGAAGAAGCTCATCCCTTTCTGCTGA

>B73_Zm00001eb061810

ATGTCCGTGTCACGCCGCGCCAGCCGCCAGAACCCCAAGCCAAGAGCTTCCTACCGTCGCCCTCCTTCTTCAAAAAGGACTGACTGGCCTGCACGTCTTCTCCAGTTCTATTTTGCTGCCAGTTCAGAGTTTGGACACTGCTTGGACTTGGAGTGGCATTTCAAGCCAGCTAAGGGTCCAGATCCACCATCCATGGCTGTGCTTCCAACGCGTCAGGATCCCTTATTAAATATCCCGGCAGCGCCTTCTCCACTCCCTGCTGGCCGTCTTGAGCATGAGCATGATGGACCTACTCCTTTTCTTTTTCCAGCCAGGAGAATCAACAACTTGGCCCTTGCAAGCACGGTGCTTCCAAAACGTGTGCCGCTCCATTTATTACAGAAAATAACAAATGATTTCTCTAAGGACCGTGAACTTGGTTCCGGTGCATACGGAAAAGTTTACAAGGGAGTGCATAAAAATGGCGAAACGATGGCTGTCAAGTTGCTTCACTCCATGGCTGGACTTGATGATAAACTTTTTGAGGAGGAGTTTCACAACATTGCAAGACTCCAACACAAGAATATTGTGCGTCTTGTTGGCTTTTGCCATGAAACTCAACGCCAATTCATACCGCACGATGGAAAAATGATTTTTGCTGATAGCAAATATATGGCCCTTTGCTTCGAATATATGCATAATGGAAGCCTTGACAAGTTCATTATTGATGAAAATAGTGTCCATGACTGGTCCACACGCTATGCAATAATCAAGGGGATCTGTGAGGGCTTGAAATACCTTCACGAGGAATTGAATCCTCCTATTTATCATTTGGATTTAAAACCAGCTAATATTTTGTTGGATGACAATATGATGCCAAAGCTTGCAGATTTTGGCTTGTCAAAGATTTTTGAACAGGAACGAACACGAATCACACAAAGTTGTGTAGGAACACATGGATATCTACCACCAGAATATATAGACAGGAAAGTAATCTCAAATAAGCTTGACATATTCAGTCTGGGTGTCATCATCATAAAGATTATAAGTGGACCTACAGGTTACACCCAGTGTGCTGAAATGCCTTCCAAACAATTTGTTGAGCTTGTACATGAAAAATGGAGGAATAAGTTACAAACAACAATGGATGCAGCTGCATTGGAGACATATTGTGAACAAGTAGTGAGATGCATTGAAATGGCTTTAAGTTGTGTGGAGACCGATCGGCACAAAAGGCCGAGTATAGGGGCTATTATTGATGAGCTAAATAAGATGGAGACAAATACCGACCAGGTATTTTCAGTGGATATATGCTCGACTCCAGCACAAACACAACCACCACCCCTTCCTGATTCTAGCCACAGCACAGAAGAAACAAGCAACACAAAGGGAGTTAGCAGACTGGGACCGAGAGAGTACGTACAGAGCACAATGCGGGACCTGGTAATTGGGATAACCAAGTCGGTGGTGGAGGGGGTGATCAGCAAGGCCCAGTCGGTGATGGAGGAAGAAAATATGCTACTTATTAGCATGCAGCATGACCTCCACTTCATCACCGATGAGTTTCAGATGATCTATTCATTCCTTAGCATCACTGATGAGGAGCGTATCAACAACGCGGTAGTGAGGATCTGGGTGATGCAGACGCGTGGTCTGGCCTATGACCTGGAAGACTACATGGATGCCTTCTCCTGCCTGGACAAGACCAGAAAATGGTGGTCCCGCTTGTTCCGAGCATGCCTGGCACCACCGGGGTCTCCATACATGTCAGTGACATCTCTGGCCGTGGAACTCAAGCATCTAAAGGCAAGGGTCGAGGACGTTAGCCACAGGAACACTCGGTATAACCTCATCAGCGACGCCGGCTCAAAGCCCGGCCTCGTGAGGCAACAGCCGGTGCCTCCTGGTGCTGTGGCATACGAAAACGACACAAGAAGGAAACACATCTTAGGGGATCTCACCCAGCTTATCACCAACAAGGACGATGCTGATCTTCAATTGATTTCAATGTGGGGTGCAGGTGGAGGCACCGGGACGACATCCATCATAAGGAAGTGCTACAGTGATCCAGAGATCATCCAAAATTTCATATGCCGAGCCTGGGTCAACCTCAGCCATCCATTCAACCCCCATGAGTTTGTGCGGTCTTTGATGGCTCACTTTTATGCAAACAGTTGCCAAGAAAGACAAGGAGGGATCATCGGTGTTGATGTCCTAACTAGCATGGATGCGACTGCCACCCAGGGGGATCTCCTCAGCGAGTTCGTTCAGCTAGTTGACAAGAACAGGTACCTTGTTGTCTTGGAAGACCTGCCCACCATGGCAGAGTGGGACTCTATCAGGACGTTCCTTCCCGACAGAAAGAATGGCAGCTGGATCATCGTGTCCTCGCAGAAATATGAAATTGCAAGTTTATGTGTCGGACATCCCTACCAAATATTGGACCTGAAGCAGTCCTCGCCCACTGAGCACTCCGTATGTGCCTTCTTGAGAGAGGAGGTCACATTAAAAGAAAAAAAAGGAGGCAATAGAAAAGAAAAAATTTATGTCGGAGATGAAAGCGAGGTGAACAACGGTAGTACATCAACCAACAGCAAGGTGATATCAACTGAGTATGACTTAAGCCATAACCATAGGATGGTCGCCAACTCTAAATGGAAAGAAGCCAGAGACTGGATGCAGAATTCTTCCCTTGTCGGACGCAGATTGCAGATGTATCAAATTCATCGCTGTATAGCTGAAGCACAAAGGAACCATTCCCATGTTATGTCTGTTTGGGGAATAGTTGGCGTTGGGAAATCAGCTATTGTCAGAAATTTTTACTGCGACAGGATACTTGAGAATCAGCAATTTCAAGAATACGGTTGGGTGGATTTATCACACTCCCAACCCTTCAATTTGAAGGACTTCTCACAGAACTTGCTTACGAATTTTCAATCAGATTCTCTTCAAGCCAATGAAACTACACCCTCTCGTGGTATACTGGGGACAGTCCGAAAATGTTGTGAGTTTCTAAGTACACGTCATTGCCTCGTGGTCATAGATGGCCTTCAGTCCAAAGAGGAATGGGATTTGATACAATCATCATTGGTATCTAGATACTCTAAATCTAATACTATTATCATTGTGATAACAACTGAAGCAAGCATTGCAGCATACTGCGCAGATAAGGATGACCTTGTGTTTAATGTCAAAACTTTAGAAACTGATGCAGCCTTTGATCTCTTCGAAAATGAGATATCTAAGAAGGCTCCGTTGTGGACCACTTCATTATGTGATCGAGGAAATTCAGTGACGCAAGAACTTGTTTCAAAGTGCGGGGGGCTCCCAATGGTAATAGTGGCTATAGCTGGCATATTGGCTACCAAGGTAGTCTCCTGGATGGACACTGCAACATCCATCAATTTGAGGTTTATGTTTGAGCTAGAGATCAATCCAGACCTGCAGGATCTGTTTAGCTGGGTGCATTCCTGCTTCCGTACTTGTCCAGATTTCATCAAGCCATGTATCTTCTACCTATCATTTTTTCCAAGAGACCACAACATTCGACGAAGGCGTCTAGTAAGGCGTTGGATCGCTGAGGGTTACTGCAGGGACAGTGATGATAAATCTGCAGAGGAGAATGGGGAGTTCTTCTTCTCCAAACTCCTCGGTATGAGCATAATCCAACCGGAGTCCAGTGACGCGAGGATGGTCTCGTGTCAAATCAATGGATTTTTCCGTGAGTGCATCCTCTCACGGTCAGGGGAAATGGAAGACCTTGTCATTGAACTGGCCGACAGATGCAACCTAACCACCCAACGCAGTGGGCGTCATCTTGTCATATCGGAAAGCTGGGACAGAGATAAGATTTTATTCGAGAGCATCGACTTCTCACGGCTACGGTCAATGACAGTGTTTGGAATGTGGAAATCATTCTTCATCTCCAAAAGTATGAAGCTTGTCCGGGTGTTGGATCTGGAAGATGCATTAGGTTTAACAGATGAAAATGTTGATCAGATGGTGAAGCTGTTGCCTTGCCTCAAGTTCCTTTCTCTACGAGGATGCCAAAAGATATCACATCTGCCTAGCTCATTAGGTGATCTGAGGCTGCTTCAGACTCTGGATGTGGTGAACACCTCCATAGTCACTTTGCCAGCAAGTATCACCAAACTAAAAAAGCTGCAGTACATCCGTGGCGGCACAACTTCTATCACATCAGAGGATCCATTAGTGCCACATACTTCTGTATCCTGGTTGTCCAAGTTCCGTCGCAGTCCTCTTGGTGGTCTTGAGGTTCCCAGTGGGATCGGGGAACTGACGGGATTGCACACGCTTGGTGTAGTTGATATTGGTGCTTCAGCGGCGGAGGCCATACTGAGAGAGCTAAAGAAGCTCACCCAATTGCGCAAGCTGGGAGTGTTCGGCGTCAACAGCAATAACTGTGGAGCGTTTGTGTCTGCAATATCGGGTCATTTCCATCTGGAATCCTTGTCAGTGTGGCTGGACGAAGAAAATGGACGTTGTTCGGATTGCATATTCGTGCCTTTAAAGAACCTACGGAGCCTCAAACTTTACGGGGTTTTAAACAGATTACCATTGATCAACCAGCTCAGCAAGCTCACAAAGTTGGATTTGGAGATAACCACATTAATGCAAGAGGACATAAGTTTTCTTGGCAAGCTACCAACACTATGCATTCTGCGCCTTTGTGTTGAAGATGTCAATGCCCCGTTCCGTTTTAGTGTTTTTACCAATGGAGTTGAGGAGCGCTCTTATCAGAAGCTCAAGGTGCTTGAGATTGCTTGCAGGTCCAGCTTAAGTGTGACTTTTGGATCAGAGGCAATGAAAAACCTTGATCTGCTGAAACTTGACTGCTGCAGTGGGCAGTTACGTCGGCTTTTTGGCCTGCTTCATCTATCTGAACTCAAGGAAGTCTGGCTGAAGGGCTCCTACGAGGAAACGCTGAAGCATGACCTAGAGGTCCAGCTTGCAGCCCACCCAAACAATCCTGTGCTGAAACTGGGGAAAAATACGCTGTGTACGCTATCTTGGATGGCG

>B73_Zm00001eb077540

ATGACTTTTCAAGCCGCAAGCCCACCGAACAACGTGCGCGCGGACGTTTTCTTGGCAAGGCCCGAATCCTTCCAATCCAATCCCCATCCGGGCAACCGTCTGCCGTGTTGCCCCATTCCTGCCATGCACCGCGTGGCGCCTAGCCTCCGCATATGCTTCCTTTATAAGCTTCAAACAACAACTCCCTTTTGCAGATTCCTCGTTCGCCTGCTGCACGCTCACCACTGTACGTCCCCGATGGCTGAAGCCATCAGCGCGACCAGCTCGTGCCTGGAACCCCTGTGCGGTTGTCTGGAAAGCACAGGCGTGTTCGAGGCAGTGGGCCGGGAGGTGGCCGCGTTCCTTCGCATCAAGTCGAATTGTGGCGACCTCGAGAAGGCCCGGGACAGCCTGCGCGCCGTCGAGACGACAGTCAGGGCGCGGGTCACGGCGGAGGAGGACAAGCTGAACGTCTGTGATCCTCAGGTGCAGGCGTGGCTCAAGCGCGTCGACGAGCTTCGCCTGGACACCATCGACGAGGATTACAGCAGTCTGTCGGGGTTCTCTTGCCTCTGCCAGTGCACCGTGCACGCTCGTCGCCGCGCCTCGATCGGCAAGCGTGTTGTGGACGCGCTGGAGGAGGTGAACAAACTGACCGAGGAAGGAAGGCGGTTCAGGACATTTGGGTTCAAGCCACCGCCGAGGGCCGTCAGTCAGTTACCCCAAACTGAGACCGTTGGGTTGGAGCCCATGTTGGCTCGGGTCCATGATTTGCTTGAGAAGGGCGAGTCGAGCATAATTGGTGTGTGGGGTCAAGGAGGCATCGGCAAGACGACTCTCCTGCACGCCTTCAACAATGATCTCGAAATGAAAGACCACCACTACCAGGTTGTTATTTTTATTGAAGTATCCAATTCAGAGACACTGAACACAGTGGAGATGCAGCAGACTATCTCCGATAGGCTTAATTTGCCATGGAATGAATCAGAGACAGTTGAGAAACGGGCCAGATTCCTATTGAAGGCACTGGCCAGGAAAAGATTTCTATTGTTACTTGATGACGTAAGGAAGAGATTCCGACTGGAGGATGTCGGTATCCCAACTCCGGACACAAAGAGCAAAAGCAAGCTGATCCTGACATCACGTTTCCAAGAAGTATGCTTCCAGATGGGTGCACAGAGGAGCCGCATTGAAATGAAGGTTTTGGATGATAATGCTGCCTGGAACCTGTTCTTGAGCAAGCTGAGCAACGAGGCTTTTGCAGCAGTTGAGTCACCGAATTTCAACAAGGTTGTTCGGGACCAGGCCAGGAAAATATTCTCCAGTTGTGGAGGTCTACCACTTGCACTCAATGTCATTGGGACTGCTGTGGCAGGGTTGGAAGGACCAAGAGAATGGATTTCAGCTGCTAATGACATCAATATGTTCAGCAATGAAGATGTGGATGAAATGTTTTATCGGCTGAAATACAGCTATGACAGGCTGAAACCCACTCAACAACAGTGCTTTTTGTACTGCACTCTTTTCCCAGAATATGGATCTATTAGTAAGGAACCATTAGTTGATTATTGGCTGGCTGAAGGTTTGCTTCTCAATGATCGTCAAAAGGGTGATCAGATAATTCAGAGCCTTATTTCAGCATGCTTGTTGCAGACCGGTAGCTCATTGTCATCAAAAGTAAAAATGCACCATGTAATCAGGCATATGGGGATTTGGTTGGTTAACAAGACAGATCAAAAGTTTCTCGTTCAAGCAGGGATGGCTTTGGATAGTGCTCCACCAGCAGAAGAGTGGAAGGAATCGACAAGGATCTCCATCATGTCTAATGATATCAAAGAGCTTCCTTTCTCACCGGAATGTGAAAACCTCACTACGTTGTTGATCCAAAATAACCCAAATTTGAACAAGCTGAGTTCAGGGTTTTTCAAGTTTATGCCCTCCTTGAAAGTGCTGGATCTTTCTCACACTGCAATAACAACACTCCCAGAATGTGAGACATTGGTTGCATTACAGCATCTCAATTTGTCACACACACGTATTAGGTTATTACCTGAGCGGCTGTGGTTATTGAAAGAGTTGAGGCATCTGGATCTCAGCGTGACTGCTGAACTCGAAGATACCTTGAACAACTGCTCAAGGTTACTCAATTTAAGAGTTCTTAATCTCTTTCGCAGTCACTATGGTATTAGTGACGTCAACGACCTGAATCTGGATTCCCTGAAGGCACTGATGTTCCTTGGAATCACTATTTATACAGAGAAGGTGTTAAAGAAACTGAACAAGACTAGTCCTTTGGCAAAGTCAACATATCGTCTGCATCTTAAGTACTGTAGAGAAATGCAGTCGATCAAAATCTCCGATCTCGACCACTTGGTGCAACTCGAGGAGCTGTATGTCGAATCATGCTATAATCTAAACACTCTTGTTGCTGATACCGAGCTGACTGCATCAGATTCAGGCCTGCAGCTCCTCACCCTCTCAGTTCTTCCTGTGCTGGAGAACGTCATTGTTGCACCAACGCCCCACCATTTTCAGCACATCCGCAAATTGACCATTTCGAGTTGCCCCAAGTTGAAGAACATCACATGGGTCCTAAAACTTGAAATGCTCGAGAGGCTCGTCATAACCCATTGTGATGGGTTGCTGAAGATTGTTGAAGAAGACAGCGGTGATGAGGCAGAGACAACAATGCTGGGTCAGGGTCATCCTTCTGAAGAACAGGAAGATAAACGGATTGATGGTGGTCAAAGTGTGTGCAAGAGCGATGACAATGCGCATGCTGAGCTCCTGAACCTGAGATCAATCGTGCTGACTGATGTCAAGAGCCTGAGAAGTATCTGCAAGCCAAGAAATTTTCCCAGCCTCGAGACCATCCGGGTGGAGGATTGCCCGAATCTGAGAAGCATCCCACTGAGCAGCACGTACAACTGTGGGAAACTGAAGCAGGTGTGCGGTTCAGTTGAATGGTGGGAGAAACTGGAGTGGGAGGACAAGGAGGGCAAGGAGAGCAAGTTCTTCATTCCAATCTGA

>B73_Zm00001eb087590

ATGGCTCCTGTAGCAATAGGAGAATGGATGGCATCAGCCTGCATCGCCAAGATGGTTAGCAAAGTGTGCTCTTACCTAGAAGACCAGTATGAATACCAAAGGGATGATGCAAAGGATAAACTAACCAAGCTCAAGAATAATCTCTGGAAGATACCATTCGTGCTGGACAAAGCTTCAAGCTTGCAAACCAAAGATCCCAGTATGAAAAGTTGGCAAGGGAGTATAAAAGATGCTGCTTACCAGGCTGTGGATGTTCTTGATTTATTTGACTATCGCTTTTTTGAAGCAAAAGCTGAAGACATAGAAAAGGTGCTGGCCGAGTCATCTGATTATGCTACTGTTAACTCTTCATCCTCCGCAACAACCAACACCACTGCCTCTACCAGCAGCAGCTCAACAGTAAAACGGTCAGTTCGTGTTCTGAAGCGTGTCCTTTTTTCTGATGAAGACCTTAATAAGTTAATTGCAATTTTGGAAAAGTTTGATAAGATTTCTAGTGAGATGCAAACATTCTTAGAGCTTGTTAACCCAAGGAATAAGAAGCCAGGAAAGGCACTTCAGTGGCGCAGAACAACCTCTATGCTAGGTACCACAAGATTGATCGGTAGAGGCGATGAAGAAACACAGCTAAAGAAATTACTAGAACAGACAAATGATTGGTGCAGAAAACCATATTCTGTAATAGCAATAGTTGGAGTCGCCGGCGTTGGTAAAACTGCGCTACTGCAGAGGGTATACAGCCATTTTCGTGATATAGGACATTTTGACATCATGGCATGGCTCTATGTCTCAGAAAAATTTGGTGTCAAACGCCTCACAAAAGAGATGGTACAGTCACAGAAATGCCGTAGGCATAAAAGGAAAAGAGGTGGAACCAGCAGGGTTTCATGGGATGGCTCCATATCAGCTGACTTGAACAGCATCAGTAACTTAGATCTAGTTCAGAGAATACTTGAGAAAAAGCTAAATGGGAGCAAGGTTTTGGTAGTACTCGATGATGTTTGGAACGAAATGAGCAGCAAATGGGAAACATTGTGCAAGCCCCTCCAGTTTGCTAGTATGGGCAGCAAAGTGGTGCTTACTACTCGAAGTGAAAAGGTTGCAAAGATAAATGGAGCAACAGAGATAATACATTTAGATGGGTTGAAAGGCAAAGAGTATTTGGATCATTTCCAGCAATGCGCGTTTGACAACGCAACACCGTCAGATTTTCCAAGATTGGTGCAAATTGGTGAACAATTGGCAATGAAATTGGCTGGTTCACCACTAGCAGCTAAGACGGTAGGAGCTGAACTGAAACTGAAGCTACAGGAAGACCACTGGAAGGCTGTCCTTCAGCTCAAATTGTGGCAGATTGAACAGACAGCAGATGATATTATGCCAGCATTACGATTGAGCTATGAGCATCTTCCAGATCACTTGAAGCAGTGCTTTGTTTACTTTGCATTGTTTCCTAAGAACTACCAACATCGGGATGATGTGCTCATACAGATGTGGCGAGCCCATGGTTACATTCAAAAGGAAACATCAGATGAAAATGCATATCGTTATATTAATGATCTCTTACAGCTTTCATTCATTAAGAAAGCAGCCAATCTAGATAACCATTATGTTGTTCATGACTTGCTACATGATTTGGCAGAATCAATCTCCAATGGAGAACACTTTCGAATTGAAGATGATTTTCATGTTAGTATTCCAAGAAATGTGCGGCACCTATATGTCAATGCAAGCAATATTTCTAAGGTGTGCATGAGTTTGGTTGAATCCCAGGAGGGATTGGCAGAATCCCCGGATTTGAAGAAAAATCTAAGGAGCCTAATAATATGCAAGCACCATGCTCCTGGAGAAAGAATCCCTCCAGATAACTTCAACGATGTTCTTAAAGAAACGCTGCATGATCTAAGGAGCTTACGTGTGCTCGTACTACAACATCCAGATGGTATTCTGCCAGATAATATTGAACATCTAGTCCACCTTAGGTATCTCGACATAAGTGAAAGCAAGATATTCACCAGTATCCCAAAATCATTATTCAGATTATACCATTTACAGGGGTTTATTCTCCAGTCATATTGTCAGCATAATCTAGGGAAAGAACTGCAGAAGCACATTAGTAGGCTGACAGCTGAGCCAGTTAAAATACTTAGCCCCATCCAGAAGTCGAGGCATCAAAATGATAGGTAA

>B73_Zm00001eb089490

ATGGATCTTGTGGCCGGCGCCGTGGGCAGCATCATCCGCAAGCTCGGCGAGCTGCTCCTGGCTGAGTACCAGCTGCAGGCGTGCCTGCCGGAGGAAATCGAGTCTCTGAAAAATGAGCTCGAGAGCGCGCACGTGGCTCTCCGCACCGTGGCGGAGGTGCCGCCGGAGCATCTTGATCAACAGGTCCAGCTCTGGGCTCGCGAGGTCAGGGAGGCGTCGTACGACATGGAGGACATCCTCGATACCTTCCTCGTCAACGACGCACCGGCTGAGAAAAAGGATGGCCTCGGCAAACGTCGTCGTCTTGGTCGTCGTCTCCTGGACAATATAGCTGGCGCCATGGAAAAGATGAGGAAGCTGTTCAAAAAGAGCAAGGAGTGTCACACCATAGCTGGCGCCATAGAGAAGATGAAGGAACGGCTCCGGGAGGTGGCTGACCGCCGCGACAGGTACGCCGTTCCGGTGGCAGCGCCTGCGCCGGCGAGGACGCTGGATCCTCGCCTCGCATACATGCACAGGGAAGCGGCACAGCTGGTCGGCATGGACAGGTCCAAGGCTGAGGTCATGGCCATGCTTCTGCCGCTGCCGTCGTCCCGCTGCCCCGAGGACGACATCGACGTCTCTGCCAGTGGCGGTGACAAGATGAAGATAGTTTCTGTGGTCGGAGCTGGTGGCCTGGGAAAGACCACTCTTGCCAAGGCCGTCTACGACGAGCTCAAACCGCGATATGACTATGAAGCGTTTGTTTCGGTTGGCCGGAAACCTGACCTGGTGCAAGTCTTTACCAGCATCTTCTTCCGTCTCGACGTACATAAGCACGAGGCCATTCGTGAAGTAAAGGACCTACAGCTGTTGACCGACGGACTACGAACATTTCTACAAGACAAGAGGTACTTGATCGTTATCGACGACGTTTGGGATACAGAATCTTGGGAAACAATCAAATTAGCTTTTGATCAGAAGAATAAGCAGAGTAGGGTAATCACAACCACTCGCAACCGACAAGTAGCTTCCAGCGAGAAGGTTTACGAGCTACATCCGCTCCCTCATGACAGCTCGAAGAAGCTATTTTATATGAGGCTGTTTGGGGGTGAGGACAAATGTCCGGCTAATCATCCTGAAGAGGCGTCTCAAAGGATTCTTAACAAATGTGGCGGTGTACCATTGGCTATCATCACAATGGCAAGCTTGCTGGTGGGTAAATCGAGAGAAGACTGGTTGGAGGTGTGCAGCTCTCGCGGTTTCTACCGCGGCGGCGGTAAAGATAACAACAAACAAGTAGATGACACCGTGTGGATACTGTCTCTGAGCTATTATGACCTACCTTCATATCTGAAGCCTTGCTTACTGTACCTAAGTGTGTATCCAGAAGACTATGAGGTCGAGAGGGAGAGATTGATATGGAAGTGGGTAGCTGAAGGTTTCATCGAGAAGAAAGCAGGAAGCAGCAGCAGCCTGTTTGAGCAGGGAGAGGAATACTTCCATGAGCTCATAAACAGATGCATGATCCAGGCGGTGGGGGACGACAAAGAGGTTGCTGACGCCATATTTGGTTGTCGTGTTCATGACATGGTGCTTGATCTCATCCGTGACATATCAAACGAAGAAAACTTCATCACTGTCTCATACGATGATGGTAGAAGAGGCGCAACGTCGTCGTCGTCGTCACGACACGTGGTGCGCCGGTTAGCTCACCAAAACAGAAGAATAACGGAGGAGGACAACCCTGTGAGTGGCAGCATGAGGTCACTGGTTGCCTGTGGGTGTGATATGGATGGTTGGGTCTTGCACACGAGCTCTAATAAGCTGCTGCGTGTGCTAGCTTTAGAGAAATGCACGCCACCATCTATGGACATCGGACATCTTGGAAAACTGCTTCATTTGAGGTACCTTGGGTTACATGGTACTCGCATCAAGACACTCCCAGAGGAAATAGGATCCCTCAAGTTTCTGCAAGCACTGGATTTAGAGGACACTAAAATATCACGGCTTCCACAGACTGTTTGCCTGCTAACACAGCTGATGTACCTACGCGGTGCAACCGGGGTCACAACATTGCCTGATGGTTTCTTGGGGCAGGTGACGTCACTAGAGGAGCTCCATATATGTCTTCCCACCAAAGATGACGAGTACAGCCAGAAGAAGTTCATGCAGGATATGGGCAAGCAGGGAGAAATCAGGATGCTCGTTTTGTATGGGAACAGAATTGAGTTGGATCCGTGGATGCAGTCCAGTCTAGTGCAATCACTAGGCGGTCTGTACAAGCTCCAGACCCTTGTGGTGAGGCATTATGCAGATGGGGTAGCAGCAGCACAGGGCAGCTGGGACACGGCGAAGCTTCGGCGACGTCTCCGGATTTTGAACTTAGATGTCCTCCGGTTCCATCGTGTACCATCGTGCATCGATCCCGCGCGCCTCCCCAACCTCTCACACCTACAACTGCTTGTGGTTCATCTGGACGAGGCAGGTCTGAGAGCACTGGGCGGCCTGCCAGAGCTCACCTACCTCGCGCTGTCGTTGAAGCCTCGTTCGCTGAACAGCTCATCATGCAAGGCTACGGTAGCTGATGTTGTTGCCGCAGATGGCTTCTTCCTCAAGTTGAGATCGCTCAAGCTGTATGGCTGGATGGTCCAGTTGGTGCCCAGCGAGGACTCGGCAAGTGTTTCGCTCAGCATCTGGAATGAAGGAGCGGAGGGTTCCACGAGAGACTGCACCGCCGGGAGGGTAGCACCTGCTATCATGCCAGACCTCATACATCTGGAGTTCAATGTCCCTATCGCAGCCTTGTATAAGATAACAAATAATGGATGCAGCTGTTACAGCCTCGGCTGGGAGTGCCTCCCTTCGCTACACAAAATCAAGGCGCTTGTCGACTATGACGGCACCTACACTGCTGATTTCGAGAAGCCAGTGGCTGAGATGATGCAGGCAGCAAAACTTCATCCCAACCAACCCATAATTGAGATGGTTACTCAATTATAA

>B73_Zm00001eb091490

ATGGAGTTTGCTACGGGTGCCATGGGTACCCTCCTCCCCAAGCTGGGCATGCTGCTGCAGGAAGAACTCCACCTGAAGAATAATGTGAAGGAGGGGATCAAGAGCCTCACTGCCGAGCTTGAGAGCATGCAAGCTGCACTTGTGAAGGTGTCTGACGTGCCATTAGACCAGCTTGACCCAAACGTCAAGATTTGGGCTAATGAAGTCAGGGGGCTGTCCTATGATATTGAGGACAGACTCGACTCCTTCAAGGTGCGCATGGAGGGTCTTGATTCAACCAAGCGCAAAACCATCATGGGATTCATCCAACAAACCCGTGGCTTGGTCACCAAGTTCAAGATTCGCCATGTAATATTTGATGACATTAAAGACTTCGGGAGCCAAGTAAAGGAGGTGAAGGAGCGGTATGACAGGTACAAGGTGCATGATGTTGTAGCTAATCCTATCGCAACCACAGTTGACCCTCGTCTCTTGGCTATGTATAACAAGGTTTCCGACCTTGTTGGCATTGACGAAGAAGCTAAGGAGCTAATGAATAATTTGTTTGAAGATGGTGACGAGCCAGCGAAAAAGATCAAGACAGTCTCTGTTGTTGGATTTGGAGGACTCGGCAAGACTACTCTTGTTAAAGCAGTCTATGACAAGGTTAAGAAGGAGTTTGATTGCAGTGCTTTTGTATCAATAGGTCAGAAATGTGATCTCAAGAAAGTTTTCAAGGACGTTCTTTATGATCTTGACAAGCAAAATCATGAAAATATCATTGCATCAGAAATGGATGAAAAACAACTCATTGATAAGCTACAGGAATTCCTTGCAGACAAGAGGTACTTGGTTGTTATTGATGACATATGGGATATATCAACATGGAAGCTGATTAGATGTGCTTTGGTGGAAAGTAACCCTGGAAGTAGAATAATCATAACTACTCGCATTTGTGAAGTTGCCAAAAAGGTTGGTGGTGTTTACAACAAGAAACCACTCTCTCTTGATGACTCCAAGACATTATTCTATACTAGAGTATTTGCTGGTGAAAGCATGAGTCTTGATAACATATCTGGTGAAGTGTGCAACAAAATCCTAAGAAAATGTGGTGGTGTGCCATTGTCCATCATTACGATAGCTAGTCTGCTTGTTGGTAAACAGAGGGAGGACTGGTCTAAGGTGTATGATTATATTGGTTTTGGGCATGAAGATAACGAGGTTATTGGGAACATGAGAAAGATATTAGCTTTCAGCTATTACAATCTACCTCCTTATCTAAAGACATGCTTATTGCACCTAAGCATATTTCCAGAAGATCACAAGATTGAGAAAAATTCATTGATATGGAGGTGGATAGCTGAAGGTTTTGTTATTGGCAGAGAAGAACTAGGGTTATTTGAGGTTGGAGAGAGCTATTTCAATGAGCTCATAAATAGAAGCATGATCCGGTGGATAGAGCTCTCTAGTAGAAGCAAGATTCGAGATGGTTGTGGTATTCATGATATGGTGCTTGATCTTATCCGCACTTTGTCAGGTGAAGTAAACTTGGTCACGGTATCAGATGTGGAGCAGCAGTGTACCACATCATCATCATATTCACCAGTCAGAAGCATTAGCGCTCGAAGATTAGCCTTCCACAAAAAAAGAAGCATTGAACACAACCCTGGCACAGAAATAGGACAGGTGAGATCATTCAATGCTTTCAACTGCTCTGGTAGTAGGATGCCCCGACTTTTAAGCTTCAGGGTCTTACGTGTACTAGCTCTGGAGAACTGTAATTTCTCAGCAGGAAACTGTTGCCTTGGAAATATTGGCAAATTGCATCAGCTGAGGTACCTAGGGCTAGTGGAGACATCCATTCGTGACGACTTGCTGCCTGGAGAAACAGGACGCCTCAAGTTTCTGCAGACACTAGATGTAAGACGAAGTGGCATAAAAATATTGCCAGCGTCTGTTGGTGAGCTAAGGAAACTGATGTGCCTGCGTGCTACCGAGGGCACAAGGATGATGGCCGAGATTGGGAAGCTGGCGTCACTGGAAGAGCTTGAGGTACACTCTGTGGACAAGTCGCCAAACTTCGCCACGGGGCTGGGGCAGCTAACCAAGGTGAGGGTGCTTGAGATCCATTTCGACGAAATGGACGAGAGCACAGAGAAGGCTCTCATGGAGTCCCTGCGCAACCTGCGGAAAATCCAGAGTCTGCAGATATGGTCCAAGAAGGAGAGGACAATTGACCTTGGCGGCTTGTTGGAAGACTGGACGCCAACCCCTTCAAATCTCCGTCAACTGATGCTGCGTGGCATCCATTTGCCCAGGCGTCCGTCATGGATTGATCCCTCATGTGTCCCGCTCCTCTCGTACTTGTCGCTCACGGTCCAGGCCGTGCAAGTGCAGGATCTAGAAATCCTCGGGAGGCTGCCGTTGCTCAGCTACCTCTACATCTGGAGTGAGGGCATCAACTGCTTATCCTATACTGCTACCAGCAGAGACGAGTTTCAGAATCTGAGATATCTGGACACAAACCTGGAGATCATGTGTGGACAGCAGGGAGCACTGCCTATGGTTGAGAAGTTGACATGCCGTGCCAGCATGGGGAAGTATGTTGCCTTCGCCAGGAGCAGCATGCCTTTCGACGATGGCAGCGTGGTGAATCCTGCTGCTGTTGCTGAGGCAGAATTGCCAGTAATTCTACCTTTGGACATTGGCTGGCCCGTGAACATGCCTTGCCTCCGGGGCATCACCTATTTGCTGGACTACCAGGACTGCAGTGCCAAGGAGTGGGCTCATGTGGAGACATTGCTCATGCACGTGAGAAAAATCCACCCCAACTGTCCACCCTTCCGAATCAAAAAGAACTGCAGAGACAAGGAGATTACTATGATCGACGCAATCATCTACCTTGAAGATGTCAAGGATGTGTTCAAGGACAACCCCTCCAAATACTCGGAGTTTCTTGATCTCTTGCAAGATTATAGGAGAGACAGAATCAAAATCAAAGATTTGATCATCCGTCTCAAGACCCTATTTACTGGACATGATCCTAATCTCATCCTTGACTTCAGCGTCTTCCTGCCCGGGGAGTGGGCCATCACTCTCGGGGACCTGTAG

>B73_Zm00001eb091500

ATGGAGTTTGCTACGGGTGCCATGGGTACCCTCCTCCCCAAGCTGGGCATGCTGCTGCAGGAAGAACTCCACCTGAAGAATAATGTGAAGGAGGGGATCAAGAGCCTCACTGCCGAGCTTGAGAGCATGCAAGCTGCACTTGTGAAGGTGTCTGACGTGCCATTAGACCAGCTTGACCCAAACGTCAAGATTTGGGCTAATGAAGTCAGGGGGCTGTCCTATGATATTGAGGACAGACTCGACTCCTTCAAGGTGCGCATGGAGGGTCTTGATTCAACCAAGCGCAAAACCATCATGGGATTCATCCAACAAACCCGTGGCTTGGTCACCAAGTTCAAGATTCGCCATGTAATATTTGATGACATTAAAGACTTCGGGAGCCAAGTAAAGGAGGTGAAGGAGCGGTATGACAGGTACAAGGTGCATGATGTTGTAGCTAATCCTATCGCAACCACAGTTGACCCTCGTCTCTTGGCTATGTATAACAAGGTTTCCGACCTTGTTGGCATTGACGAAGAAGCTAAGGAGCTAATGAATAATTTGTTTGAAGATGGTGACGAGCCAGCGAAAAAGATCAAGACAGTCTCTGTTGTTGGATTTGGAGGACTCGGCAAGACTACTCTTGTTAAAGCAGTCTATGACAAGGTTAAGAAGGAGTTTGATTGCAGTGCTTTTGTATCAATAGGTCAGAAATGTGATCTCAAGAAAGTTTTCAAGGACGTTCTTTATGATCTTGACAAGCAAAATCATGAAAATATCATTGCATCAGAAATGGATGAAAAACAACTCATTGATAAGCTACAGGAATTCCTTGCAGACAAGAGGTACTTGGTTGTTATTGATGACATATGGGATATATCAACATGGAAGCTGATTAGATGTGCTTTGGTGGAAAGTAACCCTGGAAGTAGAATAATCATAACTACTCGCATTTGTGAAGTTGCCAAAAAGGTTGGTGGTGTTTACAACAAGAAACCACTCTCTCTTGATGACTCCAAGACATTATTCTATACTAGAGTATTTGCTGGTGAAAGCATGAGTCTTGATAACATATCTGGTGAAGTGTGCAACAAAATCCTAAGAAAATGTGGTGGTGTGCCATTGTCCATCATTACGATAGCTAGTCTGCTTGTTGGTAAACAGAGGGAGGACTGGTCTAAGGTGTATGATTATATTGGTTTTGGGCATGAAGATAACGAGGTTATTGGGAACATGAGAAAGATATTAGCTTTCAGCTATTACAATCTACCTCCTTATCTAAAGACATGCTTATTGCACCTAAGCATATTTCCAGAAGATCACAAGATTGAGAAAAATTCATTGATATGGAGGTGGATAGCTGAAGGTTTTGTTATTGGCAGAGAAGAACTAGGGTTATTTGAGGTTGGAGAGAGCTATTTCAATGAGCTCATAAATAGAAGCATGATCCGGTGGATAGAGCTCTCTAGTAGAAGCAAGATTCGAGATGGTTGTGGTATTCATGATATGGTGCTTGATCTTATCCGCACTTTGTCAGGTGAAGTAAACTTGGTCACGGTATCAGATGTGGAGCAGCAGTGTACCACATCATCATCATATTCACCAGTCAGAAGCATTAGCGCTCGAAGATTAGCCTTCCACAAAAAAAGAAGCATTGAACACAACCCTGGCACAGAAATAGGACAGGTGAGATCATTCAATGCTTTCAACTGCTCTGGTAGTAGGATGCCCCGACTTTTAAGCTTCAGGGTCTTACGTGTACTAGCTCTGGAGAACTGTAATTTCTCAGCAGGAAACTGTTGCCTTGGAAATATTGGCAAATTGCATCAGCTGAGGTACCTAGGGCTAGTGGAGACATCCATTCGTGACGACTTGCTGCCTGGAGAAACAGGACACCTCAAGTTTCTGCAGACACTAGATGTAAGACGAAGTGGCATAAAAATATTGCCAGCGTCTGTTGGTGAGCTAATGAAACTGATGTGCCTGCGTGCTACCGAGGGCACAAGGATGATGGCCGAGATTGGGAAGCTGGCGTCACTGGAAGAGCTTGAGGTACACTCTGTGGACAAGTCGCCAAACTTCGCCACGGGGCTGGGGCAGCTAACCAAGGTGAGGGTGCTTGAGATCCATTTCGACGAAATGGACGAGAGCACAGAGAAGGCTCTCATGGAGTCCCTGCGCAACCTGCGGAAAATCCAGAGTCTGCAGATATGGTCCAAGAAGGAGAGGACAATTGACCTTGGCGGCTTGTTGGAAGACTGGACGCCAACCCCTTCAGATCTCCGTCAACTGATGCTGTGTGGCATCCATTTGCCCAGGCGTCCGTCATGGATTGATCCCTCATGTGTCCCGCTCCTCTCGTACTTGTCGCTCACGGTCCAGGCCGTGCAAGTGCAGGATCTAGAAATCCTCGGGAGGCTGCCGTTGCTCAGCTACCTCTACATCTGGAGTGAGGGCATCAACTGCTTATCCTATACTGCTACCAGCAGAGACGAGTTTCAGAATCTGAGACATCTGGACACAAACCTGGAGATCATGTGTGGACAGCAGGGAGCACTGCCTATGGTTGAGAAGTTGACATGCCGTGCCAGCATGGGGAAGTATGTTGCCTTTGCCAGGAGCAGCATGCCTTTCGACGATGGCAGCGTGGTGAATCCTGCTACTGTTGCTGAGGCAGAATTGCCAGTAATTCTACCTTTGGACATTGGCTGGCCCGTGAACATGCCTTGCCTCCGGGGCATCACCTATTTGCTGGATTACCAGGACTGCAGTGCCAAGGAGTGGGCTCATGTGGAGACATTGCTCTTGCACGTGAGAAAAATCCACCCCAACTGTCCACCCTTCCGAATCAAAAAGAACTGCAGAGACAAGAAGATTACCTTGATCGACGCATTCAGCTACCTTGAAGCTGTCAAGGATGTGTTCAAGGGCAACCCCTCCAAATACTCGGAGTTTTTTGATCTCATGATTGATTACAAGAGAGACAGAATCAAAATCAAAGATGTGATCATCCGTCTCAAGACCCTATTTACTGGACATGATCCTAATCTCATCCTTGACTTCAGCGTCTTCCTGCCCAGGGAGTGGGCCATCACTCTCGGGGACCTGTAG

>B73_Zm00001eb112770

ATGGGTGAGTTCCTGGTCAGCGCCTCCACGGGCGCCATGGGCTCAGTCTTGTCCAAGCTGGCCACCATGCTGAGCGACGAGTGCAAGGCATTCAAGAATGTGCGCGGCGACATCAAGGACCTCAAGAAAGAGCTTGAGCACATGCACGCTTTCCTCCTTGCCATGGCGGACGTGGAGGATCCCGACGAGCAGACCAAGCTCCGGGCAAGAGATGTGCGGGAGCTCTCCTACGACATCGAGGACACCATCGACAAGTTCACGGTGCATTTTGAACGTGAATGCAACCCCAAGTCTCGGGGCTTCAAGGTGCTCGTCGATAAATGCAGGAAGCTGATGACAAGCGACATCAAGATCCGTCGTCAGATCGCCAAGGAGGTGAAAGACATCAAGAACCAGATCAAGGACACCAGCGAGAGATATGCGAGGTACAGCACTACCGCTGGTGGCTGCAGTACAAGGAATGTGGTGGTTGACACACGAGTTCTTGCTGTGTTTAAGGACACATCAGAGCTTGTTGGCTTAGATGGCCCGAGTGATGAGCTTGTCAAGTTGCTCAAGTTTGAGGAGGGCGACTCTGCGCATCATTTGGAAGTTGTCTCTATTGTTGGATTTGGGGGGCTAGGAAAGACAACTCTGGCTAACCATGTCTATAACAAGCTTGGAGAAAACTTTGACTGTCGGGCTTTCGTTTCAATTTCACGAAATCCTGATACATCCATGATTTTGAACTCTATCTTATCTCAAGTCGGCAGCCAAAAGAATGCCAATGGTTTTGCAGAGGTTCAACAAGTCATCAGGGATATAAGAGAATTCCTCAAAGACAAAAGGTATTTTATAGTAGTTGATGATATATGGGATGTACAAGCTTGGAAAATTTTGGAGTGTGCATTTGCTAAGGGTGGTCGTCCTGGCAGCAGAATATTGATCACAACCCGAAAAATGGAAGTTGCTAAAGTATGTAGTTCACCTCCTGATGAAGATCACATCTACAGGATTAAAGCACTTAGTGATGCTGACTCAAAAAAGTTATTTTTCAAGGGAATATTTGGATGCGAAGAAAAGTGCCCTTCTGAACTTAGAGAAGCCTCTAATGGAATTCTGAAAAAATGCGGTGGTTTACCATTAGCGGTAATTACTATATCTAGTTTGCTGGCAACCGGGCAGAAAAAAGAAGATCAGTGGGAAAGGGTGCACAGATCTATTGGCTTTGCATTTGGCAGGAGTTGTGAGGTTGACGGAATGCGAGGGATATTATCCTTGAGTTATTTTGACCTTCCACACTGTTTACGAAGTTGCCTCATGTATTTGACTCTATTTCCTGAAGACTATGTGATTGAAAGGGTTCGTTTAGTGCACAAGTGGATTTCCGAGGGTTTCATCGTCTGTGGGCAGGATACTAACAATCTTGTAGAACTAGGAGACATGTATTTCCATGAGCTTATTAATAGGAGCCTGATACAACCAATTGACATTGGATATGATGGTAAGGCACGGGGCTGCCGAGTGCATGACACTATCCTTGATTTCCTCGTCCAAAAGTCGGCGGAAGAGAACTTTTCTGCTACGCTGATGCTAAGAAGCAATCAGCAGCAGCAGCAGCAGCAGCAGGAAGCCAGCAAATTCGTCCGTCGGCTATCGCTGATTGCACACGGGACTAGTGATCAGGAAGAAGAAGCCAACATATATAATGTCCTAGAAGAATGCATGGATACGTCTCATCTTCGATCATATAGTATTTTTGGGCGGGGCTCCAAGAAACTGTTGTCTTTTATTCCGTACTTGAACTATTTGCGTGTGTTGGACATAGAAGGCTGCCCCACACTTGAAGACGATGATCTGACGAACATTGGAAGGCTCTACCAGCTAAGGTACCTGAATGTTAGTAAAACAGGTATAACTTCATTTCCGACAGACATGGAAGGTTTGCAGCACCTAGATACACTTAATGCAAGATATTGTCGTCGTCTTAGGAACCTGCCAAAAGCTTTCATCTGGTTTAAGCAATTGGTGCGCTTGATCGTCACATATGGCACTAGACTACCAGACGGGATTGGAAATTTGAAGAACTTACAAGAGCTTGGTGATGTTTACGTGCCCAAGTGTTCGCTCAACTTTCCGCAGGAACTAGGTGAGCTAACAAATCTGAGGAGTCTGAGTATTGGGTTGGACACGATACAAGGCGACAAAGCATGTTACATGGAGAAGCTGGTGTCCTCTCTCTGTAAACTGGACGCATGCAACCTCAGTGATCTGTCTCTTGTATTAGCCCTGCACGGAGACGGACACGGCGATGACATTGTGTCGTTCCCTGCTCTAAGTAGCATCCGAAAGGTTTGGCTCTCGAGTCCAGGAATCTCCAAGACCACCACCAGATGGCTAGTCTCGCTCGTCAACCTAGAATACTTGAATATCTACGTTGCTGATGTGATAGAGCAGCGAGACATTGAGTTGTTAGTTGGAAGCATACCTACTACTCATCTGCTTGATCTTACAGTAACTGTCCGTAGGACCTACAACTTCGTCGGGCCACAAGTCTTCATCATCCGAGGGTTTCAACAGGTGCAGAGATTTTCATTTCGTTCTGATGATGACTCTTCAGGGACGTTGGTTGTCCAAGTTGAAGGAGGATCCGGAGGGGCCATGCCAACGCTGAAAGAACTTAAGCTCAAGATCCACCCACCAGCGTTAAATTCAGCTGTACTAGGTGGTTTTGATTTTGGCATTCAGCACCTCTCCTGCCTTGCGAGCTTCACCCTCAGGGGTGTAAAAATGGCTTCTTCAGATGTCGAGGCTGCGGAGCGTGCTTTCAAGTCCATGGTCGAGACCATTGTTCTGAACGACCGGCTAAGGCATTTGGCGGCTCCATCTTCATCACGCTTTGTTGGAATGTAA

>B73_Zm00001eb113900

ATGGCGGAGATAGTCACCGGGGCGATGGGCACTCTCTTGCCCAAGCTGGCCAACCTGATCAAGGAGGAGTATAACCTGCAGAAGAAGGTGAGGGGTGAGATCATGTTCCTGGAGGCTGAGCTCAAGAGCATGGAGGCTGCTCTCATCAAGGTCTCCGAGGCACCCATCGACCACCCACCTGACATCCAAGTCAAGCTCTGGACAAGGGAGGTGAGAGAGCTGTCCTACGACCTCGAGGACAGCATCGACAGATTCATGGTGCGAGTTGGCGATGGCAAGCCACATAGTTTCAAGGGATTCATTGATAGAAGCCTCCACCTGCTGACAAGGGGCAGGATTCAACACAGCATCGGCATAGACATCAAGGAGATCAGGAGCCGCATAAAGGATGTGAGTGAACGGCGTGACAGGTACAAGGTTGATTTGGTTCCTTCCAAGCCTGTTGGCAGAAGCATCGACAACCTGCGGCTGTCGGCTCTTTACAGAAAGGCGACAGAACTTGTTGGCGCCGAAGAGAAGAGCAGTGACCTTGTGAGAAGGCTCATGGAGGGCGACAAGGAGGCATCCAAGCAGCCAGTTGTACTGTCTATTGCTGGCTTTGGAGGGTTAGGCAAGACTACTCTTGCTAATCTTGTGTATGAGAAGATTAAAGGGCAATTTGGCTGTGGGGCATTTGTTTATGTGTCTCATAATCCTGATGTCGTCAAGGTTTTCAAAAACATGCTCTACCAGCTTGATGGAGACAAATACAGGGACATCAATCAAGGAACATGGAGTGAAGAACAACTAATCTGGGAACTGAGGAAGTTCCTTCTGCACAAGAGGTACTTCATTGTCATTGATGACATATGGAATACTTCTGTGTGGGAAACAATCCAATGTTCTTTGATGCACAATGAATGTGGAAGTATAATAATTATCACAACTCGTAATATTGATGTTGCAAAACAAGCTGGAAGTGTTTATCAAATGGAACCTCTTTCTCTCAGTGACTCAACAAAGTTATTCTGCCAAAGAATTTTTGGCAGTGAAGACAAATGTCCTCCACATAATTTAGCTGAAGTGGCTGGTAAAATCTTACAGAAATGTGGTGGTGTACCATTAGCTATCATTACCATGGCAAGTATGCTAGCCGATAAAACTGGAAAGGAAATAAATACACATAACTATTGGTCACATGTGTACCAATCCATGGGTTCTGGTCTAAATGGCAGTACTAATGTGAAGAATATGAGAAGGATACTATCAGTTAGTTATTATGACCTACCTTCACATCTAAAGACTTGCTTGCTATACCTAAGTTTGTTTCCAGAAGACTACAGAATTAAAACAAGAGGTCTCATATGGAAATGGATTGGTGAAGGTTTTGTCCATGAAGAACAGGGGAAGACCTTATATGAAGTAGGTGAGGATTACATCGAAGAGTTAATTAACAGAAGTATGTTGGAACCTGTAGATATTGGCCGTGATGGTAAGACTGTTTCTTGTCGGATACATGATATGGTCCTTGATCTTATCAGTTTCTTGTCAAATGAGGAGCATTTTCTAACAAAAGTAGGTGAGCAACAGCCCATATCTCTTGATCTGCCTAAAAAGATCCACCGGTTATCCCTCCAAATTAGCCAGGAAGAGGAAGTCAAGCAGCTGGCTACAATGAGTTTCTCCCACGTAAGATCACTTACTGTGTCCACTAAAGTGTTCCAGTTGATGCCAAAACTTTCGGCCTTTCTGGTCTTACGTGTATTGAATTTAAAGAAATGTAAGGGAGTGAGGAATCACCACTTTAAAGATATTTGCAATATGTTTCACCTGAGATATTTGAGTCTCAATGCGGAATTTATTACTGAGATGCCACGGGAGATTCAGAATCTACAATTTTTGCAAGTACTTGACATAAGTAATCTTGGGCACAAAGTAAAGATGCCAACCATTATTCACTTGCGACAGCTACTGCGTCTTTGTTTTAGGCCGATGTGGGGCATAAGACTGCCAGATGGATTCGGAAAACTAACCTCTCTACAAGAAGTTAAAGGGATCATAACTATCAAGTTACCAAGCATGCTGCATAATCTGGGGTGTCTGACCAATCTCCGGACCTTGGCCATCGACTTTTGTGATTGGGATGAGAGCTATGAGGAACCTTTCATCCAATGTCTATCTAACCTTGTCAGCCTCAAATCCATGGAAATAAAAGGTACCATGGTGAGCAGCCTATGTTCCGAATGTGACAAATTGTACCCTGGTCCTCAACATCTTTGCTCCATTGATATTGAGTCGACTGCAGTGCCAAGATGGATGTCATCGCTCTGCTTCTTGTCTAGCATAAACATTGAACTATTAGCTCTGGGAGCACAGGATTTTCATGTCCTTGGGAGCATACCATCTCTACGTTGTCTCAGTATACATGTGAAGGAAACCAGAGATGAAAGATTGGTCATTGGCAAGTGTTATCCATTCCGGTGCCTAACTGAGATGCAAATCGATTATGAATCCATGGCGGTGGTGTTCGCACCAGGAAGTATGCAAAACCTCAAAGAACTTCATTTAGTGTTCGGGGTGAAAGAGGTAATGCATAAGTATGGTGATTGTAACTTTGGTTTGGAGCACCTCATGTCACTGGAGCATGTCTCTGTTAAAACAATGTACAGTATCATGCCCGAGGAGGTGGAGGCCGTAAAAGATGAATTTCAGAAATCCCTGGACATGAATCCTGGCAAGCCCACGTTGATAGTAGATTATAAGTATCCGATAAAAAGGAAGATTAGGTCTCATGCACAAGCAATAAGAGCAGCAATTTTGTTCGCTAATGCAGGCCGCATCCCTGCTACTGAAGGATTATAA

>B73_Zm00001eb115050

ATGGCGGCGCACCCACATCCACATGGTTCCCGGAAGCAGCAGCCTTCTCCTTCCAACAAGCAGGATCAGGAGGACACGAGGGTGGCAAAGATCGCGGAGGTACTAAGCGTGTTGGAGCCCGTTCGGGCGAGTATCTCTCAACAGCTGGGTGCTGGTGCTGCCGGCCGTCGCTGCGTCGTCGCTGCAGGTTACACTAGACGGCGCTGCTTGGCCTTCATGGAGACGGAGCTGAGCATCATTGCGGAGTTTCTCAAGACACTTAGCCAGCAACATCTGGATGCCGACACGAGGAGATGGATGGAGCACTATGAGCTTGTCTTCAGTAAACTCGACAGCGTCATCCAAGAAGTTGATCCCTCACGCCGCACGCTGCTACGAACGCGATTGAGAAGTGCCACGCAGTGCATCCTAAGCCGCAACAATAGATCGTATCCGTTCTCTCCTAACATAAAACAGCTTTACTATCGCAGCGAACATCCCTGCAGGTTTAAGCATCTTCTTCAAGCTAATAGTCGTCCTGAGGCCGACATCGGGCTTGCACCACCCCAACTTGGTGGTGGCAGTGGCACTGGCACCAACTTAACGGCGAGTGCCGCCCTCCCTCTTGTTGGCATCGACCGCCCGGCGAAAAAGCTCCTCAGGTGGCTCACACCCCGGGAGGAGACGGACAAGAGCCTGAGAGTCATGTCCATTGTCGGACCTCCGGGCATGGGCAAGACGACTCTTGCCATGGAAGTCCACAGGCGCAAGAAGGCCGAGGACGCTACTGTTTCCGTTTTCCAGTGCAACCTTGTGGCTCGGTTCTCCCGGGGGCCGGACAGAAACAAGCTTCTTCTCCAAGACATCCTCTCTCAAGTTTCTGATCGAGCAGCACCAGCATTGACGTCGTCCAGCCAGTCACAACGCAAGACAATAAAGCTGCTTATTCACCTCGTCTCAAAACACCTGCGAGATAAGAGGTACTTCATCATTATTGACGATATATGGGACACATCAGATTGGGAGAAGATCAAGGATGCATTTCCTAACAATAATCTTGATAGCAGAATATTGATCACAACACGCGTTATAAGTTTGTCATGGGTATGCTGCTTTGATTCTGATGATGGGCTTGTGCATCAGATGAAGCCTTTGAATCGGACAGACTCGGAAAGGCTGCTTCTGGCAAGCGCCTTTGGTCCTTCGTCTGTGGATGATTATGATTGCCAGCAGCCATTGTGTGATGAAATACTGATCAGATGTGAAGGTGTACCATTGTTCATAATCGGTATGGCGGACTTGTTTAAAGAACAATTATTGCAGCAGAAGGACGCCGAGGATCAGAGGACCCTTGCAGTTTACAGCAGCGAGCTGGAGCAAGGCCCCCAACGACTACCGAAATGTATCGAACGAGCATTGTCCCTTGCTTATGATGACCTTCCTTATGAGTCCAAGCTACAATCTTTATGCATGACCATGTTTCCTTCTGGTTACAAGTTCGACAATGACCGTTTCTTCTTCTTCAGATGGAACGAAGACAATGAAGTGGCGATGCGCTTTACTATTCCGCATCTGGCGGACAGGAATGTCATCACGCGTGTTGCTGGTGCTGACTGTAGGCACTGTCCGGATGAAGAAGAGGGCTGCCACTGTCAGTGGCACGTCAATCATTTTATGCAGCAGTTCCTTGCCTCCAAATCTGCAGAGATGGGTTTCTATTTCACCGCCAGTAGCCTCAAGAAGTACTTATTACTAGCAGGAGCAGGAGCTAACAACAACAGGACACAGCGGAGGCTAATCCTTCACCATCCTGACCCAAATCTCCCATCTCTGCTTCAACAAATTGATCTATCTCAGACACGTTCCCTGGCTGTGTCAGGTGCTGTCAGCGGCATCCCTATGGACAAGTTCGTCAACTTGGTGGTGCTGGATCTGGAAGGCTGGGAGAATCTCAAGGACGATGACCTGCTGCAGATATGCAGAAGCAAGATGTATTTCCTGACGTACCTGAGCGTCAGGAACACTCCGACAAGCAAGATCCCGCCGGAGATCAAGGAGCTGTGGAGTCTGCAGACATTGGACGCGAGCTGCACGCAGATCAGCGATGAGCTCCCACTCCAAGTGTTCAAGCTAACAAGTTTGAAGCACCTGGATCTGAGGGGGACACGGGTCAGGAGGATAAGCAAGCTGCCCAACAAGCAGCAGATTGTGGGCTCACGGGTTCCCTTGTTTACCCTTCTCGTTGGCGGCGGCGGCGGCGGTGGTGGTCCTATGGAAACAGCAGCGAGAGTGACGCCCGATGTACGGCATCTCCAAGACCTCGAGATGCTGGCCACTGTTGACTTGACCGAGAACCCCGTGAGCTTCCTCCGGGCTCTCGGCGACCTAAAATGGTTAAAGGTGCTCAAGATCACGTGGTCCTTCTGCCACTCCACCGACGGAGAGTGCCGTGCGGCGCTTCTGTCGTCCATCGGCAGGTGGTCCCACCTCCAGTCCCTAACCATTCACTGCGGACTCGGCTGCTCCATGGAGTTCCTGGGCACCCTCTCCTATCCGCCCGAGCACCTCAGGAAGCTCAAGGTGACGGGGGGCGTGTTTGCAGGCGTTCTCCGGTGGCTCAGTGTGCCTTCACACCACTTGTCTTTCCTGCAGATCACCATTTGCAGCATCACGGCAGATGATCTCAAGGTGCTTGCCGACCTGGCCCAGCTGCACACCCTGGTGCTAGGCCTGGACTTCGTCCCCACAGAAGCTGTAGTGATCGAGGACGGTGGTTTCCCTCTGCTTCGGAAGTTCTTCGTCAACTGCCCAGTGCCATGGCTCGCCTTCGAGATAGGAGCATTGCCAAATCTCGCATATCTTCAGCTGGAGTTCGCTGCGACGCTGCCAACCCAGACCAGGGCTCCATCGGGCATCGCCAACATCCACAGGATCACGGACATTGCTCTGTGCTACGGGGAACACTACGCCAACAGCCCCAGCGTCAAGATCATAGTGGAGGCCGTAAGGAAACAGATCGTCGAGCATTGCAACCCGATCGACCTCTACATCAATGGCATTGAGCAAGACGATGTTCAGGCACCAGATGACTTGACAGAAGATGCAAATGTGACTCAGAGCCGAACCGGATCCGGATCACAATCGCCCAGGGAATGA

>B73_Zm00001eb116510

ATGGATAACATGCTAGGCGACTTGTACAAGCCAGATGACGGCGACGACTTCTACAGCAAAAAATACCGCGACGAGCTAGAGAATGTGTCATTCCAACTATTAGAAGAAATTACAGATGGTTTCTCTGAGAGTCGAGAGCTCGGCCGAGGATCCTTTGGAGTGGTTTACAGGGGATTGACTAAAAATGGTGATTACGTTGCTGTGAAGAAGCTTCATGCTAATGTCACTGATCTTAACCATAAGCAGTTTCAAAATGAGTTATATAACCTTGCAAAACTTAAGCATGAAAACATTGTACGGATTTATGGCTATTGCTATGAAATTAAGAAAACATTTATGGAGCACGATAGAAGAAAAATTCTTGTCGAAGAGCCGCGTATAGCGCTCTGCCTTGAGTATTTACACAATGGAAGCCTTCAAGATCATATTTCGGATGAGTTTTCTGGTCTTGACTGGGACACACGCTACAGGATTATGGAAGGGGTCTGTCGTGGTTTATATCATATCCATAGGAATCTAAAGAAACCTCTTTATCATTTGGACCTAAAACCTGCCAACATATTACTAAGTAAAACCATGCAACCAAAGCTTGCAGATTTTGGGTTATCTAGGATCTTTCGGAATGAAGAACTAACACGAACGACTAAAAGTCCTCTAGGAACTCTTGGGTACCAGCCACCAGAATACATTGAAAGAGGTGAAGTCTCAGAAAAATTCGACATATTCAGCTTAGGTGTCGTAATGATACACCTGGTTTCGGGACGTGGAGGCTTCGAAAGATATGCATGCATGCATAGTGAAGAATTTATCAATCAGGTAAAAACAAACTGGAGGAAAAGATTTAAGCAATTACAGAGGAATTCCTTACGTAAAGTCAAAGTGTACTGCCACGAGGTAGAGACATGCACTCGGATAGCACTGAATTGTGTGGAGAAAGACACACAGAAAAGGCCTGATATAGATATGGTCATCAATAATCTAAAGGACCGTAAGACCCATAGATTCAAGAGTACTTGGACAAGTAGTTTTCGGCAGGATGGAAGAATGTATGGAAGCTATATGCCATATTCAAACAGATATTTAAACAAATTGATGTCAGGCTCTATTTGCACCGAAACAAAATTTTATCGAAGAGAAAAATTACCGAATGTTGGAGAAGAATTCATTATAGGGAGAAAAGAAGAGAAACAGAAAATAGTGGCATCTTTACTACAGAGCATGACACAACACATCACTATCCTTCCTATCTATGGCATTGGAGGAATTGGCAAGACAACCATTGCAAAGTTGATTTACTATGATACAAATTTCAATAGTTACTCTCGAGTATGGGTCTATGTTTCACCGAAATTTGACTTGAAGAAAGTTGGAAACACTGTAATTTCACAACTCTCTAGTGAGGAGAGCCAAATTAATGAAACACAAATGATACATAGTTGCCTGATGAAATTACTTTCTGGTAAAAAGGTTCTGATTGTTTTAGATGACTTGTGGGAGGATAGTTTATTTCAACTGAATAATTTGAAGGATATGCTAAGTTATGGTGATAGCATCAATGTAATAGTTATAGTAACCACACGCAGTAAACATGTTGCAGAGAATGTTAGCACTAATGTTGAGCCACACAAGATAGAACCCTTGACAGATAGCATGTGCTGGGATATAATGAAACAAAGAAGCAACTTTGAAGCTAAAAATCACAAAGAACATTTGGCACATATAGGAATGGAGATCGCTGTAAAGTGTGGAGGTGTGGCTTTGGCAGCTCAATCCCTTGGGTTCATGTTGAAGCCCATGGAATTATGTGATGAATGGATTGAAGTCAGAGACAGTGACATCTGGAATAAATCTATTTCGAAGGATGCCAATTCACCAAACCATGTGCTTGCATCCTTGATGTTAAGTTGTACCAAAATGGATCGATGCTTGATATTATGCTTTATCTACTGTGCAATCTTTCCAAAAGGTCATCATATAGTCAAAGAAGAACTAATACAACAATGGATTTCTTTGGGTTTCATCCAGCCAACAAAATTACACTCCAATATGCAGATATGTGAGAAGTATATTTTGCAGCTCATAGGATTGTCTTTCATTCAAGATACAATGTCACCAAAGTATTCTCAAGCATACAAAAAAGGCCTCACATTGTTCACCATGCACGATCTCGTGCATGATTTGGCAAGATTACTAACGGCTGGTGAAATTCTGGATGCTTCCAATATTGGGACTATGGATGCGAGGCATGTTGCCCGCGAGCCTGCAACTAGGTCATTATTTCATGGGTACATGCATGCTAAGTCAAACTTTCGTAGCTTCATACGTACCAATTCAAATATATACACATACCGGCGACGGCGAGAGCCAGAAGGTAGCGGACAGCAATACAAATATGCATTGCTCACTAATTGTCACAAGCCCCTAGAGGTATTGACGGATTCGCCCAATATGATAAGGGCACTGCATTTTCGGGATTGTCATGGCTCGAAAGGACTAAAAGATTATGCATTTTTGCCTGCAAAGTTGTCCCTAAAAGTTCTGGATTTAAGTGGGTGCAACATAGCCTACCTGCCAGCTTCTATCGGTGAATTGGCTGTATTGAGGTATCTCAATGCTCCAGAGATTAAAAATGAAATGCTCCCAGACTCTCTGAGCAAACTGTCAAAATTAATATACCTCAACCTAAGTGGGTCCAATATTTCAGCACTGCCTGATTCAATTGGTGATATCGAAGGTCTAATGCATCTTGATATATCAAATTGTGTGCTTTTGTGTGAGCTCCCAGAATCATTTGTGGACCTAAAAAATCTGGTATATCTAGATTTATCACATTGCCAGATCAAAATTACAGCAAGAGTTTTTAGTGGTCTCACCAATATCCAGCATTTGAATTTATCAAAAAGTCTTATTCATGGAGGTGACGGGTTGGAGGGGCTGCAGGAAGCCGTTGGTGGTCTCACCGAACTTCGGTATTTAAATCTATCAGGGTGCTTTGAAAATCTGAGGCCAGATGAGGTATTAAGTTTTGTTGACCGTATTTGTAGGCTTACAAATCTGGTGCACTTGGATTTGTCTTTCAATTTGGGTCTTGTCAGTGTGCCTGAAAGTATTGGCAGCCTCAGGAAGCTGCACACCCTGGATCTCTTGTGCTGCCGAAACCTAGTGACGCTTCCAAAATGTATGTTCAACATGGATAGCATGAAGATGTTAAATGTTACCTGCTGTCCTCATCTAGATCAGTCTACGCTCCCCCGATACAGGTATTTTACCTTGCCGTACTTTGAGGTCCATGCTGATGATGGCGAATCTACCAGCAATATTGGTTTGCTTCGGTATGTGAATCCTACTCATGAGTTGTACATAAGTGGGCTTGATAACACAATATCTGCAGAAGAGGTTGACAGTATAAACCTAAGCCAAAAACAGGGGATCCAGTCGTTGAGTCTTTCATGGACTAGAGATGCTCAGAGATCTGTGGAGGACATGGAAGTCCTGGGAAAGCTAGTGCCACCTGATACTTTAAAATGCTTTGAGCTAAGAGGTTATGATAGTATGAGCTTTCCAACATGGGTTATGGGCATCACGCTCTATCTTCCTCGCCTTACCAAGGTTGTATTGTGGGAGCTGCGTAAATGCAATAGTCTACCACCACTGGGCCAGTTACCAACACTACAAAAGCTAGTTATAGGAGGAATGGACAGCACTTTGACAGTTGATGAGGGTTTCTGCTGCGCCGGCCCGGGAGCCTTTCCTCTATTACAGGAACTTCAACTATGCCAAATGGAGAACCTGGAAGTGTGGAACACAACATACTCCTGTGGCCAGAGCAATGAGGATGTGCAGGAATTCATGTTCCCAAACCTTAGGGAACTGTTAATTCGTGATTGCCCCAAGTTGAGACTGAAACCATGCCCACCTAAAACTGTGGGATGGAAGATAGAGAACAGCGACAATGTACTGTCGTCATGGGATGAGGAGGGAGAGATCGACCTTGCAGCATTGTTCCCATCAAGGAAAGAGTTTAAGAAACTTTGGGAAGGGAAAACAAAAGTGCCAGCAGCTTGTAGCGCACAATTTGAAAAATGCAGAAAACCTGGAGCTTCCTACAATGCTTGTCCTGAACGCGTGGAGGTGAAATCGAGCAAGCTGCCTTTGGGAAAATGGAGGCTGTTCCGGCACCTCCTTCCCACCTATGATTTAAGGATCTCGTGCTGCACTTATGCGACAAGCGGCTCGTCACAAGAGATCATCCAAGGCCTCGCCTTCATCGAATCATTATGCCTAGAAGACGATGCTCAGTCTGAGCTTCCGAATTGGTTGGGTGAGCTCACTTCTCTTGAAACGTTGGAGATATCGAAGTACCCAGGGCTAGAGGCACCACTGGATGGCATGAAGCAACTCGCCCACCTCCGGAAGCTGTCGCTGATAAATTGTAGAAGCATGTCAGCACTACCGCAATGGTTGGGAGAACTCATCTCTCTCAAAGAACTGATCATCTCAGAGTGGCCAAACCTGAGCGATTTTCCGGAGAGTATGCAGCTCCTCACCTCCCTCAAGATGCTACGTTTGGAGCGGTGTCCCAGAATTACTGCCCTTCCAGGATGGCTGGGCGACCTCGCTTCTCTCAAAATACTAGTCATCAGCAATTGCAAGGGCATCGTGTCTCTGCCAGACAGCATACAGAAGATCACCAGGCTTGTACGAGTATAA

>B73_Zm00001eb131200

ATGGACATCGTGGTGGGTGCGCTGTCGGGCATAGTGGACGCGTTGCCGGGGAAGCTCGGCGAGCTGCTGGAGCAGGAGTATGCGCTGCTCTCCGGCGTCCGCGGGGATGTCATCTTCCTCAAGGATGAGCTATCAAGCATGCGCGCCGCCATCCACTACTGCGAGTCCCTCGACCACCACGACTCCCAGACCACTGGATGGATCAGCCTCGTCCGCGAGGTCGCCTACGACATCGAGGACTGGGTTGACCTCTTCAGCATCCGCGTCCACGGCGGTGCCCAATCCACTTCCGGGTTTCGTGCCTGGCTTAGCCGCAGCGTGGACAAGTTAACGGCGCTCCCCGCTCGCCACACCATCGCGAGCGAGCTCCAGGGACTCAAGGAGCGCGTTCTTGAGATTAGCCGGCAGCGGAACCGCTACAGGCTGGGCCAAATGGTTGGCACCACCTCGCAACATCCCCATGATCCCAGACTTTCTGCGCTCTTCGTCGACCCCGGCCGCCTCGTCGGCTTCGATGGGAAGGTGGAGGACGTGTCCAAGACTGTCATGGACGCCGGAGGTAGTAATGGGCTGAGAATCGTCTCCATTGTCGGGATGGCCGGCTCAGGGAAGACGACGCTCGCAAATGCTGTGTACCGGCGTCTCCAAGCAGACAACACCTTCCAGTGCTCTGCTTTCGTCTCCATCGGACCGAAGCCGGACATGGTGAAGACAGTCAAGGATATGCTCTCAAGACTCGGCGACGGCCACCGAGGAGGCGAGGACATTAGCCAACTCATCCCGAGGGTCAGAGGAATACTGGAGAAGAAAAGGTACCTCGCTTGGATCGATGATATATGGAGTAGTGAACAATGGGGAGTAATAAGGTGTTGTTTTCCAGACAACAGTCTTGGTAGTAGGATAATCACCACATCAAGGAATGATGCGTTGCCCACCAATCATCATTACGGTTCAAGCAAATTTGTCTACAAGATCGGCCTCCTTACTGACAATGAAGCCAGGGAGTTGTTTCTGAAGAAAGCTTTCAGCAGCCGGAATGACTGTCCACAACATCTGGTGGATGCTTTTACCAAGGTTCTGAGAAGGTGTGCTGGCTTGCCACTTGCTGTGGTTAGCGTAGCTGCCAAGTTAGCACACAAGCAATCAAGAGAAGAATGGGAGAAGCATGGATTGAACTTGCTATACAGCTCACATTCAGATGGGTCAGATGGGCTGAAGCAAATACTCCATCTTAGCTACAGCGATCTACAGCCACAACTCAGGTCATGTTTGCTGTACCTGAGCATATTTCCTGAGAACTCAGAGGTTGAGACAGATCGCCTAGTGAGGCGATGGATTGCCGAAGGACTCATCGCTGCAAGCAATGAGGATACGGCAATCAGTTCCCTCAATGAGCTAATTGGGAGAAACTTGGTACAACCGTTGGATCTGAACCATGATAGTATCCCAAGGTGTTGCAGAGTCCACCCAGTGATATATGATTTCATTGTTTGCATGTCGATGCAAGACAACTTTGCCACTGTAACGGATGCTCAACATGTCCCAATCAACAACAAAACTGTCCGTCGGCTGTCCCTGAATTTGAAGAGCAACAGCAAGCAAGATCAGCCTGCAGCACGAAATGAGACTACTGATTTGTCTCATGCTCGCTCAGTCACTGTCTTCGGTCACGCTAGTGCCACCCCTCATCTGACTGATCTGAAAGTGGTGCGTGTGCTGGATCTCGAAGGCTGCAAAGGTCCTGTGTGCTTGGATGGCCTATGCAAGCTGGTGCTGCTGAGGTACCTGAGCCTCAGAGGCACTGATGTCAGTGAACTGCCAGCGGCAATTGGGGATCTAAGGTGCTTGGAGACACTTGATGTGAGGTCAACGAAGGTGGAAGAGCTTCCTCCCAGTATTGTAAGGCTGCAAAAGCTAATGCATTTGCTTGCTGGGAGTGCCAAGCTGCCTGACGGGATGGATAAGATGAAAGCCCTGCGGTCATTGTCATGCGCTGCTACCACAAAGAGCTCTGCCAACGTCGTGGAAGAGCTCAGCAAGCACCATAACTTGAGACAACTGGAACTGTATTACTATGCTACTGAAACGCCTGGGAATGAGAAGCAAATCAAGTTCCCCGCTGATGGGCTCCAGACTGTGAAACAACTGTGCATCCGGTGCACATCACCATCGGTGACATTTGAGCCCCGTGTACTGCCAAAGGTCCAAGCACTTGTCCTGAGATTCGAGAAAGGCCGTGCTGATGACACGATTGGCGTGTCTGGCTTAGAGCATCTGTCGAGCCTCAAGCATTTGGTACTTGAGTTTGAGCAGCATGATGCGGGTGCCATGGCGACAGTTACTGCAGTGAGGATGGCTGCTGAGGGGCTCCATCTAGATCATCAATATATAACTCTAAAGGTGGATGGGAAAAAGTACTGA

>B73_Zm00001eb134970

ATGGAGTTGGACCGGCTGCTGCTCGACCAGCTGGCTGGCGAGGCCCTGCGGGAGCTGCTGCACGCCGTCCAGGGCACCCTGTTCTGCCGCTCCACTGCCGAGCGCCTGCGCCGGAGCGTCGAGCCGCTGTTGCCGCTCGTGCAGGGCCTCGGCCCGCACGCCCAGCGCTCCGCGGGGGACCTCGGCGAGCTCGCGGCGCGGGTCAGGGAGGCGCTCGACCTGGCCCGCCGCGCCGCCACGTCCCCGCGCTGGAACGTCTACCGCTCCGCGCAGCTGTCGCGCCGGATGGAGGCGGCCGACCGCGGCATCGCGCGCTGGCTGGAGCGCCACGCCCCCGCGCACGTCATCGGCAACGTGCGCGGTCTCCGCGACGAGTCCCACGCGCGCATCGCCCGCCTCGAGCGCCGCGTTGACGAGATCGCCGCCAGCGCCGCGCAGCCGCCGCCCCCAGCCCTCTCCGTCCCCGTCGCGCCGCACAAGGGCGTGACCATGCCGATGGAGGTGCCAACTCACAAGGGCATGGCTATGCCGATGCCGATGCCGGTTCCTGTGCAGGCGGTGCCCGCCAAAGCCGGGGTGGTGGCCATGGACATGGACCTCACCGAGGGACACGAAAACGAGGGGATGGTTGGCGCCGGCGTTAAGGTGGCCAAGGAAAAGGTGAAGGAGATGGTTATGAGCGGCGGCGGCGGCGGCTGGGAGGTGGTCGGTATCTCCGGCATGGGCGGCAGCGGCAAGACCACGCTCGCCATGGAGATCTTCAGGGATCATAAGGTCCGAGCCTACTTCAATGATAGGATCTTCTTTGAGACGATCTCACAATCCGCAAACTTGGAGGCCATCAAGATGAAGCTGTGGGAGCAGATCAGCGGCAACATGGTGCTCGGTGCATACAACCAGATCCCAGAATGGCAGCTCAAGTTAGGACCAAGAGACCGAGGGCCTGTCCTTGTGATCCTTGACGACGTTTGGTCTCTCCCACAGCTCGAGGAGCTCACCTTCAGGTTCCCTGGGTGCAAGACTCTAGTTGTATCGAGGTTCAAGTTCCCCACACTGGTAAAACAGACATACGAAATGCAGTTGCTAGACGAGGCGGCGGCCTTGTCCGTCTTCTGCCGTGCCGCTTTCGATCAGGAGTGTGTTCCGCGGACTGCTGACAAGAGATTGGTCAGGCAGGTCTCTGCAGAGTGCAGGGGCCTTCCACTGGCTCTGAAGGTTATTGGTGCGTCATTGCGCGACCAGCCTCCTAAGATCTGGCTCAGCGCCAAGAACCGGCTGTCTCGAGGAGAGGCTATTTCCGACTCCCATGAGACCAAGCTTCTAGAGAGGATGGCGGCAAGTGTCGAGTGCTTGTCGGAGAAGGTCAGAGACTGCTTCCTTGATCTGGGATGCTTCCCGGAGGACAAGAAGATCCCCCTTGATGTCTTGATCAACATCTGGATGGAGGTTCATGATCTTGATGAACCAGATGCTTTCGCCATCTTGGTTGAGCTTTCGAACAAGAACCTTCTTACCCTCGTTAACGATGCACAGAACAAGGCTGGAGATTTGTACAGCAGCTACCATGACTACTCGGTGACACAGCATGATGTGCTGAGAGATCTTGCTCTTCACATGAGTGGGCGTGACCCTCTGAACAAGCGTAGGCGGTTGGTGATGCCGAGAAGAGAAGAGACACTTCCAAAGGACTGGCAGAGGAATAAGGATACCCCGTTTGAAGCTCAGATAGTTTCCATTCATACAGGTGAAATGAAGGGATCTGACTGGTTCCAGATGAACTTCCCCAAGGCAGAAGTGCTCATCCTCAACTTCGCCTCAAGCCTGTACTACCTCCCGCCGTTCATCGCGTCGATGCAGAACCTGAAAGCCCTGGTGCTGATCAACTACGGCACCAGCAGCGCGGCCCTTGACAACCTATCCGCCTTCACCACGCTGAACGGCCTGAGGAGTCTCTGGCTGGAGAAGATCAGGCTCCCGCCGCTGCCGAAGACCACCATCCCGCTGAAGAACCTGCACAAGATCTCGCTCGTCCTCTGCGAGCTGAACAGCAGCCTGAGAGGGTCGACGATGGACCTGTCCATGACATTCCCGCGCCTCTCCAACCTCACGATCGACCACTGCATAGACCTAAAGGAGCTGCCAGCAAGCATCTGCGAGATCGGCTCCCTGGAGACCGTCTCCATCTCCAACTGCCACGACCTCACCGAGCTGCCATACGAGCTGGGCAAGCTGCACTGCCTCAGCATCCTCCGGGTGTACGCCTGCCCGGCGCTGTGGCGGCTCCCGGCGTCGGTGTGCAGCCTGAAGAGGCTCAAGTACCTCGACATATCCCAGTGCATCAACCTGACGGACCTCCCGGAGGAGCTCGGACACCTGACGAGCCTCGAGAAGATCGACATGCGGGAGTGCTCGCGCCTGAGGAGCCTCCCGAGGTCGTCGTCCTCCCTCAAGTCCCTCGGCCACGTCGTGTGCGACGAGGAGACGGCGCTGCTGTGGCGGGAGGCCGAGCAGGTCATCCCTGACCTCCGGGTGCAGGTGGCCGAAGAGTGCTACAACCTGGACTGGCTAGCGGACTGA

>B73_Zm00001eb136790

ATGGAAGCTATCGTGTGCGCAACGCATGGAGCCATGGCCTCCTTGCTGTGGAAGCTAGGTACCTTGCTCTCTGACGAGCACAGGCTTCTTAGCAGCGTGAAGGTGGATATGGTGTCCCTGAAAGCCGAGGTCGAGGGCATGCATGCTTTCCTGAAGAAGATGTCGGAGGTGGAGGACCCCGACGAGCAGTCCAAGTGCTGGATGCAGGAGGTTCGGGAGCTGTCCTACGACATCGAGGACAACATCGACAGCTTCATGCTCACTCTCGGCTCTTGTACTGAGTCCAGCAGCAAACCTAGAGGCTTCAAGGGGTGTGTTGCTAGGTTCTTGAACTTGTTCGCAGGTGCTAAGGCACACCATTGGGCTGCCAAGGAGATCCAACGTCTCAAACGACAGGCTGTAGAGGCTAGCGATCGGCGTGGGAGGTACAGCCACAGGGTTGGTGATGCTGTTGCTGTCCCCAGATCGAGTAGAACAAGCATAGACCCTCGCTTGCCGGCGCTGTACGCTGAGACGACAAGGCTCGTTGGCGTTGATGGCCCAAGGAACAGACTTATCAAGCTGCTGACGACAGAAAGAGAGGGCACAGTGACAGAGCTGAATGTATTCTCCATAGTTGGGTCTGGTGGGCTTGGGAAGACCACTCTTGCCAGTGAAGTGTACCGGAGGCTCGAAGCGCAATTTGATTACAGAGCTTTCGTATCAGTGTCACAGAACCCTGATATGAAGAAGATATTGAGGCATATACTCTGCCACCGAGGGTGTGGCGGCAGCGAGGAATGGGATGAGCAGCAACTCATCCACGCCGTAAGAGAGTTCCTCCAGGATAAGAGGTACTTTGTTGTCATTGACGATATATGGAGCACATCAGCATGGAGGATTATCAGATGTGCTTTTCCTGAAAACAACTGTTCCAGTAGGATACTGACAACTACTCGCATCGTCACAGTTGCTAAGTACTGTTGCTCTCCTCACCTTGACCGTGTGTATGCACTGCAGCCTCTCGATGCAGCTCACTCTGAGAGCTTATTTTTAAGTAGGATTTTTGGTTCCGAAGGTAGATGTCCTCTCCATCTGAAAGAAGCTTCCGACAAAATACTGAAGAGATGTGGTGGCCTACCACTGGCAATCGTCACAGTCGCTAGCCTATTGGCCACTAAGGGTATCACAAAAGAGGAATGGGAGAAGACGCTCAAGTCCATTGGTTCAGCACTTGAGATTCAAGATACAGACGTGGAAGAGATGAAGAAGATTCTTCTGATCAGCTACAACGATCTCCCCTACCACTTGAAGACATGCTTGTTGTATCTAGGCGTGTTTCCTGAAGACTATGAGATCAAGAGGGACCGGTTGATAAGAAGGTGGGTCGCTGAAGGCTTTATCACCGCAGAAGGTGGACAAGACATGGAGGAGATAGGAGAATGCTATTTCAACGACCTTATCAACAGGAGCATGATCCAGCCTGTTGGCATTCAGTGTGATGGTCGCGCCGACGCTTGCCGTGTCCATGACATGGTTCTTGATCTCATCGTATCCAAATCAGCCGAAGAAAACTTTCTAACCTTGTGTGGTGACGGGGATCACAGGATGGCGCGACGAGATAAGGCTCGTCGGCTATCCATCAACTACCATGCCCGGGATGGTATTGTAGTGCCAGAGAATATGGTCGTTTCTAATGTTCGATCCCTCACAATCTTTGGGCACGCTGAGGACATGTCTGCTCTTTCCAACTTCCTGTTCTTGAGGGTTCTAGATCTAGAAAACAGGGTGGTGCTGGAATACAATTACCTTAGGCACATAGGCAGGCTACGTCAGCTGAGGTATCTCCGACTCAGTTCAAGAAGAATAGCTGCGCTCCCTGAACAGATAGGAGACCTACAGAACTTGCAGACCTTGGATCTGAGGTGGACAAGGCTGAAAAGATTGCCACGGAGCGTTGTTCTGCTACGCCGACTGACATGCTTGCTGGTCAACAGTTTAGAATTGCCCGAAGGGATTGGGAACATGCAAGCTCTGCAGGAATTGTCAGAGATCGAAATCAATCGTCACACACCAGTGTCTTCCTTGCTGGAACTGGGCAAGCTGACGAATTTGAGAATCCTTGGGATAAACTGGTGCATCATCGACGCAGATCATGATGCAAGAAAGGTCCACGCGGACAGTTTGGTTACGTCCCTCTGCAAGTTAGGCGTGCTCAGCCTTCGGTCGATACAGATCCAGAGCTACCACACCTGTTCTCTGGACTTCTTGCAGGATTCTTGGTTCCCACCTCCTCGCCGCCTCCAGAAATTCGACATGTCCATAGATTACCATTTCCCCCGAACCCCGAAGTGGATGGTCTCGCTGGAGTACCTCAGCTACCTGGACATATACCTCACTCCGGTGGACGAGGAGTCGTTCCGAACCCTGGGGGACCTGCCGTCCCTGCTGTTCCTCTGGATATCCTCGAGAGAGGCCAGGCCTGGAGAAGGCGTCGCTGTCGGCAGCCACGGTTTCCGTTGCCTCAGGGAGTTCTACTTCACCTGCTGGGAAGTCGGGACCGGGCTGGCTTTCGAACCGGGGGCCATGCCGGCGCTCGAGAAGCTCCGGATCCCATGCAACGCGCGTGGCGCGTGCTCTTCGCATGCCGGTGTTCGGGATCTCGGAATCGGGCGCCTCTCCTCCTCCCTGAGGCACCTCCAGGTTGAGATCGTTTGCCGTGGCGCGAGGCTCCAGGAAGTGGAGGCCGTGGAGGAAGCTGTCCGGAACGCCGCCAGCGAGATTTCTGACGAGCTCTTCCTTGGCGTGAGTAGGTGGGATGAAGAAGAGATCTTGAAGGACGAGGAGCATAAGCTAGAGGAAGCGGAGGCATAA

>B73_Zm00001eb138420

ATGGCCGCCGCCGCCACAATCATGTTCGCTGGGAAGTTGGCCGGGAGCTCGGTGGCAAATGCAACCATCTCCTTTTGGATCAACAAAGCTTTCACCTGCCTGACTGACTACTGGAAGGCTGATGGCTTGGAAGATGTCAAGGGTAGGGTGCTGCAGTCAGTGAAGAAGGTCCAGGTTGTATTTGATATCGTTGACCCTGAATACATCAAGGAACAAAGCTCTGCCCTTGATCTTTGGCTGTGGCAGTTCAGGGATGCAGTTGAGGAGGCAGAGGATGTTATAGATGAGCTTCACTACGATGAGCTTAGAGAGAAGGCAAAGGATCATAAGGTCAGTGACTGGGGCTCCTCTTCTGCTAAACTGAAGCATAAGTTTGTCAAGTCCGTTAAACATGCTGGTGTCATGGGTAAGACTGTGAAAGAATTTACTCACCGTGGCACACTCAAGAGGCTGAGGAAAGTCTTGGAGGGATTAGAAAAGGCAGCCACCGAAATTGTGGCTATCCTCACAGTCACACAACATCTCAAAGACATTGGTTCAGGTAGTAAAAGGCAGGTGAATTTTGTGATCAAGGATCATGACACTGGTTCAACATTAACTGAACCTTACTTTGTTGGACGAGAAGAGGAGAAACAAAAGATTGTGCAATGGCTGATTGAGGCGCCAGTGGAAGCATCTGAAATTGTGAGGAGTACTCACCATGTTCCTATTCTCTCGTTAGTTGGCCATGGTGGAATGGGGAAGACTACATTAGCTCAATATGTATGTGAAGAGGCTGAAGTTGTCAATGATTTCAAGGTTATATGGGTCCATGTTTCTACTAGATTTAGTGCAACTTCCGTGACAAGTAAACTGCTGGAATCCGTCACAGGGGTAAAACCTTGTGCAGATCATTTAGAGACGCTTCAGCAGATGCTCAAACAAGAACTCAGGTCTGTAAAGTTTTTCCTTATTTTGGATGATGTCTGGGAAGATGAGAATAAAAAGGAATGGGAAAATGTATTCGCTCCACTAAGGAAAGCAAAGAGTGGGAGCAAAATTTTAGTAACAACCAGAATGCAGTCAGTAGCAGATATGGCTGCAAATGCCATGGGGGTTGAAAGAGAATACCTGGAATTAGAAGGGCTGCAAGAAGATGAAAATCTTAAATTATTCAATCATCATGTATATTCTGGTAGGAATCCACAAGATTTTGAAAATTTAAAACCCATAGGTGAACATCTTGTAAAACAACTTGGAGGATGTCCCTTGGTAACAAAGGTTGTCAGCGGTTATTTGCAGTGCAATATGGATCCTGACAGCTGGACCGATTTCTTACAAGAAGGCCTTGTACATTTTAATGGAAGTGAAGATTATGTAATGGAAACTTTGAGATTAAGCTATTATTGCCTACCGGCACAGGTCCAGATTTGCTTTCGATATTGCAGCATATTTCCGCAGAACTATGAATTTAAAAAGAAAGATTTAGTGTTGATGTGGATGGGTTCAGGATTGATATCACAACATGGAAACAAACCAAGAAGGATTGAGAATATTGGGTATCAGATCTTGGCTGAGTTAACTAGGAAGTCATTCTTTGAAATGAAATTCAAGGTACTTCAGTATAGTCAGAGAAGAGAAGAATATTACGTCATGCATGACCTGATGCATGAACTGGCACAATATGTTTCTGCTGGCGAATGCTCAACATTAATTGATCCCGTCATGTTGGAAAATGAGAGTGAAAATATTCGACACTTGCGTATTGCTTGTGTTGACAAGTTTTCTGCTGAAGAGGTCAAGAAAATCACACGTTTTAAGAATCTGCGCACTGCTATTATTGATGGTCCAGGTTTGATTGACAACGATATGGCAGTCATGGTTGAGAATGTTATACAAAAATCAAAATCCTTGCGTCTACTGCGATCAAATCTGGAGAACACATTCCATCTTCCTAAACTTGCTGATTTAAAGCATCTTCGTTATGTCTATCTGCATAGGATATCACTTGAGGGAATGCGTGGGCTTGTTAAACTTCATCACTTACAGCTAGTTGATTGTTTGAATGATTGTGGGGAGGAACTAAGGCAAGTGATGTGTTTGGGGAACATTGATCATCTGCGATATGTAAATTATGGGTCGCGTAGAATTGGTGAGTTTCCAATTGGTAGACTCACTTCACTTCAGGAGCTGCACAACTATCGGGTACAGGGAGGTGAAGGTAACAAAATAAGTGATATCAAGAACCTGGGTGCTCTTCGCGAACTAGATGTTTTCAGCATTGAGAATGTTGAAAGTCTTGAAGAAGCTGATAATGCCAAGTTAAAAGAAAAACCATATCTCAACTCTCTATCTCTCATGTGGTCAGCACGTGCTGATGCGAAAAATGGGAAAGATGATTTGATTCTTGATCATCTTGAGCCACATGCCCACATTCGAAACCTGAATATTTCTGGTTATTGTGGTGCAAGGCCTCCTATTTGGATTGAAAATCTCCATGTGAAGAACCTGGTGTCACTTGAGTTAGCAAGATGCATGTATTGGGAACAGCTGCCTTCACTCGGAGAATTAGAATGTCTTAAGAAACTTTTGTTGGAGTGCCTTCCTAGCCTACAACAGATTGGTCAACCATCTCAACTTTCCAACATTAGCTGTATTGGTTCATACCTTCCTCCACATCTTGACACATTGATTATAAGACATTGCAAAGAACTGAAGCAGATCCCTATCCTACCACCCTGCTTGGTTCATTTGGAAATATGTAGGGTTGGGTTGACCGAATTCCCAAGTATAGGCAATATACATGGTGAGAGTATCGAAAGCAGGCCATCTAAAATGCAGTTTGTCAGTGTTGAAGAATGTGAAAGTCTGACCTTACCTAAAGGAAGCCCTCTGTTGCAAATACACTACATCAGAACAATCCATGTCCTACACATTAGTGACTGTAAAGAACTGGAATCTGCTCCCCTGTTCGATGAAATGAGAAACCTTAGAGAGCTCAGCATCATAAATTGCCCTAAGCTGAGGGCATCAAGCGAAACTGAAGGCAAGAACCTGTCACCATCACTAAAGAATCTTATAATCAAGCAGTGTGGTGATCTAGTGCATTTTCTTATTAAGTCACTACATGGTCTCGTTAACCTTTCAGAGCTGGTACTAGAAAATTGCCCCGGCCTTCTATCCCTTCCGTCAGCTGATGTGTTCAAGAGTCTCAAGTCGCTGAAGTTCTTGGAGGTAATTGGGTGTGAGAATCTTTCGTCATTTGGTGGACTCAGTTCCCTTTGTTCCCTTGTTACACTGAAGATTAGCTCCTGCAGTAAGCTTGCAGCTCCACCTGTACTGGGTGGTGCTGCGTCTGGTCCTGCTAATTATGATGATGATGTTATAGAAGAAGAGAATATGGTGTTGCCTGTTAATTCCTTGCAGATCGACTATCTTGAAGTTGATCTCCCATGTGTATTGAACATTGAACCTCTCAGTAGACTTTGCCACACCAAAGGATTGGTAATTGGAGGCGGGACACAGATGGTGAGCTTGCCAGAGCAATGGCTTCTACAGAACCACAAAGAACTGCAGTCACTAAAGGTGTTATGTGCCAGTTCATTGGAGTCTCTGCCACCAAGTATGCGAGCCCTGAGAGCGCTCAACTTCTTCTTGTTGTCAGGAGCTGGGAAACTTACATCACTTCCAGACATGCCCTCCTCCCTACAATGGCTCCATGTCATAGGCTGCTGTCCAGAGCTGGTGACCCAGATTAGAGTGAAGGACAACCCTGAATGGAGAAAGATCTCCACAGTTCCCAAGGTGCACATAGCTGCTGCCAAGACTGTCCACTCTTCTCCTCATACTTTTCATGGTGGTTATTGCTTCATGTATGGAAAAGAGTGCAGTGAAGAGACAATATATGAAGTAACCAATAACCATTGA

>B73_Zm00001eb164570

ATGACACCCTCAAGCTCAGGTTGGAAGCCATCGAAGCGGTCATCGTTGATGCTGAGAAGCAGGGGCCTCGCCGCGGTGTGCAGGAATGGCTCGAGCAGGTCAAGACGGCAGCGTACAAGGCACACGAGGTCTTTGATGACTTCGAGTACGAGGTGCTCCGCCGCCGGGCCAAGGAGAATGGGCACATCACCGAGCTTGGCGTTATGGACAGGGTCTAGACATGATGAGAGGAACAAAATGGTTAACATATTGGTCAATGCCAATGGTCGTGTTGTCGGTGGTGATCTTATGGTCGTTCCCATTGTTGGAATGGGAGGACTAGGCAAGACCACCCTCGCTCAGCTCATCCACAACGACCCTCAGGTCAAGGAGTACTTCCATCTACGAAAATGGGTATGTGTGTCAGATGATTTTAGTGTCCTTAATCTTGCCAACAAGATATGTAATGCCTCAGAGAGAGACCTCGAGGAAGCAGTGAAGAAGCTTCAAGAACATCTTAACGGAAAGAGGTACCTTCTTGTATTGGATGACGTCTGGGAAAAGGATATTAACTTTGACAAGTGGAGAAAGATCAAGGCGTTTCTTACACAAGATGTCTATGGCGCCGTATTAGTGACAACACGTGAAAAACAAGTAGCCGAGTTTATGGGTGCTGTAGTTGACAGCTCATGGACAAATCATTACCATGAAGTGGCAATCTTGGGCAAGGAATACATACAAGAAATTATAGAAACAAGAGCATTCGGATTGCAGAGGAGCAAGCCAGATGATTTAGTTGAGTTGGTTGATCGAATCGTTGAGAGATGTGTGGGGTCTCCATTGGCAGCAAAAGCAATTGGGTCTGTACTGCGTGACAAGACCACTAAGGAAGAATGGGAGGCTGTGTTACAACCAAGCACAATATGCGACGATAAGACTGGAATTATGCCTATACTCAAGCTTAGTTATAATGACCTACCAATTGACATGCAGCAATGTTTTGCCTTTTGTGCTCTGTATCCAAAGGATTATCAGATTGATATGGACAAGCTTATCCAACTATGGATGGCCAATGGTTTTATCTCAGATCAAAAAAAGGTACCTGCTGAAACCGTAGGTAAAAGGATTGTCAATGAGCTGGTCTCGAGGTCATTTTTCCAGTATGAGGAGCGAAGTTGGATGGGGTACAATTCTACAACTTTTTTGAAGATTCATGACCTCATGCATGATCTTGCACTGTCTGTTTCGGAAAAGGAATGTGTCTGTATAACAAGGGAATTGATTAAAAATAGTGAGTTGCTTCCAAGTGCTGCCCGCCACATACTTATTCAAAATTGGATAGGTAAAGAGATTCATGGTTATTTATATGGTTCTATGAGGAAAATGTCTCGGCCTATCCAAACATTTATGTTTGATGGGTCTAGTGAAGCTGCCGGTGTGCAACATTTATCAAGATATAGTTCTTTGCGAGTACTTTCTGTGCCAGGAATTGGGTTTCATTTAACAATAAAACCAAAGCATCAGTGCCACCTTAGGTTTCTTGATATCAAGGACAGTGTCATCAAAGAACTTCCAGATGACATTAGCGTCCTTTATAATCTTCAGACACTTTTCGTCATCTGTACACAGATGGGTGCACAAGGTTGGAGGGCATGCCTCCAGAGCTTGGACAAATCGCCTCACTTCGAACAATTACATGGTTTGTAG

>B73_Zm00001eb164870

ATGACAGGTGTGGAGCCAGCAATTATCGGTGCAATCGCTAACTTGGCGGCCCCGGTCCTGCCTATAGCCATTAAAGGGATACAAGGTGCACTGAAGAAACGGCAAGTCCGTGACAGTGATGTTGAAACCCTGAAATCCCAGCTTAGCTACATCCAGGGCATCATCCGTGATACTCGGAAGACTATCAGGAGTTCCCAAGACCCGTCCGACAGGCTTCAATCCTGGGCTGGATACCTCAGATGCTTGGCGTACGACATCGAAGACCTAATAGAAGGCCGCCGTGCCGGAACCATGACAGGTGCGAAGCTTAATGGCAAGATTACTATCATCCAGGATTTAATCAGATGTGTACAGTCCTATCCGGAGTTTATGGCGGTTCCGACGAATGAGGCTCCTAGTCAAGGCGCTGCTTCTTCCTCCACTACTTCAAGCACCCAGGGCTTTCCGCTGGCTGATCTTGTGGGCAAGAAGGAGGACCTCGATGAGCTTCTGGACCTCCTCGTCCGGAGACCCGACAATGAGCTGGACAAGGTCCTCAAGGTGATGGTGATCTCCGTCGTCGGCTTCGGTGGCATAGGGGAGACCAAGCTTTGCCACACAGTGTACACGGACGTACAGGAGAGCAGAAGGTTCTCCCTGCATGCGTATGTCAGCGCTGCTGGGAAGGACTGCAGCATCGTTCTGGAAGAGATAATCGAGCAATTTAGACTGCAAGAGGATCCACAAGATAGCAGTGGTGGATTTTTTCACAGATTCGCCGGAGCGTTTCCTGGGGCTCGTCGTACTGACCAAGTTCATGGGTTACCCGAGTATCTCCAAAGGAAAAGGTATTTTGTGGTGGTGGATGGCGTGGAGTCTGAAGAACTGGTGAGTGGCATAGCATCTGCCTTCCCGGATAATAGTATGGGCAGTAGAATTATCATGGGTATGAGGACCGCAGTGGGCAGGGATGCAGAGAGATGTGTGGGTCATCATCACAAGATGTGGCCACTTGAAGACAAGCAGTCGGTGGTGTGCTTCCTAAATGAAGCGGAGCGGCGGCGACGACGACATGAACAAGACGACCACTCGTCGTTCATTAGGTTTCAGGAACAAGACCACTCATCATCCTGTTTGCACAAGGTATGTGATGGCGTACCACTTGCGCTGGTTAGCGTATGTGAAGTCCGCAGAGGGTCCATCATCACTGCTGCCGTTGAAGAACAAGACCGCTGGCCACACAGAATGCCCAAGGTGCTCGACCACAGCTACGATGGTCTGCATATCCGGGGTGCTGGGAGCTGTCCGAACCAATACATCCCCTATCTCCAAGCCTGCCTGCTGTACTTTGCCATGTTCCCCCGCGGCAATCATGTCAAGAGGGGATCCCTGATCAGGCGATGGCAGGCGGAAGGCCTAGAGTTCGGAGGCAGCAATCAAGCTGCCGAAAACCTCAAGGCCCTCGTAGACCGGAACTTCGTTTGGCCCCTCCATGCGAGCCTGAATGAGCACGCCAAGACATTCCAGCCTCCTGGAGTGGTGCTCAACTACATCTCCCGCAGGTCTCAAGAGGAGGAATTCATCCTCAGGTCTTGTCCAAGTGGGGAACTTAATCCCAATTACAGCCGCCGGCTTTGTCTACATCCTGCCGAGGAAGAAGAAGGTGAACCCCAACCCGTGGTCATTACCAACGGTTCTGTACCACCACGCCTGCGAACTCTGGCTGTGTCCTGGGGGCAGCAACAGGCCAGCAGGATTGCTGAATGCGAGCAGCTCCGAGTGCTGGATCTGGCGTCATACAATGGTCTACAGCCAGACCAGCTAGAGGAGATATGCAAGAAACTGAAGCTTCTCAAATATCTGAGCCTCCTGCCAGATATCATCACTCAAGTTCCAAGCTCAATGTCTAATTTGCAGTGCTTGGAGACACTCGAGGTGGGGGAGGTCAATGGCAGGGCGGCAGTTCTGGTGCCTATCCAAGTCTTGGAACTGCCACGCATAAAACACCTAATCGGAAAATTTGAGCTTATTGACAACTTCAACGGACTACCAATTAGGGCTTATCCAGATGCACTAGTACCAAAGGCAATCAAGGAAAGCAACCTGGAGACGGTGTCGGGGTTCTTCACCCGCAGAGGCCAAGGATTTCCGCCACTCATGCGTCACATTAGGCAGCTCAGGAAGGTGAAGATATGGTTCTACAGGGATGCAGAACCCAAATACCTAGCAAGCTATCTCCCGAAAGCGATTACAAAATTCCTCAGGAATGACAACGTTCATCGCTCCCTGTCACTTGACTTCCAGGATGGCCCAAGACAAACAGAAATACTGCAGGCTTGTGTGGCTGAAGCTAGCGGTAATCTTTACTCCCTGAAGCTGTCGTCCGGCACAAATCTGAGCAGGATCCAACTGTCGGTTATTGCCACCAACAAGCTGACTGGAATCACAAAGCTATGCCTTTCCCGCTGGAAAACGATAACGTTGGATGCAGAATTTCTGAATGAGCTGACCAAATTGGCCAGTCTGACGTATCTGAAGCTGGATGCAGAAACAATAAAAGGTACGTACGACCAAAACCCAACACCAGGAGGAAATAACCAAGAGAAGGTCGTCATAGAGACTGGGCACATTGCAAATCTGCGGCGGATGTGCCTTGTGGCCAGGCAGACGCTGCCCGACATACAAGTCAAGCCTACAGCTCTGCAACGACTCGTTTCACTTCATCTCATCAGTGAAACGGACGATTATTGTCCTTCCGCCAACGTCATCTGTAAAGCCAACCCCCAGGACGACAACGAGCCGGCGCCGTTCACGAGCCTCCAGGAAGTCTCGCTGAATGCTACGGTACCCGAAAATTTAAGGGATTCTTGGCGTAATGCTGCAAGGGACCATCCAAAGAGGCCACGAATTCTCTTCATCCAACACCCTCACCGTGCGGGATAA

>B73_Zm00001eb164940

ATGGAATTAGGTGCTTCTGTTTCCATGTCTATCCTGGAAAAGCTCGTCCAGAGCTGTTCCTTTCCAGATTCATGGCGGGGGCCGCCAGACGCTGAGGTCCAGGCTCTCATAGGATATCTCGAAGAGCTACGGTCCTCCGTCCTGGATCTTTCCAAGGAGGATGAGGACGAGGATGACTCTGCTACGTCGTCGTCCACGCTGAAGATGAGCCTGACGAGCCAGTTGCAGGAACTTTGTTATGATGCTGAGGACTATCTGGAGATGGCGCAGCACTCCCGTGGTGGCTGCTCCTGGCAGATCAGTTGGGTCCGGAGCAAGGCGACGAGGCAGCGCCCCGCTCTAATCAGCGCAAAAGATCTCTCCGGCCTCATCTCCCGTGTGAATCCGGCGAAAGAAATAGCCCAAGCGTACATCAAGTCTGCTAGTTCAAGAACCACCACCAAAGAGAATGGCCCTAGGCCACAAGAAGAGCCTGCAAAAAGCAGTAGTCGCCGTGCCGTCTATTCTGATGATACTCTACAGCCTGACAGCCATGACGAGCAGCTAGTCAGGTTGCTGGCTTTGGAAAGTGATCAGCAGCTCAAGACGGTGGCAATCCTTGGCCTGCCTGGCGTTGGGAAGACAACACTTGCCAGAAGACTGTACCACTGCTATGAAGGGAGGTTCCATTGCGGGGCTTTCCTTCGGGCGTCCCGTAACCTGCAGGATACCACCAGGCTTCTCGCCACCATGCTATCCAAGATTAAGGGCCAACAAGGGTGCCGCTACTGGGGGGGCTCCGGTGATGAGCAAGATCTAATCGACAGCATCAGGCAACATCTACAAGGAAAATCATATTTCATTGTGATCGATGATTTATGGGCTACATCAGTGTGGGATTTCTTGAGCCGTGCTTTTCCCAAGGATAACTGTGGCAGCCGAATAGTAATAACTACTCAAGTTACAGAAGTTGCATTTGCTTGCTGTAACAACCACACAGTTGATATATTCAATATGAAACCTCTGGACGACGATCAGTCACTGCAATTGTTTTACAGTCGAGTAAAGCATATAAATGGTTATAATGCTGAAGAATGCAAGGCCATATCACATGGAATCGTCAGCAATTGTGGTAATCTTTCACCGCTAGCTATCATAAATATAGCTGGCATGTTAGCAGGCTGGGCGGATTTCAATATGAATGACTGGGAGTACGTAAGCAAGTGTTGTACATCAACAGCAAATCTTACTACCGAGGAGGCGACGGAGAGATTTCTGAACCTTATGTACAACAAGCTTCCAGCCAAATTGAAGACGTGCCTGCTCTATCTCAGTATGTATCCAGAGGGCTGTGTCATCGGAAAGGATGATTTGGTGAGACAATGGGCAGCTGAAGGTATTTTCAGTCAAGTGGTGGAACCAAAAGGTAGAGAACATGAAGTGGGTTTTATTTATTTTGATGAGCTCCTGAAAAGAGGATTGATCCAGCCTGTAGATACAGACTACAATGATCAAGTGTTGTCATGTATAGTTCATCAAGTGGTACTGGAATTTATTACAAAGAAATCAATGCAGGAAAACTTCATCACTGTCGTGGATTATCGTGAAACAGGGACAGTGCTTGATGATAATAAGGTTCATCGACTGTCTGCCAGGTTTGAAGGTGCCAAAAGTGCACAGATACCGCGGAGCTTCAGAGTACGCCAAGTTCGGTCCTTTATGTTTTCTGGATTCATTAAATCTTTACCTTCCCTTCTCAAGTATTGCCTTGTCCGAGTTCTGATTCTTCATGTTTGGAGTGATGATCAAGGCAAGACTGTGGTTCTAGACCTTTCTCCAGTTGGTAATATGCTTCATGTAAGGTACCTGAAGGTGGTAAGCAACATGATAGTCAAACTCCCACTCATTATTCGAGGCCTGCGACACTTAGAGACACTCGAGGTGGATGCAGAAGAAGTCGCTGTTCCACTGGATGTTTTCATCTTGAAGAGCCTGTTGCATCTCCGACTTCCGAGCAAGGCTTATCAGCCTGATTCAGATATTCAAAGGATTGATTTAACAAATACATTTCCTTTAAGATTTCTGCCCTTCGTCAGAGCGTTGTCAATGCTAGGGCGGTTTCTCCTCTCCCATGGCCATCGGATTCGCCATTTGACATCACTTCAAAGTCTTGGCTACTTTGACCTAAGCACTTGCTCCAGATATACTGTGTGGCAACTTGGCAAGCTGACCAATTTGAGGGATCTTCATCTAACCTGCTCTGCAGTTCGTTCCAGGCATCGAATTAGCAACATCCAATGCCTTGCCTCTGTTCTGGGGAAATGCACCAGCCTCGAATCTCTAACTATCCGTGGCGAAGCATCTTCAAACCAGAGCATTTCTTTTGATGGCTTAAGCAGCTTGTCTTCTCCACCGTACAACCTCGTGAGCTTTGTGCTGTCCCCGAGGATTTTCAAAATGGCGAAGCTCCCAAAGTGGATTGGGCAACTCAGCAGGCTCAGTACGCTAAAGATTGCTGTTGGCGAGCTGTCAAGTGAAGATGTTGACATCCTCAAAGGACTCTCTGCCCTCACTGCTCTTTCTCTCTACGTTCGGAGAAACCCTAAGAAAGGAAGCTGGATCCTATTCAGTAAGGGCTTCGCAGTGCTCAAGTATTTTAAGTTCACTTGCACTGCACTGTGCGTGAAATTTAGTGAACAAGCTATGCCTGCTGTCCAAAGGCTGATTGTGTGCTTCAATGCTAACACAATGCAGCAGTACAGGCCGGAAGATGCAGGAATTTGGTACCTGTCAGGCCTTCAAGTTATCTCTGCCAGAATAGGAGCTGCCGGTATTGATCAAGCCAGCAGAGAAGCCGCGAAATCTAGGTTGCTGGACGCCATTCTTAGCAACCATCCAAAGCCTCCTCCTATCAGAAACGTGCAAATGGTGGACTGGGTTATTCATGGCGACACAGAGGAGTGTTCAACCGGTGTGATGATACGTAAAGATGAAAGCAGCCGTGAGCATACTAGGTCATTTTTAAGTCAGTTCCCGTTCAGAAGACGACCCCTGCTTCCCTCCATATTCACAAGGCGTGGCCAGGATGATGAGCAACATAAAATCGAAGAAAATGATGTGCTACAAGATAGTATTATCTTAAGGAAATCCTCTTCGCTAATGCAAAAACCAGGT

>B73_Zm00001eb169030

ATGGCGGAGGCCATCGTGGGGTCGATGCTGTGGAAGCTGCAGCAGGTGGCGGTGAGTGAGGCGCGGACGCTGGTGGCCGTGAACGAGGACATCCGGAGCCTCCGGGACAAGCTCATGTGGATGCAGGCGTTCCTGCACGACGTCCAGCCGTCGCGCCGCGTGCAGCCCAACGAGCTCATCAAGGTGTGGCTGCAGCAGACCCGCGACGCCGTCTTCGACGCCGAAGACGCCGTCGACCAGTACTTCGTCCAAATCGATCTCTCAAGATTCCCTAGCTGGAGCCGTGCCATCTTGGGTTTCTTTGCCAGCTTTACCACCCAAGTGGTTGTCCGTCGCGACCTCTCCAGCAGGATCAGGCTGATCAATGGAAGGCTGGAGGGAATCATTGCCAACAAGGACAGGTACAGGTTGGGAGAGTCTACTACGCTGGGAGCAATTTGGAGGCCATCTAGCTCCACGTCACCGCTCTCGGAAATGATGGACGAGGTGGTGCTGCCACTGGTGGGACGAGAGGAACTGGTGCGCAATCTTAAGAATTGGCTTTATAAAGGCAGCGGGAATCATAAAAATGTGATCACCGTGACGGGGGAAAGTGGAGTCGGCAAGACAAAGCTGGTGAGGAACTTGTACGACAGCAAGCAGACCCTGTCTCACTTTGACATCTACGAGTGGGTGAGCTTTGGGCCCAATCTCAGTGCCTCTGATGTCCTCAAGATCATCATCAGACGCATTACAGATGGAGAAGAATGTTCCAAGGACAACATAGAGAGGAAGCTGCGGGAGATACTGAAAGAAAAGAAGTATCTGTTGGTGATAGATGCTGAGCTCAGTAACTCGGAGTGGAATCGCATTTTCGCTATGCTCCCTGACCCCAAGGATGTGGATGTCGATGCTGCCAGCAGAATAGTGAGGATTTCTCAGATTCCGCCACATAAGCCACCCCCACACTATCAAGAAGCCAAACTTGAGGTTCCGAAGTTTTATGACCAAGAAGTAGTCATCAATCTGTTCAAGGAAACCTTCCAATCATGTGGCAGAAATGAACTTCCTGACGAAGCAATAAGAGAATACAGACAGAGAATCTTGGACAACACAAAAGGGTTGCCGCTGGCAATAGTTCTTCTGTCAGGCCTTCTGCGGACCAAGGAGTACCCTAGTGAATGGGAGACGGTGTTCGAGCACCTGGACAGGATGCAGTCAAAGCAGATCGACAAGATTCTATCCCTCTGCTTCGATGACCTTCACTATGACCTGAAATCGTGCCTCCTCTACTTTGCTGCATTGCCAGTGAATACCTTTATCAGGGCAAGCAACATCGTGTGCATGTGGATGGCGGAGGGCTTCCTGGTACCCAAGGCCACAACGGTGGAAAAAGTAGGAGAGCAGTACCTGATGGAGCTGATAGATAGGCGCCTCGTCAACTTGGCGCCAGTGGGCTACAATGTTCCTGGGTACGAGCGTGTAGCTGTTCAGAGCAAAGTACACGCTTTCCTGCAGCTCGAAGCACAGGAGCAATGCTTCGTGGAGATCCACAGTGGTGACGACATCCCGGCTTTGTCGGATGCACGACGCTTGTCACTCCAGAACCACAAAGACAAGTATGCAGCACTGTCACACCCGCTGCCGAAGCTGCGAGCCATCCTGTCAAACTTTGAGACGGAGCAAGAGGCTGCACAAGTTGCTGGCGAGAAGCCACCAGATGAAGCTGGAGAAGGGCAACATGGAAATGGATGCCGGCCTGGTTTCAACAAAAAGAAAATGCAAGCCAAGTCTTGTGTGCAGGACCTGCTACGGAACTCAAGGTTCCTCCGTGTCATCCATTTCAACGGCCTCGAGGTGGACAAAGAGCTTCCTGACGAAATCGGCAAAGTTGTGAAACCTGCAGTACGTCCGAGTGACCTCCTGCTCCTTGGAGAAGATGCCGCCGTCGATTGGCAGGCTGTGCAACCTCCAGACTCTTGA

>B73_Zm00001eb174770

ATGGAGCTCGCTGAGGCGGCAGTGAAGTGTCTCCTCGTCAAGCTCGTTTCCCTTTTGAAGAAAGAGTACCATCTTCAGAAGGGCGTGAGAGGGCAGATTGTGTTCCTCACGAAGGAGCTGAAGGGTATGCAGGATGTCCTCGAGGAGTTGTCCGAGAAGCCAGCAGGTTCCGTGACCCCTTTCCAGAAGCGGTGGGCGAGGAGTTTGAAGGAGCTATCGTACGACATAGAGGACAGCGTGGACGCGTTCATGCTGCGTGTGGACGGCGGCGATGCTCACGCGAATCCGCGCAACCTGACTGGTTTCAGCAGGTTCATCGACAGGATGGGCCTCATTGGCAACATCAAGTCGGTAAAAGTGGCCAAGGTTCGTCGCCGGATCGCCAAGGAGCTCGAAGACATCACGACCCGCGTCAAAGAGGTGGCTGCATGGAAGGGAAGGCTCACCATTCCCGGCGCCACGGCACAGCCTCACACAAAGGCTAACGTCGACACCGGGATCCACCTTCTCCTCGAGGATGTGCACAAGCACAGGCACAGCCCCGTCGGCATCGACGGCCCCGCACAGAGGCTTGCCAGCTTGCTGACGACGCACAGGGAGGGGCTGCAGCAGAACCTTATGGTCGTCTCAATTGTTGGGCTAGGAGGTGTTGGCAAAACCACGCTTGCTAAGGCGTTGTATGAAAGCCTCGAGACACAATTCCAGTGCAAGGCTTTTATCCCCGTATCACTGAGGCCAGACAAAAAGAGCATCTTCAAGAGAATACTGCGTCAGGTCAGGCCGGCCGCCAGGGGTAACAACAACGACGGAGAAAAAGATGTCGACGAACTGATACGGGATATCAGGAAATACCTCAGCGACAAGAGGTACTTCATTGTTATCGATGATGTATGGGACGAGGAAATATGGAAATTGTTCGAATATGCTCTCGTTGACAATAGCTGTGGAAGCAGGGTGATTGTGACGACCCGCAATTTTCGTGTGGCAAAACTGTGCGCAACTCCTGTTGCTGGTGCATTGTTTGAACTCGAACCTCTGTCTAACGCAGACTCCAAGAGGTTGTTCTACAAAAGGGTATTTGGTAAAGATGGCGGAAATCATAATCAGTTGGATGAAATAGCTGGGAGAATTTTGAAGAAATGTGGTGGGGTACCATTAGCTATCATTACTACAGCGAGTTTGTTGGCTTCTAAACCCAAATCAATAAATGAATGGTATCGTGTGTACAGATCCCTTGGTTCAGGACTTGAGAAAGATAACACTGATATAAAGCGTATGCATGATATTTTATCTCTCAGTTACTATGAGCTTCCTTCCTATCTGAGGCCTTGTTTACTGTATCTAAGCCTTTTCCCAGAGGATTGCAGAATTGACAAAGACAGCCTTGTGAGGAGATGGGTAGCCGAAGGCCTTGTTGTGGATGAACAAGGTGGGGGGAATCTGTATGAGTTGTATGACCGTGGAGAGAAATACTTCATTGAGCTTGTTAATAGAAGCTTCATCCAGCCACTAGACATCGATGGTGTAGATGGCATTCCGAATGCTTGCTCCGTACATGATCTGATACTTGACCTTCTGATTTCTCTGTCAGTCGAGGATAATTTTGGGTTAAGGTTGGATTCTCAGAACTTCACCACCTCAACAGATAGTGAGGTTAACATTCGTCGATTGTCGCTGCAAGACAATGAGGTGGAAGTGAGTGTGCCGGAAACAGTGGACTTATCCCATGTGAGGTCAGTAATCGCATTTGGCGATGCTTTTAACTGGGCGCCACCTCTGTCAAGGTTCAAGTTCCTCCGTGCCTTGGATTTGGAGGGTTTCCCACGGAAGAACAACCATCCAAAGGAACTACGACGCCTGCACCATTTGAGGTATCTGCAGCTACGTGGCTATCTTCAGAAAGAGGTGCTAGAAGAAATTGGAAACCTACAACATCTGAAGATGTTGGACTTGAGCCATGCATATGTTTCTCAGCTGCCAGTAAGCATCACTCGGCTCAGAAACCTTCAGAGTCTACTCGTCGGATCGGATGTGAAGATGCCACAGGGGATCGGGAGTCTAAGCAAGCTGGAAGAAATGTCATGGATCGAGGTGGAACCAAACACTGCAGCGGAGCTGGGAAAGCTGACAGAAATGAGGGTGTTGTGCGTACATGGACTGGGTTACGATGAGGCTGGCGACCAGGCTTTTCTCCAGAGCCTGTCCAACCTACGCAACCTCTTGGGTCTATTCATCACTGAGTCTGAGATGTGTTCCTTAGATGACCTGCCCGATCCTGGACAGGCCCCAGTAGGCCTCCGTTTCTTTCGTGGAACCGAGACGACCTTCCAGCAGATGCCACGTTGGTTCTCTTGGCTCTCCGAGCTGTCTGGCTTAACCATCACTGTGAATAATCTCACACAGGATGACATCGATATGCTTGGAGCCTTGCCTGAGCTACGTCTTCTTCAGCTAGAAGTAGCCGAGAATGGCACCATTGCCCAAGAACAGCTGTCCATTGGTAGCGATAAGCTCTTTCAGTCATTGGAAGCATTCAAATTTAAGCACTGCGCAAGATGTTGGCTGGTGCTTTCTCAAGGAGTGATGACAAGACTTCAAAAGCTTGAGCTGTACTTCGAAGTGAGGAAGAGAGATGACGGTGCGATTGATGTCGGCTTGGAGAACCTGACTTCCCTGAAGCATGTCACTGTTGAAGTTGATTGCTATGGAGCCAACATCAGGCAGGTAGAGGATGTGGAGACCAAGCTCAGGGATGCACTGGATGCCCATCCGAAGAATCCAACTCTTGAGTTGTCACGAGTAGCTACCCACTGCATGGAACGGGATGAGCATTCTTCTGGGCGTCATCAAAGCTTCGGACGGCCGGCAGACGACGATGATTACAGCGACAATGACAGTGATGCTTCCAAATAA

>B73_Zm00001eb195760

ATGGCGGAGACGGCCATCGCGGCGGTGCTCTCCAAGTTCGGAGGGCTCGCGGCGAGCGAGGCCAAGGTGCTGCTGGAGGTGGGAGACGACATGATGCTGCTGCGGGACCGCCTCGAGTGGCTGCAGGCCTTCCTCCGCGACGCCGACCACAAGCGCCGCACCGGCGACGACCGCCTCACCCGCGTCTGGGTGCGCCAGATGCGCGACGTCGCCTTCGAGGCGGAGGACGCCGTCGACGAGTTCTTCCGCAAGGTTGACATGGAAGGCATGGGTTACCAACGCTGGATCACATGGCTCAAGAAATGCTTGCTAGGCTGCTGGGCCGAAATCATTCTCCGGCATGAGTTGTCTGGTCGCCTCGAAAAAATTAACAGCAGGCTCCATCAGATCTCGGAGAACCAAAAGGAGTACAAGGTCGAGCCTACACCATCAGCGACAGTACTCACGTCTTCCACTACAGCCACTTCAGCATGGCGGGATGGTTACAAGAACGCCGTGGGTTTTGAGAAAGAAGTGGAGACACTAAAGGAAATGCTGCTTGGTAAAGGTCGCCCTCAGCTGACGTTTATCTCCATACTTGGAGAGAGCGGTGTGGGAAAGCAAACGCTGTCACGTATTCTCTTGCATGACATGGAGAAGAACAAGCAGTTCGACGTCCGAGTCTGGTACAACATGCCGTCGGGTTCCACCACGGAAAACCTCCTCAAGCAAATCTACAAAAAAGCAGGAGGAGGAGGACAGCGGCGGCATCAGCCGTGCGAGTGCGAGTGCGACGACGACGATATCGAAGACGTGCTCCGTCGTCACCTCCTAGCCAACAAGAAGTACCTGCTGATTCTCGTTGGCATATCCTCCAAGACCATGCTCAACTGTGTCAGGGCAAGCCTACCGGACGACAACAATGGAAGCAGGGTAGTGCTCGTATTGGACATCGAGAACGAAGAGGTAGCGTGGCATGCTAATGCCATGAACAAGACGACGGGCATCAGCCACGGATTAATCCACCACTTGAACCGTCTGGACCAAGACAAGAGTGTGGAGTTGTTCTGTATGAGGGCCTTGAGGACAAATCTGTCAGATGAAACCACAGTGAACAGCATGATGAGCAAGTACAGGGAAGTTGTGTACAATATAACTTCCGGCTACCCTCTGGCTATAGTGGTTTTGGCCGGACTCCTACGGTTCAAGGAGAAGCCAGGGCAATGGAACGCGGTGCTGCAGCAGCTCAGGACGACGTCGTCTGGACCAGCAGCAGCAGATCAAGAAGCGCATCAGCAAGACGACCAAGGCGGCCATACCGAGGAGAAGACGATGAGCGCCCCTACAAGTACAACACAAGCAGCCAACAACCAGCTGTCCACAAGAACGTCGATTGAGAGGGTCTTCTGGGCAAGCTTTGAAGACCTTCCCAACGCCCTCAAGTCATGCTTCCTCTACTTGGCTGCTTTCCCCAAGGGCACCTTCCTGTCTACTGGCAGTATAGTGCGGTTGTGGATGGCCGAGGGATTCATTAGGCCACAGAAGGGCAAGACCATCGAGGAGCTGGGCCACGACTATTTCAAGGAGCTGGCCTTGAGATGCCTCGTTCAGGTTTCAGGGATGAACGAGGTTGGCGGCATCACCAATGTCATTGTTCACGGAAGGCTCCATGGGTTCCTGCACTCGGAGGCTCGTGAGGCCGGCTTCATCGACGTCCATGACATGAACGACGTCTTTGTCCCACCATCGGTGCGCCGCCTCTCTTTCATGAGCTTCCAAGACGGATATACCACATTCACTAACAGGTTCTGTAAGTTGCGCTCCTTCATATGCTGGGCCAATGAGAAAGATTCAGACAATAGTATTGGCAGCAGAGGCCGCGTGAACAATGAGGAGGAACGATGGCATGATCTCAACTTCCTGCATGGTTCAGATTTGCTTCGTGTACTCTACATATCAGGACTAAGGATCAAGGAGTTGCCGAATGAAATTGGCAACAAGATCCACTTGCGGTACTTACGTGTAAACACCGAGCACCTCAAGGAGCTCCCGGCCAGCATTGCGAGGCTGCCCAACCTGCAGACACTGGATATAAGGGACACCGAAGTCGAGGAGATCCACCCGTCCTTCTGGGAGATAAAGACGCTGCGGCATGTGATTGCCAAGAAGCTCACGCTCCCGCCATCTATCAAGGAAGAAATGGGCGAGCTGCAAACGCTGCGTGGTGTAAAGCCCAGCGAAGAAGAATGGGATCAAGATAACTGCCCACTGCTCAAGATGTCCAAACTCCGGTCGCTGGAGCTGCACGGACTCATTGGCGCCAGACACGGCGCTGCGCTGATCACTGCCCTCGGGCAAATGCATCTCCTTGGCCACTTGAAGCTCAAAGGCGACAAGATCTCTTGCTGCGTCTTCACCGGAGAACGCCTTCGATATCTTCAGACTGTAGAGCTGGATGGAACTGTGCAATGGCCGCCTGTCGCTGAGTTTAATGATCTTCGCTTCGTCCGTCCAAACCTTGTCCAGCTCAGCCTGACAAATACAAACGATGCGCCGGAAGGCATCCAACAGGAACTGAGAAATGCAGGCTTTGTCCGCTCCTACCAGCAGCTCCAACCTGTTTATCGGCTTTCATACAGACAAGGAGGCGCGCTTGCGACGAAACCGGAGCAGCAGGGTGAAGCAGGGTCCAACAAAATGGAACAGCAGCACCAAGGCGAGGAAGCTGAAGAGTGA

>B73_Zm00001eb197290

ATGGCAGAAGCTGTGGTGTTTGCTCTTGCAAAGATAAGCGTATCCTTGGCAGGGTCTGCCATTTCCGGTCTGAGGGAACATGCTTCTATAATCAAGGAACTTCCAGGGAAGGTGCGGCGAATAGAAGCACAACTGTCAATAATAAACGGCGCCCTCCAGCAGCAAGATTCAGCCTACCTCAGCGACCACGCCTACATGAAATGGATCGCCTACATCAGGACCTTGGCCTACCAGGTTGAGGATATCATGGATACATACTCACACCATGCTCATCAGCTGGAGAACAGAGGGTTCATGTGGAAGCTGACCCAAGAATACCTCGGGCCTTTCAGAAGCATCTCTGCAGAGATCACCAAGATAGAGGAGAATCTCAAGCATGCCACAGAGCTGAAACAAGCATGGCTGGAAAACTACGGCCACCATGGCCAACAGATAATGGAAGCTGAGCTTTCCCAGGACTACATCCCTGCCGAGCCCAGCTGGGACCAAGATTTTGTCGGGATGGACGGCAACACAACACTGGTGACTGAATGGCTGCGCTCTGCTAGCGATTCAGAAAGCACGTTCATGACATTGCTGGGCGCGGGAGGTCTGGGAAACACCACCCTAGCCATGGACGTGTACAAGCGCGAGAAGGACAGGTTCCGTGTCCACAGTTTCATAGCTGTGGAGAGGGACTGCACCATGGATGCCTTGATGAGGAAGATACTGTTGGAGATTGGGAGCAGCATGAAGCAGCCGCCCTCGGAAAGCGTCGACAGTATCCCCGCCAACCAACTGAAGGAAGAAGCGAGGAGAAGGATCAGCAAGCTCAGAGACGGAAGGTGCTTGATTGTCTTGGACAACGTCCGGGACCCGAGAATTTACTTTGAGATGCGTGATGTGATGAGCAATCTGCCAGGAGTTCGCATCATCTTGACGACGAGGAAGACACAGGTCGCAGCCGCTCGTGATCCCTCGTCGTCACGTTTCCTGCAACTCCAGCCGTTGGACCACATCGACGCGCTACGTCTGTTCTGCAGGAAGGCCTTTTTCAAGACGAACGACAGCAAGTGCCCCCCGAATGTCGAGGTTTTCGCTACTTCTTTAGTGAATATGTGCAAGGGCCTGCCCCTAGCGATCGTAGCTATGGGAGGCATGATGTCGTTGAAGCCACCAGTGGAGCAGATATGGAACCAGGCATGCGTTCGTCTCCAGAAAGAGCTGGAGAGGAACGCGGATCACGTGCAGGCCGTCCTGAACCTGAGCTACCATGACATGCCGGGACACCTCAGGAACTGCTTGCTGTACTGCAGCATGTTCCCCGAAGACTACCACATGTCACGGGAGAGCCTCGTGCGGCTGTGGATCGCGGAAGGCTCCGTGCTGGCAGCCAACAGCCCGACGCCGGAGACCACCGCGGAGGCATACTTCATGGAGCTGGTCCGCCGCAACATGCTGCAGGTAGTGGACAACGACGTGGTTGGCAGGGTGAGTACCTGCAAGATGCACGACATCGTGCGCAAGATGGTCCTCGTGGTTGCCAAAGAGGAGAGGTTCGCTTCTGCCACCGATTACAGCACAGTGTCGCACACCGGTAAGGACGTTCGTCACCTGGCATTGCATGGGTGGAAGGACACGAACACACCACCAGTCAAGTTCCCTCGTCTTCGACGTCTAGTGGCACTCGGAGCGAACTCATGCCTGACGAAACTGCTACCCGCGATTTTCTCTGGATCGAGCTTCCTCACTGTTCTGGTGCTGCAAGACTCCGGCATCTCCGAAGTGCCCGCGTCTATCGGGAGCTTGTTCAACCTGCGCTACATCAGCTTACGGTACACCCAGGTCAAATCCCTCCCGGAATCCGTCCAAAGGCTCGCGTACCTTGACACGCTGGACGTCAGGCAAACCAGAGTACAGAGGCTGCCACAGGGTGTCGGCAAGGCCAGGAAGCTACGCCACATCTTGGCAGACGCTTGTTGTCCCGATGGCAGCCAGCAGTCAGAATTTCGAAGCTTCACTGCGTTGGAGCCCCCAAAAGCGCTGACGAGTTTTGGAGAGCTGCAAACCCTCGAGACTGTGCAGGCTAACAAGGACATGGCGATGAAGCTGGCGAAGATGATGCAGCTGAGAAGCGTATCGATCGACAACATAAGCTCTGCCCTTTGCGGGGAGCTGTTTGCTTCCGTCTCAGAGCTGCAGTTCCTTACCAGCCTGCTTCTCTCTGCAACAGATGAGCACGAGCCGCTCAGTTTCCAGAACCTCGTGCCGAAGTCAAGCTACCTAAGCAGGCTGACTTACCTGTGCCTCCATAGGGTGCAGAGCGCAAACTCCTTGGTTCTTCCGGAGAGATGCTTTCGGGAGCTCAAGAATCTCGTCTTGGAGCGCATGCCTGATGTCAGCCAGATGAAGGTTGGAGATGGCGCCCTTCAGTGCGTCCAAGCTATCCACATCACGGCGCTGCCCAACCTAGATAAGGTACCTCAGGGCATGGAATCCCTTACCACTCTCAAGAAGCTATCTCTGCTCGATTTGCATAACGACTTCATAGTTGACTGGGAGAAGAAGGAAATGAGCCGGAAGATGCCTCTTGGTCTGGAGTTGCGCATATAG

>B73_Zm00001eb200120

ATGATCAGGAAGATCACACGTAGGTTCAGCATTTCCACAGATCGAGCCGGCTTTCAGTTCAGACCTGAGGGGGACGATGGTAGCACCAGTGAGGGAATCGTGGATATCACGAGGTGGTTTGAGGAAATCCATACAGATCAAACTGCCGCGCAGCTCAGGCCAGAGGACGAGGATGGTGACATCAGCAAGCGAATTGGGGAGATCATACGCCGATTCGAGGAGATCACTGGAGATCGAGCAGCCCTGCACCTCGGACAGGAGGATGGCGAAAGAAACACTTGGAGTGGGAGGGACTCCACGTGGGAATCAAGAGTGACTAGCCATCTTCTGGATGAATCCTGTGTTTTTGGTAGGACCAAGGAAAAAGAGCATGTTGTAAAGTTGGTGAAATCATACAGTAAGTGCCCAGGAATTCATGTTTTGCCAATTGTTGGAATGGGGGGCATTGGCAAGACAACGGTGGCACAGATGGTTTACCGCGAGGTCCAAGAAAGTTATGACCTTCTGAGTTGGGTTCATGTCCCCGAAACATTTGATTTACGCAAGCTGGCGATAGCAATCACAGAGTCTCTGTCCAGACAACCATGTACATACAACAATTTTAGCGTCGTTCATGATGTTCTGCAACAAACAGTACTAAACAAGAGAGTGTTCCTTGTTCTGGATGATCTATGGAATGAACGGCAAATTTGCTGGCAAGATTTTCTTTGTTCACTCAAGTTTGCTGATACTATGACAATCCTGGTGACTACTAGGAGCAAAGAGGTAGCACAACTTCTGCAGACCATTCCTCACTTCGAGCTTGGCTTGTTACCTGAAGATCACTGCTGGCAGTTGTTCCAATGCTATGCTTTTGGCCATAGAAACATCCATGAAGAGTCCGCTTTGGTTCAGGTAGGTAGGAAAATTATGGAGAAATGTAGTTGCTTACCATTGGCAATTAAATCCATAGGTTGCCTGTTGCGGTCCAAGATGGATATGCAGACTTGGATGGAAATATCCGATAGTGAGTTTTGGGAATATTCAGATAATAATGAAGAGATCTTGTCGGCTCTTAGACTGAGCTTTCATCGGCTGCCAGGAAGGCTGAAGCCTTGTTTTCTGCTGTGTGCTTTATATCCCAAAGGCGAGCCTTTTACCAAGGACGACATGATTCACTTATGGACTGCTCATGGTTATGTACAGCCTTCAGGATGCAAAACACTAGAGAAGGTCGCTGGTGAGTACTTTGATGAGTTGAATGAAAGATCACTGATCGAAATGGATACATACTATTTGGTCAGTCGTGAGGGTCACAATTACTTGAAAAAGTCACGTGTAAGATCACCAGTTGAAATCTCTAGTGGGGAGATCTTCGACACTGATGTTAGCTTTTATGAGCTGCACATAAGATCATTGGTTGAAAATTTTCACAAAGGAACGACAGAGTCTTCCTTGCCATTTCAATTGTTTAGGCTGCATGACATGATTTGGGACCTTGCAAAATCTCTGTCCAGCTGCTTGTTTTCTGCTGTAGCTGTTGATGAAGGTAACCTTTACATGCAAAATGAAGTTCAGCACTTATTTCTCTGGTTAGGCAGGGGCAGATCGAAACAAAATACACAGAGGGGGCACTCTGAACTTATCCCCATTTCTAAGTCTCGTGATCTATTCATCTCTTGGATAAACAATTCTCTTGGGCTTGAATCTGAGCCATTTAGATGGAGGCATAGGTCCACACCCAGGCCCAGCCATCGACCCCAGGTGTCTGATTTTGAGCTCCTAGGGCTAAGCTCAACTGCGTTGCTAGGCATTGCCTTGGAGGCCATGATCCAAGCACGACCTGATCCTGTGCAAGATCCCATGAGTTCAGCAAGACTTCTAGCATATTTGCCCGAAAACAAACATGAATACATACTTTCTACACAAAGTCGACGTTCTCAGTTGTTCAAAATAGATTATTTACGCACCTTAATTTTAAAACAATGTACATTTTACAATATTGGCATATACACATATCTGAGAGCTCTTATACTATATTCATGTAAAGATAGTGGCTGCATAGCTGCAATCCAATATCTGAAGCTCTTGCGCTATCTCAATATAAGGAATTGTGATTCATTGACTGGCAAAAATCTGAATCACTTGACACAATCAATATGTCATCTTTACAGCCTTGAGAAACTGATCGTATCCACTTGTTGGAAAGAGTTCTCCATTCAGTCGTGCCATCTTTTCAGTTTAAGATATCTCCAACTCTCCGTTCAATTCAATGACTGGTCCCAGCATCCACTTTGCCACTTCCATAACTTGGACACACTGTGCTTGCAGAACTGCCATAGCATTGCGGAGCTCCCTACAGGCATAGGAAATCTGATGAACCTGAGATGCCTTAAGCTTATTGGAATTTCAGAGATCAAGAAGTTGAATCATGATTCTTTACTATGTCAGTGTAATAACAACAAGTGCCAGTTAATGAAGGCAATATTCCCTGCCCTAATGGAGTTAGAACTTGATAGTCTGTGCGAACTACAGGACTGGTGTAAATTCCAGGATTCAGATTGCCCAAAGATGCAGAGCATCACTGTAAGAAACTGCAACAAACTTAGACGAATCCCTTACTTTGGTTCTGTCAGAAGTCTAATGATAATAAACTCAGCTCTAATTGGTCTCCAGCTTTCAGCATCTAACGAACCTTCTCAGCTGCAGACTCTTGATATCAGTTACTGTGAGAACCTGGAATCCTTGTTGGGACTGGAAAATCTTTGTTCTCTCGGGAGCTTATATATTGCCCATTGTCCTAAACTATTTGTCTTGCGTCAAGAGAAGCTTTTGTTTAGGCCTCAAAATATATTAATTGATGACTGCCCTGGACTGATCGAATGGTGTGATGAGCAAGAGCTCTACTATCATTCAAAGATGAGAAAGTTTGACCTATGA

>B73_Zm00001eb200700

ATGAAACTAATAATAATGAGCAAAGTTATACAGCAGATTGGCACAGTCTACCTTACAGATGAACTTGTGAAGAGTTGGATTGGGGAGGTCCGGAAGGTGGCTTACCGTGTTGAGGATGTAGTAGACAAATACTCATACCATCTTCTTCAACTGGAGGAAGAAGGGTTTCTGAAGAAATTTTTCGTCAAGGGTACCCATTATGCCATTGTTTTCAGTGAAATTGCTGATGAGGTAGCTGAGATAGAAGAGGAGATTCAGCAAGTTATTCAGATGAAGGATCAGTGGTTGCAGCCATCCCAGCTTGTCCCTCACCCTGAGCAACTCGCTGAAATCGAAAGACAGCGTTCCCAAGACAGTTTCCCAGAATTTGTCAAAGATGAAGATCTAGTAGGAATTGAAGAAAATAGAAAATTGCTGACTGGATGGATTTACTCAGAAGAGCAGGCTAGCATGGTGATAACAGTTTCTGGTATGGGTGGACTGGGAAAATCTACACTGGTTACAAACATTTATGAACGTGAAAAGGTCAACTTCCCGGTACATGCTTGGATTGTTGTGTCACAGGTCTACACAGTCGAGTCTTTGTTGAGAAAGCTACTATGGAAGATTGGGCATATGCAACCACCAGTGCCAAGAGAGATTGACAAAATGGATGTACATGACTTGAAGGAGGAAATAAAGAGAAAGCTCCAGAATAGAAAATGCTTGATTGTGTTGGATGATGTTTGGGAGCAAGAAGTATACTTCAAAATACATGATGCTTTCCAGACACTCCATGGAAGCCGCATCATCATTACAACACGAAAGGACCATGTTGGTGCTATTGCTTCCTTTGACCACCATCTTGAGCTCCAACCGTTGTGTGGGCCTGATGCATTTGAACTTTTCTGTAGAAGGGCTTTTCACAACAAGAAGGACCACAAATGCCCCGAGGAGCTTAAGGAAATTGCTGGTGAAATAGTGAAAAGGTGCCAAGGCCTGCCACTAGCAATTGTTACAGTCGGCAGCTTGCTGTCATCTAGACCACAAATAAACATTTGGAATCAAACATACAACCAGCTTCGGAGTGAGTTGTCAACCAATGATCATGTCCGAGCAATCTTAAATCTAAGCTACCATGATCTATCTGGAGATCTCAGAAACTGCTTCTTGTATTGCAGCTTGTTTCCTGAAGACTACCCCATGTCACGCGAAGCCCTTGTGCGGCTCTGGGTCGCAGAAGGTTTTGTTCTGAGTAAAGAAAAGAATACACCAGAGGAGGTGGCTGAGGGAAATCTCATGGAATTGATCCACCGTAATATGCTTGAAGTTGTAGACTATGATGAGCTTGGCAGGGTTAGCACTTGCAAGATGCATGATATCATGAGGGACCTGGCACTTTGTGTTGCCAAAGAAGAGAAGTTTGGTTCTGCAAACGATTATGGTGAACTGATACAGGTGGACCAGAAGGTTCGTCGCTTGTCGTTATGTGGGTGGAATGTTAAGGCAGCAGCTAAGTTTAAATTTCCATGTCTCCGTACTCTTGTGGCTCAGGGAATAATTTCATTCTCTCCTGACATGGTATCCTCAATTATGTCTCAATCAAATTATTTGACAGTTCTTGAGCTGCAAGATTCTGAGATCACTGAGGTGCCAGCATTTATAGGAAATCTCTTTAACCTACGGTATATTGGGTTAAGGCGCACCAAAGTCAAGTCACTCCCAGAGTCTATTGAGAAGCTCCTCAACCTCCACACTCTGGATATCAAACAAACTCAAATAGAGAAACTACCACGAGGGATTGTTAAGGTCAAGAAGCTAAGGCACCTTTTAGCTGACAGGTTTGCTGATGAGAAGCAGACGGAGTTCAGATATTTCATCGGAGTGGAAGCACCTAAAGGTCTGTTGAACCTGGAAGAACTACAGACTCTTGAAACAGTGCAAGCGAGCAAAGACTTGCCTGAACAGCTGAAGAAACTGATGCAACTCAGAAGCTTATGGATCGACAATGTAAGCGGTGCAGATTGTGATAACCTTTTCGCGACTCTTTCAACCATGCCACTTCTTTCCAGCCTCCTAATCTCCGCAAGAGATGTGAATGAGACACTTTGCCTCCAAGCCCTTGCTCCGGAATTTCCAAAGCTCCACAGGCTAATTGTAAGGGGCCGCTGGGCTGCCGAGACACTGGAATATCCAATATTTTGCAACCATGGGAAACATCTAAAATATTTAGCGCTTAGCTGGTGTCAGCTTGGTGAAGATCCATTGGGGGTCCTTGCTCCGCACGTGCCGAACCTCACCTATTTGAGCATGAACAGGGTCAGTAGTGCAAGCACTTTGGTTCTTTCTGCAGGGTGCTTTCCTCACCTGAAAACACTCGTCCTGAAGAAAATGCCTAACGTCGAGCAGCTGGAGATTGGACATGGTGCTCTTCCATGCATCCAAGGTCTGTACATCATGTCCCTAGCGCAGCTGGATAAGGTCCCTCAAGGCATCGAATCGCTTCTCTCCCTCAAGAAGCTTTGGCTTCTGTACCTGCACGCGGAGTTTAGAACGCAGTGGCTAACGAACGGGATGCACCAGAAGATGCAGCATGTTCCTGAGATTCGTGTCTAG

>B73_Zm00001eb200710

ATGGCAGAAGCAATAATCCTTGCTCTGAGAAAGATTGGTAGCGCTTTAGCAGATGAAACTGCCAAGAAAATGCTGGCCAAATTGTCTGAAAAGGTTAACAATCTGAGGGATCTGAATGACAAGATCGAGTCAATAAGAATGCAACTGACAGCCATGAACAATGTTATACGTAAGATTGGCACAGTATACCTCACTGATGAAGTCGTCAGGGGATGGATTGGGGAAGTGCGCAAGGTGGCCTACCATGTTGAGGATGTAATGGACATGTACTCCTATCACACACTTCAAATGGAGGAAGAATGGTTCCTGAAGAAGTACTTCATTAAAGCGTCACATTATGTCTTGGTTTTTAGTCAAATAGCAGAAGAAGTTATCAAGGTTGAGAAGGAGATCAAGAAAGTTGTAGAACTCAAAAATCTGTGGTTCGAGCCTTCGCACCTTGTTGCTGATCAGCTAATTGAGATGGAAAGACAGCGTTCGCACGATAACTACCCACTACTTTTTAAAGATGAAGATCTTGTGGGGATTGAAGATAACAGGAGAAGGCTCACCGAATGGCTATATTCTGATGAGCTTGACAGCACAGTGATAACAGTATCAGGCATGGGTGGACTTGGAAAAACTACCCTTGTAACAAATGTTTACGAGCGTGAAAAAACCAATTTTTCTGCTACAGCATGGATGGTCGTGTCCCAGACCTACACTATAGAAGCTCTGCTTAGGAAGTTACTCATGAAGGTTGGTCGTGAAGAGCAGGTGTCACCTAACATTGACAAATTGGATGTCCATGATTTGAAAGAAAACATAAAGCAAAAGCTCGATAATCGCAAATGTTTGATTGTATTGGACGATGTATGGGACCAGGAAGTGTACCTTCAAATGTCTGATGCATTCCAAAATCTTCAATCTAGTAGCATCATCATCACAACACGGAAGAATCATGTGGCTGCTCTTGCTCAGCCAACTCGTCGCCTTGTTGTCCATCCTCTGAGAAACACTCAGGCATTTGATCTCTTCTGCAGAAGGATTTTTTATAACAAGGAAGACCATGCGTGCCCCAGTGACCTTGTGGAGGTTGCTACTAACATAGTAGACAGGTGCCAGGGATTGCCACTAGCAATTGTATCAATAGCTTGCTTATTGTCTTCGAGGACGCAAACATATTACATCTGGAAACAAGTTTACAATCAGCTTCGGAGTGAGCTATCAAAAAATGATCATATCCGGGCAGTTTTGAATCTGAGCTACCATGATCTACCAGGAGACCTAAGAAATTGCTTCTTGTACTGCAGCCTCTTCCCTGAAGATTACCCTATACCACATGAGAGCCTCGTCAGGCTTTGGGTTGCAGAAGGCTTTGCATTGAGCAAAGAAAACAACACAGCAGAGGAGGTGGCTGAGGGAAACCTCATGGAACTTATCCACCGCAATATGCTTGTAGTAGTGGAAAATGATGAGCAGGGGAGGGTGAGTACATGTACAATGCATGATGTTGTGCGAGATCTTGCCCTTGTTGTTGCAAAAGAGGAGAGGTTTGGCACTGCAAACAACTATAGAGCAATGATACAGGTGGACAAGGATAAAGATGTTCGACGCCTATCATCTTATGGATGGAAAGACAGTACATCTCTAGATGTTAGACTTCCACGTCTTCGAACTCTAGTATCACTTGGAACAATTTCATCCTCCCCAAACATGCTATTGTCAATTTTGTCTGAATCCAGCTACCTTACTGTTCTCGAGCTACAAGATTCTGAAATTACCGAAGTGCCAGGGTCTATAGGGAATCTGTTTAACCTGCGTTACATTGGCTTGCGCCGTACAAAGGTCAGATCACTACCTGATTCTATCGAGAAGCTCCTGAACCTCCAGACTCTGGATATCAAGCAAACAAAAATAGAGAAACTTCCACGAGGAATCTCTAAGGTTAAGAAGCTACGGCACCTTCTAGCTGACAGATATGCTGATGAGAAGCAGTCACAGTTTCGGTACTTCGTTGGGATGCAAGCGCCTAAAGATCTGTCGAGCTTGGTAGAACTTCAGACTCTTGAGACTGTGGAAGCCAGCAAGGACTTGGCTGAACAGCTGAAGAAACTGATGCAGCTAAGGACTCTGTGGATTGACAATATTACTAATGCTGATTGTGCAAATATTTTTGCCAGCCTATCAAGTATGCCACTCCTTTCCAACTTGCTTCTTTCTGCAAAGGATGAAAATGAGCCACTTCGCATTGAGGCTCTCAAGCCTGGGTCCACAGGACTCCACAGGTTGATTATCAGAGGGCAATGGGCGCAGCGAACATTGCAGTGCCCAATATTTCAAGGCCATGGGAGACACCTCAAGTATTTAGCTCTAAGCTGGTGCCACCTTTCAGAAGATCCACTGGAGATGCTCGCTCCACATTTGCCAAACCTCACAAATCTGAGACTCAACAACATGCGTAGTGCAAGTATATTGGTTCTTCCTCCAGGATCATTTCACAACCTGAAGTTGCTTGTCCTGATGCACATGCCTAATGTCAAGCAGCTGGTGATTGGAGAAGGTGCGCTCCAATGCATCGAAGGTCTGTACATTGTGTCACTGGTGGAGCTGGATAAGGTCCCTCAAGGCATTGAATCGCTTCGCTCCCTGAAGAAGCTCTCTCTTGTCAAGCTGCACAGGGACTTCCTGACTGAGTGGAACAACAGCGGAATGCATCAGAAGATGCAACATGTCCAAGAGATTCGTGTTTAG

>B73_Zm00001eb202350

ATGGTGCATGTAGGGGAGATGCTGGCCTCGGCCGTCATTAAGGAAGTTGTTCGCAGGCTGCCTGCGCTGCTCCAGGCTCCCGTCAAAGGTCCGGCAAAGATGATCCGGAGCTTCAGGGAGGACCTCGACGAGATGAAAATGACGCTCGAGTCCATCAAGGCAACCATGGCGGAAGCAGAGCAGCGGTCTATCAACGACGAGACCGCGCGGCTGTGGCTCAAACGGCTCAAGAGGGCAGCCTATGATATCTCTGATATGTTTGATGAGTTCGAACACGGCAGCCCACCGGGCAAGAAGCTGCAGGGCCCTAGTTGGTTCGAGATAATGTTTAGCCGTGACATGGCTAAGAAAATGAAGAAAATGAATAAAAGGCTGAAAGAAATAGCAGAGCAATGGAAAAATTACGGCGGGATTGGCCGTGTATCATGTGAGCCCAAAAATGATGCTGCTGAGGAACAAGAAACAACTTCAAGTTCATCATCAGCCGTGGTTGTTGGTCGTAGAGCTGAGAAACAAGCAGTCATTAATATACTGCTTAGCTCCAATAACACATACCCTCAAGAAACTATTAGTCACTGTACTGTTATCCATGGACTTGGTGGTGTTGGTAAGAGCGAACTGGCAACATCAGTGTTCAATGACGAAAGAATCAAGGAGGCCTTTCCTCAACGGGCATGGGTTTGGTTGGGCCAAAATTTCCGCGAAAAGGATATTGGAAGAGCTATAATCTCTATAGTTGAATGTGGATCTTGTAATCTCGAGATTCTTGAATCTATATACCAACATCTCAGAAAGGTGCTCCTGGGTAGATGTCTCATTGTATTGGACAATCTCTGGGATTCAGTTCACTTAGCCAAACTGCAGGGTGAACTAGGAAGCAATGTTTCGATCTTAGTTACTTCACGCAGAGAAATTCAATTGAATATGCCCAGATCGACATTATTCCGCTTGGATCCATTGTCAGAAAGATTTAGCCTTGATTTAGTTAAGGAAGTTGCATCCTCATATTTTCCTGCAGGTGATATTCCAGAAACTGCAATGGAAGAAATTGTAAAGATGTGCGGAGGTGTACCTTTGGCCCTTAAATCTGTTGCGTCACAACTGAGACCAGAAAGAAGCGTTAAAGAGCTGTTGAGTTTAATAAGAGCTATCTCTCCGCCCAAATCAGATTATGGAACAACGGATATCCAGGACCGTGTTCTTGCATCCCTTAAGCTGACATATCACTTAATGTCCCCGAGTCTCAAGTTATGCTTTGCTTACTGTGCAATTTTCGCTAAAGGCGATGAAATTGATCGGGAAGGCTTATGCCACCAATGGATCGCTCTTGGACTAACTGAAAAGATGTATGCAGAGGACAGAGTCCGTGACTTATTGACCATGTCATTTCTTCGGGATCCAGAGCCACCTGCGATCACGAGGAGCAGTTCTGGTGGTTCATCCAAACTTAAGATGCATGATCTGGTACACGACCTTGCTATGTTAGTTGCTGATGATGAATTGCTAGTTATCAATCAGGAGTGTGTGGTATTTAAATCTGATTCACCTCGTTATGCCATGGTTTTTGCATGTAAGTTGGAAAACTTGCATAAGAATAAATTGCTAGCCGGGTTAAGGGCTCTTCATATAAAAGATTCTGATGGACTGAAATTTAAATGGTACAATTTCTCGTTTGTCAAATGCCTGCGGATCATGGATATTAGTGGGCTATGCACTGAGAAGCTTCCTTCCTCGATTGGAAATATGATGCAGCTGAGGTACCTTAATGCTTCAGGAATACAATGCGAAGTGCTCCCCAAAGCCATTGGCAGTTTATCAAAACTGCAATATCTCAATCTACATGGCAGTAGAATTTCAGCACTACCAGACTCAGTTACCAAATTGGGCCAGTTGATGCATCTGGACATTTCGGACTGTGTGCATCTGCAGACATTGCCCAACTCGTTCTGCAATCTCGAAAGCCTGTGTTTTTTGTCCCTAAAGAACTGCTGTCGACTAAGTTCATTACCTGACGATCTTGCCAGACTAGAGAATTTAGAGAAACTAAATTTGTCAGGATGCTCCTGTCTTGACACTTTGCCAAAATCCCTTGGTGAGCTGGACTCCTTAAAACTATTGGACCTATCAGGTTGTATGAAACTCACCATGCTCCCGAAATCTTTCATTAGCCTTACTAGTCTACAATACCTGAACATCTCAAGCTGTTCTGAGTTAGACATACCTGTCGATGCTCTTAACAAGCTCACAAAATTGAACTATATAGACATGTCATGTTGTCCAAAACTTGTAGGCCTCCCTCAAGAATTTTGCAGCCTTAAACATCTCCACACACTGAATCTCTCAGACTGTTCTAAATTAGCATATTTACCAGAGAAGCTGGGCCAAATGGAAAGCATCAAGTTCATTTTGCTTGATGGTTGTACGGAATCGGTGAGGAAACCTATTCTAAAACATAGGCTGGGTGCTGGTCTGCAGTCCTTACCAGCATTTGTCGTTGAAAGAAAGGCTGACAGCATACGGAGCAACATTTTCCAACTCGAACAGGAGAAGTTCTCTGAGCTGGAACTGTATCGCCTCGAGAATATACATACAGTTGATGAGGCTAAAGCACTGAAGATGCCTGATAGATCAGGACTACGTAGTTTGGGACTTATGTGGACATTAAATGTTGATCGATTCGTGGAAGATGAAGCACTGCTTCAGGCGCTTGAGCCACATGAGAATCTTAAAAAACTTACGGTGCAAGGCTACATGGGCGAAAGGTTTCCTAAATGGAAACTAGAACTTGGTTCCTCCCGCCAGGGCCATCTTCACGAAGTTGGATTAATGCATTTTCCAATGTGCAGCAGTTTGCCACAGCTTGGGCAGCTTGCAAATCTCAAGAAGCTTTACCTGTCCAGGATGCCAAAAATAAGGAGACTGGGAAGAGAGTTGTCTGATAACACTGGAGGGCTCAGGAATTTGCAAATCTTCACTTTGGAATATATGGAGAACCTGGAAGAATGGTGCACAACTATGACATCAGCTACGGGCCAGCAGCAGCAGGAAGAATTCATGTTTCCCGCGCTCCAAGAACTGACCATATACCATTGCCCTCTGTTAACGATGCGTCCCTGCCCTCCAAGAAGCATAGATTATTGGGAAGTAAGAGCAAGCAGTGGTGCAGCACAACTGCTTCTTCAGAAGGACGGCATGATGCAATCCGTGGCAGACTATATGGGCTTGCAGTGTCCCTTTGCTTACACCAGCGAGCTGCATGTTAGTGGCTCCAGCAGCAGTTCCACACTACTTCCTACTGACGGATGGAAATTCAATGGTTCTCTCATCACTCTAAAGGATTTGACAAGTGATTGCTGCAGTTTGATAGACAGATTGCTAGCCAAAGGTAACAGCATGCAATGTTTAGTTAACCTAGAGGTTTCAGGCATCAAAAATACAAATAGCTTACTGGAAGAAGTTGAATCAGTTGCATACTACACCCGATCGAGCCTCGCTAAATCATGGCCAGATTGGTTTTTACAGGAACAAGGCATAAACAGAGCATCACCACATTTTATAGTAACAGGTTATGCAAATTGTGGAGTCGATGGCTGGATTCATAAGGTGACCTCCTTTCTTGGCAACCTTATACGGATCAACATGGAGAACCTTCCTATGTGCGACTGCCTACCACCACTTGGTCAGTTGCCAATGCTGCAGGAACTGCGACTAAAAGGGATGCCTAAAATAAGAAGTATTGATCGAGACTTCTGTGGGAGTGGGAGTGGGAGTCAGCAATCGTCACACACATTGTTTTTCCCAAGGTTAACAAGGTTTGTTTTGAATGATATGCCAAACCTAGAAGATTGGGTCACTAAGGTGTCAGGTGCAAGTGATCCGTATGGCCAGGAGGAATTCATGTTTCCCAAGCTTGTCAAGTTGACAATTTGGAACTGCCCCAAGCTTAAGCTGAAGCCATGCCCCCCAAGAGCAATGGAGTGGGATATAAATAACAGCGATCAAGTAATAGCGTCAAACTATGATATAAACAGTGGTGGTTATCTTGTGACTATGCTGCAAGTATTGTTATGCAAAGTTCCACCCAGCAATTGGAAATTGCTTCATCAACTCCCTGGGATCCAAAGCTTGGCTATTGTAAGCTGCCACGGGATGGAAGCTTTGCCAGATAGCATTCAATACCTTTCCTCACTCCACTCGCTAACTGTAAGCAAATGTCATGGCCTGAAACACCTACCTGATTGGTTGGGGGACCTCACCTCCCTTGAGAGGTTGATGGTTGTGAGTTGCCCCCTGGAGTTCTTACCAGGGAGCTTGAGGCGCCTCCCTTTCCTTCGGTCGCTCACTCTGAGCCGTTGTGACCGACTGGCAGCACTGCCAGGGTGGATGGGTGATCTCAAATCACTCGTGACGATCACAATTGAAGAATGCAAGAGCCTTAAATCTTTGCCTAAGCTTTATCATCTGGAACATCTACACATTCAGTGTAATGACGAACTAGAACGTTGGTGCAAATCAGAGGTGAATCAGCATAAGTTTTCTCAAACTCTAAGGAAGGGTTTCTTCCTGGAGAGTCCTATGGGGACCAACAGCTGCATCTTACCAGCTAGATCGTTGAGTATACTTTGGGGACAGGATGACAGATACTGGAGGTTGAATTCTATTCCTGAGTCCAGGTTCGCATTATCGATGGAGCTCATAGCAGTCTGGTGGCTCGAGATTGAGGGATGGGTCCCTTTCGAGTTCCTCTCCACCGACACTAGCTATGATATATTCCTTGTCTACAAGCTGGCAGATGAGCATGATGGCCTCAGATGGGGGGAGTCCTACGTAGCAGTGGATGGAGTACACACCACTGATGGCGTTGTTTCCTTTGTAGACGAAGATGCCGTGCGTGTAGATCGCGTGGCTTACCCGGTTACCCGTTCAGAAGGTTGGATGGAGCTCTGGCTTGGTGAGTTTTACAATAAGTATGTTGATAGAGAGGTAAAAGTGAGTGTCTGGGAGAAAACCGATACCTATGCCAAGATAGGGCTCATCATTGAGGGCATGGAGATCAGAAAGAAGAGCGGGAGTATTAGCTAG

>B73_Zm00001eb226690

ATGGCAGATGCCCTCTTGGTCGTTCTCAGAAAAGTTGCTCTGTTCCTGGGAGAAGGAGCACTAGAAAAGATTGGCAAAGAGGTAGTCGAAGCAGCACCCCTCATGACAGATTTTGAGCATAGCATGAAACAAATAGAGGGTGAACTCTCGGTTCTGCAGGCCTTTATTAACCAAGTTAGTGCACAGAGAGTCAGTGACAAGGCATTTGATGCATGGTTGAACCAAGTCAGAGATGCTGCCCATGAGGTAGAAGACATCATTGATGAGTACGCTTACCTTACTGCACAAGCCGTTGATACGAGCAGCTTCTTCAAGAGAAAGTTCCACCAGATCAAGAACATTGCAGCATGGCAGAAGTTCCCAAGCCAGATCAGTCAAGTAGAAGCAAGGATTCAGAGGCTATCAGAAATGAGGAACCGATATGGTTTCTCGCTGGGTGAAATAGACAGGAATAACAATTTTCAGCTCTCCAGTCACTTTTGTCTGTCAGATTCTGAGATAGTAGGAAATGCCGATGAAATCGGAAAACTGACACAATGGCTACTTGAGGAGAAACAAGATCGGTCTCTAATTGCCATCCTTGGTATGGGAGGTTTAGGAAAAACTGCTGTTGCAAGCACCGTCTACAAGAACCAAAAGATCATAACATCTTTTGACTGTCACGCATGGGTTATTGTATCTCAGACTTACCAAGTCGAGGAACTACTAAGAGAAATTATAAATCAGCTAATAATAAAGGAAAGAGCAAGCATGGAAAGTGGCTTCATGACCATGAGTCGCATCAGATTAGTTGAGGTAATACAAAGCTATTTGCAGGACAAAAAATACTTTGTTGTCCTGGATGATGTATGGGATAAAGATGTTTGGTTAATTTTGAACTATGCATTTGTCAGAAACAGACATGGTAGTAAAGTGCTGATAACAAGTCGGAGAAAAGATGTGTCGTCTTTGGCAGCTGACAAATATGTCATTGAACTTAAAACCCTTAAAGATGCTGAATCTTGGGAGCTGTTTTGTAAGAAGGCATTTCATGCTTCAGAAGATAACATTTGTCCTGAAAATATAAGATATTGGGCAAATAAAATTGTTGCAAAGTGCCAAGGATTGCCACTGGCCATTGTAACTATTGGCAGTATTCTGTCATACCGTGACTTAAAGGAACAGGAGTGGGCATTTTTCTTCAACCAACTTAGCTGGCAATTAGCCAACAATCCAGAGCTCAACTGGATTTCCCGTGTATTAAAGTTGAGCTTGAATGATCTACCAAGTTATCTTAGGAGCTGCTTCCTATACTGCAGCATCTTTCCTGAAGATTACAAGATTAGAAGAAAGATGATTTCCAAGCTATGGATAGCGGAAGGTCTTGTGGAAGAGAGAGGAGACGGAACAACGATGGAGGAAGTTGCCGAGTGTTACCTTATGGAGCTCACTCAACGTTCTCTTTTTGAGGTCACAGAAAGGAAAACATGTGGAAGAGCTAGAACATTTCTGATGCATGATCTTGTGCGAGAGGTAACTTCAATCATTGCTAAAAAGGAGAAGTTTAGCATTGCACTTGCACATGGTGGTGCCAGTACAACCCAAGTTGCCCATGAAGCACGCCGCCTATGCATCCAAAGAGGTGCGCAGACCATTAATTCTTTAAGAAGCTCGCGGCTTCGCTCATTCATTTTGTTTGACGCTGAAGTACCATGTTCTTGGATACATGATACTGTATCATGTTTCAGACTACTGAGAGTTCTATGCCTAAGATTTGTCAATGTTGAACAAGTTCCAAGTGTAGTCACAGAACTGTATAACTTGCGTTATCTAGATATGTCATACACAAAAGTGAAGACGGTCCCTGCATCGTTCGGAAAGCTCGTTAACCTACAATCTTTGGATCTTAGAGAGACCTACGTGGAGGAGTTACCACTGGAAATAACTAGGCTAACTAAATTACGGCAGTTACAGGTGTATGCACTCTATGATATTCTACAAAGATCATCAAAATTTCTCAGTGCTACAAAAATTCCTGGTAACATTTGTCATCTAAAGGATCTCCAAACTTTGCATGTTGTTTCAGCCAATAAAGTTTTGGTTTCACAGCTGGGGAACTTGAAGTTAATGAGAAGTTTGGCTATCGCGGAAGTGCAACAAAGCTACATTGCAGAATTATGCAACTCGCTGACAAAGATGACTAACCTGAAAACACTATTTATTTCCACGTGCAATGTGAATGAGACTCTCAACATAGAAATGCTAAAGCCGCTGCCAAATCTGACATCATTTCTCCTATCAGGAAAGTTGGAGAGAGGCTTGCCCCCGTCGATATTTTCTATGAATTTAAAACAGTTAAAATTGTTCGGGTCTAGTCTGAAGAAGGATCCTGTTAGCTCGTTCTCTCATATGCTAAATCTTGTTAATCTATTCCTCACTGGAGCATATGATGGGGAACAGCTAACTTTTTGCACCAGATGGTTCCCCAATCTCAAATATCTGCAATTAGCTGATATGGAACATCTGAATTGGATTGAGCTAGAGGATGGAACAATGATGAATCTACAATATTTGTCACTTGCTGGTTTAAGGAATCTAAAGGCTGTACCTGAGGGCATCAAGTACATTAGGGCACTCCATGAGATGCTTCTGACAGATATGCCAAATGAGTTCATGCTAAAACTGCATGGAAGTGACAATCACATTGTTCAACACATACCCAACATAAAAAAGTATGACTCTTCTGATTCTCAAGCAGTAAACAACCTGGCTTATCTGCCGTGGCTTGCCAATAAGTTTGGTCCTGGTGCTGCAATTAAGTATGCCTCCATAAATTGTGGCTCATCTGGCTCTTGA

>B73_Zm00001eb226700

ATGGCAGATGCCCTCTTGGTCGTTCTCAGAAAAGTTGCTCTGTCCTTGGCAGAAGGAGCACTAGAAAAGATTGGCAAAGAGGTGGTCGAAGCAGCACCCCTCATGACAGATTTTGAGCATAGCATGAAACAAATTGAGGGTGAACTCTCGGTTCTGCAAGCCTTCATTAACCAAGTTAGTGCACAGAGAGTCAGTGACAAGGCATTTGATGCATGGTTGGACCAAGTCAGAGATGTTGCCCATGAGGTAGAAGACATCATTGATGAGTATGCTTACCTTACTGCACAAGCCGTTGATACGAGCAGCTTCTTCAAGAGAAAGTTCCACCAGTTCAAGGGCATTGCAGCATGGAAGAAGTTCCCTGGCCAGATCAGTCAAGTAGAAGCAAGGATTCAGAGGCTATCAGAAATGAGGAACCGATATGGTTTCTCAGTCAGTCAACTAGACAGGACTAACAATTTTCAGCTCTCCATTCAGTTTTCTCTGTCAAATTCTGCCTACCTGATAGATAACTCTGAGATAGTAGGAAATGCTGATGAAATCGGAAAACTGACACAATGGCTACTTGAGGAGAAACAAGACCGATCTCTAATTGCCATCCTTGGTATGGGAGGTTTAGGAAAAACTGCTATTGCAAGCACCGTCTACAAGAACCAAAAAATCATAACATCTTTCGACTGTCACGCATGGGTTATTGTCTCTCAGACTTACCAAGTCGAGGAGCTACTAAGAGAAATTATAAATCAGCTAATAATAAAAGAAAGAGCAAGCATGGCAAGTGGCTTCATGACCATGAGTCGCATGAGATTAGTTGAGGTAATACAAAGCTATTTGAGGGACAAAAAATACTTCGTTGTCCTGGATGATGTATGGGACAAAGATGCTTGGTTATTTTTGAACTATGCATTCGTCAGAAACGAACGTGGAAGTAAAGTGCTGATAACAACCCGGAGAAAAGATGTGTCTTCTTTGGCAGCCGACAACTATGTCATTGAACTTAAAACCCTTAAAGATGCTGAATCTTGGGAGCTGTTTTGTAAGAAGGCATTTCATGCTTCAGAAGATAACATTTGTCCTGAAAATATAAGATGTTGGGCAACCAAAATTGTTGCAAAGTGCCAAGGATTGCCACTAGCCATTGTAACTATTGGCAGTATTCTGTCATACCGTGACTTAAAGGAACATGAGTGGGCATTTTTCTTCAAACAACTTAGCTGGCAGTTAGCCAACAATCCAGAGCTCAGCTGGATTTCCAGTGTCTTGAAGTTGAGCTTGAATGATCTACCAAGTTATCTTAGGAGCTGCTTCCTCTACTGCAGCATCTTTCCTGAAGATTATAAGATTAGAAGAAAGCTGATTTCCAAGCTATGGATAGCGGAAGGTCTTGTGGAAGAGAGAGGAGACGGAACAACAATGGAGGAAGTTGCTGAGTGTTACCTAATGGAGCTCACTCAACGCTCTCTTCTTCAGGTCACAGAAAGGAAAGCATGTGGAAGAGCTAGAACATTTTTGATGCATGATCTTGTGAGAGAGATAACTTCAACCATTGCTAAAAAGGAGAAGTTTAGCGTTGCACTTGCACATGGTGGTGCCAGTACAAGCCAAGTTGCCCATGAAGCTCGTCGCCTATGCATCCAGAGAGGTGCCCAGACCTTGAATTCTCTCAGTAGCTCACGGCTCCGCTCATTCATTTTGTTTGACACTGAAGTACCATGTTCTTGGATACATGATACTGTATCATGTTTCAGACTACTGAGAGTCCTATGCCTAAGATTTGTCAATGTTGAACAAGTGCCAGGTGTAATCACAGAACTGTATAACTTGCGCTATCTAGACATGTCTTACACAAAAGTGAAGATGATACCAGCATCATTTGGAAAGCTCGTTAACCTACAAGTTTTGGATCTCAGAGACACCTATGTGGAGGAGTTACCACTGGAAATAACTATGCTAACTAAATTACGGCAGTTACAGGTGTATGCACTCTATGATATTCTACAAAGATCACCGAACAGCTTCAGTGCTACAAAATTTTTTGGTAACATTTGTCATCTAAAGAATCTCCAAGCTTTGCAGGTTGTTTCAGCCAATAAAGATTTGGTTTCACAGCTAGGGAACTTGAATTTAATGAGAAGTTTGGCTATTGCGGAAGTGCGACAAAGCTACATTGCAGAGTTATGGAACTCGCTGACAAAGATGCCTAACCTGAAAAGACTAATTATTTCCACGTGCAATGTGAATGAGACTCTGGACATGGAAATGCTAAAGCCACTGCCAAATCTGACAACATTTGTCCTATCAGGAAAGTTGGAGAGAGGCTTGCTCCCATCGATATTTTCTGTGAAATTAAAGCAATTAAAATTGGACTGGTCTAGTCTGAAGAAGGATCCTGTTAGCTCGCTCTCTCATATGTTAAATCTTGTTGATCTATTCCTCACTGGAGGATATGCTGGGGAACAACTAACTTTTCGCAACAGATGGTTTCCCAATCTAAAATGTCTGCAATTAGCTGACATGGAACATCTGAATTGGATTGAGGTAGAGGATGGAACAATGATGAATCTACAATGTTTGTCACTTGCTGGTCTAAGGAATCTAAAGGCTGTACCTGACGGAATCAAGTACATTAGGGCACTCCATGAGATGTTTCTAACAGATATGTCAAATGAGTTCATAGTAAGACTGCATGGAAGTGACAATCACATTGTTCAACACATACCCAACATCAAAAAGTTTGAGTCTTCTGATTCTCAAGCAGTAAACAACGTTTATATACCGTGGCTTGCCGAGAAGTTTGGTTCTGGTGCTGTGGCTCATCTGGCTCTTGACGGTTCAATGGGAGTTGTTTGTGCCAGTGGTAATCATTTTAAGTCTGAAGAGTGA

>B73_Zm00001eb226710

ATGGCAGATGCCCTATTTGTAGTTCTTAGTAAACTTGCTGATTCCTTGGGAGAACAGACACTAGAGAGGATTAGCACAAAGCTGATTGAAGTAGCACCAGTTTTGACAGATTTTGAGCATAGCATGAAGCAAATCGAGGCTGAGCTATTGATTCTTCAGGCTTTTATTGCACAGGTTGGGACAAAAGTCGGTGATAAGGCATTTGATGCCTGGCTGGACCAAGTGAGAGATGTTGCCCATGAGGTAGAAGACATCATTGATGAGTATGCTTACCTTGAGGTTCAAGCCGTGGATACTGGCAGCTTCTTTAAGAGGAAGTTCCTTCAGATAAAAAAGTTCGCTGCATGGCAGAAGTTTCATAGCCAGATCAGTCATGTAGAAGCTCGAATTCAGAGGCTAGGTGAAATTAGGAATCGATATGGCATCTTGTCAGGTGAAATAGACAGGAGTAAGAAATTGAGGAGCCCCAATCGGCTCTTTATGTCAGATTCTTCTTACTTAACTGATAACTCTGAAATAGTGGGGCATGTTGATGAAATTGGAAGATTGACACAGTGGTTACTTGAGTACAAACAAGAGCGAACTCTTATTGCCGTTTTTGGTATGGGAGGTTCAGGAAAAACTACTATTGCAAGCAGTGCATACAAGAGTCAAAAGATCACAAGGACTTTCAATTGTCATGCATGGGTTACTGTATCTCAGACTTACCAAGTTGAAGAACTACTAAGAGAAATCATAAATCAGCTAATAGATCAGAGAGCAAGCATGTCAAGTGGGTTTATGACTATGAGCGGCTTGAGGCTAGTTGAGGTAATACAAAGCTATTTGCAAGATAAAAAATATTTCATTGTCTTGGATGATGTATGGGATAAAGATGCATGGCTATTTCTAAACTATGCATTTGTCAGAAACAATTGTGGAAGCAAAGTGCTGATAACTACTCGGAGAAAGGACGTATCTAGCTTGGCAGTTGATCAGTATACAATTGAGCTTAAAACTCTTCAATATGCTGAATCCTGGGAACTTTTCTGTAAAAAGGCATTTCGTGCATCAAAGGATAACCAATGTCCTGAGAACCTTAGGTTTTGTGCAGAGAAAATTGTTGCTAGGTGTCAGGGATTGCCCCTGGCTATTGTAACCATTGGAAGTGTTCTATCATACCATGAATTCGAAGAGCAGGGATGGGAATCTTTCTACAGCCAACTTAGCTGGCAGTTAGCTAACAATCCGGAGCTGAATTGGATATCTAATGTTCTGAATATGAGCTTGAATGATCTCCCGAGTTATCTGAGGAACTGCTTTCTGTACTGCAGTCTTTATCCTGAAGATTACAAGATTAAAAGAAAAGTGATTTCCAAGCTATGGATAGCAGAAGGTTTAGTGGAAGATAGAGAAGATGGAACAACAATGGAGGAGGTTGCCAATTATTACCTTGTGGAGCTCACTCAGCGTTGTCTTCTTCGAGTCACAGAAAGTAATGCATGTGGAAGGCCGAGAGCTTTTGTTATGCATGATCTTGTGCGAGAGTTAACCTCCAACATTGCTAAAAAGGAGAAGTTTGGTATTGCATATGGCGATGCTAGTACAACCCAAGTTCCCCCTGAAGTTCGCCGCTTATGCATACAAAGAGAAGTGCCTGGCGTGGTCACAGAATTGTATAACTTGCGCTATATTGATTTTTCATACACAAAAGTGAAGACAATACCAGCATCATTCAGAAAACTTGTCAACCTACAAGTTTTGGATCTCAGATTCACCTACGTAGAGGAGTTGCCACTGGAAATAACTACGCTAACTAACTTGCGTCATCTACATGTGTTTGCAGTCCATGATTTTCAACAAAGATCATTGAATTGTTTAGGCGCAACAAAAATTCCTGTTAACATTTGTCATCTAAAGAATCTGCAAGCTATACAGATCGTTTTGGCCAACAAAGATTTGGTTTCACAACTTGGGAACTTGAAATTAATGAGAAGTTTGGCTATAGCGGAAGTGCGGCAAAGCTACATTGCAGAATTATGGAAGTCCCTGACAAAGATGCCTAACCTGAATAGGTTGGCTATTTCAACATGCAACATGGAGGAGATTCTTGATTTTAAAATGCTAAAGCCCTTATCAAATCTGGTTTTCTTCAAGCTTGCAGGAAAGTTGGAGTCAGGCGTGCTTCCTTTGATGTTGTATTATTTTGAGAAGTTAACATGGTTACAATTAGACTGGTCCGGTCTAAAGAAGGACCCTATGAGCTCCTTATCTCACATGTCGAACCTTGTTCATCTGTTCATGTGCGGGTCATATTGTGGGGAACAGCTAACTTTTTGTTCAGGATGGTTCCCCAAGCTCAATTATCTGCAATTATCTAAAATGGAGAATCTGAATTGGATTGAGATAGAGGATGGAACAATGATGTGTCTAAATAATTTGTACTTAGTTGATTTAGGGAATCTAAAGGCTGTGCCCTATGGCATCAAGTACATCAGGACACTGCACCAGATGCATCTGACAGATATGTCAAAGGAGTTTCTAGGAAGTCTGCAAGGAAGCGCGAGTCCCGTTGTTCAACATATATGCAACATCCATATTTTTGAATCCTCTGATTCTGAAGCAGTAAATAAATTCTTCTTTGAGCCATACCTTGCCACCAAGTTCGGCCCTGGTGCAACTAAGCATGCCCCTACTTACCTGGGATCATCTGGCATCTGA

>B73_Zm00001eb226720

ATGGCAGACGCGCTGTTTGTTGTTCTCAGGAAAGTTGCCCTTTCCCTGGGAGAAGGCGCGCTGGTGAAGATTGGCACGGACGTGGTCGAGGCAGCGCCCATCTTGACAGATTTTGAGCATGGCATGCGACAAACCGAGGGCGAGCTCTTGGTCCTGCAGGCCTTTATCGGGCAGGTCCGGGCGCAGAAGGCCGGTGACAAGGCGTTCGGCGCATGGTTGGACCAGGTTAGAGATGTCGCCCATGAGGTAGAAGATATCGTTGACGAGTATGCTTACCTTACCACGCAAGCCATGGATGCAAGCAGCTTCTTCAAGAGAAAGTTCCATCAGGTGAAGAACTTTGCAGCGTGGCAGAAGCTACCGATCCGGATCAGTCAAGTGGAAGCTCGGGTCCGGAGGCTGTCCGAAATGAGGAGCCGGTATGGGATTTCGGTAGGCGAACAAGACAGGGGTAGCAAGTTACAGCAATCCAATCAGTTCTCTGCGTCAGATTTTGCTTACCTAACTGATGATTCTGAAATAGTAGGGCATGGCGAGGAAATCGAGAGACTGACACAGTGGCTGCTTGAGGAGAATCAGGACCGAACTCTGATAGCCATCTTTGGTATGGGTGGTCTAGGTAAAACTACTGTTGCAAGCAGTGTCTACAAGAACCAAAAGATCAGGAGAACTTTTGATTGCCATGCATGGGTCACGGTATCTCAGACTTACCAAGCTGAAGAGCTTCTGAGAGAAATCATGAACCAGTTAATAGAGCAGAGAGCAAGTTTGGCAAGTGGCTTCATGACCATGAGTCGCATGAGATTAGTTGAGATGATACAGAACTATTTGCGGGACAAAAAATATTTTATTGTCTTGGATGATGTATGGGATAAAGATGCTTGGTTGTTTCTGAACTATGCATTTGCCAGAAACAATTGTGGAAGTAAAGTGCTGATAACAACCCGGAGAAAAGATGTGTATTCTTTGGCAGTTCACAGTCATGTAATTGAACTTAAAACTCTTAACTATGCTGAATCATGGGAACTCTTCTGCAAAAAAGCATTTTTTGCATTGGAGGGCAACATATGCCCTAAGAATCTCACGTCTTTGGTGGAGAAAGTCGTTGATAAGTGTCAAGGATTGCCATTGGCTATCATAGCCATCGGAAGGATTCTATCATGCCATGGATTAGATGAATGGGAGTGGGCATTTTTCTACAACCAACTTAATTGGCAGTTAGCTAACAATTCAGAGCTAAGCTGGATCTCTACTGTCTTGAATCTGAGCTTGGATGATCTCCCAAGTCATCTGAGGAGCTGCTTTCTATACTGCAGCCTATTTCCTGAAGATCACTTTATTAAAAGAAAACAGATAGCCAAGTTATGGATTGCGGAAGGTCTTGTGGATGAGAGAGGGGATGGGACAACAATGGAGGAAGTTGCTGAGCATTACCTTGCAGAGCTAACTCACCGTTCTCTTCTTCAGGTCATAGAAAGAAATGCAAGCGGAAGGCCAAGAACGTTTGTTATGCATGATCTTGTGCGGGAAGTGACCTCAATAACCGCTGAAAAGGAAAAGTTCGCGGTGATACATGGCCATGTTGGTACAACCCAAGTTTCCCATGATGCGCGCCGCTTGTGCATCCAAAAAAGTGCAGATTCTCAAAACTCTTTAGCAAATTCACATCTTCGATCATTCATTTTATTTGACAATTTAGTACCGTCTTCCTGGATAAATGATGTCTCATCACGTTTCAGACTGCTGAGGGTCTTAGGCCTAAGATTTACTAATATTGAACAAATGCCGTGCGGGGTCACAGAACTGTATAACTTGCGTTATCTGGATATTTCGTACACAAAAGTCAAGCAGATACCGGCATCATTCAGAAAACTCATGCACCTTCAAGTTTTAGATCTGAGGTTCACCTGCGTGGAGGAGTTGCCATTTGAAATAACTACGCTAACTAATTTACGCCATTTACATGTTGCTGCGGTCCATGATCTTCAAGAAAGATCACTGAATTGCTTCAGTGCTACAAAAATTCCTGGCAACATTTGTGGCCTAAAGAATCTTCAATCTTTACATACGGTTTCAGCTAATGAAGATTTGGTTTCACAGTTGGGAAATTTGACTCTTATGAGAAGCTTGACTATAATGAATGTGCGGCAAAGCTATATCGCAGAGCTATGGAACTCCCTGACAAAAATGCCTAACCTGAGTGTCCTGATTATTTTTGCATCTGATATGGATGAGATTCTTGATTTGAGAATGCTGAGGCCCTTACCTAACCTTAAGTTGTTCTGGCTGGCAGGAAAGATGAAGGGAGGCGTGCTCCCATCGATATTCAACAAGTTTGAGAAGCTAACACAGTTAAAAATGGACTGGTCTGGTCTGAATAAGGACCCTATAAGCTCCTTTTCTTACATGCTAACTCTAGTCGATGTGTGGTTCTTCGGGGCATATTGTGGGGAATATCTATCTTTTTGTGCTGGATGGTTCCCCAATCTCAAATCTCTGCACATTGCTGATATGGAACATCTGACTCGGATTGAGATAGAGGATGGAACAATGATGGGCCTGCATCATCTGGAACTTGTTGGTTTAAGGAATATGAGGCTAGTTCCTAAGGGTATCAAGTATATTAGAACACTCCGTCAGATGATCCTGACTGATATGCCAAAGGAGTTAGTTGAAAGCCTGCGAGGAAGTGACGCGCACATTGTTCAGCATGTTCCCAACATCCATATTTTTGACTCCAGTGATCCTGAAGCAGTAAATAACTTCATATTCTGGCCTCATCTTGCCAAGAAGTACGGCTCTGGTGTAACTAAGTATGACCCTAGAAAATGA

>B73_Zm00001eb253770

ATGGACGGGTTCATGGCAAGTGCAGCGACAGGGGTGATGAGCTCCCTTCTCGCCAAGCTCGCTGAGCTGCTTGGGGAGGACTACAAGATGCAGAGGGGCATGAGGCGCGAGATCGCGTTCCTCAAGGATGAGCTGGGCAGCATGAACGCGCTGCTGGAGAGGCTAGCCGGCTCGGAGGCGCTCGATCCGCAGACCAAGGAGTGGAGGGACCAGGTGAGGGAGATGAGCTACGACATCGAGGACTGTGTCGATGGTTACATGCGTCAGCTGCAGCACGAGCCGCAGAGGAACAGTGGAATCACGGGATTCTTCCTTGGGTATGTGCAGAAGGTGAAGGATCTCGTTACCCGTCACGAGATTGCCGAGCAGATTCAGGAGCTCAAGGCTCGGATCGTTGAGGCTAGCCACAGAAGGAAGAGGTACAAGATTGATGACACAGCTAATTCTGGTGCCGCCAATGTGATCCCTGTGGACCGTCGGTTGCCAGCGCTCTATGCAGAATTGGGTAGCCTTGTTGGTAGCGATGTTCCTAGAGATGAGATTATCAAGCTACTTGATGATGGGGCGCTGGCCGTAAAGGTGGTGTCTATTGTGGGCTGTGGAGGACTGGGAAAGACTACTGTCGTGAATCAGGTTTACATAAACATCGCCGAGAAATTTGATTGCCAAGCCTCTGTGTCCTTGTCCCAAAATCCTGATATGGTGAACATATTCCGGTCGATACTGTCTCAAGTCAAGAAAGATGAGTGTGGTAGCACCAGCTCATGTGACAAGGAACTTCTCATCAATGAATTGAGGGATTTCCTTAAGGACAAGAGGTATTTTATTGTAATTGATGACATATGGAGTACCCAAGCATGGAAGACAATTAAATTTGCTTTGGTTGAGAATACTTGCGGCAGTAGAGTAATAGTGACAACAAGAATTGGTACTATTGCCAAATCTTGTTCATCCCCATTCCATCATCTCGTATATGAATTGAGGATGCTAGGTGAAGATGACTCCAAAAGGCTATTCTTTAGAAGAATTTTTGGCTCTGAGGACAAGTGCCCTCACCATTTAAAAGAGGTTTCAGTTGAAATAATTAAGAAGTGTGGTGGTTTACCATTGGCAATCATTACTATGGCTAGTTTGTTGACTACTAAATCATATACCAGAGCTGACTGGTTGAAGGTTTCTAATTCAATTGGATCTGGGCTAGAGAAAAATTGTGATGTGGAGGAAATGAACATGATATTATCTCTGAGTTACAATCACCTTCCTCATCATTTAAAGACTTGTTTATTGTATCTAAGTATGTTTCCTGAAGATTATGTGATCAAGAGGGATTATTTGGTAAGAAGGTGGGTAGCAGAAGGATTTGTTAGTGCACATGGTAGAAGAAATCTGGACGATGAAGGCGAATGCTATTTTAATGAACTTATCAACAGAAGCTTAATACAACCGGTAGATTTTCAGTATGATGGTAGAGTATATGCATGCCGGGTTCATGATATGATTCTTGATCTGATTACATGCAAGGCTGTTGAAGAAAATTTCATAACTGTTGTTACTAATAGAAAACAAATGTTGCCCTCACATGGCAAGGTCCACCGACTGTCACTTGAGTACCATGGTCTTGAAACCTTAAGAACAAATCCCATTTTTACTACTCATGTTCGGTCCCTGAACATATTTAGATACTCTGAAGAAATGCTTCCTCTTTCAGGCTTGCACTCCCTAAGAGTGCTTGATCTAGATGGCAATGAGAATTTGGAAAGCTGTTACCTTGAAGATATAGGGAAGTTATATCAGTTGCGATACCTACGGATTAAGGCAAGTAATATTACACTTCTGGAAAGGATAGAAGAGCTTCAGTGTTTGGTAATACTGGATCTTCTGAATTGCCCTAATCTAGGTGAATTGCCTAGAAGTATTGTTCAACTTCGGAACTTGAAATGGTTAACTGTTCATCGAGCGAACTTGCCAGATGGAGTTGGGAACATGCAAGCGCTAGAGTTTCTTTCACTTGTAGTTGTGGACTACACTACCTCAACAAACTTATTGGAAGAGCTGGGCAGCTTGACCAAATTGAGAACTCTTAGGTTGGATTGGCGCATCAACCCCCTGCACAGGGATAAAAAAACATATGAGGGTAATTTTGTTTCTTCACTTGGCAAACTAGGCAGTTCAAACCTTCGATACCTAACACTCATCAGTCCATGGTCACTCGACTTCCTGTTGGAACCTTGGTCCCCAACTCCACATTTCCTTCAGGAGTTAGTGATCAAAGGATGGCATCTCAACAATATTCCAGTCTGGATGGCCTCGCTAACCAACCTCACCTACCTGGACGTTGAGGTTAAAGTTAGACAAGAAACTCTCCAGATCCTCGGAGATTTCCCTGCCTTACAATTCCTGAAGGTGTCCTCAAATGCAGCAGGATCTGAGGCAATGTGCCTTGTCGTCAGCAACGATGGATTCCGATGTCTGAAGAAGTTCAGTTTCGTTGGCTGGGTAAACATGATGTTCAAAGAAGGAGCTGTTCCAGCGCTTGAAACTCTTGAGTTTCAAATCATAGCGCACGAGATGCATACTGCACGCAGATTTGGTCCTCCTGATTTCGGCATCAGCCACCTCTCCACCCTCAGGAATCTCGTCGTCAATGTTCACTGTGAAGGTTCAAGGGTTAAAGAGGTGGAGGCAGTAGAGGCTGCTATCCAGAAGTCAGCCAGTACGCTTCCTAATTATCCCACACTAGGTTTGCACAGATTTCTCGAGTCAGAACTGGTAAAAGAATGA

>B73_Zm00001eb261660

ATGGCAGAGATTGCTGTTCTTCTCGTCCTGAAGAAGATTGCCATAGCTCTGGCAGGAGAGACCCTAAGTTTTGCTAAACCATTGCTTGCAAAGAAGTCTGAGTCGGTGGCAGCACTCCCAGATGACATGAAACTGATTAGTAATGAGCTCGAGCTTATCCGGGCGTTTCTCAAGGAAATCGGCAGGAAAGGCTGGAAAAGCGAAGTGATAGAAACATGGATAGGGCAGGTCCGAAGACTGGCTTATGATATGGAAGACACTGTAGACCATTTTATTTACGTTGTTGGTACACACGATCAGATGGGATCATGCTGGGATTACATGAAGAAGATAGCCAAGAAGCCTCGGCGTCTGGTTTCACTAGATGAAATTGCTAGTGAGATTAAGAAGATAAAGCAAGAGCTTAAACAACTCTCGGAAAGTAGAGACCGCTGGACTAAACCCTTGGATGGCGGGAGTGGTATACCTGCAGGAAGCTATGAAACTGAAAAGGAAATGTATCTTCCTGGACATGATTACACAATCAGCGACGAGGAGCTTGCAGGAATTGATGAAAATAAGCAAACCTTGATTAGTTCATTAAAATTTGAAGATCCATCACTTCGGATCATTGCTGTCTGGGGTATGGGTGGCGTTGGAAAAAGCACTCTTGTAAATAATGTGTACAAAAATGAAGGATCCAACTTTGACTGCCGTGCATGGGTTTCTATCTCTCAGTCATATAGACTAGAAGATATATGGAAGAAAATGCTGACAGATCTCATCGGGAAAGATAAGATAGAATTTGATCTTGGAACAATGGATAGTGCAGAACTAAGAGAGCAATTGACAAAAACTCTAGACAAAAGGCAGTACCTGATCATACTGGATGATGTCTGGATGGCTAATGTTTTTTTTAAAATTAAAGAAGTTCTTGTAGATAATGGCCTTGGAAGCAGAGTAATAATCACAACAAGAATTGAGGAGGTAGCTTCACTAGCTAAGGGTAGTTGTAAGATCAAAGTAGAACCTCTGGGTGTCGATGATTCCTGGCATGTATTTTGTAGGAAGGCATTTCTGAAAGATGAAAACCATATCTGCCCTCCAGAGTTGCGTCAGTGTGGTATAAACATTGTGGAGAAATGTGATGGTTTGCCATTAGCCCTTGTGGCAATAGGAAGCATATTGTCGCTGAGACCGAAGAATGTTGACGAGTGGAAGCTATTTTATGACCAGCTTATCTGGGAGCTACACAACAATGAGAACCTTAATCGCGTGGAGAAAATTATGAATCTAAGTTATAAATACTTACCAGACTATTTGAAGAACTGCTTCCTGTATTGTGCTATGTTTCCAGAAGACTATCTAATACACAGAAAGAGATTGATTAGATTGTGGATAGCTGAAGGATTTATTGAACAAAAAGGGGCATGCAGCTTAGAAGACACTGCTGAAAGTTATCTTAAAGAACTTATACGACGGAGCATGCTTCACGTTGCAGAGAGGAACTGCTTTGGTAGGATTAAATGTATTCGAATGCATGATCTTGTGCGTGAACTTGCCATTTTCCAATCTAAAAGAGAGGGTTTCAGTACAACTTATGGTGGAAATAATGAAGCAGTGCTAGTGGGATCATATTCTCGACGAGTGGCTGTGCTCCAATGCAGCAAGGGCATTCCATCAACCATTGATCCATCCAGGCTTCGCACCTTAATTACATTCGACACCAGCAGAGCATTATCTGTGTGGTATTCTTCTATTTCCTCCAAACCAAAGTACCTTGCAGTATTAGACTTATCAAGCTTGCCTATTGAGACTATTCCAAATTCAATTGGAGAGCTTTTCAACCTTAGGCTTTTATGCCTCAATAAAACCAAAGTGAAAGAGCTCCCTAAATCTATTACAAAACTTCAAAACCTACAGACAATGAGTCTTGAGAATGGGGAGTTAGTGAAGTTCCCACAAGGGTTTTCAAAACTGAAGAAATTGCGACATCTTATGGTTTCACGGTTGCAAGATGTAACTTTCAGCGGTTTCAAAAGTTGGGAAGCTGTGGAGCCATTTAAGGGCTTGTGGACTTTGATTGAACTGCAAACTCTGTATGCCATTACAGCAAGTGAAGTATTAGTTGCAAAACTTGGAAATTTATCCCAGCTGAGGCGGCTTATAATTTGTGATGTAAGGAGTAACTTATGTGCACAGTTGTGTGGCTCTTTGTCAAAGTTGTGCCAGCTGTCACGATTAACGATAAGAGCATGCAATGAAGATGAAGTGCTACAGCTGGATCATTTGACATTTCCAAATCCTCTTCAAACCCTTAGTTTAGATGGACGACTGTCAGAAGGAACTTTCAAATCTCCCTTTTTCTTAAATCATGGAAATGGGCTTCTTAGGCTAATGTTGTTTTACAGTCAGCTTTCAGAAAATCCAGTACCACACCTCTCTGAATTGTCAAACTTGACTAGGTTATCCCTTATAAAGGCATACACCGGCCAAGAATTATACTTCCAAGCAGGTTGGTTCCTGAATTTAAAAGAACTTTACTTGAAGAATTTGTCCCGGCTCAATCAAATAGACATACAGGAGGGAGCTTTGGCCAGCCTTGAACGTATAACAATGAAACACCTCCCGGAGCTACGGGAGGTTCCAGTCGGTTTCAGATTTCTCAAGTCCCTAAAAACAATATTTTTTTCCGATATGCATCCTGAGTTTGAAAGCAGCTTTCAAAAGGAAATGTAG

>B73_Zm00001eb271410

ATGGCGGAGATAGTCACCGGGGCGATGGGCACTCTCCTGCCCAAGCTGGCCAACCTCATCAAGGAGGAGTATAACCTACAGAAGAAAGTGAGGGGTGAGATCATGTTCCTGGAGACTGAGCTCAAGAGCATGGAGGCTGCTCTCATCAAGGTCTCCGAGGCACCCATCGACCACCCACCTGACGTCCAAGTCAAGCTCTGGACAAGGGAGGTGAGAGAGCTGTCCTACGACCTCGAGGACAGCATCGACAGATTCATGGTGCGAGTTGGCGATGGCAAGCCACATAGTTTCAAGGGATTCATTGATAGAAGCCTCCACCTGCTGACAAGGGGCAGGATTCAACACAGCATCGGCATAGACATCAAGGAGATCAGGAGCCGCATAAAGGATGTGAGTGAACGGCGTGACAGGTACAAGGTTGATCTGGTTCCTTCCAAGCCTGTTGGCAGAAGCATTGACAACCTGCGGCTGTCGGCTCTTTACAGAAAGGCGACAGAACTTGTTGGCGCCGAAGAGAAGAGCAGTGACCTTGTGAAAAGGCTCATGGAGGGCGACAAGGAGGCATCCAAGCAGCCAGTTGTACTGTCTATTGTTGGCTTTGGAGGGTTAGGCAAGACTACTCTTGCTAATCTTGTGTATGAGAAGATTAAAGGGCAATTTGTCTGTGGGGCATTTGTTTATGTGTCTCATAATCCTGATGTCGTCAAGGTTTTCAAAAACATGCTCTACCAGCTTGATGGAGACAAATACAGGGACATCAATCAAGGAACATGGAGTGAAGAACAACTAATCTGGGAACTGAGGAAGTTCCTTCTACACAAGAGGTACTTTATTGTCATTGATGACATATGGAATACTTCTGTGTGGGAAACAATCCAATGTTCTTTGATGCACAATGAATGTGGAAGTATAATAATCATCACAACTCGTAATATTGATGTTGCAAAACAAGCTGGAAGTGTTTATCAAATGGAACCTCTTTCTCTCAGCGACTCAACAAAGTTATTCTGCCAAATAATTTTTGGCAGTGAAGACAAATGTCCTCCAGCTAATTTAGCTGAAGTGGCTGGTAAAATCTTACAGAAATGTGGTGGTGTACCATTAGCTATCATTACCATGGCAAGTATGCTAGCCAATAAAACTGGAAAGGAAATAAACACACATAGCTATTGGTCACATGTGTACCAATCCATGGGTTATGGTCTAGATGGCAGTACTAATGTGAAGAATATGAGAAGGATACTATCAGTTAGTTACTATGACCTACCTTCACATCTAAAGACTTGCTTGTTATACCTAAGTTTGTATCCAGAGGACTACAGGATTAGAACAAGAGGTCTGATATGGAAATGGATTGGTGAAGGTTTTGTCCATGAAGAACAAGGGAAGAGCCTATATGAAGTAGGTAAGGATTATATTGAAGAGTTAGTTAACACAAGTATGTTAGAACCTGTCGGGATTGGCCATGATGGTAAGACCGTATCTTGTCGGATACATGATATGGTCCTTGATCTTATCAGTTTCTTGTCAAATGAGGAGCATTTTCTAACAAAAGTAGGTGGGCAACAACCCGTATCTCTTGATCTGCCTAAAAAGGTCCGCCGGTTATCCCTCCAAATTAGCCAGGAAGAGGAAGCCAAGCAGCTAGCTACAATGAGCTTCTCCCACGTAAGGTCACTTACTGTGTCCACTGAAGTTTTCCAGTTGACGCCAAAACTTTCGGCCTTTCTGGTCTTACGTGTATTGAATTTAAAGAAATGTAATGGAGTGAACAATCACCACTTTAAAGATATTTGCAATATGTTTCAGCTGAGATATTTGAGTCTCAATGCGAAATTTATTACTGAGATCCCAAGGGAGATTCGGAATCTGCAATTTTTGCAAGTACTTGACATAACTAATCTTGGGCACAAAGTAAAGATGACAACCATTATTCACTTGCGACAGCTGTTGCGGCTTTGTTCTAGGTCTGGGTGGAGCATAAAACAGCTAGACGGATTTGGAAAACTAACCTCTCTACAAGAAGTTAAAGGGACCATAACTATCGAGTCACCAAGCATGCTGCATGATCTGGGGTGTCTGACCAATCTCAGGACCTTGGGCATCAACTTTCGTGATTGGGATGAGAGCTATGAGGAACCTTTCATCCAATGTCTATCTAACCTTGTCAGCCTCAAATCCATGAAAATAAAAGGTACCATGATGAGCAGCCTATGTTCCGAATGTGACAAATTGTACCCTGGTCCTCAACAACTTTGCTCCATTGATATGAAGTCACTTGCAAGAACTGTCATCATGACTAGAGTACCAAGATGGATGTCATCACTCTGCTCCCTGTCTAGCATAAAGATCACATTATTAGCTCTGGGAGTACAAGATATCCATGTCCTTGGGAGCATACCATCTCTACGTTGTCTCAGTGTACATGTGAAGGAAACCAGAGATGAAAGGTTGGTCATTGACAAGTGTTATCCATTCCGGTGCCTAACTGAGATGCAAATCGATTATGAATCCATGGCGGTGGTGTTCGCACCAGGGAGTATGCAAAACCTCAAAGAACTCCATTTATTGTTCGGGGTGAAAGAGGTAATGCATAAGTACGGTGATTGTAACTTTGGTTTGGAGCACCTCATGTCACTTGAGCATGTCTCTGTTAAAACAATGTACACTATCATGCCCGAGGAGGTGGAGGCCGTAAAAGATGAATTCCAGAAATCCCTGGACATGAATCCTGGCAAGCCCACGTTGATAGTAGATTATTTGTATCCGAAAGAAAGGAAGATTAGGTCTCAAGCACAAGCAATAAGAGCAGCAATTTTGTTCGCTAATGCAGGCCGCATCCCTGCTACTGACTGA

>B73_Zm00001eb283180

ATGGAGCTGGCGGTGGGCGCGTCGGAGGCAACCCTCAAGTCACTGCTGAGCAAGCTCGGCGCCCTCCTCGCAGAGGAGTATGCCCTGATCCGTGGCGTCCGGGGCGACATCCAATTCATCACCGACGAGCTAGCCAGCATGCAGGCCTTCCTCAGCAACCTGAGCAAGTACGAGGAGGGCCACGACGACCAGACGGAGGACTGGATGAAGCAGATCCGGGACGTCGCCTACGACATCGAAGACTGCATCGACGACTTCGCCCACAGCCTCCGTCCAGACCCCAGGGGCAGCGGCTGGGTGACGGCCGTCCGCAAGATTCTCTACGAGATCCGGACGTGGTACCCCCGCCGCAACATCGCCACCCAGATCGTCGATCTGAAGAACCGCGCGCAGCATATCGGCGAGCGCCGAACCAGGTACGGCGTCCGCGACCCGCAGCCTGGGAAGAAGAAGAGCAACTTGGGGGGCGCTACGGGGTATCTCGCTGCTGAGAATCAGGACGTGACCCGGCAACTCGTCCGCGCCCAGCAGCCTGTGGGGGTGAAGGATATGCCGGATCTTAAGAAGTGGATCCATGAAGATGGCAAGAGGGAGAAAACCGGCGTGCTGAGCATTGTCGGGTTTGGTGGTGTGGGTAAAACCACCATCGCAATGGATCTCTACACAAAATACGGGCCTGAATTCCAGCGTCGAGCAATGGTCACTGTGTCCCAGAACTCAGATCCTGAGGCGGTCGTCAGAAATATACTGAACCAGGTCAAACCACTGTCCAACAACGCGGAGAGGCGAGGCGAAGATAGCTCTGGCGCCGTCTCCTTGGGGAAGAAGAATCCAGTCATTGCAACTATATTAAGCCGAATTAGGCTGCCGTTCCCAAATCAAAAGCAAGATAATGGTGGCGGTCGTGACAAGCATGAACAAATAAAAAAGGAGTTGAAGAATTGCCTCGCAGATACAAGGTACTTACTGTTAATTGATGATGTATGGTCATCATCAACATGGCAAAGTATTTGGAAATATTTTCCTGAAGAAAATAAGAAGGGAAGCAGAATAATTGTCACCACACGGTTTCAAGCTGTTGCCACGACATGCTCTGCACATAAAGATCAAGATCATATTCATTTGGTTGATGTTCTTTCCGGTGAAGAGGCCAACAGTTTATTCATCAAAACCTTGTCCGAGTGTAGAGGTAATAGTGCCAGACAATCAAACCAGACCAAGGTGCCAGACAGAGTTTGGCAGATGTGTGGTGGCTTGCCGTTGGCCATAGTTACCATGGCAGGTGTGGTGGCATCCAAGCCACTGATGATCAGAGATGAATGGATTGCCGTTTGCAATTCCTTGTTTCCGGAGCCAGAAAAATGTCATAAACCAGAGGAATTTATGAGGATAATAAACTATTGCTACAACGACTTGCCTAGTGATCTCAAGACTTGCTGTCTGTATCTTAGCATATTTCCTAAGGGCCGTGAAGTTAGCAGGAAGCGGCTGATTCGGAGGTGGATAGCGGAAGGTTTCGTCAGTGAGAAGCAAGGTTTGAGTGTGGAGGATGTTGCTGACACATGCTTCAAACAGCTCATTGAAAGGAAGATGATGAGGCCCGTTGAGCACAACAGCAATGGAAGCGTCAAAAGTTGTCGGGTCCATGACATGGTACTTGAGTACATCATTTCCAAGGCAGCAGAGGAGAATTTCATCACTGTGGTCGGTAGCCACTGGTCCATGTCAACAAGTAGCAAGAAAGTTCGTAGGCTCACTATCCATGGCAGTGACCCCAAACGTGCAAAGAATGTTGACAGTATGAACTTGTCACATGTCCGATCACTGACTGTGTTCGAGAACCTGGACAAACTGCACTTCAAATCATTTAAAACTGGAATAGTGCAAGTGTTAGATCTCGAAGGCTGCAAAGGTTTCAGGGAGAGCGATGCCAACGTTTCAGACATATGCGAAATGATTCTACTTAAGTATCTGAGCCTACGGAAAACAGACATAAAAAATCTTCCCAATAATATTTCCAAGCTCAAGTACCTAGAGACTCTCGATGTTAGGGAGACAGAAGTTAAACAGCTGCCTACGACTGTAGGGCAGTTGGAACGGATAACTAACATCCTTGGTGGTGATAAGAGAAGACAGAGAACCCTGAAACTTCCTAAGGAGCTCAAGGGAACAATGAAATCTTTACGCATATTGTCGGGGGTTGAGATTGTGGAGGGATCAACAGCTGCATCAGACTTCAGTTACTTCACTACTCTGAGGAAGCTGGCAATTTACAGGATCCACCACAATGAAGATATATTCAAAAATTTGCTGTCCTCGATCCAGTACCTCAGTGGTTATTCACTCCAAACTCTTTTAATCGTTGATGAGTCATCTGAATTCTTCAAGACCCTGGAGTCAATGTCGCCATCCCATCTAACTGACCTGAGAGCTCTGGAGCTGTCTGGCAAATTGCTTTACCTTCCAAAGTGGCTCGACACTCTTCAACATCTTGTGAAGTTAACGCTTTCAGCAACAGCCCTATGCACTGATAACTTTTTGGTCATCAGAAAACTGAACTCGTTGTTTTCCCTCACCTTTTCAATCAGCGAAAAGCAAGACCCTGCTTTGGCAGCCATTCTTGAGAAAAATAAGTCAGCTTCAAGAGGAGAAATCGTTGTGCCAGCTGGAGGCTTCAGTAAGCTCAAGCTGCTTCGGATATTTGTTCCTCTTCTTCCATCCCTTACCTTCTCAAAGAATGCTACACCACATCTGGAAAGGATTGAACTGCGTTTTAAAAAGCTGGAAGGATTTCATGGTGTGGATGAACTTGGAAAGCTCCAAGATGTGGTCTTAACAGTTGACGGGCATGCAAGTGAGAGGACAAATTCGATACTAGACGGATTGAAGCAGAGGCTAGAAGGAAAGTGCAACCTCATTGTCAACAAGTATCACGACTGA

>B73_Zm00001eb283200

ATGGAGTTCGCAACTGGAGCGCTAGGCGCCCTTCTTCCCAAGCTCTCCATGCTGCTTCATGGTGAGTACAACCTGGAGAAGGGCGTCAGGGGGGACATCCAGCGCGTCATGAGCAAGCTCGAGCGGGTTCATGCTGCCCTCCGCCATGTTGGCGAAGTGCCTGTGCCACTGGAGCAGATCATTCGTCCTGGTATAGTCAATATGTGGGCACGCGACGTCGGGGAGCTATCCTACGACATGGAGGACTTCGTCGACACCTTCCTGGTGCGTGTCCAAGGCCCTGAACGCACCAGCAAAAGAAGGTTATTCATTAAGATGATAGATATGGTCATGAATAATCGCCATGATGAGATCGCCCCAGACATCAAGCACTTCGAAAAGCGCGTCCAGGAGATGGATGATCGTCGTCAAAGGTACGGTGTTGGTACTATTGTTCCTACCGTCAAAACCTTGTTTTATCCTCGCATAATTGCTCTAAACTACACCAAGGCCACGGACCTTGTCGGCATCGATGAGGCAAGGGAGGAACTGATCACAAGGTTGACCAAGGAATATGACACCTCCACTGAACAAAGGCAAGTCTCTATCGTTGGTTTTGGAGGACTTGGCAAGACAGCGCTTGCAAAAGCAGTTTATAACAAACTTAAAGCTAAAGGGGAATTCCATTGTGCGGCCTTTGTGTCGATGTCTCGGTATCCTAAGCTCGTAGAAATCTTCAAGGAATTGCTTTATGAGCTTGACAAGACTGAGTACAAGGACGTTATTAGCACCCCAATGGAAATAGATGAACTTATAAATCTAGTGCACGAATTCCTTAATAAAAAGAGGTACCTTATCGCTGTTGATGACATATGGGATACTGACGCATGGGCAATGATACGATACGCTTTTGCTGAGAATAAGCTAGGAAGCAGAATAATCGTAACTACTCGCAGAATTGATGTTGCTGAGTATGTAGGTGGTTGTTGCTATATGATGAAACCTCTTACTCGAGAGAAATCAAAGATATTATTCTATGGACGAACATTTGGTTCTGAAGGTAAATGCCCTCCTGAACTTTCTCATGCGTCTGAGAAAATATTGAATAAATGCGGAGGCGTGCCATTGGCTATTATTACTATATCTAGCTTGCTGGCTAGTAAGTCAAAAAACATAAAAGAATGGTTCTATCTTGCTGATTCTATTGGTTCCGGAATACTAGAAAGAAGTGCTGAAATGGAGATTATGTGGAAGATACTGCTGCGTAGCTATTCTGATCTACCAGCACGATTAAAGACATGTTTGTTATATCTGAGTATTTTCCCTGAAGATTGTGAGATTGGGATGCATCGGTTAATATGGAGGTGGATAGCTGAAGGTTTTTTCAATGGAGAACTAGCACATGGTGGGCTCTTTAAGATTGGGGAATCTTGTTTCCACGAGCTCATAAGGAGAAGCATGGTGCAGCCAGTAACACTTGAAGGCACGGGTCTTGTATATGCTTGTCGTGTTCATAATATGTTTCATGATTTGATCCTCTCCATGTCACATGAAGAACAATTTGTTTCTGTAGTCAATGAAAAATTTGGTCCTCTTGATGTTCTTTCTCGGCGGTTAGCATTCCAGAACATAAAAAAAAGTCAGTACAGACTTGTGGAACATCCACGGCTGGCACAATCGAGGTCACTTAATGCCATTGGATGTCCTATATACGCGATACCTCCAATTGAAAGCTATAAATCATTGCGTGTACTGGATTTCGAAAATTGTGCAGGTATTGAAGACCATGATCTTGTTCATCTTGGGAAATTGCATCACCTCAAGTTCCTTGGGCTAAGAAACACGTTTATCGGTAAGCTGCCGGAAGGAATAGGGAACCTCAAGTTTCTGCAAACATTGGACCTCGATGGAACTGGTGTGGAAGAATTACCTCAAGCCTTGCATAATCTTACAGAATTGATGTGCCTAATTGCTGACTGGAGAACGAGAGTGCCCAATTGGATTGGTAACCTCACGTCCCTGCAGCACTTGGTGATTTATCCTGGTGGGCATGACGATGAGGATTCTGCGAGCAGGTTTGTTAATGAGCTGGGAAAGCTGAGACAACTAAGGGTGCTCCGTTTTTTGATAAAAGCACAAGATGAAGGGCAGCTGAGAGATTTGCTAGAGTCCCTATCGAATCTGCCAGAGATCGAGGCTATACATTTTGATTACTATGGAGTACAGTTAAATAGAGGTGTTCAGTTGGAACCTGAAGGCTATGCCCTCTCTAGACATATTCGTTCCGAGAAATTTGCCATTATGGAAGCCGCACCGACGGCTTTCCATAATAGCAAATTTCTCATTCGTTCCATGGAATTGCGCTGGTTGGAGTTCTCAAGGCTGCCTCTTTGGATTAATCCTGGACAACTTCCTAACCTCTACCACTTATGGCTGATGGTATCTGATGCGGAAGAGCGGGATCTGGAAATCCTTGGGGGGTTTCCAGTGCTTCACTCCCTCCACTTGTTGATTGTGAATACTGAACGTGAACATGTCATGACTTGTGGCTGTGGTGGATTCAAGAATTTGAAATGCTGCAGTATAACTAAACCGCTGAAATTTGTACATGGAGCTATGCCCAGGCTTGAAGTCCTCGATTTCCATTTCAGTGTGCAACTCCTAACGGATTCAAACCAAGAATTTGATTTTGACTTTGGCTTGGGAAACCTACATTGGCTTCAGCAAGCCATCGTTCAAATCACAGCCCTTGGTGAGGAGGTGGAGTCTGTGGGGAGAGCACAGGTGGCTCTGCGGGATGCAATACGTACCCATCTCAACCGTCCTACCCTTGAAATAAACTTATTTGGGCAAACAATACCTCCAGAGTTACCAAAGCAAGACGACGATGGAGCGAAAATTGTGGAGATATCACCGGCCGAGAGTAGTCGTCAAGCTCAAGAGCGGGAGAAAAGAAGCATCGATGTGGCGACGAAGAAAGCAACACGGGTGCCGTCTTTTTACACAAAGTCATCAATTGATGAGCCAATGGATCAGCTCATAAACATGCTATCTGTGGTTGATGACGAAGCCTACACTAAGAACATAAAGATACTATCTATTGTAAGGTCTGAGGGACTGGGGAAGACTACTCTGGCCCAAAAAGCATTCGAAGAGCTCCATTCGCAATTTGACCGTGGGGCGTTCGTTCTACTAGGCCAGAATCCTGACTTGAGGAGAGTTTTTGCTGACATTCTCCGTGGTCTTGACAAGCAAAGGTACATAGATTTCCCAGTGGCAATATTGGATCTAGTGGACCTGATCTGGCTAGTCCGTAAATCGCTCATAAACAAAAGGTTCTTTATTGTATTTGATGATATATGTGATGTAAAAGCATGGGAAATTATAAAGTGCGCTTTGATTGAAAATAACAACCACAGTGTAGTTCTTACGACAAGTCGCAACACTGGTATTACTGAAATTATTGGTGGCAGCAAGCAATTACAACCTCTATCAGCAACTATCTCTAAAAATCTACTCTGCAAAAGGTTATTTGGATCGGCAGGCAAGTGTCCTTCTGAACTAGTAAATATATGTGACAATCTTGTAGAAGAATGTGGTGGAATACTATCTGTGATCGACGAAACTGTGACATTGCTTGCAAGTATACCACCAACAGTGGAGAACTGGGAGGCAGTGTACGCCAGAAGAATGTTGGATCGGTCTTATCCTGGTTTAACTGACAGTCTAAAGAATTGCTTACTCTATTTTACTATGTTTCGAAGAGGACATGAGATTAGTGGAGAACACTTAATATGTGCATGGATAGCTGAAGGTTTTGTACATGGGCAAGAGGTAGCAGAGACCTACCTTAGTGATCTAGTAAAAAAGAAATTAATCGATGCAGTGGAGGTTGATGCTGGAGGAAAGGTCCTCACGTGCCGCATGTATGACTTGGTGCATGACTTTATCGTCTCAAAATCAATTGAAGAACGATTTGTTTATATTTTAAATGACTCGGAAGGCAGAGATTTGTCAGAAGCAGTTCACGTTCACCAGCGACTATACATCCAGGGACATAATAACAAAGAACTAGACCTGCAAATTCCTTGGCTGCCCCAAGTGAAGTCACTTGTCTCCTGTGGTACTGCGCCATCCATCTTAAAGTTTAAGGGTCTACATGTTATGGATTTAGGGGCCTGTGAATCTTTGCAGGCTAGTCATCTCAAGGGTATAAATAATGTAAGTTCTTTGAGATATCTGGTCATAGGAGGTAAGTGTATCTCTGGCATCCCTAAGGAAATTGCGAAGCTGGAACATTTGCGGACACTAGATTTAAGTGCAAGTGGTCTAAATGAATTGCCAGAATATGTTTTCATGATAAGAAAATTGGAACGCCTAATTGTTAATAGTCAGATGAAGATATCATATGGTATTGCAAAGATGTCTGCTTTACAGGAGCTAGGCGATATCAATGTCACCGACCCAGAGTTGCTGAAAAGTCTCTGTAAGCTAACCAAATTGAGGGTTCTTAGAATTTCCATATGGTCATGGGATGATAGTTTGAAGAACTATTTTAAACAACTGTGTGACAACTTGCGTTCACTGGTTCAGTGCACGGAGAACATCCAGAGTCTCTCCATAATGACATGCTGCTCCCTGGTTTTCATGGATGATTTGGGTGAGAATTGGACCCCTCAATGTCTCCAGAAGCTCGAGGTCGGTTGCAGCGCATTTGACATATTGCCAAGTTGGTTTGGCTCACTTTCTAGTATCTCCACGTTAACAATCGAGGTCTACAAGCTGTCACAGGACATAATTGATACGCTCGGAAGGCTGGCTGGTCTTGGTTCTCTATCCCTGACATCGAAACAAGTACCAAAAGGATACTTTGTGATCGGCTCTGACAGGTTCAATAAGCTACAGAGCTTAAAGTTTGTGAGCAATGCAATGGTAGAGATGTTTCCACGTCAACAATCAAATGGCACGGAACAGCTCAAAAGGCTTATGATTGTGTTCCATGCTTCACGTACACAAGATGTGAACAAAGATTTCTGCTTTGGTTTGGAGAACCTGTCTTCCCTAGAGCATGTTCGTGTTGAAATAATTTGTTTCGATGCCAGCCATAACATGGTGAAAAACGCAGAAGCTGCAGTTCAGAAAGCTATATCTGGCACAAGTATCGCAAATCTGGAAATACGAAGACTTCAGGAAAATAGTATGACACAGGACGAAGCGGACCTCTGTGATGCAGTACAAGAGCAGAATAATCAGAAGCACCAGAAAATGAAGAGTTCTATGGAGTCCCAGGATGGGTACCTCACTTCTTTACCGAACCAGGAAAGTGCAGATGTTGTTGGCTCCGATAGTATTGTGGAACCACTAGTGAATGAAATGAACTCGCAGACAATTAAGAGGGATCAATCCACGAATTTCAGTGAAGATGAGGACTTAATGTTGGTTTCTAGCTACCTTAATGTAAGCAAAGATTCTATTACTGGAAGGGATAAAAAAGAAGGCACATTTTGGGAAAGAGTATGGGAATACTATAACAAGAATAGGACATTCGAGTCCGATCATAGTTGGTCGTCATTGAAACATCGCTGGCTTGCAATTCAGAAGGAAGTGAATATCTTTCAAGGTTACTATGATGCCATAGAAAGGAAAAATCAAAGTGGCCAGACAAGTGATGACAAGCATGCTGAAGCAGAAGTAGAATTCCGAGAAAAACAAGGGAAGGCTTTTTCTGTATTCCATGTGTGGATGATTCTAAGGCATGAGCCAAAGTGGGCATTTAGAGAATCAAAGATCAAAGACCAGCATGAAGCAAACAATGCTAATACTGATGCTCCTGCCAACATTTATAGACCACAGGGGAGGAAAGCTGAGAAGGAAAAGGCTCGTGCGAGAAAGCATGGTGGATCTGATGTTGATGGTGATCCGTTCATCGAAGAAGTAAAAAATATGAGGGAAGCACGGGAAGAAACAGAACGAGACCGAAAGACCCATGATGACAAGTTCTATGAGTTGGAAAAGAGTAAGCTTGAATTGGAGCGAGATCGACATGACAAAGAGATAATGCAAACAGACACAAGCACAATGGATGAAGAATCGAAACAATACTTCAAGTTGATGAAACAAGAGATTTTGGCTCGCCGTTTCGGGAGTAGTCAGCCA

>B73_Zm00001eb291370

ATGCCGATTGGAGAGGTTGTGCTATCTGCCTTCACGCAGGCACTCTTTGAGAAAGTGCTTGCTGCTACTATCGGAGAGCTGAAACTCCCTCCAGATGTCACTGAAGAACTGCAAAGCTTATCGAGCATCCTGTCAACAATTCAATTTCATGTCGAAGATGCCGAGGAGCGGCAATTGAAGGATAAGGCTGCACGCAGCTGGCTTGCCAAGCTCAAGGATGTCGCGGATGAGATGGATGACTTGCTTGATGAGTATGCAGCCGAGACTCTGCGATCCAAACTAGAAGGTCCATCCAACCATGACCATCTGAAGAAGGTTAGGAGCTGTTTCTGCTGTTTTTGGTTGAACAAGTGTTTCTTTAATCATAAGATAGCGCAGCACATAAGGAAGATTGAGGGGAAACTCGATAGGCTTATCAAGGAAAGACAAATTATTGGTCCCAACATGAACAGTGGGACCGACAGGCAGGAGATAAAGGAGAGGCCCAAAACAAGTTCGCTGATCGATGACTCAAGTGTGTTTGGAAGAGAAAAAGATAAGGAAACCATTGTAAAGATGTTGCTGGCCCCTAATAATAACTCAGGCCATGCCAACCTTTCTATTATTCCCATAGTGGGCATGGGGGGACTAGGAAAGACGACTCTAACACAGCTCATCTACAATGATGAAAGAGTAAAGGAGCATTTCCAGTTAAGGGTGTGGTTGTGTGTTTCTGAAAATTTTGACGAGATGAAGCTTACCAAGGAAACAATTGAATCAGTTGCTAGTGGATTCTCATCAGCCACAACAAACATGAACCTGCTCCAAGAAGACCTCTCAAAAAAGCTGCAAGGTAAAAGATTTCTTCTAGTCCTTGATGATGTATGGAATGAGGATCCTGAAAAATGGGACAGATATCGTTGTGCTCTACTTAGCGGGGGAAAGGGAAGCAGGATTATAATTACCACGCGAAACAAAAATGTGGGGATACTAATGGGTGGGATGACTCCTTACCATCTAAAGCAGCTATCAAACGATGATTGCTGGCAGTTGTTCAAAAAACATGCATTTGTAGATGGTGACTCCAGTTCACACCCAGAATTAGAAATAATAGGCAAGGACATCGTGAAGAAGTTGAAAGGCCTGCCACTAGCTGCAAAAGCAGTCAGCAGTTTACTATGTACCAGGGATGCAGAGGAAGATTGGAAGAACATACTAAAGAGTGAAATATGGGAATTGCCATCAGACAAGAACAACATATTGCCAGCTCTGAGATTGAGTTACAGCCATTTGCCAGCCACACTGAAGCGATGTTTTGCATTTTGTTCAGTGTTTCCCAAAGATTACGTCTTTGAGAAAACAAGGTTGGTTCAAATATGGATGGCCCTTGGGTTCATTCAGCCTCAAGGAAGGAGAAAGATGGAAGAAATTGGGAGTGGCTATTTTGATGAATTGCAAAGCAGATCCTTCTTCCAACATCACAAAAGTGGATATGTCATGCATGATGCCATGCATGACCTAGCACAGTCTGTCTCAATTAATGAATGCCTTAGATTGGATGAAGGCCTAAGGCTGCATGATCCCCCGCACAGCAGCAGCCCTGCAACTGCAAGAAATGCCAGGCATCTATCATTCTCTTGTGACAACAGAAGCTGGACCCAGTTTGAAGCTTTTCTTGGATTTAAGAGAGCTCGCACACTTCTTCTACTAAATGGATACAAATCGATAACAAACTCTATACCCAGTGATCTGTTCATCAAGTTGAAGTACCTTCATGTGCTTGATCTGAACCGACGAGACATTACTGAGCTGCCTGATTCTATTGGTAACTTAAAATTGCTTCGATATTTGAATCTTTCAGGCACTGGAATAGAAATGTTGCCTTCATCAATCGGTAGGCTCTTCAGCCTGCAAACATTGAAGTTGCAAAACTGTCATTCACTAGATTACCTCCCAAAGACCATAACCAATCTCATAAATCTTCGATGGCTAGAAGCAAGGACGGAGTTGATCAATGGCATAGCTGGAATAGGGAACTTGACTTGCCTTCAACAGCTGGAGGAATTTGTTGTCCGTAAGGACAAAGGATACAAGATCAGTGAATTGAAGGCAATCAACGGGATCACAGGACAAATCTGCATTAAGAATCTTGAGAGCGTGGCAAGTGTGGAAGAGGCTGATGAAGCTTTGCTAACAAAGAAGACAAACATCAACAATTTACACCTTATATGGTCCGAAAGTAGGCGTTTGACTTCAAAAACAGCAGATAAAGACATGAAGATACTTGAACACCTCCAGCCACATCATGAACTCAGTGAGCTGTCAGTCAAGGCCTTTGCAGGCATATACTTTCCAAATTGGTTAAGTAAACTAACTCAATTGCAAACCATCCACCTGTCTGACTGTACAAACTGTTCAGTTCTACCAGCACTTGGAGTACTTCCCCTACTCAAGTTTTTAGATTTCGGGGGTTTCCATGCCATTGTTCACATCAACCAAGAGTTTTCAGGAACCAGTGAGGTCAAGAGGTTTCCATCACTGAAGGAACTGGTATTTGAAGACATGTCTAACCTAAAAGGTTGGACTTCTGTACAAGATGGTCAGTTGCTTCCATTGCTCACAGAGCTTGCAGTGATTGACTGCCCACTACTAGAAGAATTTCCATCTTTCCCATCATCAGTAGTGAAGCTCAAAATTTCTGAAACAGGGTTCGCTATTCTTCCAGAAATATATACTCCAAGTTCTCAAGTTTCATCATCATTGGTATGCCTAGAAATTCACCAGTGCCCAAATCTTACATCATTAGAGCGAGGACTGCTTTGCCAGAAATTATCAATGCTCCAGCAATTAACCATCACCGGTTGCCCAGAATTAACTCATCTGCCAGTTGAAGGATTCAGAGCCCTGACTGCTCTTAAGAGTATTCATATCTATGATTGTCCAAAGCTGGAACCATCCCAACAGCATAGCTTGCTGCCCTCCATGCTTGAAGATTTACGCATCAGCTCATGCTCCAATCTAATCAATCCTCTTCTTCGAGAGATTGATGGGATATTCTCAATGACAAATCTTGCCATAACTGATTGTGCCAGCCTTCGATATTTTCCAGTAAAGCTTCCTGCCACTCTGAAAAAGTTGGAGATCTTCCATTGTAGCAACCTAAGATGCTTGCCTCCTGGCATAGAAGCGACATCTTGTTTGGCAGCTATGACAATTTTGAAATGTCCTCTTATACCGAGCTTGCCAGAACAGGGCCTCCCACAATCACTGAAAGAATTGTACATCAAAGAATGCCCGCTGCTAACAAAGAGCTGTAAAGAAAATGATGGTGAAGATTGGCCTAAAATTGCTCATGTACCAACCATAGAGATTGAAGATGATAGTACCATGACCGACTGGAGCATAAGAAGATCATTCTATACACATCATAGTGACACAGGAGATCATCACTGA

>B73_Zm00001eb304860

ATGGACAACAATGGAGAGAAGACGACGACCATCATCCACACTCTGCGAGATGCGCTGCTTCACTTTGCAGTCAAGTCCAAGAAGCTCGCGTCGCCACTGCTGGAGCCATTCGGGCGGGCGACCGAGCCGACCACCGTCAACGACGACGAGCTGATGGCGCTCAAGTCCAAACTGCGGCGGATCCGCGCCACCCTCCGCGACGCCGAGAGCCTGTCCGTCACCGATTGCTCCGTCCAGCTGTGGCTGGCCGAGCTCGGCGACCTCGAGAACCGGGCCGAGGATGTGGTCGAGGAGCTGGAGTACGAGTCCCGCCGCTCGGCGCAGCTGGAGGAGCTCAAGCAAGACTTGCTCTACGCCGCCACGACGCGGAAGCAGCGCCGGGAGGTGGCGCTGCTGTTCGCGCCCCCGCCCGCGAGGCGGCTCCGCCGCAAGATCGACGACGTCTGGGCGAGGTACGAAGAGATCGCGTCGGACAGGAAGACGCTCCGGCTGCGGCCAGGCGACGGCGGCTGCGCGCCCCGGCCCGCGGCCAGCCCGCTCGTGCCAAGCAGCGTGCTTCCCCGTACCGAGCGCCTCCACGGGAGGCATGGCGACGTCGAGAGGGTCGCCGCGCTGGTCCTCGGGGATCCGGACGGCGGGACGAGCTACGCCGTCGTGCCCATCGTCGGGATGGCCGGCGTCGGCAAGACTGCTCTGATGCAGCACGTCTGCGGCATGGAGACGGTGAAGTCGTGCTTTGAACTGACGCGCTGGGTTTGGGTCTCCCAGGATTTTGACGTTGTCAGCGTGACCCGCAAGATTGTTGAGGCGATCACCAGATCGCGCCCGGAGTGCGGCGAGCTGAGCACGCTTCATGAGCTCATAGTTGAGCACCTTGCCGGGAAGAGGTGCTTGATCGTTCTTGACGACGTGTGGGATGACAATCCCAGCCACTGGAACAGCCTGACGGCCCCGCTGAGCCACTGCGCGCCAGGGAGTGCGGTTGCCGTGACGACGAGGAGCAACAAGGTTGCCAGGATGGTGAGCACCAAGGTGTATCATCTCAAATGCTTGTCAGATGAAGACTGCTGGCTTGTATGCCAGCGACGGGCACTGCCAAATAGCGGTGCCAACGTCCACAAAGAACTCGTTGAGATCGGTGAGAGGATCGCCAAGAAATGCCACGGCTTGCCATTGGCGGCAGAGGCAGCTGGTAGCGTCCTGAGCACTTCAGCCGTCTGGGAGCACTGGAATGAAGTCCTGAATAACGACTTGTGGGCTGACAATGAGGTGAAGAACCTGGTACTGCCGGTGCTGAAGGTGAGCTACGACCACCTGTCCATGCCGCTGAAGCGCAGCTTCGCGTTTTGTTCATTGTTTCCAAAGGGCTTCGTGTTCGACAAAGATCTGCTAGTCCAGCTGTGGACTGCGCAGGGCTTTGTGGATGCTGAAGGAGACTGCAGCCTTGAAGCGATCGCCAATGGTTACTTCAATGACTTGGTGTCAAGGTGCTTCTTCCACCCTTCCCCATCTCATGCTCTCAGCGAAGGGAAGTTTGTTATGCACGACCTGTATCAAGAGCTTGCTCAGTTTGTTTCAGGCAATGAATGTAGGATGATACAGCTCCCTAATTCGACGAAAATAGATGAGAGCTCTCGGCATTTGTCCTTGGTCGACGAGGAGTCCGATTCGGTCGAAGAAATAAACCTGAGCTGGTTCTGTGGTCATCGTGATCTCCGGACCTTCATGTTCATTGCAAGAACAGAACAGAACCCCGAGGAGATGACCTTCAGAACAAAGATTCCATCTGAGCTGATCACAGGTTTTGAATGCTTAAGGGCTTTAGATTTGAGCAACTCTAATATCATGGAGCTACCGAAATCCATTGGAAGTCTGATACACCTAAGGTTCCTTGGTCTGGACAACACCGCAATTCAGATGCTGCCTGAGTCAATTTGTGCTCTTCTCCACTTGCAGACAATAAAGCTTAACCATTGTTCTTCCCTTACTCAGTTGCCTCAGGGCATCAAGCTCCTATTGAACCTAAGGTGCTTAGAGATTCCACATTCAGGCATAAAGATGCCTTCTGGGATTGGAGAGTTGACTAGGCTGCAGAGACTACCTTTTTTCGCCATCGAGAATGAGCCTGCTGGATGCACCATAGCAGACCTCAACGAACTGGTAAACCTCGAAGGACATCTTCACATCACAGGTTTAAACAACCTGGATGGTGCACAAGCTTCCATCGCCAACCTTTGGAACAAGCCGCGGATTAAAAGTCTTACACTCGAATGGTCCGGAGTTACAAATTTTAGCAAGTCCCTTTGTGATCCGCAAGGAAACGCTGTGAGCTGCATATCAGACAGTCAGCACCCTGCAATCAGTGCAACAGCAGATCAGGTTTTGAATTGCCTCAAGCCACATTCAAATCTGGAGGAGCTTAGCATCAAGGGTTATAACGGATCCTTTTCTCGGTCATGGTTAGGATGGCTGCCCTTGGACAGGTTAGCTTCTATTGAACTGAAAGACTGTCGTAATTGCAAAGAAGTACCGCCCCTTGGCTGCCTACCATCACTGAAACACATTTTGATACAATCACTGCCAAGTGTGAAGCTGATTGGTCCAGAGTTCTTTGGAAATGCTGGAGATACCACTTCTAACATTAGGAGCAGAATCTGTAATGTGTTTCCTGCACTGAAGTCACTAAAGTTCAGCAACATGGAAGCTTGGGAGGAATGGCTCGGTGTCAAGAGTGAGCACTTCCCCAATCTTAAATATTTCAGCATCGTCAGATGCAGCAAACTGAAGCTGTTGCCCAAGTTCACTTCAGAACCAAAGCTGAAAATACGGTACTGTGACCTGCTGCAAATGCCTTTGTGTCAGAATCCGGTGAAGCACATACCAGCTAAAAAGGAAATCTCATATACGTGCATTGCGGAAGGTGACATTTTAGTTCTTGAAGCTTCCTGTTCCTATGGTGCATAG

>B73_Zm00001eb310010

ATGGTCGGCCCCGAGATGCTCGTCGCCGCGACGGTGAACCAGGTCGTTCGGAAGATCAACGAGATCATCGGCGTCGCGCAGGGCGAGACGAAGCTGTGCTGCAGCTTCAGCGACGACCTCGAGGGCATCAAGGACACCCTGGTATACCTTGAAGGCCTCCTCAAGAACGCGGAGAGCAACTCCTTTGGGAGCGATAGGGCGAACCTGCGGCACTGGCTGGGCCAGATCAAGTCTCTGGCTTACGATATCGAAGACATTGTTGATGGCTACTACTCCTCCAAGGAGCAATATGAGGGAAGCAACTATGCTCAGAAGGGATCTTTACTCTGCTCTTTATCCAATCCCATGCTTTCGAAGGTTAGCATGGTTCATAAGATGAAATCCAAGAGGGAGTTGTTACAGACAAGGCAAAATTTACCGACCCAGTATCATTTCATCTCACATATTAACTCAGTGGTAAATTATGATGAGAAGCAGACAACATCATACAGAAATTGTGACATCAGAATTGTTGGGAGGGGTCCAGACTTGGAGCATCTTATGTACATGTTAATGCAGATAAATTTGAAAGAGCTTACCATTATTTCCATAGTTGGGCCTGTGGGCCTTGGGAAGACAACCCTTGCACAACTTATTTTCAATGATGCAAGAGCTGAGACATTCAGATTCAGAATATGGGTTCATGTCTCCATGGGCAATGTCAGCCTCCAAAAAATTGGGAGAGATATAGTTTTGCAAACTACAGAAAAAATTGAGGGAAACATGCAGATGCAGTCAATCAAGAATGTTGTTCAAGACATACTTAATAAGTATAGTTGCTTAATAGTGTTAGATAGCTTGTGGGGAAAAGATGAAGAAGTGAATGAGCTGAAGCAGATGTTGCTTACAGGTAGAAAGACTGAAAGCAAGGTCATAGTGACCACTCACAGCAGTAAAGTAGCAGAGCTGATATCCACTGTGCCACCATACAAACTGTCTTTGTTGTCAGAAGATGATTGTTCAGCTATATTCTGTCAAAGGGCAATTGCAGGTCAGTCTATCAGTGACCCACTTTTCAGGGAATATGGAGAAGAAATTGTTAGGAGATGTGAAGGCATGCCCTTGGTAGCCAATTTTCTTGGATCTGTGGTGAATGCTCAAAGACAGAGGCGTGAGATATGGAAAGCTGCAAGGGATAAAGACATGTGGAAGATAGAGGAAGACTACCCTGAAAACAAAATTGTGCCAATGTTTCCATCTTTCAAGATAGTATACTACAGTATGCCCCATGAGCTAAGGTTATGCTTTGTCTACTGTTCAATCTTTCCTAAAGGATATGTCATAGATAAGAAAAAACTTATTCAGCAATGGATTGCACTTGACATGATTGAATCCAAACATGGAACCTTGCCGCTTGATGTTACTGCTGAGAAATACATTGATGAACTTAAAGATATTTATCTCCTTCAAGTTTTAGAGAGGCATCAGATTGATGCAGAAATATCCAATACTTCTGAAGAAAAGCTCTACATGAATAATTTGGCACATGATCTTGCTAGGTCAGTTGCTGGTGAAGATATCCTTGTGATCTTAGATGCTGAGAATGTACGCTGCAATAGAAATTATGATTACCGTTATGCACAAGTGTCAACTTCTAGCTTACAATCAATAGATAGCAAGGCTTGGCCTTCCAAGGCAAGGTCACTAGTTTTCAAGTCAAGTGCAGAATTGCAGCATGTCAGCGAAGTTCTTTCAGTGAACAAATATTTGCGTGTTTTGGATATCAGTGGGTGTTCTGTGAAGGAGATGCCCGCTCCAATTTTTCAGATGAAACAACTGAGATATCTTGATGCTTCAACATTGTCCATTGCAGATCTGCCTCCCCAAATTAGTGGCTTTCCTAAGCTACAGACACTGGATCTGTCTGATACTGAAGTAACTGAGCTACCAGCCTTCATTGCCAACTTAAAGAGACTGAATTATTTGAATCTCCAAGGTTGCAAGAAACTTAAGCAGTTGAATAACCTAGATTTGCTGCATGAGTTACATTACCTAAACTTGTCACGTTGCCTGGAAGTTAGAAGTTTTCCCGCATCTCTGAAAAATCTTAGGAAACTTCGTTTTCTGAACCTTTCGCAATGTTCTAAGCTTCCAACATTACCTGATGAATTATTGCAATCATTCTCTAGTTTTTCTTCCATTGTGGATTTAAACTTAAGTGGATTTGAGTTCCAAATGCTACCTGACTTTTTTGGCAACATTTGTTCACTTCAGTTTTTGAGTCTGTCAAAATGCTCAAAACTTGAGCTACTCCCCCAATCATTTGGTCAGCTTGCATATTTGAAAGGTCTGGACCTTTCATTTTGTTCTGATCTTAAACTCCCAGAGTCCTTTAAGTACCTTTCTTCTCTTCAGTTTCTAAATCTATCACACTGCCATAATGTAGAATATCTGCCATCTTTTGACAAACTTAGTAATTTGGAGTATCTTAATTTATCACAATGTGCTGGACTCAAAGCATTACCTAAGTCACTTTCAAACCAAAAAAATCTCCAAATTGAGGTTTTTGGGTGCCAGGATTGCATAGTGCAGTCATGTTATTTAAGCCCACAGTCCCATACATGCTCACAAGAAGTCAAGGAAGTTGGATCAAGTAGTGATATCTCAGATACCATTCTAAAGGAGTCTGCAAACAGAGATGTGGCATTAGGAATAAGTTTTCCAGAAATTGATGAAGTTGGTTATCCACATAATAATCTGAAACAAAAATTGACATTATCTTATCATATGGATGGACATAAAAGTGAAGAGCCTAATTTCATTACCAAGCAGAACTCAACTACAGAGACGGTACAACTAACCCCCAGGCACCAGTTCCCATCATCTACATGTCGTATTTCTTCGATTGCATCAAGTTCTAGTGCACTATTTGCATCTGGCTCCTCTTCAGATGTCTCGATAACTTACAATCCAGTGTCCAATGACAAAGCAGTCCCCGTGTACCACATCTTGAAGCCTGTACCTTCCACTTCCTTCGCCTTCTGTCCTTTCCACTTGGTCTCTTGA

>B73_Zm00001eb318600

ATGGAGGCGACGGCCGTGAGCTTGGCGAGGTCCGTTCTAGACGGCGTCTTAAGCAGCGCCGGTTCTTCCCTCGCCGATGAGGTCGCGCGCCTCATTGGGGTCCCGAAGGAGGTAGACTTCATCCGCAACGAGCTAGAGATGATGCAGGCCTTCCTGAAGGTGGCGTCCACGGTGTATCCCGAGGCCACCGTGCGCAACGACATCGTGAAGACGTGGGTGAAGCAAGTGCGGTACCTCGCGTGCGACGTCGAGGACTGCCTGCTCGACTTCGCGCTCTACGCCGACAGGAGGTCGTCCTCGCGGCTCGGCGCCTGGCTCCCCAGCGCCATCGCCGAGCGCCGCCGCATCGCCGGGCGGATCCGGGACCTCAAGGCCAGCGTCGAGGCGCTGAACCAGCGCAACCAGAGGTACCAGATCGCCGCCGGCCCCGGCGCTGGCGCCCATCATCATCAGTCGCCCGCGCTGCACGAGCACTACGAGCACTCGGCCGAGCTGGCGTTCCAGGTGTCGGACATCATCGGCCGGGACGACGACATTAACGCGGTCGCCAAGATGCTCCTCTCACGCAACAACGGCGCGCTGCGCGTCCTGTCCGTGTGGGGGATGGGCGGCATGGGGAAGTCGTCGCTGGCGCGCATGGTACACAACGACTCGGACGTCCTCGCCGAGTTCGACTGCCGCGCGTGGATCACGGTGCCGCACCCGCTGGACAACCCCGACGTGTTCAAGCGCCGGCTAAAGGAGGAGCTCGCCGTGCCAAACGGCCTGAAGATCGAGGATCACCTAGGGGATAAGCGATACCTGGTCGTCGTGGACGATCTACTTAGCCAGGACGAGTGGGAAAATGTATGGCAGGCTTTTCAGTTCAGCAACAAAATGGACAGCCGTATCATCGTCACGACGCGGCTGAAACTGAAAGACGTCGCCCGGCACTGCGCGGGACCGAGGAATGTGGCCGAGGAGCTTATCTACGACTACGAGCTCAAGCCACTGGATAGTAAGGAGTCTAAGCGACTCCTCTGCCAGAAGGTCTTCAAGACGACTGATTACAATTTACCATCGGATATGGAGGATCAAGCCAGACATATCTTGAGGAGATGTTGTGGGCTTCCGCTTGCCATATCTACCATCGGAGGTCTCCTAGCCAACAGGCCGAGAACAAGCATAGAATGGAGGAACCTGCATGAACATCTGGGGGCAGAGTTGGAGACTGACCTCAACAATATCACCAAGGTGATCATGTCAAGTTATGATGGCTTGCCGTATCACTTGAAACCCATCTTCTTGTACCTCAGTATCTTCCCTGAGAACCATGAGATCAGACGCACCCGCTTGCTCAGGCGGTGGATGGCTGAAGGCTACATAGCAAAGAAACGTGACATGCCTGTAGAGGTTGTAGGAGAGCATTTCTACAATGATCTCATTAACAGAAGCATGATTCAAGGTTCCATGGCCAGCTATGCAGTAAGAGCTGATCGTTGCTCGGTTCACAGCATGGTGCGCAAGATCATCTTGTCCAAGTTCATCGAGGAGAACCAACTATTGCTCATCGAGAAGCACTCCAATGAGATTCCACAAAGTAAGATACGCCACCTGGTGGTATCTAGATGGAAAGGGAGGGATGAAGAGCTTCGAAGAATTAACTTGTCATATGTCCGGTCGTTGACAATCTTTGGGGATTACCCTGCATCTCTCGTGTCTCCAAAACTGCGGCTGTTGCGTGTGCTTGACCTGGAGGACACGATCAATCTGAAGAACAGTGATCTCAAGCATATAGGCGAGCTAAGACACCTAAGATATATATGCCTGCGAGGAACGAACATTTCTAAGCTTCCGTCTTCCTTGCAGAACCTTCGATATCTGGAGACATTAGACATCCAAGACACACAGGTGACACAACTCCCCAGGGGCATTTCCAAACTTGAGAAGCTCTGTTATCTTCTAGTTGGTGTCAACTTTTCGAAAGATTTGCTGGGAAAGATGGAATTAGTGAAGAACCATAAAGCCAGCCTTTTAACCTGTCTATGCTGCAACAAAGGTGAGTGCTGCAAGGTTTTCAACTCAAGTGTAAGAGCTCCCGAAGGAATCGAGAAGTTACAGAACCTGCACATGTTGGGGGTCGTCAATGTTGGCAATGGCAATGTACCAGCACATCTTGTTTCCCTGAGGCTGTGCGGGAATCTGAGCAGTCTACCTGATTGGATCGGTTCACTCAATGATCTAGGTAAGGTGAAGCTGCTGGGGACACAACTGAAGCACAAAGACATCGTGCACCTTCAGAACCTACGCAACCTGACTTTGCTAGGCCTATGGGAGAACTCCTACATAGGGGACTCCTTGCATTTCTGCACTGGTACATTTCTAAAGCTCAAGTTCCTTGACATTGATGGGCTAGAGAAAATAGAGAAAGTGTCTGTTGAGAAAGGTGCCATGCCTGAGCTTAAGAAACTTTGGGTCAACAAGTGCCCAAAACTGCAGGACAACAGTTCTGGCTTGTTTGCGGTGCCACACCTACCGATCCTGAATGAGCTTCTTCTTAAGAAATGTGGCGATAAAGAGAACCTGACAAAGATACTGCAAAGGCAGATTAGTGATCACCATAAACGCCCTAGGTTCCTCATTGACAAGTCAATTATGCCTACTAGCCCATACGCACAATGA

>B73_Zm00001eb349330

ATGCCTGCCCCCGCGCCGTGGCCCGTGTCGCAGGACGTCGCGGCGCTCGCCGATCGCGCCAGCGCCTTCTCCCGTGCCGTCGTCGGGCCCCGCAACGACCTGGGTGCTCTCGCGGCGGCGCTCCTTCGGATCCAACCCGTAGCCCGCGAGCTCGAGCGCTGGAGGTGGCTCACGCCCGGCGACCCTGAGGTGCCCGACCTGCACGCCTGGCTCTTCGAGCTCAGGGACGCGGTGGCCGACGCCGAGAGCATCCTAGATGAGCTCCACCGCCGGCGCCAGATCGGCCCTCCGCTCTCGGCCTGCGTATACGCCACGTTCCGTGGCCCCGGACGGAAGCTGAGGCGGCTGACGGAGAGGCTCAACCGCGCCCGCGACGATTCGGAGCGTCTGCGACCTGGCAGCGCTGCGGCCATCTACGGTGTGCGGTCGCCGAACAGGGTAACCGGCTCCGTTTTCGCGGAGCGGAAGGTACTCGGGCGTGGGGAGGAGTGCAACTCCATAATCTCCAGGCTCGTCGACGATTGCGAGGAAACATGCCGTCCGGTATCTCCGGTTATTGCAGTGGTTGGCCATGGAGGCATCGGCAAAACCACGGTCGCTCAGTGCGTGTACAACGATGCGAGGGTTGAAGCGCGGTTCGATCTGAGAGCCTGGGTCTGCGTCTGGGACAGATCAGATGAGGCCGAGCTCACCAGGGAGATTTTACAGTCCATTGGTTGTGCAGATGATAAACCGTGTGATGACGGTCTTGCCAGTTTAGACAGTTTGCAGGAGAAGTTTGAGAATCTGGTTGCACGTAAGAGGTTCCTCCTGGTACTTGACGATGTTTGGATTGATGAGGGTAAAACCGAGAAGGAGAATAGGAGCATTTGGAACAGAGTGTTGGTGCCACTTAGATCTGCCACAACTGGGAGCAAGGTCTTGTTGACCACTCGGATGAAACTAGTAGCTGAGGTTCTGAATGCGGGTTATTTGGTTTCGCTTGATGGATTAAGAAGCAGTGATTGTTGGTTGCTGTTGAAGGAAGTTGCTTTGGGTGGAGAAACCATGGATTTTCCACCTGAGTTGCAAGAGGTTGCAGGAACTCTTGTTGCAATGGTTAAGGGCTCACCTTTAGCTGCTAAAGCTATTGGGCAAATGGCAAGGAGCACCAGGAGCACACGGAAATGGAGAACCCTGGTGAACACAGAAATTAGTAATGATATCATTATCTCTTCCATCCAACTCAGTTACAAACACTTGCCGGGTCATCTCCAGCGCTGCTTTGCATACTGCAGCATATTTCCATCAACTTGGAGGTTCAGTCGCTCTCAGTTGGTCAATATGTGGATAGCTCTCGGTTTCATTCAATCATCAGCAGAAGGGAAAGGGCTGGAAGAATTAGGGCAAAAATACTTTGATGATCTCCTATCACGATCCTTCTTTGGTACTGCAAATAAAGACCAGCAAACATATTACTTCCTTGATGACCTAATGCACATTTTGGCACAGCACTTCTCTGCTCATGATTGTATGAAAATCAACGAGGGCATCCCTGTTGTGATTCCACCAACAGTTCGTCATCTGTCTGTTTCAACCGATTATTTACCACAGCTGAAGAGCAAATACAGGTTGGGAAGGCTGCAAACATTGTTAGTCCTCAGAAGCTCATCGTTATCTTCAGGTCATTTCCCTAGAAAACTTCTGGCCAAATTTAAGAACTTGCGGGTCCTGGATCTGAGTGGATCTGATATTGTAGAATTGCCAGAAAGCATTAATCAGTTGGTACATCTTCATTACCTAGCTATATGCAGTATAACTAACAAACTTCCCAAGACTATATACATGCTTAAACATCTTGAGGTGTGTGATATACCTATATTCTTATTTCATGATGATCATCCTAGAGGTGTTGGTAAATCTGTTACAGTTAAACGTCTCAAAACATGCTGTGGCAAGCAGGCAAGTAATCCCAGCAGCAGGCATGAAGTGCAATTTTTCTTTTCAATTCGACACCTTCAAGCTCTAGAATCCATTTCTACTCAGCCTGTTTCTGTGATTTGGTGTCAGAATAACTCCAGTTTGGTGGATCAAGAATCTGGTTGTTCACATCCATCAAAACTTCAAACTATTTTGTGTTGGTTTGTTTGA

>B73_Zm00001eb363970

ATGGCGGAGACGGCGATCACGACGGTGCTGGCCAAAGTGGCGGAGCTGGTGGCGTGGGAGGCGGCTGTGCTGCTGGAGGTGGGCGACGACGTGCGCCTCCTCCGGGACAAGCTCGAGTGGCTCCACACCTTCATCCGCGACGCCGACCGCCGCCGCCGCCGCCGCGACGACGAGTTCGTCGCCGTCTGGGTGCGCCAGACCCGCGACGTCGCCTTCGAGGCCGAGGACGCGCTGGACGACTTCCTACACCGCGCTGGGCGGAGGAAGGCCGCGCTGGGCTCCCGGTGCGCGCTCGGGTGCTGGTGGCCCGGCGGGTGCGCCGGCCAGGTCGCGCTCCGCCACGACCTCTCGGGGCGTATCCGCCAGATCAGGAAGCGGCTCGATAAGATCTCCGAGAACCGCGCCGACTACAACATCGAGCACACGCCCGCGCCGGCCTGGGCCGCTTGCTCCTCCTCCGCCACCACCCTCGCTGCCTGGGACGATCTAGAGGAGTACACGGTCGGCCTGGACAAGTACAGCGACATGCTTAAGGAGCAGCTCCTCGACGACTCCGTCCCCGCGCGCGCGCTCGTGGCCATCGCCGGCGAGAGCAGCATTGGCAAGACGACGCTCGCGCGGAAGGTGTACCAGAGCCTCGAGGTCCGCAACCACTTCGAGATCCGCACGTGGACGGTGCTCCCGCACAAGTGCCGCGCCGCCGACGTTCTCCGCGACATCCACGAGCAGATGACCTCCCAGCTGCGGCGGACCCCGTCCGCGTCCAACTCCAAGCAGGCCGTGGAAGACGCCTGCGATGACAAAGCCTTCGGGCCGGGAAAGGACATCAGCAACCAGCTGTACAGGAGCATGACGGGGAGGCGGTACCTCGTGGTCATCGACGGCAGCGTTGCCGTCACGGACTGGAACAGCCTACGAGCTTCCCTCCCCGACGAGGGCAACGGCAGCAGGGTGCTTCTGATCACGGACTTGGAAGGCCTGGAGGTGGTCGGCCACGCTCAGGCTGGCCACACGTACGACCCGATCGAGCTGACCCGGCTGAGCCCGGAGAGCACGTACGAGGTGTTCCGACGCCGGGTGTTCGGCGCCCGCGGCGACTGCCCCGGCCGGTACAAGTCGAGGTACTACCAAGACGTGTTCCGGATCACGCGTGGCCTGCCGCTGTCGATCGTCGTCCTCGCCGGTATCCTGCGGTCCAAGGAGCTGCCCGCAGAGTGGGACGAGGTGATGGCGCAGCTGGCCCCGCCGGCGCGCGAGCAGCAAAGGGGCGGCAGCGGCGGCAGCAGCAACAGCTGGCCCCGGATCATGTCGCGGGCCTTCGACGACCTGCCGCACCACCTCAAGTCGTGCTTCCTATACCTGGCGGCGATGCGGGAGAGCACCCCGGTGGACGCGCAGCGCCTGGTGCGCCTGTGGGTGGCCGAGGGGTTCGTGCGCCCGCGCCGCGGGAGCACCATGGAGGAGGTCGGGCAGGGGTACCTCAAGGAGCTCATCTCCCGGTGCATGGTGCAGCTGGTGGACAAGGACGACTTCGGCGCCGTGCTGACGGTGGTGGTGCACGACCGGCTGCACGCATTCGCGCAGGACGAGGCACAGGAGGCCAGCTTCATCGAGAGCCACGACAGCACAGACGTGCTCGCGCCGGCCACCGTGCGGCGCCTCGCCGTCCTCAACTCCACCACGGACAGGTACGTCCAGCTCAGCAACGCGCTCCCCAAGCTGCGCTCCATCATCTGCGACCTGGTGGAGGGTCGCCGAGTCAGGTCCAGCAACTTCATCCGCACCAGCGACCTGAGCTTCCTCCATGCTTCCAAGTTCCTCCGTGTCATCGACATCCAGGGACTCGAGCTCAAGAGGCTCCCAAACGAGATCGGCTCCATGATCCATTTGAGGTACCTGGGTCTCCGCTGCGGCCACCTAGAGAAGCTCCCAAGCACCATTGGCAACCTTGTCAACCTGCAGTCGCTCATCCTCGGCGGCCGCCACGTTCTGGAGGTGCCCGCCGCGTTCTGGAGGATCGCCACGCTGCGGCACGTCGTCGCGCGGTTCGCTCTGCCGAGCAGGGCCCTGGGCAACCTGCACAGCCTCCAGACGCTACATGGCGTGCAGCCCCGCGGGTGGGGCGGCGACTACAACCCGCTGGGGAAGGCCGCCAACCTCCGGTCGCTGGAGCTGGGCGAGCTGACCTCCGAGCACGCCGATGCGCTCGAGGCCGCGTTGGAGAACCTCGACCTCCTGGAGCACCTGGCGCTGCGGGGCGACCCGCTGCCGTCGAGCGTGTTCAGCGTCCCGAGCCTCCGCCGGCTGCAGAGCCTGAGGCTGATGGGCGCCATGGATGAGCCGGAGGGGCCCAGCTGCGCGGAGGACGTCCGGTACATCCGGCCGAACCTGACCAGGCTATCGATGTGGAATACCGAGGTGGGGCAGAAGTTCGTGGACATGCTGGCCGAGCTGCCGAGCCTGGCCGAGCTGACGATGATGTACGACTCATACGACGGCGATCGGCTGGCGTTCGTGGAGACTGGGTTCCCGAGCCTGCAGAAGCTAAAGCTCGGTCTACCGGAGCTGGAGGAGTGGACGGTGGCCCCGGGGTCCATGCCGGGGCTCGGCACGCTGACGCTGTGCCGGTGCGCCAGGATGCAGATGCTCCCGGAGGCGCTCGCCGGGATGAGGGAGCTGGAGGAGGTGGTGCTGTACAGCATGCCCGACATCGTGAGCAGGATCAAGGAGGACGAAGGCCAGGATTACCACAAAGTCAAGCACGTACCCGTCATCCAGACTATATACTGA

>B73_Zm00001eb378630

ATGGCCCAACAGGCAGTTTTTGCGGTGCTGGAGCGGGGTGGTAGCATTGCAGTTGATGAGGCCGTATACTTGTTGGGCGTCTCTGAAAAGCTGGAATCAGCCAAGAAGCAACTCCTACTCATGCAAGCCTTTCTCATGGACCTGGATGAAAAGATGCTGAAGGGTAACTTCATGGCTAGGCATCTAGCTTCCGAGGTTCGGGAGATCGCATATGAGGTGGAGGACATCATTGACACCGCCAACATCTTGATGAGGAGAAACGGCCCCAAGACATCAGTCAGAGGGGCCATGTCCAAATATGCTTGCTTCCCCATATACCTCACTCGTCTCCACAAGCTGGGATCAAGGATAGATTCAACTGAAGAAAGGATGAAAAAACTTTTCGGCGACTTTGAGAAATTCAACATCGCTGCTAATGCAATTGCAGAAGAACCACGGCGTTACATCACCGAGGATGATGACATTCGGCATAGGCGATTGGTTCATCCCAACTCGGGTGATCAAGTTGGTGTCATTGGGTTTGATGAGCAGATTAAACAGATCGAGTATGACTTGCTAGACACAAAGAACAGGCACCTAACCGTTGTTTCCATTGTAGGTCCCGGTGGGGCAGGCAAATCAACAATGGCTAAGAAGGTGTATAGTTTGCCTGCAGTGAAGGGACACTTTAAAGTTCATTGTTGGTTAACTGTGTCCCAACGAGCTGTTGCAACTCATGATTTTTTGAAAGAGGTGGTTAAGATGGTTGTGCCTTCTCACCTCATGAAAGTTATGGTTCTGCGTGTGATGGGGGATGTCAAGGTCAAGAAAGTCGACAACGCTAGAAAGATGATGACCGAGAAGGAGGAGAAGAAAATTTGGGAAGACCAGAAAGCTAAAGAGCTCGAGGAAGCTAAAGAGCTTGATAAGCTGGAGGAGCATGAGGTGAAAAAGCTGCTTCACGAGTTTGCACTGAGTCAAAGGTATTTGATAGTGTTGGATGATATATGGAGCAAAGATGCCTGGGATGCTATCAAGCATGCCTTCCCTAATCAGAAGAATGGCAGCAGAATCATTCTGACCACACGCAATGTGGATGTTGCAAAGCTTCCCGGTGCAAGGAAGAAAATCTACAGGCCAAAGCTCCTGAATGAAGATGAAAGCACCCAGTTGCTTCTTACCACGGCCCTCCCAGAGTACATCTTGGATGGCGGGCAAAACTTGGACGAGTTGAAGGAGCTGGGCAAAGAGCTTGCAATAAAATGTGGTGGCCTGCCTCTTGCTTTGATTGTTTTGGGGGGATACCTGTCAAGGAATCTTGATGTTGGCGAGTGGAAGAGGTTGTTAACAAACAGTATGGATTGGCATGATTTGATCACCTCCGATAGGGTCATTGGTGCCATACTGGATCTTAGTTACTATGACATGCCGAGTCATCTGAGATCATGCTTCATGTACACCACGGCCTTCCCTGAAGACTCTCCTATTGATGTGCGAGTTCTGGCAATGCTATGGATTGCTGAGGGTTTCATCCCGCTGGTCCGAGGCCAGACCCGCGAAAAAGTAGCCTTAAAGTATGTGGCTGAGCTAGTGCAGCGGTGCATGATCCAAGCGGAGGGATGGACGAACTCTGGGATGATCAAGGTGGTCAAGGTGCATGACATCCTGCGCGAGTGGGGGTTTGGACGAGCTCAGAGAGAAGGGTTTATGAAAGATTGCCATTCTGCGGAGGATATCGAGGTGGCCTACTCGGGGGAGGAGATGATGAAAGCTTACCGAGTGGTGCTCCACAGCTCACTGTCACTGGAAAGGGGAGTTGGAACCACCACGAGAAAGCTTCGCACTCTATTAGACTTCAACAACCACACATCAGTACAGGTGCCAAAATCGTTCCAGGGTTTACGCGTGCTTCACCTGAACTGTTCTGGCGAGGTTTCTCTGCCAAAGGATATTCACCAGATGAGGTATTTGAGATATCTTGGCTTGGGAGGCAACTGCTCCTATGATCTCCCTTCCAACATTGGAGGCCTCTTAAGCCTCGAAACACTGTACTGCACAGCAAGTATAGACCACATCCCAGCATCATTGTGGCGGAACAGGACACTGAGGCAAGTGCATATCCCCTATGCTAGAAGCTTATCATCACCACAGATCGGTTCACAGTCATCAAAGGTTCTTGTTATACTAGTAGATTGTGGGAGCAGTACCCATATGGACGATGCTAAGCGGATTGTGGAGAAAACTAGAAGGCAAGTGCTGAGGAACAAGAACCTGGATCTCTCGTTCTGCTTGGGGGTGGAGTATGGGAAATATGGCATGGAAGTCATAGGGAGATGCAATACGGGGGTCCAGTTCCCCATCGACCTCCTCAATTTTGATGAGACGCATGATTACTGGGAACTGAAGATTTGCTGCGCCAATCTACTCAGCAATGATCACAAGATCCTGGAGCTTGGGAGGATAAAAAACCTCAAGGTGCTAGAGATAGGCGAGCAGTCATATACTGGCAAGGTGATGGTTTTCCCGTCAGGCAGCTTCATGACACTTGAACGACTGGTGCTCTACGATCTTGCAGTGGAGAAATGGAAAATTGAGTGTGGATCCATGATATGTCTCAGGGTGTTGACGCTTTGCAAGTGCCCCAAACTGGTCCACCTGCCTGAAGAGTTGTTAAGGCTTCCGAAACTACGCAGTCTGGCTCTTATCGCAATGCCTCTGGGTTGCTACCAGAAAGGTGAGGTTCCTCAGGGGGTGAAGATTTCAGAGTCAGATGATGAGAAAGTTTTCCAGCATCTTCCTATTTGGCGGTGGCTCCACCTGTCTGCCCCGCCCGCATTTGATGAATACTAG

>B73_Zm00001eb405770

ATGGAGGTGGCTCTGGGAACGGCCAAGTCTCTCCTTGGCCATGTTCTCAATAATCTCCCCGACGACTGGATGAAATCCTACGTGTCCAGCGCCGAGCTCGGCACCAACCTTAACATGATCAAAGAGAAGATGCGGTACGCTAGAGCGCTGCTGGACGTGGCCAAGGGGAGGGACGACGTCGTCGCCGGGAACCCCAACCTGCTGGAGCAGCTCGAGACTCTCGGCAAGAAGGCCGACGAGGCTGAGGATGCTGTGGACGAGCTTCACTACTTCATGATCCAGGACAAACACGACGGGACTCGAGACGCCGCACCGGAGTTGGGCGGTGGCCTCGCAGCCCAAGCTCACCATGCCCGCCATGCTGCTCGCCACACTGCTGGTAACTGGCTCTCATGCTTCTCTGGCTGCTGTCCCCGACCCCGAGACGATGCTGCTGCTACCGATGCCATGTCTGGTGACGGTGGCCATGTTGGAAAGTTGTCATTTAATCGAGTGGCTATGTCCAACAAAATCAAGCTCCTCATAGAGGAGCTGCAATCCAACTCTACTCCTGTCTCTGACTTGCTCAAGATAGTGTCAGACACTAGTAACCCTCAAGACAGTAGCTCCTCCACTAAAAGGTCCCCGACAAGCTCTCAAATCACACAAGACAAGTTGTTTGGGAGGGACGCCATCTTTCAGAAAACTATAGAGGATATTATCATAGCCAAAGATAGTGGCAAAACCTTGTCCGTTCTTCCTATATTTGGCCTAGGGGGCATTGGGAAGACTACCTTCACCCAGCACCTATACAATCACACAGAGTTTGAAAAACATTTCACTGTTAGGGTCTGGATATGTGTATCGACTAATTTTGATGTGCTTAGGCTCACCAAAGAGATCCTGAGCTGCCTACCCGCAACTGAAAATGCAGGAGATAAAATAGCAAATGACACAACCAACTTTGACCTGCTTCAGAAATCCATCGCAGAGAGGTTGAAATCCAAAAGGTTTCTGATTGTCTTGGATGACATATGGGAATGCAGCAATAATGAGGAGTGGGAGAAACTAGTAGCTCCATTCAAAAAGAATGATACCACTGGCAACATGATTCTTGTCACAACCCGATTCCAGAAAATTGCAGATTTGGTGAAAAAAGAAACTAACCCAGTTGACCTTCACGGTTTGGATCCTGATGAGTTCTGGAAATTCTTCCAGATATGTGCATTTGGTAGTATTCAAGATGTTGAGCATGGTGATCAAGAGTTAATTGGTATTGCAAGACAAATAGCAGATAAGCTAAAATGCTCCCCACTTGCAGCCAAAACAGTTGGTCGGCTATTGATTAAGAAACCCCTTCAGGAACATTGGATGAAAATTCTTGAGAACAAACAGTGGCTAGAGGAAAAACATGGCAATGATATTATCCCAGCCTTGCAAATTAGCTATGACTACCTTCCCTTCCATCTGAAAAAATGTTTTTCATCTTTCGCCCTTTTCCCTGAGGATTCTAAATTTTATAAGTCTGAGATTATTCGTTTATGGGATTCAATAGGAATCATAGGTTCTAGTATACAGCAAAAGAAAATAGAGGACATAGGATCAGATTATTTTGATGAACTATTAGATAGTGGTTTTCTTATAAAAGGGGGCAATGATTTTTATGTGATGCATGATTTAATCCTTGATCTTTCACGGACTGTTTCAAAACAAGATTGTGCCTATATCGATTGTTCTAGTTTTGAGGCAAATAACATCCCACGGTCTATCCGTTACCTATCCATTTCCATGCAAGATCATTGTGCTCAGAATTTCGAGGAAGAAATGGGTAAACTGAAAGAAAAGATAGACATTAAAAATTTGCGGAGTTTGATGATATTTGGAAAATACATTAGGTTACATCTGCTCAATATTTTAAGGGACACATTTAAGGAAATAAGACGTCTTCGTGTTCTATCTATATTCATATACTCCCATAGTTCCTTGCCAAACAACTTTTCAGAGCTTCTTCATCTCCGCTACTTAAAACTTTTGTCACCTTATTACTCAGAAATGTCATTGCCAAACACGGTCTCAAGGTTTTATCACCTGAAATTTCTAGATCTTGAACAATGGGGAAGTGATCGTTCTTTGCCTAAGGACATTAGCCGCCTTGAAAATCTATGCCATTTTGTTGTTTCGGAAAAGATTGATTCCAATGTTCCTGAGGTGGGGAAAATGATCTTCTTACAAGAATTGAAAGAATTCCATGTTAATAAAGAGAGTGTTGGATTTGAGCTACAAGAATTGGGTAAACTAGATGAGCTTGGAGGGAAGCTCAATATATACGGGCTTGAAAATGTGAGAACCAAGAAAGAAGCTAAAGAGGCCAAGCTGATGTCAAAGAGGAATTTAGTTGAGTTGGGATTAATTTGGAACATGAAACAAGAGTCCACCGAAGATGATATCCTAGATAGTATCCAACCACACTCTAATGTTAGGAGTCTTTTTATTGTAAATCATGGTGGTACCCTCGGTCCTAGTTGGTTGTGCAGCAGCGACACCATATACATGAAAAACTTGGAGACTCTACATCTAGAGAGCGTATCGTGGGCTAACCTTCCACCTATTGGGCAGTTCTATCACTTAAGAGAGCTAAGGCTGAGTAAAATTGTTGGCATATCACAGATTGGACCTGGCTTCTTCGATAGCACGACAGAAAAAAGTGTCTCACACTTGAAGGCAGTTGAGTTTAATGATATGCCAGAGCTTGTCGAGTGGGTTGGGGGAGCTAACTGGAATCTGTTCTCAGGAATTGAAAGAATCAAGTGTACTAATTGTCCTAGGCTGACAGGGTTGCTGGTTTCAGATTGGTCTATTTCTTCTATATAA

>B73_Zm00001eb410900

ATGGCAGAAGGTTTGATACTAGTTGTGCTTCAAAAAATCGCAACCACCTTAGGAGGAGCTGCATTGAGTGTAATTAAGTCAAAATTAGGGAAAGGAGCCAACATTTTACTTGAAGCAGAGAACAGCATGAAAGAAATTGAGAGTGAGTTCGAAATAATGCAAGCATACATAAGTCAAGCAGACCCGTACAGTGAAAGTAACAAAATTCTGAAACCATGGTTGAAGAATGTAAGAAAAATAGCTTCTGAAGTCGAGGACATCATTGATGAATATGCCTTTCTACTTGGAAAACTGGACAACGCAGGATACTTGGCGAAGAAGTTCCACCATTCGAGATACATCACTGCATGGAGTGACATTTCTTCTCAGTTGAAGCAAGTGCAAGCACGCCTACAAAATTTGACAGTTCTGAAAGACAGATATGGCATCACAGTAGTTGGACCTGGTGGTGGATCATCAAGCCATAATAACAGTCGTAAGAATTACCTGTCTGAATCTTCCTACCTGAATGATGATGGTGATGGTGTAATGGTAGGCAACGAAGATGAAGTAAAGAAGTTGACAGAATGCATAGATGGTGCTGGTGCAGATCGTGCAGTTATCTCCATCTGGGGAATGGGCGGTTCAGGAAAAACAATCCTTGCAAGGGGCATCTATAGAAAACGAGAAGTTAGAAAGAACTTCCAGTGCTGTGCCTGGATAACAGTATCACTGAATTATCAAGTCGAAGACCTTTTGAACAAGCTCATAAAGGAACTCCACATCCAAGATGTGCCTGATGCAACTGACAGTACACATTTGGTTGCTAGGATCCAGAATCATCTCAAGGACAAGAGGTACCTTGTTGTTTTGGATGACATGTGGAACAGGGAGTCTTGGTTATTCTTTGATCGAGTATTTGTCAAGAATCTTTATGGAAGTAGAGTCATCGTTACCACTCGAACAGAAGCTGTTGCGTCAATAGCTGAGCTGAACCATACTATAAGAATTGGCCTTCTTTCACAGGGGGAGTCATGGAAGCTCTTCGGCAGAAAGGCATTCTCAAAGATAGGCAAGGAAGAACCGACCTGCCCTCAGGGCCTTGTTCAATGGGCAAACAAAATCCTCGAGAGATGCCAAGGTCTGCCACTTGCTATTGTAGCCATAGGGAGCCTTCTGTCATACAGAGAAATGGAGGAACAAGAGTGGAGACTCTTCTACAATCAACTTAATTGGCAACTGACAAATAACCCAGAACTCAACTTCGTATCGAGCGTCCTAAAGCTAAGCTTGAATGATCTTCCAAGCCACTTGAGGAATTGCTTCCTGTACTGTGGCTTGTTCCCCAAAGACTATCAAATACGTCGGAAGTGTCTAATCAGACTGTGGGTAGCTGAAGGTTTTGTGGAAGACCGGGGAACAGAGATTACACTGGAAGAAGTGGCCGAGGAGTACCTCAAGGAACTCACACGGCGTTCACTGTTTCAAGTGATGGAAAGGAATGAATTTTCACGGCCAAGAAGATTCCAGGTGCACGATCTTGTTAGAGAGATGACACTGGCCATATCAAGAAATGAGAGGTTTGGTCATGTAAGTGACCAGCCAGATGTAACAGACATTGGCGATGTAGGAAAACGTGTATCAGTGCACAGTGGGGGCCAAATCTATCAGCCAGGTCCGTCTTCGCAGCATCTGCGTTCATTTTTGTTGTTTGACAAACACGTGCCGCTTTCATGGATCAGCATCGCTTCATCGGACTTCAAACTGTTAAGAGTCCTGTGCCTCAGGTACTCCCTCCTTGAGGACATTCCAGATGCCATGACTTGTTTGTTCAATCTGCACCATCTAGACTGCTCTCGTACTAAAGTAAGGAAGGTACCAAGATCAGTGGCAAGGCTGAAGAAACTAGAGACATTGCATCTCAGGTTCGCCCGTGTGAGGGAGTTGCCGTCTGAAATAACAATGCTCACAAACCTCCGCCACTTGTCTGTGAGTGACGACTTGTATGGCACATCAATTTGTGGTACTATCCGCAGTCTCAAGCACCTGCAAACTCTTCGAGAGGTGAAAGTCAACAAAGATTTGGCTAAAAGTCTTGGTTACCTGACACAACTAAGAAGCTTAGGAATTACTGGAGTCATACAAAGCCACAATGCAGATCTTTGGGCTTCTATCAGGAAGATGACTGTCCTTAACAAACTAGCCGTCGCAACTCCTGGCGAGAGTAACGAAGTGCTTAGTTTTGAAGAGCTGAGGCCGCTCAAGAACCTGGAGAAGTTCTACTTGACCGGCAAGTTGGCAGAAGGGAAGCTTTTTCCAGTATCTAATGGTTTTCAGAAACTTAAGGTCCTAACAATGCGCTGGTCTAAGCTAACACACGACCCCCTAAGTTCCTTATGTCAAATGGAAAATCTCGTCTACCTTAATCTTTACTGTGCATACGATGGGGAATGCTTGATCTTCTCTTCTGGGTGGTTTCCAAAGCTCAAGCAACTCTACCTAGGCAAGCTTGAGAGACTGAGGTCAATTCAAATAAGTGATGGCGCCATTGAAAATTTAACATACCTGGAACTTCATGAGCTGTGGAATCTAAAAAGTGTCCCTGAAGGCCTTGTATACCTTAGGTCTCTTCAGCACTTGTATGCACGAAAGATGCCTGCAGATTTCGTGGAGGAACTGGAGGGAAGTTGCCAGGGCTTTGTTCGGCACATTGCCAACATTGAATGTATGTGA

>B73_Zm00001eb414490

ATGGCGGTGGTCCTGGATGCTTTGGCATCCTACATCCAAAACATGCTGACACAGATGGCGAAAGAAGAGGTGGACATGCTGCTTGGGGTCTCCGTTGAGATTGACAACCTTGGCGCCAAGCTCGGGGACCTCAAAAACTTCCTCGCTGACGCTGACAGGAGGAACGTCACCGACCGAAGTGTGCGGGCATGGGTGAGGGAGCTCAGAGACGCCATGTACGACGCCACCGACATCCTTGACCTGTGTCAGCTCAAGGCCTTGGAGCGAGGTTCGTCATCATCCCTGGCTACGGGGTGCCTCAACCCCTTGCTGTTCTGCATGAGGAACCCCGTCTTCGCCCACGACATCGGCAGCCGCATCAAGAAGCTCAACAAGAGGCTAGACGCCATCAAGAAGAACAGCGCTACCTTCAGCTTCATCAACCTTGGTTCCTATGAGGACCGCGGTGGGAAGGCGGAGACTCCATCTCGCCTCGCCAACCGCGAGACCTCGGCGCAGCTCGACCGGTCTAGTGTGGTTGGCGAGCAGATTGAGGTCGACACAAGGAAGCTAGTGGAGATGCTAACAGAAGACCCTGGAACGACCACTGCTACCCATGACCAAGGTACGGTCTTGGCCATCGTTGGCATTGGCGGAATTGGCAAGACAACCCTCGCCCAAAAGGTCTTCAACGACGACACCATCAGCCGTGTCTTCACCAAGAAGATTTGGCTGAGTGTCAACAAGGACTTCAGCGTGGCAGAGATCCTAAAGAGAGCCATCATCGAAGCCGGGGGAGATCACCATGCAGCTGGCAATGCAAAAGCCACACTTCAGCGGACGCTTCAGAATGCCTTGGATGGGCACAAGACTATTCTGGTCATGGATGATGTTTGGGATGACAAGGCATGGGGCGATGTTCTTAAAACGCCATTTGTCAATGCTGTTGGTGGTGGTAGCCGAGTCCTCGTCACCACAAGGCATGACTTGGTTGCACGAGCGATGAAAGCCAGGGAGCCCTACCACCACGTCGACAAACTTGACCCCAAAGATGCCTGGTCCTTGCTCAAGAAACAGGTAATCAGAAATGGAGATAATGAACCTCTTATTGATATGCTAGAAGATATTGGAATGAAAATTATAGAAAAATGTGATTGCCTGCCACTTGCTATCAAAGTAATGGGAGGACTCTTGTGCAAGAAAATGGCAAGACGAGGAGACTGGGAAAGGGTATTGAATGATGCGATATGGTCAGTATCAGGAATGCCCGAAGAGCTAAATTATGCAATCTACCTTAGCTACGAAGATTTGCACCCTTCTCTAAAACAATGCTTTTTACACTACTCCCTCATCCCTAATGAGAGCACAGTGTTCTTCGTCGACGACATTGTGAGCATGTGGATCAGCGAAGGATTTGTGGAGGGGAACTCTGACGAATTAGAAGAATTAGCGATGGAGTACTACAATGAGCTAATATTGAGGAGTCTTATAGAACCAGATCTTCTGTATGTTGACCAATGGGTTTGCAACATGCATGATGTAGTTCGCTCATTTGCACAGTATGTGGCAAGAGATGAAGCACTTGTAGCACGAAAGGGGCAAATTGATGTTGGTGAACTCAACTCAAAAAGGATCATTCGACTATCACTGGAATCTGAGGAGTTAGAATGGAGCACTCTGCAACCACAAAAATCACTAAGAACACTACTAGTAGCTGGGCACATAGGGATTACAGTTGGTAACTCACTGGGTGCCTTTCCAAGCCTACGAACCCTGCATATAGACTCAACAAACTTTGATGTAGTGGCTGAATCACTGTGTCAGCTCAAACACTTGAGGTATTTCTCTGTCACAGACCCTAACATGTCTAAGCTGCCGGTAAACATTGGAAATATGAAATTCTTGCAGTATATAAGCCTTGACAGCTGCAAAAACCTGGCTAAGCTTCCTCGTAGCATTGGAAAGCTACAACAGCTTAGGTATCTTAGCCTAATGGGAACAAATATACACTTCATACCGAGAGGGTTCTCTGTCTCAACTAGTCTGAGGAAGCTCTTTGGGTTTCCAGCCCACATGGATGGCAACTGGTGTAGCCTGCAAGTGTTGGAGCCTCTTTCCCGGCTCATGGGCCTCTCTATATATGGTCTAGAAGGTGTGTCTTCTTCCTCCTTCGCCGCAAAGGCTAGGCTTGGTGAGAAGGTGCATCTTAGCTATCTGGAGTTGAGCTGCACCAGTAGACTCAAGGATGACACGCAACTAGTCAAAGAAGATGATGAAGGCTTCTCTGAGGAAGAGCAACAGCGTATTGTGGAAGTGTTTGATGAGCTCCGCCCTCCACCCTGCCTAGATGCTCTTGAAATTGAAGGGTTCTTTGGGCGGTGCTTCCCAAGGTGGATGGGACCAATGGCAGCTGTACCCCTTGAAAACCTGCGGATCCTAGCGATGGATGACCTACCTTGCTGCACTGAGCTCCCCAATGGCCTGTGTCGTCTGCCGTGCTTGGAGTTGCTTCAAATCTGTCGAGCAACAGCCATTGAGCGTGTCGGGCTAGAATTCTTGCAGCCCCATCACCACCATACACACCAGTTGACTGATGTGTTTCCTAGACTACACGACTTAACCTTAACGGAAATGGTGGAATGGGAGGAATGGGAGTGGGAGGAGAATGTGCGAGCCATGCCATTGTTGGAAGAGTTTCTTCTAGAGAGCTGCAAGTTGAGGTGTATCCCTGTTGGCCTTTCTTCCCACGCGAGGTCCTTGAAAAGGTTATATGTACATGACGTACAACACCTCAGCACCCTGGAGAACTTTGCTGCCGTTGTTGAGCTTGAAGCATATGACAACCCCGACCTGACGAGAATTGCTCATTTCCCTAGGCTGTGGAAGCTAGACATCTCTGGGTGCCAAAAGCTAAAGGCATTGGAGGGGTTACCTGCACTCGAGAGGCTTGAGATGACAGATTACAACATGGTGACACTCCCTGCTTACCTGAAGGATGTCAACCCAAAGCATTTGCAAATAGACTGCACCTTACCACTGTTGAGTTCCATAGCCGCAGGAAAATCTAGCCATGAGTGGGACAAGTTTAATCATATCCGACAAGTTAAGGTATATGCAAATGATGGAGATATTGAAAAGAAATGGTATGTGTTGTACACAAGAGATCCTTTCAAGTTGGAGACAAATGTCGTCAACATCAGAGAATGGTGA

>B73_Zm00001eb418840

ATGGATCTTGTGGCCGGCGCCGTGGGCAGCATCATCCGCAAGCTCGGCGAGCTGCTCCAGGCAGAGTACAAGCTACAGGCGGGCCTGCTAGAGCAAATCGAATCTCTGAAAAATGAGCTCGAGAGCGCGCACGCGGCTCTCCGCACCGTGTCGGAGGTGCCGCCGGAGCAGCTTGATCCACAGGTTCGGCTCTGGGCTCGTGAGGTCAGGGAAGCGTCGTACGACATGGAGGACATCCTCGACACCTTCCTCGTCGACGGCGCCCCGGCTGATGGCCTGGGCAAAGGTCGTCGTCTCCTGAAGAAGATGGAGAAGCTGTTCAGAAAGAGCAAGGAGCGCCACGCCATTGCTGGCGCCATTCAGAAGATGAAGGGACGGCTCCAGGAAGTGGCTGACCGCCGCGACAGGTACGCCGTTCCGGTGGCAGCGCCAGCGCCGGTGAGGACGCTGGATCCTCGCCTCGTGTACATGCACAGGGAAGCGGCACAGCTCGTCGGCATCGACAAGACCAAGGCTGAGCTCATGGCCATGCTTCTGCCGCTGTCGTCATCCCGCTGCCCTGAGGACGACGTCGATGTCTCTGCCAGCGACGGTGACAAGATGAAGATAGTTTCTGTGGTCGGAGCTGGTGGCCTTGGAAAGACCACTCTAGCCAAGGCCGTCTACGATGAGCTCAAACCGCGATATGATCATGGAGCATTTGTTTCGGTTGGCCGAAAACCTGACCTGGTGCAAGTCTTTACCAGCATCTTCTTCCATCTCGACGAACAAAAATACAATGCCATTCGTGAAGTGAAGGACCTACAGCTGTTGGCTGGCGAACTACGAAGATTTCTACAAGACAAGAGGTACTTGATCGTTATCGACGACGTTTGGGATACAAAATCTTGGGATACAATAAAATTAGCTTTTGATCAAAAGAATAAGCAGAGCAGAGTAATCACAACCACTCGCAACCGACAAGTAGCTTCCAGTGAGGAGGTTTACGAGCTACATCCGCTCTCTCATGACAGCTCAAAGAAGCTATTTTATATGAGGCTGTTTTGGGGTGAGGACAAATGCCCGGCTAATCATCCTGAAGAGGCATCTCAAAGGATTTTGGACAAATGTGGTGGTGTACCATTAGCTATCATCACAATGGCAAGCTTGCTAGTGGGTAAATCGAGAGAATATTGGTTGGAGGTGTGCAACTCACCTGGTTTCTATCGCGGTAAAGATACCAACGAACAAGTAGACGACACCGTGTGGATACTGTCTCTGAGCTATTATGACCTACCTTCGTATCTAAAAACTTGCTTATTGTACCTAAGTGTGTATCCAGAAGATTATGAGATCGAGAAGCATAGATTGATATGGAAGTGGGTAGCTGAAGGTTTCATCGAGAAGAATGCAGGAAGCAGCAGCTTGTTTGAGCAGGGAGAGGAATACTTCCATGAGCTCATAAATAGAAGCATGATCGAGGCGATGGAGTTCGACGAAGGGTTTGGCATCATAATTGGTTGTCGCGTTCATGACATGGTGCTTGATCTCATCCGTGACATATCAAACAAAGAAAATTTTGTCACTGTCTCATATGATGATGGTAGAAGAGGCACAACGTCGTCATCGTCACGAAACGTGGTGCGTCGGCTAGCTCACCAAAATAGAAGAATGACAGAGGACAATCCTGTGGAGGGCAGCATGACACATCTAAGGTCACTGGTTGCTTGTGGGTGTGATATGGATGGTTGGGTCATGCACTCGAGCTCTACGCTGCTATTGCGTGTGCTAGCTTTAGAGCAATGCTGCACACCACCATCTATGGACATTGGTCATCTTGGAAAACTGCTTCATCTCAGATACCTTGGGCTACGTGGTACTCTCGTAGACAAGCTCCCAGACGAAATAGGATCCCTCAAGCTTCTGCAAGCACTGGATTTACTAGGCACCGGAATATCACGACTTCCACGGACCGTTTGCCTACTAACGCAGCTGAAGTACCTATACGGTGATGCATGCACGATAGTGCCCAATGGTTTCCTCGGGAAGGTGACGTCACTGGAAGAGTTGCATATACATCCTCCTAGCGAAGGTGACGAGTACAACCAACAACAGTTTATGCAGGATTTGGGCACCCACCAGGGAGAAATTAGGGTGCTCGATTTGATGAGGTTCAGAGATGAGTTTGACGATCTGAGCATGGAGTCTGGTCTAGTGCAGGCCCTAGGAAGTCTGCACAAGCTGCAGACCCTACTAGTGAGCAGTGATTACACGAAGCAACAAGTCGCACAGTACAGCTGGGACACGGCGGCCCTTCCGCGGTGTCTCCGGATCTTGGTCTTCGTTGACCTCAGGTTCCATCATGTACCATCGTCCATCAATCCCGCGAGCCTCCCCAACCTCTCACGACTGGAATTGTCTGTGGGTCATCTAGACGAGGCAAGTCTGAGAGCCTTGGGTGGGTTGCCAGGGCTCACCTACCTCACACTAACGGCGGCTGATTGGCTGAAGAGCTCATGCAAGGCTTCGGTAGTTGATGTCGTTGTCGCTGATGGCTTCTTCCTCAAGTTGAGATCCCTCAGGCTGTATGGCTGGATGCTCCAGTTGGTGCCCAGCGAGGACTCGACTAGTGTTTCGTTCAACATCTGGAAAGGAGATGAGGACGTTGTGGCCCTTGGTTTCTGCAGAACAAGTGTAGCAGCACCTATGACCTCCTCTATCATCATGCCAGACCTCATAGACCTGTGGTTCTATGTCCCTGTTAGAGCCTTGTGTAAGAGTAGAAATGGAAGCATCTGTGACAGCCTCGGCTGGGAGTGCCTCCCTTCGCTACACAAAATCGATGCAGTTGTCGACAGTAAGGGCGCCTATATCGGCGATGTGAAGAAGGTCGAGGCTGAGATGAGGCAGGCAGCAAAACTGCATCCCAACCAACCCATAATTAATATTCTACTACTCAATCAATATATTTGA

>B73_Zm00001eb419270

ATGGATCTAGTGACAGGTCCGCTAGCCATTCTCCCGTCCAAGCTGCAGGAGCTACTCCAAATCCAAGATGAGTATAGGCTCCAGAAGGAAGTGAGGGGCAGAGTGGAGATGATCTGCCGGGAGCTGCAAAGTATGCACGCTGCCCTCCGTAAGGTGGCTGATGTGCCGCGGGATCAGATCGACCTCCAGACCAAGGTGTGGAGACGCGATGTCAGAGAGGCATCCTATGACATTGAGGATGCCATCGACACCTTTCTCATGCGCATCAGAGACCGCGCGCCAAGTGACCCAAACAGGTTTCGACTTGCCATGAAGAAGATGACCAAGCTGTCAAGAAAGGTTAAGGCTATCCATGAAATTGGTGCTGCCATCGGAGACATCGTGAGGAAAATCCAGGAGTTGGTGGACCGGCGTGTCAGGTACAAACTTGATGTTGCTGCAGACAGGTCAATCAGCATGGTTAGTGTTGATCCTCGCCTTGCAGCTTTGTACAAAGAAGCGACACAACTCATTGGCATCGACCAATCAATGTCTGAAATAATATCCATGCTGCTCTCGGATGAGTCGGGCCAGCAACACATTAAGAAGATTTCTATTGTCGGAGCTGGAGGACTAGGCAAGACCACCCTTGCTAAAGAAACATATGAAAGGCTTAGAAGTGAATACGAGTTTGCAACTTTTGTTTCAGTTGGCCAAAATCCTGACTTGGTGAGAGTTTTCAAGAACATCCTCGTTAAGCTTGACATATACCAGCAAGTGGACACTATCAACAGAACAGATACGCGAGCGCTCATCGATGAAATCAGACAGCGCCTTAGCTATGTGAGGTGCAGGTATCTCATTGTTATCGATGATGTATGGGATCTGGAGTCTTGGCATACACTAGAATTGGCATTTGTTGAAAATAACAATGGGAGTGGAATAATCATAACTACTCGTAATTTTGCTGTTGCCACAATAGCTGGTCGTGTTTACAAGTTAAGGCCACTTTCGTACCATCACTCCAAAGAATTATTCTACAGATCGTTATTTGGTGAAGGAGTATGCCTTGATGATCAAATAATCAATAAGGCATCAGATAAATTTCTAAAGAAATGTGATGGTATACCGTTAGCTCTCATCACAATGGCTAGTTTGTTCATGGGAAAACCAACACAAATGTGGTCTGAGCTAGCCAATAATGCTTTTGGACATAAAGGTCGTAACAGTCATTTAGAAGAAACGACTATGAGGATATTATCACTTAGCTACTCTGATTTGCCATTGAATTTGAGGACTTGCTTTGTATATTTGAGTAGATATCCAGATGATTATGTTATTAATAAAAGTTCTTTGATATGGAAATGGATAGCTGAAGGTTTTATCCAAGAGGAGCAAGGAAAACAGTTATTTGAGCTCGGGGAAGAATTCTTCAACGAGCTCGTAAGTAGAAGCATTATACAAGTGGTCCAATCAAAATTTGATAACACAGTGCAAGGCTTTCGTATTCATAGTATGGTTCTTGGTCTTATTCGCCTGTTGTCATCCGAAGAAAACTTTGTTTCTGTATTGGACGAAGAAGGGCAAATATCTATATCAGGCAAACTTCGTCGGTTAGCCATCCATAACAGAAAAGAAGAGCACAACCTTGGAGACAATATGGACCTCCTCCCATGGTTGAGGTCGTTTACTGCAATTGAATGTCCTATATACATGATACCTCCGGTTTTCATCTTCAAATTGTTACGTGTTCTGGATTTAGAGAGTTGTGGGTCTATGGAAGGTTATGATCTCAAGCAGCTTGGAAATTTACATAATCTCAGGTTTATCGGTCTATGTAATACATATGTCCGGACGCTCCCACAGGAATTAGGGCATCTAAAGTTTCTGCAGACGCTTGAATTGAAAGGAAGTGGCGTTGAAGAACTACCTTCGAGTATGGGTGAGCTTGCAGGGTTAATGTGCCTCAACGCTGACTGGACAACAAGAGTGCCCAAGTGGATTGGGATGCTTACTTCCCTGCAACAGTTGGTGATGTACCCTTGTGGTGGCTACTCGGCAAGGTGGTTTGTCAAGGGGCTGGGACACCTGAGGGAACTAAGGATGCTCCGTTTGTTGATAAAAGCAGATGACGAGAAGCAGCTTGCACAATTGCTGGAGTCTGTTTTAAAACTGCCCAAGATCGAGGCTTTACATCTTGATTACTATGGCGGCGTACAGTTAAACAGGCTTGTTAAGTTGGAACCTAGTGATTTTGCCTGCAGTGGACGTCTTCGTTCCTTAGAATTGCAGTTGTTGGAATTCTCAAGGCTGCCTGTGTGGCTTAATGCTTACTATCTTCCACAGATCCGTGACTTGTCGCTGTTGATGTTTGATGTGGATAAACAGGATCTGAAAAACCTTGGGGACTTCAAGGAGCTCCGTCATCTCCATCTGCTAATTGTTAACACTGAACGCAGAGATGCTATTACTTGTGCCAGTGGTGGATTCCGGAATTTGAGATTCTTCAGTATCACTAAACCATTCAAGTTTCAACAGGGAGATCTGCCTAGGCTTGAAATCCTTGATTTTCATTTCAATGTGCCACTCCAAAGTGGTGCCAATAGTGGCCTGGATTTTGACTTCGGTTTGGAAAATATCCCTTCGCTTCAGCAAGTCATTGTTCAAATCAACTGTCTAGATGCCTTTCCTCTTCCAGAGGAGGTGGAGACAGCCCTAAGGCACGCAATCGGCGTCCATCCCAACCGTCCCATCGTTGACATCAGTTTGCCTCGACGAAAATTAGACAAGGCAGACAACGACGGGGCCGAAGGGCGTAGTAAGTTGGCTCATTATTCAAAATCATTATCATCGGCACATGATAAGCCAATGGATGAGCTAATAAAGAGGTTATATGTGGTGGACAATGAGGCCTACGATAAAAAGATGAAGATTGTGCCTATTGTTAGATCTGAAGGGCTGGGGAAAACTACTCTTGCCCAAAAAGTATTTGACAAGCTTAGTCCACATTTTGACTGTGCAGCATTTGTTCTGGTAGGCCAAAATCCGGACATGAGGAAAGTTTTCACTGACATTCTCATAGGTCTAGACAACCAAAAGTACCAAGATTTCCCTATGACTATATTGGATATAATTGAGCTGATTGGGTTAGTCCGTAAATCGCTCATAAACAAAAGGTTCTTTGTTGTAGTTGACGATGTATGGGATTTGAAAGCATGGGACATAATAAAGTCTGCATTGATCGAAAACACTAATGGCAGTGCAGTTCTAACAACGACAAGTCGTCAGATCTGGGAACATGAGAACTGGAGGCCATCTGGGGATGACAAGTCGGATTCTGACGCAGAGCAGTTAGAGCCTCATTTGGACGTCACAACCCCCGCGGATGTCCTCCCAGCGTCTTCCCCGAGCAAGAGCAAGGTGGTGGCCCATGTGGAACTCCGCCACGACGACCCAGATTCCTCTGCATTTGGAATCCCGCAAGTAGCAGGGGATACTCAACCTGAGCTCCGCCTTTTCCCCGAGGCGGATGCGGATCTTAAAGGGTTCTTCTACGAAAACCTATTTGGATCGGAAGACAAGTGCCCTGATGATCTGAAAGATATATGTAAAGATCTCATACAAATATCTGCTGGTGTCCTATCAGATGCCTATGAGACCGTGGAAGAGCTCAGAGGTATTACCTACGACACTGGAAAGCTGGCAGCCTGTGTATCAGAGAAGAAAATTCGAGCTCTCTTATCCCAAACTACCTGGCCATCTAAAGGATTGCTTAAGATATTTTAG

>B73_Zm00001eb419360

ATGAACGGTATGAAGGCCGCCGCCGGAGCTGTGGTGACCGAAGCCACGGGTGCCTTTGGGCCCGTCCTGGCGAAGCTCACGGCCTTGCTGACCCCTGATCAGGATGGGGACGACGTGGAGGATTCCATCATGTCCCAGCTGGAGCGCGTGCAGTCGCTCCTCCTCAGGATATGGGAGAGGCAGGATCTTGAGGCAGCATGCGAAGATTGGATGGCGGAGGCACGGAAGGTGTCCTACGACATCCAGGACGACATCGACGGCTTCGAGCTCGGCTTGGAGCATCATGGTGGTGGTTCATCATCAGCTACCAAGAGCCTCCTCGAGAGGATCAAGGCAAAGGTCAAGGGCCTGATGCTGGATAGCCAGAGCGGCTATAAGGAGTGCTGCAGGGCCATTGGCGAGCTGTCTTCGGCGACAGCCCTGCTAGTTGACCATCGATCTCGCTTCTTTCACAAGGATGCGTCCGAGCTTGTGGGGACGATGGAGGAGAAGAAGGCTGAGGTTACCAAGTTGCTCCAAGAGCACGAGGTGGTGTGCATCCATGGTTCTGCAGGGATGGGCAAGACAACTCTTGCTGACTTGGCGTACCAGGCGATGTCGATAGGAGACGACGGACTCAAATGCCGTGCGTTCGTGTCGGTCTCCCCTAGCGCAACCATGGTGCAGATGCTGAAAACCATTCTAAGTCAACTAAGCCGCCGCCACCATTTGCTGTATGGTGGTAGCAATGAAACGTCGTCAGACGAAACGGCTGACGACAAAAGAATTGTATCTGAGGTCGCACGACATGCGGACACGGATATAGCCAAGGAACAGTATCTCATCGAGAGCATATCAAACTTACTCGTGAACAAAAGGTACCTCGTCGTAGTAGATGATGTGTGGCACTGGGAGCAATGGGAAATCATAAGGAAATCCCTTCCGAAGAATAATCTGGGAAGTAGGATAATAACTTGCACTCGTGTTGAAGCAGTAGCCATGAAATTTATGATGGAGAGTGATGCGTGTCTCTACGAAATGGCTGGCCTTTCTATGGATGCCGCCGCAGCTTTGTCGAGGAGGATATTTGAGGATGGCAGCGTGCGTGCAACATCGGAACTGCATAACTCTTGGTCTGCTTCTATAGCAAAAATGTCCGGTGGCATGCCGTTGGCAGTAATTTGTATGTCGTTGGCAGTCGCGCGCGCGCAGCTGACACCAGAGGAGGCAGTTGAAAGATTGACAAATATTCCTTGCTTGAGGCCATTGGTAGAGAGCTTGAGCCTCGGGTACCATGGCCTTCCGCTGCATCTGAAGACTTGCCTGCTGCATTGCAGCATGTATCCTCCCCAGCACATCTTGGACAGAGATGGCCTTGCCAGGATGTGGATCGCTGAAGGGTTTGTCTATGAGGAGGAGGAAGCTCGAAGCTACGTTGACCAGCTTTTCAAAATGGGGTTGATTCGGCGGTGCTCTTCTGCTCTTGTGCCGTCTCCAAAGTTTCAGATCGACGCCATGATGCTTTATTTCCTTAGGTGTAGATCACAAGAAACATTTTACTATTTGGGTTGCGGTCGTCGTCGGAATGGCGCCGGACGACGGGTGCATCATCGGCTATCTATCATCTCTGCCCAGAAGAACGGGCCGGCGGGGGGTACTTCAACACTGCCACACGTCGACGTCGTCTCGAATAATAATACTCGCTCCCTGTATGTTTTCGACCGCTCATGGAGGGTCCCTTTCAACGAGTTTGAACGGCTGCGGGTGCTGCACCTTCACTGCAGCTCCAGGAACGCTGATCTGGTGGATATATGCGGGCTGGTGTGGCTGAGATGCCTCAGTCTCAGTGGGTCACCGATCACCATGCTCCCAAAGCAAATTGGGAGGCTGCAGCTTCTAAGGACTCTGGATGTAAGCAGCACAGGAATCCTGTGTGTACCAGGTGAGATTGGGGCGCTGCAGCAATTGGAGATTCTTGACGTGAGCGACACGATGGTCCAGAAGCTTCCGGCGGAAATAGGGCAGCTGAGGCGCTTGAAGGCTCTGTACGCAAGGAACTCCAGCCTCAGAGAGCTGCACACTAGCCAGATCAGAGAGCTGCAGCTACTGGAGACGCTTGATGTAAGCAACACGGTGCTTGCTGAGGGTGGTGGTCTCATGATGTTAATTGGAGATGATGAGAACTTGAAGGTCGTGGACGCCGCAGGTGGTGGTGGAATGCTGATGATGGCCGAGGGGGGAGGGTCAACTGCGACCGTAGCCACTGGTGCCCTGGGCCCCGTCCTAGCAAAGCTGCGCAAGAACGCGGCGGACGACGACGGCGGGCGATATCTTGGCGACATAGAGTTCATCATATCAGAGCTCGAGTCCCTGCACTCCTTCCTCACAAGGATGTGGGAGAGTAGTGAGGGCGGCGGCATTGACGAGAATGCGGCATGCCAGAGCTGGATGGCGGAGGCACGAGAGCTGTCCTACCACATTGAGGACGACATCGATCGCTTTATGCTCGATTTGGAGCATGGCGGCGACAGGACGACGACGACGGCACCTTTTAGGAGAATCAAGCTACGTATCAAGGGGCTGGTGGACCGGTACCAAGAGGAATGGAGGATGGCTGCTGATTGCCGTATCAAGGCGCCCCAACTTGGTCCTCCGTCCCGGTACTACTCTCGCAAGGATGCCTCCGAGTTGTTGGAGATCGACGACAAGGAAACTGAGGTCAAGGAATTGCTTCAAGAGCATGAGCTGTTATGCATCTTCGGATTTGCAGGAATGGGTAAGACGACTCTAGCCAACCAAGTGTACCAAGCAATAGGAAAGCAATTCGAATGCCAGGCCTTCATGTCCATCTCCCCACGCATGGACGCGTCGCAGATCCTGACAACTTTTTATGCCCAAGTAACGGCTCAACAAAGAGAAATAATAGCCGCGAGTGATTCATTGTCGGACCAACAATTTATGATCGACAGCATCTCAAAATTCCTCTTCAACAAAAGGTGCCTCATCATAGTCAATGACGTCTGGCACTGGGAACAATGGGAAGTGATAAGAAAGTCCCTTCCGAAGAATAATCTGGGCAGTAGAATAATTACGAGCACTTGTGTTCATGCAGTGGCAATGAGATTTGTAGAGGACAATGGTGTCTTTCTCTACAAAATTGCGGGTCTTTCTATGGATGCCTCAGTATCTTTATGTGAAAGGATATTTAAGGAGTCCTGTAAGGATGGCGGCACCGTCGTTTCATCCGGCCTGTGCAGTTCTATAGCGAAAATGGCTGGTGGCATGCCGTTGGCAGTAATTTGTTTGTCGTCGGCAGTTGCAGCGCAGGCGCAGCTAGCAACAGAAGAAGACGTCGGGCGAGTGCATGACCCGCCGGATTGGTTTCGTATGGCAGAAGCTCGGGCACTAGCAGGACTGACAAATATTCCATGCTTGAAGCCATTGGCAGATAGCTTGTGCCTCGCCTACAATCAACTTTCAGTCCATCTCAAGACTTGCTTGCTGCACTGCAGCAGCCTGTGTCCTCCCTATCACATATTTGGGACGGATGATCTCGTCAGGCTGTGGATAGCTGAAAGGTTTGTATACGATGAGGGAGATGCTCGAAGCTATGTGGATCAGCTTGTCAGCACGGGAATGATTTCAGTACAATCTTGCTCCTCGGATCTGTTGATTGGCCCGAAGAAAGTTCAGATGAACACCATGATGCTGCACTTCCTTAGGTGCATTGCATCACATGAAGGTTATTGCATTGCTTCTTCGGATTGTGGATCTGGCATGTCATCTCTGCTCGTTGGACCAATCCGGCGGCTATCTATCCAGAGCTGCAAGAACAAGGTGGAGATTTCAAGGTTACATGCTTCGGATATTCGCTCCCTTTATGTTTTTGACCGCACGTGGACAACCCTGTTCAAGAACTTGACAAACCTTCGAGTGTTGCAGCTTCTGGGCAACAATCTTAGGAATGAAGATCTGACGGCTATATGTGGGCTGCCCCACCTTACTTACCTGAATCTGAGCGGGACACTGGTGAATCTGCTCCCAGAGGAACTAGGGAGACTGAAGCTTCTCAAGACTCTGATTGTAAGAGGCACTGGAATCCTGCGTCTACCAGTGGGAATTGGGGAGCTGATGCAATTGGAGACTCTTGATCTAGGTGACACCATGGTCGTGGAGCTACCGAGCCAAATAGTGAAGCTGCATAACTTGAGGACACTGGACCTAAGAAACTCAAGCTTGTGTGAGCTCCCCAACGAAATCGGCGACCTACTGCTCTTGGAGACTCTTGATGTAGGTGACACCATGGTCGCGGAGCTACCGAGCCAAATAGGGAAGCTGCAGCGTTTGAGGGCTCTGGGCATAAGCGGCACAGGTATCGAGAAGCTTCCAGCGGAGATTGGAGAGCTGGAGCATTTGGAAATTCTTGATGTAAGTTGCACAACGGTCGCCGAGCTACCACTACCGACAGCCGGAGGATCGATGGCGATGCTGCAGCAACTGAACATTTGCAACACAGAGATCCGAGAGCTACCTTGGGAATCAGGGCGGCGCTCGGTCCGTGTGCTGTGTGGTGACAGCGACGCTCCTCATGTGTGCAACCTGGCTGACCTTTCTGAGGGCCTTTTGCAACCATCTGTCGTCGTCGTTCTGTTTGATCGTTTCGGTTCGATCTGGGTACCTGTGCCTTTCCCGAGAATTAAGGTTCCTGGGAAGCATAGGAGTGTCCCTCAGTTTGTCGCGTCGATTTGTTACCTGGAAATCAGTCTGTGGAAGCTGGAGGAGGATTCCCTCAAGTCTCTGCGGGAGATGCCCAACCTTCGAGGTCTAGCACTACGGGTTGATGTTCTCCCCACAAAGCCTGCTATATCCATCCTAGGGTTCCGAAAGCTGGAGAGCCTCTGTATCGATTGCCGTGTGCCAAGGATAACCTTCGAGAAAGAAGCAATGCCAGAGCTCACATATCTCGAGTTCAAGTTCTATGCCTGCCGGCCGACAACTGAACACAACATGGGCATCGCTAACCTCCAGAGACTCCAAAGAGTTGTTTTCCGGAGCGCCTCTTGGTACACAAGCGACCCTTCGGCGGGCATTCATGCAGTTATTAACAGAGTGAGAGAAGAAGCAAGGGAGCACCCCAACACAATCATCCTAAGCGTCAATGGCAACGTCGAATCTTACCATGAGAAGAAAGCTGCGGCAGCAGCAGCACAAGGGATCAAGCCAAGCCTTTCCGGTGATGTTTCTCGGCGCAGCGATTCCCGAGCTCGTACAATCCGCAGAATAAAAACCTATGCGGGAGAATCATCAGCCTATAACTAG

>B97_Zm00018ab011400

ATGGAGCTACAGCTCTCGGCCGTGCTCGGCTCGCTCGCCCTCGGCGGCGCGGTGCTGGTGCTGTTCTTCGTTAAGTGGTGGCAGCCGCTGGCCGGCACCGACCGGCGCGTCAAGGAGCTTGCAGACGCGGTGGAGGCCCTGCTGCGGCAGCGGTCCGAGGTGCTGGGCCACGACCCGGCTCCGTCGTCGGATACCGTGCGCGCGTGGCTGCGGCGCGTGCAGGAGGCGCAGGACGAGATGGCGTCCATCAAGGCGCGGCACGACGGCGAGCAGCTATACGTGGTCCGCCTGGTTCAGTACCTCTTCCTCCCCACGGGCCCGGTCGCGGGGCTGGCCGAGCAGCAGCTCAAGGCAGTGCGCGCGCTCCGAGAGCAGGGCGCCGCGATCCTCGATGCCGCGCTGGCCACGCCGCAGGCGCCGCCGCCTCTTCTCTGCGACCCCGAGGAGCTGGAGGGCCTCCCGGCGGAGGCGGGGCCCGCGAGGGCCTACCTCAACGAGGCGCTGCGCTTCCTCGGCGACTGCGACGCCGCGCTTGGCGTCTGGGGTGCCGGCGGCGTGGGTAAAACCACGGTGCTGAAGCTGGTGCGCGAGGTGTGCGGCCGCGTCGCGCGCTTCGACCACGTCCTACTCGTCGCGGCCTCCAGGGACTGCACGGTGGCCAAGCTCCAGAGGGAGGTCGTGTCCGTGCTCGGGCTGCGCGACGCGCCCACGGAGCAGGCGCAGGCCGCCGGGATCCTGAGCTTCCTGAGGGACAAGAGCTTCCTGCTGCTGTTGGACAGCGTGTGGGAACGTCTGGACCTGGAGAGGGTCGGCATCCCGCAGCCCCTCGGCATGGCTAACGGCAAGGTGAGGAAGATCATAGTGGCGTCGAGGAGCGAGGCCTTGTGCGCCGACATGGGCTGCCGCAACAAGATCAAGATGGAGTGCTTGAACGAGGAGGATGCGTGGAGCCTGTTTCAAGCTAATGTTGGCGGCGACATCATCCATGGCCACGCTCAAATTCCTGCACTTGCTAAACAGGTCGCTGCCGAATGCAAGTGCTTGCCTTTGGCCCTCGTCACCGTCGGCCGCGCAATGTCAAATAAGCGCACACCAGAGGAGTGGTCCAACGCACTCGACACCCTCAAGGCATCGCTCCGCTCCGGCACGCCCGGCTTGGACAAGAGCACGCACGCGCTAGTGAAGTTCTGCTACGACAACCTGGAGAGCGACATGGTGAGGGAATGCCTCCTGACCTGTGCGCTATGGCCGGAGGACCACAACATCTCCAAGGAGGAGCTCGTGCAGAGCTGGATAGGACTCGGCCTGCTCCCCGATCTCGGCGACATCGAAGAGGCCCACAGGTTCGGGCTCTCGGTGATTGCCATCATGATGGCCGCGTGCCTGCTGGAGCCCGGGGACAACCACCGCTACAACATGTTCCCGTCAGACACTCACGTCAGGATGCACGATGTCGTGCGCGACGCGGCGCTCCGGCTCGCGCCCGCCAAGTGGCTGGTCCGCGCAGGCGCTGGGCTCAGGGAGCCCCCGCGCGAGGAGGCGCTGTGGCGGAGCGCGCAGCGCGTGTCCCTGATGCACAACACCATCGAGGACGTGCCGGCGAAGGTGGGTGGCGCCCTCGCGGACGCGCAGCCGGCGTCGCTGATGCTCCAGTGCAACAAGGCCCTGCCGAAGAGGATGCTCCAGGCGATCCAGCATTTCACCAAGCTCACGTACCTGGACCTCGAGGACACCGGCATTCAGGACGCCTTCCCCATGGAGATCTGCTGTTTGGTCAGCTTGAAGCACCTCAACCTATCCAAGAACAAGATCCTGTCGCTGCCGATGGAGCTGGGCAACCTGAGCCAGCTCGAGTACTTCTACCTGCGCGACAACTACTACATCCAGATCACCATACCACCGGGGCTGATCTCGCGGCTTGGGAAGCTGCAGGTGCTGGAGGTTTTCACCGCGAGCATCGTCTCCGTCGCGGACAACTACGTCGCGCCAGTCATTGACGACCTCGAGAGCAGCGGCGCGCGCATGGCGTCGCTCGGCATCTGGCTCGACACCACCCGCGACGTGGAGCGCCTCGCGCGGCTAGCGCCGGGCGTGCGCGCCCGGTCGCTGCACCTGCGCAAGTTAGAAGGGACGCGCGCCCTGCCGCTGCTGTCCGCGGAGCACGCGCCGGAGCTTGCCGGCGTGCAGGAGAGCCTGCGGGAGCTGGTGGTCTACTCCTCCGACGTCGACGAGATCACGGCCGACGCGCACGTGCCCATGCTGGAGGTTATCAAGTTTGGGTTCCTTACGAAGCTGCGCGTCATGGCGTGGTCCCACGCCGCCGGGTCCAACCTCCGCGAGGTCGCCATGGGCGCGTGCCACAGTCTAACTCACCTGACGTGGGTGCAGAACCTCCCCTGCCTAGAATCGCTGAACCTCAGCGGGTGCAACGGGCTGACGAGACTGCTGGGTGGCGCGGAGGACAGCGGCAGCGCCACGGAGGAGGTGATCGTGTTCCCGCGTCTGAAGCTGCTGGCCCTGCTGGGGCTGCCGAAGCTGGAGGCCGTGCGAGTCGAGGGAGAGTGCGCGTTCCCGGAGCTGCGGCGCCTGCAGACGAGGGGGTGCCCGCGGCTGAAGAGGATTCCTATGCGCCCGGCGCGCGGGCAGCAAGGTACCGTGCGGATCGAGTGCGACAAGCACTGGTGGAACGCTCTACAGTGGGCGGGCGAGGACGTCAAGGCCTGCTTCGTCCCTGTGCTGTGA

>B97_Zm00018ab016280

ATGGATTGGTTGAGCAGCATGCTGGGGGACCGCCCGCTGAAGAGCGTCTTCACGGCGCTCGGTCTCCCGGATAAGATTGGCGGCGCGGTAATCGACGCTCTCTGCTACCGGGGCGTCCGTCTGTGGAACGTCGAGGAGGAGGCCGACAAGCTGCGGCGCACCAAGGAACGCATCCGCGCCGTGCTCGAGGACGCCGAGCAACGCCGCTTCATCGACCACGACTCTGTCAGGCTCTGGCTCCGGGAGCTTAGGGCCGTCGCCTTCGACGTCGACGCCCTGCTCGACCGCTTGGGAACCATCACGGCCGTGTCCAGGCTAGCGGCCGCCGAGCAATCACGGAAGCGGAAGCGGCTATGGCCCAGCGTCGAGCTCGGCCCGCGGCAGCGGTGGGAGCTGGATGAGAAGATCGCGAAGATCAACGAACGCCTCGACGAGATCAACACGGGCAGGAAATGGTATAGGTTGCAGGCCGGGGACGGGACGAGGACAGCGTCTCAGCCGACGCAGCGCCCACGGTTCCTTGAATCTGCCGCGCATCGCGACGAGAGGCCCATTGGCCGCAACGAAGAGAAGGAGCAGATTGTCCGTGCTCTGGTTTCGGATAGCGCAGATATGGCGGTGATTTCCATATGGGGAACGACAGGCATCGGGAAGACAGCACTGGCACAATCGGTTTACAAAGATCCTGAGGTACAAAACTTCTTCACCGACAAGATCTGGGTTTGGTTATCAGATAGGTGTGATATCAGAAAGGCCACCAAAATGATCATCGAAGCGGTGACTAATCAAAAATGTGAGCTTCTAAGCTTGGACATATTGCAGCAACGGCTGCACGACCACCTACATAAAAAGCAGTTCTTGCTGGTGATTGATAACCTTTGGGCAGAGAGCTTTCAGTTCTGGGAGTTTCTGAGGCCCTCATTGACTGGTGGAGCGGAAGGAAGCAAGGTTCTGATCACTACTCAGCATGAAAAGGTGTCTAGGATGATTTCCACCAATCTAAACATCCATTTAAAGGGCTTGGAAGATGAAGAATGCTGGCAAATCCTCAAACTCTATGCGTTCTCGGGGTGGGGCAGCAGAGATCAGCATGATCTGGAACCCATTGGGCGGAGCATTGCCTCAAACTGCCAAGGCTCCCCGTTAGCTGCTAAATCTATTGGGTTACTACTGTCCGACACTCATGGAGACAAAGAACAGTGGGAAAACATACTAGGTGAAATGCAGATTCTCGGAGATGGCGAAAACACAAACAGCATATTACCAAGTTTGCAGATAAGTTACCAGCACTTGTCATATCATCTCAAACAGTGCTTTGCCTTCTGTTCAATACTTCCTCCTGGTGTTGAGTTTGAGAAGGATGAGCTCGTCAGACTCTGGATAGCTGATGGTCTTGTTAAGAGTAACGGAAGGGAAAGGGTTGAGATGGAGGCAGGACGATGCTTTGATGAGCTCCTATGGAGATCATTCTTTGAAACATCCCGCAGCTTCCCTGATCAAAAGTTTAGAGTGCCAAGTTTGATGCTTGAGCTAGCACAGCTTGTTTCTAAACACGAATCTCTGACTCTCAGACCTGAGGATTCACCGGTAGTCGACCATCCCGAGTGGATTCGTTATACAACTATACTGTGCCCGAAAGGTGAGCCTCTTGCATGCGACAAGATCTATCGCTATGAAAATTCGAGGCTCTTGAAATTATGCCCCGCAATGAAACTACCTTTGAACCAGGTACCAACAACACTTTTCTCAAAGTTAACTTGTCTGCGTGCACTAGACCTGAGTTACACTGAGCTAGACCTCCTGCCAGATTCCGTTGGGTCCTGCATACACCTCAGATACCTCAACCTTCGGAATACTCTGATAAAGACTCTTCCAGGAACAGTCTGCGGCCTATTCAATTTGCAGACACTTGACCTCAGGGACTGCTACTGGCTCACGGATCTGCCTGCAGGCATGAGCCGCTTAGTTAACCTGCGTCACCTTAGCTTGCATATCGATTGGGATAGAGTCACTGCTCTTAGATCGATGCCGAGTGGCATAGACCGGCTACAGTCACTTCAAACTCTTTCCAGGTTCGTTGTGGTCTCCAGGGATGGGGGCAGGTGCAACATCAACGAGCTGAGGAACTTGAAGATCCGCGGAGAGCTTTGCATTCTTAATCTGGAAGCTGCCACCAGCGACGGTGCCACGGAGGCCAATCTGCGCGGGAAGGAGTACCTGCGCGAACTGATGCTGAAGTGGAGCGAGGACGCCTGCAAGGACGAGCAGCAGCAGCAGCAGCAGGGCATAGAGAACAGCGAGGCGGTAATCGAGGCACTCTGTCCGCACACCAGCCTCAAGCGTCTGCGCGTCGAGAATTACCCCGGAAGACGGTTTCCTCCCTGCTTCGAGAACCTCCCGTCCCTGGAATCTCTGGAGATAGTCTCCTGCCCCAGGCTCACTCAGTTCTCCGTGCGGATGATGCGGTCTCTCAGGAATCTGAGGATACGCCAGTGTGCTGATCTTGCGGTTCTTCCCGGAGGCCTGTGCGGCCTAGAATCCCTTCGTTGTCTGGAAACCGTCGGCGCTCCGAATCTGAGAATAGGCGCAGTGGACATACTGCCGAGGAACGTATTACGACTGGCTGTGAGTGGGTGCGACTCGCTAGAGAGATGGTGCCTGGAAGAAGGCGCTGAGAGGGTGCAGCAGATTCCTGATGTAATTGTAAGGATGCGGTTGAAAAATTGA

>B97_Zm00018ab025360

ATGGCAGAGGGTGTTGTTGGCATCCTTATTCTGAAGCTTGGTTCAGCCTTGTTTTTAGAGGCTTGCAGGCTTGGCACAAAACAGCTCTACCATGAAGCTTCAGCCCTTGGCAGGCTCTTTGGTGAGATCCGTGATATCAAGGAGGAATTGGAGAGCATGCAATCTTTTCTACAAGGAGCTGAGAGATTCAAAGATACTGACAACAACACTGCCAACTTTATCAAGAAGATTCGTGGCCTTGCTTTCGACATTGAAGATGTTATCGATGAGTTTATCTACAAGATGGAGGACAAGCATGGCAGTTTTGCTACAAAGATGAATCGTCGGATTAATCGTATTTGGACATGGCGACGTCTCACGTCCAAATTGCAAAAGATCAAACTGAAACTAGAGAATGTTGATAAGAGAAATGTTCGATATGACATGAGAGGAATTGCTAGAGAAGATGGAAGCAGTGATGCTCATCATAGATCTACCGACCAGATTTCTTACTTCCCCAAAGAGGAAAATCTTGTGGGCATTGATGAAAACAAGGAGTTGTTGATGAATTGGCTAAGGGGTGATCTACAGCAGCAAAGTGTAATTACAACAGTATGGGGGATGGGGGGAGTTGGCAAGACCACTTTGGTTGCACATGTTTACAACACTGTGAAGGTAGACTTTGACAGTGCTGCATGGATAACTGTTTCAAAAGCTTACCAAGTGGAGGACTTGCTGAAGCAGATCATCAGGGGATTTCAGAAAAGTGATTTGAAGGGTGAACTTCGTGTTGACATAATTGACATGGAAAAGAGAAGCCTAGTTGAGATCATCCGTGATTATTTGCATGGCAAAAGCTATGTTTTAGTGCTAGATGACGTCTGGGGTGTTGACATCTGGTTCAAGATAAGAGATGCTTTTCCTACCAATAGCACTAGCAGGTTTATTATTACATCGAGGATACATGAAGTAGCATTGCTGGCTAATGGAAATTGCATAATTGAGTTGAAGCCACTAGAGGCACACCATTCATGGGAGTTATTTTGTAAAGAGGCCTTCTGGAAAAATGAAAACAAAATGTGCCCGCTGGAACTTAATAATTTGGCACAAAGATTTGTTGACAAGTGTAACGGGCTGCCCATTGCCATTGCATGCATAGGCCGTCTTTTGTCTTGCAGAAGCCCAACCTACTCTGATTGGGAAAGCTTGTACAAGGAACTAGAGTTGCAGATGACAAATAATGTGATTCTTAATGTTAATGTTGTTCTAAAGATTAGTTTAGAGGATCTTCCATATATTTTAAAGAACTGCTTTTTGCACTGTACAATATTTCCCGAGGATCATTTGATCAAAAGGAAAAGGTTAATTAGGCACTGGGTAGCAGAGGGATTCATCAGAGAAACAGAGCACAAAACAATGGAGGAAGTGGCAGAGGGCTATTTGTATGAACTTGTCAATCGTAGCCTATTACAAGTAGTGGAGAGAAATGAAAGTGGACGAGTGCAGAGTTGCCGAATGCATGATATTATTCGACTTCTTGCTCTGACAAAAGCAAATGAGGAAGGCTTCTGTAAAGTTTATGATGGCATGGGGAGTTATTCAGCAGAAAAGACACGTCGTTTATCGATTCACAGTGCAAATATTAAGTTGTCGACTCAACCAACAGAGCTTACAGTCCGCTCAATATATGTTTTTAGTAATGGTTTGACTATTGAATCACTTAGGTCTTTCTTGAAACATTTCTACTTGCTGTCAACTCTAGATCTCCAGGGTGCCCAGATTGTGGAGCTGCCAGATGAGGTTTTCAACTTGTTTAATCTACGGTTTCTCAGCCTTCGAAATACTGAGGTTACGAATATCCCCAGCACAGTTGGAAGATTACAAAAACTTGAAGTCTTGGATGTTTATAATGCTAAACTGTTGGCTTTGCCAGAGAGTGTTTCGAAGCTTAGAAAATTGAGATATCTACATGTAGCTACTGTTCCAAAGATAAATACTAAAGGGGTTGTGACCTGGATTGGAATCCAGGTGCCTAAAAGCATCAAATACCTGACAGGCTTGCAAACCTTGAGGCTTGTTGAGGCGAGGTCAGAGACTTTATTTCACCTTGGTGCTTTGACACAGTTGAGAACTTTTGCCATCACAAATGTGCAGAGGGAACAGTGTGCCGATTTGTGCACTGTTATAATGAGCATGAAGCATCTTGTTAGCTTAGCAATTATGGCTATAAGTGAGGAGGAAACACTTCAACTTGAAGAACTTTGTTTACCCCCAACTCTTTCAAAGCTTGAATTAGGAGGGCAACTGGACAAGAAAACAATGCCCCAGATTGTATCATCCTTTTCAGATCTTGGTAACCTCACCTTATTGGCCTTGGCATTCTCCAAACTTGATGAGGACTCATTTTCATGCCTCTTGACGTTGCATGGTCTACGTGGGCTTTGGGTTGATAAGGCTTATGAAGGGAAGAGGCTTCACTTTAATGCTATGTCTTTTCCAAATCTTCGACAGCTTGCAATATCAGATGCACCGCAGCTCAACAGTGTTGTAATCGAACGAAGCGCACTGCAAAGCCTTGTTCAGCTGACACTTGTAGATTGTCCAGAACTGAAGGCCCTGCCTGATGGCATTGAGCATCTTAGAACACTTGAGAAATTATATCTGCGAGGAGCATCCAAAGAGCTCACAAAGCTATTTCAGTGCAATGAAGAAACACATGAGTCCAATGGGAATCTTGAGAAGATCGGTCATATCCGAAGGGTTACTGTTTATCCATAA

>B97_Zm00018ab032210

ATGGACCGGATGCTGCTCGACCAGCTGGCCGGCGAGGCCCTGCGGGAGGTGCTGCACGCGGTGCAGGGCACCCTGTTCTGCCGCTCCACCGCCGAGCGCCTGCGCCGGAGCGTCGAGCCGCTGCTGCCGCTCGTCCAGGGCCTCGGCCCGCACAGCACCCAGCGCTCCGCGGGGGAGCTCGGCGAGCTCGCGGCGCGGGTCAGGGAGGCGCTCGACCTGGCGCGCCGCGCCGCCGCGTCCCCGCGCTGGAACGTCTACCGCGCCGCGCAGCTGTCGCGCCGGATGGAGGCGGCCGACCGCGGCATCGCGCGCTGGCTGGAGCGCCACGCCCCCGCGCACGTCATCGGCGGCGTGCGCAGGCTCCGCGACGAGGCCGACGCGCGCATCGGTCGCCTCGAGCGCCGCGTCGAGGAGATCGCCGCCGCCACCGCGCAGCCGCCGCCCCCCGCCCTCTCCGTCCCCGTCGCGCCGCCGCCGCACAAGGGCGTGCCCATGCCGATGGAGGCGCCGCTCGCTAAGCCCGCCTTCGTCGCTATGACGAAGGAGGTGCCGCAGCACAAGGGCATGGCTATGTCGGAGCCGGTGCCGGCGAAGGCGGCGCCCGCCAAAGCCGGGGTGATGGCCATGGACATCGCCGACGGACACGAAGACGCGGAGGGGATGGTTGGCGGCGGCGTCAAGGTGGCCAAGGAAAAGGTGAAGGAGATGGTTATGAGCGGCGGCGGCAGCTGGGAGGTGGTCGGGATCTCCGGCATGGGCGGCAGCGGCAAGACCACGCTCGCCATGGAGGTCTTCAGGGATCACAAGGTCCGAGCCTACTTCAACGACAGGATCTTCTTCGAGACGATCTCGCAGTCCGCGAATCTGGAGGCCATCAAGATGAAGCTGTGGGAGCAGATCAGCGGCAACATGGTGCTGGGTACATACAACCAGATCCCAGAATGGCAGCTCAAGCTAGGACCAAGGGACCGAGGACCCGTCCTTGTGATCCTCGACGATGTTTGGTCTCTCCCGCAGCTTGAGGAGCTCATCTTCAAGTTCCCTGGGTGCAAGACCCTAGTCGTATCAAGGTTCAAGTTCCCCACGCTGGTGAAACAGACGTATGAGATGCAGCTGCTAGACGAGGCGGCGGCTCTGTCCGTCTTCTGCCGCGCTGCGTTCGACCAGGAGTGTGTTCCGCAGACCGCCGACAAGAGATTGGTCAGGCAGGTCTCTGCAGAGTGCAGAGGTCTCCCTCTGGCTCTGAAGGTCATCGGCGCGTCGCTGCGCGACCAGCCTCCGAAGATTTGGCTCAGCGCCAAAAACCGGTTGTCTCGAGGAGAGGCCATTTCTGACTGCCATGAGACCAAGCTTCTGGAGAGGATGGCGGCCAGTGTCGAGTGCTTGTCCGAGAAGGTTAGGGACTGTTTCCTTGACCTGGGCTGCTTCCCGGAGGACAAGAAGATCCCCCTCGACGTCTTGATCAACATCTGGATGGAGATCCATGACCTTGATGAGCCAGATGCTTTTGCCATCTTGGTTGAGCTTTCGAACAAGAACCTTCTTACCCTCGTTAACGATGCACAGAACAAGGCTGGAGATCTGTACAGTAGCTACCATGACTACTCGGTGACACAGCACGACGTGTTGAGAGATCTTGCTCTTCACATGAGCGGGCGTGACCCGCTCAACAAGCGCAGGCGGTTGGTGATGCCGAGAAGGGAAGAAACACTTCCGAGGGATTGGCAGAGGAACAAGGATGCTCCGTTTGAAGCTCAGATAGTCTCCATTCATACAGGCGAAATGAAAGAATCCGACTGGTTCCAGATGAGCTTCCCCAAGGCAGAAGTGCTGATCCTCAACTTCGCGTCGAGCCTGTACTACCTGCCGCCGTTCATCGCGACGATGCAGAACCTGAAGGCCCTGGTGCTGATCAACTACGGCAGCAGCAGCAGCAGCGCAGCCCTGGACAACCTCTCCGCCTTCACCACGCTGAGCGGGCTGAGGAGCCTGTGGCTGGAGAAGATCAGGCTGCCGCCGCTGCCCAAGACGACGATCCCGCTGAGGAACCTGCACAAGATCTCGCTCGTGCTCTGCGAGCTGAACAGCAGTCTAAGAGGGTCGACGATGGACCTGTCGACGACGTTCCCGCGCCTGTCCAACCTGACGATCGACCACTGCATAGACCTCAAGGAGCTGCCGCCGAGCGTCTGCGAGATCGGGTCCCTGGAGACCATCTCCATCTCCAACTGCCACGACCTCACCGAGCTGCCATACGAGCTGGGGCGGCTGCGCTGCCTCAGCATCCTCCGCGTGTACGCCTGCCCGGCGCTGTGGCGGCTGCCGGCGTCGGTGTGCAGCCTGAAGCGGCTCAAGTACCTGGACATCTCGCAGTGCATCAACCTGACGGACCTCCCCGAGGAGCTCGGCCACCTGACGAGCCTGGAGAAGATCGACATGCGCGAGTGCTCGCGCCTCAGGAGCCTCCCCAGGTCGTCGTCCTCGCTCAAGTCCCTCGGACACGTCGTGTGCGACGAGGAGACGGCGCTGCTGTGGCGTGAGGCCGAGCAGGTCATCCCTGACCTCCGCGTGCAGGTGGCCGAGGAGTGCTACAACCTGGACTGGCTCGCGGACTGA

>B97_Zm00018ab035060

ATGGCGGACGCGGGGGTGACGGGGGTACTGGCCAAGCTGGGTGAGCTGGCGGCGGAGGAGGCGACGGCGCTGCTGCGCGTGGACGCCGAGATCCGGGCGTTGCGGCGGAAGCTGGCCTACCTGCAGGCGCTCGTACGCGGGGCAGACCGCCAGCGCCGCGGCCGCGCAAGCGAGCTGCTCCTGCTCTGGCTGCGCGAGACCAGAGAGGTTGCTTTCGAGGTTGAGGACGCCGTCGATGAGTTCCACCTCCGCGTCGAGGCCTGCCGCCCCGGGGCCCGGTGGCGCCGGCGCCGCAGATGGTGGTGGGGCTGGCACCGCGACGCCGTCAGCCTCGTCCAGGACCTCGCCACGCAGTTTTTTGTACGTCATGGGCTGTCAAATCAAATATCTAAGATCAATGAAAGGATTGATGAGCTTAACCAGAACAAGGAAACATATCAAATTGAAAGTTCTCCTTCTGAAATTTGGAGTTCTTCATCGGTTGAAATGGATCCTGAGTGGTACGAAGATAAATATGTTATAGGCTCTAGAGAACGTGAATTTGCTATCCTTAAGGACCTAATCATCAACAAAGAGGGAGATATGTCTCACCGGGCTGTCATCTCTATTTTTGGGGAGCGTGGCATTGGAAAGACTACACTTGCAAAACAACTGTACAATGACCCAGATATCATAAAACACTTTGAGGTCCATGCATGGGTATGTCTTCCACCACATGTCAGGTTCAGGGACTATGTTGAGATTATGCACATGCAGGTCAACCCACAGATTCCAGGAGCTTCTGAGAAAAATGGTAATACAACATTTGCACTTGGTAACAAAGAAACCACTGATATGGAATTCAACCTTCGGCAGAACCTTGAGAACAGGAGGTATCTAGTTGTTCTTGATGGTTTAGTCAGCATTAGTGACTGGAACTCATTATTTGCTGTGCTGCCACATACCAATGCCAATGGCAGCCGGATCTTACTTACCACACATCTCAATGTGAAGGAAATCAATCACATCGACCCACAGATAGCTCCTGTCAAGCTTCCTTATCTTGACGAAAAACATGGAGAGGAGTTATTTTGTCAAAGAGTTTTTGGGACAATAGAACCTCCACAAATTTATAAGAGCAAGGGTTACTATAAAAAAGTTCACAATATATCAACAGGTCTACCCCTGGCAATTACTGTGCTTGCAGGAATATTACGATCAAAGTTTATCCCCATGGAGTGGGATGTCATATTCGAACAACTCGAGTCCAATGGCCAGCCAAAACCGGTTAGAAGCATATGGTCTTTGGCTTTTGATGACTTGCCACACTACCTCAAGTCATGTTTCCTATACTTGGCGTCCGTTTCAGAAAATGTTATTCTTTACCCAGATCGTTTGGTGCGTCTGTGGATTGCTGAAGGTTTTGTTATGCCCAAGAAAGCAGAAACACTGGAGGACGTTGGGTTTGACTATCTAAAAGAACTGGTCGCGAGAGGGTTAGTCCAGGTCGTGCAGAAGGATGCTGGTGGATCCATCAAGCTGGTAGCCATCCACAATCTGCTCCATGCTTTTGTGGAGTCTGAAGCACAGGACTCTAGTTTCCTTGAAATCCACCATCATGCTAATGTTGTAAACCCAAATGCAGTGCGGCGCCTTGCCATACAAAACTATGTGGATGCATATGTCCACATTCCTAATGTGTTCCCCAAGCTGCGTTCGCTTCTCTGTGATTTTGCAGAAGACCAACAACGCGGTAGCTCAAGCTCTGGAGAGCTGCAACCTCAGTCGTTATGGGGCAATCTTGCAGAGTTGTGTTCAAGAGCCTGTGGCACTTCGGAGAATGTTGGCTCAAGCACATTGCATGGGCTCCACTTCTTACAGGGCTCCAGGTACCTACGAGTTGTTGACCTGTATGGTCTTAAGACGCCAAAGCTGCCAGATGAGATCGGCAGCATAATCCACTTGAGGTACCTTGGCATTAGGAACAGCAACCTGGTGGAACTCCCCTCATCCATCTACAAGCTTGACAGTCTCCAGACGTTGGATGTAAGGAAAACAAACGTGGCGAAAGCCGTCGATGAGTTCTGGGACATCGAAGCACTACGGCATGTGCTTGCCGAGAAAATGATTTTGCCCGATTGCTCAGTAGTTCCCTTGAATAATTTGATGACACTCAACGGTGTGGTGCCTTGTGGTCCATGGGATGAAAAGAGCTGCCCCTTGAACAGTATGATTTATCTCCGATCCTTGTCCCTGTCTGACATTTCAGCAGCCCATACCGCCGCGCTTTCAGCAGCTCTGAGGAAAATGGAATTCCTTTTGTACCTGAATCTGTCAGGTGAATTTCTCCCGTCCAGCATGTTCACCAGCTCAAGCATGCGCCGTCTCCAGACCCTCATCCTGCATGGTAAGTTACAAGGGATTAATGATCTGCCGAGCGACGACCGCTATGTCCTACCAAACCTTACCATGCTCTATTTGCATGGATCTGAGGTGTCTCAGCAGTTTGTGCACAAGCTTGGTACGCTGCCGTGCCTTGTTGAGATGGAGCTGTCAGCTGTTTCGTACAGTGAGGGTGAGATGAAACTGTTCTTGGACGGATTCCCAAGCCTAGCAAGGCTGAAGCTCAAGAACGTGTCTATGTTACAGGAGGTAGAGATACGTGGAGGGGCCATGCCAATGCTTTCCATCCTAGCCATGTATGATTGCGACAGCTTGAAGACCTTCAAGAACCTGAATAGCTTGGAACACCTTCAAGAAGTGGCGATCTACAATACGCTGCCGGAGATTGTTGATAACATAAAGCTTGAGGACGAGAAGCTTTTCAGCAAGATCAAACGCCTGACTAACCCCATGATGACTGCAGACCGAGACTTTCCAGGTCATTTTTTAAGAATGAGAATTAAAATGTTGGATGAACCGGCTCATGATCTACGTGTCGCAGTGGCCTCTGAACCACATTGCAGTGACATGGAGGGTGATGATCATGGTGAAAGGAAAGCAACAGACCAAGTACACTACGGCCCTGGTCTGTTATAA

>B97_Zm00018ab039170

ATGGCTGACACGCTGCTTGTCCCCGTGGTGGCCAGGGTGGCCGGCAAGGCCGCCGACGAGCTCGTCCAGAGCGTCGCCCGCACGTGGGGCGTCGACGCCGACCGTGCCATGCTGGAGCGGACGCTGCTGGCGGTGCAGCGCGTGCTGCCTGACGCCGAGGCGAAGGGCGAGTCCAGCCCCGTCGTCCGGATGTGGATGAGGGAGCTCAAGGCCGTCGCCTACCGGGCAGACGACGTCCTCGACGACCTACAGCACGAGGCTCTACGCCGTGAAGCTAGCGAGCGTGAGCCGGAGCCGCCCATGGCGTGCAAGCCGACGAGGCGGTACCTGACCCTCCGCAACCCTCTCCTGCTCCGTCGTCTCACCGTGAGCAGGAGTCTGCGCAAGGTCCTCAAGGAACTGAACGGTCTTGTCCTGGAGACGCGCGCTCTAGGCCTGGCGGAGCGCCCGGCGGCGCGACACCGACACGCACACGCTCCGTGTCAGCAAGTGCGGGTAGCACTGAACGGCGGGTCGGCTGAGATCTTCGGCAGAGACGGTGACAGGGACGAGGTAGTGAAGCTGTTGCTTGACCAGCGACACCACCAAGACCAGAAGAACGTGCAGGTGCTGCCCGTCGTAGGGGCCGGTGGTGTGGGCAAGACGACGCTGGCAAGGATGGTGTACACGGACCGCAGGGTTCAGAAGCACTTCGAGCTGAGGATGTGGCACTGCGTGTCCGGAAACTTCGGAGCCGCCTCTGTCGTGCGATCCGTCGTCGAGCTGGCTACGGGCGAGAGGTGTGACCTGCCTGACGCCGGCAGCGTCTGGAGAGCGCGGCTCCAGCAAGTCGTTGGCAGGAAACGGTTCGTGCTCGTCCTTGACGACGTGCGGGACGACGAAGAGCGGGAGAAGTGGGAGGGCGAGCTCAAGCCGCTGCTGTGCACGTGTATCGGTGGGTCAGGGAGCGTGATTCTTGTCACTACTCGAAGCCAGCAGGTGTCTGCTGTGATGGGCAGCCTTCCGACCAAGGAGCTAGCACGCTTGGCTGAAGATGATTCATGGGAACTGTTCTCGAAGAAAGCGTTTAGCAGAGGAGTACAAGAGCGACCAGAACTGGTCGCTATCGGCAGGCGCATCGTCCACGTGTGCAAGGGGCTGCCTCTTGCTCTGAGCACAATGGGTGGCCTGATGAGTTCGAAGCAAGAAGCTCAGGACTGGGAGGCCATTGCAGAAAGCTGTAGTAGTGATACTGATACCAGTACAGGCTCAGGCACGGATGATGAAGTGTTGTCCATGCTTAAACTGAGCTACAGACACTTGCCAGATGAGATAAAGCAGTGTTTTGCGTTCTGCGCAGTGTTTCCCAAGGACCATGAGATGGAGAAGGACAGGTTGATCCAGCTATGGATGGCGAATGGCTATGTTCGTGGAGAGGGAACTGTGGATTTGGCGCAGAAAAGCGAATTCGTTTTCAGCGAGCTGGTCTGGAGGTCCTTCCTTCAAGATGTGGAAGGGAAGGTGTTCTGTAACTCGCTTCATGAGACGGTTATATGTAGAATGCATGGCTTAATGCACGACCTGGCAAAAGATGTCTCGGATGAATGCGCGTCTTCAGAAGAGTTGGTTCGAGGAAAAGCAGCGATGGAAGATGTATATCACCTGCGAGTGTCATGGCATGAGCTGAATGGAATCAATGGGGGCACGCCATCTCTCCACACATTGTTATTAACTCAGTCAGAGCATGAGCATGATCATCTTAAGGAGCTGAAACTGAAGTCAGTGAGATCGTTAAGCTGCGAAGGTCTCTCTGCCATCCATGGCCGCCAGCTTGTAAACACGGCGCACTTGCGGTACCTTGATCTTTCCAGGTCAAAGATCGTTAGCCTGCCAGATTCACTATGCACGCTGCACAACCTGCAGTCGCTGTGGCTCAATGGATGCTCCAGGCTACGGTACCTACCAGATTGTATGTCAGCCATGAGGAAGATCAGCTACATCCATCTCTTGGAATGCGACAGCTTGGAACGGATGCCGCCAAAGCTCGGTCGACTGCAGAACCTTCGCACGCTGACAACGTTTATCGTGGACACTGCAGATGGATTAGGGATCGAGGAGCTCAGAGACCTGCGACACCTCGGCAACAGGTTGGAGCTGTTCAATCTAAGCAAGGTGAAGGACGACGGTTCAGAGGCGGCCAATCTCCATGAGAAGCGGAACCTTAGCGAACTGGTGCTGTACTGGGGCCGCGACCGGGATTATGATCCGTTGGATAACGAGGCCTGTGATGAAGACGAAGGGGTACTGGAATCTCTTGTCCCTCACGGTGAGCTCAAGGTTCTGAAGCTGCATGGGTACGGTGGCTTGGCTGTGTCAAAATGGATGAGAGATTCTAGGATGTTCCAGTGCCTTAGAGAGCTCGTTGTCACTGAGTGCCCGAGATGCAAGGATCTGCCGGTGGTATGGCTGTCACCTTCTCTCGAGGTCTTGGAATTATCCGGGATGATCGGCCTGACAACACTGTGCACGAACGTCGATGTGGCAGAGGCGGCAGGACGCAGCGCGTCCCGGCAGATTTTCCCGAAGCTGAGGAGGATGCGGTTGCAGTACTTGCCTGAGTTGGAGAGATGGACAGATCAGGACAGTGCAGGAGAGCCTGCTGGTGCCTCAGTGATGTTTCCCATGCTTGAAGAGCTAAGAGTATACGAGTGCTACAAGCTTGCGAGTTTCCCCGCGAGTCCAGCTCTCACGCTTCTATCTTGCCGAGGTGACTCTGGGCGCTGCCTTGTTCCTGTGAGCATGCCCATGGGCTCTTGGCCGTCTCTTGTCCACCTGGACATTGGGTTGCTGGCTGAGGTGGTGATGCCCGTAGAAGACACTCAAAGCCAAAACCAAAGGCATCTGAACACCATGCGGAGTGTGAAAGTCCTTGGCGAAGACGGCTTCGTGTCAGTGTTCAACCTGTCGAAATCTCAACTCGGGTTCCGGGGCTGCTTGGCCTTGGTGGAGAAACTGGAGATCGGATCATGCCCGAGCGTCGTTCACTGGCCAGTGGAGGAGCTCCGGTGCTTGCGTCGCCTTCGGTCTCTGGATGTCTGGTACTGTAAGAACCTGGAGGGGAAGGGCGCATCGTCTGAAGAAACGCTTCCGCTGCCCCAGCTGGAATGGCTATCGATACAGCACTGCGAGAGCTTGCTGGAGATCCCCAGGCTGCCCACGTCCCTCGAGCAAATGGCAGTCCGCTGCTGCAGCAGTCTGGTGGCTCTGCCCTCAAACCTTGGAAGTCTGGCCAAGCTCGGGCATCTCTGCGTTGATGACTGTGGTGAGATGAAAGCGCTGCCTGATGGGATGGATGGCCTCGCTTCCCTTGAGAGCTTGAGCGTTGAGGAGTGCCCGGGGGTTGAGATGTTTCCACAGGGTCTCCTCCAGCGGCTCCCAGCCCTCAAGTTCCTGGAGATCAAAGCCTGCCCTGGCCTGCAGAGACGTTGCAGACAAGGTGGGGAGTACTTCGGCTTGGTTTCTTCTATTTCGAATATAGACATTCCAGCAGTAGAGTCCAACGTAAAGAAGTTTGTGAAGAAGCTCATCCCTTTCTGCTGA

>B97_Zm00018ab045310

ATGGAGGTTATGACTGGGGCACTACCGAGCCTCATCCCCAAGCTTGCTAATCTAGTCGCTGGTGAGTACAATCTGCAAAAGGGTGTCAAAGGGGATATCATGTTCCTCCAAGAAGAGCTTGAGAGCATGAGGGATGCACTTGAGGAGATCTCCAAGGTTCCAGCAGACCAGCTTCCCAACAGAGATAAGATTTGGGCTAGGAACGTGAGAGAGCTCTCCTACGACATAGAGGATAGTATTGACGCATTCATGGTGCAGTCCAAGGGTAGGAAGCTGGTTAAACAATATGGTCTCCAGAAAGTCATTGGTATGTGTCTCGACTGGTTGTTGCAGCCCAAGATTCGCCATAAAATGGCTACTGAAATTAGGACGATCAAAAGGCGCATCGTTGAGGTGCACGAACGGCATCTCAGGTATGAAATTAATCTTGGTCCTGATAAGCTTGGCAATGCTGCTGTAGATCCCCGAATATTTGGTCAGTACACAGAGCTGAAAGAGCTTGTTGGCATCGACGAGACAAGAGATGAGTTAATCGATATTATGATGGAAGGGAATGTAGTCCCCATGAAGCAAGGCAAGATAATTTCCATTGTTGGATTTGGAGGGCTTGGAAAAACAACTCTTGCTAATGCTGTTTATGAGAAGATAAGAGCATTGTTCGATTGCTGTGCATTTGTTTCAGTGTCTCAAACTCCTGACTTGAAGAAATTATTCAAGAGTTTGCTCTATGATCTTGGCAAATACATCAATGAAGAATCGTTGGATGAGAGACAGCTCATCAATGTACTCAGAGAATTCCTTCAAGAGAGAAGGTATTTAATTGTTATTGATGACATATGGGACATCTCAGTCTGGAAAATGATTAGATGTGCTTTACCAGATAATAATATCGGGTACATTATTGTTACAACCACACGCATTTCCGATGTTGCTGAAAAAGTTGGTGGTGCTTATAAGCTAAAACCTCTTTCATTGAACAACTCTCGAGAATTAATGTATAAGATAATATTTGGGAACGAAAATAGAGAAAACACTAAAGACAAAGAAATATGTTTAGATGAGGAGCTTGCAGAAGTATCAAATAAAATACTAAAGAAATGTGCCGGTGTGCCCCTAGCTATTATTACGATGGCCAGTGTACTAGCTTGTAAAGCAAGAAATGAAATGGAGTGGTATGAGGTGTACAATTCTATTGGTGTTGGCATTGAGAACAATCTAGATGTGGGGAATATGAGAAAGATTTTGTCATTCAGCTATTACGATATGGCATCCCATCTCAAGACATGTTTATTATACTTAAGTATGTTTCCAGAAGATTACAAAATTGAGAAAGATCGTTTGATATGGATGTGGATAGCTGAAGGCTTTGTCCAATATGTGAAACAAGGGAAGAGCCTATTTGAGCATGGAGAGAGTTACTTCAATGAACTCATAAACAGAGGTATGATCCAACCCATATATAACCAAGCTGGCATGATATATGAATGTCGTGTGCATGATATGGTGCTTGGTCTCATATGTTCCTTGTCAAATGGAGAAAATTTTGTTACAATATTAAATGGTTTGGGTCACGGATCTCCATCAAATATGATTAGGAGATTATCAGTTCAAAATGGCAATGAGAGTCAAGCTATGACTCTGCAAACCAGAAGCTTGCAGCAAGTAAGGTCGGTTGTTGCCTTTCCGGATGCCACCGCTAGTGTAGTTACGGTCTTGAGGAGCTTCCGAGTTCTACGTGTACTAGATTTACAAGATTGTGACCTTTCACAATGTTGCAGCCTGAAGTACCTTGGGAATCTATTTCACTTGAGGTATCTAGGACTATGTAACACAAGTATTACACAGCTCCCTGAAGAAATTGTAAACTTGCAACTTCTACAAATACTGGATGTATGGAACAATAAAATGTATTGCCTACCCTCAACTATTGTGCAGCTGAGATATTTGATGTGCCTTTACATTGATGGTTTTACTAGAGTGCCAAACGGGATTGGGAGCCTAACAAGCCTTGAAGAACTCACATTTTTAGGCATTTATGACTCCACTGTGGACATTATAGAAGAGCTGGGCCAGCTAACGGAGCTACGGGTACTGCATATCGTTCTTTTTGGTGAATGGAACGACAAGCTGGTGGAGTGCCTGCACAAGCTGAAAAAGGTGAAATATCTATATATCAAGGACCGCAGTGGTCAACTTAACATTGGTGGTTTGGATGCCTGGGTAGCCCCTCCAAATCTCCAAATATTGAATACGCGATCGGGTTGCTGGTTCACCACACTGTCAGCATCAGCATGGATGAATCCATCTCTTCTTCAGGACCTCTCCTTCTTATCTACCACCGTGAGGGAGTTGCAGCAGGATGACATTATAATTCTTGGGAGTTTGCCAGCTCTCCGCTATCTAAATCTGATGATTGTTGGTGAGGATTTGGGAGTTCACAATGAAATCCATGGGGAATGCATTGTTGGTGCTGGTTTGTTCCCATCCCTAGTATACTGCCAGCTCTGGGGAAATGTGGCGCCTATAGTGTTTCAGCGAGGAGCCATGCCACGGCTCAGAACCCTTGACTTTCGGTTATCTGTTCAAGAGGTGAGAGGAATCAACGGCAGGGATGGTGGTATTGACTTGGGCCTCAGGAACCTGCCATCACTCCAGAAGGTTTGGGTTCATTTGGACCCTGAGGGCGCTAACAAGGCAGAGGTGAAGGAACTGGAGGCTGAGTTGAGGCGTGCCATCAAAATTCATCCAAATCATCCTCAGCTGTATGGAATTCAGGATGAAGATGCAGATGATGAATGA

>B97_Zm00018ab079680

ATGACTTTTCAAGCCGCAAGCCCACCGAACAACGTGCGCGCGGACGTTTTCTTGGCAAGGCCCGAATCCTTCCAATCCAATCCCCATCCGGGCAACCGTCTGCCGTGTTGCCCCATTCCTGCCATGCACCGCGTGGCGCCTAGCCTCCGCATATGCTTCCTTTATAAGCTTCAAACAACAACTCCCTTTTGCAGATTCCTCGTTCGCCTGCTGCACGCTCACCACTGTACGTCCCCGATGGCTGAAGCCATCAGCGCGACCAGCTCGTGCCTGGAACCCCTGTGCGGTTGTCTGGAAAGCACAGGCGTGTTCGAGGCAGTGGGCCGGGAGGTGGCCGCGTTCCTTCGCATCAAGTCGAATTGTGGCGACCTCGAGAAGGCCCGGGACAGCCTGCGCGCCGTCGAGACGACAGTCAGGGCGCGGGTCACGGCGGAGGAGGACAAGCTGAACGTCTGTGATCCTCAGGTGCAGGCGTGGCTCAAGCGCGTCGACGAGCTTCGCCTGGACACCATCAACGAGGATTACAGCAGTCTGTCGGGGTTCTCTTGCCTCTGCCAGTGCACCGTGCACGCTCGTCGCCGCGCCTCGATCGGCAAGCGTGTTGTGGACGCGCTGGAGGAGGTGAACAAACTGACCGAGGAAGGAAGGCGGTTCAGGACATTTGGGTTCAAGCCACCGCCGAGGGCCGTCAGTCAGTTACCCCAAACTGAGACCGTTGGGTTGGAGCCCATGTTGGCTCGGGTCCATGATTTGCTTGAGAAGGGCGAGTCGAGCATAATTGGTGTGTGGGGTCAAGGAGGCATCGGCAAGACGACTCTCCTGCACGCCTTCAACAATGATCTCGAAATGAAAGACCACCACTACCAGGTTGTTATTTTTATTGAAGTATCCAATTCAGAGACCCTGAACACAGTGGAGATGCAACAGACTATCTCTGATAGGCTTAATTTGCCATGGAATGAATCAGAGACAGTTGAGAAACGGGCCAGATTCCTATTGAAGGCACTGGCCAGGAAAAGATTTCTATTGCTACTTGATGACGTAAGGAAGAGATTCCGACTGGAGGATGTCGGTATCCCAACTCCGGACACGAAGAGCCAAAGCAAGCTGATCCTGACATCACGTTTCCAAGAAGTATGCTTCCAGATGGGTGCACAGAGGAGCCGCATTGAAATGAAGGTTTTGGATGATAATGCTGCCTGGAACCTGTTCTTGAGCAAGCTGAGCAACGAGGCTTTTGCAGCAGTTGAGTCACCGAATTTCAACAAGGTTGTTCGGGACCAGGCCAGGAAAATATTCTCCAGTTGTGGAGGTCTACCACTTGCACTCAATGTCATTGGGACTGCTGTGGCAGGGTTGGAAGGACCAAGAGAATGGATTTCAGCTGCTAATGACATCAATATGTTCAGCAATGAAGATGTGGATGAAATGTTTTATCGGCTGAAATACAGCTATGACAGGCTGAAACCCACTCAACAACAGTGCTTTTTGTACTGCACTCTTTTCCCAGAATATGGATCTATTAGTAAGGAACCATTAGTTGATTATTGGCTGGCTGAAGGTTTGCTTCTCAATGATCGTCAAAAGGGTGATCAGATAATTCAGAGCCTTATTTCAGCATGCTTGTTGCAGACCAGTAGCTCATTGTCATCAAAGGTAAAAATGCACCATGTAATCAGGCATATGGGGATTTGGTTGGTTAACAAGACAGATCAAAAGTTTCTTGTTCAAGCAGGGATGGCTTTGGATAGTGCTCCACCAGCAGAAGAGTGGAAGGAATCGACAAGGATCTCCATCATGTCTAATGATATCAAAGAGCTTCCTTTCTCACCGGAATGTGAAAACCTCACTACATTGTTGATCCAAAATAACCCAAATTTGAACAAGCTGAGTTCAGGGTTTTTCAAGTTTATGCCCTCCTTGAAAGTGCTGGATCTTTCTCACACTGCAATAACAACACTCCCAGAATGTGAGACATTGGTTGCATTACAGCATCTCAATTTGTCACACACACGTATTAGGTTATTACCTGAGCGGCTGTGGTTATTGAAAGAGTTGAGGCATCTGGATCTCAGCGTGACTGCTGAACTCGAAGATACCTTGAACAACTGCTCAAGGTTACTCAATTTAAGAGTTCTTAATCTCTTTCGCAGTCACTATGGTATTAGTGACGTCAACGACCTGAATCTGGATTCCCTGAAGGCACTGATGTTCCTTGGAATCACTATTTATACAGAGAAGGTGTTAAAGAAACTGAACAAGACTAGTCCTTTGGCAAAGTCAACATATCGTCTGCATCTTAAGTACTGTAGAGAAATGCAGTCGATCAAAATCTCCGATCTCGACCACTTGGTGCAACTCGAGGAGCTGTATGTCGAATCATGCTATAATCTAAACACTCTTGTTGCTGATACTGAGCTGACTGCATCAGATTCAGGCCTGCAGCTCCTCACCCTCTCAGTTCTTCCTGTGCTGGAGAACGTCATTGTTGCACCAACGCCCCACCATTTTCAGCACATCCGCAAATTGACCATTTCGAGTTGCCCCAAGTTGAAGAACATCACATGGGTCCTAAAACTTGAAATGCTCGAGAGGCTCGTCGTGACCCATTGTGATGGGTTGCTGAAGATTGTTGAAGAAGACAGCGGTGATGAGGCAGAAACAACAATGCTGGGTCAGGGTCATCCTTCTGAAGAACAGGAAGATAAACGGATTGATGGTGGTCAAAGTGTGTGCAAGAGCGATGACAATGCGCATGCTGAGCTCCTGAACCTGAGATCAATCGTGTTGACTGATGTCAAGAGCCTGAGAAGTATCTGCAAGCCAAGAAATTTTACCAGCCTCGAGACCATCCGGGTGGAGGATTGCCCGAATCTGAGAAGCATCCCACTGAGCAGCACGTACAACTGTGGGAAACTGAAGCAGGTGTGCGGTTCAGTTGAATGGTGGGAGAAACTGGAGTGGGAGGACAAGGAGGGCAAGGAGAGCAAGTTCTTCATTCCAATCTGA

>B97_Zm00018ab090200

ATGGCTCCTGTAGCAATAGGAGAATGGATGGCATCAGCCTGCATCGCCAAGATGGTTAGCAAAGTGTGCTCTTACCTAGAAGACCAGTATGAATACCAAAGGGATGATGCAAAGGATAAACTAACCAAGCTCAAGAATAATCTCTGGAAGATACCATTCGTGCTGGACAAAGCTTCAAGCTTGCAAACCAAAGATCCCAGTATGAAAAGTTGGCAAGGGAGTATAAAAGATGCTGCTTACCAGGCTGTGGATGTTCTTGATTTATTTGACTATCGCTTTTTTGAAGCAAAAGCTGAAGACATAGAAAAGGTGCTGGCCGAGTCATCTGATTATGCTACTGTTAACTCTTCATCCTCCGCAACAACCAACACCACTGCCTCTACCAGCAGCAGCTCAACAGTAAAACGGTCAGTTCGTGTTCTGAAGCGTGTCCTTTTTTCTGATGAAGACCTTAATAAGTTAATTGCAATTTTGGAAAAGTTTGATAAGATTTCTAGTGAGATGCAAACATTCTTAGAGCTTGTTAACCCAAGGAATAAGAAGCCAGGAAAGGCACTTCAGTGGCGCAGAACAACCTCTATGCTAGGTACCACAAGATTGATCGGTAGAGGCGATGAAGAAACACAGCTAAAGAAATTACTAGAACAGACAAATGATTGGTGCAGAAAACCATATTCTGTAATAGCAATAGTTGGAGTCGCCGGCGTTGGTAAAACTGCGCTACTGCAGAGGGTATACAGCCATTTTCGTGATATAGGACATTTTGACATCATGGCATGGCTCTATGTCTCAGAAAAATTTGGTGTCAAACGCCTCACAAAAGAGATGGTACAGTCACAGAAATGCCGTAGGCATAAAAGGAAAAGAGGTGGAACCAGCAGGGTTTCATGGGATGGCTCCATATCAGCTGACTTGAACAGCATCAGTAACTTAGATCTAGTTCAGAGAATACTTGAGAAAAAGCTAAATGGGAGCAAGGTTTTGGTAGTACTCGATGATGTTTGGAACGAAATGAGCAGCAAATGGGAAACATTGTGCAAGCCCCTCCAGTTTGCTAGTATGGGCAGCAAAGTGGTGCTTACTACTCGAAGTGAAAAGGTTGCAAAGATAAATGGAGCAACAGAGATAATACATTTAGATGGGTTGAAAGGCAAAGAGTATTTGGATCATTTCCAGCAATGCGCGTTTGACAACGCAACACCGTCAGATTTTCCAAGATTGGTGCAAATTGGTGAACAATTGGCAATGAAATTGGCTGGTTCACCACTAGCAGCTAAGACGGTAGGAGCTGAACTGAAACTGAAGCTACAGGAAGACCACTGGAAGGCTGTCCTTCAGCTCAAATTGTGGCAGATTGAACAGACAGCAGATGATATTATGCCAGCATTACGATTGAGCTATGAGCATCTTCCAGATCACTTGAAGCAGTGCTTTGTTTACTTTGCATTGTTTCCTAAGAACTACCAACATCGGGATGATGTGCTCATACAGATGTGGCGAGCCCATGGTTACATTCAAAAGGAAACATCAGATGAAAATGCATATCGTTATATTAATGATCTCTTACAGCTTTCATTCATTAAGAAAGCAGCCAATCTAGATAACCATTATGTTGTTCATGACTTGCTACATGATTTGGCAGAATCAATCTCCAATGGAGAACACTTTCGAATTGAAGATGATTTTCATGTTAGTATTCCAAGAAATGTGCGGCACCTATATGTCAATGCAAGCAATATTTCTAAGGTGTGCATGAGTTTGGTTGAATCCCAGGAGGGATTGGCAGAATCCCCGGATTTGAAGAAAAATCTAAGGAGCCTAATAATATGCAAGCACCATGCTCCTGGAGAAAGAATCCCTCCAGATAACTTCAACGATGTTCTTAAAGAAACGCTGCATGATCTAAGGAGCTTACGTGTGCTCGTACTACAACATCCAGATGGTATTCTGCCAGATAATATTGAACATCTAGTCCACCTTAGGTATCTCGACATAAGTGAAAGCAAGATATTCACCAGTATCCCAAAATCATTATTCAGATTATACCATTTACAGGGGTTTATTCTCCAGTCATATTGTCAGCATAATCTAGGGAAAGAACTGCAGAAGCACATTAGTAGGCTGACAGCTGAGCCAGTTAAAATACTTAGCCCCATCCAGAAGTCGAGGCATCAAAATGATAGGTAA

>B97_Zm00018ab092070

ATGGATCTTGTGGCCGGCGCCGTGGGCAGCATCATCCGCAAGCTCGGCGAGCTGCTCCTGGCAGAGTACCAGCTGCAGGCGTGCCTGCCGGAGGAAATCGAGTCTCTGAAAAATGAGCTCGAGAGCGCGCACGTGGCTCTCCGCACCGTGGCGGAGGTGCCGCCGGAGCATCTTGATCAACAGGTCCAGCTCTGGGCTCGCGAGGTCAGGGAGGCGTCGTACGACATGGAGGATATCCTCGATACCTTCCTCGTCAATGACGCACCGGCTGAGAAAAAGGATGGCCTCGGCAAACGTCGTCGTCTTCGTCGTCTCCTGGACAATATAGCTGGCGCCATGGAGAAGATGAGGAAACTGTTCAGAAAGAGCAAGGAGTGTCACACCATAGCTGGCGCCATAGAGAAGATGAAGGAACGGCTCCGGGAGGTGGCTGACCGCCGCGACAGGTACGCCGTTCCGGTGGCAGCGCCTGCGCCGGCGAGGACGCTGGATCCTCGCCTTGCATACATGCACAGGGAAGCAGCACAGCTGGTCGGCATGGACAGGACCAAGGCTGAGGTCATGGCCATGCTTCTGCCGCTGCCGTCGTCCCGCTGCCCCGAGGACGACATCGACGTCTCTGCCAGCGGCGGAGACAAGATGAAGATAGTTTCTGTGGTCGGAGCTGGTGGCCTGGGAAAGACCACTCTTGCCAAGGCCGTCTACGACGAGCTCAAACCGCGATATGACTATGGAGCGTTTGTTTCGGTTGGCCGAAAACCTGATCTGGTGCAAGTGTTTACCAACATCTTCTTCCTTCTCGACAGACAGGAGCACGCGGCCATTCGTGAAGTAAAGGACCTACAGCTGTTGGCCGACGGACTACGAACATTTCTACAACACAAGAGGTACTTGATCGTTATCGACGATGTTTGGGATACAGAATCTTGGGAAACAATCAAATTAGCTTTTGATCAGAAGAATAAGAAGAGTAGGGTAATCACAACCACTCGCAACCGACAAGTAGCTTCCGGCGAGAAGGTTTACGAGCTACATCCGCTCCCTCATGACAGCTCGAAGAAGCTATTTTATATGAGGCTGTTTGGGGGTGAGGACAAATGTCCGGCTAATCATCCTGAAGAGGCGTCTCAAAGGATTCTTAACAAATGTGGCGGTGTACCATTGGCTATCATCACAATGGCAAGCTTGCTGGTGGGTAAATCGAGAGAAGACTGGTTGGAGGTGTGCAGCTCTCGCGGTTTCTACCACGGCGGCGGTAAAGATAACAACAAACAAGTAGATGACACCGTGTGGATACTGTCTCTGAGCTATTATGACCTACCTTCATATCTGAAGCCTTGCTTACTGTACCTAAGTGTGTATCCAGAAGACTATGAGGTCGAGAGGGAGAGATTGATATGGAAGTGGGTAGCTGAAGGTTTCATCGAGAAGAAAGCAGGAAGCAGCAGCAGCCTGTTTGAGCAGGGAGAGGAATACTTCCATGAGCTCATAAACAGAAGCATGATCCAGGCGGTGGGGGACGACAAAGAGGTTGCTGACGCCATATTTGGTTGTCGTGTTCATGACATGGTGCTTGATCTCATCCGTGACATATCAAACGAAGAAAACTTCATCACTGTCTCATACGATGATGGTAGAAGAGGCGCACGGTCGTCGTCGTCGTCGTCGTCGTCGTCACGGCACGTGGTGCGCCGGTTAGCTCACCAAAACAGAAGAATAACGGAGGAGGACAACCCTGTGGGGGGCAGCATGAGGTCACTTGTTGCCTGTGGGTGTGATATGGATGGTTGGGCCTTGCACTCGAGCTCTAATAAGCTGCTGCGTGTGCTAGCTTTAGAGGAATGCACGCCATCTATGGACATCGGACATCTTGGAAAACTGCTTCATTTGAGGTACCTTGGGTTACATGGTACTAGCATCAAGACACTCCCAGAGGAAATAGGATCCCTCAAGTTTCTGCAAGCACTGGATTTAGAGGGCACTGAAATATCACGTCTTTCACAGACTGTTTGCCTGCTAACACAGCTGATGTACCTACGGGGTGCAACCGGGGTCACAACAGTGCCTGATGGTTTCCTGGGGCAGGTGACGTCACTAGAGGAGCTCCATATATGTCTTCCCACCAAAGATGACGAGTACAGCCAGAAGAAGTTCATGCAGGATCTGGGCAAGCAGGGAGAAATCAGGATTCTCGTTTTGTATGGGAACAGAATTGAGTTGGATCCGTGGATGCAGTCCAGTCTAGTGCAATCACTAGGCGGTCTGTACAAGCTCCAGACCCTAGTGGTGAGGCATTATGCAGATGGGGTAGCAGCAGCACAGGGCAGCTGGGACACGGCGAAGCTTCGGCGACGTCTCCGGATTTTGAACTTAGATGTCCTCCGGTTCCATCGTGTACCATCGTGCATCGATCCCGCGCGCCTCCCCAACCTCTCACACCTACAACTGCTTGTGGTTCATCTGGACGAGGCAGGTCTGAGAGCACTGGGCGGCCTGCCAGAGCTCACCTACCTCGCGCTGTCGTTGAAGCCTCGTTCGCTGAACAGCACATCATGCAAGGCTACGGTAGCTGATGTTGTTGCCGCAGATGGCTTCTTCCTCAAGTTGAGATCGCTCAAGCTGTATGGCTGGATGGTTCAGTTGGTGCCCAGCCAGGACTCGGCAAGTGTTTCGTTCAGCATCTGGAATGAAGGAGCGGAGGTTGTGGCCCTTGGTTCCACGAGAGACTGCACCGCCGGGAGGGTAGCACCTGCTATCATGCCAGACCTCATACATCCTGACATCCAGACCTGCTATCACGAGAGACTGCACTACCGGAATCAGCTTCTTTGCCGTGTGTTC

>B97_Zm00018ab092140

ATGGATCTTGTGGCCGGCGCGGTGGGCAGCATCATCCGCAAGCTCGGCATGCTGCTCCAGGCGGAGTACAAGCTGCAGGCTGGCGTGCCGGAGCAAATCGAGTCTCTGAAAAATGAGCTCGAGAGCGCGCACGCTGCTCTCCGCAACGTGGCGGAGGTGCCGCCGGAGCAGCTTAATCCACAGGTCCGGCTCTGGGCTCGCGAGGTCAGGGAGGCGTCGTACGACATGGAGGACATCCTCGACACCTTCCTCGTCAACGCCGCCCCGGCTGATGGCCTGGGCAAACGTCGTCTCCTGGAGAAGATAGGGAAGTTGTTCAGAAAGAGCAAGGCGCGCCACGATGTCGCTGGCGCCATGGAGAAGATGAAGGGACGGCTCCAGGAGGTAGCTGACCGCCGCGATCGGTACGCCGTTCCGGTGGCAGCGCCAGCGCCTGCGAGAACGCTGGATCCTCGGCTCGTGTTCATGCATAGGGAAGCGGCGCAGCTGGTGGGCATCGACAAGACAAAGGCTGAGCTGATGGCCATGCTTCTGCCGCTACCACCATGGTCACGCTACGGCACAGATACCGAGGACGACGTGGATGTCTCTGCCAGCGGCGGTGACAAGATGACGATAGTTTCTGTGGTCGGAGCTGGTGGCCTGGGAAAGACCACTCTTGCCAAGGCCGTCTACGACGAGCTCAAACCGCGATATGACTATGGAGCGTTTGTTTCGGTTGGCCGAAAACCTGATCTGGTGCAAGTGTTTACCAACATCTTCTTCCTTCTCGACAGACAGGAGCACGCGGCCATTCGTGAAGTAAAAAACCTACAGTTGTTGACTGGCGAGCTACGAAAATTTCTACAAGACAAGAGGTACTTGATCGTTATCGACGATGTTTGGGATATAAAATCTTGGGAAATAATCAAATCAGCTTTCGATGAAAAGAATAAGGAAGGAAGGGTAATCGCAACCACTCGCAACCGACAATTAGCTTCCAGCGAGGAGGAGGTTTACGAGCTACATCCGCTTTCGCATGACAGCTCAAAGAAGCTATTTTATATGAGGCTGTTTCGGGGCGAAGACAAATGCCCGGCTAATCATCCTGAAGAAGCATCTAAAAGGATTCTGGACAAATGTGGTGGTGTGCCATTAGCTATCATCACAATGGCGAGCTTGCTGGTGGGTAAATCGAGAGAAGATTGGTTGGAGGTGTGCAACTCTCCCGGTTTCTATCGCGGTGGTAAAGATAACAAGCAAGTAGATGACACCGTGTGGATACTGTCTCTGAGCTATTATGACCTACCTTCTCATCTGAAGACCTGCTTACTGTACCTAAGTGTGTATCCAGAAGACTATGTGGTCCAGAAGCACTGCCTGATATGGAAGTGGGTGGCTGAAGGTTTCATCGAGAGGAAAGCAGGAAGCAGCAGCTTGTTTGAGCAGGGAGAGGAATACTTCCATGAGCTCATAAACAGAAGCATGATCCAGGCGGTGGAGTTCAAAGAGTCGTCTGGCATCATATATGGTTGTCGTGTTCATGACATGGTGCTTGATCTCATCTGTGACATATCAAAGGAAGAAGACTTGGTCACTGTCTCATCATACGATGATGGTGATGGACGAGGCACACCGTCGTCACGAAGCGTGGTGCGCCGCTTAGCTCACCAGAACCGAAGAATAACCAAGGAGACGACGACTCACCAGGACAGTCGCAGTCCTACGGAGGAGGGCGCTAAGCTGAGGTCACTGGTTGCCTGTGGGTGTGATATCGATGGTTGGGTCTTGCACCCGAGCTCTAACAAGCTACTTCGTGTGCTAGCTCTAGAGGAATGCACACCATCTACGGACATGAGAAATCTGCTTCTGTTGGAAGATTCAGAGATAGAAAGGTTTACTAAGAGTTGGGACGGTCTTGGCCGCGATCTCGGAAAGCTGGTCCATCTGAGATACCTTGGGCTACGCGGTACTCGCATCAATAAGCTCCCAGAGGAAATAGGGTCCCTGAAGTTTCTGCAAGCACTGGATTTAGTAGGCACTGGAATATCACGGCTTCCACGGACCGTCTGCCTGCTAACGCAGCTGGTGTACCTGCTGGGTGACAGAGGCACGACAGTGCCTGATGGTTTCCTCGGGAAGGTGACGTCACTGGAGGAGCTGCATATACATCCTCCTACCGAAGATGAAGAGTACAGCCAGCAGTTCATGCAGGATCCAGGGAGAAATCAGGGTGCTCAGTTTGAGGGGGGTCGTAGATGGGTTCGATGA

>B97_Zm00018ab094110

ATGGAGTTTGCTACGGGTGCCATGGGTACCCTCCTCCCCAAGCTGGGCATGCTGCTGCAGGAAGAACTCCACCTGAAGAATAATGTGAAGGAGGGGATCAAGAGCCTCACTGCCGAGCTTGAGAGCATGCAAGCTGCACTTGTGAAGGTGTCTGACGTGCCATTAGACCAGCTTGACCCAAACGTCAAGATTTGGGCTAATGAAGTCAGGGGGCTGTCCTATGATATTGAGGACAGACTCGACTCCTTCAAGGTGCGCATGGAGGGTCTTGATTCAACCAAGCGCAAAACCATCATGGGATTCATCCAACAAACCCGTGGCTTGGTCACCAAGTTCAAGATTCGCCATGTAATATTTGATGACATTAAAGACTTCGGGAGCCAAGTAAAGGAGGTGAAGGAGCGGTATGACAGGTACAAGGTGCATGATGTTGTAGCTAATCCTATCGCAACCACAGTTGACCCTCGTCTCTTGGCTATGTATAACAAGGTTTCCGACCTTGTTGGCATTGACGAAGAAGCTAAGGAGATAATGAATAATTTGTTTGAAGATGGTGACGAGCCAGCGAAAAAGATCAAGACAGTCTCTGTTGTTGGATTTGGAGGACTCGGCAAGACTACTCTTGTTAAAGCAGTCTATGACAAGGTTAAGAAGGAGTTTGATTGCAGTGCTTTTGTATCAATAGGTCAGAAATGTGATCTCAAGAAAGTTTTCAAGGACGTTCTTTATGATCTTGACAAGCAAAATCATGAAAATATCATTGCATCAGAAATGGATGAAAAACAACTCATTGATAAGCTACAGGAATTCCTTGCAGACAAGAGGTACTTGGTTGTTATTGATGACATATGGGATATATCAACATGGAAGCTGATTAGATGTGCTTTGGTGGAAAGTAACCCTGGAAGTAGAATAATCATAACTACTCGCATTTGTGAAGTTGCCAAAAAGGTTGGTGGTGTTTACAACAAGAAACCACTCTCTCTTGATGACTCCAAGACATTATTCTATACTAGAGTATTTGCTGGTGAAAGCATGAGTCTTGATAACATATCTGGTGAAGTGTGCAACAAAATCCTAAGAAAATGTGGTGGTGTGCCATTGTCCATCATTACGATAGCTAGTCTGCTTGTTGGTAAACAGAGGGAGGACTGGTCTAAGGTGTATGATTATATTGGTTTTGGGCATGAAGATAACGAGGTTATTGGGAACATGAGAAAGATATTAGCTTTCAGCTATTACAATCTACCTCCTTATCTAAAGACATGCTTATTGCACCTAAGCATATTTCCAGAAGATCACAAGATTGAGAAAAATTCATTGATATGGAGGTGGATAGCTGAAGGTTTTGTTATTGGCAGAGAAGAACTAGGGTTATTTGAGGTTGGAGAGAGCTATTTCAATGAGCTCATAAATAGAAGCATGATCCGGTGGATAGAGCTCTCTAGTAGAAGCAAGATTCGAGATGGTTGTGGTATTCATGATATGGTGCTTGATCTTATCCGCACTTTGTCAGGTGAAGTAAACTTGGTCACGGTATCAGATGTGGAGCAGCAGTGTACCACATCATCATCATATTCACCAGTCAGAAGCATTAGCGCTCGAAGATTAGCCTTCCACAAAAAAAGAAGCATTGAACACAACCCTGGCACAGAAATAGGACAGGTGAGATCATTCAATGCTTTCAACTGCTCTGGTAGTAGGATGCCCCGACTTTTAAGCTTCAGGGTCTTACGTGTACTAGCTCTGGAGAACTGTAATTTCTCAGCAGGAAACTGTTGCCTTGGAAATATTGGCAAATTGCATCAGCTGAGGTACCTAGGGCTAGTGGAGACATCCATTCGTGACGACTTGCTGCCTGGAGAAACAGGACGCCTCAAGTTTCTGCAGACACTAGATGTAAGACGAAGTGGCATAAAAATATTGCCAGCGTCTGTTGGTGAGCTAAGGAAACTGATGTGCCTGCGTGCTACCGAGGGCACAAGGATGATGGCCGAGATTGGGAAGCTGGCGTCACTGGAAGAGCTTGAGGTACACTCTGTGGACAAGTCGCCAAACTTCGCCACGGGGCTGGGGCAGCTAACCAAGGTGAGGGTGCTTGAGATCCATTTCGACGAAATGGACGAGAGCACAGAGAAGGCTCTCATGGAGTCCCTGCGCAACCTGCGGAAAATCCAGAGTCTGCAGATATGGTCCAAGAAGGAGAGGACAATTGACCTTGGCGGCTTGTTGGAAGACTGGACGCCAACCCCTTCAGATCTCCGTCAACTGATGCTGTGTGGCATCCATTTGCCCAGGCGTCCGTCATGGATTGATCCCTCATGTGTCCCGCTCCTCTCGTACTTGTCGCTCACGGTCCAGGCCGTGCAAGTGCAGGATCTAGAAATCCTCGGGAGGCTGCCGTTGCTCAGCTACCTCTACATCTGGAGTGAGGGCATCAACTGCTTATCCTATACTGCTACCAGCAGAGACGAGTTTCAGAATCTGAGACATCTGGACACAAACCTGGAGATCATGTGTGGACAGCAGGGAGCACTGCCTATGGTTGAGAAGTTGACATGCCGTGCCAGCATGGGGAAGTATGTTGCCTTTGCCAGGAGCAGCATGCCTTTCGACGATGGCAGCGTGGTGAATCCTGCTACTGTTGCTGAGGCAGAATTGCCAGTAATTCTACCTTTGGACATTGGCTGGCCCGTGAACATGCCTTGCCTCCGGGGCATCACCTATTTGCTGGATTACCAGGACTGCAGTGCCAAGGAGTGGGCTCATGTGGAGACATTGCTCTTGCACGTGAGAAAAATCCACCCCAACTGTCCACCCTTCCGAATCAAAAAGAACTGCAGAGACAAGAAGATGACCTTGATCGACGCATTCAGCTACCTTGAAGCTGTCAAGGATGTGTTCAAGGGCAACCCCTCCAAATACTCGGAGTTTCTTGATCTCATGATTGATTACAAGAGAGACAGAATCAAAATCAAAGATGTGATCATCCGTCTCAAGACCCTATTTACTGGACATGATCCTAATCTCATCCTTGACTTCAGCGTCTTCCTGCCCAGGGAGTGGGCCATCACTCTCGGGGACCTGTAG

>B97_Zm00018ab113130

ATGGCTGAACTAGCGATTAGCCTGGTGGTCGCCCCACTGGTGTCCCGGTTGAAGGAGAAGGCGTCCAGCTCTCTCCTCGACCAGTACAACGTGATGGAGGGTATGGAGAAGCACCACGAGACCCTCGTGCGCTGGCTGCCTCCCATCCTCAAAGTCATCACCGACGCCGAGAGGCAGGCATCTCGCCGCGGCGTGGAGAAATGGCTCGAGCAGCTCAAGACAGCGGTGTACGAGGCGAACGAGGTCTTCGACGACTTCGAGTACGAGGCGCTCCGCCGTCGAGCCAAGAAGAATGGGCACATCGCCGAGCTTGGCGTTATGACCAGTGTAAAACTCTTCCCTACCCACAATCGTGTTGCCTTCCATATCAGGATGGGCTATAGGCTTCGCAGAGTTGTTGACACCTTCAAGGACCTTATAAAAGAAATGGACACCTTTAGATTCAACAAGCTTGAGCCCGAGGCAACAACGGCACGGAAGGAGTTGCGTGAGATGGATTCCATTATCGTTGATCCTGAAAATATTGTTGCCAGGTCTAGAGACGATGAGAGGAAGAAAATTGTTAACATATTGGTCAATGGCCATGTTAACAGTCGTGATCTCATGGTCGTTCCCATTGTTGGAATGCCAGGACTAGGCAAGACCACCCTCGCTCAGCTCATCTACAAGGACCCTGAGGTCAAGGAGCATTTCCATCTACTAAAGTGGGTAACTGTGTCAGATGATTTTAGTGTTCTTAATCTTGCCAACAAGATATGTAATGCCTCAGAGAGAGTCCTCGAGGATGCAGTGAAGAAGCTTCAGGAACATCTTAAAGGAAAGAGGTACCTTCTTGTATTGGATGACGTCTGGAATAGGGATATTGATAAGTGGAGAAAGCTGAAGGCATGTCTTATGCAAGGTATTGGTTGTGCCATATTAGTGACGACACGTGAACAACAAATAGCCCAGTTTATGGGTACTGTAGTTGACAGCTCATGGGCAAAAAGTTACCATGAAGTGGCAATTTTGGGCAAGGAATACATACAAGAAATTATTGAAACAAGAGCATTCAGTTTGCCGAAGAGCAAGTCAGATTATTTAGTTAAGTTGGCTGGTCTGATCACTGAGAGATGTGCGGGGTCTCCATTGGCAGCAAAAGCAATAGGGTCTGTACTGCGTAACAAGACCACTGATGGAGAATGGGAGGATGTGTTACAACGAAGCACCATATGTAATGATGAGACTGGAATTTTGCCTATACTCAAGCTTAGTTATAATGACTTGCCAATTGACATGAAGCAATGTTTTGCCTTTTGTGCTCTATATCCAAAGGATTATCATATTGATGTGGACAAACTTATCCAACTATGGATGGCCAATGGTTTTATCTCGGATCAAGAAAATGAACCTGCTGAAACCATAGGTAAAAGGATTGTCAATGAGTTGGTCTCAAGGTCCTTTTTCCAATATGAGGAGCAAACTATGATCGGATACAATTCTACTACATTTTTGAAGATTCATGACCTCATGCAGGAGGTTGCACTGTCTGTTTCGGAAAAGGAGTGTGCTTGTGTAACGGATAAATTCATTACAAATAGTGAGTTGCTTCCAAGTGCTGCCCGCCACATACTTATTCAAACATGGAGTAACAAACGGATACATGGTTATTTATATGGTTTTATGAGGAAATTGTCTCGACCTATCCAAACATTGATGTTTGATGGGTCTTGTGAAGATGCCGTTGTGCAACATTTATCAAGACATAGTTCTTTGCGAGTACTTTCCATGCCAGGATTTTGGTTTCGTTTTCCAATAAAACCAAAGCATATGTGCCACCTTAGGTTCCTTGATGTCACGGGTAGTAGAATCAAAGAACTTCCATATGACATAAGCATCCTTTATAATCTTCAGACACTCAAACTTTCTGGATGCAGGAATCTTATTAGACTTCCTGAGCAAATGAAGCACATGAGTGCCCTTCGTCATCTCTACACAGATGGGTGCACAAGGTTGGAGTGCATGCCTCCAGATCTTGGACAAATCACCTCGCTTCGAACAATTACATGGTTTGTAGTGGGAAGTGGCTTGAGTTGTAGTAGCCTTGGAGAGCTAAGGGATTTAAATATTGGTGGCTCATTAATGCTAAAGCAGCTCGAAAATGTGACAGGGAGAAGAAATGCAGAAGCAGCCAAACTTGAGAATAAGAAGGAACTGAGACAACTGTCACTAGAGTGGACAAGTGGTAAGGAGGAGGAACAACAGTGTCATGAGGTGCTAGAGAGCCTTGAAGCTCATGATGGACTGTTGGCTCTAGAAATATATTCCTACCAAGGCACCCGTTTTCCATCTTGGATGGGTATGTTGAAAAACATACTCGAGCTTCGGTTGTTCGATTGCTGTAAAGTAGAGCAGCTTCCACCACTATGTCAACTAGCAGAACTGCAACTCCTTCATTTGAAAAGATTGGGAAATTTGCGGTCCCTGTGTAGCAGATGTACATCCTCCACATTTGGAAAGCTTAAGGATCTTAAGCTAGTTGATCTTCACGTTTTTGAAGGATTTTGTAAGACAATGCATGGATCTACAGTAGCATTCCCTCAGCTTGAGATATTGCACATCGAGCGCTGTGGAAATCTAGCAGCTCTAACAGAAGCATCGCATTGCGGTGGAGATTATACAGTGGCCCGCTCAACATTTCCAGAACTGAAGAGGCTCATATTGGAAGATTTGTGTAGCTTTGAGAGATGGGTGGCTGGCCTGCTTGAAATTGAAGAAGAACATGCACTATTCCCTGTGGTTGAGATTGTTGTTATTAGTAAATGCCCAAAGTTAACAACTGTGCCTAGGGCACCGAAGGTCAAAGAACTAGTTTTGCGTGATGTACATGCACATATCTCTCTAGGAGGGATCAGATGTATGACGTCATTGTCCACTTTGCTTTTGGACGGCGTGAAGCTTGATGTTAAGGAGAGATGGGACCATCCATCGTCCGTGGTAGATATGCAACTGTGGAGGTGCAGTTTGTTCTTCCAACCACGCGCACTAGTGATGTGGGTTTGCTATTGGCAGCTGCAAGATCTGACAATTTATAGATGTGATGAGCTTGTGTACTGGCCGGAGAAAGTATTCCAAAGCTTGGTTTCTCTGCGGAGGCTATGGATTGGCAATTGCAAGAACCTAATTGGATATGCAGCGGCGAATGTCCCCGACCAGGCAACTTCTGGAAGGAGCGAGCTTTTGCCCCACCTAGAGTATCTGGAGATATGGGGGTGCCAAAATCTGGTAGAGTTATTCAACTCCTCCCCTGCTCTCAAGAGAATGGAAGTTAGAGAATGTTGTAAGCTTGAGTCCCTATATGGCAATCAGTTGTTGGATGAAGCTGCTAGTAGTACCGATGACGTGACGGCATCCGCACATGTTGAGGAGAAGCTATCACCATCATCCCTAGAATCTCTAACAATATTGGACTGTGACAGGTTGTCAGAGGTTGTCAATCTTCCTTCGTCTCTCAGGGTAATAGATATTCAGGGTTGCTTCAAACTACGGCTCATGTCAGGGCAGCTGGATGCACTCAATACCTTAGCAATCACCAACTGCCCGGAGTTGCGATCACTGGAAACATGCATCGTAGATCTCACGTCACTGGAAATCCTCGCTCTGTGTGGTTGCAAAAGCTTGGCGTCCTTGCCTAGTTCGTGGGCAGGACGACAAGAATATTCATCTCTCCGTCAGCTTACGATTAGGGAGTGCCCAGGTATAAAATCGTTGCCTTCAACTCTGCAGCAGCGACTGGACAACGGCCTCTTGGATTTTACGAACCTAGATTCCCGTCGTCGTGAAGATCGGCCTCCACGACGGCTGCTTGGGTGTCTCTTCCTCCTGCCTTTATTTTGTCTGGTCTCAAGGGCCAAATGTTGCTTTGTCTCTGAT

>B97_Zm00018ab113410

ATGGAAGGAGCTGGGGAGGGGAGCGCCTTGACGGGGATGATGGGTCCTGCGCTCGACAAGCTCGCTAGCCTCGTTGACAAGTACACCGAGCTCAGAAACGCGAGGAAGAAGATGGAGCAGCTGAGGAAGGAGCTGATTGCCATCAACCTCGCGCTTGAGAAGCACGCGGCCATGGAGAACCCAGACGCGCAGGCGAAGGCGTGGGCGGCGGAGATGCGCGAGCTGGCCTACGACATGGAGGACAGCATCGATCTCTTCACCCACCACGTCGACCACGAACCGGCCGACACCGCCACCACCGGCGTCAAGAGGTTCTTCCTCCGGATCATCCGGAAGCTTAAGAAACTCCACTACCGCCACAGGTTTGCTCAGGAGATCAAACAACTCCACGACCTTGCCAACGAATCGTACCGGCGTAGGAAGAGGTACAGGATTGAGGAGGGCGGTTCAAGCCTCCCGCACGCGGAGATCGATCCTCGGTTAGAGGCGCTCTACGTGGAGGTGGAGAAACTCGTGGGCATCCAGGGCCCAAGCCAGGAGATCATTGGACAGCTCGTCGGCGAGAACGCAGCGGAGCGGCGGAGGGTTGTCGCCGTTGTTGGATCTGGAGGTTCAGGCAAGACCACACTTGCCAAACAGGTGTACGAGAAAATCAGGTGCCAATTCTCTTGTGCAGCCTTTGTGTCCGTGTCGCAAAAGCCCAACATGAATAGCCTCCTGTGGGAGTTGTTATCTCAAATCGGGAACCATGGTGGAGATTTAGGAATGATGGCAGTAGGATATTGCAGTGACAAACAACTGATCGACAGACTAAGATCACATCTTGAAAAGCAGAGGTATCTCGTTGTGATAGATGATGTTTGGACAAACTCAGCGTGGGAGACCATACAATGTGCGCTCCCTAAAAATGCCCATGCAAGTAAAATAATTCTGACAACACGAATCAACAGTGTAGGCCAGTTCTCCTGCACTCCAGATGAGGGTTTTATCTATCAGATGAAGCCTCTTTGCAGAAACGATTCTGAAAATCTGTTTCTGAAAAGGACACTATGTGATAAAGATAAGTTTCCTGCTCAGCTGGAGGGGATTAAAAACGAGATAATCGAGAAATGCGATGGTTTGCCACTGGCTATTGTTACTCTAGCTAGCATGTTAGCTACTAAACAGAGAACAAGGGAAGAATGGGAGAGGGCACTTGATTCAATCCATTCTACGCACAAGAAAGATAGTAGCCTGGAAGTGATGGACAAGATACTGTCTCTGAGTTACAGGGATCTACCTCACAACATGAGAAATTGCTTGCTGTATATCAGTACATTTCCAGAGGACCACACGATTTACAAAGATGCTCTAGTATGGAGATGGATGGCTGAAGGGTTTATCGCTGAAACACAAGGCTTTACTTTGGAGCAGGTTGCCGAGGGCTACTTCTACGAGTTTGTGAACAGGAGTTTGGTTCAGCCCATAACCTTGCGTTCAAGATATGAAATGCGTGGAGAAGGAGGTTGCCGAGTCCATGACATTGTACTGAACTTCCTCATCTCTCGTGCAGCTGAAGAGAACTTTTTAACTACGCTGTATGGCGCCCAGGGGGTTCCATCTTCAGACCGAAGGATTCGCCGGCTCTCTGTCTGGGACAGTCCAGAACACGCACTGGCAGTCTCTAGAGCGACCATGAATCTGTCCCATCTCCGGTCAGTTAGAATATGCAACGTTGGAGACTGGCCCGTGCCTGCTGTTCTAGACTTACCTGTCCTTCGAGTGTTAGATCTAGAGGGATGCCGTGATCTGAGGATCGTCGACCCTGACTGCATTCTAAGCTTGTTTCATCTGAGGTACCTGGGTTTCCGCAGCGCAAGTGGTGTCGTGCTACCGGCTCAAATAGGAAATTTACACCATCTGCAGACCATCGATTTAAGCGGGACTGGAGTGACACAGCTGCCAGAAAGCATTGTCCAGCTCAAGCGACTGATGCATCTTGTTGGGCAACGGCTCATCATGCCAGACGGGTTTGGTAGCATGGAATCCCTTGAGGAGTTAGGTACTATCGACTGCTGCAAGTGCCCCGTCAGTTTTGGGGAAGACCTGGCACTTCTGAGCAGGCTGAGGGTGCTCCGAGTGACCGAGTGGCTTTCGTCGGGGTCGAAACAAGTGACATGGAAACCAGAAGGAAATCTTTGA

>B97_Zm00018ab116670

ATGGCGGAGATAGTCACCGGGGCGATGGGCACTCTCCTGCCCAAGCTGGCCAACCTGATCAAGGAGGAGTATAACCTGCAGAAGAAGGTGCGGGGTGAGATCATGTTCCTGGAGGCTGAGCTCAAGAGCATGGAGGCTGCTCTCATCAAGGTCTCCGAGGCACCCATCGACCACCCACCTGACATCCAAGTCAAGCTCTGGACAAGGGAGGTGAGAGAGCTGTCCTACGACCTCGAGGACAGCATCGACAGATTCATGGTGCGAGTTGGCGATGGCAAGCCACATAGTTTCAAGGGATTCATTGATAGAAGCCTCCACCTGCTGACAAGGGGCAGGATTCAACACAGCATCGGCATAGACATCAAGGAGATCAGGAGCCGCATAAAGGATGTGAGTGAACGGCGTGACAGGTACAAGGTTGATCTGGTTCCTTCCAAGCCTGTTGGCAGAAGCATCGACAACCTGCGGCTGTCGGCTCTTTACAGAAAGGCGACAGAACTTGTTGGCGCCGAAGAGAAGAGCAGTGACCTTGTGAGAAGGCTCATGGAGGGCGACAAGGAGGCATCCAAGCAGCCAGTTGTACTGTCTATTGCTGGCTTTGGAGGGTTAGGCAAGACTACTCTTGCTAATCTTGTGTATGAGAAGATTAAAGGGCAATTTGGCTGTGGGGCATTTGTTTATGTGTCTCATAATCCTGATGTCGTCAAGGTTTTCAAAAACATGCTCTACCAGCTTGATGGAGACAAATACAGGGACATCAATCAAGGAACATGGAGTGAAGAACAACTAATCTGGGAACTGAGGAAGTTCCTTCTGCACAAGAGGTACTTCATTGTCATTGATGACATATGGAATACTTCTGTGTGGGAAACAATCCAATGTTCTTTGATGCACAATGAATGTGGAAGTATAATAATTATCACAACTCGTAATATTGATGTTGCAAAACAAGCTGGAAGTGTTTATCAAATGGAACCTCTTTCTCTCAGTGACTCAACAAAGTTATTCTGCCAAAGAATTTTTGGCAGTGAAGACAAATGTCCTCCAGATAATTTAGCTGAAGTGGCTGGTAAAATCTTACAGAAATGTGGTGGTGTACCATTAGCTATCATTACCATGGCAAGTATGCTAGCCGATAAAACTGGAAAGGAAATAAATACACATAACTATTGGTCACATGTGTACCAATCCATGGGTTCTGGTCTAAATGGCAGTACTAATGTGAAGAATATGAGAAGGATACTATCAGTTAGTTATTATGACCTACCTTCACATCTAAAGACTTGCTTGCTATACCTAAGTTTGTTTCCAGAAGACTACAGAATTAAAACAAGAGGTCTCATATGGAAATGGATTGGTGAAGGTTTTGTCCATGAAGAACAGGGGAAGACCTTATATGAAGTAGGTGAGGATTACATCGAAGAGTTAATTAACAGAAGTATGTTGGAACCTGTAGATATTGGCCGTGATGGTAAGACTGTTTCTTGTCGGATACATGATATGGTCCTTGATCTTATCAGTTTCTTGTCAAATGAGGAGCATTTTCTAACAAAAGTAGGTGAGCAACAGCCCATATCTCTTGATCTGCCTAAAAAGATCCACCGGTTATCCCTCCAAATTAGCCAGGAAGAGGAAGTCAAGCAGCTGGCTACAATGAGTTTCTCCCACGTAAGATCACTTACTGTGTCCACTAAAGTGTTCCAGTTGATGCCAAAACTTTCGGCCTTTCTGGTCTTACGTGTATTGAATTTAAAGAAATGTAAGGGAGTGAGGAATCACCACTTTAAAGATATTTGCAATATGTTTCACCTGAGATATTTGAGTCTCAATGCGGAATTTATTACTGAGATGCCACGGGAGATTCAGAATCTACAATTTTTGCAAGTACTTGACATAAGTAATCTTGGGCACAAAGTAAAGATGCCAACCATTATTCACTTGCGACAGCTACTGCGTCTTTGTTTTAGGCCGATGTGGGGCATAAGACTGCCAGATGGATTCGGAAAACTAACCTCTCTACAAGAAGTTAAAGGGATCATAACTATCAAGTCACCAAGCATGCTGCATAATCTGGGGTGTCTGACCAATCTCCGGACCTTGGCCATCGACTTTTGTGATTGGGATGAGAGCTATGAGGAACCTTTCATCCAATGTCTATCTAACCTTGTCAGCCTCAAATCCATGGAAATAAAAGGTACCATAGTGAGCAGCCTATGTTCCGAATGTGACAAATTGTACCCTGGTCCTCAACATCTTTGCTCCATTGATATTGAGTCGACTGCAGTGCCAAGATGGATGTCATCGCTCTGCTTCTTGTCTAGCATAAACATTGAACTATTAGCTCTGGGAGCACAGGATTTTCATGTCCTTGGGAGCATACCATCTCTACGTTGTCTCAGTATACATGTGAAGGAAACCAGAGATGAAAGATTGGTCATTGGCAAGTGTTATCCATTCCGGTGCCTAACTGAGATGCAAATCGATTATGAATCCATGGCGGTGGTGTTCGCACCAGGAAGTATGCAAAACCTCAAAGAACTTCATTTAGTGTTCGGGGTGAAAGAGGTAATGCATAAGTATGGTGATTGTAACTTTGGTTTGGAGCACCTCATGTCACTGGAGCATGTCTCTGTTAAAACAATGTACAGTATCATGCCCGAGGAGGTGGAGGCCGTAAAAGATGAATTTCAGAAATCCCTGGACATGAATCCTGGCAAGCCCACGTTGATAGTAGATTATAAGTATCCGATAAAAAGGAAGATTAGGTCTCATGCACAAGCAATAAGAGCAGCAATTTTGTTCGCTAATGCAGGCCGCATCCCTGCTACTGAAGGATTATAA

>B97_Zm00018ab118170

ATGGCGGCGCACCCACATGGTTCCCGGAAGCAGCAGCCTTCTCCTTCCAACAAGCAGGATCAGGAGGACACGAGGGTGGCAAAGATAGCGGAGGTACTAAGCGTGTTGGAGCCCGTTCGGGCGAGTATCTCTCAACAGCTGGGTGCTGGTGCTGCCGGCCCTCGCTGCGTCGTCGCTGCAGGTTACACTAGACGGCGCTGCTTGGCCTTCATGGAGACGGAGCTGAGCATCATTGCGGCGTTTCTCAAGACACTTAGCCAGCAACATCTGGACGTCGACACGAGGAGATGGATGGAAGACTATGAGCATGTCTTCAGTAAACTCCAGAACGTCGTCATCCATGAAGTTGATCCCTCACGCCGCACGCTGCTACGAACGCGGTTGAGAAGAAGAGCCAAGCAGTGCTTCCTACTACTCCGCAATAGATCGTATCCGTTTTCTCCTGTCATAGCACGGCTCTACTATCGCACCGAACATCCCTGCAGGTATAAGAATCTTCTTCAAGCTAGCCCCGACGACGACGACCTTGGCCTTGCTGGTGGCAGTCACACTGACACCGACCCCAACATCACGGCGACCCCTCTTCCGCTCGTTGGCATCGACCGCCCGGCGAAAAAGCTCCTCAGGTGGCTCACACCCCGGGAGGAGACGGACAAGAGCCTGAGAGTCATGTCCGTTGTTGGACCTCCGGGCATGGGTAAGACGACTCTTGCCATGGAAGTCCACAACCGACTACTCCGCAGGCGCCATGATGACGTCCTTGCCATTCCGGCGGAGGAGGACGCTACTATTTCCGTTTTCCAGTGCAACATTGTGGCTCGGTTCTCCCGGGGGTCCGACAGAAACAAGCTCCTTCTCCAGGACATCCTCTCTCAAATTTCAGATGGAGCAGCACCAGCATTGGCGTCCAGTCAGTCACAAGGCCAGACAATAAAGCTGCTGATTCACCTCGTCTCAAAACGCCTGCGAGATAAGAGGTACTTCATCATTATTGACGATATATGGAACGGTTCAGATTGGGAGAAGATCAAGGATGCATTTCCTAACAATAATCTTGATAGCAGAATACTGATTACAACACGCATCAGAAGTTTAGCATGGGCATGTTGCTCTGATTCTGATGATGGGCTTGTGCATGAGATGAGGCCTTTGAACCAGATCGAGTCAGAAAGGCTGCTTCTGACAAAAGCCTTTGGTTCCGTGGATTATTGCCGGGTGGACAAGGAGGAGGACGATGTGAAGCTGAAACCATTGTGTGATGAAATTCTGAGCAGATGTGAAGGTGTACCATTGTTCATAATCGGTATGGCGGCAGAGTTGTTTAAACAGCATTCGCCGCAGCAGCAGAGGCATAGGAAAGACGAGGAGGATCAGAAACAGATTGAACGAGCATTGTCCGCTGCTTATGATGACCTTCCTTATGTGTCCAAGTTAGCATCCTTATGCATGAGCATGTTGCCTTCTGGTTACAAGTTCGAGAAGGATCGTTTCTTCTTCAAAAGATGGAACGAAGTCGAAGTGGCGATGCAAGGTAACATGTGGGAGCTAATGGATAGGAATGTCATCACCTGTGTAGCTGCTAGCTGTAGGCACTGTCCGGATGAAGAAGAGGGCTGCCACTGTCAGTGGCACGTCAATCATTTTATGCAGCAGTTCCTTGCCTCCAAATCTGCAGAGATGGGTTTCTTTTTCACCGCCAGTAGCCTCAACTTATTAGCAGGAGGAGGAGGAGGTGATGGCGACCAAACTAACAGAACACCACGTGGGCTAGCCCTTCACCATCCTGACCCAAATTTGCCATCTCTGCTTCAAAACATTGATCTATCTCAAACACGTTCCCTGACTGTGTCTGGTGCTGTCATCGGCATCCCTATTGACAGGTTCGTCAACTTGGTGGTGCTGGATCTGGAAGGCTGGGGGAATTTTAAGGATGATGACCTATTGAAGCTATGCAGAAGCAAGATGTACTTCCTGACGTACCTGAGCGTCAGGAACACTCGGATAAGCAAGATCCCACCGGAGATCAAGGAACTGTGGAGTTTGCGGACGTTGGATGCGAGCTGCACGCAGATAAGCGATGAGCTCCCACTCCAAGTGTTCAAGCTAACAAGATTGAAGCACCTAGATCTCAGGGGCACACGGGTCAGGATAAGCAAGCTACCGGCCAAGCAGCAGATTGTGGGCACACGGATCCACTTGTTTACCCTTCTCGTTGGTGGTGGTGGTGGTGGTGGTGGTGGTGAAAGTGAAACAGCAGCGAGAGTGACGCCCGATGTACGGCATCTTCGGGACCTCGAGATGCTGGCCACTGTTGACATGACCGAGAACCCCGTGAGCTTCCTTCGGGCTCTCGGTGACCTAAAATGGTTGAAGGTGCTCGCGATTACGTGGTCCTTCCGCCACTCCACCGACAAGGAGTGCTGCGCGGCGCTTCTGTCGTCCATCGGCAAGTGGTCCTACCTCGAGTCCCTCACCATTCACTGCGGGCTCGGCTGCTCCATGGAGTTTCTGGGCACCCTCTCCTATCCGCCCGAGCACCTCACGAAGCTCAAGGTGACGGGGGGTGTGTTTGCAGGCGTTCTCCGGTGGCTCAGTGTGCCCTCGCAGCACCTGTCTTTCCTGCAGATCACCATCTGCAGCATCACGGCAGATGATCTCAAGGTGCTTGCCGACCTGGCCCAGCTGCGGACCCTGGTGCTAGGCCTGGATTTCGTCCCCACAGAAGCTGTAGTGATCGAGGACGGTGGTTTCCGTCTGCTTCGGAAGTTCTTCGTCAACTGCCCAGTGCCATGGCTCGCCTTCGAGATAGGAGCCTTGCCAAATCTCGCATATCTTCAGCTGGAGTTCGCTGCGACGCTGCCAACCCAGACCAGGGCTCCATCGGGCATCGGCAACATCCACAGGATCACGGACATTGCTCTGTGCTACGGGGAACACTACGCCAACAGCCCCAGCGTCAAGATCATAGTGGAGGCCGTAAGGAAACAGATCGTCGAGCATTGCAACCCGATCGACCTCTACATCAATGGCATTGAGCAAGACGATGTTCAGGCACCAGATGACTTGACAGAAGATGCAAATGTGACTCAGAGCCGAACCGGATCCGGATCACAATCGCCCAGGGAATGA

>B97_Zm00018ab119730

ATGCAATGCAGTGGGTACCAGCCACCAGAATACATTGAAAGAGGTGAAGTCTCAGAAAAATTCGACATATTCAGCTTAGGTGTCGTAATGATACATCTGGTTTCGGGACGTGGAGGCTTCGAAAGATATGCATGCATGCATAGTGAAGAATTTATCAATCAGGTAAAAACAAACTGGAGGAATAAATTTCAGCAATTACAGATGAATTCCTTATGTGAAGTGTACTGCCACGAGGTAGAGACATGCACTCGGATAGCACTGAATTGTGTGGAGAAAGACACACAGAAAAGGCCTGATATAGATATGGTCATCAATAATCTAAAGGACCGTAAGACCCATAGATTCAAGAGTACTTGGACAAGTAGTTTTCGGCAGGATGGAAGAATGTATGGAAGCTATATGCCATATTCAAACAGACATTTAAACAAATTGATGTCAGGCTCTATTTGCACTGAAACAAAATTTTATCTAAGAGAAAAATTACCGAATGTTGGAGAAGAATTCATTATAGGGAGAAAAGAAGAGAAAAAGAAAATAGTGGCATCTTTACTACAGAGCATGCCGCAACACATCACTATCCTTCCTATCTATGGTATTGGAGGAATTGGCAAGACAACCATTGCAAAGTTGATTTACTATGATACAAATTTCAATAGTTACTCTCGAGTATGGGTCTATGTTTCACCGAAATTTGACTTGAAGAAAGTTGGAAACACTGTAATTTCACAACTCTCTAGTGAGGAGAGCCAAATTAATGAAACACAAATGATAAATAGTTGCCTGATGAAATTACTTTCTGGTAAAAAGGTTCTGATTGTTTTAGATGACTTGTGGGAGGATAGTTTATTTCAACTGAATAATTTGAAGGATATGCTAAGTTATGGTGATAGCATCAATGTAATAGTTATAGTAACCACACGCAGTAAACATGTTGCAGAGAATGTTAGCACTAATGTTGAGCCACACAAGATAGAACCCTTGACAGATAGCATGTGCTGGGATATAATAAAACAAAGAAGCAACTTTGAAGCTAAAAATCACAAAGAACATTTGGCACATATAGGAATGGAGATCGCTGTAAAGTGTGGAGGTGTGGCTTTGGCAGCTCAATCCCTTGGGTTCATGTTGAAGCCCATGGAATTATGTGATGAATGGATTGAAGTCAGAGACAGTGACATCTGGAATAAATCTATTTCGAAGGATGCCAATTCACCAAACCATGTGCTTGCATCCTTGATGTTAAGTTGTACCAAAATGGATCGATGCTTGATATTATGCTTTATCTACTGTGCAATCTTTCCAAAAGGTCATCATATAGTCAAAGAAGAACTAATACAACAATGGATTTCTTTGGGTTTCATCCGGCCAACAAAATTACACTCCAATATGCAGATATGTGAGAAGTATATTTTGCAGCTCATAGGATTGTCTTTCATTCAAGATACAATGTCACCAAAGGTGAGTTATTTATGCACACTACTACCAGCATTGAGTATTTAA

>B97_Zm00018ab135370

ATGGACATCGTGGTGGGTGCGCTGTCGGGCATAGTGGACGCGTTGCCGGGGAAGCTCGGCGAGCTGCTGGAGCAGGAGTATGCGCTGCTCTCCGGCGTCCGCGGGGATGTCATCTTCCTCAAGGATGAGCTATCAAGCATGCGCGCCGCCATCCACTACTGCGAGTCCCTCGACCACCACGACTCCCAGACCACTGGATGGATCAGCCTCGTCCGCGAGGTCGCCTACGACATCGAGGACTGGGTTGACCTCTTCAGCATCCGCGTCCACGGCGGTGCCCAATCCACTTCCGGGTTTCGTGCCTGGCTTAGCCGCAGCGTGGACAAGTTAACGGCGCTCCCCGCTCGCCACACCATCGCGAGCGAGCTCCAGGGACTCAAGGAGCGCGTTCTTGAGATTAGCCGGCAGCGGAACCGCTACAGGCTGGGCCAAATGGTTGGCACCACCTCGCAACATCCCCATGATCCCAGACTTTCTGCGCTCTTCGTCGACCCCGGCCGCCTCGTCGGCTTCGATGGGAAGGTGGAGGACGTGTCCAAGACTGTCATGGACGCCGGAGGTAGTAATGGGCTGAGAATCGTCTCCATTGTCGGGATGGCCGGCTCAGGGAAGACGACGCTCGCAAATGCTGTGTACCGGCGTCTCCAAGCAGACAACACCTTCCAGTGCTCTGCTTTCGTCTCCATCGGACCGAAGCCGGACATGGTGAAGACAGTCAAGGATATGCTCTCAAGACTCGGCGACGGCCACCGAGGAGGCGAGGACATTAGCCAACTCATCCCGAGGGTCAGAGGAATACTGGAGAAGAAAAGGTACCTCGCTTGGATCGATGATATATGGAGTAGTGAACAATGGGGAGTAATAAGGTGTTGTTTTCCAGACAACAGTCTTGGTAGTAGGATAATCACCACATCAAGGAATGATGCGTTGCCCACCAATCATCATTACGGTTCAAGCAAATTTGTCTACAAGATCGGCCTCCTTACTGACAATGAAGCCAGGGAGTTGTTTCTGAAGAAAGCTTTCAGCAGCCGGAATGACTGTCCACAACATCTGGTGGATGCTTTTACCAAGGTTCTGAGAAGGTGTGCTGGCTTGCCACTTGCTGTGCTTAGCGTAGCTGCCAAGTTAGCACACAAGCAATCAAGAGAAGAATGGGAGAAGCATGGATTGAACTTGCTATACAGCTCACATTCAGATGGGTCAGATGGGCTGAAGCAAATACTCCATCTTAGCTACAGCGATCTACAGCCACAACTCAGGTCATGTTTGCTGTACCTGAGCATATTTCCTGAGAACTCAGAGGTTGAGACAGATCGCCTAGTGAGGCGATGGATTGCCGAAGGACTCATCGCTGCAAGCAATGAGGATACGGCAATCAGTTCCCTCAATGAGCTAATTGGGAGAAACTTGGTACAACCGTTGGATCTGAACCATGATGGTATCCCAAGGTGTTGCAGAGTCCACCCAGTGATATATGATTTCATTGTTTGCATGTCGATGCAAGACAACTTTACCACTGTAACGGATGCTCAACATGTCCCAATCAACAACAAAACTGTCCGTCGGCTGTCCCTGAATTTGAAGAGCAATAGCAAGCAAGATCAACCTGCAGCACGAAATGAGACTACTGATTTGTCTCATGCTCGCTCAGTCACTGTCTTTGGTCACGCTAGTGCCACCCCTAATCTGACTGATCTGAAAGTGGTGCGTGTGCTGGATCTCGAAGGCTGCAAAGGTCCTGTGTGCTTGGATGGCCTATGCAAGCTGGTGCTGCTGAGGTACCTGAGCCTCAGAGGCACTGATGTCAGTGAACTCCCAGCGGCAATTGGGGATCTAAGGTGCTTGGAGACACTTGATGTGAGGTCAACGAAGGTGGAAGAGCTTCCTCCCAGTATTGTCAGGCTGCAAAAGCTAATGCATTTGCTTGCTGGGAGTGCCAAGCTGCCTGACGGGATGGATAAGATGAAAGCCCTGCGGTCATTGTCATGCGCTGCTACCACGAAGAGCTCTGCCAACGTCGTGGAAGAGCTCAGCAAGCACCATAACTTGAGAGAACTGGAACTGCATTACTATGCTACTGAAACGCCTGGGAATGAGAAGCAAATCAAGTTCCCCGCTGATGGGCTCCAGACTGTGAAACAACTGTGCATCCGGTGCACATCACCATCGGTGACATTTGAGCCCCGTATACTGCCAAAGGTCCAAGCACTTGTGCTGAGATTCGAGAAAGGCCGTGCTGATGACACGAATGGCGTGTCTGGCTTAGAGCATCTGTCGAGCCTCAAGCATTTGGTCCTTGAGTTTGAGCAGCATGATGCGGGTGCCATGGCTACAGTTACTGCAGTGAGGATGGCTGCTGAGGGGCTCCATCTAGATCATCAATATATAACTCTAAAGGTGGATGGGAAAAAGTACTGA

>B97_Zm00018ab139330

ATGGAGTTGGACCGGCTGCTGCTCGACCAGCTGGCTGGCGAGGCCCTGCGGGAGCTGCTGCACGCCGTCCAGGGCACCCTGTTCTGCCGCTCCACTGCCGAGCGCCTGCGCCGGAGCGTCGAGCCGCTGTTGCCGCTCGTGCAGGGCCTCGGCCCGCACGCCCAGCGCTCCGCGGGGGACCTCGGCGAGCTCGCGGCGCGGGTCAGGGAGGCGCTCGACCTGGCCCGCCGCGCCGCCACGTCCCCGCGCTGGAACGTCTACCGCTCCGCGCAGCTGTCGCGCCGGATGGAGGCGGCCGACCGCGGCATCGCGCGCTGGCTGGAGCGCCACGCCCCCGCGCACGTCATCGGCAACGTGCGCGGTCTCCGCGACGAGTCCCACGCGCGCATCGCCCGCCTCGAGCGCCGCGTTGACGAGATCGCCGCCAGCGCCGCGCAGCCGCCGCCCCCAGCCCTCTCCGTCCCCGTCGCGCCGCACAAGGGCGTGACCATGCCGATGGAGGTGCCAACTCACAAGGGCATGGCTATGCCGATGCCGATGCCGGTTCCTGTGCAGGCGGTGCCCGCCAAAGCCGGGGTGGTGGCCATGGACATGGACCTCACCGAGGGACACGAAAACGAGGGGATGGTTGGCGCCGGCGTTAAGGTGGCCAAGGAAAAGGTGAAGGAGATGGTTATGAGCGGCGGCGGCGGCGGCTGGGAGGTGGTCGGTATCTCCGGCATGGGCGGCAGCGGCAAGACCACGCTCGCCATGGAGATCTTCAGGGATCATAAGGTCCGAGCCTACTTCAATGATAGGATCTTCTTTGAGACGATCTCACAATCCGCAAACTTGGAGGCCATCAAGATGAAGCTGTGGGAGCAGATCAGCGGCAACATGGTGCTCGGTGCATACAACCAGATCCCAGAATGGCAGCTCAAGTTAGGACCAAGAGACCGAGGGCCTGTCCTTGTGATCCTTGACGACGTTTGGTCTCTCCCACAGCTCGAGGAGCTCACCTTCAGGTTCCCTGGGTGCAAGACTCTAGTTGTATCGAGGTTCAAGTTCCCCACACTGGTAAAACAGACATACGAAATGCAGTTGCTAGACGAGGCGGCGGCCTTGTCCGTCTTCTGCCGTGCCGCTTTCGATCAGGAGTGTGTTCCGCGGACTGCTGACAAGAGATTGGTCAGGCAGGTCTCTGCAGAGTGCAGGGGCCTTCCACTGGCTCTGAAGGTTATTGGTGCGTCATTGCGCGACCAGCCTCCTAAGATCTGGCTCAGCGCCAAGAACCGGCTGTCTCGAGGAGAGGCTATTTCCGACTCCCATGAGACCAAGCTTCTAGAGAGGATGGCGGCAAGTGTCGAGTGCTTGTCGGAGAAGGTCAGAGACTGCTTCCTTGATCTGGGATGCTTCCCGGAGGACAAGAAGATCCCCCTTGATGTCTTGATCAACATCTGGATGGAGGTTCATGATCTTGATGAACCAGATGCTTTCGCCATCTTGGTTGAGCTTTCGAACAAGAACCTTCTTACCCTCGTTAACGATGCACAGAACAAGGCTGGAGATTTGTACAGCAGCTACCATGACTACTCGGTGACACAGCATGATGTGCTGAGAGATCTTGCTCTTCACATGAGTGGGCGTGACCCTCTGAACAAGCGTAGGCGGTTGGTGATGCCGAGAAGAGAAGAGACACTTCCAAAGGACTGGCAGAGGAATAAGGATACCCCGTTTGAAGCTCAGATAGTTTCCATTCATACAGGTGAAATGAAGGGATCTGACTGGTTCCAGATGAACTTCCCCAAGGCAGAAGTGCTCATCCTCAACTTCGCCTCAAGCCTGTACTACCTCCCGCCGTTCATCGCGTCGATGCAGAACCTGAAAGCCCTGGTGCTGATCAACTACGGCACCAGCAGCGCGGCCCTTGACAACCTATCCGCCTTCACCACGCTGAACGGCCTGAGGAGTCTCTGGCTGGAGAAGATCAGGCTCCCGCCGCTGCCGAAGACCACCATCCCGCTGAAGAACCTGCACAAGATCTCGCTCGTCCTCTGCGAGCTGAACAGCAGCCTGAGAGGGTCGACGATGGACCTGTCCATGACATTCCCGCGCCTCTCCAACCTCACGATCGACCACTGCATAGACCTAAAGGAGCTGCCAGCAAGCATCTGCGAGATCGGCTCCCTGGAGACCGTCTCCATCTCCAACTGCCACGACCTCACCGAGCTGCCATACGAGCTGGGCAAGCTGCACTGCCTCAGCATCCTCCGGGTGTACGCCTGCCCGGCGCTGTGGCGGCTCCCGGCGTCGGTGTGCAGCCTGAAGAGGCTCAAGTACCTCGACATATCCCAGTGCATCAACCTGACGGACCTCCCGGAGGAGCTCGGACACCTGACGAGCCTCGAGAAGATCGACATGCGGGAGTGCTCGCGCCTGAGGAGCCTCCCGAGGTCGTCGTCCTCCCTCAAGTCCCTCGGCCACGTCGTGTGCGACGAGGAGACGGCGCTGCTGTGGCGGGAGGCCGAGCAGGTCATCCCTGACCTCCGGGTGCAGGTGGCCGAAGAGTGCTACAACCTGGACTGGCTAGCGGACTGA

>B97_Zm00018ab142940

ATGGCCGCCGCCGCCACAATCATGTTCGCTGGGAAGTTGGCCGGGAGCTCGGTGGCAAATGCAACCATCTCCTTTTGGATCAACAAAGCTTTCACCTGCCTGACTGACTACTGGAAGGCTGATGGCTTGGAAGATGTCAAGGGTAGGGTGCTGCAGTCAGTGAAGAAGGTCCAGGTTGTATTTGATATCGTTGACCCTGAATACATCAAGGAACAAAGCTCTGCCCTTGATCTTTGGCTGTGGCAGTTCAGGGATGCAGTTGAGGAGGCAGAGGATGTTATAGATGAGCTTCACTACGATGAGCTTAGAGAGAAGGCAAAGGATCATAAGGTCAGTGACTGGGGCTCCTCTTCTGCTAAACTGAAGCATAAGTTTGTCAAGTCCGTTAAACATGCTGGTGTCATGGGTAAGACTGTGAAAGAATTTACTCACCGTGGCACACTCAAGAGGCTGAGGAAAGTCTTGGAGGGATTAGAAAAGGCAGCCACCGAAATTGTGGCTATCCTCACAGTCACACAACATCTCAAAGACATTGGTTCAGGTAGTAAAAGGCAGGTGAATTTTGTGATCAAGGATCATGACACTGGTTCAACATTAACTGAACCTTACTTTGTTGGACGAGAAGAGGAGAAACAAAAGATTGTGCAATGGCTGATTGAGGCGCCAGTGGAAGCATCTGAAATTGTGAGGAGTACTCACCATGTTCCTATTCTCTCGTTAGTTGGCCATGGTGGAATGGGGAAGACTACATTAGCTCAATATGTATGTGAAGAGGCTGAAGTTGTCAATGATTTCAAGGTTATATGGGTCCATGTTTCTACTAGATTTAGTGCAACTTCCGTGACAAGTAAACTGCTGGAATCCGTCACAGGGGTAAAACCTTGTGCAGATCATTTAGAGACGCTTCAGCAGATGCTCAAACAAGAACTCAGGTCTGTAAAGTTTTTCCTTATTTTGGATGATGTCTGGGAAGATGAGAATAAAAAGGAATGGGAAAATGTATTCGCTCCACTAAGGAAAGCAAAGAGTGGGAGCAAAATTTTAGTAACAACCAGAATGCAGTCAGTAGCAGATATGGCTGCAAATGCCATGGGGGTTGAAAGAGAATACCTGGAATTAGAAGGGCTGCAAGAAGATGAAAATCTTAAATTATTCAATCATCATGTATATTCTGGTAGGAATCCACAAGATTTTGAAAATTTAAAACCCATAGGTGAACATCTTGTAAAACAACTTGGAGGATGTCCCTTGGTAACAAAGGTTGTCAGCGGTTATTTGCAGTGCAATATGGATCCTGACAGCTGGACCGATTTCTTACAAGAAGGCCTTGTACATTTTAATGGAAGTGAAGATTATGTAATGGAAACTTTGAGATTAAGCTATTATTGCCTACCGGCACAGGTCCAGATTTGCTTTCGATATTGCAGCATATTTCCGCAGAACTATGAATTTAAAAAGAAAGATTTAGTGTTGATGTGGATGGGTTCAGGATTGATATCACAACATGGAAACAAACCAAGAAGGATTGAGAATATTGGGTATCAGATCTTGGCTGAGTTAACTAGGAAGTCATTCTTTGAAATGAAATTCAAGGTACTTCAGTATAGTCAGAGAAGAGAAGAATATTACGTCATGCATGACCTGATGCATGAACTGGCACAATATGTTTCTGCTGGCGAATGCTCAACATTAATTGATCCCGTCATGTTGGAAAATGAGAGTGAAAATATTCGACACTTGCGTATTGCTTGTGTTGACAAGTTTTCTGCTGAAGAGGTCAAGAAAATCACACGTTTTAAGAATCTGCGCACTGCTATTATTGATGGTCCAGGTTTGATTGACAACGATATGGCAGTCATGGTTGAGAATGTTATACAAAAATCAAAATCCTTGCGTCTACTGCGATCAAATCTGGAGAACACATTCCATCTTCCTAAACTTGCTGATTTAAAGCATCTTCGTTATGTCTATCTGCATAGGATATCACTTGAGGGAATGCGTGGGCTTGTTAAACTTCATCACTTACAGCTAGTTGATTGTTTGAATGATTGTGGGGAGGAACTAAGGCAAGTGATGTGTTTGGGGAACATTGATCATCTGCGATATGTAAATTATGGGTCGCGTAGAATTGGTGAGTTTCCAATTGGTAGACTCACTTCACTTCAGGAGCTGCACAACTATCGGGTACAGGGAGGTGAAGGTAACAAAATAAGTGATATCAAGAACCTGGGTGCTCTTCGCGAACTAGATGTTTTCAGCATTGAGAATGTTGAAAGTCTTGAAGAAGCTGATAATGCCAAGTTAAAAGAAAAACCATATCTCAACTCTCTATCTCTCATGTGGTCAGCACGTGCTGATGCGAAAAATGGGAAAGATGATTTGATTCTTGATCATCTTGAGCCACATGCCCACATTCGAAACCTGAATATTTCTGGTTATTGTGGTGCAAGGCCTCCTATTTGGATTGAAAATCTCCATGTGAAGAACCTGGTGTCACTTGAGTTAGCAAGATGCATGTATTGGGAACAGCTGCCTTCACTCGGAGAATTAGAATGTCTTAAGAAACTTTTGTTGGAGTGCCTTCCTAGCCTACAACAGATTGGTCAACCATCTCAACTTTCCAACATTAGCTGTATTGGTTCATACCTTCCTCCACATCTTGACACATTGATTATAAGACATTGCAAAGAACTGAAGCAGATCCCTATCCTACCACCCTGCTTGGTTCATTTGGAAATATGTAGGGTTGGGTTGACCGAATTCCCAAGTATAGGCAATATACATGGTGAGAGTATCGAAAGCAGGCCATCTAAAATGCAGTTTGTCAGTGTTGAAGAATGTGAAAGTCTGACCTTACCTAAAGGAAGCCCTCTGTTGCAAATACACTACATCAGAACAATCCATGTCCTACACATTAGTGACTGTAAAGAACTGGAATCTGCTCCCCTGTTCGATGAAATGAGAAACCTTAGAGAGCTCAGCATCATAAATTGCCCTAAGCTGAGGGCATCAAGCGAAACTGAAGGCAAGAACCTGTCACCATCACTAAAGAATCTTATAATCAAGCAGTGTGGTGATCTAGTGCATTTTCTTATTAAGTCACTACATGGTCTCGTTAACCTTTCAGAGCTGGTACTAGAAAATTGCCCCGGCCTTCTATCCCTTCCGTCAGCTGATGTGTTCAAGAGTCTCAAGTCGCTGAAGTTCTTGGAGGTAATTGGGTGTGAGAATCTTTCGTCATTTGGTGGACTCAGTTCCCTTTGTTCCCTTGTTACACTGAAGATTAGCTCCTGCAGTAAGCTTGCAGCTCCACCTGTACTGGGTGGTGCTGCGTCTGGTCCTGCTAATTATGATGATGATGTTATAGAAGAAGAGAATATGGTGTTGCCTGTTAATTCCTTGCAGATCGACTATCTTGAAGTTGATCTCCCATGTGTATTGAACATTGAACCTCTCAGTAGACTTTGCCACACCAAAGGATTGGTAATTGGAGGCGGGACACAGATGGTGAGCTTGCCAGAGCAATGGCTTCTACAGAACCACAAAGAACTGCAGTCACTAAAGGTGTTATGTGCCAGTTCATTGGAGTCTCTGCCACCAAGTATGCGAGCCCTGAGAGCGCTCAACTTCTTCTTGTTGTCAGGAGCTGGGAAACTTACATCACTTCCAGACATGCCCTCCTCCCTACAATGGCTCCATGTCATAGGCTGCTGTCCAGAGCTGGTGACCCAGATTAGAGTGAAGGACAACCCTGAATGGAGAAAGATCTCCACAGTTCCCAAGGTGCACATAGCTGCTGCCAAGACTGTCCACTCTTCTCCTCATACTTTTCATGGTGGTTATTGCTTCATGTATGGAAAAGAGTGCAGTGAAGAGACAATATATGAAGTAACCAATAACCATTGA

>B97_Zm00018ab170390

ATGACAGGTGTGGAGCCAGCAATTATCGGTGCAATCGCTAACTTGGCGGCCCCGGTCCTGCCTATAGCCATTAAAGGGATACAAGGTGCACTGAAGAAACGGCAAGTCCGTGACAGTGATGTTGAAACCCTGAAATCCCAGCTTAGCTACATCCAGGGCATCATCCGTGATACTCGGAAGACTATCAGGAGTTCCCAAGACCCGTCCGACAGGCTTCAATCCTGGGCTGGATACCTCAGATGCTTGGCGTACGACATCGAAGACCTAATAGAAGGCCGCCGTGCCGGAACCATGACAGGTGCGAAGCTTAATGGCAAGATTACTATCATCCAGGATTTAATCAGATGTGTACAGTCCTATCCGGAGTTTATGGCGGTTCCGACGAATGAGGCTCCTAGTCAAGGCGCTGCTTCTTCCTCCACTACTTCAAGCACCCAGGGCTTTCCGCTGGCTGATCTTGTGGGCAAGAAGGAGGACCTCGATGAGCTTCTGGACCTCCTCGTCCGGAGACCCGACAATGAGCTGGACAAGGTCCTCAAGGTGATGGTGATCTCCGTCGTCGGCTTCGGTGGCATAGGGGAGACCAAGCTTTGCCACACAGTGTACACGGACGTACAGGAGAGCAGAAGGTTCTCCCTGCATGCGTATGTCAGCGCTGCTGGGAAGGACTGCAGCATCGTTCTGGAAGAGATAATCGAGCAATTTAGACTGCAAGAGGATCCACAAGATAGCAGTGGTGGATTTTTTCACAGATTCGCCGGAGCGTTTCCTGGGGCTCGTCGTACTGACCAAGTTCATGGGTTACCCGAGTATCTCCAAAGGAAAAGGTATTTTGTGGTGGTGGATGGCGTGGAGTCTGAAGAACTGGTGAGTGGCATAGCATCTGCCTTCCCGGATAATAGTATGGGCAGTAGAATTATCATGGGTATGAGGACCGCAGTGGGCAGGGATGCAGAGAGATGTGTGGGTCATCATCACAAGATGTGGCCACTTGAAGACAAGCAGTCGGTGGTGTGCTTCCTAAATGAAGCGGAGCGGCGGCGACGACGACATGAACAAGACGACCACTCGTCGTTCATTAGGTTTCAGGAACAAGACCACTCATCATCCTGTTTGCACAAGGTATGTGATGGCGTACCACTTGCGCTGGTTAGCGTATGTGAAGTCCGCAGAGGGTCCATCATCACTGCTGCCGTTGAAGAACAAGACCGCTGGCCACACAGAATGCCCAAGGTGCTCGACCACAGCTACGATGGTCTGCATATCCGGGGTGCTGGGAGCTGTCCGAACCAATACATCCCCTATCTCCAAGCCTGCCTGCTGTACTTTGCCATGTTCCCCCGCGGCAATCATGTCAAGAGGGGATCCCTGATCAGGCGATGGCAGGCGGAAGGCCTAGAGTTCGGAGGCAGCAATCAAGCTGCCGAAAACCTCAAGGCCCTCGTAGACCGGAACTTCGTTTGGCCCCTCCATGCGAGCCTGAATGAGCACGCCAAGACATTCCAGCCTCCTGGAGTGGTGCTCAACTACATCTCCCGCAGGTCTCAAGAGGAGGAATTCATCCTCAGGTCTTGTCCAAGTGGGGAACTTAATCCCAATTACAGCCGCCGGCTTTGTCTACATCCTGCCGAGGAAGAAGAAGGTGAACCCCAACCCGTGGTCATTACCAACGGTTCTGTACCACCACGCCTGCGAACTCTGGCTGTGTCCTGGGGGCAGCAACAGGCCAGCAGGATTGCTGAATGCGAGCAGCTCCGAGTGCTGGATCTGGCGTCATACAATGGTCTACAGCCAGACCAGCTAGAGGAGATATGCAAGAAACTGAAGCTTCTCAAATATCTGAGCCTCCTGCCAGATATCATCACTCAAGTTCCAAGCTCAATGTCTAATTTGCAGTGCTTGGAGACACTCGAGGTGGGGGAGGTCAATGGCAGGGCGGCAGTTCTGGTGCCTATCCAAGTCTTGGAACTGCCACGCATAAAACACCTAATCGGAAAATTTGAGCTTATTGACAACTTCAACGGACTACCAATTAGGGCTTATCCAGATGCACTAGTACCAAAGGCAATCAAGGAAAGCAACCTGGAGACGGTGTCGGGGTTCTTCACCCGCAGAGGCCAAGGATTTCCGCCACTCATGCGTCACATTAGGCAGCTCAGGAAGGTGAAGATATGGTTCTACAGGGATGCAGAACCCAAATACCTAGCAAGCTATCTCCCGAAAGCGATTACAAAATTCCTCAGGAATGACAACGTTCATCGCTCCCTGTCACTTGACTTCCAGGATGGCCCAAGACAAACAGAAATACTGCAGGCTTGTGTGGCTGAAGCTAGCGGTAATCTTTACTCCCTGAAGCTGTCGTCCGGCACAAATCTGAGCAGGATCCAACTGTCGGTTATTGCCACCAACAAGCTGACTGGAATCACAAAGCTATGCCTTTCCCGCTGGAAAACGATAACGTTGGATGCAGAATTTCTGAATGAGCTGACCAAATTGGCCAGTCTGACGTATCTGAAGCTGGATGCAGAAACAATAAAAGGTACGTACGACCAAAACCCAACACCAGGAGGAAATAACCAAGAGAAGGTCGTCATAGAGACTGGGCACATTGCAAATCTGCGGCGGATGTGCCTTGTGGCCAGGCAGACGCTGCCCGACATACAAGTCAAGCCTACAGCTCTGCAACGACTCGTTTCACTTCATCTCATCAGTGAAACGGACGATTATTGTCCTTCCGCCAACGTCATCTGTAAAGCCAACCCCCAGGACGACAACGAGCCGGCGCCGTTCACGAGCCTCCAGGAAGTCTCGCTGAATGCTACGGTACCCGAAAATTTAAGGGATTCTTGGCGTAATGCTGCAAGGGACCATCCAAAGAGGCCACGAATTCTCTTCATCCAACACCCTCACCGTGCGGGATAA

>B97_Zm00018ab171670

ATGGCCGAAGCATTAGTTGGCTTGCTCGCATCCGCTTTCGTCGGCATCGCCAAGGCTAAGCTGGGCTCTGCCATCGCAGAGCAGGCCAGTTTGCTGTGGAACTTTGGCGAGGACCTAGAGGAGATGAAGGATCATCTGGAGACTATCTCAGCAGTGCTGGCGGATGCCGAGAGGCAGTCTGTCAAAGATGAGTTGGTGCGGCTATGGCTGAAGCGCCTCAAACATGCTGCCATAGACATCTCTGACATGCTCGAAGACTACCAAGACACGGGCGACCAAGCAAGCGCAAAGATGTCAGGAGTTCTCTCATGTTTCCCCATGGCGTCCAAGAAGATTGTCGGGGCTAATAGGATAAAGGGTTTGAGAGAAAATCTGAGGAAAATCATAAAAGAAATCAATAGCTTTAACTTCAAAAATGGCAGTGGCACTACTAATGAGCAGTCATACAATGAACGTGAAACAACGTCATATCTGTGTGAAGAACCAGTAATAGGGAGAGACCAAGAAAAACAGGAAATCATAAACCTGTTGTTATCTGCAAACAATAGCAACGATGAGATAGGTATTGTTCCTATTTATGGCCTTGGAGGTATGGGCAAGAGTACTTTGGCACAACTAGTTTACAATGATGCCCAGTTCAAGAAGTATGATCATCGTGTATGGGTTTATGTGTCCCAACATTTCAGTTTGACCAAAATAGGACTCTCTATAATTTCTCAACTACCAACACATGGATGTCAACAAAATATGGGTACGCAGCAGGTGATAAAACAACACCTTAATAAATTGCTTCATGGACAGACCGTTCTGGTTGTTTTAGATGACTTGTGGGAGGAAAAGATGACTGAGTTGGATAAACTTAGAACGATGCTTCATGTCAAGGGCAGTAAGGTAGATGTGATAGTAACCACACGAAAGGAAGAGATTGCAAGGAAAATTTCCACGAGTGGACGATCATACAAGCTACAAATTTTGGAAGATGATAAATGTTGGGAAATAATTAAGAGATACAGTAATTTTGAACTTAAATCTAACCAAGAAAAAATGAGGGAAATAGGATTGGATATTGCAAATAAATGTGGAGGTGTGGCATTAGCGGCTCAAGCTGTTGGATTTATGCTACGATCCATTGACGATATAAGTGGATGGGTAGAAATAAATAGAAGTGATATCTGGAATGGATCTTCTGAAGACAATGGTGTGCTTCCATCATTGAAACTAAGCTATGAAAGGATGCCGCCACAACTAAGGATATGCTTTTCCTATTGTGCCATATTCCCCAAAGGCCATAATATTATAGAAGATGATTTGGTTCAACAATGGGTTGCTCTAGATTTCATCGAGCAATCCAAGGGAAAGGAATATGTGAACAAACTTTTGGGGATGTCCTTCCTCCAAGTTTCAAAGTTGCCTTCGAATTCTCAAGAGCATGCTGTGGTACGATACACCATGCATGATCTGGTGCATGACTTGGCGACATTGACCGTGGCAGACGAGTTACGAGTTTTTGATGTTGCACCAAGGAGAAATACACATGCCAACAAATATTGTCGCTATTCAGCACTAAGAAAATACGACCGGGCAGTGAAGTTGGCAAATATGCCTCCAAAGATGAGGGCACTTCGGTTCTCTGATAGCGGTGAGCTACTGGATATCCAGAGTGGTGCATTTTCATTTACTAAGTGCTTGCGCATTTTGGATTTCAGTGAATGCTCCGGTATACTATTGCCAGCTTCAATTGGTGAACCAAAACACCTAAGGTGTTTTATTGCTCCAAGAATGCAAAATGATAGTCTGCCAGGTTGTATCACTGAGCTAACGAAATTGCAGTACCTGAACATAAATGGATCTTCTCAAGTTTGTTCACTGCCAGAATCAATTGGCAAGCTTGGATGTCTAAAACATCTGTGTTTGTCTGGTTGCTCTGGTATATCTGAACTGCCAAAATCATTTGGTGACTTAAAATCTATGGTTCATATTAACATGTCAGGTTGTTCTTGGATCACAGAACTGCCAGACTCGCTTGGAAAACTCGCGAATTTGAAGCATCTTGAGTTATCTGGATGCTCTAGCCTAAAAGCAATCCCTGAACCATTGTGTGGCCTCAGACAGCTCCAATATCTGAACATATCATCTTGTGAAGGTCTTGATCAGCTACCAGAAGGCATTGGCGGTCTCATGGATTTGCAGTATTTAAACATGTCATCCTGTAGCCAAATCAGAGAACTGCCAGATTCACTGATGAAGCTCCAAAATCTACTAAATCTAAATTTGTCAGGCTGTAGTAACATGCGACACCTGGGAGGTTTACATGGCCTCACTGCACTACAGCACGTGGATATGTCAAGGTTATGGAAAGTTGGCCTTCAATATTTATCAGATGTTTTGGCGAACCTCACCAATCTCAAGTGCTTGCGCTTATGTAGTTTAATCATAAGCGGCGCAGAAGGAAGCGTTAGTTATGTCCCTGACTGGATCGGTGGTATTGCAAATCTGGAGCACTTGGACCTATCTTGGAATAATGGTTTAGCTTGTTTACCGGAAGGTATTGGTAACCTGAAAAGATTGCATACACTAGATCTTAGTGGTTGCTCCAACCTTAAATCTCTACCGGAGAGTATACGTGATCTTGGGCTCAAGTCTCTAGTGCTGGACCATTGCTCGGATGAACTGTTGGATCAGGCTAGTTCTCTAGTGCACTATTCACACATACTACCAGTCTTCAAGGTGCGCGCTGATGCTGCCAATCGGTGCAGACGTGCAGTAATCTCCATCTCCTCGAGGGCGATAATGTCAGTGAATTAA

>B97_Zm00018ab174790

ATGGCGGAGGCCATCGTGGGGTCGATGCTGTGGAAGCTGCAGCAGGTGGCGGTGAGTGAGGCGCGGACGCTGGTGGCCGTGAACGAGGACATCCGGAGCCTCCGGGACAAGCTCATGTGGATGCAGGCCTTCCTGCACGACGTCCAGCCGTCGCGCCGCGTGCAGCCCAACGAGCTCATCAAGGTGTGGCTGCAGCAGACCCGCGACGCCGTCTTCGACGCCGAAGACGCCGTCGACCAGTACTTCGTCCAAATCGATCTCTCGAGATTCCCTAGCTGGAGCCGTGCCATCTTGGGTTTCTTTGCCAGCTTTACCACCCAAGTGGTTGTCCGTCGCGACCTCTCGAGCAGGATCAGGCTGATCAATGGAAGGCTGGAGGGAATCATTGCCAACAAGGACAGGTACAGGTTGGGAGAGTCTACTACGCTGGGAGCAATTTGGAGGCCATCTAGCTCCACGTCACCGCTCTCGGAAATGATGGACGAGGTGGTGCTGCCACTGGTGGGACGAGAGGAACTGGTGCGCAATCTTAAGAATTGGCTTTATAAAGGCAGCGGGAATCATAAAAATGTGATCACCGTGACGGGGGAAAGTGGAGTCGGCAAGACAAAGCTGGTGAGGAACTTGTACGACAGCAAGCAGACCCTGTCTCACTTTGACATCTACGAGTGGGTGAGCTTTGGGCCCAACCTCAGTGCCTCTGATGTCCTCAAGATCATCATCAGACGCATTACAGATGGAGAAGAATGTTCCAAGGACAACATTGAGAGGAAGCTGCGGGAGATACTGAAAGAAAAGAAGTATCTGTTGGTGATAGATGCTGAGCTCAGTAACTCGGAGTGGAATCGCATTTTCGCTATGCTCCCTGACCCCAAGGATGTGGATGTCGATGCTGCCAGCAGAATAGTGAGGATTTCTCAGATTCCGCCACATAAGCCACCCCCACACTATCAAGAAGCCAAACTTGAGGTTCCGAAGTTTTATGACCAAGAAGTAGTCATCAATCTGTTCAAGGAAACCTTCCAATCATGTGGCAGAAATGAACTTCCTGACGAAGCAATAAGAGAATACAGACAGAGAATCTTGGACAACACAAAAGGGTTGCCGCTGGCAATAGTTCTTCTGTCAGGCCTTCTGCGGACCAAGGAGTACCCTAGTGAATGGGAGACGGTGTTCGAGCACCTGGACAGGATGCAGTCAAAGCAGATCGACAAGATTCTATCCCTCTGCTTCGATGACCTTCACTATGACCTGAAATCGTGCCTCCTCTACTTTGCTGCATTGCCAGTGAATACCTTTATCAGGGCAAGCAACATCGTGTGCATGTGGATGGCGGAGGGCTTCCTGGTACCCAAGGCCACAACGGTGGAAAAAGTAGGAGAGCAGTACCTGATGGAGCTGATAGATAGGCGCCTCGTCAACTTGGCGCCAGTGGGCTACAATGTTCCTGGGTACGAGCGTGTAGCTGTTCAGAGCAAAGTACACGCTTTCCTGCAGCTCGAAGCACAGGAGCAATGCTTCGTGGAGATCCACAGTGGTGACGACATCCCGGCTTTGTCGGATGCACGACGCTTGTCACTCCAGAACCACAAAGACAAGTATGCAGCACTGTCACACCCGCTGCCGAAGCTGCGAGCCATCCTGTCAAACTTTGAGACGGAGCAAGAGGCTGCACAAGTTGCTGGCGAGAAGCCACCAGATGAAGCTGGAGAAGGGCAACATGGAAATGGATGCCGGCCTGGTTTCAACAAAAAGAAAATGCAAGCCAAGTCTTGTGTGCAGGACCTGCTACGGAACTCAAGGTTCCTCCGTGTCATCCATTTCAACGGCCTCGAGGTGGACAAAGAGCTTCCTGACGAAATCGGCAAAGTTGTGAACCTGCAGTACGTCCGAGTGACCTCCTGCTCCTTGGAGAAGATGCCGCCGTCGATTGGCAGGCTGTGCAACCTCCAGACTCTTGATGTCCGGGGCACCTCGGTCCGAAGCTTGCCAGATGGGTTCTGGAGGATTCAGACGCTCCGTCATGTGCTGGGCGACCGTCTCATCCTGCCTAAACGTGTTGGTGACTTGAGGAATCTGCAGACACTCAACAGCGTGAAGCCCGACTATGGTGACCCCCATGCCTGGGACGACAAGACCTTCAACCACATGATCCGCCTCTTGTACTTGCACATCCGGCGCGGTGAGAGTCTGGGCGTCCAGGAAATCTTCTCCAACCGCAAGAACAAGGCTGCCTTGGTAAAAGCAGTATCCATTCTCAAGTACCTTGTCGTCCTCATCATAAAAGCTCCTGTGATCCCCTCAGAAGTGTTCACTAGGTCCAACCTCCAACGTCTCAAGGCAATGGAACTGGTCGGGAAGCTTGACCTTCCTAAAAATGGCATCCCTGAGATGGATGTCCGCAATCGCCTCCCAAACCTCAAGAAGCTGAGGCTTATGAAGACAATGGTGTCACAGGAATTCATTAACCAACTTGGAGGCCTACAGTTCCTTTCCACCCTTGAACTAACCAGCAATTCTTTCAGGGAGCTTGTCTTCACGGAAGGATTTTGCAGCCTCAAAGAACTGACATTGCATGAGGAGACACTCGAGAGGTTGGAGATACATGAGTCGGCACTTCCCGAGCTTGGGGATCTGGATATTGTCGGCCATAGGGACAAGCTCCATGTTGTTATCCATGGTCATTCCGTGCTCGTGCAACACATTGAAGCAGAGGATGAAGATATTTTTAAGGTTACGGAAGTAATAACGACGCAACAAGTCCAAGTGGAAGACCACAAAGTCTGA

>B97_Zm00018ab203450

ATGGCGGAGACGGCCATCGCGGCGGTGCTCTCCAAGTTCGGAGGGCTCGCGGCGAGCGAGGCCAAGGTGCTGCTGGAGGTGGGAGACGACATGATGCTGCTGCGTGACCGCCTCGAGTGGCTGCAGGCCTTCCTCCGCGACGCCGACCACAAGCGCCGCACCGGCGACGACCGCCTCACCCGCGTCTGGGTGCGCCAGATGCGCGACGTCGCCTTCGAGGCGGAGGACGCCGTCGACGAGTTCTTCCGCAAGGTTGACATGGAAGGCATGGGTTACCAACGCTGGATCACATGGCTCAAGAAATGCTTGCTAGGCTCCATCAGAAAATTGTCTGGTCGCCTCGAAAAAATTAACAGCAGGCTCCATCAGATCTCGGAGAACCAAAAGGAGTACAAGGTCGAGCATACACCATCAGCGACAGTACTGACGTCTTCCACTACAGCCACTTCAGCATGGCGGGATGGTTACAAGAACGCCGTGGGTTTTGAGAAAGAAGTGGAGACACTAAAGGAAATGCTGCTTGGTAAAGGTTGCCCTCAGCTGACGTTTATCTCCATACTTGGAGAGAGCGGTGTGGGAAAGCAAACGCTGTCACGTATTCTCTTGCATGACATGGAGAAGAACAAGCAGTTCGACGTCCGAGTCTGGTACAACATGCCGTCGGGTTCCACCACGGAAAACCTCCTCAAGCAAATCTACAAAAAAGCAGGAGGAGGAGGACAGCGGCGGCATCAGCCGTGCGAGTGCGACGACGACGGTATCGAAGACGTGCTCCGTCGTCACCTCCTAGCCAACAAGAAGTACCTGCTGATTCTCGTTGGCATATCCTCCAAGACCATGCTCAACTGTGTCAGGGCAAGCCTACCGGACGACAACAATGGAAGCAGGGTAGTGCTCATATTGGACATCGAGAACGAAGAGGTAGCGTGGCATGCTAATGCCATGAACAAGACGACGGGCATCAGCCACGGATTAATCCACCACTTGAACCGTCTGGACCAAGACAAGAGTGTGGAGTTGTTCTGTATGAGGGCCTTGAGGACAAATCTGTCAGATGAAACCACAGTGAACAGCATGATGAGCAAGTACAGGGAAGTTGTGTACAATATAACTTCCGGCTACCCTCTGGCTATAGTGGTTTTGGCCGGACTCCTACGGTTCAAGGAGAAGCCAGGGCAATGGAACGCGGTGCTGCAGCAGCTCAGGACGACGTCGTCTGGACCAGCAGCAGCAGATCAAGAAGCGCATCAGCAAGACGACCAAGGCGGCCATACCGAGGAGAAGACGATGAGCGCCCCTACAAGTACAACACAAGCAGCCAACAACCAGCTGTCCACAAGAACGTCGATTGAGAGGGTCTTCTGGGCAAGCTTTGAAGACCTTCCCAACGCCCTCAAGTCATGCTTCCTCTACTTGGCTGCTTTCCCCAAGGGCACCTTCCTGTCTACCGGCAGTATAGTGCGGTTGTGGATGGCCGAGGGATTCATTAGGCCACAGAAGGGCAAGACCATCGAGGAGCTGGGCCACGACTATTTCAAGGAGCTCGCCTTGAGATGCCTCGTTCAGGTTTCAGGGATGAACGAGGTTGGCGGCATCACCAATGTCATTGTTCACGGAAGGCTCCATGGGTTCCTGCACTCGGAGGCTCGTGAGGCCGGCTTCATCGACGTCCATGACATGAACGACGTCTTTGTCCCACCATCGGTGCGCCGCCTCTCTTTCATGAGCTTCCAAGACGGATATACCACATTCACTAACAGGTTCTGTAAGTTGCGCTCCTTCATATGCTGGGCCAATGAGAAAGATTCAGACAATAGTATTGGCAGCAGAGGCCGCGTGAACAATGAGGAGGAACGATGGCATGATCTCAACTTCCTGCATGGTTCAGATTTGCTTCGTGTACTCTACATATCAGGACTAAGGATCAAGGAGTTGCCGAATGAAATTGGCAACAAGATCCACTTGCGGTACTTACGTGTAAACACCGAGCACCTCAAGGAGCTCCCGGCCAGCATTGCGAGGCTGCCCAACCTGCAGACACTGGATATAAGGGACACCGAAGTCGAGGAGATCCACCCGTCCTTCTGGGAGATAAAGACGCTGCGGCATGTGATTGCCAAGAAGCTCACGCTCCCGCCATCTATCAAGGAAGAAATGGGCGAGCTGCAAACGCTGCGTGGTGTAAAGCCCAGCGAAGAAGAATGGGATCAAGATAACTGCCCACTGCTCAAGATGTCCAAACTCCGGTCGCTGGAGCTGCACGGACTCATTGGCGCCAGACACGGCGCTGCGCTGATCACTGCCCTCGGGCAAATGCATCTCCTTGGCCACTTGAAGCTCAAAGGCGACAAGATCTCTTGCTGCGTCTTCACCGGAGAACGCCTTCGATATCTTCAGACTGTAGAGCTGGATGGAACTGTGCAATGGCCGCCTGTCGCTGAGTTTAATGATCTTCGCTTCGTCCGTCCAAACCTTGTCCAGCTCAGCCTGACAAATACAAACGATGCGCCGGAAGGCATCCAACAGGAACTGAGAAATGCAGGCTTTGTCCGCTCCTACCAGCAGCTCCAACCTGTTTATCGGCTTTCATACAGACAAGGAGGCGCGCTTGCGACGAAACCGGAGCAGCAGGGTGAAGCAGGGTCCAACAAAATGGAACAGCAGCACCAAGGCGAGGAAGCTGAAGAGTGA

>B97_Zm00018ab204930

ATGCTGATGGTGGAGAAGCAAGAGCAGGTCATCTCCATCGTCGGGTTCGGAGGGCTGGGAAAGACAGCTCTCGCCACCGTGGTGTTCCAAAGCCTCCGAGCGCAGTTCGACTGCTCAGCCTTTGTTGCGGTGTCCCAGACCCCCGACATTGAGAGACTGTTCAGTAGCCTGCTGTACCAGCTCGGCAGGAGAGACGACAGCGCAAGAGGGAGTGACGCGATCGACGAGCTCAGAGGGCTCCTTCACGACAAGAGGTACTTGGTCGTCGTCGACGACGTGTGGGACACCACCGTGTGGAAGATCATCAGATGCGCTCTGCCGCCTGGCGGTGGTCATCACGGTCGTCAAGGATGCAAGATCATCGCGACCACACGCATCTTCAAAGTCGCGCAAGAAGTCGGCCGCGTGCACGAGATGAAACCGCTTGGGCTGCACAGCTCGAGGGTGCTGCTGTATACAAGGGTATTCGGCAACAGAAACAAGGACAGATGCCCTGACGAGAAGCTAGCCGAGGTATCGGACAGGATACTGATGAAATGTGCCGGGGTGCCCTTGGCTATCATCACGGTAGCCAGTCTGTTGGCCAGCAAAGGAAGGAGCAAGCTGGACTGGTACGAGGTGTACAACTCCATTAGCACTGGGACGGAGCACAGCAGCGCCGACGTCGAGAATATGAGGAAGGTTCTGTCTCTCAGCTACTACGATCTGCCGTCTCATCTGAGGACGTGCCTGCTCTATCTAAGCGTGTTCCCAGAAGACTACGAGATCGACAAAAGTCGTCTGATATGGATGTGGATAGCGGAAGGTTTTATTATTGGAGAACATGCTGGGGGCTGCAGCACCGGCCTGTTCGAAGTGGGAGAGAGCTACTTCAACGAGCTCATAAACCGAAGCATGATTCAGCCAAGGTACGAGACGAACAACGACATGAGAGTGTCCTCCTGCCGTGTACATGACATGGTGCTCGATCTCATCTGCTCCCTGTCGAGCGAAGAGAACTTCGTCACCATATCGAATGATGTGATGAGCATGAGCCGCGCATCTGCTGCGTCGTCGTCGCGCAAGGTTCTTCGGAGGCTGTCCCTTCAAAGCTGCAACAAGGCAGGCGACGGCGCGCATGGAGTTGGTGGAGCGAGCATGCAGCAAGTGAGGTCCGTGGTTGCCTTCTCGTCTGCCGCGACTCTGATGCCGGCCCTTTCCAGGTTCAAAGTTGTGCGCGTGCTGTGCTTGGAAAACTGCGATCTTTCGCGCGGGTACAGCCTTGCCGGCATCGGGAGGCTAGTCCACCTGCGGTTCCTAGGGCTACGGTCCACAAAGATAGTCCTTCTCCCCGACGAAATAGGGGACCTGCGGTTCCTTCAGATGCTGGACATCTCCGGCAACGCGATCCCATCCCTGCCGCCCACTGTTGCGCAGCTGAAGCAGCTCAAGTGCCTGCACATCGGCGAGGTGAATGCAGCGATGTCAAACAGCATCGGGCGTCTCACGTCGCTGCAGGAGCTGTCAGGACTGCGCGTCAAAGACTCCCATGCTATGGGAGACCTAGGCCACCTGACGCAGCTGAGGGAGCTGGATATCGTCTACTCCAGCGAGCAGGAACACGCGCTCGTGATGTGCCTTCAGAAGCTGCAGAATCTCCGGAACCTCTCCGTCTCCTTCCACGCGCTCCGAGGCACCTTGGACGGCTGGGTCGGGCCTCGGAGTCTCCGTAGCTTCCATGTGCGTGGCTACTGGCTGCTGAGGCTGCCCGGCTGGATGAGCTGTTCACGTGTCGAGAACCTCTCCTTCCTCGAGATCGACGTCGAGGAGCTTCACCCGGAGGATCTGGAGACCCTTGGTAGCCTCCCCGCTCTCCGGAGCCTGCACATGTACCTGGAAGAGACGACGACGCGTCCTAGGGATGCCGCCGCCGTTGCCGTTTCTAGGGTGGAGTTCGTCATCGGCGACGGCTGGTTCCCTTGCCTGGAACACGTGAAACTGCGCGGGCACTACGAGCGCGTGGTGTTCCGGCCGTCGGGGAGCTATGCCGAGGCTCACGAGCCTTGA

>B97_Zm00018ab205010

ATGGCAGAAGCTGTGGTGTTTGCTCTTGCAAAGATAAGCGTATCCTTGGCAGGGTCTGCCATTTCCGGTCTGAGGGAACATGCTTCTATAATCAAGGAACTTCCAGGGAAGGTGCGGCGAATAGAAGCACAACTGTCAATAATAAACGGCGCCCTCCAGCAGCAAGATTCAGCCTACCTCAGCGACCACGCCTACATGAAATGGATCGCCTACATCAGGACCTTGGCCTACCAGGTTGAGGATATCATGGATACATACTCACACCATGCTCATCAGCTGGAGAACAGAGGGTTCATGTGGAAGCTGACCCAAGAATACCTCGGGCCTTTCAGAAGCATCTCTGCAGAGATCACCAAGATAGAGGAGAATCTCAAGCATGCCACAGAGCTGAAACAAGCATGGCTGGAAAACTACGGCCACCATGGCCAACAGATAATGGAAGCTGAGCTTTCCCAGGACTACATCCCTGCCGAGCCCAGCTGGGACCAAGATTTTGTCGGGATGGACGGCAACACAACACTGGTGACTGAATGGCTGCGCTCTGCTAGCGATTCAGAAAGCACGTTCATGACATTGCTGGGCGCGGGAGGTCTGGGAAACACCACCCTAGCCATGGACGTGTACAAGCGCGAGAAGGACAGGTTCCGTGTCCACAGTTTCATAGCTGTGGAGAGGGACTGCACCATGGATGCCTTGATGAGGAAGATACTGTTGGAGATTGGGAGCAGCATGAAGCAGCCGCCCTCGGAAAGCGTCGACAGTATCCCCGCCAACCAACTGAAGGAAGAAGCGAAGAGAAGGATCAGCAAGCTCAGAGACGGAAGGTGCTTGATTGTCTTGGACAACGTCCGCGACCCGAGAATTTACTTTGAGATGCGTGATGTGATGAGCAATCTGCCAGGAGTTCGCATCATCTTGACGACGAGGAAGACACAGGTCGCAGCCGCTCGTGATCCCACGTCGTCACGTTTCCTGCAACTCCAGCCGTTGGACCACATCGACGCGCTACGTCTGTTCTGCAGGAAGGCCTTTTTCAAGACGAACGACAGCATGTGCCCCCCGGATGTCGAGGTTTTCGCTACTTCTTTAGTGAAGATGTGCAAGGGCCTGCCCCTAGCCATCGTAGCTATGGGAGGCATGATGTCGTTGAAGCCACCAGTGGAGCAGATATGGAACCAGGCATGCGTTCGTCTCCAGAAAGAGCTGGAGAGGAACGCGGATCACGTGCAGGCCGTCCTGAACCTGAGCTACCATGACATGCCGGGACACCTCAGGAACTGCTTGCTGTACTGCAGCATGTTCCCCGAAGACTACCACATGTCACGGGAGAGCCTCGTGCGGCTGTGGATCGCGGAAGGCTCCGTGCTGGCAGCCAACAGCCCGACGCCGGAGACCATCGCGGAGGCATACTTCATGGAGCTGGTCCGCCGCAACATGCTGCAGGTAGTGGACAACGACGTGGTTGGCAGGGTGAGTACCTGCAAGATGCACGACATCGTGCGCAAGATGGTCCTCGTGGTTGCCAAAGAGGAGAGGTTCGCTTCTGCCACCGATTACAGCACAGTGTCGCACACCGGTAAGGACGTTCGTCACCTGGCATTGCATGGGTGGAAGGACACGAACACACCACCAGTCAAGTTCCCTCGTCTTCGACGTCTAGTGGCACTCGGAGCGAACTCATGCCTGACGAAACTGCTACCCGCGATTTTCTCTGGATCGAGCTTCCTCACTGTTCTGGTGCTGCAAGACTCCGGCATCTCCGAAGTGCCCGCGTCTATCGGGAGCTTGTTCAACCTGCGCTACATCAGCTTACGGTACACCCAGGTCAAATCCCTCCCGGAATCCGTCCAAAGGCTCGCGTACCTTGACACGCTGGACGTCAGGCAAACCAGAGTACAGAGGCTGCCACAGGGTGTCGGCAAGGCCAGGAAGCTACGCCACATCTTGGCAGACGCTTGTTGTCCCGATGGCAGCCAGCAGTCAGAATTTCGAAGCTTCACTGCGTTGGAGCCCCCAAAAGCGCTGACGAGTTTTGGAGAGCTGCAAACCCTCGAGACTGTGCAGGCTAACAAGGACATGGCGATGAAGTTAGCGAGGATGATGCAGCTGAGAAGCGTATCGATCGACAACATAAGCTCTGCCCTTTGCGCGGAGCTGTTTGCTTCCGTCTCAAAGCTGCAGTTCCTTACCAGCCTGCTTCTCTCTGCAACAGATGAGCACGAGCCGCTCAGTTTCCAGAACCTCGTGCCGAAGTCAAGCTACCTAAGCAGGCTGACTGTGAGAGGAAGCTGGTTGGGGAAGACACTGGATTACCCGGTTTTTAAGGAGCATGGGCGGAATCTCGCGTATCTGTCGCTTAGCTGGTGTCTCGTTTTGGGAGATCCACTCCAGTTCCTTGGGTCACACTGTCCACATCTTCAGTACCTGTGCCTCCATAGGGTGCAGAGCGCAAACTCCTTGGTTCTTCCGGAGAGATGCTTTCGGGAGCTCAAGAATCTCGTCTTGGAGCGCATGCCTGATGTCAGCCAGATGAAGGTTGGAGATGGCGCCCTTCAGTGCGTCCAAGCTATCCACATCACGGCGCTGCCCAACCTAGATAAGGTACCTCAGGGCATGGAATCCCTTACCACTCTCAAGAAGCTATCTCTGCTCGATTTGCATAACGACTTCATAGTTGACTGGGAGAAGAAGGAAATGAGCCGGAAGATGCCTCTTGGTCTGGAGTTGCGCATATAG

>B97_Zm00018ab207760

ATGATCAGGAAGATCACACGTAGGTTCAGCATTTCCACAGATCGAGCCGGCTTTCAGTTCAGACCTGAGGGGGACGATGGTAGCACCAGTGAGGGAATCGTGGATATCACGAGGTGGTTTGAGGAAATCCATACAGATCAAACTGCCGCGCAGCTCAGGCCAGAGGACGAGGATGGTGACATCAGCAAGCGAATTGGGGAGATCATACGCCGATTCGAGGAGATCACTGGAGATCGAGCAGCCCTGCACCTCGGACAGGAGGATGGCGAAAGAAACACTTGGAGTGGGAGGGACTCCACGTGGGAATCAAGAGTGACTAGCCATCTTCTGGATGAATCCTGTGTTTTTGGTAGGACCAAGGAAAAAGAGCATGTTGTAAAGTTGGTGAAATCATACAGTAAGTGCCCAGGAATTCATGTTTTGCCAATTGTTGGAATGGGGGGCATTGGCAAGACAACGGTGGCACAGATGGTTTACCGCGAGGTCCAAGAAAGTTATGACCTTCTGAGTTGGGTTCATGTCCCCGAAACATTTGATTTACGCAAGCTGGCGATAGCAATCACAGAGTCTCTGTCCAGACAACCATGTACATACAACAATTTTAGCGTCGTTCATGATGTTCTGCAACAAACAGTACTAAACAAGAGAGTGTTCCTTGTTCTGGATGATCTATGGAATGAACGGCAAATTTGCTGGCAAGATTTTCTTTGTTCACTCAAGTTTGCTGATACTATGACAATCCTGGTGACTACTAGGAGCAAAGAGGTAGCACAACTTCTGCAGACCATTCCTCACTTCGAGCTTGGCTTGTTACCTGAAGATCACTGCTGGCAGTTGTTCCAATGCTATGCTTTTGGCCATAGAAACATCCATGAAGAGTCCGCTTTGGTTCAGGTAGGTAGGAAAATTATGGAGAAATGTAGTTGCTTACCATTGGCAATTAAATCCATAGGTTGCCTGTTGCGGTCCAAGATGGATATGCAGACTTGGATGGAAATATCCGATAGTGAGTTTTGGGAATATTCAGATAATAATGAAGAGATCTTGTCGGCTCTTAGACTGAGCTTTCATCGGCTGCCAGGAAGGCTGAAGCCTTGTTTTCTGCTGTGTGCTTTATATCCCAAAGGCGAGCCTTTTACCAAGGACGACATGATTCACTTATGGACTGCTCATGGTTATGTACAGCCTTCAGGATGCAAAACACTAGAGAAGGTCGCTGGTGAGTACTTTGATGAGTTGAATGAAAGATCACTGATCGAAATGGATACATACTATTTGGTCAGTCGTGAGGGTCACAATTACTTGAAAAAGTCACGTGTAAGATCACCAGTTGAAATCTCTAGTGGGGAGATCTTCGACACCGATCTTAGCTTTTATGAGCTGCACATAAGATCATTGGTTGAAAATTTTCACAAAGGAACGACAGAGTCTTCCTTGCCATTTCAATTGTTTAGGCTGCATGACATGATTTGGGACCTTGCAAAATCTCTGTCCAGCTGCTTGTTTTCTGCTGTAGCTGTTGATGAAGGTAACCTTTACATGCAAAATGAAGTTCAGCACTTATTTCTCTGGTTAGGCAGGGGCAGATCGAAACAAAATACACAGAGGGGGCACTCTGAACTTATCCCCATTTCTAAGTCTCGTGATCTATTCATCTCTTGGATAAACAATTCTCTTGGGCTTGAATCTGAGCCATTTAGATGGAGGCATAGGTCCACACCCAGGCCCAGCCATCGACCCCAGGTGTCTGATTTTGAGCTCCTAGGGCTAAGCTCAACTGCGTTGCTAGGCATTGCCTTGGAGGCCATGATCCAAGCACGACCTGATCCTGTGCAAGATCCCATGAGTTCAGCAAGACTTCTAGCATATTTGCCCGAAAACAAACATGAATACATACTTTCTACACAAAGTCGACGTTCTCAGTTGTTCAAAATAGATTATTTACGCACCTTAATTTTAAAACAATGTACATTTTACAATATTGGCATATACACATATCTGAGAGCTCTTATACTATATTCATGTAAAGATAGTGGCTGCATAGCTGCAATCCAATATCTGAAGCTCTTGCGCTATCTCAATATAAGGAATTGTGATTCATTGACTGGCAAAAATCTGAATCACTTGACACAATCAATATGTCATCTTTACAGCCTTGAGAAACTGATCGTATCCACTTGTTGGAAAGAGTTCTCCATTCAGTCGTGCCATCTTTTCAGTTTAAGATATCTCCAACTCTCCGTTCAATTCAATGACTGGTCCCAGCATCCACTTTGCCACTTCCATAACTTGGACACACTGTGCTTGCAGAACTGCCATAGCATTGCGGAGCTCCCTACAGGCATAGGAAATCTGATGAACCTGAGATGCCTTAAGCTTATTGGAATTTCAGAGATCAAGAAGTTGAATCATGATTCTTTACTATGTCAGTGTAATAACAACAAGTGCCAGTTAATGAAGGCAATATTCCCTGCCCTAATGGAGTTAGAACTTGATAGTCTGTGCGAACTACAGGACTGGTGTAAATTCCAGGATTCAGATTGCCCAAAGATGCAGAGCATCACTGTAAGAAACTGCAACAAACTTAGACGAATCCCTTACTTTGGTTCTGTCAGAAGTCTAATGATAATAAACTCAGCTCTAATTGGTCTCCAGCTTTCAGCATCTAACGAACCTTCTCAGCTGCAGACTCTTGATATCAGTTACTGTGAGAACCTGGAATCCTTGTTGGGACTGGAAAATCTTTGTTCTCTCGGGAGCTTATATATTGCCCATTGTCCTAAACTATTTGTCTTGCGTCAAGAGAAGCTTTTGTTTAGGCCTCAAAATATATTAATTGATGACTGCCCTGGACTGATCGAATGGTGTGATGAGCAAGAGCTCTACTATCATGCCCCCAAAACGGCGAATATTTCAGATATAATAAGGGCAAAGCAGCGTGGCATGGCTTATTTTCAATCCTTCGAACAGATCTGTCTCGACATTTGCCCGGAGCAGGGATCAGAATTGGTTTTGTCACCAGATACTTGGTTGCCGTTTGAACTGCGGCTCTTAAAGTTCGGAATTGAAAGTTCTGGTGGTGTTCCACCCTTCCACCGAGGTTTTTCTACACTTGACAAGCTGGAGATCAGGGGATGCCCAAAGCTTGAAGCACTGATGGATTTGGAAGAAATGAAAGTCCTCCAAAGCTTGGTCATAGTGGACTGCCCATCACTTTATATACTGCCTGAAATGAAGTTTCCACCGCTGTTGGGATCGTTAACAGTAGAAGGGTGTCATAAGCTCCAATCTTTGCCCTTAAATACATCTGACCCTTCAATGTTCACCGAGCTTGAGGTATCTGATTGCCAAGGACTTATGTACATCGCATCTCTGAGCCGTTTGAGCAACCTCGAAAATTTGGTACTACTTCATTGTCCTCTTCTCGAGCTCCAGGAACCGTTGCCAGCTATTCCTGAATCTGTCGTTGTTTTCCTCTGTCCCAAATTGAAGAAATGGTGTGGAATCCAAAATATTGAATACCTGGAAAATCTGTCTGATATGTCGCAAGAGGTGAATATTTGA

>B97_Zm00018ab208330

ATGAAACTAATAATAATGAGCAAAGTTATACAGCAGATTGGCACAGTCTACCTTACAGATGAACTTGTGAAGAGTTGGATTGGGGAGGTCCGGAAGGTGGCTTACCGTGTTGAGGATGTAGTAGACAAATACTCATACCATCTTCTTCAACTGGAGGAAGAAGGGTTTCTGAAGAAATTTTTCGTCAAGGGTACCCATTATGCCATTGTTTTCAGTGAAATTGCTGATGAGGTAGCTGAGATAGAAGAGGAGATTCAGCAAGTTATTCAGATGAAGGATCAGTGGTTGCAGCCATCCCAGCTTGTCCCTCACCCTGAGCAACTCGCTGAAATCGAAAGACAGCGTTCCCAAGACAGTTTCCCAGAATTTGTCAAAGATGAAGATCTAGTAGGAATTGAAGAAAATAGAAAATTGCTGACTGGATGGATTTACTCAGAAGAGCAGGCTAGCATGGTGATAACAGTTTCTGGTATGGGTGGACTGGGAAAATCTACACTGGTTACAAACATTTATGAACGTGAAAAGGTCAACTTCCCGGTACATGCTTGGATTGTTGTGTCACAGGTCTACACAGTCGAGTCTTTGTTGAGAAAGCTACTATGGAAGATTGGGCATATGCAACCACCAGTGCCAAGAGAGATTGACAAAATGGATGTACATGACTTGAAGGAGGAAATAAAGAGAAAGCTCCAGAATAGAAAATGCTTGATTGTGTTGGATGATGTTTGGGAGCAAGAAGTATACTTCAAAATACATGATGCTTTCCAGACACTCCATGGAAGCCGCATCATCATTACAACACGAAAGGACCATGTTGGTGCTATTGCTTCCTTTGACCACCATCTTGAGCTCCAACCGTTGTGTGGGCCTGATGCATTTGAACTTTTCTGTAGAAGGGCTTTTCACAACAAGAAGGACCACAAATGCCCCGAGGAGCTTAAGGAAATTGCTGGTGAAATAGTGAAAAGGTGCCAAGGCCTGCCACTAGCAATTGTTACAGTCGGCAGCTTGCTGTCATCTAGACCACAAATAAACATTTGGAATCAAACATACAACCAGCTTCGGAGTGAGTTGTCAACCAATGATCATGTCCGAGCAATCTTAAATCTAAGCTACCATGATCTATCTGGAGATCTCAGAAACTGCTTCTTGTATTGCAGCTTGTTTCCTGAAGACTACCCCATGTCACGCGAAGCCCTTGTGCGGCTCTGGGTCGCAGAAGGTTTTGTTCTGAGTAAAGAAAAGAATACACCAGAGGAGGTGGCTGAGGGAAATCTCATGGAACTGATCCACCGTAATATGCTTGAAGTTGTAGACTATGATGAGCTTGGCAGGGTTAGCACTTGCAAGATGCATGATATCATGAGGGACCTGGCACTTTGTGTTGCCAAAGAAGAGAAGTTTGGTTCTGCAAACGATTATGGTGAACTCATACAGGTGGACCAGAAGGTTCGTCGCTTGTCGTTATGTGGGTGGAATGTTAAGGCAGCAGCTAAGTTTAAATTTCCATGTCTCCGTACTCTTGTGGCTCAGGGAATAATTTCATTCTCTCCTGACATGGTATCCTCAATTATGTCTCAATCAAATTATTTGACAGTTCTTGAGCTGCAAGATTCTGAGATCACTGAGGTGCCAGCATTTATAGGAAATCTCTTTAACCTACGGTATATTGGGTTAAGGCGCACCAAAGTCAAGTCACTCCCAGAGTCTATTGAGAAGCTCCTCAACCTCCACACTCTGGATATCAAACAAACTCAAATAGAGAAACTACCACGAGGGATTGTTAAGGTCAAGAAGCTAAGGCACCTTTTAGCTGACAGGTTTGCTGATGAGAAGCAGACGGAGTTCAGATATTTCATCGGAGTGGAAGCACCTAAAGGTCTGTTGAACCTGGAAGAACTACAGACTCTTGAAACAGTGCAAGCGAGCAAAGACTTGCCTGAACAGCTGAAGAAACTGATGCAACTCAGAAGCTTATGGATCGACAATGTAAGCGGTGCAGATTGTGATAACCTTTTCGCGACTCTTTCGGCCATGCCACTTCTTTCCAGCCTCCTAATCTCCGCAAGAGATGTGAATGAGACACTTTGCCTCCAAGCCCTTGCTCCGGAATTTCCCAAGCTCCACAGGCTAATTGTAAGGGGCCGCTGGGCTGCCGAGACACTGGAATATCCAATATTTTGCAACCATGGGAAACATCTAAAATATTTAGCGCTTAGCTGGTGTCAGCTTGGTGAAGATCCATTGGGGGTCCTTGCTCCGCACGTGCCGAACCTCACCTATTTGAGCATGAACAGGGTCAGTAGTGCAAGCACTTTGGTTCTTTCTGCAGGGTGCTTTCCTCACCTGAAAACACTCGTCCTGAAGAAAATGCCTAACGTCGAGCAGCTGGAGATTGGACATGGTGCTCTTCCATGCATCCAAGGTCTGTACATCATGTCCCTAGCGCAGCTGGATAAGGTCCCTCAAGGCATCGAATCGCTTCTCTCCCTCAAGAAGCTTTGGCTTCTGTACCTGCACGCGGAGTTTAGAACGCAGTGGCTAACGAACGGGATGCACCAGAAGATGCAGCATGTTCCTGAGATTCGTGTCTAG

>B97_Zm00018ab208340

ATGGCAGAAGCAATACTCCTTGCTCTGAGAAAGATTGGTAGCGCTTTAGCAGATGAAACTGCCAAGAAAATGCTGGCCAAATTGTCTGAAAAGGTTAACAATCTGAGGGATCTGAATGACAAGATCGAGTCAATAAGAATGCAACTGACAGCCATGAACAATGTTATACGTAAGATTGGCACAGTATACCTCACTGATGAAGTCGTCAGGGGATGGATTGGGGAAGTGCGCAAGGTGGCCTATCATGTTGAGGATGTAATGGACATGTACTCCTATCACACACTTCAAATGGAGGAAGAATGGTTCCTGAAGAAGTACTTCATTAAAGCGTCACATTATGTCTTGGTTTTTAGTCAAATAGCAGAAGAAGTTATCAAGGTTGAGAAGGAGATCAAGAAAGTTGTAGAACTCAAAAATCTGTGGTTTGAGCCTTCGCACCTTGTTGCTGATCAGCTAATTGAGATGGAAAGACAGCGTTCACACGATAACTACCCACTACTTTTTAAAGATGAAGATCTTGTGGGGATTGAAGATAACAGGAGAAGGCTCACCGAATGGCTATATTCTGATGAGCTTGACAGCACAGTGATAACAGTATCAGGCATGGGTGGACTTGGAAAAACTACCCTTGTAACAAATGTTTACGAGCGTGAAAAAACCAAATTTTCTGCTACAGCATGGATGGTCGTGTCCCAGACCTACACTATAGAAGCTCTGCTTAGGAAGTTACTCATGAAGGTTGGTCGTGAAGAGCAGGTGTCACCTAACATTGACAAATTGGATGTCCATGATTTGAAAGAAACCATAAAGCAAAAGCTCGAAAATCGCAAATGTTTGATTGTATTGGACGATGTATGGGACCAGGAAGTGTACCTTCAAATGTCTGATGCATTCCAAAATCTTCAATCAAGTCGCATCATCATCACAACACGGAAGAATCATGTGGCTGCTCTTGCTCAGCCAACTCGTCGCCTTGTTGTCCATCCTCTGAGAAACACTCAGGCATTTGATCTCTTCTGCAGAAGAATTTTCTATAACAAGGAAGACCATGCGTGCCCCAGTGACCTTGTGGAGGTTGCTGCTAACATAGTAGACAGGTGCCAGGGATTGCCACTAGCAATTGTATCAATAGCTTGCTTATTGTCTTCGAGGACGCAAACATATTACATCTGGAAACAAGTTTACAATCAGCTTCGGAGTGAGCTATCAAAAAATGATCATGTCCGGGCAGTTTTGAATCTGAGCTACCATGATCTACCAGGAGACCTAAGAAATTGCTTCTTGTACTGCAGCCTCTTCCCTGAAGATTACCCTATACCACGTGAGAGCCTCGTCAGGCTTTGGGTTGCAGAAGGCTTTGCATTGAGCAAAGAAAACAACACAGCAGAGGAGGTGGCTGAGGGAAACCTCATGGAACTTATCCACCGCAATATGCTTGTAGTAGTGGAAAATGATGAGCAGGGGAGGGTGAGTACATGTACAATGCATGATATTGTGCGAGATCTTGCCCTTGTTGTTGCAAAAGAGGAGAGGTTTGGCACTGCAAACAACTATAGAGCAATGATACAGGTGGACAAGGATAAAGATGTTCGACGCCTATCATCTTATGGATGGAAAGACAGTACATCTCTAGATGTTAGACTTCCACGTCTTCGAACTCTAGTATCACTTGGAACAATTTCATCCTCCCCAAACATGCTATTGTCAATTTTGTCTGAATCCAGCTACCTTACTGTTCTCGAGCTACAAGATTCTGAAATTACCGAAGTGCCAGGGTCTATAGGGAATCTGTTTAACCTGCGTTACATTGGCTTGCGCCGTACAAAGGTCAGATCACTACCTGATTCTATCGAGAAGCTCCTGAACCTCCAGACTCTGGATATCAAGCAAACAAAAATAGAGAAACTTCCACGAGGAATCTCTAAGGTTAAGAAGCTACGGCACCTTCTAGCTGACAGATATGCTGATGAGAAGCAGTCACAGTTTCGGTACTTCGTTGGGATGCAAGCGCCTAAAGATCTGTCGAGCTTGGTAGAACTTCAGACTCTTGAGACTGTGGAAGCCAGCAAGGACTTGGCTGAACAGCTGAAGAAACTGATGCAGCTAAGGACTCTATGGATTGACAATATTACTAATGCTGATTGTGCAAATATTTTTGCCAGCCTATCAAATATGCCACTCCTTTCCAACTTGCTTCTTTCTGCAAAGGATGAAAATGAGCCACTTCGCATTGAGGCTCTCAAGCCTGGGTCCACAGGACTCCACAGGTTGATTATCAGAGGGCAATGGGCGCAGCGAACATTGCAGTGCCCAATATTTCAAGGCCATGGGAGACACCTCAAGTATTTAGCTCTAAGCTGGTGCCACCTTTCAGAAGATCCACTGGAGATGCTCGCTCCACATTTGCCAAACCTCACAAATCTGAGACTCAACAACATGCGTAGTGCAAGTATATTGGTTCTTCCTCCAGGATCATTTCCCAACCTGAAGTTGCTTGTCCTGATGCACATGCCTAATGTCAAGCAGCTGGTGATTGGAGAAGGTGCGCTCCAATGCATCGAAGGTCTGTACATTGTGTCACTGGTGGAGCTGGATAAGGTCCCTCAAGGCATTGAATCGCTTCGCTCCCTGAAGAAGCTCTCTCTTGTCAAGATGCACAGGGACTTCCTGACTGAGTGGAACAACAGCGGAATGCATCAGAAGATGCAACATGTCCAAGAGATTCGTGTTTAG

>B97_Zm00018ab210090

ATGGTGCATGTAGGGGAGATGCTGGCCTCGGCCGTCATTAAGGAAGTTGTTCGCAGGCTGCCTGCGCTGCTCCAGGCTCCCGTCAAAGGTCCGGCAAAGATGATCCGGAGCTTCAGGGAGGACCTCGACGAGATGAAAATGACGCTCGAGTCCATCAAGGCAACCATGGCGGAAGCAGAGCAGCGGTCTATCAACGACGAGACCGCGCGGCTGTGGCTCAAACGGCTCAAGAGGGCAGCCTATGATATCTCTGATATGTTTGATGAGTTCGAACACGGCAGCCCACCGGGCAAGAAGCTGCAGGGCCCTAGTTGGTTCGAGATAATGTTTAGCCGTGACATGGCTAAGAAAATGAAGAAAATGAATAAAAGGCTGAAAGAAATAGCGGAGCAATGGAAAAATTACGGCCGTGTATCATGTGAGCCCAAAAATGATGCTGCTGAGGAACAAGAAACAACTTCAAGTTCATCATCAGCCGTGGTTGTTGGTCGTCGAGCTGAGAAACAAGCAGTCATTAATATACTGCTTAGCTCCAATAACACATACCCTCAAGAAACTATTAGTCACTGTACTGTTATCCATGGACTTGGTGGTGTCGGTAAGAGCGAACTGGCAACATCGGTGTTCAATGACGAAAGAATCAAGGAGGCCTTTCCTCAACGGGCATGGGTTTGGTTGGGCCAAAATTTCCGCGAAAAGGATATTGGAAGAGCTATAATCTCTATAGTTGAATGTGGATCTTGTAATCTCGAGATTCTTGAATCTATATACCAACATCTCAGAAAGGTGCTCCTGGGTAGATGTCTCATTGTATTGGACAATCTCTGGGATTCAGTTCACTTAGCCAAACTGCAGGGTGAACTAGGAAGCAATGTTTCCATCTTAGTTACTTCACGCAGAGAAATTCAATTGAATATGCCCAGATCGACATTATTCCGCTTGGATCCATTGTCAGAAAGATTTAGCCTTGATTTAGTTAAGGAAGTTGCATCCTCATATTTTCCTGCAGGTGATATTCCAGAAACTGCAATGGAAGAAATTGTAAAGATGTGCGGAGGTGTACCTTTGGCCCTTAAATCTGTTGCGTCACAACTGAGACCAGAAAGAAGCGTTAAAGAGCTGTTGAGTTTAATAAGAGCTATCTCTCCGCCCAAATCAGATTATGGAACAACGGATATCCAGGACCGTGTTCTTGCATCCCTTAAGCTGACATATCACTTAATGTCCCCGAGTCTCAAGTTATGCTTTGCTTACTGTGCAATTTTCGCTAAAGGCGATGAAATTGATCGGGAAGGCTTATGCCACCAATGGATCGCTCTTGGACTAACTGAAAAGATGTATGCAGAGGACAGAGTCCGTGACTTATTGACCATGTCATTTCTTCGCGATCCAGAGCCACCTGCGATCACGAGGAGCAGTTCTGGTGGTTCATCCAAACTTAAGATGCATGATCTGGTACACGACCTTGCTATGTTAGTTGCTGATGATGAATTGCTAGTTATCAATCAGGAGTGTGTGGTATTTAAATCTGATTCACCTCGTTATGCCATGGTTTTTGCATGTAAGTTGGAAAACTTGCATAAGAATAAATTGCTAGCCGGGTTAAGGGCTCTTCATATAAAAGATTCTGATGGACTGAAATTTAAATGGTACAATTTCTCGTTTGTCAAATGCCTGCGGATCATGGATATTAGTGGGCTATGCACTGAGAAGCTTCCTTCCTCGATTGGAAATATGATGCAGCTGAGGTACCTAAATGCTTCAGGAATACAATGCGAAGTGCTCCCCGAAGCCATTGGCAGTTTATCAAAACTGCAATATCTCAATCTACATGGCAGTAGAATTTCAGCACTACCAGACTCAGTTACCAAATTGGGCCAGTTGATGCATCTGGACATTTCGGACTGTGTGCATCTGCAGACATTGCCCAACTCGTTCTGCAATCTCGAAAGCCTGTGTTTTTTGTCCCTAAAGAACTGCTGTCGACTAAGTTCATTACCTGACGATCTTGCCAGACTAGAGAATTTAGAGAAACTAAATTTGTCAGGATGCTCCTGTCTTGACACTTTGCCAAAATCCCTTGGTGAGCTGGACTCCTTAAAACTATTGGACCTATCAGGTTGTATGAAACTCACCATGCTCCCGAAATCTTTCATTAGCCTTACTAGTCTACAATACCTGAACATCTCAAGCTGTTCTGAGTTAGATATACCTGTCGATGCTCTTAACAAGCTCACAAAATTGAACTATATAGACATGTCATGTTGTCCAAAACTTGTAGGCCTCCCTCAAGAATTTTGCAGCCTTAAACATCTCCACACACTGAATCTCTCAGACTGTTCTAAATTAGCATATTTACCAGAGAAGCTGGGCAAAATGGAAAGCATCAAGTTCATTTTGCTTGATGGTTGTACGGAATCGGTGAGGAAACCTATTCTAAAACATAGGCTGGGTGCTGGTCTGCAGTCCTTACCAGCATTTGTCGTTGAAAGAAAGGTTGACAGCATACGGAGCAACATTTTCCAACTCGAACAGGAGAAGTTCTCTGAGCTGGAACTGTATCGCCTCGAGAATATACATACAGTTGATGAGGCTAAAGCACTGAAGATGCCTGATAGATCAGGACTACGTAGTTTGGGACTTATGTGGACATTAAATGTTGATCGATTCGTGGAAGATGAAGCACTGCTTCAGGCGCTTGAGCCACATGAGAATCTTAAAAAACTTACGGTGCAAGGCTACATGGGCGAAAGGTTTCCTAAATGGAAACTAGAACTTGGTTCCTCCCGCCAGGGCCATCTTCACGAAGTTGGATTAATGCATTTTCCAATGTGCAGCAGTTTGCCACAGCTTGGGCAGCTTGCAAATCTCAAGAAGCTTTACCTGTCCAGGATGCCAAAAATAAGGAGACTGGGAAGAGAGTTGTCTGATAACACTGGAGGGCTCAGGAATTTGCAAATCTTCACTTTGGAATATATGGAGAACCTGGAAGAATGGTGCACAACTATGACATCAGCTACGGGCCAGCAGAAGCAGGAAGAATTCATGTTTCCCGCGCTCCAAGAACTGACCATATACCATTGCCCTCTGTTAACGATGCGTCCCTGCCCTCCAAGAAGCATAGATTATTGGGAAGTAAGAGCAAGCAGTGGTGCAGCACAACTGCTTCTTCAGAAGGACGACATGATGCAATCCGTGGCAGACTATATGGGCTTGCAGTGTCCCTTTGCTTACACCAGCGAGCTGCATGTTAGTGGCTCCAGCAGCAGTTCCACACTACTTCCTACTGACGGATGGAAATTCAATGGTTCTCTCATCACTCTAAAGGATTTGACAAGTGATTGCTGCAGTTTGATAGACAGATTGCTAGCCAAAGGTAACAGCATGCAATGTTTAGTTAACCTAGAGATTTCAGGCATCAAAAATACAAATAGCTTACTGGAAGAAGTTGAATCAGTTGCATACTACACCCGATCGAGCCTCGCTAAATCATGGCCAGATTGGTTTAAACAGGAACAAGGCATAAACAGAGCATCACCACATTTTATAGTAACAGGTTATGCAAATTGTGGAGTCGATGGCTGGATTCATAAGGTGACCTCCTTTCTTGGCAACCTTATACGGATCAACATGGAGAACCTTCCTATGTGCGACTGCCTACCACCACTTGGTCAGTTGCCAATGCTGCAGGAACTGCGACTAAAAGGGATGCCTAAAATAAGAAGTATTGATCGAGACTTCTGTGGGAGTGGGAGTGGGAGTCAGCAATCGTCACACACATTGTTTTTCCCAAGGTTAACAAGGTTTGTTTTGAATGATATGCCAAACCTAGAAGATTGGGTCACTAAGGTGTCAGGTGCAAGTGATCCGTATGGCCAGGAGGAATTCATGTTTCCCAAGCTTGTCAAGTTGACAATTTGGAACTGCCCCAAGCTTAAGCTGAAGCCATGCCCCCCAAGAGCAATGGAGTGGGATATAAATAACAGCGATCAAGTAATAGCGTCAAACTATGATATAAACAGTGGTGGTTATCTTGTGACTATGCTGCAAGTATTGTTATGCAAAGTTCCACCCAGCAATTGGAAATTGCTTCATCAACTCCCTGGGATCCAAAGCTTGGCTATTGTAAGCTGCCACGGGATGGAAGCTTTACCAGATAGCATTCAATACCTTTCCTCACTCCACTCGCTAACTGTAAGCAAATGTCATGGCCTGAAACACCTACCTGATTGGTTGGGGGACCTCACCTCCCTTGAGAGGTTGATGGTTGTGAGTTGCCCCCTGGAGTTCTTACCAGGGAGCTTGAGGCGCCTCCCTTTCCTTCGGTCGCTCACTCTGAGCCGTTGTGACCGACTGGCAGCACTGCCAGGGTGGATGGGTGATCTCAAATCACTCGTGACGATCACAATTGAAGAATGCAAGAGCCTTAAATCTTTGCCTAAGCTTTATCATCTGGAACATCTACACATTCAGTGTAATGACGAACTAGAACGTTGGTGCAAATCAGAGGTGAATCAGCATAAGTTTTCTCAAACTCTAAGGAAGGGTTTCTTCCTGGAGAGTCCTATGGGGACCAACAGCTGCATCTTACCAGCTAGATCGTTGAGTATACTTTGGGGACAGGATGACAGATACTGGAGGTTGAATTCTATTCCTGAGTCCAGGTTCGCATTATCGATGGAGCTCATAGCAGTCTGGTGGCTCGAGATTGAGGGATGGGTCCCTTTCGAGTTCCTCTCCACCGACACTAGCTATGATATATTCCTTGTCTACAAGCTGGCAGATGAGCATGATGGCCTCAGATGGGGGGAGTCCTACGTAGCAGTGGATGGAGTACACACCACTGATGGCGTTGTTTCCTTTGTAGACGAAGATGCCGTGCGTGTAGATCGCGTGGCTTACCCGGTTACCCGTTCAGAAGGTTGGATGGAGCTCTGGCTTGGTGAGTTTTACAATAAGTATGTTGATAGAGAGGTAAAAGTGAGTGTCTGGGAGAAAACCGATACCTATGCCAAGATAGGGCTCATCATTGAGGGCATGGAGATCAGAAAGAAGAGCGGGAGTATTAGCTAG

>B97_Zm00018ab228530

ATGGATATCTCCAGTTACTTGGCTGTAGGTGGCTGGTTCATCCAGGTTATCTTCGACAAATACCTGTCCTACCAGCTCCGGCGATGGGCGGCCGACTGTGGCATCGAGCATGAGCTGGACAGGCTTCGTGTTGCTTTGCTTCGCACGCAGTCAGTTCTCCATGGTGCCGAGCTGGTACCCGCACTCTCCTACAGCTCCCTTCCATGGATGCGAGAGCTCCGAGATGTTATGTATGATGCAGAGGATCTCCTAGACAAGCTTGAGTACAACCGTCTCCACCATGAGGTAGAAGAATCAAGCGCAAACGAGAGCAGCAGTAGCCCAATCAGTGCCTTCATGCTCTCCCGGTTTCACAGCCAAGGTGTCGCAGCTTCTAGCCTTGAACCATGTTGGGATAGATCAGCAAAGGTAAAGAATAAGATGGTAAATCTGTTGGAGCGTATTGAGCAGGTTACAAGTGGTGTAAGTGAAGTACTCAGTCTACCCAGGAACATCGGAAGCAGCAATCGCAACGCCATGACAAGCTCAATACCCTACGGGAGAATCACCGGGCGGGACTTCGAAGCCCAACAGCTGGTAACTGCTCTGATAAGTTCTCAGGTCGAGAACCCAGTTTCTGTTGTCTCTATTGTTGGGGTAGGTGGCATAGGTAAGACTGCTCTAGCACAGCATGTGTACAGCAACGCTAGAATAACAGAGAGCTTTGATGTGAGGATGTGGATTTGTGTTACTTCTCTCTTGGACGAGTTGAGAATCACAAAAGAAATGCTGGAGTCAGCTTCCAGCAGTCGGTTTAGGCATGGTGGCATAGCAAATTTCAATAGACTTCAAGTCGCCTTGAAGGCAAGGCTTGCTTCAAAACGTTTCCTCCTTGTCTTAGATGACGTTTGGAACAATGACAACAAAACAATAGCAATAGAACAAGAGAACTGGCAGAAGCTGCTAGCCCCTCTGAAGGACGGAGCAAAGGGGAGCAAGATACTTTTGACAACTCGGTCAAGTATAGTAGCAGAGATGCTGCAATCATCCTCTATAATTAATCTGGAGACATTACAAGTTAATGACTGTTGGTCCCTGATAAAGACTTATGTGTTTGATGAGACAAAACATACCATCGACTCAAAACTGGAGAACATTGGAAGGAAAATTGCTGAGACACTCAGTGGCCTTCCTCTTGCCGCAAAGGTGGTAGCCGGACACCTGAAACGTAAACACAGTGTAGATGAGTGGAAACAAGTCTTGCAGAGCAATGCAGTATGGGAGGAAATCATGCCAATTCTACGAACTAGCTATGACAATCTACCACCACAGCTGAAACAATGCTTTGCATATTGCAGCATCTTCCCCAGAAACTGGGAATTTGAAGCTGAGCAGCTGATTCTTCTGTGGTTAGCACAAGGTTTTGTGCACCCAGATGGTTGCAGGAGATTGGAGGACATTGGGAAAGAATACATAAATGATCTATGTAATAAGTCATTCTTCACGATACAGAAGAAAGAATTTGTCAGCTATTATGTGATGCCACCTGTAATTTATGAGCTCGCAAAATCAGTGGCAGCTGAAGAATGCTTCAGAATAGGAGGTGATGAAAGGACAAGAATCCCATCGTCAGTGCGTCATCTATCAGTACATCTAGATAGTCTTTCAGCACTTGATGACACAATCCCATACAAGAATCTACGCACTCTCATTTTCCTCACTAGCAGAACAGTGGCTCCAATCAATGTCTCCATCCCTCCAGTGGCTCTCAATAACATAAGAAGCCTCCGTGTGTTGGATTTATCCCTGTGCATGATGGGCAGATTTCCTGATAGCATATCAAATTGTATGCATCTTCGCTACCTAAATATCTCATCTACTACCATTGCAACAGTACCAGAATTCTTGTGCAAACTCTACCACCTACAGGTTTTGAATTTATCAGGTTGTAGGCTTGGAAAATTGCCTTCCAGAATGAACAACTTGGTCAATCTACGGCATCTCACAGCAGCTTATCAGATTATTTCAGCCATAACAGACATTGGCAGGCTGAAATGCCTTCAGAGGTTGCCAACTTTCAAGGTTACCAGAGAGAAAACACAAAGTATAGTTCAGCTTGGGTACCTACTAGAACTCCAAGGATCCCTACAGATCAGAAACCTTGAGAATGTCGAAGCTCCAAATGAGGCAAAGGAAGCAATGCTTTGCAAGAAACGCCAGCTCTCTGTGCTGCAGCTAATGTGGGCATCTGATCGAGATGAGGTAAATGGAAATAGGGAAGAGGATGTGCTAGAAGCTCTCCAACCGCACGAAAATCTGAAGAGACTAGACATCGTGGGTTGGATGGGAGTCAAATCCCCTAATTGGCTTAAAAATGAATGGTTAAGCAATCTTGAGCTTATTTTCCTAAGTGGCTGCAATGCATGGGAGCAGCTTCCACCACTTGGTCAGCTTCCCTCCATCCGAACAATCTGGTTGCAGCATTTGAAAATATTGAGACAGATAGGCCCAGAGGCATATGGCAGTGGCAGCCAAATGAAGCCATTCCAGTCACTAGAAGAGCTGGTGCTTGATGACATGCCAGAACTCAATGAGTGGTTATGGAGTGACCAGACAATGAGGAATCTACAGAACGTCGTGATCAAGGACTGCAATAAGCTAAAAGTGCTGCCTCCAGTACCTCCTAACCTTACAGAGATAACAATTGCAAGGAAAGGGTATTGGGTGCCATACCACCATGATGTAAAGTTGGCACATGTGACCACTGGCAGGTCCAGTGTCTCATCTCTTTGCATATTCAATTGCCCCCTGTTACTTGCCAGATTATCTGCACAAACAAACAATGAAATAATTGAAAGATTTAGGTCACTTAGAAGCATTATCACTGATCAAATGGCAATACTAAGGTGTTCACTTTTAAAAGAAAGG

>B97_Zm00018ab231130

ATGGCGGAGACGGCGATCTCAGTGGTTCTGTCCAAGCTCGGGGAGCTAGCGACGAACGAGGCGACCATACTGCTTGAGGTGGGCGACAACATGGCGCAGCTTCGCGACCGCCTCGAGTGGCTGCAGGCCTTCGTCCGCGACGCCGACAGGAAGCGCCGGGCCGGCACCGACCAGCTCACCCGCGTGTGGGTGCGCCAGACGCGCGACGTCGCCTTCGAAGCCGAGGATGCACTCGATGACTTCTTTTACGAGATTGATCTAGAAAGCAAAGGTTATCAAGGCTGGAAACTGTGCCACGAATTCCTGGCAAGTTTGTACACCCAAATTGTTGTTCGACACGGATTGTCTACTCGGATTAAAAAAATCAATACAAGGCTTGAGAAAATCTCAGAGAACCAGAAGGAGTACAAGATCGAGCACACACCATTAGCGCCGATGGCATCTTCCACTATAGCCTTTTCAGCATGGTGGGATGATGCGGTGGGTTTTGAAGAGGATGTTGAAGCACTTAAGAAAATGCTGCTGCATCGTGAAGATGGCAATTGGCAAAACTTAATGTTCATCTCCATACTCGGGGAGAGTGGTGTGGGCAAGTACACCCTTGTAGACAAGATCTACTTTGATCTGGCAGAAGACGACACCACAAAAGAACGAGAAATTTTCGTGGGCGAAAGAAATAAGTTTGCTGTTAAAGTCTGGTACATGATGCCTCCAGGTTCCAGCACAGAACATCTCCTCCGAATTATCTACGAGAGAGTAGAAGAGCAAGTACAGGGTTGGCAAGCCGAAGGAGATGCGGCAACAGAAGATATCCCTGACAGGATCCGTGACCTCCTGGCAAATACAAAGTACATATTGGTTATCAGTGGCGTCTCCTCCAAAGCCATGCTCAACCGCCTGAGGGCGTGCCTGCCCGACGATGCCTGTAATGGCAGCCGGGTGGTCCTGGTGCTGGACATCGAGAGCGAAGTGGTGGCACAGCGGGCCAACTCCATGAACGCCGAAGACATCAGCGGGATCCATCTGCTGAGACGCCTGGACCAAGAAAGGAGCGGGCGGTTGTTCTGCGCCAGGGCCTTCGCCAAACAAGTAGATAACAGAGCTGTGCATGCGATAACAGGGGGCCACCCACTGGCCATCGTGCTTCTGGCCGGGCTCCTCCGATTCAAGGAGAGGCCGGGACAGTGGAAAGCTGTGCTGCAGCAGCTCATGGTGTCATCGTCGTCGTCAGGACCTGCAGCAGTGGAAGACGAAGAAGAAGCAGGGAGCAACGACCAGATGGTGACGATAACCGCTGGAGGAGGAGGTGGTGGAGGAGCTGCAGCGAGCAGGAGGACAGCCCTCGAGAGGGTCTTCTGGGAAAGCTTTGAGAACCTTCCCGACGGCCTCAAGTCGTGCCTCCTCTACTTCGCAGCTCAGCCCAAGGACACGGCGTGGACTCCCGACATGATCGTGCGGATGTGGATTGCCGAAGGCTTCATCAAGGCGCAGAAGGGCAAGACCGTGGAGGAGGTGGGGCACAGCTACCTCAAGGAGCTCACCCTGAGATGCCTTGTCCAGTCTTTCTACACCAACGACGCTATCGGCACCAAGGGGGTCCAGGTTCACAGGAGCCTGCACGGCTTCCTGCTGTCGGAGGCTCGCGAGGCTGGATTCATGGAGGTACATGAGACGCAGGACGACTTCATTCCATCGTCGGTGCGCCGCCTCTCGTCCTACATCTCCGACGGCGGATTCACCACTTTCGCCAACAAGTTCTCCAAGCTGCGTTCCTTCCTATGCTGGGCCAGTGACGACTACAGTGCTACTGCAGGCGGAAGCATTAATACGGATAAGAATCTTGATGATCTCAAGTTCCTGCTTAGGTCCAAGTTCCTCCGTGTCATCGAGATCAAGGGGCTGAGGATCAAGGAGCTGCCGGCTGAGATCTGCGACATGTTCCACCTGCGGTACCTTGGTGTTCACAGCCCGGGCCTCGAGAAGCTCCCACCCGCCATTGCCAAGCTGATCAACCTGCAGAAACTGGACATAAGGGGCACTGCAGTCAAGAAGATCGACCCGTCCTTCTGGAACATAAAGACGCTGCGCCATGTCCTTGCCCAGGACCTCGAGCTTCCAGCGTCGTCGTCCATCGACCAACAGCTGGACGGCCTGCAGACGCTGCAGGGAGTCAGGCCCTGCCGCCGAAGAAGAGGGGGACGGCCTGCAGACGCTGCAGGGTGTCAGGCCTGTCGCCGAAGAAGAGGGGGACGGCCTGCAGATGCAGGGTGTCAGGCCTACCGCCGAAGAAGAGGGGGACGGCCTGCAGACGCTGCAGGGTGTCAGGCCTGCCGCCAAAAAAGGGTGGAATCAAGAGAATTGCCCACTGCACAAGATGACCCAGCTCCGCACACTGTGCCTGCACGGAATCAAAATCGACAAGCACAGGGATGCACTGGGAACTGCACTCCTCAAGATGCATCTCCTGCGCCACTTGGAGCTGCATGGTCATGA

>B97_Zm00018ab231150

ATGCTGCTGCATCGTGAAGATGGCAATTGGCAAAACTTAATGTTCATCTCCATACTCGGGGAGAGTGGTGTGGGCAAGTACACCCTTGTAGACAAGATCTTCTTTGATCTGGCAGAAGACGACACCACAAAAAAACGAGAAATTTTCGTGGGCGAAAGAAATAAGTTTGCTGTTAAAGTCTGGTACATGATGCCTCCAGGTTCCAGCACAGAACATCTCCTCCGAATTATCTACGAGAGAGTAGAAGAGCAAGTACTTCGTTGGCAAGCCGAAGGAGATGCGGCAACAGAAGATATCCCTGACAGGATCCGTCACCTCCTGGCAAATACAAAGTACATATTGGTTATCACTGGCGTCTCCTCCAAAGCCATGCTCAACCGCCTGAGGGCGTGCCTGCCGGACGATGCCTGTAATGGCAGCCGGGTGGTCCTGGTGCTGGACATGGAGAGCGAAGTGGTGGCACAGCGGGCCAACTCCATGAACGCCGGAGGCATCAGCGGGATCCATCTGCTGAGACGCCTGGACCAAGAAAGGAGCGGGCGGTTGTTCTGCGCCAGGGCCTTCGCCAAACAAGTGCGACTGTCGCCTGATTATCCCGTCGAGGAGAAGAAACTGACGAAAATGTACAGCAGAGCTGTGCATGCGATAACAGGGGGCCACCCACTGGCCATCGTGCTTCTGGCCGGGCTCCTCCGATTCAAGGAGAGGCCGGGACAGTGGAAAGCTGTGCTGCAGCAGCTCATGGTGTCATCGTCGTCGTCAGGACCTGCAGCAGTGGAAGACGAAGAAGAAGCAGGGAGCAACGACCAGATGGTGACGATAACCGCTGGAGGTGGAGGAGCTGCAGCGAGCAGGAGGACAGCCCTCGAGAGGGTCTTCTGGGAAAGCTTTGAGAACCTTCCCGACGGCCTCAAGTCGTGCCTCCTCTACTTCGCAGCTCAGTCCAAGGACACGGCGTGGACTCCCGACATGATCGTTCGGATGTGGATTGCCGAAGGCTTCATCAAGGCGCAGAAGGGCAAGACCGTGGAGGAGGTGGGTCACAGCTACCTCAAGGAGCTCACCCTGAGATGCCTTGTCCAGTCTTTCTACACCAACGACGCTATCGGCACCAAGGGGGTCCAGGTTCACAGGAGCCTGCACGGCTTCCTGCTGTCGGAGGCTCGCGAGGCTGGATTCATGGAGGTACATGAGACGCAGGACGACTTCATTCCATCGTCGGTGCGCCGCCTCTCGTCCTACATCTCCGACGGCGGATTCACCACTTTCGCCAACAAGTTCTCCAAGCTGCGTTCCTTCCTATGCTGGGCCAGTGACGACTACAGTGCTACTGCAGGCGGAAGCATTAATACGGATAAGAATCTTGATGATCTCAAGTTCCTGCTTAGGTCCAAGTTCCTCCGTGTCATCGAGATC

>B97_Zm00018ab231170

ATGCTGCTGCATCGTGAAGATGGCAATTGGCAAAACTTAATGTTCATCTCCATACTCGGGGAGAGTGGTGTGGGCAAGTACACCCTTGTAGACAAGATCTTCTTTGATCTGGCAGAAGACGACACCACAAAAAAACGAGAAATTTTCGTGGGCGAAAGAAATAAGTTTGCTGTTAAAGTCTGGTACATGATGCCTCCAGGTTCCAGCACAGAACATCTCCTCCGAATTATCTACGAGAGAGTAGAAGAGCAAGTACTTCGTTGGCAAGCCGAAGGAGATGCGGCAACAGAAGATATCCCTGACAGGATCCGTCACCTCCTGGCAAATACAAAGTACATATTGGTTATCACTGGCGTCTCCTCCAAAGCCATGCTCAACCGCCTGAGGGCGTGCCTGCCGGACGATGCCTGTAATGGCAGCCGGGTGGTCCTGGTGCTGGACATGGAGAGCGAAGTGGTGGCACAGCGGGCCAACTCCATGAACGCCGGAGGCATCAGCGGGATCCATCTGCTGAGACGCCTGGACCAAGAAAGGAGCGGGCGGTTGTTCTGCGCCAGGGCCTTCGCCAAACAAGTGCGACTGTCGCCTGATTATCCCGTCGAGGAGAAGAAACTGACGAAAATGTACAGCAGAGCTGTGCATGCGATAACAGGGGGCCACCCACTGGCCATCGTGCTTCTGGCCGGGCTCCTCCGATTCAAGGAGAGGCCGGGACAGTGGAAAGCTGTGCTGCAGCAGCTCATGGTGTCATCGTCGTCGTCAGGACTTGCAGCATTGGAAGACGAAGAAGCAGCAGGGAGCAGCGACCAGATGGTGACGATAACCGCTGGAGGTGGAGGAGCTGCAGCGAGCAGGAGGACAGCCCTCGAGAGGGTCTTGTGGGAAAGCTTTGAGAACCTTCCCGACGGCCTCAAGTCGTGCCTCCTCTACTTCGCAGCTCAGCCCAAGGACACGGCGTGGACTCCCGACATGATCGTGCGGATGTGGATTGCCGAAGGCTTCATCAAGGCGCAGAAGGGCAAGACCGTGGAGGAGGTGGGTCACAGCTACCTCAAGGAGCTCACCCTGAGATGCCTTGTCCAGTCTTTCTACACCAACGACGCTATCGGCACCAAGGGGGTCCAGGTTCACAGGAGCCTGCACGGCTTCCTGCTGTCGGAGGCTCGCGAGGCTGGATTCATGGAGGTTCATGAGACGCAGGACGACTTCATTCCATCGTCGGTGCGCCGCCTCTCGTCCTACATCTCCGACGGCGGATTCACCACTTTCGCCAACAAGTTCTCCAAGCTGCGTTCCTTCCTATGCTGGGCCAGTGACGACTACAGCGCTACTGCAGGCGGAAGCATTAATACGGATAAGAATCTTGATGATCTCAAGTTCCTGCTTAGGTCCAAGTTCCTCCGTGTCATCGAGATCAAGGGGCTGAGGATCAAGGAGCTGCCGGCTGAGATCTGCGACATGTTCCACCTGCGGTACCTTGGTGTTCACAGCCCGGGCCTCGAGAAGCTCCCACCCGCCATTGCCAAGCTGATCAACCTGCAGACACTGGACATAAGGGGCACTGCAGTCAAGAAGATCGACCCGTCCTTCTGGAACATAAAGACGCTGCGCCATGTCCTTGCCCAGGACCTCGAGCTTCCAGCGTCGTCGTCCATCGACCAACAGCTGGACGGCCTGCAGACGCTGCAGGGAGTCAGGCCTGCCGCCGAAGAAGAGGGGGACGGCCTGCAGACGCTGCAGGGTGTCAGGCCTGCCGCCGAAGAAGAGGGGGACGGCCTGCAGATGCAGGGTGTCAGGCCTACCGCCGAAGAAGAGGGGGACGGCCTGCAGACGCTGCAGGGTGTCAGGCCTGCCGCCGCAAAAGGGTGGAATCAAGAGAATTGCCCACTGCACAAGATGACCCAGCTCCGCACGCTGTGCCTGCACGGAATCAAAATCGACAAGCACAGGGATGCACTGGGAACTGCACTCCTCAAGATGCATCTCCTGCGCCACTTGGAGCTGCATGGTCATGATTATGAGCTGCATGGCTACGATGCTCATCGCGACGATGAGTTTCCTCCATGCATTTTCAAGGACCGGGGGCTTCAAAATCTTGAGACCGTAAAGCTGTATGGGATATACCATTTGCCTCATGACGACGACGCCGCATCCAATCTTCGTGTGCTCCGACCCAACCTCGCTTGGGTCGATTTGAAGTATACCTACTGTTGGCTGCCTCAACACATCGAAGACAAGTTCAAGAAGATGGGAGTGAGAGGTCAGTGGCTGAAGCTCAATACATCGTATTACTCAAGCTAG

>B97_Zm00018ab235820

ATGGCAGATGCCCTCTTGGTCGTTCTCAGAAAAGTTGCTCTGTTCCTGGGAGAAGGAGCACTAGAAAAGATTGGCAAAGCGGTAGTCGAAGCAGCACCCCTCATGACAGATTTTGAGCATAGCATGAAACAAATAGAGGGTGAACTCTCGGTTCTGCAGGCCTTTATTAACCAAGTTAGTGCACAGAGAGTCAGTGACAAGGCATTTGATGCATGGTTGAACCAAGTCAGAGATGCTGCCCATGAGATAGAAGACATCATTGATGAGTACGCTTACCTTACTGCACAAGCTGTTGATACGAACAGCTTCTTCAAGAGAAAGTTCCACCAGATCAAGAACATTGCAGCATGGCAGAAGTTCCCAAGCCAGATCAGTCAAGTAGAAGCAAGGATTCAGAGGCTATCAGAAATGAGGAACCGATATGGTTTCTCGCTGGGTGAAATAGACAGGAATAACAATTTTCAGCTCTCCAGTCACTTTTGTCTGTCAGATTCTGCTTACCTGATAGATAACTCTGAGATAGTAGGAAATGCCGATGAAATCGGAAAACTGACACAATGGCTACTTGAGGAGAAACAAGATCGGTCTCTAATTGCCATCCTTGGTATGGGAGGTTTAGGAAAAACTGCTGTTGCAAGCACCGTCTACAAGAACCAAAAGATCATAACATCTTTTGACTGTCACGCATGGGTTATTGTATCTCAGACTTACCAAGTCGAGGAACTACTAAGAGAAATTATAAATCAGCTAATAATAAAGGAAAGAGCAAGCATGGCAAGTGGCTTCATGACCATGAGTCGCATCAGATTAGTTGAGGTAATACAAAGCTATTTGCAGGATAAAAAATACTTTGTTGTCCTGGATGATGTATGGGATAAAGATGTTTGGTTAATTTTGAACTATGCATTTGTCAGAAACAGACATGGTAGTAAAGTGCTGATAACAAGTCGGAGAAAAGATGTGTCGTCTTTGGCAGCTGACAAATATGTCATTGAACTTAAAACCCTTAAAGATGTTGAATCTTGGGAGCTGTTTTGTAAGAAGGCATTTCATGCTTCAGAAGATAACATTTGTCCTGAAAATATAAGATATTGGGCAAATAAAATTGTTGCAAAGTGCCAAGGATTGCCACTGGCCATTGTAACTATTGGCAGTATTCTGTCATACCGTGACTTAAAGGAACAGGAGTGGGCATTTTTCTTCAACCAACTTAGCTGGCAATTAGCCAACAATCCAGAGCTCAACTGGATTTCCTGTGTATTGAAGTTGAGCTTGAATGATCTACCAAGTTATCTTAGGAGCTGCTTCCTATACTGCAGCATCTTTCCTGAAGATTACAAGATTAGAAGAAAGATGATTTCCAAGCTATGGATAGCGGAAGGTCTTGTGGAAGAGAGAGGAGACGGAACAACGATGGAGGAAGTTGCCGAGTGTTACCTTATGGAGCTCACTCAACGTTCTCTTTTTGAGGTCACAGAAAGGAAAACATGTGGAAGAGCTAGAACATTTCTGATGCATGATCTTGTGCGAGAGGTAACTTCAATCATTGCTAAAAAGGAGAAGTTTAGCATTGCACTTGCACATGGTGGTGCCAGTACAACCCAAGTTGCCCATGAAGCACGCCGCCTTTGCATCCAAAGAGGTGCCCAGACCATTAATTCTTTAAGAAGCTCGCGGCTTCGCTCATTCATTTTGTTTGACGCTGAAGTACCATGTTCTTGGATACATGATACTGTATCATGTTTCAGACTACTGAGAGTTCTATGCCTAAGATTTGTCAATGTTGAACAAGTTCCAAGTGTAGTCACAGAACTGTATAACTTGCGCTATCTAGATATGTCATACACAAAAGTGAAGACGGTCCCTGCATCGTTCGGAAAGCTCGTTAACCTACAATCTTTGGATCTTAGAGAGACCTACGTGGAGGAGTTACCACTGGAAATAACTAGGCTAACTAAATTACGGCAGTTACAGGTGTATGCACTCTATGATATTCTACAAAGATCATCGAAATTTCTCAGTGCTACAAAAATTCCTGGTAACATTTGTCATCTAAAGGATCTCCAAACTTTGCATGTTGTTTCAGCCAATAAAGTTTTGGTTTCACAGCTGGGGAACTTGAAGTTAATGAGAAGTTTGGCTATCGCGGAAGTGCAACAAAGCTACATTGCAGAATTATGCAACTCGCTGACAAAGATGACTAACCTGAAAACACTATTTATTTCCACGTGCAATGTGAATGAGACTCTCGACATAGAAATGCTAAAGCCGCTGCCAAATCTGACATCATTTCTCCTATCAGGAAAGTTGGAGAGAGGCTTGCCCCCGTCGATATTTTCTATGAATTTAAAACAGTTAAAATTGTTCGGGTCTAGTCTGAAGAAGGATCCTGTTAGCTCGTTCTCTCATATGCTAAATCTTGTTAATCTATTCCTCACTGGAGCATATGATGGGGAACAGCTAACTTTTTGCACCAGATGGTTCCCCAATCTCAAATATCTGCAATTAGCTGATATGGAACATCTGAATTGGATTGAGCTAGAGGATGGAACAATGATGAATCTACAATATTTGTCACTTGCTGGTTTAAGGAATCTAAAGGCTGTACCTGAGGGCATCAAGTACATTAGGGCACTCCATGAGATGCTTCTGACAGATATGCAAAATGAGTTCATGCTAAAACTGCATGGAAGTGACAATCACATTGTTCAACACATACCCAACATAAAAAAGTATGACTCTTCTGATTCTCAAGCAGTAAACAACCTGGCTTATCTGCCGTGGCTTGCCAATAAGTTTGGTCCTGGTGCTGCAATTAAGTATGCCTCCATAAACTGTGGCTCATCTGGCTCTTGA

>B97_Zm00018ab235850

ATGGCAGATGCCCTCTTGGTCGTTCTCAGAAAAGTTGCTCTGTCCTTGGCAGAAGGAGCACTAGAAAAGATTGGCAAAGAGGTGGTCGAAGCAGCACCCCTCATGACAGATTTTGAGCATAGCATGAAACAAATTGAGGGTGAACTCTCGGTTCTGCAAGCCTTCATTAACCAAGTTAGTGCACAGAGAGTCAGTGACAAGGCATTTGATGCATGGTTGGACCAAGTCAGAGATGTTGCCCATGAGGTAGAAGACATCATTGATGAGTATGCTTACCTTACTGCACAAGCCGTTGATACGAGCAGCTTCTTCAAGAGAAAGTTCCACCAGTTCAAGGGCATTGCAGCATGGAAGAAGTTCCCTGGCCAGATCAGTCAAGTAGAAGCAAGGATTCAGAGGCTATCAGAAATGAGGAACCGATATGGTTTCTCAGTCAGTCAACTAGACAGGACTAACAATTTTCAGCTCTCCATTCAGTTTTCTCTGTCAAATTCTGCCTACCTGATAGATAACTCTGAGATAGTAGGAAATGCTGATGAAATCGGAAAACTGACACAATGGCTACTTGAGGAGAAACAAGACCGATCTCTAATTGCCATCCTTGGTATGGGAGGTTTAGGAAAAACTGCTATTGCAAGCACCGTCTACAAGAACCAAAAAATCATAACATCTTTCGACTGTCACGCATGGGTTATTGTCTCTCAGACTTACCAAGTCGAGGAGCTACTAAGAGAAATTATAAATCAGCTAATAATAAAAGAAAGAGCAAGCATGGCAAGTGGCTTCATGACCATGAGTCGCATGAGATTAGTTGAGGTAATACAAAGCTATTTGAGGGACAAAAAATACTTCGTTGTCCTGGATGATGTATGGGACAAAGATGCTTGGTTATTTTTGAACTATGCATTCGTCAGAAACGAACGTGGAAGTAAAGTGCTGATAACAACCCGGAGAAAAGATGTGTCTTCTTTGGCAGCCGACAACTATGTCATTGAACTTAAAACCCTTAAAGATGCTGAATCTTGGGAGCTGTTTTGTAAGAAGGCATTTCATGCTTCAGAAGATAACATTTGTCCTGAAAATATAAGATGTTGGGCAACCAAAATTGTTGCAAAGTGCCAAGGATTGCCACTAGCCATTGTAACTATTGGCAGTATTCTGTCATACCGTGACTTAAAGGAACATGAGTGGGCATTTTTCTTCAAACAACTTAGCTGGCAGTTAGCCAACAATCCAGAGCTCAGCTGGATTTCCAGTGTCTTGAAGTTGAGCTTGAATGATCTACCAAGTTATCTTAGGAGCTGCTTCCTCTACTGCAGCATCTTTCCTGAAGATTATAAGATTAGAAGAAAGCTGATTTCCAAGCTATGGATAGCGGAAGGTCTTGTGGAAGAGAGAGGAGACGGAACAACAATGGAGGAAGTTGCTGAGTGTTACCTAATGGAGCTCACTCAACGCTCTCTTCTTCAGGTCACAGAAAGGAAAGCATGTGGAAGAGCTAGAACATTTTTGATGCATGATCTTGTGAGAGAGGTAACTTCAACCATTGCTAAAAAGGAGAAGTTTAGCGTTGCACTTGCACATGGTGGTGCCAGTACAAGCCAAGTTGCCCATGAAGCTCGTCGCCTATGCATCCAGAGAGGTGCCCAGACCTTGAATTCTTTCAGTAGCTCATGGCTCCGCTCATTCATTTTGTTTGACACTGAAGTACCATGTTCTTGGATACATGATACTGTATCATGTTTCAGACTACTGAGAGTCCTATGCCTAAGATTTGTCAATGTTGAACAAGTGCCAGGTGTAATCACAGAACTGTATAACTTGCGCTATCTAGACATGTCTTACACAAAAGTGAAGATGATACCAGCATCAGTTGGAAAGCTCGTTAACCTACAAGTTTTGGATCTCAGAGACACCTATGTGGAGGAGTTACCACTGGAAATAACTATGCTAACTAAATTACGGCAGTTACAGGTGTATGCACTCTATGATATTCTACAAAGATCACCGAACAGCTTCAGTGCTACAAAAATTTTTGGTAACATTTGTCATCTAAAGAATCTCCAAGCTTTGCAGGTTGTTTCAGCCAATAAAGATTTGGTTTCACAGCTAGGGAACTTGAATTTAATGAGAAGTTTGGCTATTGCGGAAGTGCGACAAAGCTACATTGCAGAGTTATGGAACTCGCTGACAAAGATGCCTAACCTGAAAAGACTAATTATTTCCACGTGCAATGTGAATGAGACTCTGGACATGGAAATGCTAAAGCCACTGCCAAATCTGACAACATTTGTCCTATCAGGAAAGTTGGAGAGAGGCTTGCTCCCATCGATATTTTCTGTGAAATTAAAGCAATTAAAATTGGACTGGTCTAGTCTGAAGAAGGATCCTGTTAGCTCGCTCTCTCATATGTTAAATCTTGTTGATCTATTCCTCACTGGAGGATATGCTGGGGAACAACTAACTTTTCGCAACAGATGGTTTCCCAATCTAAAATGTCTGCAATTAGCTGACATGGAACATCTGAATTGGATTGAGGTAGAGGATGGAACAATGATGAATCTACAATGTTTGTCACTTGCTGGTCTAAGGAATCTAAAGGCTGTACCTGACGGAATCAAGTACATTAGGGCACTCCATGAGATGTTTCTAACAGATATGTCAAATGAGTTCATAGTAAGACTGCATGGAAGTGACAATCACATTGTTCAACACATACCCAACATCAAAAAGTTTGAGTCTTCTGATTCTCAAGCAGTAAACAACGTTTATATACCGTGGCTTGCCGAGAAGTTTGGTTCTGGTGCTGTGGCTCATCTGGCTCTTGATGGTTCAATGGGAGTTGTTTGTGCCAGTGGTAATCATTTTAAGTCTGAAGAGTGA

>B97_Zm00018ab235860

ATGGCAGATGCCCTATTTGTAGTTCTTAGTAAACTTGCTGATTCCTTGGGAGAACAGACACTAGAGAGGATTAGCACAAAGCTGATTGAAGTAGCACCAGTTTTGACAGATTTTGAGCATAGCATGAAGCAAATCGAGGCTGAGCTATTGATTCTTCAGGCTTTTATTGCACAGGTTGGGACAAAAGTCGGTGATAAGGCATTTGATGCCTGGCTGGACCAAGTGAGAGATGTTGCCCATGAGGTAGAAGACATCATTGATGAGTATGCTTACCTTGAGGTTCAAGCCGTGGATACTGGCAGCTTCTTTAAGAGGAAGTTCCTTCAGATAAAAAAGTTCGCTGCATGGCAGAAGTTTCATAGCCAGATCAGTCATGTAGAAGCTCGAATTCAGAGGCTAGGTGAAATTAGGAATCGATATGGCATCTTGTCAGGTGAAATAGACAGGAGTAAGAAATTGAGGAGCCCCAATCGGCTCTTTATGTCAGATTCTTCTTACTTAACTGATAACTCTGAAATAGTGGGGCATGTTGATGAAATTGGAAGATTGACACAGTGGTTACTTGAGTACAAACAAGAGCGAACTCTTATTGCCGTTTTTGGTATGGGAGGTTCAGGAAAAACTACTATTGCAAGCAGTGCATACAAGAGTCAAAAGATCACAAGGACTTTCAATTGTCATGCATGGGTTACTGTATCTCAGACTTACCAAGTTGAAGAACTACTAAGAGAAATCATAAATCAGCTAATAGATCAGAGAGCAAGCATGTCAAGTGGGTTTATGACTATGAGCGGCTTGAGGCTAGTTGAGGTAATTCAAAGCTATTTGCAAGATAAAAAATATTTCATTGTCTTGGATGATGTATGGGATAAAGATGCATGGCTATTTCTAAACTATGCATTTGTCAGAAACAATTGTGGAAGCAAAGTGCTGATAACTACTCGGAGAAAGGACGTATCTAGCTTGGCAGTTGATCAGTATACAATTGAGCTTAAAACTCTTCAATATGCTGAATCCTGGGAACTTTTCTGTAAAAAGGCATTTCGTGCATCAAAGGATAACCAATGTCCTGAGAACCTTAGGTTTTGTGCAGAGAAAATTGTTGCTAGGTGTCAGGGATTGCCCCTGGCTATTGTAACCATTGGAAGTGTTCTATCATACCATGAATTCGAAGAGCAGGGATGGGAATCTTTCTACAGCCAACTTAGCTGGCAGTTAGCTAACAATCCGGAGCTGAATTGGATTTCTAATGTTCTGAATATGAGCTTGAATGATCTCCCGAGTTATCTGAGGAACTGCTTTCTGTACTGCAGTCTTTATCCTGAAGATTACAAGATTAAAAGAAAAGTGATTTCCAAGTTATGGATAGCAGAAGGTTTAGTGGAAGATAGAGAAGATGGAACAACAATGGAGGAGGTTGCCAATTACTACCTTGTGGAGCTCACTCAGCGTTGTCTTCTTCGAGTCACGGAAAGTAATGCATGTGGAAGGCCGAGAGCTTTTGTTATGCATGATCTTGTGCGAGAGTTAACCTCCAACATTGCTAAAAAGGAGAAGTTTGGTATTGCATATGGCGATACTAGTACAACCCAAGTTCCCCCTGAAGTTCGCCGTTTATGCATACAAAGAGGTGCCCAAACCTTGAATTCTATAGGTAGTTCGCGGCTCCGCTCATTTATTTTGTTTGACACTGAAGTACCATGCTCTTGGATAGATGATGTTTTGTCACGTTTCAGACTACTGAGGGTCCTATGCCTAAGATTTGCAAATATTGCAGAAGTGCCTGGCGTGGTCACAGAATTGTATAACTTGCGCTATATTGATTTTTCATACACAAAAGTGAAGACAATACCAGCATCATTCAGAAAACTTGTCAACCTACAAGTTTTGGATCTCAGATTCACCTACGTAGAGGAGTTGCCACTGGAAATAACTACGCTAACTAACTTGCGTCATCTACATGTGTTTGCAGTCCATGATTTTCAACAAAGATCATTGAATTGGTTAGGCGCAACAAAAATTCCTGTTAACATTTGTCATCTAAAGAATCTGCAAGCTATACAGATTGTTTTGGCCAACAAAGATTTGGTTTCACAACTTGGGAACTTGAAATTAATGAGAAGTTTGGCTATAGCAGAAGTGCGGCAAAGCTACATTGCAGAATTATGGAAGTCCCTGACAAAGATGCCTAACCTGAATAGGTTGGCTATTTCAACATGCAACATGGAGGAGATTCTTGATTTTAAAATGCTAAAGCCCTTATCAAATCTGGTTTTCTTCAAGCTTGCAGGAAAGTTGGAGTCAGGCGTGCTTCCTTTGATGTTGTATTATTTTGAGAAGTTAACATGGTTACAATTAGACTGGTCCGGTCTAAAGAAGGACCCTATGAGCTCCTTATCTCACATGTCGAACCTTGTTCATCTGTTCATGTGCGGGTCATATTGTGGGGAACAGCTAACTTTTTGTTCAGGATGGTTCCCCAAGCTCAATTATCTGCAATTATCTAAAATGGAGAATCTGAATTGGATTGAGATAGAGGATGGAACAATGATGTGTCTAAATAATTTGTACTTAGTTGATTTAGGGAATCTAAAGGCTGTGCCCTATGGCATCAAGTACATCAGGACACTGCACCAGATGCATCTGACAGATATGTCAAAGGAGTTTCTAGGAAGTCTGCAAGGAAGCGCGAGTCCCGTTGTTCAACATATATGCAACATCCATATTTTTGAATCCTCTGATTCTGAAGCAGTAAATAAATTCTTCTTTGAGCCGTACCTTGCCACCAAGTTCGGCCCTGGTGCAACTAAGCATGCCCCTACTTACCTGGGATCATCTGGCATCTGA

>B97_Zm00018ab235870

ATGGCAGACGCGCTGTTTGTTGTTCTTAGGAAAGTTGCCCTTTCCCTGGGAGAAGGCGCGCTGGTGAAGATTGGCACGGACGTGGTCGAGGCAGCGCCCATCTTGACAGATTTTGAGCATGGCATGAGACAAACCGAGGGCGAGCTTTTGGTCCTGCAGGCCTTTATCGGGCAGGTCCGGGCGCAGAAGGCCGGTGACAAGGCGTTCGGCGCATGGTTGGACCAGGTTAGAGATGTCGCCCATGAGGTAGAAGATATCGTTGACGAGTATGCTTACCTTACCACGCAAGCCATGGATGCAAGCAGCTTCTTCAAGAGAAAGTTCCATCAGGTGAAGAACTTTGCAGCGTGGCAGAAGCTACCGATCCGGATCAGTCAAGTGGAAGCTCGGGTCCGGAGGCTGTCCGAAATGAGGAGCCGGTATGGGATTTCGGTAGGCGAACAAGACAGGGGTAGCAAGTTACAGCAATCCAATCAGTTCTCTGCGTCAGATTTTGCTTACCTAACTGATGATTCTGAAATAGTAGGGCATGGCGAGGAAATCGAGAGACTGACACAGTGGCTGCTTGAGGAGAATCAGGACCGAACTCTGATAGCCATCTTTGGTATGGGTGGTCTAGGTAAAACAACTGTTGCAAGCAGTGTCTACAAGAACCAAAAGATCAGGAGAACTTTTGATTGCCATGCATGGGTCACTGTATCTCAGACTTACCAAGCTGAAGAGCTTCTGAGAGAAATCATGAACCAGTTAATAGAGCAGAGAGCAAGTTTGGCAAGTGGCTTCATGACCATGAGTCGCATGAGATTAGTTGAGATGATACAGAACTATTTGCGGGACAAAAAATATTTCATTGTCTTGGATGATGTATGGGAAAAAGATGCTTGGTTGTTTCTGAACTATGCATTTGCCAGAAACAATTGTGGAAGTAAAGTGCTGATAACAACCCGGAGAAAAGATGTGTCTTCTTTGACAGTTCACAGTCGTGTAATTGAACTTAAAACTCTTAACTATGCTGAATCATGGGAACTCTTCTGCAAAAAGGCATTTTTTGCATTGGAGGGCAACATATGCCCTAAGAATCTCACGTCTTTGGCGGAGAAAGTTGTTGATAAGTGTCAAGGATTGCCATTGGCTATCATAGCCATCGGAAGGATTCTATCATGCCATGGATTAGATGAATGGGAGTGGGCATTTTTCTACAACCAACTTAATTGGCAGTTAGCTAACAATTCAGAGCTAAGCTGGATCTCTACTGTCTTGAATCTGAGCTTGGATGATCTCCCAAGTCATCTGAGGAGCTGCTTTCTGTACTGCAGCTTATTTCCTGAAGATCATTGGATTAAAAGAAAACAGATAGCCAAGTTATGGATTGCGGAAGGTCTTGTGGACGAGAGAGGGGATGGGACAACAATGGAGGAAGTTGCTGAGCATTACCTTGCAGAGCTAACTCACCGTTCTCTTCTTCAGGTCATAGAAAGAAATGCAAGCGGAAGGCCAAGAACGTTTGTTATGCATGATCTTGTGCGGGAAGTGACCTCAATAACCGCTGAAAAGGAAAAGTTCGCGGTGATACATGGCCATGTTGGTACAACCCAAGTTTCCCATGATGCACGCCGCTTGTGCATCCAAAAAAGTGCAGATTCTCAAAACTCTTTAGCAAATTCACATCTTCGATCATTCATTTTATTTGACAATTTAGTACCGTCTTCCTGGATAAATGATGTCTCATCACGTTTCAGACTGCTGAGGGTCTTAGGCCTAAGATTTACTAATATTGAACAAGTGCCGTGCGGGGTCACAGAACTGTATAACTTGCGTTATCTGGATATTTCGTACACAAAAGTCAAGCGGATACCGGCATCATTCAGAAAACTCATGCACCTTCAAGTTTTAGATCTTAGGTTCACCTACGTGGAGGAGTTGCCATTTGAAATAACTATGCTAACTAATTTACGCCATTTACATGTTGCTGCGGTCCATGATCTTCAAGAAAGATCACTGAATTGCTTCAGTGCTACAAAAATTCCTGGCAACATTTGTGGCCTAAAGAATCTTCAATCTTTACATACCGTTTCAGCTAATGAAGATTTGGTTTCACAGTTGGGAAATTTGACTCTTATGAGAAGCTTGACTATAATGAATGTGCGGCAAAGCTATATCGCAGAGCTATGGAACTCCCTGACAAAAATGCCTAACCTGAGTGTCCTGATTATTTTTGCATCTGATATGGATGAGATTCTTGATTTGAGAATGCTGAGGCCCTTACCTAACCTTAAGTTGTTCTGGCTGGCAGGAAAGATGAAGGGAGGCGTGCTCCCATCGATATTCAACAAGTTTGAGAAGCTAACACAGTTAAAAATGGACTGGTCTGGTCTGAATAAGGACCCTATAAGCTCCTTCTCTTACATGCTAACTCTAGTTGATGTGTGGTTCTTCGGGGCATATTGTGGGGAATATTTATCTTTTTGTGCAGGATGGTTCCCCAATCTCAAATCTCTGCACATTGCTGATATGGAACATCTGACTCGGATTGAGATAGAGGATGGAACGATGATGGGTCTGCATCATCTGGAACTTATTGGTTTTAGGAATATGAGGCTAGTTCCTAAGGGTATCAAGTATATTAGAACACTCCGTCAGATGATCCTAACTGATATGCCAAAGGAGTTAGTTGAAAGCCTACGAGGAAGTGACGCTCACATTGTTCAGCATGTTCCCAACATCCATATTTTTGACTCCAGTGATCCTGAAGCAGTAAATAACTTCATATTCTGGCCTCATCTTGCCAAGAAGTACGGCTCTGGTGTAACTAAGTATGACCCTAGAAAATGA

>B97_Zm00018ab263780

ATGGACGGGTTCATGGCAAGTGCAGCGACAGGGGTGATGAGCTCCCTTCTCGCCAAGCTCGCTGAGCTGCTTGGGGAGGACTACAAGATGCAGAGGGGCATGAGGCGCGAGATCGCGTTCCTCAAGGATGAGCTGGGCAGCATGAACGCGCTGCTGGAGAGGCTAGCCGGCTCGGAGGCGCTCGATCCGCAGACCAAGGAGTGGAGGGACCAGGTGAGGGAGATGAGCTACGACATCGAGGACTGTGTCGATGGTTACATGCGTCAGCTGCAGCACGAGCCGCAGAGGAACAGTGGAATCACGGGATTCTTCCTTGGGTATGTGCAGAAGGTGAAGGATCTCGTTACCCGTCACGAGATTGCCGAGCAGATTCAGGAGCTCAAGGCTCGGATCGTTGAGGCTAGCCACAGAAGGAAGAGGTACAAGATTGATGACACAGCTAATTCTGGTGCCGCCAATGTGATCCCTGTGGACCGTCGGTTGCCAGCGCTCTATGCAGAATTGGGTAGCCTTGTTGGTAGCGATGTTCCTAGAGATGAGATTATCAAGCTACTTGATGATGGGGCGCTGGCCGTAAAGGTGGTGTCTATTGTGGGCTGTGGAGGACTGGGAAAGACTACTGTCGTGAATCAGGTTTACATAAACATCGCCGAGAAATTTGATTGCCAAGCCTCTGTGTCCTTGTCCCAAAATCCTGATATGGTGAACATATTCCGGTCGATACTGTCTCAAGTCAAGAAAGATGAGTGTGGTAGCACCAGCTCATGTGACAAGGAACTTCTCATCAATGAATTGAGGGATTTCCTTAAGGACAAGAGGTATTTTATTGTAATTGATGACATATGGAGTACCCAAGCATGGAAGACAATTAAATTTGCTTTGGTTGAGAATACTTGCGGCAGTAGAGTAATAGTGACAACAAGAATTGGTACTATTGCCAAATCTTGTTCATCCCCATTCCATCATCTCGTATATGAATTGAGGATGCTAGGTGAAGATGACTCCAAAAGGCTATTCTTTAGAAGAATTTTTGGCTCTGAGGACAAGTGCCCTCACCATTTAAAAGAGGTTTCAGTTGAAATAATTAAGAAGTGTGGTGGTTTACCATTGGCAATCATTACTATGGCTAGTTTGTTGACTACTAAATCATATACCAGAGCTGACTGGTTGAAGGTTTCTAATTCAATTGGATCTGGGCTAGAGAAAAATTGTGATGTGGAGGAAATGAACATGATATTATCTCTGAGTTACAATCACCTTCCTCATCATTTAAAGACTTGTTTATTGTATCTAAGTATGTTTCCTGAAGATTATGTGATCAAGAGGGATTATTTGGTAAGAAGGTGGGTAGCAGAAGGATTTGTTAGTGCACATGGTAGAAGAAATCTGGACGATGAAGGCGAATGCTATTTTAATGAACTTATCAACAGAAGCTTAATACAACCGGTAGATTTTCAGTATGATGGTAGAGTATATGCATGCCGGGTTCATGATATGATTCTTGATCTGATTACATGCAAGGCTGTTGAAGAAAATTTCATAACTGTTGTTACTAATAGAAAACAAATGTTGCCCTCACATGGCAAGGTCCACCGACTGTCACTTGAGTACCATGGTCTTGAAACCTTAAGAACAAATCCCATTTTTACTACTCATGTTCGGTCCCTGAACATATTTAGATACTCTGAAGAAATGCTTCCTCTTTCAGGCTTGCACTCCCTAAGAGTGCTTGATCTAGATGGCAATGAGAATTTGGAAAGCTGTTACCTTGAAGATATAGGGAAGTTATATCAGTTGCGATACCTACGGATTAAGGCAAGTAATATTACACTTCTGGAAAGGATAGAAGAGCTTCAGTGTTTGGTAATACTGGATCTTCTGAATTGCCCTAATCTAGGTGAATTGCCTAGAAGTATTGTTCAACTTCGGAACTTGAAATGGTTAACTGTTCATCGAGCGAACTTGCCAGATGGAGTTGGGAACATGCAAGCGCTAGAGTTTCTTTCACTTGTAGTTGTGGACTACACTACCTCAACAAACTTATTGGAAGAGCTGGGCAGCTTGACCAAATTGAGAACTCTTAGGTTGGATTGGCGCATCAACCCCCTGCACAGGGATAAAAAAACATATGAGGGTAATTTTGTTTCTTCACTTGGCAAACTAGGCAGTTCAAACCTTCGATACCTAACACTCATCAGTCCATGGTCACTCGACTTCCTGTTGGAACCTTGGTCCCCAACTCCACATTTCCTTCAGGAGTTAGTGATCAAAGGATGGCATCTCAACAATATTCCAGTCTGGATGGCCTCGCTAACCAACCTCACCTACCTGGACGTTGAGGTTAAAGTTAGACAAGAAACTCTCCAGATCCTCGGAGATTTCCCTGCCTTACAATTCCTGAAGGTGTCCTCAAATGCAGCAGGATCTGAGGCAATGTGCCTTGTCGTCAGCAACGATGGATTCCGATGTCTGAAGAAGTTCAGTTTCGTTGGCTGGGTAAACATGATGTTCAAAGAAGGAGCTGTTCCAGCGCTTGAAACTCTTGAGTTTCAAATCATAGCGCACGAGATGCATACTGCACGCAGATTTGGTCCTCCTGATTTCGGCATCAGCCACCTCTCCACCCTCAGGAATCTCGTCGTCAATGTTCACTGTGAAGGTTCAAGGGTTAAAGAGGTGGAGGCAGTAGAGGCTGCTATCCAGAAGTCAGCCAGTACGCTTCCTAATTATCCCACACTAGGTTTGCACAGATTTCTCGAGTCAGAACTGGTAAAAGAATGA

>B97_Zm00018ab273190

ATGGCAGAGATTGCTGTTCTTCTTGTTCTAAAAAAGATTGCCATAGCTCTGGCAGGAGATACCCTAAGTTTTGCCAAACCTTTGTTTGCAAAGAAATCTGAGTTGGTGGCAGCACTCCCAAATGACATGAAACTGATTAGTAATGAGCTTGAGCTTATTCGGGCATTTCTTAAGGAAATTGGCCGTAAGGGCTGGAAAGGTGAAGTGATAGAAACATGGATAGGGCAAGTCCGAAGACTGGCTTACAATATGGAAGACACTGTAGACCATTTTATTTATGTAGTTGGCACTCGCAATCAGATAGGATCATGCTGGGATTACATGAAGAAGATAGCCAAGAAGCCTCAGTGTCTTGTTTCACTAGATGAAATTGCTAGTGAGATTAAGAAGATAAAGCAAGAGCTTAAACAAATCTCAGAAAGTAGAGACCGCTGGACCAAACCCTTGGATGGCGGGACAGATATACCTGCAGGAAGCTATCAAACTGAAAAGGAAATATATCTTCCGGGACACGATTACTCAATTAGAGACGAGGAGCTTGCAGGAATTGGTAAAAATAAGCAAACCTTGATTAGTTCGTTAAAATTTGGAGATCCATCACTTCGGATCGCTGCTGTCTGGGGTATGGGTGGCATTGGAAAAAGCACTCTTGTCAATAATGTGTACAAAAATGAAGGGTCCAAATTTGGCTGTCGTGCATGGGTTTCTATCTCTCAGTCATACAAACTAGAAGATATATGGAAGAAAATGCTGACAGATCTCGTCGGTAAAGATAATAAAGAATTTGATTTTGGAACAATGAATAGTGCAGAACTAACAGAGCAACTGATAGAAACTCTAGACAAAAGGCAGTACTTGATCATACTGGATGATGTCTGGACGGCTGATGTTTTTTTTAGAATTAAAGAAGTTCTTGTAGATAATGGCTTTGGAAGCAGAGTAATAATCACGACAAGAATTGAAGAGGTAGCTTCACTAGCTGAGGATAGTTGTAAGATCAAAATAGAACCTCTTGGTGTCGATGATTCCTGGCATGTATTTTGTAGGAAGGCATTTCCGAAAGTTGAAAACCATATCTGCCCTCCAGAGTTACATCAGTGTGGTATAAACATTGTGGAGAAATGTGATGGTTTGCCATTAGCCCTTGTGGCAATAGGAAGCGTATTGTCGCTGAGACCGAAGAATGTTGATGAGTGGAAGATATTTTATGATCAGCTTATCTGGGAGCTACACAACAATGAGAACATTAATCGTGTGGAGAAAATTATAAATCTAAGCTATAAATACCTACCAGACTATTTAAAGAACTGCTTCTTGTATTGTGCTATGTTTCCAGAAGATTATCTAATACACAGAAAGAGACTACTTAGATTGTGGATAGCTGAAGGATTTATTGAACAAAAAGGGGCATGCAGCTTAGAAGACACTGCCGAAAGTTATCTTAGAGAACTTATACGACGGAGCATGCTTCACGTTGCAGAGAGGAACAGCTTTGGTAGGATTAAATGTATTCAAATGCATGATCTTGTACGTGAACTAGCCATTTTCCAATGTAAAAGAGAAGGTTTCAGTACAACATATGATGGAAATAATGACATAATGCTAGTGGGATCAGATTCCCGACGAGTGGCTGTGCTCCAATGCGGCAAGGGCATTCTATCAACCATTGATCCATCCAGGCTTCGCACCTTCATAACATTTGACACCAGCAGGGAATTATCTTTGTGGTATTCTTCTATTTGCTCCAAACCAAAGTACCTTGCAGTATTAGACTTATCAGGGTTGCCTATTGAGACTATTCCAAATTCAATTGGGGAGCTATTCAACCTTAGGCTTTTGTGTCTCGATGACACCAAAGTGAAAGAGCTGCCTAAATCTTTTACAAAGCTTCGAAACCTACAGACATTGAGTCTTGAGCGTGCAGAGTTAGTGAAGTTCCCACAAGGGTTTTCAAACCTGAAAAAATTGAGACATCTTATGGTTTCACGGTTGCGAGATGTAACTTATAGGAGTTTAAAAAGTTGGGAAGCTCTGGAGCCATTTAAAGGCTTGTGGGGTTTGACTGAACTGCAAACTCTGTTTGCCATTACTGCAAGTGAAGTATTAGTTGCAAAACTTGGAAATTTATCCCAGCTGAGGAGCCTTGAAATTTGTGATGTAAGGAGTAACTTATGTGCACATTTATGTGGCTCTTTGTCAAAGATGTGCCAGCTGTCACGATTAACGATAAGAGCATGCAATGAAGATGAAGTGCTACAGCTGGATGATTTGACATTTCCAAATCCTCTTCAAACCCTTAGTTTAGATGGACGACTGTCAGAAGGAACTTTCACGTCTCCCTTTTTCTTAAATCACGGGAATGGGCTTCTTTGTCTAATGTTGTGGTACAGTCAGCTTTCAGAAAATCCAGTACCACGTCTCTCTAAATTATCAAACTTGACTAGGTTATCTCTTATAAAGGCATACACCGGCCAAGAATTATACTTCCAAGCAGGTTGGTTCCTGAATTTGAAGGAACTTTACATGAAGGATTTGCCCCATCTCAATCAAATACACATACAGGAGGGAGCTTTGCCCAGCCTTGAACTTATAGCAATTATTCACCTCCCAGATCTACGGCAGGCTCCAATCGGCTTCAGATTTCTCAAGTCCCTAGAAACAACATTTTTTTATGATATGCATCCTACTTTTGAAAGCAACATTGAAGAAAGAAATTTAGAGCGTATTGCATACATATTCGGGACAATGGCATAA

>B97_Zm00018ab273270

ATGGCAGAGATTGCTGTTCTTCTCGTCCTGAAAAAGATTGCCATAGCTCTGGCAGGAGAGACCCTAAGTTTTGCTAAACCATTGCTTGCAAAGAAGTCTGAGTCGGTGGCAGCACTCCCAGATGACATGAAACTGATTAGTAATGAGCTCGAGCTTATCCGGGCGTTTCTCAAGGAAATCGGCAGGAAAGGCTGGAAAAGCGAAGTGATAGAAACATGGATAGGGCAGGTCCGAAGACTGGCTTATGATATGGAAGACACTGTAGACCATTTTATTTATGTTGTTGGTACACACGATCAGATGGGATCATGCTGGGATTACATGAAGAAGATAGCCAAGAAGCCTCGGCGTCTGGTTTCACTAGATGAAATTGCTAGTGAGATTAAGAAGATAAAGCAAGAGCTTAAACAACTCTCGGAAAGTAGAGACCGCTGGACTAAACCCTTGGATGGCGGGAGTGGTATACCTGCAGGAAGCTATGAAACTGAAAAGGAAATGTATCTTCCTGGACATGATTACACAATCAGCGACGAGGAGCTTGCAGGAATTGATGAAAATAAGCAAACCTTAATTAGTTCATTAAAATTTGAAGATCCAACACTTCGGATCATTGCTGTCTGGGGTATGGGTGGCGTTGGAAAAAGCACTCTTGTAAATAATGTGTACAAAAATGAAGGATCCAACTTTGACTGCCGTGCATGGGTTTCTATCTCTCAGTCATATAGACTAGAAGATATATGGAAGAAAATGCTGACAGATCTCATCGGGAAAGATAAGATAGAATTTGATCTTGGAACAATGGATAGTGCAGAACTAAGAGAGCAATTGACAAAAACTCTAGACAAAAGGCAGTACCTGATCATACTGGATGATGTCTGGATGGCTAATGTTTTTTTTAAAATTAAAGAAGTTCTTGTAGATAATGGCCTTGGAAGCAGAGTAATAATCACAACAAGAATTGAGGAGGTAGCTTCACTAGCTAAGGGTAGTTGTAAGATCAAAGTAGAACCTCTGGGTGTCGATGATTCCTGGCATGTATTTTGTAGGAAGGCATTTCTGAAAGATGAAAACCATATCTGCCCTCCAGAGTTGCGTCAGTGTGGTATAAACATTGTGGAGAAATGTGATGGTTTGCCATTAGCCCTTGTGGCAATAGGAAGCATATTGTCGCTGAGACCGAAGAATGTTGACGAGTGGAAGCTATTTTATGACCAGCTTATCTGGGAGCTACACAACAATGAGAACCTTAATCGCGTGGAGAAAATTATGAATCTAAGTTATAAATACTTACCAGACTATTTGAAGAACTGCTTCCTGTATTGTGCTATGTTTCCAGAAGACTATCTAATACACAGAAAGAGATTGATTAGATTGTGGATAGCTGAAGGATTTATTGAACAAAAAGGGGCATGCAGCTTAGAAGACACTGCTGAAAGTTATCTTAAAGAACTTATACGACGGAGCATGCTTCACGTTGCAGAGAGGAACTGCTTTGGTAGGATTAAATGTATTCGAATGCATGATCTTGTGCGTGAACTTGCCATTTTCCAATCTAAAAGAGAGGGTTTCAGTACAACTTATGGTGGAAATAATGAAGCAGTGCTAGTGGGATCATATTCTCGACGAGTGGCTGTGCTCCAATGCAGCAAGGGCATTCCATCAACCATTGATCCATCCAGGCTTCGCACCTTAATTACATTCGACACCAGCAGAGCATTATCTGTGTGGTATTCTTCTATTTCCTCCAAACCAAAGTACCTTGCAGTATTAGACTTATCAAGCTTGCCTATTGAGACTATTCCAAATTCAATTGGAGAGCTTTTCAACCTTAGGCTTTTATGCCTCAATAAAACCAAAGTGAAAGAGCTCCCTAAATCTATTACAAAGCTTCAAAACCTACAGACAATGAGTCTTGAGAATGGGGAGTTAGTGAAGTTCCCACAAGGGTTTTCAAAACTGAAGAAATTGCGACATCTTATGGTTTCACGGTTGCAAGATGTAACTTTCAGCGGTTTCAAAAGTTGGGAAGCTGTGGAGCCATTTAAGGGCTTGTGGACTTTGATTGAACTGCAAACTCTGTATGCCATTACAGCAAGTGAAGTATTAGTTGCAAAACTTGGAAATTTATCCCAGCTGAGGCGGCTTATAATTTGTGATGTAAGGAGTAACTTATGTGCACAGTTGTGTGGCTCTTTGTCAAAGTTGTGCCAGCTGTCACGATTAACGATAAGAGCATGCAATGAAGATGAAGTGCTACAGCTGGATCATTTGACATTTCCAAATCCTCTTCAAACCCTTAGTTTAGATGGACGACTGTCAGAAGGAACTTTCAAATCTCCCTTTTTCTTAAATCATGGAAATGGGCTTCTTAGGCTAATGTTGTTTTACAGTCAGCTTTCAGAAAATCCAGTACCACACCTCTCTGAATTGTCAAACTTGACTAGGTTATCCCTTATAAAGGCATACACCGGCCAAGAATTATACTTCCAAGCAGGTTGGTTCCTGAATTTAAAAGAACTTTACTTGAAGAATTTGTCCCGGCTCAATCAAATAGATATACAGGAGGGAGCTTTGCCCAGCCTTGAACGTATAACAATGAAACACCTCCCGGAGCTACGGGAGGTTCCAGTCGGTTTCAGATTTCTCAAGTCCCTAAAAACAATATTTTTTTCCGATATGCATCCTGAGTTTGAAAGCAGCTTTCAAAAGGAAATGTAG

>B97_Zm00018ab284590

ATGGAGGGCGACAAGGAGGCATCCAAGCAGCCAGTTGTACTGTCTATTGTTGGCTTTGGAGGGTTAGGCAAGACTACTCTTGCTAATCTTGTGTATGAGAAGATTAAAGGGCAATTTGTCTGTGGGGCATTTGTTTATGTGTCTCATAATCCTGATGTCGTCAAGGTTTTCAAAAACATGCTCTACCAGCTTGATGGAGACAAATACAGGGACATCAATCAAGGAACATGGAGTGAAGAACAACTAATCTGGGAACTGAGGAAGTTCCTTCTACACAAGAGGTACTTTATTGTCATTGATGACATATGGAATACTTCTGTGTGGGAAACAATCCAATGTTCTTTGATGCACAATGAATGTGGAAGTATAATAATCATCACAACTCGTAATATTGATGTTGCAAAACAAGCTGGAAGTGTTTATCAAATGGAACCTCTTTCTCTCAGCGACTCAACAAAGTTATTCTGCCAAATAATTTTTGGCAGTGAAGACAAATGTCCTCCAGCTAATTTAGCTGAAGTGGCTGGTAAAATCTTACAGAAATGTGGTGGTGTACCATTAGCTATCATTACCATGGCAAGTATGCTAGCCAATAAAACTGGAAAGGAAATAAACACACATAGCTATTGGTCACATGTGTACCAATCCATGGGTTATGGTCTAGATGGCAGTACTAATGTGAAGAATATGAGAAGGATACTATCAGTTAGTTACTATGACCTACCTTCACATCTAAAGACTTGCTTGTTATACCTAAGTTTGTCTCCAGAGGACTACAGGATTAGAACAAGAGGTCTGATATGGAAATGGATTGGTGAAGGTTTTGTCCATGAAGAACAAGGGAAGAGCCTATATGAAGTAGGTGAGGATTATATTGAAGAGTTAGTTAACACAAGTATGTTAGAACCTGTCGGGATTGGCCATGATGGTAAGACCGTATCTTGTCGGATACATGATATGGTCCTTGATCTTATCAGTTTCTTGTCAAATGAGGAGCATTTTCTAACAAAAGTAGGTGGGCAACAACCCGTATCTCTTGATCTGCCTAAAAAGATCCGCCGGTTATCCCTCCAAATTAGCCAGGAAGAGGAAGCCAAGCAGCTAGCTACAATGAGCTTCTCCCACGTAAGGTCACTTACTGTGTCCACTGAAGTTTTCCAGTTGACGCCAAAACTTTCGGCCTTTCTGGTCTTACGTGTATTGAATTTAAAGAAATGTAATGGAGTGAACAATCACCACTTTAAAGATATTTGCAATATGTTTCAGCTGAGATATTTGAGTCTCAATGCGAAATTTATTACTGAGATCCCAAGGGAGATTCGGAATCTGCAATTTTTGCAAGTACTTGACATAACTAATCTTGGGCACAAAGTAAAGATGACAACCATTATTCACTTGCGACAGCTGTTGCGGCTTTGTTCTAGGTCTGGGTGGAGCATAAAACAGCTAGACGGATTTGGAAAACTAACCTCTCTACAAGAAGTTAAAGGGACCATAACTATCGAGTCACCAAGCATGCTGCATGATCTGGGGTGTCTGACCAATCTCAGGACCTTGGGCATCAACTTTCGTGATTGGGATGAGAGCTATGAGGAACCTTTCATCCAATGTCTATCTAACCTTGTCAGCCTCAAATCCATGAAAATAAAAGGTACCATGATGAGCAGCCTATGTTCCGAATGTGACAAATTGTACCCTGGTCCTCAACAACTTTGCTCCATTGATATGAAGTCACTTGCAAGAACTGTCATCATGACTAGAGTACCAAGATGGATGTCATCACTCTGCTCCCTGTCTAGCATAAAGATCACATTATTAGCTCTGGGAGTACAAGATATCCATGTCCTTGGGAGCATACCATCTCTACGTTGTCTCAGTGTACATGTGAAGGAAACCAGAGATGAAAGGTTGGTCATTGACAAGTGTTATCCATTCCGGTGCCTAACTGAGATGCAAATCGATTATGAATCCATGGCGGTGGTGTTCGCACCAGGGAGTATGCAAAACCTCAAAGAACTCCATTTATTGTTCGGGGTGAAAGAGGTAATGCATAAGTACGGTGATTGTAACTTTGGTTTGGAGCACCTCATGTCACTTGAGCATGTCTCTGTTAAAACAATGTACACTATCATGCCCGAGGAGGTGGAGGCCGTAAAAGATGAATTCCAGAAATCCCTGGACATGAATCCTGGCAAGCCCACATTGATAGTAGATTATTTGTATCCGAAAGAAAGGAAGATTAGGTCTCAAGCACAAGCAATAAGAGCAGCAATTTTGTTCGCTAATGCAGGCCGCATCCCTGCTACTGACTGA

>B97_Zm00018ab296850

ATGGAGCTGGCGGTGGGCGCGTCGGAGGCAACCCTCAAGTCACTGCTGAGCAAGCTCGGCGCCCTCCTCGCAGAGGAGTATGCCCTGATCCGTGGCGTCCGGGGCGACATCCAATTCATCACCGACGAGCTAGCCAGCATGCAGGCCTTCCTCAGCAACCTGAGCAAGTACGAGGAGGGCCACGACGACCAGACGGAGGACTGGATGAAGCAGATCCGGGACGTCGCCTACGACATCGAAGACTGCATCGACGACTTCGCCCACAGCCTCCGTCCAGACCCCAGGGGCAGCGGCTGGGTGACGGCCGTCCGCAAGATTCTCTACGAGATCCGGACGTGGTACCCCCGCCGCAACATCGCCACCCAGATCGTCGATCTGAAGAACCGCGCGCAGCATATCGGCGAGCGCCGAACCAGGTACGGCGTCCGCGACCCGCAGCCTGGGAAGAAGAAGAGCAACTTGGGGGGCGCTACGGGGTATCTCGCTGCTGAGAATCAGGACGTGACCCGGCAACTCGTCCGCGCCCAGCAGCCTGTGGGGGTGAAGGATATGCCGGATCTTAAGAAGTGGATCCATGAAGATGGCAAGAGGGAGAAAACCGGCGTGCTGAGCATTGTCGGGTTTGGTGGTGTGGGTAAAACCACCATCGCAATGGATCTCTACACAAAATACGGGCCTGAATTCCAGCGTCGAGCAATGGTCACTGTGTCCCAGAACTCAGATCCTGAGGCGGTCGTCAGAAATATACTGAACCAGGTCAAACCACTGTCCAACAACGCGGAGAGGCGAGGCGAAGATAGCTCTGGCGCCGTCTCCTTGGGGAAGAAGAATCCAGTCATTGCAACTATATTAAGCCGAATTAGGCTGCCGTTCCCAAATCAAAAGCAAGATAATGGTGGCGGTCGTGACAAGCATGAACAAATAAAAAAGGAGTTGAAGAATTGCCTCGCAGATACAAGGTACTTACTGTTAATTGATGATGTATGGTCATCATCAACATGGCAAAGTATTTGGAAATATTTTCCTGAAGAAAATAAGAAGGGAAGCAGAATAATTGTCACCACACGGTTTCAAGCTGTTGCCACGACATGCTCTGCACATAAAGATCAAGATCATATTCATTTGGTTGATGTTCTTTCCGGTGAAGAGGCCAACAGTTTATTCATCAAAACCTTGTCCGAGTGTAGAGGTAATAGTGCCAGACAATCAAACCAGACCAAGGTGCCAGACAGAGTTTGGCAGATGTGTGGTGGCTTGCCGTTGGCCATAGTTACCATGGCAGGTGTGGTGGCATCCAAGCCACTGATGATCAGAGATGAATGGATTGCCGTTTGCAATTCCTTGTTTCCGGAGCCAGAAAAATGTCATAAACCAGAGGAATTTATGAGGATAATAAACTATTGCTACAACGACTTGCCTAGTGATCTCAAGACTTGCTGTCTGTATCTTAGCATATTTCCTAAGGGCCGTGAAGTTAGCAGGAAGCGGCTGATTCGGAGGTGGATAGCGGAAGGTTTCGTCAGTGAGAAGCAAGGTTTGAGTGTGGAGGATGTTGCTGACACATGCTTCAAACAGCTCATTGAAAGGAAGATGATGAGGCCCGTTGAGCACAACAGCAATGGAAGCGTCAAAAGTTGTCGGGTCCATGACATGGTACTTGAGTACATCATTTCCAAGGCAGCAGAGGAGAATTTCATCACTGTGGTCGGTAGCCACTGGTCCATGTCAACAAGTAGCAAGAAAGTTCGTAGGCTCACTATCCATGGCAGTGACCCCAAACGTGCAAAGAATGTTGACAGTATGAACTTGTCACATGTCCGATCACTGACTGTGTTCGAGAACCTGGACAAACTGCACTTCAAATCATTTAAAACTGGAATAGTGCAAGTGTTAGATCTCGAAGGCTGCAAAGGTTTCAGGGAGAGCGATGCCAACGTTTCAGACATATGCGAAATGATTCTACTTAAGTATCTGAGCCTACGGAAAACAGACATAAAAAATCTTCCCAATAATATTTCCAAGCTCAAGTACCTAGAGACTCTCGATGTTAGGGAGACAGAAGTTAAACAGCTGCCTACGACTGTAGGGCAGTTGGAACGGATAACTAACATCCTTGGTGGTGATAAGAGAAGACAGAGAACCCTGAAACTTCCTAAGGAGCTCAAGGGAACAATGAAATCTTTACGCATATTGTCGGGGGTTGAGATTGTGGAGGGATCAACAGCTGCATCAGACTTCAGTTACTTCACTACTCTGAGGAAGCTGGCAATTTACAGGATCCACCACAATGAAGATATATTCAAAAATTTGCTGTCCTCGATCCAGTACCTCAGTGGTTATTCACTCCAAACTCTTTTAATCGTTGATGAGTCATCTGAATTCTTCAAGACCCTGGAGTCAATGTCGCCATCCCATCTAACTGACCTGAGAGCTCTGGAGCTGTCTGGCAAATTGCTTTACCTTCCAAAGTGGCTCGACACTCTTCAACATCTTGTGAAGTTAACGCTTTCAGCAACAGCCCTATGCACTGATAACTTTTTGGTCATCAGAAAACTGAACTCGTTGTTTTCCCTCACCTTTTCAATCAGCGAAAAGCAAGACCCTGCTTTGGCAGCCATTCTTGAGAAAAATAAGTCAGCTTCAAGAGGAGAAATCGTTGTGCCAGCTGGAGGCTTCAGTAAGCTCAAGCTGCTTCGGATATTTGTTCCTCTTCTTCCATCCCTTACCTTCTCAAAGAATGCTACACCACATCTGGAAAGGATTGAACTGCGTTTTAAAAAGCTGGAAGGATTTCATGGTGTGGATGAACTTGGAAAGCTCCAGGATGTGGTATTAACAGTTGACGGGCATGCAAGTGAGAGGACAAATTCGATACTAGACGGATTGAAGCACAGGCTAGAAGGAAAGTGCAACCTCATTGTCAACAAGTATCACGACTGA

>B97_Zm00018ab296860

ATGGAGTTCGCAACTGGAGCGCTAGGCGCCCTTCTTCCCAAGCTCTCCATGCTGCTTCATGGTGAGTACAACCTGGAGAAGGGCGTCAGGGGGGACATCCAGCGCGTCATGAGCAAGCTCGAGCGGGTTCATGCTGCCCTCCGCCATGTTGGCGAAGTGCCTGTGCCACTGGAGCAGATCATTCGTCCTGGTATAGTCAATATGTGGGCACGCGACGTCGGGGAGCTATCCTACGACATGGAGGACTTCGTCGACACCTTCCTGGTGCGTGTCCAAGGCCCTGAACGCACCAGCAAAAGAAGGTTATTCATTAAGATGATAGATATGGTCATGAATAATCGCCATGATGAGATCGCCCCAGACATCAAGCACTTCGAAAAGCGCGTCCAGGAGATGGATGATCGTCGTCAAAGGTCATTGAAACATTTAGGGTACGGTGTTGGTACTATTGTTCCTACCGTCAAAACCTTGTTTTATCCTCGCATAATTGCTCTGAACTACACCAAGGCCACGGACCTTGTCGGCATCGATGAGGCAAGGGAGGAACTAATCACAAGGTTGACCAAGGAAGATGACACCTCCACTGAACAAAGGCAAGTCTCTATCGTTGGTTTTGGAGGACTTGGCAAGACAGCGCTTGCAAAAGCAGTTTATAACAAACTTAAAGCTAAAGGGGAATTCCATTGTGCGGCCTTTGTGTCGATGTCTCGGTATCCTAAGCTCGTAGAAATCTTCAAGGAATTGCTTTATGAGCTTGACAAGACTGAGTACAAGGACGTTATTAGCACCCCAATGGAAATAGATGAACTTATAAATCTAGTGCACGAATTCCTTAATAAAAAGAGGTACCTTATCGCTGTTGATGACATATGGGATACTGACGCATGGGCAATGATACGATACGCTTTTGCTGAGAATAAGCTAGGAAGCAGAATAATCGTAACTACTCGCAGAATTGATGTTGCTGAGTATGTAGGTGGTTGTTGCTATATGATGAAACCTCTTACTCGAGAGAAATCAAAGATATTATTCTATGGACGAACATTTGGTTCTGAAGGTAAATGCCCTCCTGAACTTTCTCATGCGTCTGAGAAAATATTGAATAAATGCGGAGGCGTGCCATTGGCTATTATTACTATATCTAGCTTGCTGGCTAGTAAGTCAAGAAACATAAAAGAATGGTTCTATCTTGCTGATTCTATTGGTTCCGGAATACTAGAAAGAAGTGCTGAAATGGAGATTATGTGGAAGATACTGCTGCGTAGCTATTCTGATCTACCAGCACGATTAAAGACATGTTTGTTATATCTGAGTATTTTCCCTGAAGATTGTGAGATTGGGATGCATCGGTTAATATGGAGGTGGATAGCTGAAGGTTTTTTCAATGGAGAACTAGCACATGGTGGGCTCTTTAAGATTGGGGAATCTTGTTTCCACGAGCTCATAAGGAGAAGCATGGTGCAGCCAGTAACACTTGAAGGCACGGGTCTTGTATATGCTTGTCGTGTTCATAATATGTTTCATGATTTGATCCTCTCCATGTCACATGAAGAACAATTTGTTTCTGTAGTCAATGAAAAATTTGGTCCTCTTGATGTTCTTTCTCGGCGGTTAGCATTCCAGAACATAAAAAAAAGTCAGTACAGACTTGTGGAACATCCACGGCTGGCACAATCGAGGTCACTTAATGCCATTGGATGTCCTATATACGCGATACCTCCAATTGAAAGCTATAAATCATTGCGTGTACTGGATTTCGAAAATTGTGCAGGTATTGAAGACCATGATCTTGTTCATCTTGGGAAATTGCATCACCTCAAGTTCCTTGGGCTAAGAAACACGTTTATCGGTAAGCTGCCGGAAGGAATAGGGAACCTCAAGTTTCTGCAAACATTGGACCTCGATGGAACTGGTGTGGAAGAATTACCTCAAGCCTTGCATAATCTTACAGAATTGATGTGTCTAATTGCTGACTGGAGAACGAGAGTGCCCAATTGGATTGGTAACCTCACGTCCCTGCAGCACTTGGTGATTTATCCTGGTGGGCATGACGATGAGGATTCTGCGAGCAGGTTTGTTAATGAGCTGGGAAAGCTGAGACAACTAAGGGTGCTCCGTTTTTTGATAAAAGCACAAGATGAATGGCAGCTGAGAGATTTGCTAGAGTCCCTATCGAATCTGCCAGAGATCGAGGCTATACATTTTGATTACTATGGAGTACAGTTAAATAGAGGTGTTCAGTTGGAACCTGAAGGCTATGCCCTCTCTAGACATATTCGTTCCATGGAATTGCGCTGGTTGGAGTTCTCAAGGCTGCCTCTTTGGATTAATCCTGGACATCTTCCTAACCTCTACCACTTATGGCTGATGGTATCTGAGGCGGAAGAACGGGATCTGGAAATCCTTGGGGGGTTTCCAGTGCTTCACTCCCTCCACTTGTTGATTGTGAATACTGAACGTGAAGATGTCATGACTTGTGGCTGTGGTGGATTCAAGAATTTGAAATGCTGCAGTATAACTAAACCGCTGAAATTTGTACATGGAGCTATGCCCAGGCTTGAAGTCCTCGATTTCCATTTCAGTGTGCAACTCCTAACGGATTCAAACCAAGATTTTGATTTTGACTTTGGCTTGGGAAACCTACATTGGCTTCAGCAAGCCATCGTTCAAATCACAGCCCTTGGTGAGGAGGTGGAGTCTGTGGGGAGAGCACAGGTGGCTCTGCGGGATGCAATACGTACCCATCCCAACCGTCCTACCCTTGAAATAAACTTATTTGGGCAAACAATACCTCCAGAGTTACCAAAGCAAGACGACGATGGAGCGAAAATTGTGGAGATATCACCGGCCGAGAGTAGTCGTCAAGCTCAAGAGCGGGAGAAAAGAAGCATCGATGTGGCGACGAAGAAAGCAACACGGGTGCCGTCTTTTTACACAAAGTCATCAATTGATGAGCCAATGGATCAGCTCATAAACATGCTATCTGTGGTTGATGACGAAGCCTACACTAAGAACATAAAGATACTATCTATTGTAAGGTCTGAGGGACTGGGGAAGACTACTCTGGCCCAAAAAGCATTCGAAGAGCTCCATTCGCAATTTGACCGTGGGGCGTTCGTTCTACTAGGCCAGAATCCTGACTTGAGGAGAGTTTTTGCTGACATTCTCCGTGGTCTTGACAAGCAAAGGTACATAGATTTCCCAGTGGCAATATTGGATCTAGTGGACCTGATCTGGCTAGTCCGTAAATCGCTCATAAACAAAAGGTTCTTTATTGTATTTGATGATATATGTGATGTAAAAGCATGGGAAATTATAAAGTGCGCTTTGATTGAAAATAACAACCACAGTGTAGTTCTTACGACAAGTCGCAACACTGGTATTACTGAAATTATTGGTGGCAGCAAGCAATTACAACCTCTATCAGCAACTATCTCTAAAAATCTACTCTGCAAAAGGTTATTTGGATCGGCAGGCAAGTGTCCTTCTGAACTAGTAAATATATGTGACAATCTTGTAGAAGAATGTGGTGGAATACTATCTGTGATCGACGAAACTGTGACATTGCTTGCAAGTATACCACCAACAGTGGAGAACTGGGAGGCAGTGTACGCCAGAAGAATGTTGGATCGGTCTTATCCTGGTTTAACTGACAGTCTAAAGAATTGCTTACTCTATTTTACTATGTTTCGAAGAGGACATGAGATTAGTGGAGAACACTTAATATGTGCATGGATAGCTGAAGGTTTTGTACATGGGCAAGAGGTAGCAGAGACCTACCTTAGTGATCTAGTAAAAAAGAAATTAATCGATGCAGTGGAGGTTGATGCTGGAGGAAAGGTCCTCACGTGCCGCATGTATGACTTGGTGCATGACTTTATCGTCTCAAAATCAATTGAAGAACGATTTGTTTATATTTTAAATGACTCGGAAGGCAGAGATTTGTCAGAAGCAGTTCACGTTCACCAGCGACTATACATCCAGGGACATAATAACAAAGAACTAGACCTGCAAATTCCTTGGCTGCCCCAAGTGAAGTCACTTGTCTCCTGTGGTACTGCGCCATCCATCTTAAAGTTTAAGGGTCTACATGTTATGGATTTAGGGGCCTGTGAATCTTTGCAGGCTAGTCATCTCAAGGGTATAAATAATGTAAGTTCTTTGAGATATCTGGTCATAGGAGGTAAGTGTATCTCTGGCATCCCTAAGGAAATTGCGAAGCTGGAACATTTGCGGACACTAGATTTAAGTGCAAGTGGTCTAAATGAATTGCCAGAATATGTTTTCATGATAAGAAAATTGGAACGCCTAATTGTTAATAGTCAGATGAAGATATCATATGGTATTGCAAAGATGTCTGCTTTACAGGAGCTAGGCGATATCAATGTCACCGACCCAGAGTTGCTGAAAAGTCTCTGTAAGCTAACCAAATTGAGGGTTCTTAGAATTTCCATATGGTCATGGGATGATAGTTTGAAGAACTATTTTAAACAACTGTGTGACAACTTGCGTTCACTGGTTCAGTGCACGGAGAACATCCAGAGTCTCTCCATAATGACATGCTGCTCCCTGGTTTTCATGGATGATTTGGGTGAGAATTGGACCCCTCAATGTCTCCAGAAGCTCGAGGTCGGTTGCAGCGCATTTGACATATTGCCAAGTTGGTTTGGCTCACTTTCTAGTATCTTCACGTTAACAATCGAGGTCTACAAGTTGTCACAGGACATAATTGATACGCTCGGAAGGCTGCCTGGTCTTGGTTCTCTATCCCTGACATCGAAACAAGAACCAAAAGGATACTTTGTGATCGGCTCTGACAGGTTCAGTAAGCTAGAGAGCTTAAAGTTTGTGAGCAATGCAATGGTAGAGATGTTTCCACGTCAACAATCAAATGGCACGGAACAGCTCAAAAGGCTCATGATTGTGTTCCATGCTTCACGTACACAAGATGTGAACAAAGATTTCTGCTTTGGTTTGGAGAACCTGTCTTCCCTAGAGCATGTTCGTGTTGAAATAATTTGTTTCGATGCCAGCCATAACATGGTGAAAAACGCAGAAGCTGCAGTTCGGAAAGCTATATCTGGCACAAGTATCCCAAATCTGGAAATACGAAGACTTCAGGAAAATAGTATGATACAGGACGAAGCGGACCTCTGTGATGCAGTACAAGAGCAGAATAATCAGAAGCACCAGAAAATGAAGAGTTCTATGGAGTCCCAGGATGGGTACCTCACTTCTTTACCGAACCAGGAAAGTGCAGATGTTGTTGGCTCCGATAATATTGTGGAAGTTCATAATAAAGTGATAGAGTTACCACTAGTGAATGAAATGAACTTGCAGACGATTAAGAGGGATCAATACTATAATTTCAGTGAAGATGAGGACTTAATGTTGGTTTCTAGCTACCTTAATGTAAGCAAAGATTCTATTACTGGAAGGGATAAAAAAGAAGGCACATTTTGGGAAAGAGTATGGAAATACTATAACAAGAATAGGACATTCGAGTCCGATCATAGTTGGTTGTCATTGAAACATCGCTGGCTTGCAATTCAGAAGGAAGTGAATATCTTTCAAGGTTACTATGATGCCATAGAAAGGAAAAATCACAGTGGCCAGACAAGTGATGACAAGCATGCTGAAGCAGAAGTAGAATTCCGAGAAAAACAAGGGAAGGCTTTTTCTGTATTCCATGTGTGGATGATTCTAAGGCATGAGCCAAAGTGGGCATTTAGAGAATCAAAGATCAAAGACCAGCATGAAGCAAACAATGCTAATACTGATGCTCCTGCCAACATTTATAGACCACAGGGGAGGAAAGCTGAGAAGGAAAAGGCTCGTGTGAGAAAGCATGGTGGATCTGATGTTGATGGTGATCCGTTCATTGAAGAAGTAAAAAATATGAGGGAAGCACGGGAAGAAACAGAACGAGACCGAAAGACCCATGATGACAAGTTCTATGAGTTGGAAAAGAGTAAGCTTGAATTGGAGCGAGATCGACATGACAAAGAGATAATGCAAACAGACACAAGCACAATGGATGAAGAATCGAAACAATACTTCAAGTTGATGAAACAAGAGATTTTGGCTCGCCGTTTCGGGAGTAGTCAGCCA

>B97_Zm00018ab305250

ATGCCGATTGGAGAGGTTGTGCTATCTGCCTTCATGCAGGCACTCTTTGAGAAAGTGCTTGCTGCTACTATCGGAGAGCTGAAACTCCCTCCAGATGTCACTGAAGAACTGCAAAGCTTATCGAGCATCCTGTCAACAATTCAATTTCATGTCGAAGATGCCGAGGAGCGGCAATTGAAGGATAAGGCTGCACGCAGCTGGCTTGCCAAGCTCAAGGATGTCGCGGATGAGATGGATGACTTGCTTGATGAGTATGCAGCCGAGACTCTGCGATCCAAACTAGAAGGTCCATCCAACCATGACCATCTGAAGAAGGTTAGGAGCTGTTTCTGCTGTTTTTGGTTGAACAAGTGTTTCTTTAATCATAAGATAGCGCAGCACATAAGGAAGATTGAGGGGAAACTCGATAGGCTTATCAAGGAAAGACAAATTATTGGTCCCAACATGAACAGTGGGACCGACAGGCAGGAGATAAAGGAGAGGCCCAAAACAAGTTCGCTGATCGATGACTCAAGTGTGTTTGGAAGAGAAAAAGATAAGGAAACCATTGTAAAGATGTTGCTGGCCCCTAATAATAACTCAGGCCATGCCAACCTTTCTATTATTCCCATAGTGGGCATGGGGGGACTAGGAAAGACGACTCTAACACAGCTCATCTACAATGATGAAAGAGTAAAGGAGCATTTCCAGTTAAGGGTGTGGTTGTGTGTTTCTGAAAATTTTGACGAGATGAAGCTTACCAAGGAAACAATTGAATCAGTTGCTAGTGGATTCTCATCAGCCACAACAAACATGAACCTGCTCCAAGAAGACCTCTCAAAAAAGCTGCAAGGTAAAAGATTTCTTCTAGTCCTTGATGATGTATGGAATGAGGATCCTGAAAAATGGGACAGATATCGTTGTGCTCTACTTAGCGGGGGAAAGGGAAGCAGGATTATAATTACCACGCGAAACAAAAATGTGGGGATACTAATGGGTGGGATGACTCCTTACCATCTAAAGCAGCTATCAAACGATGATTGCTGGCAGTTGTTCAAAAAACATGCATTTGTAGATGGTGACTCCAGTTCACACCCAGAATTAGAAATAATAGGCAAGGACATCGTGAAGAAGTTGAAAGGCCTGCCACTAGCTGCAAAAGCAGTCAGCAGTTTACTATGTACCAGGGATGCAGAGGAAGATTGGAAGAACATACTAAAGAGTGAAATATGGGAATTGCCATCAGACAAGAACAACATATTGCCAGCTCTGAGATTGAGTTACAGCCATTTGCCAGCCACACTGAAGCGATGTTTTGCATTTTGTTCAGTGTTTCCCAAAGATTACGTCTTTGAGAAAACAAGGTTGGTTCAAATATGGATGGCCCTTGGGTTCATTCAGCCTCAAGGAAGGAGAAAGATGGAAGAAATTGGGAGTGGCTATTTTGATGAATTGCAAAGCAGATCCTTCTTCCAACATCACAAAAGTGGATATGTCATGCATGATGCCATGCATGACCTAGCACAGTCTGTCTCAATTAATGAATGCCTTAGATTGGATGAAGGCCTAAGGCTGCATGATCCCCCGCACAGCAACAGCCCTGCAACTGCAAGAAATGCCAGGCATCTATCATTCTCTTGTGACAACAGAAGCTGGACCCAGTTTGAAGCTTTTCTTGGATTTAAGAGAGCTCGCACACTTCTTCTACTAAATGGATACAAATCGATAACAAACTCTATACCCAGTGATCTGTTCATCAAGTTGAAGTACCTTCATGTGCTTGATCTGAACCGACGAGACATTACTGAGCTGCCTGATTCTATTGGTAACTTAAAATTGCTTCGATATTTGAATCTTTCAGGCACTGGAATAGAAATGTTGCCTTCATCAATCGGTAGGCTCTTCAGCCTGCAAACATTGAAGTTGCAAAACTGTCATTCACTAGATTACCTCCCAAAGACCATAACCAATCTCATAAATCTTCGATGGCTAGAAGCAAGGACGGAGTTGATCAATGGCATAGCTGGAATAGGGAACTTGACTTGCCTTCAACAGCTGGAGGAATTTGTTGTCCGTAAGGACAAAGGATACAAGATCAGTGAATTGAAGGCAATCAACGGGATCACAGGACAAATCTGCATTAAGAATCTTGAGAGCGTGGCAAGTGTGGAAGAG

>B97_Zm00018ab313960

ATGGAGACAATCATGGTGAGTGCTGCTTGTGGGGTGATGAATTCCCTGCTGCGCAAGCTTGCCACTCTACTGGAGAAGGAATACATGCTACTGAAAGATGTGAAGCACAACATTACCTTCTTAAGAAATGAGCTTGCTAGCATGAACTTGCTCCTCTTCAAGCTGGCAGACATAGAAGATCTCGATGTGCAAGTAAAGGAGTGGAGGAATAAGGTACGGGAGCTCGCCTATGATATCGAGGATTGCATTGATAACTTTATGGTTAATGATAGGCCCAACAGAAGCCTTATTCGGAAGACTGTAGGTAGGATAAAGAAGTTATGGTTTCAACATGATATTGGCAAACAGATCCAAGAACTCAGAACCCGTGTTGTGGAGGAGAGCGATCGCCGTTATAGATACAAGCTGGACGATTCAACCTTCAGGCCTGAAATGATGGAAATCGACCATCGTCTCAAGGCGCTCTATGTTGACACAAACAAGCTTGAGGGTATTGATAGCCCAGTGGAACAAATCATACAGTGGCTTACAGGAAATGGGAAACACGATCAAGAACTAAACATAATGGCCATAGTGGGCTTCGGAGGTTTAGGCAAAACTACCCTTGCAATGCAGGTATACAATAAATTCAAGGACAATTTTGATTGCACAGCTTTTGTGTCAGTGTCAAGAGGCCCCAACATCAAGAAAGTTTTGATGGACTTACTTAAAGATGTGGGAGCTGCCATTGACACAACAGATGATGAGATGCGTCTCATCAACAAACTCAGAGGACATCTCACAAAAAAGAGGTATTTTGTAGTGATTGATGATTTATGGGATGTCTCAGCATGGAGCTTTATTACATGTGCTTTCCATCAGAATAATCGTGGGAGTCGAATAATCATAACAACGCGCAAAATTGATGTGGCTAAGGCATGTTGCTTGTCCTCTGGTGATCATATTTATGAAATGCAACCCCTTAGCGTTGCTGCCTCAGAAAGATTATTTTTTAAGAGAATTTTTGGTTCTGAAGAAAGATGTCCTTCTCACCTAAAAGAAGCCTCCATCAAAATTCTAAGGAAGTGTGGTGGCCTACCATTGGCTATCATTACTGTATCAAGCTTGCTAGCTAGTAAAGATCTGACAATGGATCAGTGGAACAGAGTGGCAAATTCTATTGGGTCTACACTCGAGAATAATCCAGACATAGAGGTCATGAGAAAGATTCTATCTATCAGTTATTTTGATCTACCACATTATCTGAAAACTTGTCTTCTTTATATAAGTATCTTCCCAGAAGATTACATCATAAATAGAAAGAGTTTGATAGTTAGATGGATTAATGAAGGATTTGTTCAAGAAGAATATGGGAAAAATGCACATGATATAGGTGAAAGTTACTTTAATGAGCTTATCAATAGAAGACTGGTCCAACCATGGTTTATCGATCATGACAGTGGGAGTGTGGTGACTTGTCGAGTTCATGACATGATTCTTGATTTAATCATAACAAAGTCTGTAGAGGAGAACTTTGTTACTTTGTTAGACTCTCGAGAACTTACATCAAGTCCACAGAACAAGATACGAAGATTATCTATCCAATGTGGTGACGGAGAACCACCAGCTTTGGTGTTAGAAGAGTCAACATCGCTATCTCATGTCAGATCAGTGACTATCTTTGGGCATGGGAAGCAACTGCCATCTCTTTCAAACATGAAGGCTCTTCGCGTGCTTGACCTAGAAGGTTGTAAGGGATTAGAAAACCACCACCTGGAAAATATAGAGAGGCTAATCCACCTGAAATACTTGAACCTTAGAGAAACAGAAATCACAGAGCTCCCGAAACAAGTTGTAAAACTCCAATATTTGGATACATTGGATATAAGAAACACTGGGGTGAGTGAGTTACCTTCGGCTATTATTCAACTTCGTCAATTGGCTCGGCTGTTCATTGATCTTGATACCAGATTGCCATATGAAATTGATAAAATGCAAAATTTGGAAGAGCTGACACATGTCAATACGTGTATGTACCATATGAACTTCCTGAAAAAGCTGGCTCAGCTAAGTAAACTTAGAGCGTTGGAGATAAGTTGGGATCGTTACGCTACACAAGTGGATAAAGTTTCTTATCAGAATATTTTGATACATTCGCTTCACATACTGGCCATGCACAACCTTCAGTCTCTCACTATTCACATTGTGGACGAACAGAGCTTCCCTCTCCATACCTGGCATCCTGCTCCTTGTGCACTTCGAAGACTCCACTTCGACATGAAGCTTGGATCAATTTCTAAAGTTCCAACCTGGATGGGATCACTTGTTAATCTCCAAGAACTAAGCTTCCGTATTTGGGGAATGGCTCAAGATGACCTTGATATCCTTGGAGATATACCGGCTCTCCGCTCTCTTTCCTGCTTCATGGGTATTTGCTGCAGAACTGACCGCTTCTGGGTAGATCCACACGATTATTGGCTTAGGATCAGAAAAGGGTTTGAAAGTTTGAACTCTTTCCGCTTCGAATGCGGCAAGTACATGTGCCTCATATTTGAAGCTGGATCGATGCCAAAGCTCGAAATACTTGACCTCGAGGTTTCATCTGTTGAGGACTACGTGATTCAGAAACTTGGTTTTGATTTTGGCATCAATAACCTATCCTTCATCTCCAAAGCTACTCTTAGATTTAGTAATCGGGGATGCAGCCAAGAAGATGAAGTTGCCATCAGGAAAGCAGTCAGCACACATCGCAACAGACCTGCACTGGAAATAATAAGAAAAAAAGCTCGAAGTTAA

>B97_Zm00018ab319080

ATGGACAACAATGGAGAGAAGACGACGACCATCATCATCCACACGCTGCGAGATGCGCTGCTTCACTTTGCAGTCAAGTCCAAGAAGCTCGCGTCGCCACTGCTGGAGCCATTCGGGCGGGCAACCGAGCCGACCACCGTCAACGACGACGAGCTGATGGCGCTCAAGTCCAAACTGCGGCGGATCCGCGCCACCCTCCGCGACGCCGAGAGCCTGTCCGTCACCGATTGCTCCGTCCAGCTGTGGCTGGCCGAGCTCGGCGACCTCGAGAACCGGGCCGAGGATGTGGTCGAGGAGCTGGAGTACGAGTCCCGCCGCTCGGCGCAGCTGGAGGAGCTCAAGCAAGACTTGCTCTACGCCGCCACGACGCGGAAGCAGCGCCGGGAGGTGGCGCTGCTGTTCGCGCCCCCGCCCGCGAGGCGGCTCCGCCGCAAGATCGACGACGTCTGGGCGAGGTACGAAGAGATCGCGTCGGACAGGAAGAGGCTCCGGCTGCGGCCAGGCGACGGCGGCTGCGCGCGCCGGCCCGCGGCCAGCCCGCTCGTGCCAAGCAGCGTGCTTCCCCGTACCGAGCGCCTCCACGGGAGGCATGGCGACGTCGAGAGGGTCGCCGCGCTGGTCCTCGGGGATCCGGACGGCGGGACGAGCTACGCCGTCGTGCCCATCGTCGGGATGGCCGGCGTCGGCAAGACTGCTCTGATGCAGCACGTCTGCGGCATGGAGACGGTGAAGTCATGCTTTGAACTGACGCGCTGGGTTTGGGTCTCCCAGGATTTTGACGTTGTCAGCGTCACCCGCAAGATTGTTGAGGCGATCACCAGATCGCGCCCGGAGTGCGGCGAGCTGAGCACGCTTCATGAGCTCATAGTTGAGCACCTTGCCGGGAAGAGGTGCTTGATCGTTCTTGACGACGTGTGGGATGACAATCCCAGCCACTGGAACAGCCTGACGGCCCCGCTGAGCCACTGCGCGCCAGGGAGTGCGGTTGCCGTGACGACGAGGAGCAACAAGGTTGCCAGGATGGTGAGCACCAAGGTGTATCATCTCAAATGCTTGTCAGATGAAGACTGCTGGCTTGTATGCCAGCGACGGGCACTGCCAAATAGCGGTGCCAACGTCCACAAAGAACTCGTTGAGATCGGTGAGAGGATCGCCAAGAAATGCCACGGCTTGCCATTGGCGGCAGAGGCAGCTGGTAGCGTCCTGAGCACTTCAGCCGTCTGGGAGCACTGGAATGAAGTCCTGAATAACGACTTGTGGGCTGACAATGAGGTGAAGAACCTGGTACTGCCGGTGCTGAAGGTGAGCTACGACCACCTGTCCATGCCGCTGAAGCGCAGCTTCGCGTTTTGTTCATTGTTTCCAAAGGGCTTCGTGTTCGACAAAGATCTGCTAGTCCAGCTGTGGACTGCGCAGGGCTTTGTGGATGCTGAAGGAGACTGCAGCCTTGAAGCGATCGCCAATGGTTACTTCAATGACTTGGTGTCAAGGTGCTTCTTCCACCCTTCCCCATCTCATGCTCTCAGCGAAGGGAAGTTTGTTATGCACGACCTGTATCAAGAGCTTGCTCAGTTTGTTTCAGGCAATGAATGTAGGATGATACAGCTCCCTAATTCGACGAAAATAGATGAGAGCTCTCGGCATTTGTCCTTGGTCGACGAGGAGTCCGATTCGGTCGAAGAAATAAACCTGAGCTGGTTCTGTGGTCATCGTGATCTCCGGACCTTCATGTTCATTGCAAGAACAGAACAGAACCCCGAGGAGATGACCTTCAGAACAAAGATTCCATCTGAGCTGATCACAGGTTTTGAATGCTTAAGGGCTTTAGATTTGAGCAACTCTAATATCATGGAGCTACCGAAATCCATTGGAAGTCTGATACACCTAAGGTTCCTTGGTCTGGACAACACCGCAATTCAGATGCTGCCTGAGTCAATTTGTGCTCTTCTCCACTTGCAGACAATAAAGCTTAACCATTGTTCTTCCCTTACTCAGTTGCCTCAGGGCATCAAGCTCCTATTGAACCTAAGGTGCTTAGAGATTCCACATTCAGGCATAAAGATGCCTTCTGGGATTGGAGAGTTGACTAGGCTGCAGAGACTACCTTTTTTCGCCATCGGGAATGAGCCTGCTGGATGCACCATAGCAGACCTCAACGAACTGGTAAACCTCGAAGGACATCTTCACATCACAGGTTTAAACAACCTGGATGGTGCACAAGCTTCCATCGCCAACCTTTGGAACAAGCCGCGGATTAAAAGTCTTACACTCGAATGGTCCGGAGTTACAAATTTTAGCAAGTCCCTTTGTGATCCGCAAGGAAATGCTGTGAGCTGCATATCAGACAGTCAGCACCCTGCAATCAGTGCAACAGCAGATCAGGTTTTGAATTGCCTCAAGCCACATTCAAATCTGGAGGAGCTTAGCATTAAGGGTTATAACGGATCCTTTTCTCGGTCATGGTTAGGATGGCTGCCCTTGGACAGGTTAGCTTCTATTGAACTGAAAGACTGTCGTAATTGCAAAGAAGTACCGCCCCTTGGCTGCCTACCATCACTGAAACACATTTTGATACAATCACTGCCAAGTGTGAAGCTGATTGGTCCAGAGTTCTTTGGAAATGCTGGAGATACCACTTCTAACATTAGGAGCAGAATCTGTAATGTGTTTCCTGCACTGAAGTCACTAAAGTTCAGCAACATGGAAGCTTGGGAGGAATGGCTCGGTGTCAAGAGTGAGCACTTCCCCAATCTTAAATATTTCAGCATCGTCAGATGCAGCAAACTGAAGCTGTTGCCCAAGTTCACTTCAGAACCAAAGCTGAAAATCCGGTACTGTGACCTGCTGCAAATGCCTTTGTGTCAGAATACGGTGAAGCACATACCAGCTAAAAAGGAAATCTCATATACGTGCATTGCGGAAGGTGACATTTTAGTTCTTGAAGCTTCCTGTTCCTATGGTGCATAG

>B97_Zm00018ab324720

ATGGTCGGCCCCGAGATGCTCGTCGCCGCGACGGTGAACCAGGTCGTTCGGAAGATCAACGAGATCATCGGCGTCGCGCAGGGCGAGGCGAAGCTGTGCTGCAGCTTCAGCGACGACCTCGAGGGCATCAAGGACACCCTGGTATACCTTGAAGGCCTCCTCAAGAACGCGGAGAGCAACTCCTTTGGGAGCGATAGGGCGAACCTGCGGCACTGGCTGGGCCAGATCAAGTCTCTGGCTTACGATATCGAAGACATTGTTGATGGCTACTACTCCTCCAAGGAGCAATATGAGGGAAGCAACTATGCTCAGAAGGGATCTTTACTCTGCTCTTTATCCAATCCCATGCTTTCGAAGGTTAGCATGGTTCATAAGATGAAATCCAAGAGGGAGTTGTTACAGACAAGGCAAAATTTACCGACCCAGTATCATTTCATCTCACATATTAACTCAGTGGTAAATTATGATGAGAAGCAGACAACATCATACAGAAATTGTGACATCAGAATTGTCGGGAGGGGTCCAGACTTGGAACATCTTATGTACATGTTAATGCAGATAAATTTGAAAGAGCTTACCATTATTTCCATAGTTGGGCCTGTGGGCCTTGGGAAGACAACCCTTGCACAACTTATTTTCAATGATGCAAGAGCTGAGACATTCAGATTCAGAATATGGGTTCATGTCTCCATGGGCAATGTCAGCCTCCAAAAAATTGGGAGAGATATAGTTTTGCAAACTACAGAAAAAATTGAGGGAAACATGCAGATGCAGTCAATCAAGAATGTTGTTCAAGACATACTTAATAAGTATAGCTGCTTAATAGTGTTAGATAGCTTGTGGGGAAAAGATGAAGAAGTGAATGAGCTGAAGCAGATGTTGCTTACAGGTAGAAAGACTGAAAGCAAGGTCATAGTGACCACTCACAGCAGTAAAGTAGCAGAGCTGATATCCACTGTGCCACCATACAAACTGTCTTTGTTGTCAGAAGATGATTGTTCAGCTATATTCTGTCAAAGGGCAATTGCAGGTCAGTCTATCAGTGACCCACTTTTCAGGGAATATGGAGAAGAAATTGTTAGGAGATGTGAAGGCATGCCCTTGGTAGCCAATTTTCTTGGATCTGTGGTGAATGCTCAAAGACAGAGGCGTGAGATATGGAAAGCTGCAAGGGATAAAGACATGTGGAAGATAGAGGAAGACTACCCTGAAAACAAAATTGTGCCAATGTTTCCATCTTTCAAGATAGTATACTACAGTATGCCCCATGAGCTAAGGTTATGCTTTGTCTACTGTTCAATCTTTCCTAAAGGATATGTCATAGATAAGAAAAAACTTATTCAGCAATGGATTGCACTTGACATGATTGAATCCAAACATGGAACCTTGCCGCTTGATGTTACTGCTGAGAAATACATTGATGAACTTAAAGATATTTATCTCCTTCAAGTTTTAGAGAGGCATCAGATTGATGCAGAAATATCCAATACTTCTGAAGAAAAGCTCTACATGAATAATTTGGCACATGATCTTGCTAGGTCAGTTGCTGGTGAAGATATCCTTGTGATCTTAGATGCTGAGAATGTACGCTGCAATAGAAATTATGATTACCGTTATGCACAAGTGTCAACTTCTAGCTTACAATCAATAGATAGCAAGGCTTGGCCTTCCAAGGCAAGGTCACTAGTTTTCAAGTCAAGTGCAGAATTGCAGCATGTCAGCGAAGTTCTTTCAGTGAACAAATATTTGCGTGTTTTGGATATCAGTGGGTGTTCTGTGAAGGAGATGCCCGCTCCAATTTTTCAGATGAAACAACTGAGATATCTTGATGCTTCAACATTGTCCATTGCAGATCTGCCTCCCCAAATTAGTGGCTTTCCTAAGCTACAGACACTGGATCTGTCTGATACTGAAGTAACTGAGCTACCAGCCTTCATTGCCAACTTAAAGAGACTGAATTATTTGAATCTCCAAGAAGTCAAGGAAGTTGGATCAAGTAGTGATATCTCAGATACCATTCTAAAGGAGTCTGCAAACAGAGATGTGGCATTAGGAATAAGTTTTCCAGAAATTGATGAAGTTGGTTATCCACATAATAATCTGAAACAAAAATTGACATTATCTTATCATATGGATGGACATAAAAGTGAAGAGCCTAATTTCATTACCAAGAACTCAACTACAGAGACGATGTCTCGATAA

>B97_Zm00018ab366720

ATGCCTGCCCCCGCGCCGTGGCCCGTGTCGCAGGACGTCGCGGCGCTCGCCGATCGCGCCAGCTCCTTCTCCCGTGCTGTCGTCGGGCCCCGCAACGACCTGGGTGCTCTCGCGGCGGCGCTCCTTCGGATCCAGCCCGTAGCCCGCGAGCTCGAGCGCTGGAGGTGGCTCACGCCCGGCGACCCTGAGGTGCCCGACCTGCACGCCTGGCTCTTCGAGCTCAGGGACGCGGTGGCCGACGCCGAGAGCATCCTAGATGAGCTCCACCGTCGGCGCCAGATCGGCCCTCCGCTCTCGGCCTGCGTATACGCCACGTTCCGTGGCCCCGGACGGAAGCTGAGGCGGCTGACGGAGAGGCTCAACCGCGCCCGCGACGATTCGGAGCGTCTGCGACCTGGCAGCGCTGCGGCCATCTACGGTGTGCGGTCGCCGAACAGGGTAACCGGCTCCGTTTTCGCGGAGCGGAAGGTACTCGGGCGTGGGGAGGAGTGCAACTCCATAATCTCCAGGCTCGTCGACGATTGCGAGGAAACATGCCGTCCGGTATCTCCGGTTATTGCAGTGATTGGCCATGGAGGCATCGGCAAAACCACGGTCGCTCAGTGCGTGTACAACGATGCGAGGGTTGAAGCGCGGTTCGATCTGAGAGCCTGGGTCTGCGTCTGGGACAGATCAGATGAGGCCGAGCTCACCAGGGAGATTTTACAGTCCATTGGTTGTGCAGATGATAAACCGTGTGATGACGGTCTTGCCAGTTTAGACAGTTTGCAGGAGAAGTTTGAGAATCTGGTTGCACGTAAGAGGTTCCTCCTGGTACTTGACGATGTTTGGATTGATGAGGGTAAAACCGAGAAGGAGAATAGGAGCATTTGGAACAGAGTGTTGGTGCCACTTAGATCTGCCACAACTGGGAGCAAGGTCTTGTTGACCACTCGGATGAAACTAGTAGCTGAGGTTCTGAATGCGGGTTATTTGGTTTCGCTTGATGGATTAAGAAGCAGTGATTGTTGGTTGCTGTTGAAGGAAGTTGCTTTGGGTGGAGAAACCATGGATTTTCCACCTGAGTTGCAAGAGGTTGCAGGAACTCTTGTTGCAATGGTTAAGGGCTCACCTTTAGCTGCTAAAGCTATTGGGCAAATGGCAAGGAGCACCAGGAGCACACGGAAATGGAGAACCCTGGTGAACACAGAAATTAGTAATGATATCATTATCTCTTCCATCCAACTCAGTTACAAACACTTGCCGGGTCATCTCCAGCGCTGCTTTGCATACTGCAGCATATTTCCATCAACTTGGAGGTTCAGTCGCTCTCAGTTGGTCAATATGTGGATAGCTCTCGGTTTCATTCAATCATCAGCAGAAGGGAAAGGGCTGGAAGAATTAGGGCAAAAATACTTTGATGATCTCCTATCACGATCCTTCTTTGGTACTGCAAATAAAGACCAGCAAACATATTACTTCCTTGATGACCTAATGCACATTTTGGCACAGCACTTCTCTGCTCATGATTGTATGAAAATCAACGAGGGCATCCCTGTTGTGATTCCACCAACAGTTCGTCATCTGTCTGTTTCAACCGATTATTTACCACAGCTGAAGAGCAAATACAGGTTGGGAAGGCTTCAAACATTGTTAGTCCTCAGAAGCTCATCATTATCTTCAGGTCATTTCCCTAGAAAACTTCTGGCCAAATTTAAGAACTTGCGGGTCCTGGATCTGAGTGGATCTGATATTGTAGAATTGCCAGAAAGCATTAACCAGTTGGTACATCTTCATTACCTAGCTATCTGCAGTATAACTAACAAACTTCCCAAGACTATATACATGCTTAAACATCTTGAGGTGTGTGATATACCTATATTCTTATTTCATGATGATCATCCTAGAGGTGTTGGTAAATCTGCTACAGTTAAACGTCTCAAAACATGCTGTGGCAAGCAGGCAAGGTCAGTTAAGTCCTTGATTGATGTGCAAGGACTGTTATAG

>B97_Zm00018ab381670

ATGGCGGAGACGGCGATCACGACGGTGCTGGCCAAGGTGGCGGAGCTGGTGGCGTGGGAGGCGGCTGTGCTGCTGGAGGTGGGCGACGACGTGCGCCTCCTCCGGGACAAGCTCGAGTGGCTCCACACCTTCATCCGCGACGCCGACCGCCGCCGCCGCCGCCGCGACGACGAGTTCGTCGCCGTCTGGGTGCGCCAGACCCGCGACGTCGCCTTCGAGGCCGAGGACGCGCTGGACGACTTCCTCCACCGCGCTGGGCGGAGGAAGGCCGCGCTGGGCTCCCGGTGCGCGCTCGGGTGCTGGTGGCCCGGCGGGTGCGCCGGCCAGGTCGCGCTCCGCCACGACCTCTCGGGGCGTATCCGCCAGATCAGGAAGCGGCTCGATAAGATCTCCGAGAACCGCGCCGACTACAACATCGAGCACACGCCCGCGCCGGCCTGGGCCGCTTGCTCCTCCTCCGCCACCACCCTCGCTGCCTGGGACGATCTAGAGGAGTACACGGTCGGCCTGGACAAGTACAGCGACATGCTTAAGGAGCAGCTCCTCGACGACTCCGTCCCCGCGCGCGCGCTCGTGGCCATCGCCGGCGAGAGCAGCATTGGAAAGACGACACTCGCGCGGAAGGTGTACCAGAGCCTCGAGGTCCGCAACCACTTCGAGATCCGCACGTGGACGGTGCTCCCGCACAAGTGCCGCGCCGCCGACGTTCTCCGCGACATCCACGAGCAGATGACCTCCCAGCTGCGGCGGACCCCGTCCGCGTCCAACTCCAAGCAGGCCGTGGAAGACGCCTGCGATGACAAAGCCTTCGGGCCGGGAAAGGACATCAGCAACCAGCTGTACAGGAGCATGACGGGGAGGCGGTACCTCGTGGTCATCGACGGCAGCGTTGCCGTCACGGACTGGAACAGCCTACGAGCTTCCCTCCCCGACGAGGGCAACGGCAGCAGGGTGCTTCTGATCACGGACTTGGAAGGCCTGGAGGTGGTCGGCCACGCTCAGGCTGGCCACACGTACGACCCGATCGAGCTGACCCGGCTGAGCCCGGAGAGCACGTACGAGGTGTTCCGACGCCGGGTGTTCGGCGCCCGCGGCGACTGCCCCGGCCGGTACAAGTCGAGGTACTACCAAGACGTGTTCCGGATCACGCGCGGCCTGCCGCTGTCGATCGTCGTCCTCGCCGGTATCCTGCGGTCCAAGGAGCTGCCGGCAGAGTGGGACGAGGTGATGGCGCAGCTGGCCCCGCCGGCGCGCGAGCAGCAAAGGGGCGGCAGCGGCGGCAGCAGCAACAGCTGGCCCCGGATCATGTCGCGGGCCTTCGACGACCTGCCGCACCACCTCAAGTCGTGCTTCCTCTACCTGGCGGCGATGCGGGAGAGCACCCCGGTGGACGCGCAGCGCCTCGTGCGCCTGTGGGTGGCCGAGGGGTTCGTGCGCCCGCGCCGCGGGAGCACCATGGAGGAGGTCGGGCAGGGGTACCTCAAGGAGCTCATCTCCCGGTGCATGGTGCAGCTGGTGGACAAGGACGACTTCGGCGCCGTGCTGACGGTGGTGGTGCACGACCGGCTGCACGCATTCGCGCAGGACGAGGCACAGGAGGCCAGCTTCATCGAGAGCCACGACAGCACAGACGTGCTCGCGCCGGCCACCGTGCGCCGCCTCGCCGTCCTCAACTCCACCACGGACAGGTACGTCCAGCTCAGCAACGCGCTCCCCAAGCTGCGCTCCATCATCTGCGACTTGGTGGAGGGTCGCCGAGTCAGGTCCAGCAACTTCATCCGCACCAGCGACCTGAGCTTCCTCCATGCTTCCAAGTTCCTCCGTGTCATCGACATCCAGGGACTCGAGCTCAATAGGCTCCCAAACGAGATCGGCTCCATGATCCACTTGAGGTACCTGGGTCTCCGCTGCGGCCACCTGGAGAAGCTCCCGAGCACCGTTGGCAACCTTGTCAACCTGCAGTCGCTCATCCTCGGCGGCCGCCACGTTCTGGAGGTGCCCGCCGCGTTCTGGAGGATCGCCACGCTGCGGCACGTCGTCGCGCGGTTCGCTCTGCCGAGCAGGGCCCTGGGCAACCTGCACAGCCTCCAGACGCTACATGGCGTGCAGCCCCGCGGGTGGGGCGGCGACTACAACCCGCTGGGGAAGGCCGCCAACCTCCGGTCGCTGGAGCTGGGCGAGCTGACCTCCGAGCACGCCGATGCGCTCGAGGCCGCGTTGGAGAACCTCGACCTCCTGGAGCACCTGGCGCTGCGGGGCGACCCGCTGCCGTCGAGCGTGTTCAGCGTCCCGAGCCTCCGCCGGCTGCAGAGCCTGAGGCTGATGGGCGCCATGGATGAGCCAGAGGGGCCCAGCTGCGCGGAGGACGTCCGGTACATCCGGCCGAACCTGACCAGGCTATCGATGTGGAATACCGAGGTGGGGCAGAAGTTCGTGGACATGCTGGCCGAGCTGCCGAGCCTGGCCGAGCTGACGATGATGTACGACTCATACGACGGCGATCGGCTGGCGTTCGTGGAGACTGGGTTCCCGAGCCTGCAGAAGCTAAAGCTCGGTCTACCGGAGCTGGAGGAGTGGACGGTGGCCCCGGGGTCCATGCCGGGGCTCGGCACGCTGACGCTGTGCCGGTGCGCCAGGATGCAGATGCTCCCGGAGGCGCTCGCCGGGATGAGGGAGCTGGAGGAGGTGGTGCTGTACAGCATGCCCGACATCGTGAGCAGGATCAAGGAGGACGAAGGCCAGGATTACCACAAAGTCAAGCACGTACCCGTCATCCAGACTATATACTGA

>B97_Zm00018ab396690

ATGGCCCAACAGGCAGTTTTTGCGGTGCTGGAGCGGGGTGGTAGCATTGCAGTTGATGAGGCCGTATACTTGTTGGGCGTCTCTGAAAAGCTGGAATCAGCCAAGAAGCAACTCCTACTCATGCAAGCCTTTCTCATGGACCTGGATGAAAAGATGCTGAAGGGTAACTTCATGGCTAGGCATCTAGCTTCCGAGGTTCGGGAGATCGCATATGAGGTGGAGGACATCATTGACACCGCCAACATCTTGATGAGGAGAAACGGCCCCAAGACATCAGTCAGAGGGGCCATGTCCAAATATGCTTGCTTCCCCATATACCTCACTCGTCTCCACAAGCTGGGATCAAGGATAGATTCAACTGAAGAAAGGATGAAAAAACTTTTCGGCGACTTTGAGAAATTCAACATCGCTGCTAATGCAATTGCAGAAGAACCACGGCGTTACATCACCGAGGATGATGACATTCGGCATAGGCGATTGGTTCATCCCAACTCGGGTGATCAAGTTGGTGTCATTGGGTTTGATGAGCAGATTAAACAGATCGAGTATGACTTGCTAGACACAAAGAACAGGCACCTAACCGTTGTTTCCATTGTAGGTCCCGGTGGGGCAGGCAAATCAACAATGGCTAAGAAGGTGTATAGTTTGCCTGCAGTGAAGGGACACTTTAAAGTTCATTGTTGGTTAACTGTGTCCCAACGAGCTGTTGCAACTCATGATTTTTTGAAAGAGGTGGTTAAGATGGTTGTGCCTTCTCACCTCATGAAAGTTATGGTTCTGCGTGTGATGGGGGATGTCAAGGTCAAGAAAGTCGACAACGCTAGAAAGATGATGACCGAGAAGGAGGAGAAGAAAATTTGGGAAGACCAGAAAGCTAAAGAGCTCGAGGAAGCTAAAGAGCTTGATAAGCTGGAGGAGCATGAGGTGAAAAAGCTGCTTCACGAGTTTGCACTGAGTCAAAGATATTTGATAGTGTTGGATGATATATGGAGCAAAGATGCCTGGGATGCTATCAAGCATGCCTTCCCTAATCAGAAGAATGGCAGCAGAATCATTCTGACCACACGCAATGTGGATGTTGCAAAGCTTCCCGGTGCAAGGAAGAAAATCTACAGGCCAAAGCTCCTGAATGAAGATGAAAGCACCCAGTTGCTTCTTACCACGGCCCTCCCAGAGTACATCTTGGATGGCGGGCAAAACTTGGACGAGTTGAAGGAGCTGGGCAAAGAGCTTGCAATAAAATGTGGTGGCCTGCCTCTTGCTTTGATTGTTTTGGGGGGATACCTGTCAAGGAATCTTGATGTTGGCGAGTGGAAGAGGTTGTTAACAAACAGTATGGATTGGCATGATTTGATCACCTCCGATAGGGTCATTGGTGCCATACTGGATCTTAGTTACTATGACATGCCGAGTCATCTGAGATCATGCTTCATGTACACCACGGCCTTCCCTGAAGACTCTCCTATTGATGTGCGAGTTCTGGCAATGCTATGGATTGCTGAGGGTTTCATCCCGCTGGTCCGAGGCCAGACCCGCGAAAAAGTAGCCTTAAAGTATGTGGCGGAGCTAGTGCAGCGGTGCATGATCCAAGCGGAGGGATGGACGAACTCTGGGATGATCAAGGTGGTCAAGGTGCATGACATCCTGCGCGAGTGGGGGTTTGGACGAGCTCAGAGAGAAGGGTTTATGAAAGATTGCCATTCTGCGGAGGATATCGAGGTGGCCTACTCGGGGGAGGAGATGATGAAAGCTTACCGAGTGGTGCTCCACAGCTCACTGTCACTGGAAAGGGGAGTTGGAACCACCACGAGAAAGCTTCGCACTCTATTAGACTTCAACAACCACACATCAGTACAGGTGCCAAAATCGTTCCAGGGTTTACGCGTGCTTCACCTGAACTGTTCTGGCGAGGTTTCTCTGCCAAAGGATATTCACCAGATGAGGTATTTGAGATATCTTGGCTTGGGAGGCAACTGCTCCTATGATCTCCCTTCCAACATTGGAGGCCTCTTAAGCCTCGAAACACTGTACTGCACAGCAAGTATAGACCACATCCCAGCATCATTGTGGCGGAACAGGACACTGAGGCAAGTGCATATCCCCTATGCTAGAAGCTTATCATCACCACAGATCGGTTCACAGTCATCAAAGGTTCTTGTTATACTAGTAGATTGTGGGAGCAGTACCCATATGGACGATGCTAAGCGGATTGTGGAGAAAACTAGAAGGCAAGTGCTGAGGAACAAGAACCTGGACCTCTCGTTCTGCTTGGGGGTGGAGTATGGGAAATATGGCATGGAAGTCATAGGGAGATGCAATACGGGGGTCCAGTTCCCCATCGACCTCCTCAATTTTGATGAGACGCATGATTACTGGGAACTGAAGATTTGCTGCGCCAATCTACTCAGCAATGATCACAAGATCCTGGAGCTTGGGAGGATAAAAAACCTCAAGGTGCTAGAGATAGGCGAGCAGTCATATACTGGCAAGGTGATGGTTTTCACGTCAGGCAGCTTCATGACACTTGAACGACTGGTGCTCTACGATCTTGCAGTGGAGAAATGGAAAATTGAGTGTGGATCCATGATATGTCTCAGGGTGTTGACGCTTTGCAAGTGCCCCAAACTGGTCCACCTGCCTGAAGAGTTGTTAAGGCTTCCGAAACTACGCAGTCTGGCTCTTATCGCAATGCCTCTGGGTTGCTACCAGAAAGGTGAGGTTCCTCAGGGGGTGAAGATTTCAGAGTCAGATGATGAGAAAGTTTTCCAGCATCTTCCTATTTGGCGGTGGCTCCACCTGTCTGCCCCGCCCGCATTTGATGAATACTAG

>B97_Zm00018ab424670

ATGGAGGTGGCTCTGGGAACGGCCAAGTATCTCCTTGGCCATGTTCAGAACAATATCTCCGACGACTGGATGAATTCCTACGTGTCCAGCGCCGAGCTCGGCACCAACCTTAACATGATCAAAGAGAAGATGCGGTACGCTAGAGTGCTGCTGGACGTGGCCAAGAGGAGGGACGACGTCGTCGCCGGGGACCCCAACCGGCTGGAGCAGCTCGAGACTCTCGGCAAGAAGGCCGACGAGGCTGAGGATGCCGTGGACGAGCTTCACTACTTCATGATCCAGGACAAACACGACGGGACTCGAGACGCCGCACCGGAGTTGGGCGGTGGCCTCGCAGCCCAAGCTCACCATGCCCGCCATGCTGCTCGCCACACTGCTGGTAACTGGCTCTCATGCTTCTCTGGCTGCTGTCCCCGAGGAAAGTTGTCATTCAATCGAGTGGCTATGTCCAACAAAATCAGGCTCCTCATAGAGGAGCTGCAATCCAACTCTACTCATGTCTCTCACTTGCTCAAGATAGTGTCAGACACAACCAACTTTAGCTCCTCCACTGAAAGGCCCCCGACAAGCTCTATAATCACACAGGACGAGTTGTTTGGGAGGGATGCCATCTTTCAGAAAACTATAGAGGATATTATCATAGCTAAAGATAGTGGCAAAACCTTGTCCGTTCTTCCTATATTTGGCCTAGGGGGCATTGGGAAGACTACCTTCACCCAGCACCTATACAATCACACAGAGTTTGAAAAACATTTCACTGTTAGGGTCTGGATATGTGTATCGACTAATTTTGATGTGCTTAGGCTCACCAAAGAGATCCTGAGCTGCCTACCCGCAACTGAAAATGCAGGAGATAAAATAGCAAATGACACAACCAACTTTGACCTGCTTCAGAAATCCATCGCAGAGAGGTTGAAATCCAAAAGGTTTCTGATTGTCTTGGATGACATATGGGAATGCAGCAATAATGAGGAGTGGGAGAAACTAGTAGCTCCATTCAAAAAGAATGATACCACTGGCAACGTGATTCTTGTCACAACCCGATTCCAGAAAATTGCAGATTTGGTGAAAAAAGAAACTAACCCAGTTGACCTTGGCGGTTTGGATCCTGATGAGTTCTGGAAATTCTTCCAGATATGTGCATTTGGTAGTATTCAAAATGTTGAGCATGGTGATCAAGAGTTAATTGGTATTGCAAGACAAATAGCAGATAAGCTAAAATGCTCCCCACTTGCAGCCAAAACAGTTGGTCGGCTATTGATTAATAAACCCCTTCAGGAACATTGGAAGATAATTCTTGAGAACAAACAGTGGCTAGAGGAAAAAAAGGGCGATGATATTATCCCAGCCTTGCAAATTAGCTATGACTACCTTCCCTTCCATCTGAAAAAATGTTTTTCATATTTCTCCCTTTTCCCAGATGATTATAAATTTGATAAGTCTGAGATTATTCGTTTATGGGATTCAATAGGAATCATAGGTTCTAGTATACAGCAAAAGAAAATAGAGGACATAGGATCGAATTATTTTGATGAACTATTAGATAGTGGTTTTCTTATAAAAGGGGACAATGATTTTTATGTAATGCATGATTTACTCCTTGATCTTTCACGGACTGTTTCAAAACAAGAGTGTGCCTATATCGATTGTTCTAGTTTTGAGGCAAATAACATCCCACAGTCTATCCGTTACCTATCCATTTCCATGCATGATCATTGTGCTCAGAATTTCGAGGAAGAAATGGGTAAACTGAAAGAAAAGATAGACATTAAAAATTTGCGGGGTTTGATGATATTTGGAAGTTACATTAGGTTACATCTGGTCAATATTTTAAGGGACACATTTAAGGAAATAAGATGTCTTCGTGTTCTATCTATATTTATATACTCCCATAGTTCCTTGCCAAACAACTTTTCAGAGCTTATCCATCTTCGCTACTTAAAACTCAGTTCACCTTATTACTCAGAAATGTCTTTGCCAAACACAGTCTCAAGGTTTTATCACCTGAAATTTCTAGATCTTAAACAATGGGGAACTGATCGTTCTTTGCCTAAGGACATTAGCCGCCTTGAAAATCTACGCCATTTCATTGCTTCAAAAGAGTTTCATACCAATGTTCCTGAGGTGGGGAAAATGAAATCTTTACAAGAACTGAAAGAATTCCATGTTAAGAAAGAGAGTGTTGGATTTGAGCTAGGAGAGTTGGGGAAACTAGCAGAGCTTGGAGGGGAGCTCAATATACTTGGGCTTGAAATGGTGAGAACCGAGCAAGAAGCAAAAGATGCCAAACTGATGTCAAAGAGGAATTTAGTTGAGTTGAGATTAGTTTGGAACACGAAACAAGAGTCCACTGTGGATGATATCCTAGATAGTATGCAGCCGCACTCTAATGTTAGGAGACTTTTCATTGTAAATCATGGTGGTACCATCGGTCCTAGTTGGTTGTGCAGCAACAGCAACATATACATGAAAAACTTGGAGACTCTACATCTAGAGAGCGTATCGTGGGCTAACCTTCCACCTATTGGGCAGTTCAATCACTTAAGAAAGCTAAGGCTGAGTAAAATTGTTGGGATATCACAGATTGGACCTGGCTTCTTCGGTAGCACGACAGAAAAAAGTGTCTCACACTTGAAGGCAGTTGAGTTTAATGATATGCCAGAGCTTGTCGAGTGGGTTGGGGGAGCTAACTGGAATCTGTTCTCAGGAATTGAAAGAATCAAGTGTACTAATTGTCCTAGGCTGACAGGGCTGCTGGTCTCAGATTGGTCTATTTCTTCTATAGAAGACAACACTGTATGGTTCCCTAATCTTCATGACCTTTGCATTTATGAATGCCCGAAGTTGTGCCTTCCACCCTTGCCTCACACTTCAAAGGTATCCTGTATTTATATGGGAGACTTTTCTTATGATGATCGTACCGTGTTGCACATTAATAATCCCTCTAGATTAGCCTTCCAAAATCTAGGTGACCTGGAGAAATTGAGAGCTACGGATGCACTACTCTTGTCATTTATGGATCTTAAAAAGCTACATTCCTTGAGACATATAAGGGTCAATAGATGCGAGGAAACATTTTTGAGAGGACTGGATAATGGTGTTGTGCTACCCACAGTCCAATCTCTCGAACTTGGACAATTTACACCTACCAAAAATTCTATGTCAAATTTGTTCAAATGTTTCCCAGCACTTTCTTCTTTGGATGTGATGGCATCACCGTCAGATGAGGACCACGAGGAAGTGGTACTGCATTTTCCACCCTCTAGCTCTCTGAGAGATGTCACCTTCAAATGGTGTAAGAATCTGATCCTACCCATGGAGGAAGGAGCTGGATTCTGTGGCCTCTTGTCGCTCGAGTCAGTGACCATACGCAAATGTGACAAGCTGTTCTCTCAGTGGTCCATCGGAGGACTAGCAACTCAGACTCAGAGCATCATCAACCCTCTCCCACCCTACCTGAGGAAACTCTCCCTTTATTATTATAATATGGAAACTCTGCCTCAGGAAGCTCTGCTCGCGAATTTGACATCTCTTGAGACACTCGAACTACATAATTGTCTTGGCTGTGAGCAAAGCACCGAGCCGATGGCTCTGCCCGCGAATCTAGCATCTCTTACCAGTCTAGTGTTAGGTAATTGCAGAAATATCACAATGGATGGATTTGATCCTCGCATCACATTCAGCCTCAAGGATCTATGGGTGTACAATGAGAGAAATGATGGGACTGATCCGTATTCTGTAGCAGCAGACCTACTTGTAGCGGCGGTGAGGACCAAAACAATGCCCGACGTTTCCTTCAAACTGGTGACACTTTATGTGGACAACATCTCGGGAGTGCTTGTTGCTCCCATCTGCAGGCTGCTCTCCGCTACCCTCGAGATGTTAACCTTCATTAATGATTGGCGGACAGAGAACTTCACGAAAGAGCAGGACGAGACGCTTCAGCTCCTCACGTCTCTCAAATCCCTTGATTTCTATCACTGCAGGGCTCTGCAGTCGCTCCCCCAAGGACTGCATCGCCTTCCTTCTCTCCATGTAATAAATATCCGTGGGCCTCAAAACATCATATCCCTGCCTAAGGAGGGCCTCCCCGATTCACTACGACAACTATATATAGCAGAGTGTTGTACCGAGATTTATGAGGCATGCCAGCAATTGAAGGGAACAAGGCCAGATATACGTGTAGTTGCCTCCAAAGCTCGTGTGCAAAATTGA

>B97_Zm00018ab424760

ATGGAGTCGGCTCTAGGAATGGCCCACTGGCTTCTCGGCCAAGTCCTCAACAAGCTCTCTGACGACTTGGTGAAAGCCTACGTGTCCAGCACCGAGCTCGGCTCCAACCTTGAGAAGATCGAAAGGGAGATGCTGTTCACTAGAGGGCTGCTGGACAGGGCCCTCAAGAGCGACGTCACCGGGGACCTCAACCTGCAAAGGCTACTCGAGAGGCTCGGCAAGAAGGCCGACGAGGCTGAGGACACCCTGGACGAGCTCCAATACTTCATGATCCAGGACAAGCACGACGGCACTCGAGAGGCCACTCTAGAACTGGGCGGTAGCCTCGCCGCTCAAGCTCAGCATGTCCGCCATGCTGCTCGCCACACTGCCGGTAACTGGCTCTCATGCTTTTCTTCCTGCTGTCCACGCGACAATGATGCTGCTGCCGATGCCAATGCCATGTGTGGTGACGGTGGCCATGGTGGAAAGTTGCCATTCGATCGAGTGGCCACTCCGGAACTCCGCGATGGCCTCGGCGCCAAAGCTCAGCATGCCCACCACGCTGCACGCCACACCTCTGGTAACTGGCTCTCATGCTTCTCTTGCTGCCAGCCGCGAGACGATGTTGCTGCTGCCGATGCCATTTTTGGTGACCGTGGCCATGTTGGAAAGTTGCCATTCGATCGAGTTGCCCTGTCCGACAAAATCAAGCTCCTCATAGAGGAAATGCACTCTTATTGCACTCCTATCTCTAAATTGCTCGATAAAACGCCAATGAGTAACCTTCACATGCCTGCCTCCATTAAAAGGCCGGACACAAGCTCTCAAATCACACAGGACAAGTTGTTTGGGAGGGATGCCATCCTTCAGAAAACTATAGATGATATTATCATAGCCAAAGATAGTGGCAAAACCTTGTCCGTTCTTCCAATAGTTGGACCAGGGGGTATTGGGAAGACTACCTTCGCCCAGCACCTATACAATCACACAAGGATTAAAGGACATTTCACTGTTAGGGTCTGGATATGTGTGTCGACTAATTTTGATGTCCTTAGACTCACCAAAGAGATCCTGAGCTGCCTACCTGCAACTGAAAATGCAGGAGATAAAACAGCAAATGAAACTACCAACTTGGACCTACTCCAGAAATTCATCGAACAGAGGTTGAAATCCAAAAGGTTTCTGATTGTCTTGGATGACATATGGGAATGTCGTAGTAGTGACGAGTGGGAAAAACTATTAGCTCCACTCAAAAAGGACGAGACCATTGGCAACATGATTCTTGTCACAACTCGGTTCCCAAAAATTGTACATATGGTGACAAAAGAAACTAATCCAGTTGACCTTCGCGGTTTGGATCCTGATAAGTTCTGGAAATTCTTCCAGATATGTGCATTTGGTAGAGTCCAAGATGAGCATGGTGACCAAGAGTTAATTGGCATTGCAAGACAAATATCAGATAAGCTGAAATGCTCCCCACTTGCAGCCAAAACAGTTGGTCGCCTATTGATTAAAAAACCCCTTCAGGAACATTGGATGAAAATTCTTGAAAACAAACAGTGGCTAGAAGAAAAACATGACAATGATGTTATCCCAGCCTTGCAAATTAGCTATGACTACCTCCCCTTCCATCTGAAAAAATGTTTTTCATATTTTGCTCTTTTTCCTGAGGATTATAACTTTGATAAGTCTCAGATTATTCGTTTTTGGGATTCGATAGGAATCATAGATTCCACTAGACAACACAAGAAAATAGAAGACATAGGATCCGATTATTTTGATGAACTATTAGATAGTGGTTTTCTTATACAAGGGGATCATAATCTTTATAAAATGCATGATTTGCTCCATGATCTTTCACGGATTGTTTCATTAGAAGATTGTGCCTACATCAATTGTTCTAGTTTCAAGGCAAAAAACACCCCACGGTCTATTCGCTACCTATCCATTTTCATGCATGATACTAATTTTCAAAATTTTGAGGAAGAGATGGGTAAACTGAAGGAAAGGGTAGACATTAAAAATTTGCGGACTTTGATGATTTTTGGAGAATACAGTAGGTTACACCTGATCAATATTTTAAGGGACACGTTTAAGGAAATAAAACGTCTTCGTGTTCTATCTATATTCATGAACTCCCATAGTTCCTTGCCAAACAACTTTTCAGAGCTTATCCATCTTCGCTACTTAAAACTCAGTTCACCGTATTACCTAAAAATGTCTTTGCCAAGCACAGTCTCAAGGTTTTATCACCTGAAATTTCTAGATCTTGAACAATGGGAAAGTGGTTATTCTTTGCCTAAGGACATTAGCCTCCTTGAAAATCTACGCCATTTCATTGGTTCAAAACATTTTCATACCAATGTTCCTGAGGTGGGGAAAATGATCTTCTTACAAGAACTGAAAGAATTCCATGTTAAGAAAGAGAGTGTTGGATTTGAGCTAGAAGAGTTGGGGAAACTAGCAGAGCTTGGAGGGGAGCTCAATATACTTGGTCTTGAAAAGGTGAGAACCGAGCAAGAAGCAAAAGATGCCAAACTGATGTCAAAGAGGAATTTGGTTAAGTTGGGATTAGTTTGGAACACGAAACAAGAGTCCACTGTGGATGATATCCTAGATAGTATGCAGCCGCACTCTAATGTTAGGAGACTTTTCATTGTAAATCATGGTGGTACAATCGGTCCTAGTTGGTTGTGCAGCAACAACAACATATACATGAAAAACTTGGAGACTCTACATCTAGAGAGCGTATCGTGGGCTAACCTTCCACCAATTGGGCAGTTCTATCACTTAAGAGAGCTAAGGCTGAGTAAAATTGTTGGGATATCACAGATTGGACCTGGCTTCTTCGGTAGCACGACAGAAAAAAGTGTCTCACACTTGAAGGCAGTTGAGTTTAATGAGCTTGTCGAGTGGGTTGAGGAAGCTAACTGGAATCTGTTCTCAGGAATTGAAAGAATCCGGTGTACTAATTGTCCTAGGCTGACAGGGTTGCTGGTCTCAGATTGGTCTATTTCTTCTATAGAAGACAACACTGTATGGTTCCCTAATCTTCATGAGCTTTACATTGTTGAATGCCCGAAATTGTGCCTTCCTCCCTTGCCTCACACTTCAAAGGTATCCCGTATTCATATGGGAGAATTTTCTTATTATGGTGATCGTACCGAATTGTACATTAATAACCCCTCTAGATTAGCCTTCCAAAATCTGGGTGACCTGGAGACATTGATAGCTAGCGATGCACTACTCTTGTCATTTATGGATCTTAAAAAGCTACATTCCTTGAGACATATAAGGGTCAATAGATGCGAGGAAACATTTTTGAGAAGACTGGATGATGGTGTTGTGCTACCCACAGTCCAATCTCTCCAACTTGGACAATTTACACCTACCAAAAATTCTTTGTCAAATTTGTTCAAATGTTTCCCAGCACTTTCTTCTTTGCATGTGATGGCATCACTATGGGATGAGGACCACGAGGAAGTGGTACTGCATTTTCCACCCTCTAGCTCTCTGAGAGATGTCACCTTCACAGGGTGTAAGAATCTGATCCTACCCATGGAGGAAGGAGCTGGATTCTGTGGCCTCTCGTCGCTCGAGTCAGTGACCATACACAAATGTGATAAGTTATTCTCTCGATGGTCCATCGGAGGACGAGCAACTCAGACTCAGAGCATCATCAACCCTCTCCCACCCTACCTGAGGAAACTCTCCCTTTATTATATGGAAACTCTGCCTCAGGAAGCTCTGCTCGCGAATTTGACATCTCTTGAGGAACTCACACTACGTAATCGTCTTGGCTGTGAGCAAAGCACCGAGCTAATGGCTCTGCCCGCGAATCTAACATCTCTTACCACGCTAGATTTATATAATTGCAGAAATATCACAATGGATGGATTTGATCCTCACGTCACATTCAGCCTCAAGAGTCTATGGGTGTACAATGAGAGAAGAGATGGGACTGATCCGTATTCTGTAGCAGCAGATCTACTTGTAGCGGTGGTGAGGACCAAAACAATGCCCGACGTTTCCTTCAAACTGGTGACCCTTGAAGTGGACAGCATCTTGGGAGTGCTTGTTGCTCCCATCTGCAGGCTGCTCTCCGCTACCCTTGAGATGTTAACCTTCAGTCATGATTGGCGGACAGAGAACTTCACAAAAAAGCAGGACGAGGCGCTTCAGCTCCTCACGTCTCTCCAATTCCTTCAGTTTTATAACTGCAGGGCTCTCCAGTCTCTCCCCCAAGGACTGCATCGCCTTCCTTCTCTCCAGCATATACGTATCAGTGGCCGTCAAAACATTAAATCACTGCCTAAGGAGGGCCTCCCCGATTCACTACGACAACTACATATAACTAAGTGTTGTGCCGAGATTTATGAGGCATGCCAGCAATTGAAGGGAACAAGGCCAGATATAGAAGTAGTTGCCTCCAAAGCTCATGTGCAAAATTGA

>B97_Zm00018ab424780

ATGCTTCTCTGGCTGCTGTCCCCGACCCCGAGACACGATGCTGCTGCTACCGATGCCATGTCTGGTGACGGTGGCCATGTTGGAAAGTTGTCATTCAATCGAGTGGCTATGTCCAACAAAATCAAGCTCCTCATAGAGGAGCTGCAATCCAACTCTACTCCTGTCTCTGACTTGCTCAAGATAGTGTCAGACACTAGTAACCCTCAAGACAGTAGCTCCTCCACTAAAAGGCCCCCGACAAGCTCTCAAATCACACAGGACAAGTTGTTTGGGAGGCATGCCATCTTTCAAAAAACTATAGAGGATATTATCATAGCCAAAGATAGTGGCAAAACCTTGTCCGTTCTTCCTATATTTGGCCCAGGGGGCATTGGGAAGACTACCTTTACCCAGCACCTATACAATCACACAGAGTTTGAAAAACATTTCACTGTTAGGGTCTGGATATGTGTATCGACTAATTTTGATGTGCTTAGGCTTACCAAAGAGATCCTGAGCTGCCTACTCGCAACTGAAAATGCAGGAGATAAAACAGCAAATGAAACAACCAACTTTGACCTGCTTCAGAAATCCATCGCAGAGAGGTTGAAATCCAAAAGGTTTCTGATTGTCTTGGATGACATATGGGAATGCAGCAATAATGAGGAGTGGGAGAAACTAGTAGCTCCATTCAAAAAGAATGATACCACTGGCAACATGATTCTTGTCACAACCCGCTTCCCGAAAATTGTAGATTTGGTGAAAAAAGAAACTAACCCAGTTGACCTTCGCGGTTTGGATCCTGATGAGTTCTGGAAATTCTTCCAGATATGTGCATTTGGTAGAATTCAAGATGTTGAGCATGGTGATCAAGAGTTAATTGGTATTGCAAGACAAATAGCAGATAAGCTAAAATGCTCCCCACTTGCAGCCAAAACAGTTGGTCGGCTATTGATTAAGAAACCCCTTCAGGAACATTGGATGAAAATTCTTGAGAACAAACAGTGGCTAGAGGAAAAAAATGGCGATGATATTATCCCAGCCTTGCAAATTAGCTATGACTACCTTCCCTTCCATCTGAAAAAATGTTTTTCATATTTCTCCCTTTTCCCAGATGATTATAAATTTGATAAGTCTGAGATTATTCGCTTTTGGGATTCAATAGGAATCATAGGTTCTAGTATACAGCAAAAGAAAATCGAGGACATAGGATCGGATTATTTGGATGAACTATTAGATAGTGGTTTTCTTATAAAAGGGGACAATGATTTTTATGTAATGCATGATTTACTCCTTGATCTTTCACGGACTGTTTCATTACAAGAGTATGCCTATATCGATTGTTCTAGTTTTGAGGCAAATAAAATCCCACAGTCTATCCGTTACCTGTCCATTTCCATGCATGATCATTGTGCTCAAAATTTTGAGGAAGAGATGGGTAAACTGAAGGAAAGGATAGACATTAAAAATTTGCGAACTTTGATGATTTTTGGAGAATATAGTAGGTTACACCTGATCAATATTTTAAGGGACACATTTAAGGATGTAAAACGTCTTCGTGTTCTATCTATATTCATGAACTCCCATAGTTCCTTGCCAAACAACTTTTCAGAGCTTCTTCATCTTCGCTACTTAAAACTTTTGTCACCTCGTTACTCAGAAATGTCTTTGCCAAACACAGTCTCAAGGTTTTATCACCTGAAATTTCTAGATCTTAAACAATGGGGAAGTGATCGTTCTTTGCCTAAGGACATTAGCCGCCTTGAAAATCTACGCCATTTCATTGCTTCAAAAGAGTTTCATACCAATGTTCCTGAGGTGGGGAAAATGAAATTTTTACAAGAACTGAAAGAATTCCATGTTAAGAAAGAGAGTGTTGGATTTGAGCTAGGAGAGTTGGGGAAACTAGCAGAGCTTGGAGGGGAGCTCAATATACTTGGTCTTGAAATGGTGAGAACCGAGCAAGAAGCAAAAGATGCCAAACTGATGTCAAAGAGGAATTTAGTTGAGTTGGGATTAGTTTGGAACACGAAACAAGAGTCCACTGTGGATGATATCCTAGATAGTATCCAGCCGCACTCTAATGTTAGGAGACTTTTCATTGTAAATCATGGTGGTACAATCGGTCCTAGTTGGTTGTGCAGCAACAGCAACATATACATGAAAAACTTGGAGACTCTACATCTAGAGAGCGTATCCTGGGCTAACCTTCCACCTATTGGGCAGTTCAATCACTTAAGAAAGCTAAGGCTGAGTAAAATTGTTGGGATATCACAGATTGGACCTGGCTTCTTCGGTAGCACGACAGAAAAAAGTGTCTCACACTTGAAGGAAGTTGAGTTTAATGATATGCCAGAGCTTGTCGAGTGGGTTGGGGGAGCTAACTGGAATCTGTTCTCAGGAATTGAAAGAATCAAGTGTACTAATTGTCCAAGGCTGACAGGGTTGCTGGTATCAGATTGGTCTATTTCTTCTATAGAAGACAACACTGTATGGTTCCCTAATCTTCATGACCTTTACATTTATGAATGCCCGAAATTGTGCCTTCCACCCTTGCCTCACACTTCAAAGGTATCCCGTATTCGGATGGAAGACTTGTCTTATTATGATGATCGTACCACGTTGAGCACTAATAACCCCTCTAGATTAGCCTTCCAAAATCTGGGTGACCTGGAGAAATTGATAGCTAAGGATGCACTACTCTTGTCATTTATGGATCTTAAAAATCTACATTCCTTGAGACATATAAGGGTCAATAGATGCGAGGAAACATTTTTGAGAAGACTGGATGATGGTGTTGTGCTACCCACAGTCCAATCTCTCCAACTTGAACAATTTACACCTACCAAAAATTCTATGTCAAATTTGTTCAAATGTTTCCCAGCACTTTCTTCTTTGGACGTGATGGCATCACCATCGGATGAGGACCACGAGGAAGTGGTACTGCATTTTCCACCCTCTAGCTCGCTGAGAGATGTCAACTTCAGAGGGTGTAAGAATCTGATCCTACCCATGGAGGAAGGAGCTGGATTCTGTGGCCTCTCTTCGCTCGAGTCAGTGACCATACACAAATGTGACAAGTTATTCTCTCGATGGTCCATCGGAGGACGAGCAACTCAGACTCAGAGCATCATCAACCCTCTCCCACCCTACCTGAGGGAACTCTCCCTTTCTTATATGGAAACTCTGCCTCAGGAAGCTCTGCTCGCGAATTTGACATCTCTTGAGAGACTCACACTACATAATTGTCTTGGCTGTGAGCAAAGCACCGAGCCGATGACATCTCTTACCAGTCTAGAGTTATTTAATTGCAGAAATATCACAATGGATGGATTCGATCCTCACATCACATTCAGCCTCAAGGATCTATGGGTGTACAATGAGAGGAATGATGGGACTGATCCGTATTCTGTAGCAGCAGATCTACTTGTAGCGGCGGCTGCTCTCCGCTACCCTCGGCGCGTTAAAGTTCAGAAATGA

>B97_Zm00018ab424800

ATGGAGGTGGCTCTGGGAACGGCCAAGTATCTCCTTGGCCATGTTCTCAATAATCTCCCCGACGACTGGATGAAATCCTACGTGTCCAGCGCCGAGCTCGGCACCAACCTTAACATGATCAAAGAGAAGATGCGGTACGCTAGAGCGCTGCTGGACGTGGCCAAGGGGAGGGACGACGTCGTCGCCGGGAACCCCAACCTGCTGGAGCAGCTCGAGATTCTCGGCAAGAAGGCCGACGAGGCTGAGGATGCTGTGGACGAGCTTCACTACTTCATGATCCAGGACAAACACGACGGGACTCGAGACGCCGCACCGGAGTTGGGCGGTGGCCTCGCAGCCCAAGCTCACCATGCCCGCCATGCTGCTCGCCACACTGCTGGTAACTGGCTCTCATGCTTCTCTGGCTGCTGTCCCCGACCCCGAGACGATGCTGCTGCTACCGATGCCATGTCTGGTGACGGTGGCCATGTTGGAAAGTTGTCATTTAATCGAGTGGCTATGTCCAACAAAATCAAGCTCCTCATAGAGGAGCTGCAATCCAACTCTACTCCTGTCTCTGACTTGCTCAAGATAGTGTCAGACACTAGTAACCCTCAAGACAGTAGCTCCTCCACTAAAAGGTCCCCGACAAGCTCTCAAATCACACAAGACAAGTTGTTTGGGAGGGACGCCATCTTTCAGAAAACTATAGAGGATATTATCATAGCCAAAGATAGTGGCAAAACCTTGTCCGTTCTTCCTATATTTGGCCTAGGGGGCATTGGGAAGACTACCTTCACCCAGCACCTATACAATCACACAGAGTTTGAAAAACATTTCACTGTTAGGGTCTGGATATGTGTATCGACTAATTTTGATGTGCTTAGGCTCACCAAAGAGATCCTGAGCTGCCTACCCGCAACTGAAAATGCAGGAGATAAAATAGCAAATGACACAACCAACTTTGACCTGCTTCAGAAATCCATCGCAGAGAGGTTGAAATCCAAAAGGTTTCTGATTGTCTTGGATGACATATGGGAATGCAGCAATAATGAGGAGTGGGAGAAACTAGTAGCTCCATTCAAAAAGAATGATACCACTGGCAACATGATTCTTGTCACAACCCGATTCCAGAAAATTGCAGATTTGGTGAAAAAAGAAACTAACCCAGTTGACCTTCACGGTTTGGATCCTGATGAGTTCTGGAAATTCTTCCAGATATGTGCATTTGGTAGTATTCAAGATGTTGAGCATGGTGATCAAGAGTTAATTGGTATTGCAAGACAAATAGCAGATAAGCTAAAATGCTCCCCACTTGCAGCCAAAACAGTTGGTCGGCTATTGATTAAGAAACCCCTTCAGGAACATTGGATGAAAATTCTTGAGAACAAACAGTGGCTAGAGGAAAAACATGGCAATGATATTATCCCAGCCTTGCAAATTAGCTATGACTACCTTCCCTTCCATCTGAAAAAATGTTTTTCATCTTTCGCCCTTTTCCCTGAGGATTCTAAATTTTATAAGTCTGAGATTATTCGTTTATGGGATTCAATAGGAATCATAGGTTCTAGTATACAGCAAAAGAAAATAGAGGACATAGGATCAGATTATTTTGATGAACTATTAGATAGTGGTTTTCTTATAAAAGGGGGCAATGATTTTTATGTGATGCATGATTTAATCCTTGATCTTTCACGGACTGTTTCAAAACAAGATTGTGCCTATATCGATTGTTCTAGTTTTGAGGCAAATAACATCCCACGGTCTATCCGTTACCTATCCATTTCCATGCAAGATCATTGTGCTCAGAATTTCGAGGAAGAAATGGGTAAACTGAAAGAAAAGATAGACATTAAAAATTTGCGGAGTTTGATGATATTTGGAAAATACATTAGGTTACATCTGCTCAATATTTTAAGGGACACATTTAAGGAAATAAGACGTCTTCGTGTTCTATCTATATTCATATACTCCCATAGTTCCTTGCCAAACAACTTTTCAGAGCTTCTTCATCTCCGCTACTTAAAACTTTTGTCACCTTATTACTCAGAAATGTCATTGCCAAACACGGTCTCAAGGTTTTATCACCTGAAATTTCTAGATCTTGAACAATGGGGAAGTGATCGTTCTTTGCCTAAGGACATTAGCCGCCTTGAAAATCTATGCCATTTTGTTGTTTCGGAAAAGATTGATTCCAATGTTCCTGAGGTGGGGAAAATGATCTTCTTACAAGAATTGAAAGAATTCCATGTTAATAAAGAGAGTGTTGGATTTGAGCTACAAGAATTGGGTAAACTAGATGAGCTTGGAGGGAAGCTCAATATATACGGGCTTGAAAATGTGAGAACCAAGAAAGAAGCTAAAGAGGCCAAGCTGATGTCAAAGAGGAATTTAGTTGAGTTGGGATTAATTTGGAACATGAAACAAGAGTCCACCGAAGATGATATCCTAGATAGTATCCAACCACACTCTAATGTTAGGAGTCTTTTTATTGTAAATCATGGTGGTACCCTCGGTCCTAGTTGGTTGTGCAGCAGCGACACCATATACATGAAAAACTTGGAGACTCTACATCTAGAGAGCGTATCGTGGGCTAACCTTCCACCTATTGGGCAGTTCTATCACTTAAGAGAGCTAAGGCTGAGTAAAATTGTTGGCATATCACAGATTGGACCTGGCTTCTTCGATAGCACGACAGAAAAAAGTGTCTCACACTTGAAGGCAGTTGAGTTTAATGATATGCCAGAGCTTGTCGAGTGGGTTGGGGGAGCTAACTGGAATCTGTTCTCAGGAATTGAAAGAATCAAGTGTACTAATTGTCCTAGGCTGACAGGGTTGCTGGTTTCAGATTGGTCTATTTCTTCTATAGAAGACAGCACTGTATGGTTCCCTAATCTTCATGACCTTTACATTTATGAATGCCCGAAGTTGTGCCTTCCACCCTTGCCTCACACTTCAAAGGTATCCCGTATTCATATGGGAGACTTTTCTTATGAAGGTCGTACCATGTTGAAAATTAATAACCCCTCTAGATTTGCCTTCGAAAATCTGGGTGACCTAGAGACATTGGTAGCTTGGGATGCACTACCCTTGTCCTTTATGGATCTTAAAAAGCTACATTCCTTGAGACGTATAAATGTCACTAGATGTGAGGAAACATTTTTGAGAGGACTGGATGATGGTGTTGTGCTACCCACAGTCCAATCTCTCTACCTTGGACGATTTACACCTACCAAAAATTATTTGTCAAAATTATTCAAATGTTTTCCAGCACTTTCTTCTTTGCATGTGATGGCATCACCATCAGATGAGGACAACGAGGAAGTGGTACTGCATTTTCCACCCTCTAGCTCGCTGAGAGATGTCAACTTCACAGGGTGTAAGAATCTGATCCTACCCATGGAGGAAGGAGCTGGATTCTGTGGCCTCTCGTCGCTCGAGTCAGTGACCATACACAAATGTGACAGGTTATTCTCTCGATGGTCCATCGGAGGACGAGCAACTCAGACTCAGAGCATCATCAACCCTCTCCCACCCTACCTGAGGAAACTCTTCCTTTCTTATATGGAAACTCTGCCTCAGGAAGCTCTGCTCATGAATTTGACATCTCTTGAGAAACTCACACTATGTAATTGTCTTGGCTGTGAGCAAAGCACCGAGCGAATGGCTGTACTCGCGAATCTAACATCTCTTACCACGCTATATTTATATAATTGCAGAAATATCACAATGGATGGATTCAATCCTCACATCACATCCAGCCTCAAGTATCTATGCGTGTACAATGAGAGAAATGATGGAACTGATCCGTATTCTGTAGCAGCAGATCTACTTGTAGCGGTGGTGAGGACCAAAACAATGCCCGACGTTTCCTTCAAACTGGTGGGACTTTATGTGGACAACATCTCGGGAGTGCTTGTTGCTCCCATCTGCAAGCTGCTCTCCGCTACCCTCGAGACGTTAAACTTCAGTAATGATTGGCGGACAGAGAACTTCACGAAAGAGCAGGACGAGGCGCTTCAGCTCCTCACGTCTCTCCAATTCCTTGAGTTTTATAACTGCAGGGCTCTGCAGTCCCTCCCCCAAGGACTGCACCGCCTTCCTTCTCTCCACGGAATAACTATCTATGGGCCTCAAAACATTAGATCACTGCCTAAGGAGGGCCTCCCCGATTCACTACGAATACTACGTATAACTAACTGTTGTGCTGAGATTTATGAGGCATGCCAGCAATTGAAGGGAACAAGGCCAGATATAAATGTACTTGCCTCCAAAGCTCGTGTGCAAAATTGA

>B97_Zm00018ab424810

ATGGAGGTGGCTCTGGGAACGGCCAAGTCTCTCCTTGGCCATGTTCTCAACAATATCTCCGACGACTGGATGAAATCCTACGTGTCCAGCGCCGAGCTCGGCACCAACCTTAACATGATCAAAGAGAAGATGCGGTACGCTAGAGCGCTGCTGGACGTGGCCAAGGGGAGGGATGACGTCGTCGCCGGGAACCCCAACCTGCTGGAGCAGCTCGAGACTCTCGGCAAGAAGGCCGACGAGGCTGAGGATGCTGTGGACGAGCTTCACTACTTCATGATCCAGGACAAACACGACGGGACTCGAGACGCCGCACCGGAGTTGGGCGATGGCCTCGCAGCCCAAGCTCACCATGCCCGCCATGCTGCTCGCCACACTGCTGGTAACTGGCTCTCATGCTTCTCTGGCTGCTGTCCCCGAGGCGATGCTGCTGCTACCGATGCCATGTCTGGTGACGGTGGCCATGTTGGAAAGTTGTCATTCAATCGAGTGGCTATGTCCAACAAAATCAAGCTCCTCATAGAGGAGCTGCAATCCAACTCTACTCCTGTCTCTGACTTGCTCAAGATAGTGTCAGACACAACCAACTTTAGCTCCTCCACTAAAAGGCCCCCGACAAGCTCTATAATCACACAGGACAAGTTGTTTGGGAGGGATGCCATCTTTCAGAAAACTATAGAGGATATTATCATAGCCAAAGATAGTGGCAAAACCTTGTCCGTTCTTCCTATATTTGGCCTAGGGGGCATTGGGAAGACTACCTTCACCCAGCACCTATACAATCACACAGAGTTTGAAAAACATTTCACTGTTAGGGTCTGGATATGTGTATCGACTAATTTTGATGTGCTTAGTCTCACCAAAGAGATCCTGAGCTGCCTACCCGCAACTGAAAATGCAGGAGATAAAACAGCAAATGACACAACCAACTTTGACCTGCTTCAGAAATCCATCGCAGAGAGGTTGAAATCCAAAAGGTTTCTGATTGTCTTGGATGACATATGGGAATGCAGCAATAATGAGGAGTGGGAGAAACTAGTAGCTCCATTCAAAAAGAATGATACCACTGGCAACATGATTCTTGTCACAACCCGATTCCAGAAAATTGCAGATTTGGTGAAAAAAGAAACTAGCCCAGTTGACCTTCGCGGTTTGGATCCTGATGAGTTCTGGAAATTCTTCCAGATATGTGCATTTGGTAGAATTCAAGATGTTGAGCATGGTGATCAAGAGTTAATTGGTATTGCAAGACAAATAGCAGATAAGCTAAAATGCTCCCCACTTGCAGCCAAAACAGTTGGTCGGCTATTGATTAAGAAACCCCTTCAGGAACATTGGATGAAAATTCTTGAGAACAAACAGTGGCTAGAGGAAAAACATGACAATGATATTATCCCAGCCTTGCAAATTAGCTATGACTACCTTCCCTTCCATCTGAAAAAATGTTTTTCATATTTCTCCCTTTTCCCAGATGATTATAAATTTGATAAGTCTGAGATTATTCGTTTATGGGATTCAATAGGAATCATAGGTTCTAGTATACAGCAAAAGAAAATAGAGGACATAGGATCGGATTATTTTGATGAACTATTAGATAGTGGTTTTCTTATAAAAGGGAGCAATGATTTTTATGTAATGCATGATTTAATCCTTGATCTTTCACGGACTGTTTCAAAACAAGAGTGTGCTTATATCGATTGTTCTAGTTTTGAGGCAAATAACATCCCACGGTCTATCCGTTACCTGTCCATTTCCATGCATGATCATTGTGCTCAGAATTTCGAGGAAGAAATGGGTAAACTGAAAGAAAAGATAGACATTAAAAATTTGCGGAGTTTGATGATATTTGGAAAATACATTAGGTTACATCTGGTCAATATTTTAAGGGACACATTTAAGGAAATAAGACGTCTTCGTGTTCTATCTATATTCATATACTCCCATAGTTTCTTGCCAAACAACTTTTCAGAGCTTCTTCATCTCCGCTACTTGAAACTTTTGTCACCTCATTACTCAGAAATGTCTTTGCCAAACACAGTCTCAAGGTTTTATCACCTAAAATTTCTAGATCTTGAACAATGGGAAAGTGGTTATTCTTTGCCTAAGGACATTAGCCGCCTTGAAAATCTACGCCATTTGATTGCTTCAAAAAGGTTTCATACCAATGTTCCTGAGGTGGGGAAAATGAATTTTTTACAAGAACTGAAAGAATTCCATGTTAAGAAAGAGAGTGTTGGATTTGAGCTAGGAGAGTTGGGGAAACTAGCAGAGCTTGGAGGGGAGCTCAATATACTTGGGCTTCAAAAGGTGAGAACCGAGCAAGAAGCAAAAGAAGCCAAACTGATGTCAAAGAGGAATTTAGTTGAGTTGGGATTAGTTTGGAACACGAAACAAGAGTCCACTGTGGATGATATCCTAGATAGTATGCAGCCGCACTCTAATGTTAGGAGACTTTTCATTGTAAATCATGGTGGTACCATCGGTCCTAGTTGGTTGTGCAGCAACAGCAACATATACATGAAAAACTTGGAGACTCTACATCTAGAGAGCGTATCATGGGCTAACCTTCCACCTATTGGGCAGTTCTATCACTTAAGAGAGCTAAGGCTGAGTAAAATTGTTGGGATATCACAGATTGGACCTGGCTTCTTCGGTAGCACGACTGAAAAAAGTGTCTCACTCTTGAAGGCAGTTGAGTTTAATGATATGCCAGAGCTTGTCGAGTGGGTTGGGGGAGCTAACTGGAATCTGTTCTCAGGAATTGAAAGAATCCGGTGTACTAATTGTCCTAGGCTGACAGGGTTGCTGGTCTCAGATTGGTCTATTTCTTCTATAGAAGACAACACTGTATGGTTCCCTAATCTTCATGACCTTTGCATTTATGAATGCCCGAAGTTGTGCCTTCCACCCTTGCCTCACACTTCAAAGGTATCCCGTATTTATATGGGAGACTTTTCTTATGATGATCGTACCGTGTTGCACATTAATAATCCCTCTAGATTAGCCTTCCAAAATCTAGGTGACCTGGAGAAATTGAGAGCTACGGATGCACTACCCTTGTCATTTATGGATCTTAAAAAGCTACATTCCTTGAGACGTATAAAGGTCGATAGATGTGAGGAAACATTTTTGAGAGGACTGGATGATGGTGTTGTGCTACCCACAGTCCAATCTCTCCAACTTGGACAATTTACACCTACCAAAAATTCTTTCTCAAATTTGTTCAAATGTTTCCCAGCACTTTCTTCTTTGCATGTGATGGCATCACCAATAGTTCAGGACCACGAGGAAGTGGTACTGCATTTTCCACCCTCTAGCTCGCTGAGAGATGTCACCTTCAATGGGTGTAAGAATCTGATCCTACCTATGGAGGAAGGAGCTGGATTCTGTGGCCTCTCGTCGCTCGAGTCAGTGACCATACACAAATGTGACAAGTTATTCTCTCGATGGTCCATCGGAGGACGAGCAACTCAGACTCAGAGCATCATCAACCCTCTCCCCCCCTGCCTAAGGAAACTCTCCCTTTCTTATATGGAAACTCTGCCTCAGGAAGCTCTGCTCGCGAATCTAACATCTCTTACCACGCTAGATTTATATAATTGCAGAAATATCACAATGGATGGAATCGATCCTCACATCACATTCAGCCTCAAGTATCTATGGGTGTACAATGAGAGAAATAATGGGACTGATCCGTATTCTGTAGCAGCAGATCTACTTGTAGCGGTGGTGAGGACCAGAACAATGCCCGACATTTCCTTCAAACTGGTGAGCCTTGATGTGGACAGCATCTCGGGAGTGCTTGTTGTTCCCATCTGCAGGCTGCTCTCCGCTACCCTCGAGATGTTAAAGTTCAGAAATGATTGGCAGACAGAGAACTTCACGAAAGAGCAGGACGAGGCGCTTCAGCTCCTCACGTCTCTCCTATCACTTGAGTTTTATAACTGCAGGGCTCTCCAGTCCCTCCCCCAAGGACTGCATCGCCTTCCTTCTCTCCAGGGAATAACTATCCGTGGGCCTCAAAACATCATATCCCTGCCTAAGGAGGGCCTCCCCGATTCACTACGACTACTAGAAATAACTAATTGTTGTGCCGAGATTTATGAGGCATGCCAGCGATTGAAGGGAACAAGGCCAGATATACGAGTATTTGCCTCCAAAGCTAATGTGCAAAATTGA

>B97_Zm00018ab424950

ATGGCGGACTTGGCGCTAGTTGGCTTAAGGTGGGCAGCATCGCCGATTGTCAAGGAGCTTCTTACTAAAGCTTCAGCTTACCTCAGTGTGGACATGGTGCGTGAAATCGAACGACTACAAGACACTGTCCTGCCACAGTTCGAGTTGGTGATTCAAGCGGCCCAGAAGAGCCCCCATAGGGGCAAGCTAGAATCCTGGCTTCGGCGTCTCAAAGAAGCCTTCTATGATGCCGAGGACCTGCTGGACGAGCATGAGTACAACGTCCTTAAGGCCAAGGCCAAGAGCGGAAAAGGTCCCCTGCTCCGAGAGGATGAAAGCTCCTCCACTGCAACCACTGTCATGAAGCCTTTTCATTCTGCTATGAACAGGGCACGCAACTTGCTCCCTGGGAACAGAAGGCTAATTAGCAAGATGAACGAGCTCAAAGCTATTCTGACAGAAGCCAAGCAGCTTCGAGATCTTCTTGGCTTACCACATGGCAATACTACCGAGTGCCCAGCTGCAGCACCTACCGATGTTCCCACAACTACATCACTTCCCACTTCCAAGGTTTTCGGTCGCGACAGGGATCGTGATCGCATAGTAAAATTTCTTCTCGGCAAGACAACAACTGCTGAGGCAAGCTCAACTAAGTACTCCGGTTTGGCCATTGTTGGATTGGGAGGAATGGGGAAGTCTACCTTAGCACAATATGTCTATAATGACAAGAGGATTGAAGAATGCTTTGATGTCAGGATGTGGATCTGTATCTCGCGCAAACTTGATGTGCATCGTCACACAAGGGAGATCATTGAGTCCGCAAAAAAGGGGGAGTGCCCACGTGTCGATAATCTCGATACTCTCCAGTGCAAACTACGAGACATACTACAACAGTCAAAAAAATTCCTGCTTGTCTTGGATGATGTTTGGTTTGAAAAATCTGATAGTGAGACAGAGTGGGACCTACTCCTTGCTCCATTAGTCTCTAAACAGCCGGGAAGCAGAGTTTTGGTGACTTCTCGACGTGAAATGCTTCCAGCCGCTGTTTGCTGTGAACGAGTTGTTCGTTTGGAAAACATGGATGATACTGAGTTCTTGGCTCTCTTTAAACAACATGCTTTCTCTGGAGCAAAAATCAAAGACCAGCTGTTACGCACGAATCTGGAACATACTGCAGCGGAGCTTGCTAAAAGGCTTGGACAATGTCCTTTGGCAGCGAAAGTTCTTGGTTCTCAGCTGTGTAGGAAAAAGGATATTGATGAATGGGAAGCTGCTCTAGAGCTTGGAGATTTAAGTGATCCCTTGACATCTCTGTTGTGGAGCTACGAGAAGTTAGATCCATGTCTGCAAAGGTGCTTCTTGTATTGCAGCTTGTTTCCAAAAGGTCATAGATATAGACGTGGTGAGTTGGTTCAACTTTGGGTGGCAGAAGGATTTGTTTGTTCATGCAATTTGAGTAGGAGAACATTGGAAGAGGTTGGGATGGATTACTTCACTGAGATGGTCTCTGGATCTTTCTTCCAATTGGTTCCTGAAAGACTTTATTCATACTATACCATGCACGATATCCTTCATGATTTGGCAGAGTCACTCTCTAGGGAAGACTGCTTCAGATTGGAAGATGATAATGTTACAAAAATACCAGGCACTGTTCGATATCTATCTGTTCATGTTGAGAGTATGCAAAAGCATAAGAAAATTATCTGCAAGCTACTTCATTTACGCACCATTATCTGCATCAATCCACTAATGGATGGTGCAAGTGATCTTTTTGATCAAATGCTACACAACCAAAGAAAATTGCGTGTATTGTATTTGTCATTTTACACCAGCAGCAAGTTGCCGGAATCTATTGGTGAGCTAAAGCACCTCCGGTATTTGAACCTTGTCAGGACATTAATTTCTCAAATGCCTAGATCATTATGTACTCTCTACCACTTACAATTACTTTGGTTGAACTGCATGGTAGAGAGATTGCCTGACAAACTTTGCAATTTAAGTAAGCTGCGACATCTAGGAGCATACCCATATTACTTTCATGGTTTCGTGGATGAAAGGCCTAATTACCAAGTTCCGAACATAGGTAAGCTAACTTCACTACAGCACATTTATGTCTTTTCTGTGCAAAAGAAGCAAGGGTATGAGTTGCGACAGCTGAGGGACTTGAATGAGCTTGGTGGCAGTTTAAGAGTAAAAAATCTTGAGAATGTCATTGGAAAGGATGAAGCCTTAGAGTCGAAGCTATTTCTGAAACGTCGCCTTAAAGAGTTGGCACTTGAGTGGAGTTCCGAGAATGCAACAGATATCCTGCACTTGGATATTCTAGAAGGTCTAAGACCACCACCCCAACTGAGTAAGCTCACAATCGAAGGTTACAAATCTGACACATACCCTGGGTGGTTACTTGAGCGATCCTATTTTAAGAATTTGGAATGTTTTGAGCTTAATAATTGCAGTTTATTAGAAGGTCTGCCACCTGATGCCAGGCTGCTTCGGCGTTGCTCTAGGCTACATATAAAAAACGTTCCAAATTTGAAGGAACTATCTTATCTTCCAGCAGGCCTTACAGAGTTGTCAATTCACAAGTGTCCACTGCTTATGTTTATCACCAACAATGAGCTAGGACAACATGGCTTGGAGGAAAATGTAATGAAGATAGACGACCTGACATCAAAACTTGCATTGATGTGGGAGGTGGATTCAGGTTCGTGTATTAGGAGAGCGCTATCAGAGGACTGTTCATCTCTGAAGCAGATTATTATGACACTAATGGATGATGATATATCAAAGCACCTCCAAATTATTGAAAGTGGTCTAGAGGAAGGAGGAGAAAAGGTATGGATGAAAGAAAACATCATCAAAGCATGGCTCTTTTGCCATGAGCAGAGGATAAGTTTCATTTATGGGAGGGCCATGGAGATGCCACTAGTTCTACCGTCAGGACTCTGTAGTCTTTCTCTTTGTTCATGCAGTATTACAAATGAAGCTTTAGCTATTTGCCTTGGTGGCCTTACTTCACTGAGGAATTTAGAATTGAAATATAATATGGAATTAACTACACTTCCAACAAAAGGAGTGTTTGAGCATTTAGCAAAGCTTGACAGGTTGTTTATAAGTGGTTGTTTGTGTCTCAGATCACTGGGTGGTCTACGTGCTGCTCCATCTCTTTCCTTTATTAACTGTTGGGATTGTCCTTCTTTAGAGCTAGCACGTGGAGCAGAACTAGTTCCGTCGAACCTTGATAGATACCTCAACATCCGTGGCTGCATTCTTTCAGCTGATTCATTCATTAACGGCCTGCTGGACCTTAAATACCTTTCCATTGATAGCTGCAGAAGCTCCCCATCCTTGTCGATTGGCCACATGACCTCGCTTCAATCATTACATCTAGTTGGTCTCCCCGATCTTTACCTTGTTGAAGGCCTGTCTTGCCTGCGCCTTAAGTTCTTACAACTAGTAGACGTTCCAAACCTCACTGCAAAGTGCATCTCACAGTTTCGTGTCCGGGTATCGCTCACCGTTAGTAGCTCCTTATTGCTCAACCACATGCTCATGGCTGAAGGGTTTAGGGTCCCACTGAATCTTTATCTTTTAGATTGCAAGGAGCCGTCGATTTCGTTTGAAGATCCTGCAAATCTGTCATCTGTCAAGTGCCTGCAGTTTTTGCGTTGTGAAACGGAGTCCCTGCCAGGAAATCTAAAATCTCTCTCAAGTCTGGAGAGTCTTCATATAGGGCGTTGCCCCAACATAGCATCTTTACCAGATCTGCCATCCTCCCTCCAGCGCATAACTATATGGGATTGTCCCGTCTTGAAGAAGAACTGCCAAGAACCTGATGGAGAAAGCTGGCCAAAGATTTCGCACGTTCGTTGGAAGAGCTTTCTACAAATACCGCACTGGCTTCCTTAG

>B97_Zm00018ab427290

ATGGAGGCGACGGCGCTGAGCTTGGGCAAGGCAGTGCTGGGCGGAGCGCTCAGCTATGCCAAGTCCAAGGCCGCAGAGGAGGTCGCACTGCAGCTCGGCGTCGAGGACGACGTGACCTTCATCACGGACGAGCTGCAGATGATGCAGTCCTTCCTGATGACAGCCGATGAGGAGCGAAGCCAGACCAAGGTGGTCACGACCTGGGTGACGCAGGTCCGCGACCTGGCCTACAACGTGGAGGACAGTCTCATGGATTTCGGCATCCACGTTGGGAAGAAGCCGATTTGGGGGTGCATCCCCCGTAGCTCGTGCGATCGACGGCGCATAGCCAAGGAGGTGAAGAAGCTAAGGTCCAAGGTCGAGGATGTGAGCAACAGGAACCTGCGCTACCGCCTCATCAAGGATGGCTCAAGATCCAAGCCTGCCGGTGCGGCAGAGCAAACTACTGCCTCTGATGGTGTAGAGCGTCAAAAACCAAAGGCAGACCTGCTGCAGCTGGTCACTAGCAAGGAGGTCGACCTGAGGGTCGTGGCTGTGTGTGGAACAACCGGTGATCGTGGGAAGACGGCCGCCATCCATGAGGTCTATGATCACCCAAGTGTGGCATCAAAGTATGGTGGACTCAGGGCATGGGTTAGGCTGATCCACCCTTTCAATCCAGAAGAGTTTCTTCGTAGCATGGTACGGCAATTCTTGGAGAATTTCTATGATAAGCCTCAAGGTGGAGAAGAAGCAACAAATGTTGGGGCTACTGTCCTTCTGAAGATGGACAAGATGAACCAAAGTGATTTAGTGCACACGTTTACTACACAGTTGAGTAGCAATAGCTATCTAATTGTAATAGATGACCTATGCACAGTAGAAGAGTGGCAATGCATCAGAAGGTTCTTCCCTGACAACAAGAAAGAGAGCAGAATCATAGTGTCCACTCAGCAAGTTGAGGTTGCAGGCTTATGTACAGAGCAACCATACCAAATGTCCGAGTTTAAGCAGCTCCCGTATGACCAAACACTTTATCTGTTCCATAGGAAGGTTATTAGTGAGATACAGGAAGAATATCAACCACCAAAGAGTACAGGAAATAGTTTCTCTACCTCAACTACTGGCAAAAAGTTTGATCGCATGAGGACAATGACACTGAATGATGATGTACTTGTTGGGAGAAAGACTGAGAAATCTGAAGTTATTGATTTGATTGGTCAACCTTCGGACAAGCTCTGTAAGGTGATCTCTGTGTGGGGAATGGGAGGACTTGGGAAAACTACTCTTGTCCGAAGCATCTACAGAAGCCAACAGCTTGATGACTGGAACTGGAAGCGCGCTTGGGTCACTGCTACGCGTCCTTTCGACAATGAAGTGCTCATCAGAACCCTAGCTTCGCAACTACTCCACGATTCTAATACAGCAAATATGAAGTTGGATGACTTAAGAAAAAAGATAGACCAGCTTCTACAGAACGATAATAACTATCTCATTGTTCTTGATGACCTATCATCTATTGAAGAATGGAACTTGGTTCGAGACCAATGGGCAAAGGCTAAACGCATCATAGTCTCCACAAGAGAATATAGTGTTGCCAAATATTGCTCGGGAGGAGACATGAACACGCACAAACTTCAAGTCCTACAAGAAGAGGATGCACTTGCCCTCTTCCTAAAGAAGGTGTTTAACAACCACACTGAAAATACAAATTTAGGTACTGATATGTTGGATCAAGCAGCACTAATCATTAAGAAGTGTCATGGACTTCCCCTTGCAATATCTACAATAGGTGGATTCCTAGCTAATAAGCCCAAAACTGCTACTGAATGGAGGAAGGTCAATGATCGCATCAGGGCTGAATTGGACATGAATCCGGAACTTAGGAGTATAAAGACAGTTCTTATGAGGAGCTATGACGGTTTACCATACCATCTCAAGTCCGCTTTCTTATACATGTCCATATTTCCAGAAGATTACAAAATTAAGCGGAAACGTTTGATAAGGAGATGGATTGCTGAGGGTTACTGTAGAGGAATGCATGGCATGAGTGCAGAAGAAGTTGGTGACAGATATTTTGAGGAGCTTTTGGATAGGAGCATGATCCTGCTGTTGGAAGAGGACACTAAGTATGGTGGGACCATTGGTTCTTGTCAGCTTCATGATATCATCCGTGAAATATGCATACTAAAGGCTAGGGAGGAAAACCTTGCTTTTACTCTGGAGGAAGGGTGTTGTTTGAGCGACGCACAAGGTACAATACGTCATCTTGCTATAGGCAGCAACTGGAAAAGAGATAAAGATGTACTGGTGGAGAGCATGCTGGACTTGTCGCATGTGCGGTCATTGACCGTGTTTGGAGAATGGAAACCATTTTTTATTTCTGATAAGATGAGGTTTCTCAGAGTGCTCGATTTGGGTGATACATTGGGGTTAAGAGATCATCATCTTGATCAGATTGGGCAGCTCCTTCACCTCAAGTACCTGTCTATACGAGGATGTTCCAGTATCTGCTGCCTTCCAAGTTGTTTGGGAAATCTGAGGCACCTCCAAACTCTGGATGGTAGAGGTACAAGAATAGCCAGTTTTCCATCTGCGATCACCAAGCTACAGAAGCTAGAGCAACTTCTTGCATCTGGTGCTGATGATGGATTCAACACGCGAGATGTATTGAACACCTTCTTCAGGTTTCTATTTGGGGGCGTTAAAGCTCCTAGAGGAATCAGGAAACTGAAGGCCCTGCACACACTAGGAGTTGTCGATGTTGCACGGAGCAAGACCACTCTGAAAGAACTTGAAGAGCTTACTCAGCTACGTAAGCTTGCTGTGGCTGGGGTGCACAGCAAATACAGCAACAAGTTCTGGTCTGTCATTTCCGCTAACAAGCAACTCCGATCTCTGTCAGTGAAGGGTTTAGGGTTAGATAGTTGTTTCGGTGGGGATTTGTTGCCACCAATACACCTTGAGAGCCTCAAGCTGGAAGGCCAGCTTGTCAGAATAAATGAGTGGATCCATAAGCTTCAGAATCTATCCAAGTTGCAGCTGTATAATACCGAAATTGACTCAGCTGATCCCATGCAAGCTATTGGACAGCTACCAAATCTTACAATGCTGACTCTGTGTTATGGTTCTTTTATGGACAACAGCGAGCTCCTTTTCCATGGTCCATCGTCTTTCCCGGAGCTCATGGTGCTGAAGCTCAAAGGAGTAGATTGCGAGATAGTATCTTTTAAAGAACAGGCAATGCCGAAGCTCGAGCTGATACACGCTGATTTTCCGGGGGGAATTTGTGGGCTAGCATTTCTCGCGAGCCTCAAGGAAATTCGGCTGGGTAGTAGCACCAGAGACACATTGAAGAAAGATCTGCAGAAGCAGCTCACAGGAGATCTAGAACGTGTCAGTCTGAAGCTGTTGTAA

>B97_Zm00018ab430370

ATGGCAGAAGGTTTGATACTAGTTGTGCTTCAAAAAATCGCAACCACCTTAGGAGGAGCTGCATTGAGTGTAATTAAGTCAAAATTAGGGAAAGGAGCCAACATTTTACTTGAAGCAGAGAACAGCATGAAAGAAATTGAGAGTGAGTTCGAAATAATGCAAGCATACATAAGTCAAGCAGACCCGTACAGTGAAAGTAACAAAATTCTAAAACCATGGTTGAAGAATGTAAGAAAAATAGCTTCTGAAGTCGAGGACATCATTGATGAATATGCCTTTCTACTTGGAAAACTGGACAACGCAGGAAACTTGGCGAAGAAGTTCCACCATTCGAGATACATCACTGCATGGAGTGACATTTCTTCTCAGTTGAAGCAAGTGCAAGCACGCCTACAAAATTTGACAGTTCTGAAAGACAGATATGGCATCACAGTAGTTGGACCTGGTGGTGGATCATCAAGCCATAATAACAGTCGTAAGAATTACCTGTCTGAATCTTCCTACCTGAATGATGATGGTGATGGTGTAATGGTAGGCAACGAAGATGAAGTAAAGAAGTTGACAGAATGCATAGATGATGCTGGTGCAGATCGTGCAGTTATCTCCATCTGGGGAATGGGCGGTTCAGGAAAAACAATCCTTGCAAGGGACATCTATAGAAAACGAGAAGTTAGAAAGAACTTCCAGTGCTGTGCCTGGATCACAGTATCACTGAATTACCAAGTCGAAGATCTTTTGAACAAGCTCATAAAGCAACTCCACATCCAAGATGTGCCTGATGCAACTGACAGTACACATTTGGTTGCTAGGATCCAGAATCATCTCAAGGACAAGAGGTACCTTGTTGTTTTAGATGACATGTGGAACAGGGAGTCTTGGTTATTCTTTGATCGAGTATTTGTCAAGAATCTTTATGGAAGTAGAGTCATCGTTACCACTCGAACAGAAGCTGTTGCGTCAATAGCTGAGCTGAACCATACTATAAGAATTGGCCTTCTTTCACAGGGGGAGTCATGGAAGCTCTTCAGCAGAAAGGCATTCTCAAAGATAGGCAAGGAAGAACCGACCTGCCCTCAGGGCCTTGTTCAATGGGCAAACAAAATCCTCGAGAGATGCCAAGGTCTGCCACTTGCTATTGTAGCCATAGGGAGCCTTCTGTCATACAGAGAAATGGAGGAACAAGAGTGGAGAATCTTCTACAATCAACTTAATTGGCAACTGACAAATAACCCAGAACTCAACTTCGTATCGAGCGTCCTAAAGCTAAGCTTGAATGATCTTCCAAGCCACTTGAGGAATTGCTTCCTGTACTGTGGCTTGTTCCCCAAAGACTACCAGGTACGTCGGAAGTGTCTAATCAGACTGTGGATAGCTGAAGGTTTTGTGGAAGACCGGGGAACAGAGATTACACTGGAAGAAGTGGCCGAGGAGTACCTCAAGGAACTCACACGGCGTTCACTGTTTCAAGTGATGGAAAGGAATGAATTTTCACGGCCAAGAAGATTCCAGGTGCACGACCTTGTTAGAGAGATGACACTGGCCATATCAAGAAATGAGAGGTTTGGTCATGTAAGTGACCAGCCAGATGTAACAGACATTGGCGATGTAGGAAAACGTGTATCAGTGCACAGTGGGGGCCAAATCTATCAGCCAGGTCCGTCTTCTCAGCATCTGCGTTCATTTTTGTTGTTTGACAAACACGTGCCGCTTTCATGGATCAGCATCGCTTCATCGGACTTCAAACTGTTAAGAGTCCTGTGCCTCAGGTACTCCCTCCTTCAGGACATTCCAGATGCCATGACTTGTTTGTTCAATCTGCACCATCTAGACTGCTCTCGTACTAAAGTAAGGAAGGTACCAAGATCAGTGGCAAGGCTGAAGAAACTAGAGACATTGCATCTCAGGTTCGCCCGTGTGAGGGAGTTGCCGTCTGAAATAACAATGCTCACAAACCTCCGCCACTTGTCTGTGAGTGACGACTTGTATGGCACATCAATTTGTGGTACTATCCGCAGTCTCAAGCACCTGCAAACTCTTCGAGAGGTGAAAGCCAACAAAGATTTGGCTAAAAGTCTTGGTTACCTGACACAACTAAGAAGCTTAGGAATTACTGGAGTCATACAAAGCCACAATGCAGATCTTTGGGCTTCTATCAGGAAGATGACTGTCCTTAACAAACTAGCCGTCGCAACTCCTGGCGAGAGTAACGAAGTGCTTAGTTTTGAAGAGCTGAGGCCGCTCAAGAACCTGGAGAAGTTCTACTTGACCGGCAAGTTGGCAGAAGGGAAGCTTTTTCCAGTATCTAATGGTTTTCAGAAACTTAAGGTCCTAACAATGCGCTGGTCTAGGCTAACACACGACCCCCTAAGTTCCTTATGTCAAATGGAAAATCTCGTCTACCTTAATCTTTACTGTGCATACGATGGGGAATGCTTGATCTTCTCTTCTGGGTGGTTTCCAAAGCTCAAGCAACTCTACCTAGGCAAGCTTGAGAGACTGAGGTCAATTCAAATAAGTGATGGCGCCATTGAAAATTTAACATACCTGGAACTTCATGAGCTGTGGAATCTGAAAAGTGTCCCTGAAGGCCTTGTATACCTTAGGTCTCTTCAGCACTTGTATGCACGAAAGATGCCTGCAGATTTCGTGGAGGAACTGGAGGGAAGTTGCCTGGGCTTGGTTCGGCACATTGCCAACATTGAATGTATGTGA

>B97_Zm00018ab434530

ATGGCGGTGGTCCTGGATGCTTTGGCATCCTACATCCAAAACATGCTGACACAGATGGCGAAAGAAGAGGTGGACATGCTGCTTGGGGTCTCCGTTGAGATTGACAACCTTGGCGCCAAGCTCGGGGACCTCAAAAACTTCCTCGCTGACGCTGACAGGAGGAACGTCACCGACCGAAGTGTGCGGGCATGGGTGAGGGAGCTCAGAGACGCCATGTACGACGCCACCGACATCCTTGACCTGTGTCAGCTCAAGGCCTTGGAGCGAGGTTCGTCATCATCCCTGGCTACGGGGTGCCTCAACCCCTTGCTGTTCTGCATGAGGAACCCCGTCTTCGCCCACGACATCGGCAGCCGCATCAAGAAGCTCAACAAGAGGCTAGACGCCATCAAGAAGAACAGCGCTACCTTCAGCTTCATCAACCTTGGTTCCTATGAGGACCGCGGTGGGAAGGCGGAGACTCCATCTTGCCTCGCCAACCGCGAGACCTCGGCGCAGCTCGACCGGTCTAGTGTGGTTGGCGAGCAGATTGAGGTCGACACAAGGAAGCTAGTGGAGATGCTAACAGAAGACCCTGGAACGACCACTGCTACCCATGACCAAGGTACGGTCTTGGCCATCGTTGGCATTGGCGGAATTGGCAAGACAACCCTCGCCCAAAAGGTCTTCAACGACGACACCATCAGCCGTGTCTTCACCAAGAAGATTTGGCTGAGTGTCAACAAGGACTTCAGCGTGGCAGAGATCCTAAAGAGAGCCATCATCGAAGCCGGGGGAGATCACCATGCAGCTGGCAATGCAAAAGCCACACTTCAGCGGACGCTTCAGAATGCCTTGGATGGGCACAAGACTATTCTGGTCATGGATGATGTTTGGGATGACAAGGCATGGGGCGATGTTCTTAAAACGCCATTTGTCAATGCTGTTGGTGGTGGTAGCCGAGTCCTCGTCACCACAAGGCATGACTTGGTTGCACGAGCGATGAAAGCCAGGGAGCCCTACCACCACGTCGACAAACTTGACCCCAAAGATGCCTGGTCCTTGCTCAAGAAACAGGTAATCAGAAATGGAGATAATGAACCTCTTATTGATATGCTAGAAGATATTGGAATGAGAATTATAGAAAAATGTGATTGCCTGCCCCTTGCTATCAAAGTAATGGGAGGACTCTTGTGCAAGAAAATGGCAAGACGAGGAGACTGGGAAAGGGTATTGAATGATGCAATATGGTCAGTATCAGGAATGCCCGAAGAGCTAAATTATGCAATCTACCTTAGCTACGAAGATTTGCACCCTTCTCTAAAACAATGCTTTTTACACTACTCCCTCATCCCTAATGAGAGCACAGTGTTCTTCGTCGACGACATTGTGAGCATGTGGATCAGCGAAGGATTTGTGGAGGGGAACTCTGACGAATTAGAAGAATTAGCGATGGAGTACTACAATGAGCTAATATTGAGGAGTCTTATAGAACCAGATCTTCTGTATGTTGACAAATGGGTTTGCAGCATGCATGATGTAGTTCGCTCATTTGCACAGTATGTGGCAAGAGATGAAGCACTTGTAGCACGAAAGGGGCAAATTGATGTTGGTGAACTCAACTCAAAAAGGATCATTCGACTATCACTGGAAACTGAGGAGTTAGAATGGAGCACTCTGCAACCACAAAAATCACTAAGAACACTACTAGTAGCTGGGCACATAGGGATTAAAGTTGGTAACTCACTGGGTGCCTTTCCAAGCCTACGAACCCTGCATATAGACTCAACAAACTTTGATGTAGTGGCTGAATCACTGTGTCAGCTCAAACACTTGAGGTATTTCTCTGTCACAGACCCTAACATGTCTAAGCTGCCGGTAAACATTGGAAATATGAAATTCTTGCAGTATATAAGCCTTGACAGCTGCAAAAACCTGGCTAAGCTTCCTCGTAGCATTGGAAAGCTACAACAGCTTAGGTATCTTAGCCTAATGGGAACAAATATACACTTCATACCGAGAGGGTTCTCTGTCTCAACTAGTCTGAGGAAGCTCTTTGGGTTTCCAGCCCACATGGATGGCAACTGGTGTAGCCTGCAAGTGTTGGAGCCTCTTTCCCGGCTCATGGGCCTCTCTATATATGGTCTAGAAGGTGTGTCTTCTTCCTCCTTCGCCGCAAAGGCTAGGCTTGGTGAGAAGGTGCATCTTAGCTATCTGGAGTTGAGCTGCACCAGTAGACTCAAGGATGACACGCAACTAGTCAAAGAAGATGATGAAGGCTTCTCTGAGGAAGAGCAACAGCGTATTGTGGAAGTGTTTGATGAGCTCCGCCCTCCACCCTGCCTAGATGCTCTTGAAATTGAAGGGTTCTTTGGGCGGTGCTTCCCAAGGTGGATGGGACCAATGGCAGCTGTACCCCTTGAAAACCTGCGGATCCTAGCGATGGATGACCTACCTTGCTGCACTGAGCTCCCCAATGGCCTGTGTCGTCTGCCGTGCTTGGAGTTGCTTCAAATCTGTCGAGCACCAGCCATTGAGCGTGTCGGGCTAGAATTCTTGCAGCCCCATCACCACCATACACACCAGTTGACTGATGTGTTTCCTAGACTACACGACTTAACCTTAACGGAAATGGTGGAATGGGAGGAATGGGAGTGGGAGGAGAATGTGCGAGCCATGCCATTGTTGGAAGAGTTTCTTCTAGAGAGCTGCAAGTTGAGGTGTATCCCTGTTGGCCTTTCTTCCCACGCGAGGTCCTTGAAAAGGTTATATGTACATGACGTACAACACCTA

>B97_Zm00018ab439450

ATGGATCTTGTGGCCGGCGCCGTGGGCAGCATCATCCGCAAGCTCGGCGAGCTGCTCCAGGCAGAGTACAAGCTACAGGCGGGCCTGCTAGAGCAAATCGAATCTCTGAAAAATGAGCTCGAGAGCGCGCACGCGGCTCTCCGCACCGTGTCGGAGGTGCCGCAGGAGCAGCTTGATCCACAGGTTCGGCTCTGGGCTCGTGAGGTCAGGGAAGCGTCGTACGACATGGAGGACATCCTCGACACATTCCTCGTCGACGGCGCCCCGGCTGATGGCCTGGGCAAAGGTCGTCGTCTCCTGAAGAAGATGGAGAAGCTGTTCAGAAAGAGCAAGGAGCGCCACGCCATTGCTGGCGCCATTCAGAAGATGAAGGGACGGCTCCAGGAAGTGGCTGACCGCCGCGACAGGTACGCCGTTCCGGTGGCAGCGCCAGCGCCGGTGAGGACGCTGGATCCTCGCCTCGTGTACATGCACAGGGAAGCGGCACAGCTCGTCGGCATCGACAAGACCAAGGCTGAGCTCATGGCCATGCTTCTGCCGCTGTCGTCATCCCGCTGCCCTGAGGACGACGTCGATGTCTCTGCCAGCGACGGTGACAAGATGAAGATAGTTTCTGTGGTCGGAGCTGGTGGCCTTGGAAAGACCACTCTAGCCAAGGCCGTCTACGATGAGCTCAAACCGCGATATGATCATGGAGCATTTGTTTCGGTTGGCCGAAAACCTGACCTGGTGCAAGTCTTTACCAGCATCTTCTTCCATCTCGACGAACAAAAATACAATGCCATTCGTGAAGTGAAGGACCTACAGCTGTTGGCTGGCGAACTACGAAGATTTCTACAAGACAAGAGGTACTTGATCGTTATCGACGACGTTTGGGATACAAAATCTTGGGATACAATAAAATTAGCTTTTGATCAAAAGAATAAGCAGAGCAGAGTAATCACAACCACTCGCAACCGACAAGTAGCTTCCAGTGAGGAGGTTTACGAGCTACATCCGCTCTCTCATGACAGCTCAAAGAAGCTATTTTATATGAGGCTGTTTTGGGGTGAGGACAAATGCCCGGCTAATCATCCTGAAGAGGCATCTCAAAGGATTTTGGACAAATGTGGTGGTGTACCATTAGCTATCATCACAATGGCAAGCTTGCTAGTGGGTAAATCGAGAGAATATTGGTTGGAGGTGTGCAACTCACCTGGTTTCTATCGCGGTAAAGATACCAACGAACAAGTAGACGACACCGTGTGGATACTGTCTCTGAGCTATTATGACCTACCTTCGTATCTAAAAACTTGCTTATTGTACCTAAGTGTGTATCCAGAAGATTATGAGATCGAGAAGCATAGATTGATATGGAAGTGGGTAGCTGAAGGTTTCATCGAGAAGAATGCAGGAAGCAGCAGCTTGTTTGAGCAGGGAGAGGAATACTTCCATGAGCTCATAAATAGAAGCATGATCGAGGCGATGGAGTTCGACGAAGGGTTTGGCATCATAATTGGTTGTCGCGTTCATGACATGGTGCTTGATCTCATCCGTGACATATCAAACAAAGAAAATTTTGTCACTGTCTCATATGATGATGGTAGAAGAGGCACAACGACGTCGTCATCGTCACGAAACGTGGTGCGTCGGCTAGCTCACCAAAATAGAAGAATGACAGAGGACAATCCTGTGGAGGGCAACATGACACATCTAAGGTCACTAGTTGCTTGTGGGTGTGATATGGATGGTTGGGTCATGCACCCGAGCTCTACGCTGCTATTGCGTGTGCTAGCTTTAGAGCAATGCTGCACACCACCATCTATGGACATTGGTCATCTTGGAAAACTGCTTCATCTCAGATACCTTGGGCTACGTGGTACTCTCGTAGACAAGCTCCCAGGCGAAATAGGATCCCTCAAGCTTCTGCAAGCACTGGATTTACTAGGCACCGGAATATCACGACTTCCACAGACCGTTTGCCTGCTAACGCAGCTGAAGTACCTATACGGTGATGCATGCACGATAGTGCCCGACGGTTTCCTCAGGAAGGTGACGTCACTGGAGGAGCTGCATATACATCCTCCTAGCGAAGGTGACGAGTACAACCAACAACAGTTTATGCAGGATTTGGGCACCCACCAGGGAGAAATTAGGGTGCTCGATTTGATGAGGTTCAGAGATGAGTTTGACGATCTGAGCATGGAGTCTGGTCTAGTGCAGGCCCTAGGAAGTCTGCACAAGCTGCAGACCCTACTAGTGAGTAGTGATTACACGAAGCAACAAGTCGCACAGTACAGCTGGGACACGGCGGCCCTTCCGCGGTGTCTCCGGATCTTGGTCTTCGTTGACCTCAGGTTCCATCATGTACCATCGTCCATCAATCCCGCGAGCCTCCCCAACCTCTCACGACTGGAATTGTCTGTGGGTCATCTAGACGAGGCAAGTCTGAGAGCCCTGGGTGGGTTGCCAGGGCTCACCTACCTCACACTAACGGCGGCTGATTGGCTGAAGAGCTCATGCAAGGCTTCGGTAGTTGATGTCGTTGTCGCTGATGGCTTCTTCCTCAAGTTGAGATCCCTCAGGCTGTATGGCTGGATGCTCCAGTTGGTGCCCAGCGAGGACTCGACTAGTGTTTCGTTCAACATCTGGAAAGGAGATGAGGACGTTGTGGCCCTTGGTTTCTGCAGAACAAGTGTAGCAGCACCTATGACCTCCTCTATCATCATGCCAGACCTCATAGACCTGTGGTTCTATGTCCCTGTTAGAGCCTTGTGTAAGAGTAGAAATGGAAGCAGCTGTGACAGCCTCGGCTGGGAGTGCCTCCCTTCGCTACACAAAATCGATGCAGTTGTCGACAGTAAGGGCGCCTATATCGGCGATGTGAAGAAGGTCGAGGCTGAGATGAGGCAGGCAGCAAAACTGCATCCCAACCAACCCATAATTAATATTCTACTACTCAATCAATATATTTGA

>B97_Zm00018ab439470

ATGGATCTTGTGGCCGGCGCCGTGGGCAGCATCATCCGCAAGCTCGGCGAGCTGCTCCAGGCAGAGTACAAGCTACAGGCGGGCCTGCTAGAGCAAATCGAATCTCTGAAAAATGAGCTCGAGAGCGCGCACGCGGCTCTCCGCACCGTGTCGGAGATGCCGCCAGAGCAGCTTGATCCACAGGTTCGGCTCTGGGCTCGTGAGGTCAGGGAAGCGTCGTACGACATGGAGGACATCCTCGACACCTTCCTCGTCGACGGCGCCCCGGCTGATGGCCTGGGCAAAGGTCGTCGTCTCCTGAAGAAGATGGAGAAGCTGTTCAGAAAGAGCAAGGAGCGCCACGCCATTGCTGGCGCCATTCAGAAGATGAAGGGACGGCTCCAGGAAGTGGCTGACCGCCGCGACAGGTACGCCGTTCCGGTGGCAGCGCCAGCGCCGGTGAGGACGCTGGATCCTCGCCTCGTGTACATGCACAGGGAAGCGGCACAGCTCGTCGGCATCGACAAGACCAAGGCTGAGCTCATGGCCATGCTTCTGCCGCAGTCGTCATCCCGCTGCACTGAGGACGACGTCGATGTCTCTGCCAGCGACGGTGACAAGATGAAGATAGTTTCTGTGGTCGGAGCTGGTGGCCTTGGAAAGACCACTCTAGCCAAGGCCGTCTACGACGAGCTCAAACCGCGATATGATCATGGAGCATTTGTTTCGGTTGGCCGAAAACCTGACCTGGTGCAAGTCTTTACCAGCATCTTCTTCCATCTCGACGAACAAAAATACAATGCCATTCGTGAAGTGAAGGACCTACAGCTGTTGGCTGGCGAACTACGAAGATTTCTACAAGACAAGAGGTACTTGATCGTTATCGACGACGTTTGGGATACAAAATCTTGGGATACAATAAAATTAGCTTTTGATCAAAAGAATAAGCAGAGCAAAGTAATCACAACCACTCGCAACCGACAATTAGCTTCCAGTGAGGAGGTTTACGAGCTACATCCGCTCTCTTATGACAGCTCAAAGAAGTTATTTTATATGAGGTTGTTTGGGGATGAGGACAAATGCCCGTCTAATCATCCTGAAGATGTGTCTCAAAGGATTCTGGACAAATGTGGTGGTGTACCATTAGCTATCATCACAATGGCAAGCTTGCTAGTGGGTAAATCAAGAGAATATTGGTTGGAGGTGTGCAACTCACCTGGTTTCTATCGCGATAAAGATACCAACGAACAAGTAGACGACACCGTGTGGATATTGTCTCTGAGCTATTATGACCTACCTTCATATCTGAAAACTTGCTTATTGTACCTAAGTGTGTATCCAGAAGATTATGAGATCGAGAAGCATAGATTGATATGGCAGTGGGTAGCTGAAGGTTTCATCGAGAAGAATGCAGGAAGCAGCAGCTTGTTTGAGCAGGGAGAGGAATACTTCCATGAGCTCATAAATAGAAGCATGATCGAGGCGGTGGAGTTCGACGAAGGGTTTGGCATCATAATTGGTTGTCGTGTTCATGACATGGTGCTTGATCTCATCCGTGACATATCAAACAAAGAAAATTTTGTCACTGTCTCATATGATGATGGTAGAAGAGGCACAACGTCGTCATCGTCACGAAACATGGTGCGTCGGTTAGCTCACCAAAATAGAAGAATGACAGAGGACAATCCTGTGGAGGGCAACATGACACATCTAAGGTCACTAGTTGCTTGTGGGTGTGATATGGATGGTTGGGTCATGCACCCGAGCTCTACGCTGCTATTGCGTGTGCTAGCTTTAGAGCAATGCACACCACCATCTATGGACATTGGTCATCTTGGAAAACTGCTTCATCTCAGATACCTTGGGCTACGTGGTACTCTCGTAGACAAGCTCCCAGACGAAATAGGATCCCTCAAGCTTCTGCAAGCACTGGATTTACTAGGCACCGGAATATCACGGCTTCCACGGACCGTTTGCCTGCTAACGCAGCTGAAGTACCTATACGGTGACATATGCACGAGAGTGCCCGGTGGTTTCCTCAAGAAGGTGACGTCACTGGAGGAGCTGCATATATGTCCTCCTAGCGAAGGTGACGAGTACAACCAACAGTTTATGCAGGATCTAGGCACCCACCAGGGAGAAATCAGGGTGCTCCATTTGATGGGGTTCATAGATGAGTTCGATCGGAGCATGCAGTCTGGTCTAGTGCAGGCACTAGGAGGTCTGCACAATCTGCAGACCCTACAAATGAGTCCATTAGCACTAGACATCTGGGACACAAAGGAACAATTAGCATCAGACAGCTGGGATACGGCGGCCCTTCCACGGCGTCTCCGGATTTTGGCCTTCATTGATATCAAGTTACATCGTGTACCATCGTCGATCGGTCCCGCTAGCCTTCCCAACCTCTCACGACTGGAATTGTCTGTGGGTCATCTAGACGAGGCAGGTCTGAGAGCCCTGGGCGGGTTGCAAGAGCTCACCTACCTCACACTGTTTGCGGCTGATTGGCCGAAGGGCTCATGCAAGCCTACGATAGTTGATGTCGTTGCCGCGGATGGCTTCTTCCTCAAGTTGAGATCCCTCAGGCTGTATGGCTGGATGGTCCAGTTGGTGCCCAGCGAGGACTCGACTAGTGTTTCGTTCAACATCTGGAAAGGAGATGAGGACGTTGTGGCCCTTGGTTCCTACAAAACAAAAGGAGACTGCAGCAGGAGAGTAGCACCTACGACCCCTATCATCATGCCAGACCTCGTACACCTGGAGTTCAGAGTCGCTATCAGAGCCTTGTGTAAGAGTAGAAATGGAAGTAGTTGTGACATCCTCGGCTGGGAGTGCATCCCTTCGCTACACAAAATCATGGTATCTGTAAGTTGGGAGGGCGTCTATGATGACGATGCGGAGAACGCAGTGGCTAAGATGAGGCAGGCAGCAAAACTCCATCCCAACCAACCCATACTTAAGATATTCTAA

>B97_Zm00018ab440080

ATGGATCTAGTGACAGGTCCGCTAGCCATTCTCCCGTCCAAGCTGCAGGAGCTACTCCAAATCCAAGATGAGTATAGGCTCCAGAAGGAAGTGAGGGGCAGAGTGGAGATGATCTGCCGGGAGCTGCAAAGTATGCACGCTGCCCTCCGTAAGGTGGCTGATGTGCCGCGGGATCAGATCGACCTCCAGACCAAGGTGTGGAGACGCGATGTCAGAGAGGCATCCTATGACATTGAGGATGCCATCGACACCTTTCTCATGCGCATCAGAGACCGCGCGCCAAGTGACCCAAACAGGTTTCGACTTGCCATGAAGAAGATGACCAAGCTGTCAAGAAAGGTTAAGGCTATCCATGAAATTGGTGCTGCCATCGGAGACATCGTGAGGAAAATCCAGGAGTTGGTGGACCGGCGTGTCAGGTACAAACTTGATGTTGCTGCAGACAGGTCAATCAGCATGGTTAGTGTTGATCCTCGCCTTGCAGCTTTGTACAAAGAAGCGACACAACTCATTGGCATCGACCAATCAATGTCTGAAATAATATCCATGCTGCTCTCGGATGAGTCGGGCCAGCAACACATTAAGAAGATTTCTATTGTCGGAGCTGGAGGACTAGGCAAGACCACCCTTGCTAAAGAAACATATGAAAGGCTTAGAAGTGAATACGAGTTTGCAACTTTTGTTTCAGTTGGCCAAAATCCTGACTTGGTGAGAGTTTTCAAGAACATCCTCGTTAAGCTTGACATATACCAGCAAGTGGACACTATCAACAGAACAGATACGCGAGCGCTCATCGATGAAATCAGACAGCGCCTTAGCTATGTGAGGTGCAGGTATCTCATTGTTATCGATGATGTATGGGATCTGGAGTCTTGGCATACACTAGAATTGGCATTTGTTGAAAATAACAATGGGAGTGGAATAATCATAACTACTCGTAATTTTGCTGTTGCCACAATAGCTGGTCGTGTTTACAAGTTAAGGCCACTTTCGTACCATCACTCCAAAGAATTATTCTACAGATCGTTATTTGGTGAAGGAGTATGCCTTGATGATCAAATAATCAATAAGGCATCAGATAAATTTCTAAAGAAATGTGATGGTATACCGTTAGCTCTCATCACAATGGCTAGTTTGTTCATGGGAAAACCAACACAAATGTGGTCTGAGCTAGCCAATAATGCTTTTGGACATAAAGGTCGTAACAGTCATTTAGAAGAAACGACTATGAGGATATTATCACTTAGCTACTCTGATTTGCCATTGAATTTGAGGACTTGCTTTGTATATTTGAGTAGATATCCAGATGATTATGTTATTAATAAAAGTTCTTTGATATGGAAATGGATAGCTGAAGGTTTTATCCAAGAGGAGCAAGGAAAACAGTTATTTGAGCTTGGGGAAGAATTCTTCAACGAACTCGTAAGTAGAAGCATTATACAAGTGGTCCAATCAAAATTTGATAACACAGTGCAAGGCTTTCGTATTCATAGTATGGTTCTTGGTCTTATTCGCCTGTTGTCATCCGAAGAAAACTTTGTTTCTGTATTGGACGAAGAAGGGCAAATATCTATATCAGGCAAACTTCGTCGGTTAGCCATCCATAACAGAAAAGAAGAGCACAACCTTGGAGACAATATGGACCTCCTCCCATGGTTGAGGTCGTTTACTGCAATTGAATGTCCTATATACATGATACCTCCGGTTTTCATCTTCAAATTGTTACGTGTTCTGGATTTAGAGAGTTGTGGGTCTATGGAAGGTTATGATCTCAAGCAGCTTGGAAATTTACATAATCTCAGGTTTATCGGTCTATGTAATACATATGTCCGGACGCTCCCACAGGAATTAGGGCATCTAAAGTTTCTGCAGACGCTTGAATTGAAAGGAAGTGGCGTTGAAGAACTACCTTCGAGTATGGGTGAGCTTGCAGGGTTAATGTGCCTCAACGCTGACTGGACAACAAGAGTGCCCAAGTGGATTGGGATGCTTACTTCCCTGCAACAGTTGGTGATGTACCCTTGTGGTGGCTACTCGGCAAGGTGGTTTGTCAAGGGGCTGGGACACCTGAGGGAACTAAGGATGCTCCGTTTGTTGATAAAAGCAGATGACGAGAAGCAGCTTGCACAATTGCTGGAGTCTGTTTTAAAACTGCCCAAGATCGAGGCTTTACATCTTGATTACTATGGCGGCGTACAGTTAAACAGGCTTGTTAAGTTGGAACCTAGTGATTTTGCCTGCAGTGGACGTCTTCGTTCCTTAGAATTGCAGTTGTTGGAATTCTCAAGGCTGCCTGTGTGGCTTAATGCTTACTATCTTCCACAGATCCGTGACTTGTCGCTGTTGATGTTTGATGTGGATAAACAGGATCTGAAAAAACTTGGGGACTTCAAGGAGCTCCGTCATCTCCATCTGCTAATTGTTAACACTGAACGCAGAGATGCTATTACTTGTGCCAGTGGTGGATTCCGGAATTTGAGATTCTTCAGTATCACTAAACCATTCAAGTTTCAACAGGGAGATCTGCCTAGGCTTGAAATCCTTGATTTTCATTTCAATGTGCCACTCCAAAGTGGTGCCAATAGTGGCCTGGATTTTGACTTCGGTTTGGAAAATATCCCTTCGCTTCAGCAAGTCATTGTTCAAATCAACTGTCTAGATGCCTTTCCTCTTCCAGAGGAGGTGGAGACAGCCCTAAGGCACGCAATCGGCGTCCATCCCAACCGTCCCATCGTTGACATCAGTTTGCCTCGACGAAAATTAGACAAGGCAGACAACGACGGGGCCGAAGGGCGTAGTAAGTTGGCTCATTATTCAAAATCATTATCATCGGCACATGATAAGCCAATGGATGAGCTAATAAAGAGGTTATATGTGGTGGACAATGAGGCCTACGATAAAAAGATGAAGATTGTGCCTATTGTTAGATCTGAAGGGCTGGGGAAAACTACTCTTGCCCAAAAAGTATTTGACAAGCTTAGTCCACATTTTGACTGTGCAGCATTTGTTCTGGTAGGCCAAAATCCGGACATGAGGAAAGTTTTCACTGACATTCTCATAGGTCTAGACAACCAAAAGTACCAAGATTTCCCTATGACTATATTGGATATAATTGAGCTGATTGGGTTAGTCCGTAAATCGCTCATAAACAAAAGGTTCTTTGTTGTAGTTGACGATGTATGGGATTTGAAAGCATGGGACATAATAAAGTCTGCATTGATCGAAAACACTAATGGCAGTGCAGTTCTAACAACGACAAGTCGTCAGATCTGGGAACATGAGAACTGGAGGCCATCTGGGGATGACAAGTCGGATTCTGACGCAGAGCAGTTAGAGCCTCATTTGGACGTCACAACCCCCGCGGATGTCCTCCCAGCGTCTTCCCCGAGCAAGAGCAAGGTGGTGGCCCATGTGGAACTCCGCCACGACGACCCAGATTCCTCTGCATTTGGAATCCCGCAAGTAGCAGGGGATACTCAACCTGAGCTCCGCCTTTTCCCCGAGGCGGATGCGGATCTTAAAGGGTTCTTCTACGAAAACCTATTTGGATCGGAAGACAAGTGCCCTGATGATCTGAAAGATATATGTAAAGATCTCATACAAATATCTGCTGGTGTCCTATCAGATGCCTATGAGACCGTGGAAGAGCTCAGAGGTATACCTACGACACTGGAAAGCTGGCAGCCTGTGTATCAGAGAAGAAAATTCGAGCTCTCTTATCCCAAACTACCTGGCCATCTAAAGGATTGCTTAAGATATTTTAGTATGTTTCCAAAAGGTTACGAGATCAGTGCAGAACGCTTAGTTTGGGCGTGGATAGCTCAAGGTTTCGTAAAGGAGACTCAGGGACAGAATGATTTGGAGAAAGTCGGAAAGAAGTACCTTATTGACCTTATAAGCAGAAAGATGATCGAAGCAGTGGAGGTTCATGCTGATGGTATGGCCCTCTCATGCCGTGCGTACGATTTGTTGCATGGCTTGATCGTTTCATTGTCAACAGAAGAAAAATTCGTTGAAATTCTAAATGACTCGCAGTCGCAGGAGAGAGCTTCGAATCCGAATACAGAAGTTCACCGGCTGTCCATCCAAATCCAGAGAAATAATATGCTACACCCCCTGCCAAAAGCTTGGATGAAACAAGTGCAGTCACTTGTTGTCTCGTTTGATACCGTGCCAGACCTCTCAGATTTTCAGGGTCTTCATGCCATGGACTTAGGAGGCTGCGATTCATTGCAGGTTGATCATCTCAAGGGTATAAGTAAAACAACATCTTTGATATATCTGGCCATAGGAGGAAAATATATCGCTGACGTCCCGAAAGAAGTTTCTTGGCTCAAAAAGCTGCGGACATTGGATTTAACTGCAAGTGGTCTGAGTGAATTGCCACGATGTATTTTAACGCTAACTAATTTGGAGCGGCTATTTGTTAATAGCCATATGAAGATTCCTGGTAAGATCTCAAAAATGTCAGGTTTACAAGAACTGGGCGACATCAATATTGTTAAGCCAGAGTTACTTAAAAGGCTCCGTAAATTAAGTAAGTTAAGGGTTCTCAGAATTGCCATGTGGTCTTGGGATGAGGGTTCTAAGAGCTCTGTTAAACTATGGTGGGAGACCCTGCACTCATTAATCGGGTCCTGCAAAAAAATTGAGAGTCTCTGCATACTGACCTTCTGCTCCCTTGATTTCATGGATGGTTCCGGTGATGATAATAAATGGCCGCCTCAAACTCTCAAAAACCTCGAGATAAGTCACAGCGCATTTCTGGATAAGCTCCCAAGTTGGTTTGACTCACTTTCCACCATCTCCTCGTTGACAATCGAGGTCAACAAGTTATCAAAGAACATCACTGACACTCTGGCAAAGCTGTCTGCTCTAAGATCCCTTTCCCTGACATCCATAGAAAAGCCAGAACCAGAAGTACGCTTTGTCATCGGCTCTGCTGGTAGATTCGAGAATCTAGAAAGCTTAAAGTTTGTGTGTAATGAAATGGTAAATTTGCTTGAAAGACCGGAATCGAATGCTACCCAACAGTTAACAGGGCTTACTATCGTGTTTCATGCTTCACGAACAGAACGCAGCCGAAACAAAGATTTCAGGTTTGGTTTGGACAAACTGCTGTCCCTACAGCACGTTCGTGTTGAAATAAACTGTTCCAATGCGACGCAGAGGACGGTGAAAAATGCAGAAGCTGCAATTGGGGAAGAAATATCAAAAATACGCAGGGATGACGGGAGTCAGCCAATTCTGGAAATACGAAGACTCCAGGAAGAAAGTATGATAGCAGGGAAGGACCAAACCCAGGATGCTAATAAGACACCATCTTCGTCTACTATGGAAACTACATTGCGAGCGCAACCTATTGGGGAATCATTAATGCCTAGTGTTGAGACTGTTTCTTCCAAGGTCTTTGTCTCTCAGAACAGCTCCAACGACACATTTTTTAATAATGTAGGTATCCTCACAAGCTCCACGAAAATAAAGACATTGAAAGAGAGAGCGCTTCAGGAACTTACTGCTGAAAAGCAAGGCTCAACTGCTCTCCACCAAGAAGTAGATGAACTCAACAAGAAGTTGGCAAAAACTCAAAGGGAGTTTGAGGAGTTTAAGAAGCAACAGGAAGAGAACAATCTGCTTCTCAAGCATATCTTGCACTCCAACAACGCTGGTGCAGCTTGA

>B97_Zm00018ab440110

ATGGAGCTGGTGGTAGGTGCTTCGGAATCCACCATGAACACTCTCCTGGGCAAGCTGGGCAACCTTCTCGCCCAGGAGTATGCTATGCTCAGGGGCGTCCGCGGTGACATCCAGTACATCAACGACGAGCTCGCCAGCATGAAGGCTTTCCTTCTCGACCTTGCTCGGGACAACCAAGACAACCGGAGGAAGGACTGGATGAAGCAGATCCGTGAGATGGCCTACGACTGCGAGGACTGCATAGATGACTTCGCCCACCGCCTCCCCAAGGATTCATCCAACTTGCTGAACACCAAATGCTGGCCCAGGTGGATGGCTACACTGATCTATGACCTCACGACGTGCTGGCCTCGCCGTGAGATCGCTTGCAACATTGCTGAGCTCAAGGTCCGGGCGCAACTGATTGCCGAACGACGCGTAAGATATGGAGTGAAGGAACCGGACAGCAGCGGAAGCATGCCTGCTGACGCCCCCCCGGAGAACATTGCTGAGGACCAGCTCGCAAGGCGTGATCTCACCATCAAGGAACCCGTAGGGATGGGGCACGTCATGGCGGATCTTGAGAGGTGGGTGAGGCACGACGAGCAGGGCGCAGGCGGCCAGCGAGCTGTCGCGTCCATTGTAGGATTTGGGGGCGTGGGGAAGACCACCGTTGCCATGGCACTGTACAAGAAAGTCATGAAGGATTTTGACTGCCGGGCGTGGGTCACTGTGTCCCAGAACTACGACATAGAAGCCGTCCTCCGTGAGATCCTCAAGCAAATCAACCCAGATTACATGCATCAAGACGGCGGCACTGCTGCAACCATCTCTGAGAAGACCAAGACCAACAGGACAGCATCAGCATCCATTGCTTGCAAATTATTGAAGCGAGCTTTGAACCACCGCACAGGCCACACGCATGGAGGAGGCACTGCTGGAAGCTCTAAGAAAAATGACACCAGCAAAGCTGTAATGGAGCACCTCAAAGGGAAGAGGTACCTCATCTTAGTTGATGACATATGGTCTGCAGAAACGTGGAAGAGGATATCAAAGTCTTTGCCTGACAATGATAAGGGCAGTAGAATAATAGTTACTTCGAGGCTTCGTGCTGTTGGTGCGACATGCTGCCAGAATGGAAAGGATCACCTCTGTGCAGTTGATTTTCTCCGTGACGAAGATGCTGGGCTGTTATTCAGTCGAAGTTTTTCTGAATCGTCTAAGAGCCACAACGAACACATGGTCCCGGAAATTATAGACCTTGAAGATCTATGGAGGAGGTGTGGGGGCCAGCCTTTGGCCATAGTCACCATGGCTGGTCTTGTGGCCTGCAACCTGAACCAACCGATCAGTTACTGGGAGGAACTTGGGAAATTATTGCCAGCGCGAGAGATCTCCAATACTCGCGGGGCTCCAAACCAAGTAAATTCTCTTACTTTGGATGGGGTTAAAAGGATCCTTGATTGTTGCTACAACGATCTGCCTGGAGATGTCAAGACCTGCTTGCTGTACTTGGGCATGTTTCCAAAGGGCTGTAAGATCAGTAGGAAGTGTGTGACCCGGCGATGGATAGCTGAAGGTTTTGTTAGTAGGAGGCATGGGCTGATGGAGGAGGAAGTCGCGGAGACATATTTCAACCAGCTTCTAAGAAGGAAGTTGATCCGTCCTGTGGACCACAGCAGCAACGGGAGGCTAAAAACCTTTCAGGCTCATGACATGGTTCTTGATTATATTGCGTCCAAGGCCAGCGAGGAGAACTTTATCACTGTGGTCGGCGGCCACTGGATGATGCCAGCGCCCAGCGGCAAAGTGCGTCGACTTTCCATGCAGAGCACCAGTCGTCCCAAGGCGGCCGCAGGAGATTATTCAACAACTAGAGGCATGAATTTGTCTCAGGTGCGGTCACTGGCCGCTTTTGGGAGCCTCGCGCAGCTGCCTTTCCAAGCGTTCAACAACAGGATCATGCAAGTCCTAGATTTCCAAGGCCTCAAGGGATTTAAGAACAGACATATGAAACACATCTGTGAAATGCTTGTTCTCAAGTACCTGAGCCTCAGAGGAACAGATATCACCGAAATCCCGTCGAGGATTGGGAAACTTGAGTATCTAGAGACGCTTGACATAAGGGAGACCGATGTCGTGGAGCTGCCAAAAGATGTGAAGCAACTAAAAAGGATCAGCAGCATACTTGGTGGGAACAAGCACAAGAACCCTCGGAAAGGCCTGAGGCTGCCCCAAGAAAAGAGCAGAAAGAAGAAGAGACGACGGACGACGGACGACGACGGGATGATGAAAGCACTCCGTATACTGTCTGGGATCGAGATCAACGAGAATACAGACGTAAGTGGGCTTCACCAGTTGACGGAGCTAAAGAAGCTTACAGTTTACATGCTAAACGTGCTAGACGAATACAGAGCTGACATCTTTGAACAGCTGCTCTCTTCTATCGAGTATCTTTGCAGCTGCGGTCTGCAAACTCTTGCGATCAATGACGAAGATTCTAAATTTACCCAGTCACTGGACAAGATGTCTGCAGCTCCAAGATATCTCGTCGCCCTTGAACTCTCCGGAAAGTTGGTGAAACCCCCAGAATGGATCGATAAGCTACTGACACTGAACAAGCTAACCCTTTCCATCACCATTCTTCGGACTCCTACTTTCGAGAAACTCCGCAGCTTGCCGTTGCTGTATTCTCTAACCTTTTCATTCAGTGCAGTAAAGCAGGATCAGCAGGACGACATCAAGGAAATTCTGGAGGAGAACAAATCAACCACAGATGGTGAGATCTTTGTACCAAAAGGATTTCAGAATCTTAAACTCCTTCGTTTCTTTGCACCACATATTCCAAAGCTGGGGTTTTCTGACAATGCAATGCCAGTACTTGAGACTATTGAGGCGCGGTTTAAAGCCTTCGAAGGTCTGTTTGGCGTTGACACACTAGAAAATCTCAAAGGGGTACATCTCAGAGAAGCTAAAGTGGGGGAAAATGGCAAGGAAATAGCAGACATTAACGAAATCTTGGTACATGATTTGAAAGATAGCACCGAGGGACTCAAGGCAATCGTTGATCACACTTTCATCACTTATTGA

>B97_Zm00018ab459070

ATGACAGGTGTGGAGCCAGCAATTATCGGTGCAATCGCTAACTTGGCGGCCCCGGTCCTGCCTATAGCCATTAAAGGGATACAAGGTGCACTGAAGAAACGGCAAGTCCGTGACAGTGATGTTGAAACCCTGAAATCCCAGCTTAGCTACATCCAGGGCATCATCCGTGATACTCGGAAGACTATCAGGAGTTCCCAAGACCCGTCCGACAGGCTTCAATCCTGGGCTGGATACCTCAGATGCTTGGCGTACGACATCGAAGACCTAATAGAAGGCCGCCGTGCCGGAACCATGACAGGTGCGAAGCTTAATGGCAAGATTACTATCATCCAGGATTTAATCAGATGTGTACAGTCCTATCCGGAGTTTATGGCGGTTCCGACGAATGAGGCTCCTAGTCAAGGCGCTGCTTCTTCCTCCACTACTTCAAGCACCCAGGGCTTTCCGCTGGCTGATCTTGTGGGCAAGAAGGAGGACCTCGATGAGCTTCTGGACCTCCTCGTCCGGAGACCCGACAATGAGCTGGACAAGGTCCTCAAGGTGATGGTGATCTCCGTCGTCGGCTTCGGTGGCATAGGGGAGACCAAGCTTTGCCACACAGTGTACACGGACGTACAGGAGAGCAGAAGGTTCTCCCTGCATGCGTATGTCAGCGCTGCTGGGAAGGACTGCAGCATCGTTCTGGAAGAGATAATCGAGCAATTTAGACTGCAAGAGGATCCACAAGATAGCAGTGGTGGATTTTTTCACAGATTCGCCGGAGCGTTTCCTGGGGCTCGTCGTACTGACCAAGTTCATGGGTTACCCGAGTATCTCCAAAGGAAAAGGTATTTTGTGGTGGTGGATGGCGTGGAGTCTGAAGAACTGGTGAGTGGCATAGCATCTGCCTTCCCGGATAATAGTATGGGCAGTAGAATTATCATGGGTATGAGGACCGCAGTGGGCAGGGATGCAGAGAGATGTGTGGGTCATCATCACAAGATGTGGCCACTTGAAGACAAGCAGTCGGTGGTGTGCTTCCTAAATGAAGCGGAGCGGCGGCGACGACGACATGAACAAGACGACCACTCGTCGTTCATTAGGTTTCAGGAACAAGACCACTCATCATCCTGTTTGCACAAGGTATGTGATGGCGTACCACTTGCGCTGGTTAGCGTATGTGAAGTCCGCAGAGGGTCCATCATCACTGCTGCCGTTGAAGAACAAGACCGCTGGCCACACAGAATGCCCAAGGTGCTCGACCACAGCTACGATGGTCTGCATATCCGGGGTGCTGGGAGCTGTCCGAACCAATACATCCCCTATCTCCAAGCCTGCCTGCTGTACTTTGCCATGTTCCCCCGCGGCAATCATGTCAAGAGGGGATCCCTGATCAGGCGATGGCAGGCGGAAGGCCTAGAGTTCGGAGGCAGCAATCAAGCTGCCGAAAACCTCAAGGCCCTCGTAGACCGGAACTTCGTTTGGCCCCTCCATGCGAGCCTGAATGAGCACGCCAAGACATTCCAGCCTCCTGGAGTGGTGCTCAACTACATCTCCCGCAGGTCTCAAGAGGAGGAATTCATCCTCAGGTCTTGTCCAAGTGGGGAACTTAATCCCAATTACAGCCGCCGGCTTTGTCTACATCCTGCCGAGGAAGAAGAAGGTGAACCCCAACCCGTGGTCATTACCAACGGTTCTGTACCACCACGCCTGCGAACTCTGGCTGTGTCCTGGGGGCAGCAACAGGCCAGCAGGATTGCTGAATGCGAGCAGCTCCGAGTGCTGGATCTGGCGTCATACAATGGTCTACAGCCAGACCAGCTAGAGGAGATATGCAAGAAACTGAAGCTTCTCAAATATCTGAGCCTCCTGCCAGATATCATCACTCAAGTTCCAAGCTCAATGTCTAATTTGCAGTGCTTGGAGACACTCGAGGTGGGGGAGGTCAATGGCAGGGCGGCAGTTCTGGTGCCTATCCAAGTCTTGGAACTGCCACGCATAAAACACCTAATCGGAAAATTTGAGCTTATTGACAACTTCAACGGACTACCAATTAGGGCTTATCCAGATGCACTAGTACCAAAGGCAATCAAGGAAAGCAACCTGGAGACGGTGTCGGGGTTCTTCACCCGCAGAGGCCAAGGATTTCCGCCACTCATGCGTCACATTAGGCAGCTCAGGAAGGTGAAGATATGGTTCTACAGGGATGCAGAACCCAAATACCTAGCAAGCTATCTCCCGAAAGCGATTACAAAATTCCTCAGGAATGACAACGTTCATCGCTCCCTGTCACTTGACTTCCAGGATGGCCCAAGACAAACAGAAATACTGCAGGCTTGTGTGGCTGAAGCTAGCGGTAATCTTTACTCCCTGAAGCTGTCGTCCGGCACAAATCTGAGCAGGATCCAACTGTCGGTTATTGCCACCAACAAGCTGACTGGAATCACAAAGCTATGCCTTTCCCGCTGGAAAACGATAACGTTGGATGCAGAATTTCTGAATGAGCTGACCAAATTGGCCAGTCTGACGTATCTGAAGCTGGATGCAGAAACAATAAAAGGTACGTACGACCAAAACCCAACACCAGGAGGAAATAACCAAGAGAAGGTCGTCATAGAGACTGGGCACATTGCAAATCTGCGGCGGATGTGCCTTGTGGCCAGGCAGACGCTGCCCGACATACAAGTCAAGCCTACAGCTCTGCAACGACTCGTTTCACTTCATCTCATCAGTGAAACGGACGATTATTGTCCTTCCGCCAACGTCATCTGTAAAGCCAACCCCCAGGACGACAACGAGCCGGCGCCGTTCACGAGCCTCCAGGAAGTCTCGCTGAATGCTACGGTACCCGAAAATTTAAGGGATTCTTGGCGTAATGCTGCAAGGGACCATCCAAAGAGGCCACGAATTCTCTTCATCCAACACCCTCACCGTGCGGGATAA

>CML103_Zm00021ab011160

ATGGAGCTACAGCTCTCGGCCGTGCTCGGCTCGCTCGCCCTCGGCGGCGCGGTGCTGGTGCTGTTCTTCGTTAAGTGGTGGCAGCCGCTGGCCGGCACCGACCGGCGCGTCAAGGAGCTTGCAGACGCGGTGGAGGCCCTGCTGCGGCAGCGGTCCGAGGTGCTGGGCCACGACCCGGCGCCGTCGTCGGATACCGTGCGCACGTGGCTGCGGCGCGTGCAGGAGGCGCAGGACGAGATGGCGTCCATCAAGGCGCGGCACGACGGCGAGCAGCTATACGTGGTCCGCCTGGTTCAGTACCTCTTCCTCCCCACGGGCCCGGTCGCGGGGCTGGCCGAGCAGCAGCTCAAGGCAGTGCGCGCGCTCCGAGAGCAGGGCGCCGCGATCCTCGATGCCGCGCTGGCCACGCCGCAGGCGCCGCCGCCTCTTCTCTGCGACCCCGAGGAGCTGGAGGGCCTCCCGGCGGAGGCGGGGCCCGCGAGGGCCTACCTCAACGAGGCGCTGCGCTTCCTCGGCGACTGCGACGCCGCGCTTGGCGTCTGGGGTGCCGGCGGCGTGGGTAAAACCACGGTGCTGAAGCTGGTGCGCGAGGTGTGCGGCCGCGTCGCGCGCTTCGACCACGTCCTACTCGTCGCGGCCTCCAGGGACTGCACGGTGGCCAAGCTCCAGAGGGAGGTCGTGTCCGTGCTCGGGCTGCGCGACGCGCCCACGGAGCAGGCGCAGGCCGCCGGGATCCTGAGCTTCCTGAGGGACAAGAGCTTCCTGCTGCTGTTGGACAGCGTGTGGGAACGTCTGGACCTGGAGAGGGTCGGCATCCCGCAGCCCCTCGGCATGGCTAACGGCAAGGTGAGGAAGATCATAGTGGCGTCGAGGAGCGAGGCCTTGTGCGCCGACATGGGCTGCCGCAACAAGATCAAGATGGAGTGCTTGAACGAGGAGGATGCGTGGAGCCTGTTTCAAGCTAATGTTGGCGGCGACATCATCCATGGCCACGCTCAAATTCCTGCACTTGCTAAACAGGTCGCTGCCGAATGCAAGTGCTTGCCTTTGGCCCTCGTCACCGTCGGCCGCGCAATGTCAAATAAGCGCACACCAGAGGAGTGGTCCAACGCACTCGACACCCTCAAGGCATCGCTCCGCTCCGGCACGCCCGGCTTGGACAAGAGCACGCACGCGCTAGTGAAGTTCTGCTACGACAACCTGGAGAGCGACATGGTGAGGGAATGCCTCCTGACCTGTGCGCTATGGCCGGAGGACCACAACATCTCCAAGGAGGAGCTCGTGCAGAGCTGGATAGGACTCGGCCTGCTCCCCGATCTCGGCGACATCGAAGAGGCCCACAGGTTCGGGCTCTCGGTGATTGCCATCATGATGGCCGCGTGCCTGCTGGAGCCCGGGGACAACCACCGCTACAACATGTTCCCGTCAGACACTCACGTCAGGATGCACGATGTCGTGCGCGACGCGGCGCTCCGGCTCGCGCCCGCCAAGTGGCTGGTCCGCGCAGGCGCTGGGCTCAGGGAGCCCCCGCGCGAGGAGGCGCTGTGGCGGGGCGCGCAGCGCGTGTCCCTGATGCACAACACCATCGAGGACGTGCCGGCGAAGGTGGGTGGCGCCCTAGCGGACGCGCAGCCGGCGTCGCTGATGCTCCAGTGCAACAAGGCCCTGCCGAAGAGGATGCTCCAGGCGATCCAGCATTTCACCAAGCTCACGTACCTGGACCTCGAGGACACCGGCATTCAGGACGCCTTCCCCATGGAGATCTGCTGTTTGGTCAGCTTGAAGCACCTCAACCTATCCAAGAACAAGATCCTGTCGCTGCCGATGGAGCTGGGCAACCTGAGCCAGCTCGAGTACTTCTACCTGCGCGACAACTACTACATCCAGATCACCATACCACCGGGGCTGATCTCGCGGCTTGGGAAGCTGCAGGTGCTGGAGGTTTTCACCGCGAGCATCGTCTCCGTCGCGGACAACTACGTCGCGCCAGTCATTGACGACCTCGAGAGCAGCGGCGCGCGCATGGCGTCGCTCGGCATCTGGCTCGACACCACCCGCGACTTGGAGCGCCTCGCGCGGCTAGCGCCGGGCGTGCGCGCCCGGTCGCTGCACCTGCGCAAGTTAGAAGGGACGCGCGCCCTGCCGCTGCTGTCCGCGGAGCACGCGCCGGAACTTGCCGGCGTGCAGGAGAGCCTGCGGGAGCTGGTGGTCTACTCCTCCGACGTCGAGGAGGTCACGGCCGACGCGCACGTGCCCATGCTGGAGGTCATCAAGTTTGGGTTCCTTACGAAGCTGCGCGTCATGGCGTGGTCCCACGCCGCCGGGTCCAACCTCCGCGAGGTCGCCATGGGCGCGTGCCACAGTCTAACTCACCTGACGTGGGTGCAGAACCTCCCCTGCCTAGAATCGCTGAACCTCAGCGGGTGCAACGGGCTGACGAGACTGCTGGGTGGCGCGGAGGACAGCGGCAGCGCCACGGAGGAGGTGATCGTGTTCCCGCGTCTGAAGCTGCTGGCCCTGCTGGGGCTGCCGAAGCTGGAGGCCGTGCGAGTCGAGGGAGAGTGCGCGTTCCCGGAGCTGCGGCGCCTGCAGACGAGGGGGTGCCCGCGGCTGAAGAGGATTCCTATGCGCCCGGCGCGCGGGCAGCAAGGTACCGTGCGGATCGAGTGCGACAAGCACTGGTGGAACGCTCTACAGTGGGCGGGCGAGGACGTCAAGGCCTGCTTCGTCCCTGTGCTGTGA

>CML103_Zm00021ab015450

ATGGATTGGTTGAGCAGCATGCTGGGGGACCGCCCGCTGAAGAGCGTCTTCACGGCGCTCGGTCTCCCGGATAAGATTGGCGGCGCGGTAATCGACGCTCTCTGCTACCGAGGCGTCCGCCTGTGGAACGTCGAGGAGGAGGCCGACAAGCTGCGGCGCACCAAGGAACGCATCCGCGCCGTGCTCGAGGACGCCGAGCAACGCCGCTTCATCGACCACGACTCTGTCAGGCTCTGGCTCCGGGAGCTTAGGGCCGTCGCTTTCGACGTCGACGCCCTGCTCGACCGCTTGGGAACCATCACGGCCGTGTCCAGGCTAGCGGCCGCCGAGCAGTCACGGAAGCGGAAGCGGCTATGGCCCAGCGTCGAGCTCGGCCCGCGGCAGCGGTGGGAGTTGGATGAGAAGATCGCGAAGATCAACGAACGCCTCGACGAGATCAACACGGGCAGGAAATGGTATAGGTTGCAGGCCGGGGACGGGACGAGGACAGCGTCCCAGCCGACGCAGCGCCCACGGTTCCTTGAATCTGCCGCGCATCGCGACGAGAGGCCCATTGGTCGCAACGAAGAGAAGGAGCAGATTGTCCGTGCTCTGGTTTCGGATAGCGCAGATATGGCGGTGATTTCCATATGGGGAACGACAGGCATCGGGAAGACAGCACTGGCACAATCGGTTTACAAAGATCCTGAGGTACAAAACTTCTTCACCGACAAGATCTGGGTTTGGTTACCAGATAGGTGTGATGTCAGAAAGGCCACCAAAATGATCATCGAAGCGGTGACCAGTCAAAAATGTGAGCTTCTAAGCTTGGACATATTGCAGCAACGGCTGCACGACCACCTACATAAAAAGCAGTTCTTGCTGGTGATTGATAACCTTTGGGCAGAGGGCTTTCAGTTCTGGGAGTTTCTGAGGCCCTCATTGACTAGTGGAGCGGAAGGACGCAAGGTTCTGATCACTACTCAGCATGAAAAGGTGTCTAGGATGATTTCCACCAATCTAAACATCCATTTAAAGGGCTTGGAAGATGAAGAATGCTGGCAAATCCTCAAACTCTATGCGTTCTCGGGATGGGGCAGCAGAGATCAGCATGATCTGGAACCCATTGGGCAGAGCATTGCCTCAAACTGCCAAGGCTCCCCGTTAGCTGCTAAATCTCTTGGGTTACTACTGTCCGACACTCATGGAGACAAAGAACAATGGGAAAGCATACTAGGTGAAATGCAGATTCTCGGAGATGGCGAAAACACAAACAGCATATTACCAAGTTTGCAGATAAGTTACCAGCACTTGTCATATCATCTCAAACAATGCTTTGCCTTCTGTTCAATACTTCCTCCTGGTGTTGAGTTTGAGAAGGATGAGCTCGTCAGACTCTGGATAGCTGATGGTCTTGTTAAGAGTAACGGAAGGGAAAGGGTTGAGATGGAAGCAGGACGATGCTTTGATGAGCTCCTATGGAGATCATTCTTTGAAACTTCCCGCAGCTTCCCTGATCAAAAGTTTAGAGTGCCAAGTTTGATGCTTGAGCTAGCACAGCTTGTTTCTAAACACGAATCTCTGACTCTCAGACCTGAGGATTCACCGGTAGTCGACCATCCCGAGTGGATTCGTTATACAACTATACTGTGCCCGAAAGATGAGCCTCTTGCATTCGACAAGATCTATCGCTATGAAAATTCGAGGCTCTTGAAATTATGCCCGGCAATGAAACTACCTTTGAACCAGGTACCAACGGCACTTTTCTCGAAGTTAACTTGTCTGCGTGCACTAGACCTGAGTTACACTGAGCTAGACGTCCTGCCGGATTCCGTTGGGTCCTGCATACACCTCAGATACCTCAACCTTCGGAATACTCTGATAAAGACTCTTCCAGGAACAGTCTGCGACCTATTCAATTTGCAGACACTTGACCTCAGGGACTGCTACTGGCTCACGGATCTGCCTGCAGGCATGAGCCGCTTAGTTAACCTGCGTCACCTTAGCTTGCATATCGATTGGGATAGAGTTACTGCTCTTAGATCGATGCCGAGTGGCATAGACCGGCTACAGTCACTTCAAACTCTTTCCAGGTTCGTTGTGGTCTCCAGGGATGGAGGCAGGTGCAACATCAACGAGCTGAGGAACTTGAAGATCCGCGGAGAGCTTTGCATTCTTAATCTGGAAGCTGCCACCAGCGACGGATCTGCGCGGGAAGGAGTACCTGCGCGAACTGATGCTGAAGTGGAGCGAGGACACCTGCAAGGACGTGCAGCAGCAGCAGCAGCAGCAGGGCATAGAGAACAGCGAGGCGGTAATCGAGGCACTCTGTCCGCACACCGGCCTCAAGCGTCTGCGCGTCGAGAATTACCCCGGAAGACGGTTTCCTCCCTGCTTCGAGAACCTCCCGTCCCTGGAATCTCTGGAGATAGTCTCCTGCCCCAGGCTCACTCAGTTCTCCGTGCGGATGATGCGGTCTCTCAGGAATCTGAGGATACGCCAGTGTGCTGA

>CML103_Zm00021ab024170

ATGGCAGAGGGTGTTGTTGGCATCCTTATTCTGAAGCTTGGTTCAGCCTTGTTTTTAGAGGCTTGCAGGCTTGGCACAAAACAGCTCTACCATGAAGCTTCAGCCCTTGGCAGGCTCTTTGGTGAGATCCGTGATATCAAGGAGGAATTGGAGAGCATGCAATCTTTTCTACAAGGAGCTGAGAGATTCAAAGATACTGACAACAACACTGCCAACTTTATCAAGAAGATTCGTGGCCTTGCTTTCGACATTGAAGATGTTATCGATGAGTTTATCTACAAGATGGAGGACAAGCATGGCAGTTTTGCTACAAAGATGAATCGTCGGATTAATCGTATTTGGACATGGCGACGTCTCACGTCCAAATTGCAAAAGATCAAACTGAAACTAGAGAATGTTGATAAGAGAAATGTTCGATATGACATGAGAGGAATTGCTAGAGAAGATGGAAGCAGTGATGCTCATCATAGATCTACCGACCAGATTTCTTACTTCCCCAAAGAGGAAAATCTTGTGGGCATTGATGAAAACAAGGAGTTATTGATGAATTGGCTAAGGGGTGATCTACATCAGCAAAGTGTAATTACAACAGTATGGGGGATGGGGGGAGTTGGCAAGACCACTTTGGTTGCACATGTTTACAACACTGTGAAGGTAGACTTTGACAGTGCTGCATGGATAACTGTTTCAAAAGCTTACCAAGTGGAGGACTTGCTGAAGCAGATCATCAGGGGATTTCAGAAAAGTGATTTGAAGGGTGAACTTCGTGTTGACATAATTGACATGGAAAAGAGAAGCCTAGTTGAGATCATCCGTGATTATTTGCATGGCAAAAGCTATGTTTTAGTGCTAGATGACGTCTGGGGTGTTGACATCTGGTTCAAGATAAGAGATGCTTTTCCTACCAATAGCACTAGCAGGTTTATTATTACATCGAGGATACATGAAGTAGCATTGCTGGCTAATGGAAATTGCATAATTGAGTTGAAGCCACTAGAGGCACACCATTCATGGGAGTTATTTTGTAAAGAGGCCTTCTGGAAAAATGAAAACAAAATGTGCCTGCTGGAACTTAATAATTTGGCACAAAGATTTGTTGACAAGTGTAACGGGCTGCCCATTGCCATTGCATGTGTAGGCCGTCTTTTGTCTTGCAGAAGCCCAACCTACTCTGATTGGGAAAGCTTTTTCAAGGAACTAGAGTTGCAGATGACAAATAATGTGATTCTTAATGTTAATGTTCTTCTAAAGGTTAGTTTAGAGGATCTTCCATATATTTTAAAGAACTGCTTTTTGCACTGTACAATATTTCCCGAGGATCATTTGATCAAAAGGAAAAGGTTAATTAGGCACTGGGTAGCAGAGGGATTCATCAGAGAAACAGAGCACAAAACAATGGAGGAAGTGGCAGAGGGCTATTTGTATGAACTTGTCAATCGTAGCCTATTACAAGTAGTGGAGAGAAATGAAAGTGGACGAGTGCAGAGTTGCCGAATGCATGATATTATTCGACTTCTTGCTCTGACAAAAGCAAATGAGGAAGGCTTCTGTAAAGTTTATGATGGCATGGGGAGTTATTCAGCAGAAAAGACACGTCGTTTATCGATTCACAGTGCAAATATTAAGTTGTCGACTCAACCAACAAAGCTTACAGTCCGCTCAATATATGTTTTTAGTAATGGTTTGACTATTGAATCACTTAGGTCTTTCTTGAAACATTTCTACTTGCTGTCAACTCTAGATCTCCAGGGTGCCCAGATTGTGGAGCTGCCAGATGAGGTTTTCAACTTGTTTAATCTACGGTTTCTCAGCCTTCGAAATACTGAGGTTACGAATATCCCCAGCACAGTTGGAAGATTACAAAAACTTGAAGTCTTGGATGTTTATAATGCTAAACTGTTGGCTTTGCCAGAGAGTGTTTCGAAGCTTAGAAAATTGAGATATCTACATGTAGCTACTGTTCCAAAGATAAATACTAAAGGGGTTGTGACCTGGATTGGAATCCAGGTGCCTAAAAGCATCAAATACCTGACAGGCTTGCAAACCTTGAGGCTTGTTGAGGCGAGCTCAGAGACTTTATTTCACCTTGGTGCTTTGACACAGTTGAGAACTTTTGCCATCACAAATGTGCAGAGGGATCAGTGTGCCGATTTGTGCACTGTTATAATGAGCATGAAGCATCTTGTTAGCTTAGCAATTATGGCTATAAGTGAGGAGGAAATACTTCAACTTGAAGAACTTTGTTTACCCCCAACTCTTTCAAAGCTTGAATTAGGAGGGCAGCTGGACAAGAAAGCAATGCCCCAGATTGTATCATCCTTTTCAGATCTTGGTAACCTCACCTTATTGGCCTTGGCATTCTCCAAACTTGATGAGGACTCATTTTCATGCCTCTTGACGTTGCATGGTCTACGTGGGCTTTGGGTTGATAAGGCTTATGAAGGGAAGAGGCTTCACTTTAATGCTATGTCTTTTCCAAATCTTCGACAGCTTGCAATATCAGATGCACCGCAGCTCAACAGCGTTGTAATCGAACGAAGCGCACTGCAAAGCCTTGTTCAGCTGACACTTGTAGATTGTCCAGAACTGAAGGCCCTGCCTGATGGCATTGAGCATCTTAGAACACTTGAGAAATTATATCTGCGAGGAGCATCCAAAGAGCTCACAAAGCTATTTCAGTGCAATGAAGAAACACATGAGTCCAATGGGAATCTTGAGAAGATCGGTCATATCCGAAGGGTTACTGTTTATCCA

>CML103_Zm00021ab030750

ATGGACCGGATGCTGCTCGACCAGCTGGCCGGCGAGGCCCTGCGGGAGGTGCTGCACGCGGTGCAGGGCACCCTGTTCTGCCGCTCCACCGCCGAGCGCCTGCGCCGGAGCGTCGAGCCGCTGCTGCCGCTCGTCCAGGGCCTCGGCCCGCACAGCACCCAGCGCTCCGCGGGGGAGCTCGGCGAGCTCGCGGCGCGGGTCAGGGAGGCGCTCGACCTGGCGCGCCGCGCCGCCGCGTCCCCGCGCTGGAACGTCTACCGCGCCGCGCAGCTGTCGCGCCGGATGGAGGCGGCCGACCGCGGCATCGCGCGCTGGCTGGAGCGCCACGCCCCCGCGCACGTCATCGGCGGCGTGCGCAGGCTCCGCGACGAGGCCGACGCGCGCATCGGTCGCCTCGAGCGCCGCGTCGAGGAGATCGCCGCCGCCACCGCGCAGCCGCCGCCCCCCGCCCTCTCCGTCCCCGTCGCGCCGCCGCCGCACAAGGGCGTGCCCATGCCGATGGAGGCGCCGCTCGCTAAGCCCGCCTTCGTCGCTATGGCGAAGGAGGTGCCGCAGCACAAGGGCATGGCTATGTCGGAGCCGGTGCCGGTGAAGGCGGCGCCCGCCAAAGCCGGGGTGATGGCCATGGACATCGCCGACGGACACGAAGACGCGGAGGGGATGGTTGGCGGCTGCGTCAAGGTGGCCAAGGAAAAGGTGAAGGAGATGGTTATGAGCGGCGGCGGCAGCTGGGAGGTGGTCGGGATCTCCGGCATGGGCGGCAGCGGCAAGACCACGCTCGCCATGGAGGTCTTCAGGGATCACAAGGTCCGAGCCTACTTCAACGACAGGATCTTCTTCGAGACGATCTCGCAGTCCGCGAATCTGGAGGCCATCAAGATGAAGCTGTGGGAGCAGATCAGCGGCAACATGGTGCTGGGTACATACAACCAGATCCCAGAATGGCAGCTCAGGCTAGGACCAAGGGACCGAGGACCCGTCCTTGTGATCCTCGACGATGTTTGGTCTCTCCCGCAGCTTGAGGAGCTCATCTTCAAGTTCCCTGGGTGCAAGACCCTAGTCGTATCAAGGTTCAAGTTCCCCACGCTGGTGAAACAGACGTATGAGATGCAGCTGCTAGACGAGGCGGCAGCTCTGTCCGTCTTCTGCCGCGCTGCGTTCGACCAGGAGTGTGTTCCGCAGACCGCCGACAAGAGATTGGTCAGGCAGGTCTCTGCAGAGTGCAGAGGTCTCCCTCTGGCTCTGAAGGTCATCGGCGCGTCGCTGCGCGACCAGCCTCCGAAGATTTGGCTCAGCGCCAAAAACCGGTTGTCTCGAGGAGAGGCCATTTCTGACTGCCATGAGACCAAGCTTCTGGAGAGGATGGCGGCCAGTGTCGAGTGCTTGTCCGAGAAGGTTAGGGACTGTTTCCTTGACCTGGGCTGCTTCCCGGAGGACAAGAAGATCCCCCTCGACGTCTTGATCAACATCTGGATGGAGATCCATGACCTTGATGAGCCAGATGCTTTTGCCATCTTGGTTGAGCTTTCGAACAAGAACCTTCTTACCCTCGTTAACGATGCACAGAACAAGGCTGGAGATCTGTACAGTAGCTACCATGACTACTCGGTGACACAGCACGACGTGTTGAGAGATCTTGCTCTTCACATGAGCGGGCGTGACCCGCTCAACAAGCGCAGGCGGTTGGTGATGCCGAGAAGGGAAGAAACACTTCCGAGGGATTGGCAGAGGAACAAGGATGCTCCATTTGAAGCTCAGATAGTCTCCATTCATACAGGCGAAATGAAAGAATCCGACTGGTTCCAGATGAGCTTCCCCAAGGCAGAAGTGCTGATCCTCAACTTCGCGTCGAGCCTGTACTACCTGCCGCCGTTCATCGCGACGATGCAGAACCTGAAGGCCCTGGTGCTGATCAACTACGGCAGCGGCAGCGCGGCCCTGGACAACCTCTCCGCCTTCACCACGCTGAGCGGGCTGAGGAGCCTGTGGCTGGAGAAGATCATGCTGCCGCCGCTGCCCAAGACGACGATCCCGCTGAGGAACCTGCACATGATCTCGCTGGTCCTCTGCGAGCTGAACAGCAGCCTGAGGGGGTCGACGATGGACCTGTCGACGACGTTCCCGCGCCTGTCCAACCTGACGATCGACCACTGCATAGACCTCAAGGAGCTGCCGCCGAGCGTCTGCGAGATCGGGTCCTTGGAGACCATCTCCATCTCCAACTGCCACGACCTCACCGAGCTGCCGTACGAGCTGGGGCGGCTGCGCTGCCTCAGCATCCTCCGCGTGTACGCCTGCCCGGCGCTGTGGCGGCTGCCGGCGTCGGTGTGCAGCCTGAAGCGGCTCAAGTACCTGGACATCTCACAGTGCATCAACCTGACGGACCTCCCCGAGGAGCTCGGCCACCTGACGAGCCTGGAGAAGATCGACATGCGCGAGTGCTCGCGCCTCAGGAGCCTCCCCAGGTCGTCGTCCTCGCTCAAGTCCCTCGGCCATGTCGTGTGCGACGAGGAGACGGCGCTGCTGTGGCGTGAGGCCGAGCAGGTCATCCCTGACCTCCGCGTGCAGGTGGCCGAGGAGTGCTACAACCTGGACTGGCTCGCGGACTGA

>CML103_Zm00021ab033500

ATGGCGGACGCGGGGGTGACGGGGGTACTGGCCAAGCTGGGTGAGCTGGCGGCGGAGGAGGCGACGGCGCTGCTGCGCGTGGACGCCGAGATCCGGGCGTTGCGGCGGAAGCTGGCCTACCTGCAGGCGCTCGTACGCGGGGCAGACCGCCAGCGCCGCGGCCGCGCAAGTGAGCTGCTCCTGCTCTGGCTGCGCGAGACCAGAGAGGTTGCTTTCGAGGTTGAGGACGCCGTCGATGAGTTCCACCTCCGCGTCGAGGCCTGCCGCCCCGGGGCCCGGTGGCGCCGGCGCCGCAGATGGTGGTGGGGCTGGCACCGCGACGCCGTCAGCCTCGTCCAGGACCTCGCCACGCAGTTTTTTGTACGTCATGGGCTGTCAAATCAAATATCTAAGATCAATGAAAGGATTGATGAGCTTAACCAGAACAAGGAAACATATCAAATTGAAAGTTGTCCTTCTGAAATTTGGAGTTCTTCATCGGTTGAAATGGATCCTGAGTGGTACGAAGATAAATATGTTATAGGCTCTAGAGAACGTGAATTTGCTATCCTTAAGGACCTAATCATCAACAAAGAGGGAGATATGTCTCACCGGGCTGTCATCTCTATTTTTGGGGAGCGTGGCATTGGAAAGACTACACTTGCAAAACAACTGTACAATGACCCAGATATCATAAAACACTTTGAGGTCCATGCATGGGTATGTCTTCCACCACATGTCAGGTTCAGGGACTATGTTGAGATTATGCACATGCAGGTCAACCCACAGATTCCAGGAGCTTCTGAGAAAAATGGTAATACAACATTTGCACTTGGTAACAAAGAAACCACTGATATGGAATTCAACCTTCGGCAGAACCTTGAGAACAGGAGGTATCTAGTTGTTCTTGATGGTTTAGTCAGCATTAGTGACTGGAACTCATTATTTGCTGTGCTGCCACATACCAATGCCAATGGCAGCCGGATCTTACTTACCACACATCTCAATGTGAAGGAAATCAATCACATCGACCCACAGATAGCTCCTGTCAAGCTTCCTTATCTTGACGAAAAACATGGAGAGGAGTTATTTTGTCAAAGAGTTTTTGGGACAATAGAACCTCCACAAATTTATAAGAGCAAGGGTTACTATAAAAAAGTTCACAATATATCAACAGGTCTACCCCTGGCAATTACTGTGCTTGCAGGAATATTACGATCAAAGTTTATCCCCATGGAGTGGGATGTCATATTCGAACAACTCGAGTCCAATGGCCAGCCAAAACCGGTTAGAAGCATATGGTCTTTGGCTTTTGATGACTTGCCACACTACCTCAAGTCATGTTTCCTATACTTGGCGTCCGTTTCAGAAAATGTTATTCTTTACCCAGATCATTTGGTGCGTCTGTGGATTGCTGAAGGTTTTGTTATGCCCAAGAAAGCAGAAACACTGGAGGACGTTGGGTTTGACTATCTAAAAGAACTGGTCGCGAGAGGGTTAGTCCAGGTCGTGCAGAAGGATGCTGGTGGATCCATCAAGCTGGTAGCCATCCACAATCTGCTCCATGCTTTTGTGGAGTCTGAAGCACAGGACTCTAGTTTCCTTGAAATCCACCATCATGCTAATGTTGTAAACCCAAATGCAGTGCGGCGCCTTGCCATACAAAACTATGTGGATGCATATGTCCACATTCCTAATGTGTTCCCCAAGCTGCGTTCGCTTCTCTGTGATTTTGCAGAAGACCAACAACGCGGTAGCTCAAGCTCTGGAGAGCTGCAACCTCAGTCGTTATGGGGCAATCTTGCAGAGTTGTGTTCAAGAGCCTGTGGCACTTCGGAGAATGTTGGCTCAAGCACATTGCATGGGCTCCACTTCTTACAGGGCTCCAGGTACCTACGAGTTGTTGACCTGTATGGTCTTAAGACGCGAAAGCTGCCAGATGAGATCGGCAGCATAATCCACTTGAGGTACCTTGGCATTAGGAACAGCAACTGT

>CML103_Zm00021ab077930

ATGACTTTTCAAGCCGCAAGCCCACCGAACAACGTGCGCGCGGACGTTTTCTTGGCAAGGCCCGAATCCTTCCAATCCAATCCCCATCCGGGCAACCGTCTGCCGTGTTGCCCCATTCCTGCCATGCACCGCGTGGCGCCTAGCCTCCGCATATGCTTCCTTTATAAGCTTCAAACAACAACTCCCTTTTGCAGATTCCTCGTTCGCCTGCTGCACGCTCACCACTGTACGTCCCCGATGGCTGAAGCCATCAGCGCGACCAGCTCGTGCCTGGAACCCCTGTGCGGTTGTCTGGAAAGCACAGGCGTGTTCGAGGCAGTGGGCCGGGAGGTGGCCGCGTTCCTTCGCATCAAGTCGAATTGGGGCGACCTCGAAAAGGCCCGGGACAGCCTGCGCGCCGTCGAGACGACAGTCAGGGCGCGGGTCACGGCGGAGGAGGACAAGCTGAACGTCTGTGATCCTCAGGTGCAGGCGTGGCTCAAGCGCGTCGACGAGCTTCGCCTGGACACCATCGACGAGGATTACAGCAGTCTGTCGGGTTTCTCTTTCCTCTGCCAGTGCACCGTGCACGCTCGTCGCCGCGCCTCGATCGGCAAGCGTGTTGTGGACGCGCTGGAGGAGGTGAACAAACTGACCGAGGAAGGAAGGCGGTTCAGGACATTTGGGTTCAAGCCACCGCCGAGGGCCGTCAGTCAGTTGCCCCAAACTGAGACCGTTGGGTTGGAGCCCATGTTGGCTCGGGTCCATGATTTGCTTGAGAAGGGCGAGTCGAGCATAATTGGTGTGTGGGGTCAAGGAGGCGTCGGCAAGACGACTCTCCTGCATGCCTTCAACAATGATCTCGAAATGAAAGACCACCACTACCAGGTTGTTATTTTTATTGAAGTATCCAATTCAGAGACCCTGAACACAGTGGAGATGCAGCAGACTATCTCCGATAGGCTTAATTTGCCATGGAATGAATCAGAGACAGTTGAGAAACGGGCCAGATTCCTATTGAAGGCACTGGCCAGGAAAAGATTTCTATTGTTACTTGATGACGTAAGGAAGAGATTCCGACTGGAGGATGTCGGTATCCCAACTCCGGACACGAAGAGCCAAAGCAAGCTGATCCTGACATCACGTTTCCAAGAAGTATGCTTCCAGATGGGTGCACAGAGGAGCCGCATTGAAATGAAGGTTTTGGATGATAATGCTGCCTGGAACCTGTTCTTGAGCAAGCTGAGCAACGAGGCTTTTGCAGCAGTTGAGTCACCGAATTTCAACAAGGTTGTTCGGGACCAGGCCAGGAAAATATTCTCCAGTTGTGGAGGTCTACCACTTGCACTCAATGTCATTGGGACTGCTGTGGCAGGGTTGGAAGGACCAAGAGAATGGATTTCAGCTGCTAATGACATCAATATGTTCAACAATGAAGATGTGGATGAAATGTTTTATCGGCTGAAATACAGTTATGACAGGCTGAAACCCACTCAACAACAGTGTTTTTTGTACTGCACTCTTTTCCCAGAATATGGATCTATTAGTAAGGAACCATTAGTTGATTATTGGCTGGCTGAAGGTTTGCTTCTCAGTGATCGTCAAAAGGGTGATCAGATAATTCAGAGCCTTATTTCAGCATGCTTGTTGCAGACCGGTAGCTCATTGTCATCAAAGGTAAAAATGCACCATGTAATCAGGCATATGGGGATTTGGTTGGTTAACAAGACAGATCAAAAGTTTCTCGTTCAAGCAGGGATGGCTTTGGATAGTGCTCCACCAGCAGAAGAGTGGAAGGAATCGACAAGGATCTCCATCATGTCTAATGATATCAAAGAGCTTCCTTTCTCACCGGAATGTGAAAACCTCACTACATTGTTGATCCAAAATAACCCAAATTTGAACAAGCTGAGTTCAGGGTTTTTCAAGTTTATGCCCTCCTTGAAAGTGCTGGATCTTTCTCACACTGCAATAACGACACTCCCAGAATGTGAGACATTGGTTGCATTACAGCATCTCAATTTGTCACACACACGTATTAGGTTATTACCTGAGCGGCTGTGGTTATTGAAAGAGTTGAGGCATCTGGATCTCAGCGTGACTGCTGAACTCGAAGATACCTTGAACAACTGCTCAAGGTTACTCAATTTAAGAGTTCTTAATCTCTTTCGCAGTCACTATGGTATTAGTGACGTCAACGACCTGAATCTGGATTCCCTGAAGGCACTGATGTTCCTTGGAATCACTATTTATACAGAGAAGGTGTTAAAGAAACTGAACAAGACTAGTCCTTTGGCAAAGTCAACATATCGTCTGCATCTGAAGTACTGTAGAGAAATGCAGTCGATCAAAATCTCCGATCTCGACCACTTGGTGCAACTCGAGGAGCTGTATGTCGAATCATGCTATAATCTAAACACTCTTGTTGCTGATACCGAGCTGACTGCATCAGATTCAGGCCTGCAGCTCCTCACCCTCTCAGTTCTTCCTGTGCTGGAGAACGTCATTGTTGCACCAACGCCACACCATTTTCAGCACATCCGCAAATTGACCATTTCGAGTTGCCCCAAGTTGAAGAACATCACATGGGTCCTAAAACTTGAAATGCTCGAGAGGCTCGTCGTAACCCATTGTGATGGGTTGCTGAAGATTGTTGAAGAAGACAGCGGTGATGAGGCAGAGACAACAATGCTGGGTCAGGGTCATCCTTCTGAAGAACAGGAAGATAAACGGATTGATGGTGGTCAAAGTGTGTGCAAGAGCGATGACAATGCGCATGCTGAGCTCCTGAACCTGAGATCAATCGTGCTGACTGATGTCAAGAGCCTGAGAAGTATCTGCAAGCCAAGAAATTTTCCCAGCCTCGAGACCATCCGGGTGGAGGATTGCCCGAATCTGAGAAGCATCCCACTGAGCAGCACGTACAACTGTGGGAAACTGAAGCAGGTGTGCGGTTCAGTTGAATGGTGGGAAAAACTGGAGTGGGAGGACAAGGAGGGCAAGGAGAGCAAGTTCTATATTCCAATCTGA

>CML103_Zm00021ab089950

ATGGATCTTGTGGCCGGCGCCGTGGGCAGCATCATCCGCAAGCTCGGCGAGCTGCTCCTGGCAGAGTACCAGCTGCAGGCGTGCCTGCCGGAGGAAATCGAGTCTCTGAAAAATGAGCTCGAGAGCGCGCACGTGGCTCTCCGCACCGTGGCGGAGGTGCCGCCGGAGCATCTTGATCAACAGGTCCAGCTCTGGGCTCGCGAGGTCAGGGAGGCGTCGTACGACATGGAGGATATCCTCGATACCTTCCTCGTCAATGACGCACCGGCTGAGAAAAAGGATGGCCTCGGCAAACGTCGTCGTCTTCGTCGTCTCCTGGACAATATAGCTGGCGCCATGGAGAAGATGAGGAAACTGTTCAGAAAGAGCAAGGAGTGTCACACCATAGCTGGCGCCATAGAGAAGATGAAGGAACGGCTCCGGGAGGTGGCTGACCGCAGCGACAGGTACGCCGTTCCGGTGGCAGCGCCTGCGCCGGCGAGGACGCTGGATCCTCGCCTTGCATACATGCACAGGGAAGCGGCACAGCTGGTCGGCATGGACAGGACCAAGGCTGAGGTCATGGCCATGCTTCTGCCGCTGCCGTCGTCCCGCTGCCCCGAGGACGACATCGACGTCTCTGCCAGCGGCGGAGACAAGATGAAGATAGTTTCTGTGGTCGGAGCTGGTGGCCTGGGAAAGACCACTCTTGCCAAGGCCGTCTACGACGAGCTCAAACCGCGATATGACTATGGAGCGTTTGTTTCGGTTGGCCGAAAACCTGATCTGGTGCAAGTGTTTACCAACATCTTCTTCCTTCTCGACAGACAGGAGCACGCGGCCATTCGTGAAGTAAAGGACCTACAGCTGTTGGCCGACGGACTACGAACATTTCTACAACACAAGAGGTACTTGATCGTTATCGACGATGTTTGGGATACAGAATCTTGGGAAACAATCAAATTAGCTTTTGATCAGAAGAATAAGAAGAGTAGGGTAATCACAACCACTCGCAACCGACAAGTAGCTTCCGGCGAGAAGGTTTACGAGCTACATCCGCTCCCTCATGACAGCTCGAAGAAGCTATTTTATATGAGGCTGTTTGGGGGTGAGGACAAATGTCCGGCTAATCATCCTGAAGAGGCGTCTCAAAGGATTCTTAACAAATGTGGCGGTGTACCATTGGCTATCATCACAATGGCAAGCTTGCTGGTGGGTAAATCGAGAGAAGACTGGTTGGAGGTGTGCAGCTCTCGCGGTTTCTACCACGGCGGCGGTAAAGATAACAACAAACAAGTAGATGACACCGTGTGGATACTGTCTCTGAGCTATTATGACCTACCTTCATATCTGAAGCCTTGCTTACTGTACCTAAGTGTGTATCCAGAAGACTATGAGGTCGAGAGGGAGAGATTGATATGGAAGTGGGTAGCTGAAGGTTTCATCGAGAAGAAAGCAGGAAGCAGCAGCAGCCTGTTTGAGCAGGGAGAGGAATACTTCCATGAGCTCATAAACAGAAGCATGATCCAGGCGGTGGGGGACGACAAAGAGGTTGCTGACGCCATATTTGATTGTCGTGTTCATGACATGGTGCTTGATCTCATCCGTGACATATCAAACGAAGAAAACTTCATCACTGTCTCATACGATGATGGTAGAAGAGGCGCACGGTCGTCGTCGTCGTCGTCGTCGTCGTCACGGCACGTGGTGCGCCGGTTAGCTCACCAAAACAGAAGAATAACGGAGGAGGACAACCCTGTGGGGGGCAGCATGAGGTCACTTGTTGCCTGTGGGTGTGATATGGATGGTTGGGCCTTGCACTCGAGCTCTAATAAGCTGCTGCGTGTGCTAGCTTTAGAGGAATGCACGCCATCTATGGACATCGGACATCTTGGAAAACTGCTTCATTTGAGGTACCTTGGGTTACATGGTACTAGCATCAAGACACTCCCAGAGGAAATAGGATCCCTCAAGTTTCTGCAAGCACTGGATTTAGAGGGCACTGAAATATCACGTCTTTCACAGACTGTTTGCCTGCTAACACAGCTGATGTACCTACGGGGTGCAACCGGGGTCACAACAGTGCCTGATGGTTTCCTGGGGCAGGTGACGTCACTAGAGGAGCTCCATATATGTCTTCCCACCAAAGATGACGAGTACAGCCAGAAGAAGTTCATGCAGGATCTGGGCAAGCAGGGAGAAATCAGGATTCTCGTTTTGTATGGGAACAGAATTGAGTTGGATCCGTGGATGCAGTCCAGTCTAGTGCAATCACTAGGCGGTCTGTACAAGCTCCAGACCCTAGTGGTGAGGCATTATGCAGATGGGGTAGCAGCAGCACAGGGCAGCTGGGACACGGCGAAGCTTCGGCGACGTCTCCGGATTTTGAACTTAGATGTCCTCCGGTTCCATCGTGTACCATCGTGCATCGATCCCGCGCGCCTCCCCAACCTCTCACACCTACAACTGCTTGTGGTTCATCTGGACGAGGCAGGTCTGAGAGCACTGGGCGGCCTGCCAGAGCTCACCTACCTCGCGCTGTCGTTGAAGCCTCGTTCGCTGAACAGCACATCATGCAAGGCTACGGTAGCTGATGTTGTTGCCGCAGATGGCTTCTTCCTCAAGTTGAGATCGCTCAAGCTGTATGGCTGGATGGTTCAGTTGGTGCCCAGCCAGGACTCGGCAAGTGTTTCGTTCAGCATCTGGAATGAAGGAGCGGAGGTTGTGGCCCTTGGTTCCACGAGAGACTGCACCGCCGGGAGGGTAGCACCTGCTATCATGCCAGACCTCATACATCCTGACATCCAGACCTGCTATCACGAGAGACTGCACTACCGGAATCAGCTTCTTTGCCGTGTGTTCTAA

>CML103_Zm00021ab089960

ATGGATCTTGTGGCCGGCGCCGTGGGCAGCATCATCCGCAAGCTCGGCGAGCTGCTCCTGGCAGAGTACCAGCTGCAGGCGTGCCTGCCGGAGGAAATCGAGTCTCTGAAAAATGAGCTCGAGAGCGCGCACGTGGCTCTCCGCACCGTGGCGGAGGTGCCGCCGGAGCATCTTGATCAACAGGTCCAGCTCTGGGCTCGCGAGGTCAGGGAGGCGTCGTACGACATGGAGGATATCCTCGATACCTTCCTCGTCAATGACGCACCGGCTGAGAAAAAGGATGGCCTCGGCAAACGTCGTCGTCTTCGTCGTCTCCTGGACAATATAGCTGGCGCCATGGAGAAGATGAGGAAACTGTTCAGAAAGAGCAAGGAGTGTCACACCATAGCTGGCGCCATAGAGAAGATGAAGGAACGGCTCCGGGAGGTGGCTGACCGCCGCGACAGGTACGCCGTTCCGGTGGCAGCGCCTGCGCCGGCGAGGACGCTGGATCCTCGCCTTGCATACATGCACAGGGAAGCGGCACAGCTGGTCGGCATGGACAGGACCAAGGCTGAGGTCATGGCCATGCTTCTGCCGCTGCCGTCGTCCCGCTGCCCCGAGGACGACATCGACGTCTCTGCCAGCGGCGGAGACAAGATGAAGATAGTTTCTGTGGTCGGAGCTGGTGGCCTGGGAAAGACCACTCTTGCCAAGGCCGTCTACGACGAGCTCAAACCGCGATATGACTATGGAGCGTTTGTTTCGGTTGGCCGAAAACCTGATCTGGTGCAAGTGTTTACCAACATCTTCTTCCTTCTCGACAGACAGGAGCACGCGGCCATTCGTGAAGTAAAGGACCTACAGCTGTTGGCCGACGGACTACGAACATTTCTACAACACAAGAGGTACTTGATCGTTATCGACGATGTTTGGGATACAGAATCTTGGGAAACAATCAAATTAGCTTTTGATCAGAAGAATAAGAAGAGTAGGGTAATCACAACCACTCGCAACCGACAAGTAGCTTCCGGCGAGAAGGTTTACGAGCTACATCCGCTCCCTCATGACAGCTCGAAGAAGCTATTTTATATGAGGCTGTTTGGGGGTGAGGACAAATGTCCGGCTAATCATCCTGAAGAGGCGTCTCAAAGGATTCTTAACAAATGTGGCGGTGTACCATTGGCTATCATCACAATGGCAAGCTTGCTGGTGGGTAAATCGAGAGAAGACTGGTTGGAGGTGTGCAGCTCTCGCGGTTTCTACCACGGCGGCGGTAAAGATAACAACAAACAAGTAGATGACACCGTGTGGATACTGTCTCTGAGCTATTATGACCTACCTTCATATCTGAAGCCTTGCTTACTGTACCTAAGTGTGTATCCAGAAGACTATGAGGTCGAGAGGGAGAGATTGATATGGAAGTGGGTAGCTGAAGGTTTCATCGAGAAGAAAGCAGGAAGCAGCAGCAGCCTGTTTGAGCAGGGAGAGGAATACTTCCATGAGCTCATAAACAGAAGCATGATCCAGGCGGTGGGGGACGACAAAGAGGTTGCTGACGCCATATTTGATTGTCGTGTTCATGACATGGTGCTTGATCTCATCCGTGACATATCAAACGAAGAAAACTTCATCACTGTCTCATACGATGATGGTAGAAGAGGCGCACGGTCGTCGTCGTCGTCGTCGTCGTCGTCACGGCACGTGGTGCGCCGGTTAGCTCACCAAAACAGAAGAATAACGGAGGAGGACAACCCTGTGGGGGGCAGCATGAGGTCACTTGTTGCCTGTGGGTGTGATATGGATGGTTGGGCCTTGCACTCGAGCTCTAATAAGCTGCTGCGTGTGCTAGCTTTAGAGGAATGCACGCCATCTATGGACATCGGACATCTTGGAAAACTGCTTCATTTGAGGTACCTTGGGTTACATGGTACTAGCATCAAGACACTCCCAGAGGAAATAGGATCCCTCAAGTTTCTGCAAGCACTGGATTTAGAGGGCACTGAAATATCACGTCTTTCACAGACTGTTTGCCTGCTAACACAGCTGATGTACCTACGGGGTGCAACCGGGGTCACAACAGTGCCTGATGGTTTCCTGGGGCAGGTGACGTCACTAGAGGAGCTCCATATATGTCTTCCCACCAAAGATGACGAGTACAGCCAGAAGAAGTTCATGCAGGATCTGGGCAAGCAGGGAGAAATCAGGATTCTCGTTTTGTATGGGAACAGAATTGAGTTGGATCCGTGGATGCAGTCCAGTCTAGTGCAATCACTAGGCGGTCTGTACAAGCTCCAGACCCTAGTGGTGAGGCATTATGCAGATGGGGTAGCAGCAGCACAGGGCAGCTGGGACACGGCGAAGCTTCGGCGACGTCTCCGGATTTTGAACTTAGATGTCCTCCGGTTCCATCGTGTACCATCGTGCATCGATCCCGCGCGCCTCCCCAACCTCTCACACCTACAACTGCTTGTGGTTCATCTGGACGAGGCAGGTCTGATAGCACTGGGCGGCCTGCCAGAGCTCACCTACCTCGCGCTGTCGTTGAAGCCTCGTTCGCTGAACAGCACATCATGCAAGGCTACGGTAGCTGATGTTGTTGCCGCAGATGGCTTCTTCCTCAAGTTGAGATCGCTCAAGCTGTATGGCTGGATGGTTCAGTTGGTGCCCAGCCAGGACTCGGCAAGTGTTTCGTTCAGCATCTGGAATGAAGGAGCGGAGGTTGTGGCCCTTGGTTCCACGAGAGACTGCACCGCCGGGAGGGTAGCACCTGCTATCATGCCAGACCTCATACATCCTGACATCCAGACCTGCTATCACGAGAGACTGCACTACCGGAATCAGCTTCTTTGCCGTGTGTTCTAA

>CML103_Zm00021ab090050

ATGGATCTTGTGGCCGGCGCGGTGGGCAGCATCATCCGCAAGCTCGGCATGCTGCTCCAGGCGGAGTACAAGCTGCAGGCTGGCGTGCCGGAGCAAATCGAGTCTCTGAAAAATGAGCTCGAGAGCGCGCACGCTGCTCTCCGCAACGTGGCGGAGGTGCCGCCGGAGCAGCTTAATCCACAGGTCCGGCTCTGGGCTCGCGAGGTCAGGGAGGCGTCGTACGACATGGAGGACATCCTCGACACCTTCCTCGTCAACGCCGCCCCGGCTGATGGCCTGGGCAAACGTCGTCTCCTGGAGAAGATAGGGAAGTTGTTCAGAAAGAGCAAGGCGCGCCACGATGTCGCTGGCGCCATGGAGAAGATGAAGGGACGGCTCCAGGAGGTAGCTGACCGCCGCGATCGGTACGCCGTTCCGGTGGCAGCGCCAGCGCCTGCGAGAACGCTGGATCCTCGGCTCGTGTTCATGCATAGGGAAGCGGCGCAGCTGGTGGGCATCGACAAGACAAAGGCTGAGCTGATGGCCATGCTTCTGCCGCTACCACCATGGTCACGCTACGGCACAGATACCGAGGACGACGTGGATGTCTCTGCCAGCGGCGGTGACAAGATGACGATAGTTTCTGTGGTCGGAGCTGGTGGCCTGGGAAAGACCACTCTTGCCAAGGCCGTCTACGACGAGCTCAAACCGCGATATGACTATGGAGCGTTTGTTTCGGTTGGCCGAAAACCTGATCTGGTGCAAGTGTTTACCAACATCTTCTTCCTTCTCGACAGACAGGAGCACGCGGCCATTCGTGAAGTAAAAAACCTACAGTTGTTGACTGGCGAGCTACGAAAATTTCTACAAGACAAGAGGTACTTGATCGTTATCGACGATGTTTGGGATATAAAATCTTGGGAAATAATCAAATCAGCTTTCGATGAAAAGAATAAGGAAGGAAGGGTAATCGCAACCACTCGCAACCGACAATTAGCTTCCAGCGAGGAGGAGGTTTACGAGCTACATCCGCTTTCGCATGACAGCTCAAAGAAGCTATTTTATATGAGGCTGTTTCGGGGCGAAGACAAATGCCCGGCTAATCATCCTGAAGAAGCATCTAAAAGGATTCTGGACAAATGTGGTGGTGTGCCATTAGCTATCATCACAATGGCGAGCTTGCTGGTGGGTAAATCGAGAGAAGATTGGTTGGAGGTGTGCAACTCTCCCGGTTTCTATCGCGGTGGTAAAGATAACAAGCAAGTAGATGACACCGTGTGGATACTGTCTCTGAGCTATTATGACCTACCTTCTCATCTGAAGACCTGCTTACTGTACCTAAGTGTGTATCCAGAAGACTATGTGGTCCAGAAGCACTGCCTGATATGGAAGTGGGTGGCTGAAGGTTTCATCGAGAGGAAAGCAGGAAGCAGCAGCTTGTTTGAGCAGGGAGAGGAATACTTCCATGAGCTCATAAACAGAAGCATGATCCAGGCGGTGGAGTTCAAAGAGTCGTCTGGCATCATATATGGTTGTCGTGTTCATGACATGGTGCTTGATCTCATCTGTGACATATCAAAGGAAGAAGACTTGGTCACTGTCTCATCATACGATGATGGTGATGGACGAGGCACACCGTCGTCACGAAGCGTGGTGCGCCGCTTAGCTCACCAGAACCGAAGAATAACCAAGGAGACGACGACTCACCAGGACAGTCGCAGTCCTACGGAGGAGGGCGCTAAGCTGAGGTCACTGGTTGCCTGTGGGTGTGATATCGATGGTTGGGTCTTGCACCCGAGCTCTAACAAGCTACTTCGTGTGCTAGCTCTAGAGGAATGCACACCATCTACGGACATGAGAAATCTGCTTCTGTTGGAAGATTCAGAGATAGAAAGGTTTACTAAGAGTTGGGACGGTCTTGGCCGCGATCTCGGAAAGCTGGTCCATCTGAGATACCTTGGGCTACGCGGTACTCGCATCAATAAGCTCCCAGAGGAAATAGGGTCCCTGAAGTTTCTGCAAGCACTGGATTTAGTAGGCACTGGAATATCACGGCTTCCACGGACCGTCTGCCTGCTAACGCAGCTGGTGTACCTGCTGGGTGACAGAGGCACGACAGTGCCTGATGGTTTCCTCGGGAAGGTGACGTCACTGGAGGAGCTGCATATACATCCTCCTACCGAAGATGAAGAGTACAGCCAGCAGTTCATGCAGGATCTGGGCAACAACCAGGGAGAAATCAGGGTGCTCAGTTTGAGGGGGGTCGTAGATGGGTTCGATGAGAGCATGCAGTCCGGTCTAGTGCAGGCACTAGGCGATCTACACAAGCTCCAGACCCTAGTGTTGAGCGGTGACGACATGTATATGGACAAAGCCGCACAGGACAGCTGGGATAGGGCGGCGCTTCCCCGGCGTCTCCGCATTTTGTTCTTCGAAAACCTCTGGTTCGATTGTCTACCATCGTGCATCGATCCCGCGCGCCTCCCCAACCTCTCACACCTAAACCTGCTTTTGAGTCATCTGGACGAGGCAGGTCTGAGAGCCCTGGGCGGGTTGCCGGAACTCACCTACCTTCAACTGTCATGGACACGTGGTTCGCTGAAGAGATCTACGGTAGCTGATGTCGTCGTCGTCGTTGCCGCGGATGGCGGCTTCTTCCTCAAGCTGAGATTCCTCAGGCTGGATGGCTGGATGCTCAAGTTGGCGCCCAGCGACGAGGACTCGGCCAGTGTTTCGTTCAGCATCTGGAGAGGAGATGAGGACGATGTGGTAGTAGCGCCACCACCACCTACGACTACGACTACGACTACGACTAGGACGAACCTCCTTGTCGTGATGCCAGACCTCATAGACCTGGGGTTCTATGTCCCTGTCAGAGCCTTGTATCACAAGGGTAGAAGTAGAAATGGAAGCAGATGTGACAGCCTCGGCTGGGAGTACCTCCCTTCGCTACACAAAATCAACGTAGTTGTCGATTGTGATGGCGCCTACCATGACGATGTGGAGAAGGCAAGGGCTGAGATGAGGGAGGCAGCAAAACTCCATCCCAACCAACCCAGAATTCAGATTGAACTACGCGATGAATATAGGACGAGGTACTACTACACATCAACAGATACAGAGGTACCGTACTACGTACATTATTTATTTATTTATTTATTTATTTATTTATTTATTGTTATTACTACTATTATTCTAACACCAGTACATACATCTTTAAATGCTTCGATGACCTGTTCTCACAACCAATTCCAGTCGGACGACGACAACAATTTACCTGTACAAGAAAAGGCGACATCGTCTTTCAGCAGCTTCAATGGACTACAGACATAG

>CML103_Zm00021ab091790

ATGGAGTTTGCTACGGGTGCCATGGGTACCCTCCTCCCCAAGCTGGGCATGCTGCTGCAGGAAGAACTCCACCTGAAGAATAATGTGAAGGAGGGGATCAAGAGCCTCACTGCCGAGCTTGAGAGCATGCAAGCTGCACTTGTGAAGGTGTCTGACGTGCCATTAGACCAGCTTGACCCAAACGTCAAGATTTGGGCTAATGAAGTCAGGGGGCTGTCCTATGATATTGAGGACAGACTCGACTCCTTCAAGGTGCGCATGGAGGGTCTTGATTCAACCAAGCGCAAAACCATCATGGGATTCATCCAACAAACCCGTGGCTTGGTCACCAAGTTCAAGATTCGCCATGTAATATTTGATGACATTAAAGACTTCGGGAGCCAAGTAAAGGAGGTGAAGGAGCGGTATGACAGGTACAAGGTGCATGATGTTGTAGCTAATCCTATCGCAACCACAGTTGACCCTCGTCTCTTGGCTATGTATAACAAGGTTTCCGACCTTGTTGGCATTGACGAAGAAGCTAAGGAGCTAATGAATAATTTGTTTGAAGATGGTGACGAGCCAGCGAAAAAGATCAAGACAGTCTCTGTTGTTGGATTTGGAGGACTCGGCAAGACTACTCTTGTTAAAGCAGTCTATGACAAGGTTAAGAAGGAGTTTGATTGCAGTGCTTTTGTATCAATAGGTCAGAAATGTGATCTCAAGAAAGTTTTCAAGGACGTTCTTTATGATCTTGACAAGCAAAATCATGAAAATATCATTGCATCAGAAATGGATGAAAAACAACTCATTGATAAGCTACAGGAATTCCTTGCAGACAAGAGGTACTTGGTTGTTATTGATGACATATGGGATATATCAACATGGAAGCTGATTAGATGTGCTTTGGTGGAAAGTAACCCTGGAAGTAGAATAATCATAACTACTCGCATTTGTGAAGTTGCCAAAAAGGTTGGTGGTGTTTACAACAAGAAACCACTCTCTCTTGATGACTCCAAGACATTATTCTATACTAGAGTATTTGCTGGTGAAAGCATGAGTCTTGATAACATATCTGGTGAAGTGTGCAACAAAATCCTAAGAAAATGTGGTGGTGTGCCATTGTCCATCATTACGATAGCTAGTCTGCTTGTTGGTAAACAGAGGGAGGACTGGTCTAAGGTGTATGATTATATTGGTTTTGGGCATGAAGATAACGAGGTTATTGGGAACATGAGAAAGATATTAGCTTTCAGCTATTACAATCTACCTCCTTATCTAAAGACATGCTTATTGCACCTAAGCATATTTCCAGAAGATCACAAGATTGAGAAAAATTCATTGATATGGAGGTGGATAGCTGAAGGTTTTGTTATTGGCAGAGAAGAACTAGGGTTATTTGAGGTTGGAGAGAGCTATTTCAATGAGCTCATAAATAGAAGCATGATCCGGTGGATAGAGCTCTCTAGTAGAAGCAAGATTCGAGATGGTTGTGGTATTCATGATATGGTGCTTGATCTTATCCGCACTTTGTCAGGTGAAGTAAACTTGGTCACGGTATCAGATGTGGAGCAGCAGTGTACCACATCATCATCATATTCACCAGTCAGAAGCATTAGCGCTCGAAGATTAGCCTTCCACAAAAAAAGAAGCATTGAACACAACCCTGGCACAGAAATAGGACAGGTGAGATCATTCAATGCTTTCAACTGCTCTGGTAGTAGGATGCCCCGACTTTTAAGCTTCAGGGTCTTACGTGTACTAGCTCTGGAGAACTGTAATTTCTCAGCAGGAAACTGTTGCCTTGGAAATATTGGCAAATTGCATCAGCTGAGGTACCTAGGGCTAGTGGAGACATCCATTCGTGACGACTTGCTGCCTGGAGAAACAGGACGCCTCAAGTTTCTGCAGACACTAGATGTAAGACGAAGTGGCATAAAAATATTGCCAGCGTCTGTTGGTGAGCTAAGGAAACTGATGTGCCTGCGTGCTACCGAGGGCACAAGGATGATGGCCGAGATTGGGAAGCTGGCGTCACTGGAAGAGCTTGAGGTACACTCTGTGGACAAGTCGCCAAACTTCGCCACGGGGCTGGGGCAGCTAACCAAGGTGAGGGTGCTTGAGATCCATTTCGACGAAATGGACGAGAGCACAGAGAAGGCTCTCATGGAGTCCCTGCGCAACCTGCGGAAAATCCAGAGTCTGCAGATATGGTCCAAGAAGGAGAGGACAATTGACCTTGGCGGCTTGTTGGAAGACTGGACGCCAACCCCTTCAAATCTCCGTCAACTGATGCTGCGTGGCATCCATTTGCCCAGGCGTCCGTCATGGATTGATCCCTCATGTGTCCCGCTCCTCTCGTACTTGTCGCTCACGGTCCAGGCCGTGCAAGTGCAGGATCTAGAAATCCTCGGGAGGCTGCCGTTGCTCAGCTACCTCTACATCTGGAGTGAGGGCATCAACTGCTTATCCTATACTGCTACCAGCAGAGACGAGTTTCAGAATCTGAGATATCTGGACACAAACCTGGAGATCATGTGTGGACAGCAGGGAGCACTGCCTATGGTTGAGAAGTTGACATGCCGTGCCAGCATGGGGAAGTATGTTGCCTTCGCCAGGAGCAGCATGCCTTTCGACGATGGCAGCGTGGTGAATCCTGCTGCTGTTGCTGAGGCAGAATTGCCAGTAATTCTACCTTTGGACATTGGCTGGCCCGTGAACATGCCTTGCCTCCGGGGCATCACCTATTTGCTGGACTACCAGGACTGCAGTGCCAAGGAGTGGGCTCATGTGGAGACATTGCTCATGCACGTGAGAAAAATCCACCCCAACTGTCCACCCTTCCGAATCAAAAAGAACTGCAGAGACAAGGAGATTACTATGATCGACGCAATCATCTACCTTGAAGATGTCAAGGATGTGTTCAAGGACAACCCCTCCAAATACTCGGAGTTTCTTGATCTCTTGCAAGATTATAGGAGAGACAGAATCAAAATCAAAGATTTGATCATCCGTCTCAAGACCCTATTTACTGGACATGATCCTAATCTCATCCTTGACTTCAGCGTCTTCCTGCCCGGGGAGTGGGCCATCACTCTCGGGGACCTGTAG

>CML103_Zm00021ab091800

ATGGAGTTTGCTACGGGTGCCATGGGTACCCTCCTCCCCAAGCTGGGCATGCTGCTGCAGGAAGAACTCCACCTGAAGAATAATGTGAAGGAGGGGATCAAGAGCCTCACTGCCGAGCTTGAGAGCATGCAAGCTGCACTTGTGAAGGTGTCTGACGTGCCATTAGACCAGCTTGACCCAAACGTCAAGATTTGGGCTAATGAAGTCAGGGGGCTGTCCTATGATATTGAGGACAGACTCGACTCCTTCAAGGTGCGCATGGAGGGTCTTGATTCAACCAAGCGCAAAACCATCATGGGATTCATCCAACAAACCCGTGGCTTGGTCACCAAGTTCAAGATTCGCCATGTAATATTTGATGACATTAAAGACTTCGGGAGCCAAGTAAAGGAGGTGAAGGAGCGGTATGACAGGTACAAGGTGCATGATGTTGTAGCTAATCCTATCGCAACCACAGTTGACCCTCGTCTCTTGGCTATGTATAACAAGGTTTCCGACCTTGTTGGCATTGACGAAGAAGCTAAGGAGCTAATGAATAATTTGTTTGAAGATGGTGACGAGCCAGCGAAAAAGATCAAGACAGTCTCTGTTGTTGGATTTGGAGGACTCGGCAAGACTACTCTTGTTAAAGCAGTCTATGACAAGGTTAAGAAGGAGTTTGATTGCAGTGCTTTTGTATCAATAGGTCAGAAATGTGATCTCAAGAAAGTTTTCAAGGACGTTCTTTATGATCTTGACAAGCAAAATCATGAAAATATCATTGCATCAGAAATGGATGAAAAACAACTCATTGATAAGCTACAGGAATTCCTTGCAGACAAGAGGTACTTGGTTGTTATTGATGACATATGGGATATATCAACATGGAAGCTGATTAGATGTGCTTTGGTGGAAAGTAACCCTGGAAGTAGAATAATCATAACTACTCGCATTTGTGAAGTTGCCAAAAAGGTTGGTGGTGTTTACAACAAGAAACCACTCTCTCTTGATGACTCCAAGACATTATTCTATACTAGAGTATTTGCTGGTGAAAGCATGAGTCTTGATAACATATCTGGTGAAGTGTGCAACAAAATCCTAAGAAAATGTGGTGGTGTGCCATTGTCCATCATTACGATAGCTAGTCTGCTTGTTGGTAAACAGAGGGAGGACTGGTCTAAGGTGTATGATTATATTGGTTTTGGGCATGAAGATAACGAGGTTATTGGGAACATGAGAAAGATATTAGCTTTCAGCTATTACAATCTACCTCCTTATCTAAAGACATGCTTATTGCACCTAAGCATATTTCCAGAAGATCACAAGATTGAGAAAAATTCATTGATATGGAGGTGGATAGCTGAAGGTTTTGTTATTGGCAGAGAAGAACTAGGGTTATTTGAGGTTGGAGAGAGCTATTTCAATGAGCTCATAAATAGAAGCATGATCCGGTGGATAGAGCTCTCTAGTAGAAGCAAGATTCGAGATGGTTGTGGTATTCATGATATGGTGCTTGATCTTATCCGCACTTTGTCAGGTGAAGTAAACTTGGTCACGGTATCAGATGTGGAGCAGCAGTGTACCACATCATCATCATATTCACCAGTCAGAAGCATTAGCGCTCGAAGATTAGCCTTCCACAAAAAAAGAAGCATTGAACACAACCCTGGCACAGAAATAGGACAGGTGAGATCATTCAATGCTTTCAACTGCTCTGGTAGTAGGATGCCCCGACTTTTAAGCTTCAGGGTCTTACGTGTACTAGCTCTGGAGAACTGTAATTTCTCAGCAGGAAACTGTTGCCTTGGAAATATTGGCAAATTGCATCAGCTGAGGTACCTAGGGCTAGTGGAGACATCCATTCGTGACGACTTGCTGCCTGGAGAAACAGGACACCTCAAGTTTCTGCAGACACTAGATGTAAGACGAAGTGGCATAAAAATATTGCCAGCGTCTGTTGGTGAGCTAATGAAACTGATGTGCCTGCGTGCTACCGAGGGCACAAGGATGATGGCCGAGATTGGGAAGCTGGCGTCACTGGAAGAGCTTGAGGTACACTCTGTGGACAAGTCGCCAAACTTCGCCACGGGGCTGGGGCAGCTAACCAAGGTGAGGGTGCTTGAGATCCATTTCGACGAAATGGACGAGAGCACAGAGAAGGCTCTCATGGAGTCCCTGCGCAACCTGCGGAAAATCCAGAGTCTGCAGATATGGTCCAAGAAGGAGAGGACAATTGACCTTGGCGGCTTGTTGGAAGACTGGACGCCAACCCCTTCAGATCTCCGTCAACTGATGCTGTGTGGCATCCATTTGCCCAGGCGTCCGTCATGGATTGATCCCTCATGTGTCCCGCTCCTCTCGTACTTGTCGCTCACGGTCCAGGCCGTGCAAGTGCAGGATCTAGAAATCCTCGGGAGGCTGCCGTTGCTCAGCTACCTCTACATCTGGAGTGAGGGCATCAACTGCTTATCCTATACTGCTACCAGCAGAGACGAGTTTCAGAATCTGAGACATCTGGACACAAACCTGGAGATCATGTGTGGACAGCAGGGAGCACTGCCTATGGTTGAGAAGTTGACATGCCGTGCCAGCATGGGGAAGTATGTTGCCTTTGCCAGGAGCAGCATGCCTTTCGACGATGGCAGCGTGGTGAATCCTGCTACTGTTGCTGAGGCAGAATTGCCAGTAATTCTACCTTTGGACATTGGCTGGCCCGTGAACATGCCTTGCCTCCGGGGCATCACCTATTTGCTGGATTACCAGGACTGCAGTGCCAAGGAGTGGGCTCATGTGGAGACATTGCTCTTGCACGTGAGAAAAATCCACCCCAACTGTCCACCCTTCCGAATCAAAAAGAACTGCAGAGACAAGAAGATTACCTTGATCGACGCATTCAGCTACCTTGAAGCTGTCAAGGATGTGTTCAAGGGCAACCCCTCCAAATACTCGGAGTTTTTTGATCTCATGATTGATTACAAGAGAGACAGAATCAAAATCAAAGATGTGATCATCCGTCTCAAGACCCTATTTACTGGACATGATCCTAATCTCATCCTTGACTTCAGCGTCTTCCTGCCCAGGGAGTGGGCCATCACTCTCGGGGACCTGTAG

>CML103_Zm00021ab110580

ATGGCTGAACTAGCGATTAGCCTGGTGGTCGCCCCACTGGTGTCCCGGTTGAAGGAGAAGGCGTCCAGCTCTCTCCTCGACCAGTACAACGTGATGGAGGGTATGGAGAAGCACCACGAGACCCTCGTGCGCTGGCTGCCTCCCATCCTCAAAGTCATCACCGACGCCGAGAGGCAGGCATCTCGCCGCGGCGTGGAGAAATGGCTCGAGCAGCTCAAGACAGCGGTGTACGAGGCGAACGAGGTCTTCGACGACTTCGAGTACGAGGCGCTCCGCCGTCGAGCCAAGAAGAATGGGCACATCGCCGAGCTTGGCGTTATGACCAGTGTAAAACTCTTCCCTACCCACAATCGTGTTGCCTTCCATATCAGGATGGGCTATAGGCTTCGCAGAGTTGTTGACACCTTCAAGGACCTTATAAAAGAAATGGACACCTTTAGATTCAACAAGCTTGAGCCCGAGGCAACAACGGCACGGAAGGAGTTGCGTGAGATGGATTCCATTATCGTTGATCCTGAAAATATTGTTGCCAGGTCTAGAGACGATGAGAGGAAGAAAATTGTTAACATATTGGTCAATGGCCATGTTAACAGTCGTGATCTCATGGTCGTTCCCATTGTTGGAATGCCAGGACTAGGCAAGACCACCCTCGCTCAGCTCATCTACAAGGACCCTGAGGTCAAGGAGCATTTCCATCTACTAAAGTGGGTAACTGTGTCAGATGATTTTAGTGTTCTTAATCTTGCCAACAAGATATGTAATGCCTCGGAGAGAGTCCTCGAGGATGCAGTGAAGAAGCTTCAGGAACATCTTAAAGGAAAGAGGTACCTTCTTGTATTGGATGACGTCTGGAATAGGGATATTGATAAGTGGAGAAAGCTGAAGGCGTGTCTTATGCAAGGTATTGGTTGTGCCATATTAGTGACGACACGTGAACAACAAATAGCCCAGTTTATGGGTACTGTAGTTGACAGCTCATGGGCAAAAAGTTACCATGAAGTGGCAATTTTGGGCAAGGAATACATACAAGAAATTATTGAAACAAGAGCATTCAGTTCGCCGAAGAGCAAGTCAGATTATTTAGTTAAGTTGGCTGGTCTGATCACTGAGAGATGTGCGGGGTCTCCATTGGCAGCAAAAGCAATAGGGTCTGTACTGCGTAACAAGACCACTGATGGAGAATGGGAGGATGTGTTACAACGAAGCACCATATGTAATGATGAGACTGGAATTTTGCCTATACTCAAGCTTAGTTATAATGACTTGCCAAATGACATGAAGCAATGTTTTGCCTTTTGTGCTCTATATCCAAAGGATTATCATATTGATGTGGACAAACTTATCCAACTATGGATGGCCAATGGTTTTATCTCGGATCAAGAAAATGAACCTGCTGAAACCATAGGTAAAAGGATTGTCAATGAGATGGTCTCAAGGTCCTTTTTCCAATATGAGGAGCAAACTATGATCGGATACAATTCTACTACATTTTTGAAGATTCATGACCTCATGCAGGAGGTTGCACTGTCTGTTTCGGAAAAGGAGTGTGCTTGTATAACGGATAAATTCATTACAAATAGTGAGTTGCTTCCAAGTGCTGCCCGCCACATACTTATTCAAACATGGAGTAACAAACGGATACATGGTTATTTATATGGTTTTATGAGGAAATTGTCTCGACCTATCCAAACATTGATGTTTGATGGGTCTTGTGAAGATGCCGTTGTGCAACATTTATCAAGACATAGTTCTTTGCGAGTACTTTCCATGCCAGGATTTTGGTTTCGTTTTCCAATAAAACCAAAGCATATGTGCCACCTTAGGTTCCTTGATGTCACGGGTAGTAGAATCAAAGAACTTCCATATGACACAAGCATCCTTTATAATCTTCAGACACTCAAACTTTCTGGATGCAGGAATCTTATTAGACTTCCTGAGCAAATGAAGCACATGAGTGCCCTTCGTCATCTCTACACAGATGGGTGCACAAGGTTGGAGTGCATGCCTCCAGATCTTGGACAAATCACCTCGCTTCGAACAATTACATGGTTTGTAGTGGGAAGTGGCTTGAGTTGTAGTAGCCTTGGAGAGCTAAGGGATTTAAATATTGGTGGCTCATTAATGCTAAAGCAGCTCGAAAATGTGACAGGGAGAAGAAATGCAGAAGCAGCCAAACTTGAGAATAAGAAGGAACTGAGACAACTGTCACTAGAGTGGACAAGTGGTAAGGAGGAGGAACAACAGTGTCATGAGGTGCTAGAGAGCCTTGAAGCTCATGATGGACTGTTGGCTCTAGAAATATATTCCTACCAAGGCACCCGTTTTCCATCTTGGATGGGTATGTTGAAAAACATACTCGAGCTTCGGTTGTTCGATTGTTGTAAAGTAGAGCAGCTTCCACCACTGTGTCAACTAGCAGAACTGCAACTCCTTCATTTGAAAAGATTGGGAAATTTGCGGTTCCTGTGTAGCAGATGTACATCCTCCACATTTGGAAAGCTTAAGGATCTTAAGCTAGTTGATCTTCACGTTTTTGAAGGATTTTGTAAGACAATGCATGGATCTACAGTAGCATTCCCTCAGCTTGAGATATTGCACATTGAGCGCTGTGGAAATCTAGCAGCTCTAACAGAAGCATCGCATTGCGGTGGAGATTATACAGTGGCCCGCTCAACATTTCCAGAACTGAAGAGGCTCATATTGGAAGATTTGTGTAGCTTTGAGAGATGGGTGGCTGGCCTGCTTGAAATTGAAGAAGAACATGCACTATTCCCTGTGGTTGAGATTGTTGTTATTAGTAAATGCCCAAAGTTAACAACTGTGCCTAGGGCACCGAAGGTCAAAGAACTAGTTTTGCGTGATGTACATGCACATATCTCTCTAGGAGGGATCAGATGTATGACGTCATTGTCCACTTTGCTTTTGGACGGCGTGAAGCTTGATGTTAAGGAGAGATGGGACCATCCATCGTCCGTGGTAGATATGCAACTGTGGAGGTGCAGTTTGTTCTTCCAACCACGCGCACTAGTGATGTGGGTTTGCTATTGGCAGCTGCAAGATCTGACAATTTATAGATGTGATGAGCTTGTGTACTGGCCGGAGAAAGTATTCCAAAGCTTGGTTTCTCTGCGGAGGCTATGGATTGGCAATTGCAAGAACCTAATTGGATATGCAGCGGCGAATGTCCCCGACCAGGCAACTTCTGGAAGGAGCGAGCTTTTGCCCCATCTAGAGTATCTGGAGATATGGGGGTGCCAAAATCTGGTAGAGTTATTCAACTCCTCCCCTGCTCTCAAGAGAATGGAAGTTAGAGAATGTTGTAAGCTTGAGTCCCTATATGGCAAGCAATTGTTGGATGAAGCTGCTAGTAGTACCGATGACGTGACGGCATCCGCACATGTTGAGGAGAAGCTATCACCATCATCCCTAGAATCTCTAACAATATTGGACTGTGACAGGTTGTCAGAGGTTGTCAATCTTCCTTCGTCTCTCAGGGTAATAGATATTCAGGGTTGCTTCAAACTACGGCTCATGTCAGGGCAGCTGGATGCACTCAATACCTTAGCAATCACCAACTGCCCGGAGTTGCGATCACTGGAAACATGCATCGTAGATCTCACGTCAATGGAAATCCTCGCTCTGTGTGGTTGCAAAAGCTTGGCGTCCTTGCCTAGTGCGTGGGCAGGACGACAAGAATATTCATCTCTCCGTCAGCTTACGATTAGGGAGTGCCCAGGTATAAAATCGTTGCCTTCAACTCTGCAGCAGCGACTGGACAACGGCCTCTTGGATTTTACGAACCTAGATTCCCGTCGTCATGAAGATCGGCCTCCACGACGGCTGCTTGGGTGTCTCTTCCTCCTGCCTTTATTTTGTCTGGTCTCAAGGGCCAAATGTTGCTTTGTCTCTGAT

>CML103_Zm00021ab114170

ATGGCGGAGATAGTCACCGGGGCGATGGGCACTCTCCTGCCCAAGCTGGCCAACCTGATCAAGGAGGAGTATAACCTGCAGAAGAAGGTGAGGGGTGAGATCATGTTCCTGGAGGCTGAGCTCAAGAGCATGGAGGCTGCTCTCATCAAGGTCTCCGAGGCACCCATCGACCACCCACCTGACATCCAAGTCAAGCTCTGGACAAGGGAGGTGAGAGAGCTGTCCTACGACCTCGAGGACAGCATCGACAGATTCATGGTGCGAGTTGGCGATGGCAAGCCACATAGTTTCAAGGGATTCATTGATAGAAGCCTCCACCTGCTGACAAGGGGCAGGATTCAACACAGCATCGGCATAGACATCAAGGAGATCAGGAGCCGCATAAAGGATGTGAGTGAACGGCGTGACAGGTACAAGGTTGATCTGGTTCCTTCCAAGCCTGTTGGCAGAAGCATCGACAACCTGCGGCTGTCGGCTCTTTACAGAAAGGCGACAGAACTTGTTGGCGCCGAAGAGAAGAGCAGTGGCCTTGTGAGAAGGCTCATGGAGGGCGACAAGGAGGCATCCAAGCAGCCAGTTGTACTGTCTATTGCTGGCTTTGGAGGGTTAGGCAAGACTACTCTTGCTAATCTTGTATATGAGAAGATTAAAGGGCAATTTGGCTGTGGGGCATTTGTTTATGTGTCTCATAATCCTGATGTCGTCAAGGTTTTCAAAAACATGCTCTACCAGCTTGATGGAGACAAATACAGGGACATCAATCAAGGAACATGGAGTGAAGAACAACTAATCTGGGAACTGAGGAAGTTCCTTCTGCACAAGAGGTACTTCATTGTCATTGATGACATATGGAATACTTCTGTGTGGGAAACAATCCAATGTTCTTTGATGCACAATGAATGTGGAAGTATAATAATTATCACAACTCGTAATATTGATGTTGCAAAACAAGCTGGAAGTGTTTATCAAATGGAACCTCTTTCTCTCAGTGACTCAACAAAGTTATTCTGCCAAAGAATTTTTGGCAGTGAAGACAAATGTCCTCCAGATAATTTAGCTGAAGTGGCTGGTAAAATCTTACAGAAATGTGGTGGTGTACCATTAGCTATCATTACCATGGCAAGTATGCTAGCCGATAAAACTGGAAAGGAAATAAATACACATAACTATTGGTCACATGTGTACCAATCCATGGGTTCTGGTCTAAATGGCAGTACTAATGTGAAGAATATGAGAAGGATACTATCAGTTAGTTATTATGACCTACCTTCACATCTAAAGACTTGCTTGCTATACCTAAGTTTGTTTCCAGAAGACTACAGAATTAAAACAAGAGGTCTCATATGGAAATGGATTGGTGAAGGTTTTGTCCATGAAGAACAGGGAAAGACCTTATATGAAGTAGGTGAGGATTACATCGAAGAGTTAATTAACAGAAGTATGTTGGAACCTGTAGATATTGGCCGTGATGGTAAGACTGTTTCTTGTCGGATACATGATATGGTCCTTGATCTTATCAGTTTCTTGTCAAATGAGGAGCATTTTCTAACAAAAGTAGGTGAGCAACAGCCCATATCTCTTGATCTGCCTAAAAAGATCCACCGGTTATCCCTCCAAATTAGCCAGGAAGAGGAAGTCAAGCAGCTGGCTACAATGAGTTTCTCCCACGTAAGATCACTTACTGTGTCCACTGAAGTGTTCCAGTTGATGCCAAAACTTTCGGCCTTTCTGGTCTTACGTGTATTGAATTTAAAGAAATGTAAGGGAGTGAGGAATCACCACTTTAAAGATATTTGCAATATGTTTCACCTGAGATATTTGAGTCTCAATGCGGAATTTATTACTGAGATGCCACGGGAGATTCAGAATCTACAATTTTTGCAAGTACTTGACATAAGTAATCTTGGGCACAAAGTAAAGATGCCAACCATTATTCACTTGCGACAGCTACTGCGTCTTTGTTTTAGGCCGATGTGGGGCATAAGACTGCCAGATGGATTCGGAAAACTAACCTCTCTACAAGAAGTTAAAGGGATCATAACTATCAAGTCACCAAGCATGCTGCATAATCTGGGGTGTCTGACCAATCTCCGGACCTTGGCCATCGACTTTTGTGATTGGGATGAGAGCTATGAGGAACCTTTCATCCAATGTCTATCTAACCTTGTCAGCCTCAAATCCATGGAAATAAAAGGTACCATGGTGAGCAGCCTATGTTCCGAATGTGACAAATTGTACCCTGGTCCTCAACATCTTTGCTCCATTGATATTGAGTCGACTGCAGTGCCAAGATGGATGTCATCGCTCTGCTTCTTGTCTAGCATAAACATTGAACTATTAGCTCTGGGAGCACAGGATTTTCATGTCCTTGGGAGCATACCATCTCTACGTTGTCTCAGTATACATGTGAAGGAAACCAGAGATGAAAGATTGGTCATTGGCAAGTGTTATCCATTCAGGTGCCTAACTGAGATGCAAATCGATTATGAATCCATGGCGGTGGTGTTCGCACCAGGAAGTATGCAAAACCTCAAAGAACTTCATTTAGTGTTCGGGGTGAAAGAGGTAATGCATAAGTATGGTGATTGTAACTTTGGTTTGGAGCACCTCATGTCACTGGAGCATGTCTCTGTTAAAACAATGTACAGTATCATGCCCGAGGAGGTGGAGGCCGTAAAAGATGAATTTCAGAAATCCCTGGACATGAATCCTGGCAAGCCCACGTTGATAGTAGATTATAAGTATCCGATAAAAAGGAAGATTAGGTCTCATGCACAAGCAATAAGAGCAGCAATTTTGTTCGCTAATGCAGGCCGCATCCCTGCTACTGAAGGATTATAA

>CML103_Zm00021ab114420
[truncated: 4,050,641 more chars]
